# Somatic Double Inactivation of *NF1* Associated with NF1-Related Pectus Excavatum Deformity

**DOI:** 10.1155/2023/3160653

**Published:** 2023-04-28

**Authors:** Cristina Chelleri, Marcello Scala, Patrizia De Marco, Vittorio Guerriero, Marzia Ognibene, Francesca Madia, Sara Guerrisi, Marco Di Duca, Michele Torre, Serena Tamburro, Paolo Scudieri, Gianluca Piccolo, Girolamo Mattioli, Francesca Buffelli, Paolo Uva, Diego Vozzi, Ezio Fulcheri, Pasquale Striano, Maria Cristina Diana, Federico Zara

**Affiliations:** ^1^Pediatric Neurology and Neuromuscular Disorders Unit, IRCCS Istituto Giannina Gaslini, Genoa, Italy; ^2^Department of Neurosciences, Rehabilitation, Ophthalmology, Genetics, Maternal and Child Health (DINOGMI), University of Genoa, Genoa, Italy; ^3^Medical Genetics Unit, IRCCS Istituto Giannina Gaslini, Genoa, Italy; ^4^Pediatric Thoracic and Airway Surgery Unit, IRCCS Istituto Giannina Gaslini, Genoa, Italy; ^5^Pediatric Surgery Unit, IRCCS Istituto Giannina Gaslini, Genoa, Italy; ^6^Clinical Pathology Unit, IRCCS Istituto Giannina Gaslini, Italy; ^7^Genomic Facility, Istituto Italiano di Tecnologia, Genova, Italy; ^8^Unità di Bioinformatica Clinica, Direzione Scientifica, IRCCS Istituto Giannina Gaslini, Genoa, Italy

## Abstract

Neurofibromatosis type 1 (NF1) is a neurocutaneous genetic disorder with a broad spectrum of associated signs and symptoms, including skeletal anomalies. The association of NF1 with anterior chest wall deformities has been recently reported, especially the pectus excavatum (PE). Over the years, several authors have suggested loss of heterozygosity (LOH) as the possible pathogenic mechanism underlying the development of the typical NF1 skeletal features. Here, we report a NF1 patient with severe chest deformity and harboring the germline heterozygous pathogenic *NF1* variant NM_001042492.3: c.4271delC p.(Ala1424Glufs^∗^4). Through next-generation sequencing (NGS), we investigated the affected cartilage from the PE deformity and identified the additional frameshift variant NM_001042492.3: c.2953delC p.(Gln985Lysfs^∗^7), occurring as a somatic NF1 second hit mutation. Exome sequencing confirmed the absence of additional variants of potential pathogenic relevance. Western blot analysis showed the absence of wild-type NF1 protein in the cartilage of the patient, consistent with a somatic double inactivation (SDI) of *NF1*. Taken together, our findings support the role of SDI in NF1-related PE, widening the spectrum of the pathophysiological mechanisms involved in NF1-related skeletal features.

## 1. Introduction

Neurofibromatosis type 1 (NF1) is a complex neurocutaneous condition caused by pathogenic variants in the NF1 gene (OMIM ^∗^ 613113) and characterized by a heterogeneous clinical presentation [1, 2]. So far, genotype-phenotype correlations remain elusive in NF1, and this is especially true for skeletal abnormalities, although these lesions are common in NF1 patients [2]. NF1-related skeletal defects include a broad range of manifestations, such as osteoporosis, short stature, dysplasia of the tibia and other long bones, vertebral defects, sphenoid wing dysplasia, and anterior chest wall deformities [3]. Of note, the latter have been only recently reported in association with NF1, especially *pectus excavatum* (PE) [3]. This consists of a depression in the anterior chest wall resulting from a dorsal deviation of the sternal bone and 3^rd^-7^th^ rib or costal cartilage [4, 5]. PE is the most common chest wall deformity (90% of all cases) and has been recently found to be especially frequent in NF1 patients, with a higher incidence as compared to the general population [6–8].

The pathogenic mechanism underlying NF1-related skeletal abnormalities remains elusive. Previous studies have suggested that the somatic loss of heterozygosity (LOH) in the *NF1* gene may contribute to the development of these defects [9–12]. This mechanism is relevant for carcinogenesis, and LOH of essential genes accounts for potential cancer vulnerabilities [13]. LOH of *NF1* has been identified in cells extracted from skeletal and nervous tissues in affected individuals, such as tibial pseudoarthrosis, dystrophic scoliosis, or plexiform neurofibroma samples [9–12]. Furthermore, mouse models have been found to recapitulate in part the bone abnormalities observed in NF1 patients, helping clarify that *NF1* haploinsufficiency accounts for the generalized bone remodeling defects, while the complete loss due to LOH is responsible for the focal defects, such as the dysplasia [3, 12, 13].

In this study, we thoroughly investigated a cartilage sample from a PE deformity in a subject with NF1 caused by a germinal NF1 frameshift variant. The combination of different techniques allowed us to identify a somatic double inactivation (SDI) of NF1 in the affected cartilage leading to the complete absence of the wild-type NF1 protein.

## 2. Materials and Methods

### 2.1. Ethical Approval and Clinical Examination

The study was conducted in accordance with the Declaration of Helsinki, and ethical approval was obtained by the “Comitato Unico Regionale Regione Liguria,” Genoa, Italy. Informed consent was waived for this study as all clinical and radiological information has been anonymized. The patient was thoroughly evaluated through specialistic pediatric and surgical assessment. Imaging studies were reviewed by experts in thoracic disorders of childhood.

### 2.2. Biopsy and Pathology Examination

A sample of affected cartilage was obtained from the central core of the tissue corresponding to the PE deformity. After surgery, a cylindrical fragment of discarded cartilage measuring 1 × 1 cm was frozen to allow genetic and histoenzymatic investigations. It was then thawed, fixed in 10% buffered formalin, and embedded in paraffin in two blocks [14]. Histochemical stains were performed using PAS (AB pH 2.5; AB pH 1).

### 2.3. Next-Generation Sequencing

DNA was extracted from peripheral blood samples and from discarded surgical frozen tissue using commercial kits. A next-generation sequencing (NGS) custom-designed panel was created using the Ion AmpliSeq™ Designer v6.13 algorithm provided by Thermo Fisher Scientific (Carlsbad, CA, USA) in order to target the entire coding sequence (CDS) and 10 bases of the adjacent intronic regions of NF1 gene (NM_001042492.3). NGS was performed on genomic DNA extracted from both blood and cartilaginous biopsies using the Ion Gene Studio S5 platform (Thermo Fisher Scientific, Inc.). Variants were analyzed using both the Ion Reporter Software v.5.6 (Thermo Fisher Scientific, Inc.) and the CLC Genomics Workbench 6.5.1 software (Qiagen).

### 2.4. Multiplex Ligation-Dependent Probe Amplification

Multiplex ligation-dependent probe amplification (MLPA) was performed in order to detect somatic exonic deletions of NF1 using two commercial kits, the SALSA P081/P082 kits (MRC-Holland, Amsterdam, the Netherlands). Both cartilage and peripheral blood were also tested by high-resolution oligonucleotide array CGH (comparative genomic hybridization) using the 4 × 180 K Kit, with probe design 086332 (Agilent 119 Technologies, Santa Clara, CA), according to the manufacturer's instructions, in order to detect large somatic LOH inclusive of NF1.

### 2.5. Exome Sequencing

After standard DNA extraction, exome sequencing (ES) was performed according to local protocol. The quality of the sequence reads was assessed by generating QC statistics with FastQC (http://www.bioinformatics.bbsrc.ac.uk/projects/fastqc). BWA with default parameters was used for the read alignment to the reference human genome (hg38, UCSC assembly). HaplotypeCaller algorithm in the GATK package was used for quality score recalibration, indel realignment, and variant calling. Variants were annotated with ANNOVAR and filtered for population genetics, conservation (GERP), and impact on the protein function according to *in silico* tools as previously described [15]. Variants with a minor allele frequency (MAF) > 0.01 in control databases (internal database of 4,500 exomes and gnomAD) were excluded.

### 2.6. Western Blot

NF1 frameshift mutations that generate premature termination codons (PTCs) lead to the synthesis of truncated neurofibromin. To study the effects of NF1 causative mutations at the protein level on the pathological tissue, we performed a western blot (WB) analysis. Fragments of the abnormal and autoptic control cartilage samples were pulverized in liquid nitrogen and lysed and analyzed by WB to evaluate NF1 protein expression (Supplementary Material (available here)). Using a primary Ab against the N-terminus domains of neurofibromin, a specific band of about 260 kDa was detected in the total lysates from control cartilage samples.

## 3. Results

### 3.1. Clinical Study

**Figure 1:**
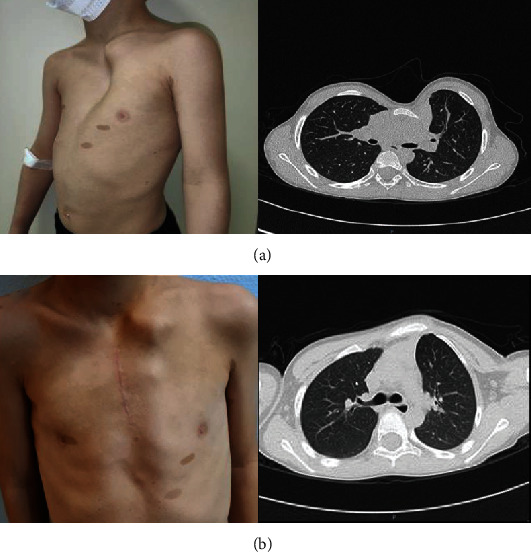
Clinical and imaging findings. Clinical photographs and axial CT scans of the chest of the reported individual before (a) and after (b) surgical intervention. The patient had a severe deformity of the anterior chest wall and some typical cafè-au-lait macules (a). The PE deformity consisted of a severe dorsal deviation of the sternal manubrium and costal cartilage, as shown by the preoperative CT scan (a). After surgery, a significant correction of the PE deformity was achieved. This result could be appreciated at the clinical exam and was confirmed by follow-up CT scan (b).

This is a 4-year-old patient of Italian ancestry, with a negative family history for NF1 and neurodevelopmental disorders. The patient was diagnosed with NF1 at the age of 2 years, when he presented with cafè-au-lait macules and Lisch nodules. Over the years, he developed a plexiform neurofibroma in the left popliteal fossa, and brain MRI at the age of 5 years showed unidentified bright objects (UBOs), thus confirming a clinical NF1 diagnosis. At the age of 4 years, the patient was referred to our institution for a rapidly enlarging deformity of the anterior chest wall. Clinical examination revealed a severe PE deformity, consisting of a severe depression of the sternal manubrium associated with marked thoracic asymmetry and marked protrusion of the left costal cartilages (Figure 1). The Haller index was calculated to be 4.3 (pathologic over 3.25). Thoracic CT scan confirmed the severe bony depression between the manubrium and the body of the sternum, which led to the compression of the right ventricle and supra-aortic vessels (Figure 1) (Figure S1). The patient underwent a major elective surgery combining minimal invasive repair of PE/MIRPE (Nuss procedure) and open reconstruction of thoracic wall, with good overall outcome.

### 3.2. Pathology

**Figure 2:**
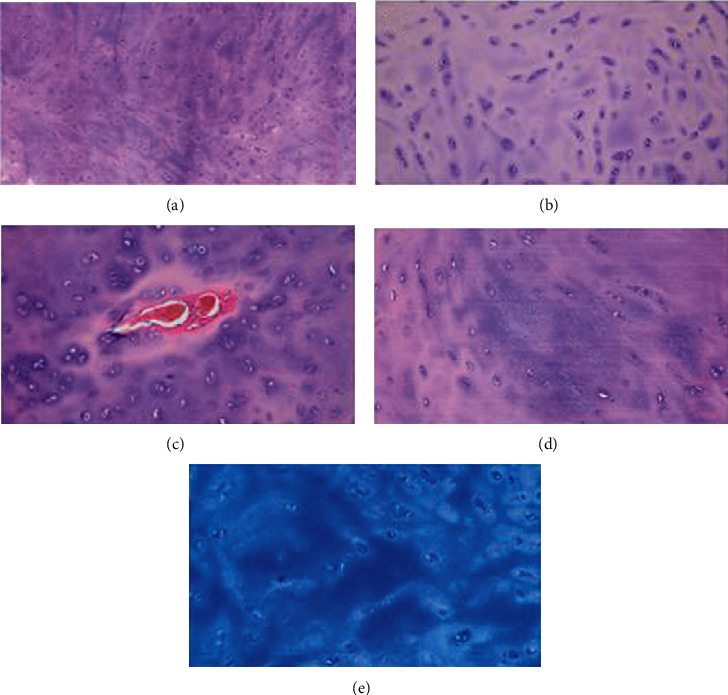
Pathology analysis. (a) The cartilage extracted from the PE deformity is of mature type. (b) The chondrocytes appear lightly more immature at the periphery. (c) The endochondral vascularization network is normal. (d, e) The isogenic groups are distributed in an abundant amorphous matrix (AB pH 2.5).

The histological examination revealed a mature type cartilage (Ki67-negative) with isogenic groups distributed in an abundant amorphous matrix (Figure 2). The endochondral vascularization network was normal. A nucleus of accentuated condrification with initial degeneration of the matrix around the largest chondrocytes with prominent nucleus was seen. The intercellular matrix, where more abundant, presented accentuated basophilia suggestive of mild degenerative phenomena (Figure 2). Histochemical stains (PAS; AB pH 2.5; AB pH 1) confirmed the presence of outbreaks of the mentioned degeneration with excessive accumulations of acidic mucopolysaccharides (chondroitin sulphate). The chondrocytes appeared slightly immature at the periphery but lacked fetal cartilage features, which was also confirmed by immunohistochemical stains.

### 3.3. Genetic Investigation

**Figure 3:**
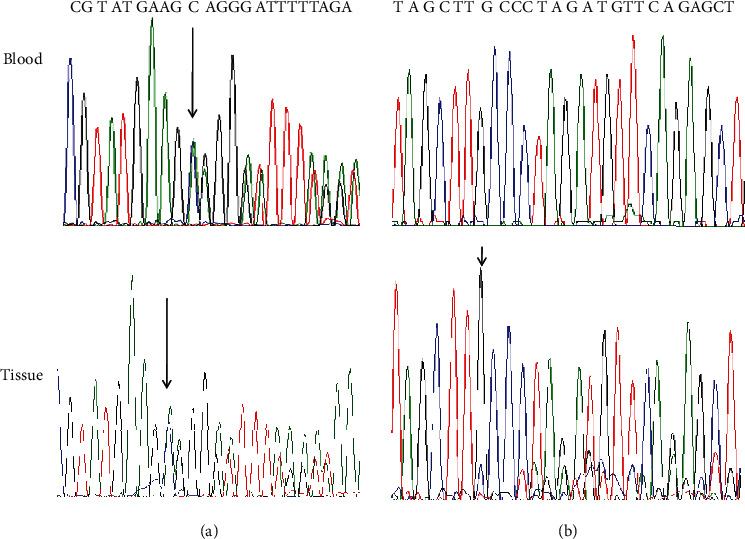
Electropherogram traces of variants confirmed by Sanger. Sanger traces of (a) germline heterozygous *NF1* c.4271delC variant present both in the blood and in the tissue and (b) somatic NF1 c.2953delC (allelic frequency 18%) present only in the pathological tissue sample (reverse sequence).

We identified a germline pathogenic variant c.4271delC p.(Ala1424Glufs^∗^4) present both in blood (variant allele frequency 48%, coverage 341X) and in the pathological tissue (variant allele frequency 38%; coverage 531X). This variant is absent in gnomAD and considered pathogenic according to the ACMG/AMP guidelines, as it is predicted to lead to a truncated transcript or nonsense-mediated mRNA decay (NMD). In the tissue, we identified a somatic second hit in NF1, the c.2953delC p.(Gln985Lysfs^∗^7), with a variant allelic frequency of 18%. This variant, a frameshift resulting in a stop gain 7 codons after the mutation, is not present in public databases, and it is predicted to be “likely pathogenic.” Both the germline and the somatic variants were validated by Sanger sequencing (Figure 3). The MLPA assays excluded somatic loss of the *NF1* wild-type allele by copy number variations (data not shown). These data therefore support the assumption that the pectus excavatum of this patient was a manifestation of NF1 and was driven by a copy neutral SDI in the pathological tissue. Exome sequencing on peripheral blood confirmed the presence of the p.(Ala1424Glufs^∗^4) variant and did not reveal variants of potential pathogenic relevance in other genes (Table S1).

### 3.4. Western Blot

**Figure 4:**
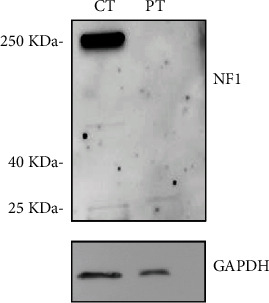
Western blot analysis of neurofibromin. WB images showing immunodetection of NF1 (top) and GAPDH (bottom, as a loading control) in total lysates from control (CT) and patient cartilage samples (PT). Control cartilage sample was obtained from autoptic biopsy of a sex- and age-matched control. In the patient's cartilage, no signal was detected, consistent with a complete loss of NF1 protein expression.

No signal was instead detected in the patient's cartilage, demonstrating a complete loss of wild-type neurofibromin (Figure 4). The theoretical molecular weight estimated for the two truncated form of neurofibromin resulting from the mutated alleles of our patient was 108 and 164 kDa. However, no truncated protein was detected in the assay. It is likely that mutations determining mRNAs with PTCs can render the mutant transcripts susceptible to degradation by the nonsense-mediated mRNA decay machinery, rendering the protein undetectable.

## 4. Discussion

Skeletal manifestations are particularly common and heterogeneous in NF1 patients [3]. Of note, in comparison to the general population, these subjects show a higher incidence of anterior chest wall deformities, especially PE [6], even in the pediatric age range [7]. However, the pathogenic mechanisms underlying these developmental abnormalities remained elusive. LOH of *NF1* has been implicated in other NF1 manifestations. In 2009, Steinmann et al. published the results of a LOH analysis study conducted on 43 plexiform neurofibromas from 31 NF1 patients [11]. The authors observed LOH involving 17q markers in a total of 13 (30%) tumors [11]. Some evidence in favor of the role of a somatic LOH in the pathogenesis of NF1-related skeletal manifestations has been provided by a few previous studies, which focused on the possible role of the double inactivation of the *NF1* gene in the affected tissues [3].

In 2006, Stevenson et al. reported two cases of tibial pseudoarthrosis associated with a double *NF1* inactivation [9]. This was caused by the combination of germinal stop gain variants (NM_000267.3: c.2446C>T (p.Arg816^∗^) and c.7846C>T (p.Arg2616^∗^)) and somatic LOH of *NF1* in the abnormal tissue of affected individuals, as suggested by allele imbalance [9]. The authors concluded that the loss of *NF1* function causes a dysregulation of the *Ras* pathway, which leads to an impairment of the differentiation and proliferation of osteoblasts and osteoprogenitors [9]. In a further study, the somatic LOH for most of the 17q region was detected in spinal samples from two NF1 patients with dystrophic scoliosis, who harbored germinal splicing (NM_000267.3: c.6642-1G>T or frameshift (NM_000267.3: c.4953_4965del (p.Asp1651Glufs^∗^22)) variants [10].

The role of LOH in relation to NF1-related skeletal manifestations was also investigated through animal models. Wang et al. focused on the study of the role of the loss of *NF1* in osteo-chondro progenitors in the development of *NF1*-related skeletal manifestations [12]. Crossing the *Col2a1*(collagen, type II, alpha 1)*-Cre* promoter mouse with the *Nf1^flox/flox^* mouse, the authors found that the *Nf1/Col2*^−/−^ mice showed progressive scoliosis, tibial pseudoarthrosis, and skeletal abnormalities involving the skull and anterior chest wall, demonstrating that loss of *Nf1* in axial and appendicular osteo-chondro progenitors recapitulates the skeletal abnormalities of NF1 patients [12]. Taken together, these findings suggest that the SDI of NF1 resulting from somatic LOH may be involved in the pathogenesis of NF1-related skeletal abnormalities, although this should be still considered multifactorial [3].

In our patient, we detected the *de novo* germline frameshift variant p.(Ala1424Glufs^∗^4) in *NF1* and a second, somatic, and *de novo* frameshift variant p.(Gln985Lysfs^∗^7). Although it was not possible to assess if this second variant occurred in *cis* or in *trans* to the germline variant, the western blot revealed absence of wild-type neurofibromin in the abnormal tissue. This stands in favor of the occurrence of the second variant in the wild-type allele of *NF1*, thus in trans with the germline variant. This finding confirms the occurrence of SDI of *NF1* in the affected cartilage. Accordingly, in line with previous studies about other NF1-related skeletal features, our observation further supports the role of SDI, either as a result of LOH or somatic second hit, in the pathogenesis of PE in NF1 patients. Whether the Ras/Erk constitutive activation caused by the complete loss of NF1 function or additional molecular mechanisms are implicated in the development of these skeletal alterations remains to be elucidated [3, 12]. Further studies will play a crucial role in the clarification of this aspect. However, our observations confirm that somatic double hits may be critically involved in the development of diverse clinical manifestations NF1, leading to suggest that the adoption of radiation-sparing approaches might be advisable in the diagnosis and management of affected individuals.

Our study had some limitations related to the availability and processing of the abnormal tissue analyzed. First, the cartilage samples were obtained from discarded tissue from surgical procedures, which was initially stored for future studies. Thus, the identification of the affected tissue relied on the judgment of surgeons and pathologists and was based on the labels indicated on specimen containers. Second, the DNA employed for genetic studies was extracted from frozen heterogeneous samples containing a mixed cellular population. Then, we specifically obtained pathological tissue from the central core of the abnormal cartilage of the pectus excavatum, and, therefore, we were not able to investigate cells from the peripheral regions of the malformation. Since the second variant occurred as a somatic change, the abnormal tissue is predicted to be a mosaic, and it is possible that other cells within the PE have traces of wild-type NF1. Indeed, while NGS was performed in cells from peripheral regions, the WB analysis was performed on tissue from the core of the malformation, to allow a more precise detection of NF1 in abnormal tissue. Here, the somatic mutation rate is predicted to be much higher (potentially ≈100%), explaining the finding of complete absence of wt NF1 protein (Figure S2). Unfortunately, no additional material was available to investigate the peripheral regions of the PE deformity through WB. Eventually, although WB results stand in favor of the SDI of *NF1* in our patient, we could not directly investigate the phase of the two variants in NF1, providing evidence of their position in *trans*.

## 5. Conclusion

In this study, we detected a somatic double inactivation of the *NF1* gene in the cartilage tissue from a PE deformity in a subject with NF1 due to a germline frameshift variant. Tissue analysis revealed that this neutral LOH was due to a somatic double hit frameshift variant resulting in a premature termination codon. Accordingly, the biallelic inactivation of *NF1* resulted in no wild-type neurofibromin in the affected tissue. In line with the limited previous studies, our findings suggest that SDI of *NF1* is a relevant contributing factor in the pathogenesis of skeletal abnormalities in NF1, including anterior chest wall deformities. Considering the possible overlapping pathophysiological mechanisms, this would lead to speculate about the possible use of MEK inhibitors in NF1 patients with severe skeletal abnormalities, which demonstrated effective in patients with NF1-associated tumors and plexiform neurofibromas. Further studies will be crucial to delineate the underlying pathogenic mechanisms and shed light on the possible involvement of similar alterations in other NF1-related extraneurological manifestations.

## Data Availability

There is an absence of shared data.

## Supplementary Materials

Supplementary Materials Supplementary Methods: description of western blot analysis 2. Figure S1: 3D reconstruction of the thoracic malformation. Figure S2: schematic representation of our study. In our patient, the abnormal tissue within the PE deformity was found to harbor a somatic NF1 variant as a second hit. While NGS was performed in a more peripheral region, thus leading to the detection of the variant in a mosaic of ≈18% of reads, the WB targeted the core of the malformation, where the rate of the somatic mutation is expected to be much higher, and detected no evident wt NF1 protein.
