# Supplementary material for: Somatic Double Inactivation of NF1 Associated with NF1-Related Pectus Excavatum Deformity
Source: Hum Mutat. 2023 Apr 28;2023:3160653. doi: 10.1155/2023/3160653 (PMC11918561; doi:10.1155/2023/3160653)
Supplement: Supplementary Materials — Supplementary Methods: description of western blot analysis 2. Figure S1: 3D reconstruction of the thoracic malformation. Figure S2: schematic representation of our study. In our patient, the abnormal tissue within the PE deformity was found to harbor a somatic NF1 variant as a second hit. While NGS was performed in a more peripheral region, thus leading to the detection of the variant in a mosaic of ≈18% of reads, the WB targeted the core of the malformation, where the rate of the somatic mutation is expected to be much higher, and detected no evident wt NF1 protein. [file 3160653.f1.zip › Figure S2.pptx]

## Slide 1
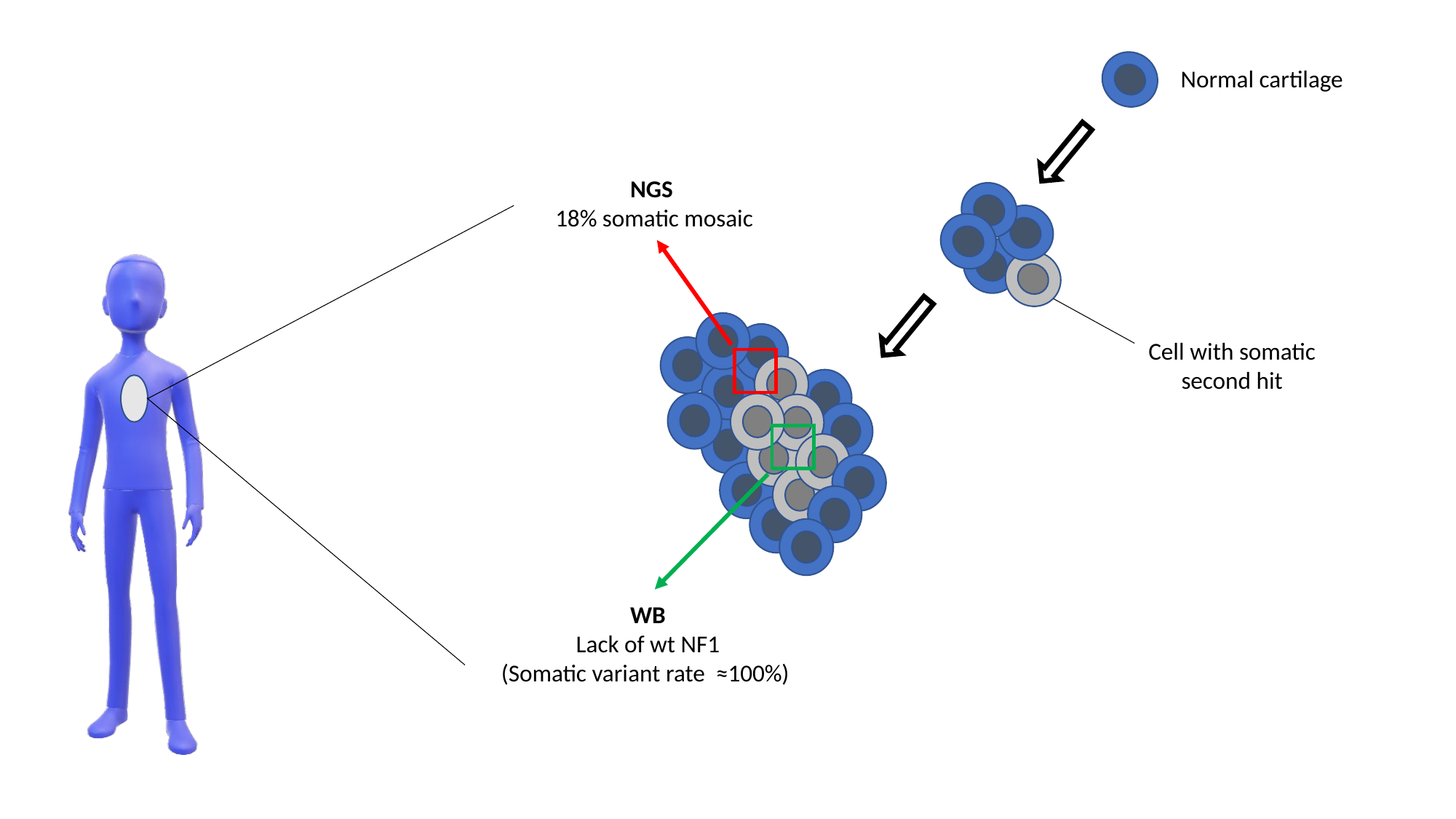

Normal cartilage
NGS
18% somatic mosaic
Cell with somatic second hit
WB
Lack of wt NF1
(Somatic variant rate  ≈100%)
