# Supplementary material for: Somatic Double Inactivation of NF1 Associated with NF1-Related Pectus Excavatum Deformity
Source: Hum Mutat. 2023 Apr 28;2023:3160653. doi: 10.1155/2023/3160653 (PMC11918561; doi:10.1155/2023/3160653)
Supplement: Supplementary Materials — Supplementary Methods: description of western blot analysis 2. Figure S1: 3D reconstruction of the thoracic malformation. Figure S2: schematic representation of our study. In our patient, the abnormal tissue within the PE deformity was found to harbor a somatic NF1 variant as a second hit. While NGS was performed in a more peripheral region, thus leading to the detection of the variant in a mosaic of ≈18% of reads, the WB targeted the core of the malformation, where the rate of the somatic mutation is expected to be much higher, and detected no evident wt NF1 protein. [file 3160653.f1.zip › Supplementary Material (1).docx]

**Supplementary Material**

**1. Supplementary Methods**

**2. Supplementary Figure**

**1. Supplementary Methods**

***Western Blot Analysis***

Cartilage fragments were finely pulverized in liquid nitrogen using mortar and pestle and then lysed in lysis buffer (50 mM Tris-Cl pH 7.4, 150 mM NaCl, 1 mM EDTA, 1% Triton X-100 and 5% β-mercaptoethanol) supplemented with a protease inhibitor cocktail (Roche). Protein concentration of each sample was determined using the Bradford method (Bio-Rad). 15 µg of total protein was denatured at 95°C and separated on 4–15% Mini-PROTEAN gel (Bio-rad). The gel was transferred on to a nitrocellulose membrane using Trans-Blot Turbo Transfer System and Trans-Blot Turbo Mini 0.2 µm Nitrocellulose Transfer Pack (Bio-Rad). NF1 protein was detected using NF1 Polyclonal Antibody (1:1000, A304-931A - Bethyl Laboratories, Inc) and rabbit secondary antibody (1:10000, Millipore). Clarity™ Western ECL Substrate (Bio-Rad) was used for the detection of the signals. Images were acquired by Uvitec Mini HD9 (Uvitec).

**2. Supplementary Figure**


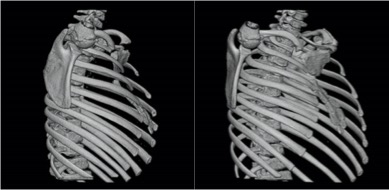


**Supplementary Figure 1.** 3D computed tomography reconstruction of the anterior chest wall showing the severe dorsal deviation of the manubrium and the sternal bone.
