# Supplementary material for: Somatic Double Inactivation of NF1 Associated with NF1-Related Pectus Excavatum Deformity
Source: Hum Mutat. 2023 Apr 28;2023:3160653. doi: 10.1155/2023/3160653 (PMC11918561; doi:10.1155/2023/3160653)
Supplement: Supplementary Materials — Supplementary Methods: description of western blot analysis 2. Figure S1: 3D reconstruction of the thoracic malformation. Figure S2: schematic representation of our study. In our patient, the abnormal tissue within the PE deformity was found to harbor a somatic NF1 variant as a second hit. While NGS was performed in a more peripheral region, thus leading to the detection of the variant in a mosaic of ≈18% of reads, the WB targeted the core of the malformation, where the rate of the somatic mutation is expected to be much higher, and detected no evident wt NF1 protein. [file 3160653.f1.zip › Table S1.pdf]

	CHROM	POS	ID	REF	ALT	QUAL	FILTER	SYMBOL
1390	chr12	6936733	rs7821992	AGC	A	31,04	PASS	ATN1
1391	chr12	6936736	rs7825555	AGCAGCA(	A	35,42	PASS	ATN1
2926	chr17	31258440	.	GC	G	26,71	PASS	NF1
3035	chr17	58216123	rs8632252	AG	A	47,44	PASS	MKS1
3340	chr19	2252388	rs7740586	CG	C	50,76	PASS	JSRP1
6496	chr6	1,57E+08	.	C	CG	3,64	PASS	ARID1B
6497	chr6	1,57E+08	rs1394628	G	GGC	6,33	PASS	ARID1B
7304	chr9	34723585	.	GA	G	42,16	PASS	FAM205A
7613	chrX	57910016	rs7530633	GCA	G	19,24	PASS	ZXDA
7614	chrX	57910019	rs7586684	G	GGC	25,67	PASS	ZXDA
112	chr1	30908539	rs1010528	GCCGGCC(	G	24,43	PASS	SDC3
1019	chr11	1630163	.	CCTGTGGC	C	12,62	PASS	KRTAP5-5
1101	chr11	18706105	rs7470480	CCTT	C	48,89	PASS	IGSF22
1222	chr11	71538456	rs5324381	GCCAGTCC	G	14,79	PASS	KRTAP5-8
1284	chr11	1,07E+08	rs3682791	CCAA	C	42,23	PASS	CWF19L2
1607	chr12	1,04E+08	rs5499150	GCAGAAG,	G	39,82	PASS	HSP90B1
1776	chr13	63832904	rs2013991	GGAGCCA	G	3,12	PASS	
2389	chr15	99712504	.	CCAGCAG(	C	50,3	PASS	MEF2A
2466	chr16	4895398	rs5679142	TCTC	T	49,26	PASS	PPL
2729	chr16	88436563	rs2818651	GCTTCCCG	G	41,27	PASS	ZNF469
4201	chr2	2,31E+08	.	TTCG	T	30,95	PASS	NCL
4873	chr22	20566526	.	ACAGCAG	A	35,3	PASS	MED15
6285	chr6	43012970	rs3682380	CCCT	C	41,12	PASS	MEA1
7247	chr9	2039776	.	ACAGCAG(	A	50,83	PASS	SMARCA2
348	chr1	1,46E+08	rs1476860	G	GGTCTTC	12,01	PASS	NBPF10
546	chr1	2,12E+08	.	T	TGTC	45,38	PASS	PPP2R5A
1783	chr13	71866566	.	G	GGCC	33,69	PASS	DACH1
2189	chr15	40036395	.	G	GTGCTGC	43,96	PASS	SRP14
4799	chr21	44592338	rs5876118	C	CGGGGCG	32,68	PASS	KRTAP10-6
4871	chr22	20427488	rs7682429	C	CCAG	42,21	PASS	SCARF2
5843	chr5	44809331	.	G	GGAGCCC	48,07	PASS	MRPS30
5975	chr5	1,27E+08	.	A	AGAG	46,07	PASS	LMNB1
6797	chr7	1,01E+08	rs1424367	T	TCAG	17,03	PASS	MUC3A
14	chr1	1968786	rs7263630	C	T	50,81	PASS	CFAP74
23	chr1	3833890	rs7725470	A	T	57,09	PASS	CEP104
43	chr1	12115595	rs7782033	C	T	54,62	PASS	TNFRSF8
48	chr1	13321864	rs1460806	T	C	12,06	PASS	PRAMEF15
66	chr1	17339721	rs7456766	C	T	51,55	PASS	PADI4
73	chr1	18908644	rs4127317	C	T	54,51	PASS	IFFO2
83	chr1	20323486	rs2013703	T	C	52,16	PASS	VWA5B1
86	chr1	20899718	rs1492005	C	A	47,68	PASS	EIF4G3
102	chr1	25321930	rs1500590	G	A	35,88	PASS	RHD
104	chr1	25811499	.	C	A	51,77	PASS	SELENON
137	chr1	37999018	rs1425281	G	A	50,25	PASS	FHL3
147	chr1	42410139	rs1012321	A	G	51,81	PASS	RIMKLA
153	chr1	43222212	rs7461819	G	A	51,58	PASS	CFAP57
166	chr1	46512214	rs1144863	G	C	50,42	PASS	DMBX1
189	chr1	56912476	rs1620075	G	A	40,01	PASS	C8A
205	chr1	64647869	rs1454328	G	A	42,45	PASS	CACHD1

**227**	chr1	78628971	rs1316013	G	A	4,3	PASS	IFI44L
**233**	chr1	84555012	rs3416052	A	G	42,11	PASS	CTBS
**237**	chr1	85071948	rs1487249	C	G	48,49	PASS	DNAI3
**260**	chr1	1E+08	rs1013226	C	T	49,67	PASS	SASS6
**299**	chr1	1,15E+08	rs7730274	G	A	50,55	PASS	DENND2C
**300**	chr1	1,15E+08	rs6148110	A	G	45,09	PASS	DENND2C
**312**	chr1	1,17E+08	rs7458048	A	G	49,53	PASS	TTF2
**319**	chr1	1,2E+08	rs7821690	G	A	50,63	PASS	NOTCH2
**331**	chr1	1,21E+08	rs2289361	T	C	19,06	PASS	SRGAP2C
**336**	chr1	1,44E+08	rs1375027	A	C	3,74	PASS	FAM72C
**375**	chr1	1,5E+08	rs1430053	C	T	52,1	PASS	CIART
**379**	chr1	1,51E+08	.	T	A	48,9	PASS	ADAMTSL4
**394**	chr1	1,52E+08	rs2004447	C	T	20,4	PASS	HRNR
**395**	chr1	1,52E+08	rs4126613	A	G	46,1	PASS	HRNR
**407**	chr1	1,54E+08	.	G	C	52,35	PASS	CRTC2
**413**	chr1	1,55E+08	rs1392488	A	G	43,62	PASS	PMVK
**421**	chr1	1,57E+08	rs1487272	A	G	53,12	PASS	ARHGEF11
**425**	chr1	1,59E+08	rs1785157	C	T	47,47	PASS	ACKR1
**447**	chr1	1,7E+08	rs1338927	T	G	3,69	PASS	F5
**454**	chr1	1,72E+08	rs1119280	C	G	48,06	PASS	PRRC2C
**466**	chr1	1,74E+08	rs7474129	T	C	43,95	PASS	SERPINC1
**499**	chr1	1,97E+08	rs1751428	A	G	39,75	PASS	F13B
**502**	chr1	1,97E+08	rs1393672	G	A	38,26	PASS	ASPM
**511**	chr1	2,01E+08	rs6181816	G	C	8,91	PASS	IGFN1
**519**	chr1	2,04E+08	rs1014119	C	A	48,25	PASS	ETNK2
**526**	chr1	2,06E+08	rs1785075	C	T	49,52	PASS	CDK18
**533**	chr1	2,07E+08	.	A	C	4,44	PASS	C4BPA
**559**	chr1	2,2E+08	rs1159903	T	G	49,22	PASS	EPRS1
**578**	chr1	2,28E+08	.	C	G	47,23	PASS	WNT9A
**580**	chr1	2,28E+08	rs1999335	C	T	48,99	PASS	OBSCN
**582**	chr1	2,28E+08	rs5602174	G	A	48,16	PASS	OBSCN
**603**	chr1	2,38E+08	rs3601713	T	C	24,28	PASS	ZP4
**613**	chr1	2,4E+08	rs7725114	A	C	54,06	PASS	CHRM3
**677**	chr10	14897071	rs7637843	A	G	44,14	PASS	SUV39H2
**702**	chr10	24585182	rs1420361	G	A	31,68	PASS	ARHGAP21
**708**	chr10	27120497	.	G	A	43,72	PASS	YME1L1
**720**	chr10	29554792	.	G	T	47,02	PASS	SVIL
**727**	chr10	32287249	rs7658456	G	A	48,41	PASS	EPC1
**732**	chr10	37158517	rs1693741	A	G	27,67	PASS	ANKRD30A
**757**	chr10	44977865	rs1228601	T	C	45,68	PASS	DEPP1
**765**	chr10	47368142	rs7502644	A	G	52,62	PASS	ZNF488
**774**	chr10	50092189	rs2004370	G	A	25,01	PASS	WASHC2A
**782**	chr10	58267530	rs1455192	T	C	44,12	PASS	IPMK
**836**	chr10	87152189	rs7639716	A	G	34,59	PASS	SHLD2
**849**	chr10	89417395	rs1433995	C	G	49,2	PASS	IFIT5
**858**	chr10	93068351	.	T	C	53,2	PASS	CYP26C1
**861**	chr10	94246253	rs6175149	G	A	32,17	PASS	PLCE1
**869**	chr10	94688163	rs1171111	C	T	43,97	PASS	CYP2C18
**873**	chr10	96158462	rs7148239	T	C	44,78	PASS	ZNF518A
**883**	chr10	1E+08	rs1178610	G	C	44,86	PASS	SEC31B

**892**	chr10	1,02E+08	rs7523467	C	T	50,49	PASS	CUEDC2
**899**	chr10	1,04E+08	.	T	C	51,22	PASS	CFAP43
**924**	chr10	1,2E+08	rs6175708	G	C	52,37	PASS	INPP5F
**929**	chr10	1,22E+08	rs2003062	G	A	48,68	PASS	BTBD16
**956**	chr10	1,3E+08	rs2308318	G	A	49,52	PASS	MGMT
**963**	chr10	1,31E+08	rs4128290	G	C	54,29	PASS	TCERG1L
**966**	chr10	1,32E+08	rs7500152	C	A	53,17	PASS	DPYSL4
**979**	chr10	1,33E+08	rs5497811	C	T	56,99	PASS	CFAP46
**980**	chr10	1,33E+08	rs1480867	A	G	53,45	PASS	VENTX
**998**	chr11	400655	.	C	A	48,91	PASS	PKP3
**1004**	chr11	556048	rs1997515	G	A	49,43	PASS	LMNTD2
**1005**	chr11	560710	rs2009224	A	G	51,79	PASS	LMNTD2
**1007**	chr11	598481	rs1821440	G	T	53,3	PASS	PHRF1
**1023**	chr11	1956322	rs12812	G	A	49,45	PASS	MRPL23
**1025**	chr11	1972745	rs190063	C	A	51,17	PASS	MRPL23
**1061**	chr11	4390240	rs2014752	C	T	36,68	PASS	TRIM21
**1062**	chr11	4682068	rs5455741	A	G	30,78	PASS	OR51E2
**1066**	chr11	6217526	.	T	C	52,2	PASS	FHIP1B
**1077**	chr11	7825582	rs1495009	G	A	53,83	PASS	OR5P3
**1078**	chr11	7928314	rs1480697	C	T	48,35	PASS	OR10A6
**1084**	chr11	11842208	.	A	G	43,86	PASS	USP47
**1096**	chr11	17387701	.	T	C	52,8	PASS	KCNJ11
**1103**	chr11	18707941	rs1463427	C	T	50,72	PASS	IGSF22
**1145**	chr11	50033724	rs1219681	T	C	42,58	PASS	OR4C45
**1147**	chr11	55343361	rs1397716	G	C	46,02	PASS	OR4A16
**1156**	chr11	58422520	rs1402382	C	T	12,1	PASS	OR5B2
**1182**	chr11	62726780	rs1404419	T	C	50,41	PASS	HNRNPUL2
**1200**	chr11	65641112	rs2008288	C	T	47,23	PASS	SIPA1
**1205**	chr11	66315572	rs7481890	C	T	52,41	PASS	CD248
**1206**	chr11	66367538	rs1998733	C	T	49,7	PASS	SLC29A2
**1207**	chr11	66526151	rs7581394	G	A	54,37	PASS	BBS1
**1215**	chr11	69699566	rs7654408	G	A	53	PASS	FGF19
**1232**	chr11	75172528	rs7649854	C	T	47,56	PASS	SLCO2B1
**1244**	chr11	78702097	rs5500196	C	T	42,64	PASS	TENM4
**1261**	chr11	93821076	rs1388351	C	G	52	PASS	VSTM5
**1296**	chr11	1,13E+08	rs3587732	G	A	39,12	PASS	ANKK1
**1312**	chr11	1,19E+08	rs1385443	A	C	51,27	PASS	DPAGT1
**1329**	chr11	1,24E+08	rs7490960	A	G	50,83	PASS	OR10D3
**1333**	chr11	1,25E+08	rs1926220	G	A	49,7	PASS	ROBO3
**1337**	chr11	1,26E+08	.	A	T	51,8	PASS	PATE4
**1341**	chr11	1,26E+08	rs1438780	A	G	55,92	PASS	ST3GAL4
**1374**	chr12	2888202	rs1478406	C	G	55,58	PASS	RHNO1
**1393**	chr12	7108268	rs7766391	C	G	58,86	PASS	C1RL
**1401**	chr12	8456134	rs1180840	A	C	41,38	PASS	CLEC6A
**1409**	chr12	11308615	rs1105424	G	C	13,53	PASS	PRB4
**1410**	chr12	11353354	rs1408252	T	C	7,62	PASS	PRB1
**1427**	chr12	18399681	rs2002639	T	C	40,42	PASS	PIK3C2G
**1466**	chr12	32640381	rs6175335	G	A	36,75	PASS	FGD4
**1469**	chr12	39586278	.	T	G	50,96	PASS	ABCD2
**1490**	chr12	49295694	rs7486422	T	C	48,27	PASS	PRPH

**1493**	chr12	49795062	rs7448420	C	T	40,65	PASS	NCKAP5L
**1502**	chr12	52290240	rs2018469	T	G	5,09	PASS	KRT81
**1503**	chr12	52448859	rs1389888	C	T	26,97	PASS	KRT6B
**1519**	chr12	56083757	rs5601715	C	T	49,03	PASS	ERBB3
**1530**	chr12	57102918	rs1454062	G	C	25,01	PASS	STAT6
**1570**	chr12	81268054	rs6175641	C	T	44,57	PASS	PPFIA2
**1575**	chr12	85154032	rs7593168	C	T	46,74	PASS	LRRIQ1
**1589**	chr12	95084627	rs1419383	C	T	52,04	PASS	FGD6
**1590**	chr12	95141450	rs1320983	C	G	48,69	PASS	FGD6
**1591**	chr12	95210752	rs1411867	C	T	38,24	PASS	FGD6
**1628**	chr12	1,13E+08	rs6173239	G	A	51,5	PASS	OAS3
**1641**	chr12	1,19E+08	rs2015598	G	A	33,15	PASS	SRRM4
**1658**	chr12	1,24E+08	rs6174578	C	A	44,42	PASS	DNAH10
**1661**	chr12	1,25E+08	rs7590974	C	G	58,15	PASS	DHX37
**1666**	chr12	1,29E+08	rs1914814	C	A	49,27	PASS	TMEM132
**1699**	chr13	19825016	rs2018120	A	G	51,49	PASS	ZMYM5
**1709**	chr13	24710469	rs1513274	C	A	50,53	PASS	ATP12A
**1720**	chr13	32267277	.	G	A	52,55	PASS	FRY
**1723**	chr13	32379413	rs1157176	G	A	46,66	PASS	BRCA2
**1739**	chr13	41336653	rs1414365	A	G	47,14	PASS	NAA16
**1798**	chr13	76992103	rs1469938	C	T	47,44	PASS	CLN5
**1809**	chr13	77168483	rs1417176	T	C	47,8	PASS	MYCBP2
**1827**	chr13	95790919	rs7563446	G	C	47,8	PASS	DNAJC3
**1892**	chr14	18601318	rs1411526	T	C	20,61	PASS	OR11H12
**1893**	chr14	18977471	rs1133806	T	C	34,13	PASS	POTEM
**1896**	chr14	19713015	rs1995188	T	C	15,38	PASS	OR11H2
**1898**	chr14	20699678	rs5954336	G	A	49,24	PASS	RNASE4
**1904**	chr14	20802007	rs1419233	C	T	46,99	PASS	RNASE1
**1907**	chr14	21022116	rs1403269	C	T	51,07	PASS	NDRG2
**1961**	chr14	41887171	rs7535169	G	A	46,2	PASS	LRFN5
**1988**	chr14	57233867	rs3506409	T	G	36,15	PASS	EXOC5
**2003**	chr14	64487864	rs7641385	C	T	50,62	PASS	ZBTB25
**2015**	chr14	69709037	rs1206268	A	T	52,33	PASS	SUSD6
**2017**	chr14	70738281	rs7455898	G	A	49,64	PASS	MAP3K9
**2054**	chr14	90290518	rs4546299	C	G	50,35	PASS	NRDE2
**2075**	chr14	96329581	rs2017275	G	A	24,77	PASS	ATG2B
**2081**	chr14	1,01E+08	rs1999967	C	T	47,06	PASS	BEGAIN
**2082**	chr14	1,01E+08	rs1434298	G	A	48,76	PASS	RTL1
**2091**	chr14	1,03E+08	rs1180549	G	A	44,12	PASS	LBHD2
**2101**	chr14	1,05E+08	rs2014348	C	T	56,56	PASS	AHNAK2
**2102**	chr14	1,05E+08	rs1995501	T	C	32,76	PASS	AHNAK2
**2104**	chr14	1,05E+08	rs2006015	T	A	31,24	PASS	AHNAK2
**2106**	chr14	1,05E+08	rs1504465	G	A	18,03	PASS	AHNAK2
**2107**	chr14	1,05E+08	rs2013953	C	G	9,34	PASS	AHNAK2
**2108**	chr14	1,05E+08	rs1998704	G	C	9,37	PASS	AHNAK2
**2112**	chr14	1,05E+08	rs3676004	G	A	7	PASS	AHNAK2
**2148**	chr15	28710495	.	G	A	3,25	PASS	GOLGA8M
**2167**	chr15	32392862	rs5278251	G	T	30,42	PASS	GOLGA8K
**2168**	chr15	32393182	rs1349996	G	A	6,17	PASS	GOLGA8K
**2169**	chr15	32393507	rs1196515	A	G	13,32	PASS	GOLGA8K

**2181**	chr15	33153798	rs1483317	G	C	51,77	PASS	FMN1
**2200**	chr15	41226494	rs7815732	C	T	48,23	PASS	EXD1
**2212**	chr15	43828142	rs3462323	G	A	44,61	PASS	WDR76
**2225**	chr15	50722170	rs1169393	A	T	53,33	PASS	SPPL2A
**2246**	chr15	57516796	rs7803590	C	T	53,65	PASS	CGNL1
**2254**	chr15	59679747	rs1385950	T	C	48,04	PASS	BNIP2
**2257**	chr15	61969413	rs1464605	T	C	38,04	PASS	VPS13C
**2296**	chr15	72163291	rs1413616	G	A	52,26	PASS	GRAMD2A
**2312**	chr15	75266309	rs7547383	C	T	5,27	PASS	GOLGA6C
**2319**	chr15	79458077	rs1175006	A	T	52,47	PASS	MINAR1
**2342**	chr15	84817531	.	G	C	45,65	PASS	ALPK3
**2358**	chr15	90201721	rs1998098	T	C	51,15	PASS	SEMA4B
**2415**	chr16	631414	.	G	C	52,99	PASS	WFIKKN1
**2416**	chr16	653395	rs7613897	G	A	50,98	PASS	WDR90
**2428**	chr16	1497044	rs1427531	G	T	51,69	PASS	TELO2
**2432**	chr16	1777413	rs7487249	C	T	56,4	PASS	SPSB3
**2434**	chr16	1945869	rs1479482	G	C	56,5	PASS	RPL3L
**2435**	chr16	1968876	rs1813990	T	C	49,04	PASS	RNF151
**2454**	chr16	3249675	rs1048951	G	A	54,82	PASS	MEFV
**2455**	chr16	3254268	rs1048950	G	A	47,02	PASS	MEFV
**2463**	chr16	4513004	rs7675416	G	A	49,67	PASS	CDIP1
**2482**	chr16	10694767	rs1459957	T	C	25,94	PASS	TEKT5
**2486**	chr16	11276207	rs7672822	C	T	44,24	PASS	PRM2
**2498**	chr16	14857532	rs1379630	G	A	23	PASS	NOMO1
**2503**	chr16	15750283	rs7711284	G	A	51,31	PASS	MYH11
**2531**	chr16	20362577	rs1397481	G	A	48,83	PASS	PDILT
**2532**	chr16	20384735	rs1396886	T	C	45,04	PASS	PDILT
**2534**	chr16	21087002	rs1174701	C	G	49,46	PASS	DNAH3
**2543**	chr16	24791323	rs3775719	G	A	34,93	PASS	TNRC6A
**2551**	chr16	28766167	rs1227499	G	A	11,37	PASS	NPIPB9
**2577**	chr16	31915039	rs1441188	G	A	52,16	PASS	ZNF267
**2595**	chr16	48561471	rs7771784	A	C	52,46	PASS	N4BP1
**2600**	chr16	51140315	rs7571694	G	A	41,02	PASS	SALL1
**2628**	chr16	67132828	rs3401745	G	A	48,08	PASS	PHAF1
**2650**	chr16	70827274	rs1423470	G	A	10,17	PASS	HYDIN
**2655**	chr16	70921102	rs3694903	C	T	18,42	PASS	HYDIN
**2672**	chr16	71999708	rs7574447	T	G	42,78	PASS	PKD1L3
**2674**	chr16	72096839	rs3751815	G	A	53,11	PASS	DHX38
**2696**	chr16	80608133	.	C	T	48,94	PASS	CDYL2
**2707**	chr16	84130150	rs7761862	C	T	50,25	PASS	HSDL1
**2737**	chr16	88714634	rs1286773	A	G	45,61	PASS	CTU2
**2738**	chr16	88714718	rs7537817	G	C	46,97	PASS	CTU2
**2740**	chr16	88738609	rs1056843	G	A	47,59	PASS	PIEZO1
**2772**	chr16	89697603	rs7577741	T	C	36,8	PASS	SPATA2L
**2813**	chr17	2698361	rs7651502	G	A	51,83	PASS	CLUH
**2820**	chr17	3751832	rs1495401	C	T	53,48	PASS	ITGAE
**2822**	chr17	3905234	rs7622277	C	T	54,17	PASS	P2RX1
**2827**	chr17	4956862	rs5614740	G	A	57,47	PASS	ENO3
**2838**	chr17	6813007	rs9433717	C	T	29,54	PASS	TEKT1
**2842**	chr17	7283266	rs1390110	C	G	58,65	PASS	SLC2A4

**2852**	chr17	8230464	rs3708366	G	C	53,22	PASS	CTC1
**2872**	chr17	13600978	rs2006143	C	T	44,12	PASS	HS3ST3A1
**2915**	chr17	28591738	rs3708231	G	A	47,1	PASS	SPAG5
**2951**	chr17	40408118	rs7467651	C	T	41,86	PASS	TOP2A
**2953**	chr17	41097858	rs1407887	T	C	3,71	PASS	KRTAP4-8
**2955**	chr17	41275765	rs4890107	C	T	36,88	PASS	KRTAP9-7
**2962**	chr17	42672321	rs2002100	G	A	52,84	PASS	PLEKHH3
**2991**	chr17	45821396	rs3721813	G	A	53,24	PASS	CRHR1
**2998**	chr17	47156956	.	T	A	49,1	PASS	CDC27
**3011**	chr17	49298555	rs7799045	T	C	39,08	PASS	ZNF652
**3034**	chr17	58207104	rs2016195	C	T	44,27	PASS	MKS1
**3064**	chr17	65193569	rs1245228	C	T	51,49	PASS	RGS9
**3088**	chr17	74854900	rs7834982	G	A	52,23	PASS	GRIN2C
**3094**	chr17	75620337	rs1390452	C	T	51,71	PASS	MYO15B
**3099**	chr17	76140074	rs1378722	G	A	47,01	PASS	FOXJ1
**3139**	chr17	81537064	rs7520070	C	T	41,02	PASS	FSCN2
**3190**	chr18	21573874	rs1485369	C	T	38,66	PASS	ESCO1
**3194**	chr18	24170334	rs1451640	C	T	52,18	PASS	OSBPL1A
**3219**	chr18	33742937	.	T	C	53,79	PASS	ASXL3
**3280**	chr18	62972609	rs7547265	A	G	48,2	PASS	PHLPP1
**3311**	chr18	76880165	rs1394519	C	T	50,98	PASS	ZNF236
**3334**	chr19	1456384	rs1465794	C	A	55,13	PASS	APC2
**3341**	chr19	2353076	rs1130090	A	G	48,54	PASS	SPPL2B
**3350**	chr19	3820250	rs9915717	C	T	38,38	PASS	ZFR2
**3355**	chr19	4433399	rs1184924	G	T	51,74	PASS	CHAF1A
**3360**	chr19	4512878	rs1999441	G	A	38,89	PASS	PLIN4
**3361**	chr19	4512933	rs7503143	C	T	17,23	PASS	PLIN4
**3367**	chr19	4513076	rs5423841	G	A	35,97	PASS	PLIN4
**3369**	chr19	4513082	rs1232780	C	G	37,26	PASS	PLIN4
**3374**	chr19	5694892	rs1510250	C	G	45,82	PASS	LONP1
**3378**	chr19	6040035	rs1438382	C	A	25,89	PASS	RFX2
**3389**	chr19	7766051	rs5900245	G	C	14,28	PASS	CLEC4M
**3403**	chr19	10110993	rs7440688	G	A	48,08	PASS	PPAN
**3407**	chr19	10923605	rs3726869	C	T	48,23	PASS	YIPF2
**3412**	chr19	11575174	rs1470255	G	A	51,52	PASS	ACP5
**3451**	chr19	16471938	rs1437691	G	A	44,46	PASS	EPS15L1
**3452**	chr19	16993429	rs1513350	T	C	52,79	PASS	CPAMD8
**3463**	chr19	18617040	rs6173160	G	A	49,55	PASS	TMEM59L
**3466**	chr19	18784263	rs7453998	C	T	53,86	PASS	COMP
**3467**	chr19	18907090	rs1456392	C	G	68,52	PASS	COPE
**3468**	chr19	19305923	rs2007408	G	A	49,9	PASS	SUGP1
**3469**	chr19	19679881	rs3535894	C	T	17,97	PASS	ZNF101
**3481**	chr19	22181059	rs7253392	T	C	5,61	PASS	ZNF676
**3484**	chr19	22858297	rs1420171	A	C	43,38	PASS	ZNF723
**3503**	chr19	35841784	rs1381731	C	A	53,33	PASS	NPHS1
**3530**	chr19	40223137	rs7568597	C	T	47,48	PASS	CCNP
**3549**	chr19	45064410	rs7135225	C	A	49,59	PASS	CLASRP
**3557**	chr19	46649639	rs5506540	C	T	48,87	PASS	DACT3
**3559**	chr19	46782545	rs1409083	G	C	55,49	PASS	SLC1A5
**3579**	chr19	49022947	rs1120031	G	C	22,95	PASS	CGB3

**3581**	chr19	49032576	rs3693119	C	G	19,69	PASS	CGB2
**3582**	chr19	49035674	rs2012314	T	G	31,53	PASS	CGB1
**3585**	chr19	49294407	rs1382532	C	T	12,61	PASS	SLC6A16
**3629**	chr19	53352398	rs1822520	A	G	52,42	PASS	ZNF845
**3659**	chr19	55500750	rs1413073	G	A	38,55	PASS	SSC5D
**3701**	chr2	1643470	rs1470669	C	A	54,29	PASS	PXDN
**3739**	chr2	17510922	rs1838824	C	T	41,29	PASS	RAD51AP2
**3769**	chr2	27224375	rs1462827	C	A	51,14	PASS	CAD
**3777**	chr2	32617828	rs1510180	G	A	50,56	PASS	BIRC6
**3781**	chr2	36808574	rs1416659	C	T	55	PASS	VIT
**3802**	chr2	43675895	rs8026301	G	A	51,07	PASS	C1GALT1C
**3806**	chr2	43943840	rs1413844	G	T	47,52	PASS	LRPPRC
**3826**	chr2	54334933	rs7744675	G	A	54,35	PASS	C2orf73
**3840**	chr2	61188133	rs7792439	A	G	51,13	PASS	USP34
**3870**	chr2	70277247	rs1997231	A	G	49,1	PASS	PCYOX1
**3887**	chr2	74527043	rs2016486	G	A	56,56	PASS	AUP1
**3893**	chr2	75687859	rs4128600	A	T	37,48	PASS	GCFC2
**3901**	chr2	84517989	rs1121661	C	T	22,68	PASS	DNAH6
**3919**	chr2	88182960	.	A	G	50,69	PASS	THNSL2
**3931**	chr2	95596417	rs2022397	T	G	9,68	PASS	TRIM43
**3936**	chr2	95889820	rs7747625	C	T	34,24	PASS	ANKRD36C
**3944**	chr2	98391955	rs7729823	G	A	53,69	PASS	CNGA3
**4009**	chr2	1,3E+08	rs5332362	G	A	17,53	PASS	POTEI
**4010**	chr2	1,3E+08	rs4588203	G	A	18,36	PASS	POTEI
**4011**	chr2	1,3E+08	rs4380247	T	C	4,33	PASS	POTEI
**4020**	chr2	1,33E+08	rs6173571	G	A	46,92	PASS	GPR39
**4022**	chr2	1,35E+08	rs1167759	A	G	47,81	PASS	RAB3GAP1
**4039**	chr2	1,52E+08	rs3570776	G	A	12,39	PASS	NEB
**4049**	chr2	1,57E+08	rs7976650	T	C	44,48	PASS	GALNT5
**4067**	chr2	1,66E+08	rs7767215	A	G	38,17	PASS	TTC21B
**4070**	chr2	1,69E+08	rs3411012	C	T	49,45	PASS	CERS6
**4089**	chr2	1,78E+08	rs1484959	C	T	43,81	PASS	TTC30A
**4097**	chr2	1,79E+08	rs7559351	T	C	47,89	PASS	TTN
**4107**	chr2	1,86E+08	rs7500048	C	T	50,03	PASS	FSIP2
**4142**	chr2	2,06E+08	rs1154638	G	A	50,17	PASS	EEF1B2
**4144**	chr2	2,07E+08	rs1147097	G	A	49,26	PASS	DYTN
**4147**	chr2	2,08E+08	rs7813497	C	T	52,06	PASS	CRYGD
**4175**	chr2	2,19E+08	.	G	T	52,17	PASS	BCS1L
**4182**	chr2	2,2E+08	rs7706635	G	T	47,76	PASS	STK11IP
**4240**	chr2	2,41E+08	rs1159271	G	A	54,46	PASS	CROCC2
**4256**	chr20	1305447	rs1443387	A	G	53,47	PASS	SNPH
**4275**	chr20	4221530	.	T	C	53,28	PASS	ADRA1D
**4296**	chr20	16404849	rs1448841	C	T	36,84	PASS	KIF16B
**4315**	chr20	23749122	rs1122328	C	G	42,88	PASS	CST1
**4414**	chr20	35274296	rs2001022	G	A	44,41	PASS	MMP24
**4424**	chr20	38765470	rs1996049	G	C	51,78	PASS	ACTR5
**4439**	chr20	44930722	rs2005292	C	T	42,77	PASS	PABPC1L
**4445**	chr20	45222882	rs1447083	G	A	45,83	PASS	SEMG2
**4464**	chr20	45883374	rs1159260	C	G	52,69	PASS	ZSWIM1
**4487**	chr20	49249611	rs1396441	A	C	43,21	PASS	ZNFX1

**4524**	chr20	62193496	.	T	A	48,36	PASS	MTG2
**4531**	chr20	62414108	rs4556583	G	A	52,51	PASS	RBBP8NL
**4558**	chr21	14221164	rs1419538	C	A	48,98	PASS	RBM11
**4559**	chr21	14966964	rs3692614	G	A	46,28	PASS	NRIP1
**4572**	chr21	29034891	rs1174849	A	G	45,61	PASS	USP16
**4577**	chr21	30215433	.	G	C	40,97	PASS	CLDN8
**4581**	chr21	32352817	rs7706458	C	T	50,49	PASS	URB1
**4584**	chr21	33414915	.	A	G	32,54	PASS	IFNGR2
**4608**	chr21	34449523	rs1805127	T	C	52,24	PASS	KCNE1
**4617**	chr21	36278075	rs1437149	G	A	49,39	PASS	DOP1B
**4620**	chr21	36397429	rs1406307	A	C	46,08	PASS	CHAF1B
**4702**	chr21	43426136	rs3746951	C	T	63,79	PASS	SIK1
**4731**	chr21	44109038	rs2020945	G	A	65,19	PASS	PWP2
**4746**	chr21	44133714	rs968714	T	C	69,72	PASS	GATD3
**4793**	chr21	44236891	rs1155881	C	T	43,92	PASS	ICOSLG
**4796**	chr21	44250887	rs7354779	T	C	51,02	PASS	DNMT3L
**4840**	chr22	18529038	rs2930761	C	T	20,36	PASS	TMEM191
**4843**	chr22	18608593	rs1997688	C	G	18,31	PASS	RIMBP3
**4851**	chr22	18913491	rs450046	C	T	48,54	PASS	PRODH
**4859**	chr22	19766661	rs1458962	G	C	44,64	PASS	TBX1
**4860**	chr22	20055981	rs1424422	G	A	47,7	PASS	TANGO2
**4870**	chr22	20425389	rs1205842	C	T	52,2	PASS	SCARF2
**4906**	chr22	23617618	rs3741685	C	T	41,51	PASS	DRICH1
**4910**	chr22	24313452	rs3578391	C	T	48,91	PASS	SPECC1L
**4916**	chr22	24905175	rs6174227	A	G	55,86	PASS	SGSM1
**4918**	chr22	25039919	rs1125873	C	T	42,01	PASS	KIAA1671
**4928**	chr22	29060683	rs3596836	G	A	45,6	PASS	C22orf31
**4943**	chr22	32221015	rs7453094	C	T	46,18	PASS	SLC5A4
**4946**	chr22	35323508	rs9269162	G	A	30,74	PASS	TOM1
**4972**	chr22	40418883	.	C	G	47,41	PASS	MRTFA
**4981**	chr22	41926277	rs5473523	C	A	36,78	PASS	TNFRSF13(
**5001**	chr22	44859802	rs1496967	G	A	45,59	PASS	ARHGAP8
**5002**	chr22	45328017	rs6200186	G	A	32,44	PASS	FAM118A
**5010**	chr22	46534872	rs7690300	C	T	56,03	PASS	CELSR1
**5057**	chr3	9704754	rs2015179	G	C	47,68	PASS	CPNE9
**5109**	chr3	37994336	rs7636412	G	A	50,92	PASS	VILL
**5113**	chr3	38400906	rs1469982	C	T	49,63	PASS	XYLB
**5115**	chr3	38550704	rs7668755	C	T	51,79	PASS	SCN5A
**5126**	chr3	42223223	rs1435910	C	G	56,51	PASS	TRAK1
**5132**	chr3	44721907	.	C	G	45,86	PASS	ZNF502
**5138**	chr3	46671021	rs1441817	C	T	53,43	PASS	ALS2CL
**5145**	chr3	48631930	rs1235575	C	T	38,17	PASS	SLC26A6
**5150**	chr3	49132631	rs1447657	G	C	51,29	PASS	LAMB2
**5157**	chr3	49897376	rs1486945	C	T	49,69	PASS	MST1R
**5159**	chr3	50331440	rs1509992	C	T	53,25	PASS	RASSF1
**5162**	chr3	52513174	rs3775412	G	A	45,28	PASS	STAB1
**5163**	chr3	52513236	rs4129285	A	G	47,15	PASS	STAB1
**5164**	chr3	52693659	rs3421613	G	C	47,98	PASS	GNL3
**5169**	chr3	57363715	rs1008564	A	G	38,22	PASS	DNAH12
**5186**	chr3	66383135	rs5293412	C	T	48,38	PASS	LRIG1

**5207**	chr3	88466939	rs1047629	A	T	42,33	PASS	CSNKA2IP
**5243**	chr3	1,08E+08	rs1442211	G	C	46,84	PASS	MYH15
**5249**	chr3	1,13E+08	rs1477621	C	T	45,46	PASS	NEPRO
**5262**	chr3	1,22E+08	rs1443852	C	G	48,25	PASS	GOLGB1
**5263**	chr3	1,22E+08	rs1144200	G	A	51,71	PASS	GOLGB1
**5275**	chr3	1,29E+08	rs1161873	C	A	49,3	PASS	CFAP92
**5308**	chr3	1,41E+08	rs7640677	A	G	53,55	PASS	TRIM42
**5314**	chr3	1,44E+08	rs1996687	C	T	51,8	PASS	DIPK2A
**5317**	chr3	1,49E+08	rs1453469	G	A	49,17	PASS	CPB1
**5335**	chr3	1,59E+08	rs7545390	A	T	44,67	PASS	RARRES1
**5383**	chr3	1,86E+08	rs1389003	G	C	52,36	PASS	LIPH
**5409**	chr3	1,96E+08	rs1146083	G	A	56,05	PASS	MUC4
**5410**	chr3	1,96E+08	rs1191743	A	G	14,02	PASS	MUC4
**5411**	chr3	1,96E+08	rs1465257	G	A	28,73	PASS	MUC4
**5413**	chr3	1,96E+08	rs7792461	C	T	4,49	PASS	MUC4
**5414**	chr3	1,96E+08	rs7688945	T	C	13,87	PASS	MUC4
**5415**	chr3	1,96E+08	rs7442094	C	G	14,1	PASS	MUC4
**5416**	chr3	1,96E+08	rs7683914	A	C	9,58	PASS	MUC4
**5417**	chr3	1,96E+08	rs7129186	C	T	22,56	PASS	MUC4
**5418**	chr3	1,96E+08	rs7507550	C	G	13,37	PASS	MUC4
**5441**	chr4	859671	rs1471759	C	G	54,27	PASS	GAK
**5454**	chr4	3317528	rs1511941	G	T	35,36	PASS	RGS12
**5466**	chr4	6609222	rs1384186	G	A	54	PASS	MAN2B2
**5468**	chr4	8291466	rs1116208	G	A	57,04	PASS	HTRA3
**5471**	chr4	8468049	rs1498948	C	T	57,87	PASS	TRMT44
**5502**	chr4	36121190	rs7533471	C	T	40,52	PASS	ARAP2
**5521**	chr4	47643167	rs1447221	T	A	47,36	PASS	CORIN
**5541**	chr4	61934906	rs6174765	A	G	40,23	PASS	ADGRL3
**5551**	chr4	70656333	.	T	G	45,6	PASS	JCHAIN
**5559**	chr4	75793806	rs7633139	A	G	46,38	PASS	USO1
**5568**	chr4	78511299	rs6172936	G	A	29,12	PASS	FRAS1
**5580**	chr4	84724570	rs2014783	C	T	48,33	PASS	WDFY3
**5606**	chr4	1,1E+08	rs1148163	C	T	48,1	PASS	PLA2G12A
**5623**	chr4	1,19E+08	.	A	G	22,06	PASS	SYNPO2
**5632**	chr4	1,22E+08	rs1455137	C	T	51,41	PASS	ANXA5
**5656**	chr4	1,41E+08	rs1382467	A	G	47,86	PASS	ZNF330
**5672**	chr4	1,53E+08	rs7570170	C	A	48,88	PASS	ARFIP1
**5683**	chr4	1,57E+08	rs7785682	C	T	4,73	PASS	GLRB
**5713**	chr4	1,77E+08	rs2001825	C	T	46,64	PASS	VEGFC
**5726**	chr4	1,84E+08	rs2005969	T	G	51,41	PASS	IRF2
**5803**	chr5	23527528	rs6105179	A	C	11,71	PASS	PRDM9
**5809**	chr5	32074423	rs3474821	C	T	36,88	PASS	PDZD2
**5820**	chr5	35033474	rs7479425	T	C	33,79	PASS	AGXT2
**5821**	chr5	36985779	rs1031384	G	A	41,78	PASS	NIPBL
**5824**	chr5	37183056	.	G	A	47,18	PASS	CPLANE1
**5825**	chr5	37291910	rs2021941	C	T	43,99	PASS	NUP155
**5892**	chr5	72358383	rs7490963	C	T	55,27	PASS	PTCD2
**5910**	chr5	80559007	rs1485644	A	G	29,91	PASS	ANKRD34E
**5929**	chr5	95620472	rs1009249	G	T	40,26	PASS	GPR150
**5934**	chr5	98879628	rs7682564	C	T	44,17	PASS	CHD1

**5949**	chr5	1,12E+08	rs2002043	A	C	50,24	PASS	EPB41L4A
**5950**	chr5	1,12E+08	rs1997991	T	C	49,75	PASS	EPB41L4A
**5968**	chr5	1,23E+08	rs1142804	G	A	40,84	PASS	CEP120
**5978**	chr5	1,28E+08	rs5616807	C	T	48,69	PASS	FBN2
**5989**	chr5	1,33E+08	rs2890308	G	A	39,49	PASS	RAD50
**5991**	chr5	1,33E+08	rs1410688	C	G	49,61	PASS	AFF4
**5995**	chr5	1,35E+08	rs3683239	G	T	18,28	PASS	CAMLG
**6000**	chr5	1,38E+08	rs1996063	G	A	54,16	PASS	PKD2L2
**6013**	chr5	1,41E+08	rs7158363	G	C	51,55	PASS	PCDHB7
**6014**	chr5	1,41E+08	rs7819505	T	A	35,74	PASS	PCDHB11
**6016**	chr5	1,41E+08	rs7642410	G	A	29,07	PASS	PCDHGA3
**6017**	chr5	1,41E+08	rs2019018	T	G	52,59	PASS	PCDHGB4
**6018**	chr5	1,41E+08	rs1411340	G	C	51,77	PASS	PCDHGB4
**6031**	chr5	1,48E+08	rs9404431	A	G	47,08	PASS	SPINK5
**6033**	chr5	1,49E+08	rs1800888	C	T	51,12	PASS	ADRB2
**6060**	chr5	1,62E+08	rs1150696	G	C	50,9	PASS	GABRA6
**6075**	chr5	1,73E+08	rs2010267	C	G	53,58	PASS	DUSP1
**6076**	chr5	1,74E+08	.	G	A	49,14	PASS	CPEB4
**6088**	chr5	1,78E+08	rs1928989	T	C	58,6	PASS	FAM193B
**6094**	chr5	1,78E+08	.	T	C	54,32	PASS	PHYKPL
**6127**	chr6	7373201	rs3474489	C	G	54,4	PASS	CAGE1
**6130**	chr6	10874672	rs6173427	A	C	51,42	PASS	GCM2
**6145**	chr6	17624782	rs1561852	G	C	48,67	PASS	NUP153
**6148**	chr6	20546466	rs1117390	G	A	52,01	PASS	CDKAL1
**6162**	chr6	30704981	rs7486015	C	A	16,82	PASS	MDC1
**6163**	chr6	31029302	rs1407780	G	A	40,99	PASS	MUC22
**6173**	chr6	31269990	rs1130935	T	C	34,45	PASS	HLA-C
**6174**	chr6	31269992	rs1050105	G	A	32,21	PASS	HLA-C
**6176**	chr6	31269996	rs1130947	T	C	34,54	PASS	HLA-C
**6178**	chr6	31270002	rs1469113	C	T	33,15	PASS	HLA-C
**6180**	chr6	31270025	rs1050147	A	G	32,59	PASS	HLA-C
**6181**	chr6	31270056	rs1050180	T	C	29,99	PASS	HLA-C
**6195**	chr6	31270214	rs2308628	G	T	15,08	PASS	HLA-C
**6197**	chr6	31270233	rs1131015	T	G	18,02	PASS	HLA-C
**6198**	chr6	31270250	rs4154011	C	A	17,77	PASS	HLA-C
**6199**	chr6	31270252	rs2308622	T	C	21,29	PASS	HLA-C
**6200**	chr6	31270276	rs707908	G	C	25,84	PASS	HLA-C
**6201**	chr6	31270378	rs1050328	G	A	30,84	PASS	HLA-C
**6202**	chr6	31270482	rs1131096	G	T	34,77	PASS	HLA-C
**6206**	chr6	31271153	rs2308592	A	C	24,64	PASS	HLA-C
**6207**	chr6	31271165	rs2308590	G	T	17,22	PASS	HLA-C
**6209**	chr6	31271280	rs2308575	C	T	18,92	PASS	HLA-C
**6213**	chr6	31271830	rs4154242	G	A	21,45	PASS	HLA-C
**6214**	chr6	31271844	rs1071650	T	G	25,07	PASS	HLA-C
**6229**	chr6	32589702	rs9270302	G	A	3,49	PASS	HLA-DRB1
**6237**	chr6	33670361	rs2229633	C	A	40,3	PASS	ITPR3
**6241**	chr6	34036228	.	C	G	52,56	PASS	GRM4
**6246**	chr6	36225308	rs1421710	G	A	48,41	PASS	BRPF3
**6297**	chr6	43551437	rs6173988	T	C	51,61	PASS	XPO5
**6301**	chr6	43604716	rs3567557	C	T	40,12	PASS	POLH

**6343**	chr6	55874504	rs3512464	T	C	27,4	PASS	BMP5
**6345**	chr6	56604013	rs1873151	A	G	46,58	PASS	DST
**6386**	chr6	88145084	rs7678107	G	A	46,85	PASS	CNR1
**6411**	chr6	1,09E+08	rs1383602	A	G	51,95	PASS	AFG1L
**6426**	chr6	1,14E+08	.	C	A	48,66	PASS	MARCKS
**6443**	chr6	1,29E+08	rs1464625	G	A	46,7	PASS	LAMA2
**6447**	chr6	1,31E+08	rs1422963	T	C	31,15	PASS	EPB41L2
**6483**	chr6	1,5E+08	rs7476653	C	T	51,56	PASS	LRP11
**6509**	chr6	1,59E+08	rs1460145	G	C	54,06	PASS	FNDC1
**6534**	chr6	1,68E+08	rs2001863	C	T	50,6	PASS	DACT2
**6559**	chr7	195594	rs6173497	G	A	45,34	PASS	FAM20C
**6572**	chr7	1555313	rs1389177	C	T	52,5	PASS	TMEM184
**6584**	chr7	4788844	rs3725172	G	T	29,49	PASS	AP5Z1
**6618**	chr7	17339293	rs6175596	A	G	53,97	PASS	AHR
**6630**	chr7	21842591	rs3487920	G	A	45,68	PASS	DNAH11
**6650**	chr7	30755840	rs7629788	C	T	40,04	PASS	INMT
**6651**	chr7	32558973	rs7797206	C	T	49,96	PASS	AVL9
**6676**	chr7	44540147	rs3708110	C	T	54,17	PASS	NPC1L1
**6678**	chr7	44624507	rs2230445	C	T	49,74	PASS	OGDH
**6680**	chr7	45084307	rs6174088	T	G	3,26	PASS	NACAD
**6686**	chr7	47396863	.	A	G	50,1	PASS	TNS3
**6688**	chr7	48046554	rs1114808	G	A	46,1	PASS	C7orf57
**6696**	chr7	51029558	rs6173917	G	A	39,01	PASS	COBL
**6697**	chr7	51219840	rs2013257	T	C	47,33	PASS	COBL
**6704**	chr7	57126056	rs1999329	T	C	22,57	PASS	ZNF479
**6724**	chr7	73341348	rs2018628	C	A	52,65	PASS	FKBP6
**6725**	chr7	73459571	.	C	G	43,76	PASS	BAZ1B
**6745**	chr7	77274590	rs1481642	C	T	49,73	PASS	CCDC146
**6787**	chr7	99425250	rs1470452	C	T	49,7	PASS	PTCD1
**6790**	chr7	99863602	rs1421554	G	A	48,77	PASS	CYP3A43
**6795**	chr7	1E+08	rs2012409	G	A	48,78	PASS	NYAP1
**6796**	chr7	1,01E+08	rs3751885	A	T	47,2	PASS	MUC3A
**6800**	chr7	1,01E+08	rs2016667	G	A	48,79	PASS	PLOD3
**6816**	chr7	1,08E+08	rs5563845	T	C	51,02	PASS	SLC26A4
**6829**	chr7	1,12E+08	rs1390291	G	A	48,2	PASS	IFRD1
**6842**	chr7	1,23E+08	rs1473882	C	T	48,95	PASS	CADPS2
**6865**	chr7	1,38E+08	rs1469359	G	A	54,96	PASS	AKR1D1
**6881**	chr7	1,44E+08	rs1121901	C	T	31,34	PASS	NOBOX
**6885**	chr7	1,49E+08	rs3718735	G	A	50,13	PASS	ZNF786
**7013**	chr8	20254844	rs1491406	A	G	50,1	PASS	LZTS1
**7016**	chr8	22249274	rs7664760	G	A	52,29	PASS	POLR3D
**7035**	chr8	29116847	rs3606894	T	C	47,02	PASS	KIF13B
**7040**	chr8	30727777	rs5749807	G	A	47,03	PASS	GSR
**7045**	chr8	37877237	rs6174662	G	A	51,02	PASS	RAB11FIP1
**7046**	chr8	38105563	rs1930805	G	C	52,32	PASS	ASH2L
**7054**	chr8	42865438	rs1444351	C	T	49,65	PASS	RNF170
**7067**	chr8	53841605	rs3743328	T	C	48,97	PASS	ATP6V1H
**7068**	chr8	54101787	rs1479940	T	C	52,04	PASS	LYPLA1
**7091**	chr8	66460424	rs3735751	A	G	49,46	PASS	ADHFE1
**7157**	chr8	1,09E+08	.	G	T	45,06	PASS	NUDCD1

**7189**	chr8	1,33E+08	rs1388398	C	T		54,65	PASS	NDRG1
**7208**	chr8	1,43E+08	rs7500418	T	C		52,69	PASS	ZNF696
**7209**	chr8	1,44E+08	rs1154825	G	T		45,66	PASS	ZC3H3
**7221**	chr8	1,44E+08	rs1414217	C	G		50,72	PASS	WDR97
**7222**	chr8	1,44E+08	rs9937845	G	C		56,62	PASS	BOP1
**7226**	chr8	1,44E+08	rs7819589	C	T		43,59	PASS	SLC39A4
**7227**	chr8	1,44E+08	rs7818972	C	T		47,86	PASS	SLC39A4
**7243**	chr9	14889	rs7150992	A	G		7,81	PASS	WASHC1
**7302**	chr9	34256377	rs3410167	G	T		38,12	PASS	KIF24
**7307**	chr9	35663009	rs7551362	C	T		46,62	PASS	ARHGEF39
**7369**	chr9	74739854	rs1429466	C	T		52,69	PASS	TRPM6
**7373**	chr9	76708238	rs2012999	C	T		51,26	PASS	PRUNE2
**7395**	chr9	93259093	rs1464111	C	A		49,67	PASS	WNK2
**7417**	chr9	98807888	rs9170329	G	A		48,31	PASS	GALNT12
**7464**	chr9	1,23E+08	.	T	A		29,72	PASS	OR1L1
**7487**	chr9	1,31E+08	rs1482036	C	G		51,28	PASS	EXOSC2
**7538**	chrM	8860	rs2001031	A	G	.		PASS	MT-ATP6
**7545**	chrM	15326	rs2853508	A	G	.		PASS	MT-CYB
**7586**	chrX	26195008	rs1472282	C	T		44,73	PASS	MAGEB6
**7589**	chrX	44844601	rs7666275	G	A		54,99	PASS	DUSP21
**7598**	chrX	49571621	rs1215241	A	G		7,3	PASS	GAGE12G
**7600**	chrX	50916015	rs7822271	A	G		38,21	PASS	BMP15
**7608**	chrX	57594011	rs2007270	T	A		25,31	PASS	ZXDB
**7609**	chrX	57909999	rs5686334	T	C		42,39	PASS	ZXDA
**7611**	chrX	57910008	rs2235730	T	A		18,57	PASS	ZXDA
**7612**	chrX	57910015	rs7659845	A	T		19,79	PASS	ZXDA
**7624**	chrX	71604600	rs1446028	G	T		45,13	PASS	GCNA
**7640**	chrX	1,03E+08	rs7808176	G	T		46,82	PASS	TCEAL8
**7666**	chrX	1,36E+08	rs1434412	A	T		38,85	PASS	ADGRG4
**7676**	chrX	1,51E+08	rs7826446	C	T		53,57	PASS	MTMR1
**423**	chr1	1,58E+08	rs1999144	C	G		48,52	PASS	FCRL4
**574**	chr1	2,26E+08	rs7696898	C	T		50,34	PASS	PARP1
**589**	chr1	2,29E+08	rs1486283	G	A		23,44	PASS	NUP133
**773**	chr10	49472376	rs1457201	C	T		52,41	PASS	ERCC6
**935**	chr10	1,24E+08	rs1424838	C	G		50,75	PASS	CPXM2
**1119**	chr11	34136978	rs7592594	G	T		49,39	PASS	NAT10
**1166**	chr11	60869537	rs1457073	G	A		52,37	PASS	ZP1
**2541**	chr16	23695894	rs5617696	C	T		53,07	PASS	ERN2
**2891**	chr17	18282699	rs3677176	C	T		50,82	PASS	TOP3A
**3580**	chr19	49032504	rs1449295	G	A		29,53	PASS	CGB2
**3689**	chr19	58499219	.	A	G		49,35	PASS	SLC27A5
**3942**	chr2	97164285	.	T	C		20,27	PASS	ANKRD36
**4153**	chr2	2,12E+08	rs7595517	G	A		49,42	PASS	ERBB4
**4988**	chr22	42573937	rs1382654	A	G		26,37	PASS	SERHL2
**5534**	chr4	53476753	rs1435738	C	T		48,99	PASS	LNX1
**6194**	chr6	31270210	rs4155632	C	T		21,16	PASS	HLA-C
**6673**	chr7	44058434	rs1472339	C	T		50,9	PASS	DBNL
**7520**	chr9	1,37E+08	rs1450779	C	T		52,19	PASS	MAMDC4
**2911**	chr17	21702730	rs1365858	G	A		32,3	PASS	KCNJ18
**4019**	chr2	1,31E+08	rs1368717	A	G		7,64	PASS	POTEE

**6541**	chr6	1,7E+08	rs1380653	T	C	42,49	PASS	WDR27
**7679**	chrX	1,53E+08	rs7825775	CA	C	51,04	PASS	MAGEA1
**334**	chr1	1,43E+08	rs9729175	C	T	22,57	PASS	
**2376**	chr15	95498390	.	CA	C	49,52	PASS	LINC00924
**532**	chr1	2,07E+08	rs7555739	G	T	52,18	PASS	PFKFB2
**1075**	chr11	6943642	rs1386606	G	A	51,07	PASS	ZNF215
**5184**	chr3	66362931	rs7576627	T	G	45,64	PASS	SLC25A26
**6716**	chr7	66994210	rs1139939	A	G	32,06	PASS	SBDS
**474**	chr1	1,78E+08	rs1913400	C	A	47,97	PASS	CRYZL2P
**3993**	chr2	1,22E+08	rs5435278	G	A	48,93	PASS	LINC01823
**342**	chr1	1,45E+08	rs1158745	G	A	8,88	PASS	PPIAL4D
**366**	chr1	1,49E+08	rs1271530	G	C	12,16	PASS	NBPF14
**634**	chr1	2,48E+08	rs1022493	C	A	46,92	PASS	TRIM58
**763**	chr10	47310703	.	T	A	41,24	PASS	GDF10
**1067**	chr11	6390694	rs7862045	G	A	43,85	PASS	SMPD1
**2345**	chr15	86907271	rs7820573	C	T	46,41	PASS	AGBL1
**3022**	chr17	50763441	rs1430312	G	A	52,15	PASS	ANKRD40C
**3346**	chr19	2917881	rs7483780	T	G	36	PASS	ZNF57
**3438**	chr19	15537409	.	G	A	50,18	PASS	CYP4F22
**4148**	chr2	2,08E+08	rs2022337	G	C	47,35	PASS	CRYGD
**5782**	chr5	10448263	rs4128036	T	A	50,99	PASS	ROPN1L
**7563**	chrX	2752332	rs2014412	C	T	28,05	PASS	XG
**4651**	chr21	43063074	.	A	ATGATCTG	10,01	PASS	CBS

MQ	QD	DP	FS	SOR	Cases	Controls	FORMAT
250	1,47	34	18,915	0,761	0,1,1	0,0,0	GT:AD:AF:I
248,95	1,39	36	17,294	0,774	0,1,1	0,0,0	GT:AD:AF:I
168,24	4,1	10	14,771	3,897	0,1,1	0,0,0	GT:AD:AF:I
250	1,22	41	4,922	0,208	0,1,1	0,0,0	GT:AD:AF:I
248,76	1,25	40	2,795	1,177	0,1,1	0,0,0	GT:AD:AF:I
250	0,06	2	0	0,693	0,1,1	0,0,0	GT:AD:AF:I
250	0,04	2	0	0,693	0,1,1	0,0,0	GT:AD:AF:I
190,26	1,51	33	5,418	0,868	0,1,1	0,0,0	GT:AD:AF:I
7,07	5,34	6	0	0,693	1,0,1	0,0,0	GT:AD:AF:I
7,07	5,34	6	0	0,693	1,0,1	0,0,0	GT:AD:AF:I
250	11,75	4	0	2,303	0,1,1	0,0,0	GT:AD:AF:I
187,62	2,5	20	0	1,022	0,1,1	0,0,0	GT:AD:AF:I
250	0,94	53	1,075	0,997	0,1,1	0,0,0	GT:AD:AF:I
77,44	0,74	42	18,675	3,434	0,1,1	0,0,0	GT:AD:AF:I
250	3,13	16	0	0,582	0,1,1	0,0,0	GT:AD:AF:I
250	1,77	27	10,488	2,011	0,1,1	0,0,0	GT:AD:AF:I
88,92	0,3	53	1,352	1,047	0,1,1	0,0,0	GT:AD:AF:I
247,29	1,19	42	5,721	1,647	0,1,1	0,0,0	GT:AD:AF:I
250	1,09	45	1,275	0,484	0,1,1	0,0,0	GT:AD:AF:I
250	0,64	75	0,88	0,88	0,1,1	0,0,0	GT:AD:AF:I
250	1	40	0	0,479	0,1,1	0,0,0	GT:AD:AF:I
243,06	1	46	0	0,657	0,1,1	0,0,0	GT:AD:AF:I
250	0,98	51	0	0,728	0,1,1	0,0,0	GT:AD:AF:I
246,89	0,98	51	0	0,742	0,1,1	0,0,0	GT:AD:AF:I
1,94	3,2	4	0	1,609	1,0,2	0,0,0	GT:AD:AF:I
250	1,04	48	0	0,748	0,1,1	0,0,0	GT:AD:AF:I
239,98	1,28	39	8,809	2,531	0,1,1	0,0,0	GT:AD:AF:I
221,63	1,19	42	2,693	0,572	0,1,1	0,0,0	GT:AD:AF:I
29,59	3,99	24	0	4,615	1,0,2	0,0,0	GT:AD:AF:I
247,64	0,9	53	5,704	0,585	0,1,1	0,0,0	GT:AD:AF:I
250	1,39	36	0	0,914	0,1,1	0,0,0	GT:AD:AF:I
250	2,05	24	0	0,917	0,1,1	0,0,0	GT:AD:AF:I
158,44	0,65	73	8,254	0,858	0,1,1	0,0,0	GT:AD:AF:I
250	0,85	59	1,02	0,469	0,1,1	0,0,0	GT:AD:AF:I
250	1,35	37	13,111	1,326	0,1,1	0,0,0	GT:AD:AF:I
250	1,56	32	1,412	0,34	0,1,1	0,0,0	GT:AD:AF:I
31,54	1,04	28	10,089	2,876	0,1,1	0,0,0	GT:AD:AF:I
250	1,22	41	2,694	0,709	0,1,1	0,0,0	GT:AD:AF:I
250	1,14	44	1,116	0,54	0,1,1	0,0,0	GT:AD:AF:I
250	1,34	37	0	0,619	0,1,1	0,0,0	GT:AD:AF:I
250	2,48	20	3,916	1,334	0,1,1	0,0,0	GT:AD:AF:I
87,32	1,07	44	0	0,693	0,1,1	0,0,0	GT:AD:AF:I
249,15	0,81	61	0,974	0,544	0,1,1	0,0,0	GT:AD:AF:I
250	1,06	47	2,552	0,763	0,1,1	0,0,0	GT:AD:AF:I
250	1,85	27	5,904	0,959	0,1,1	0,0,0	GT:AD:AF:I
250	2,27	22	0	0,804	0,1,1	0,0,0	GT:AD:AF:I
250	1,16	43	0	0,551	0,1,1	0,0,0	GT:AD:AF:I
250	1,35	37	3,683	0,859	0,1,1	0,0,0	GT:AD:AF:I
250	2,03	24	1,674	0,942	0,1,1	0,0,0	GT:AD:AF:I

250	0,21	16	0	2,303	0,1,1	0,0,0	GT:AD:AF:I
250	1,47	33	0	0,486	0,1,1	0,0,0	GT:AD:AF:I
250	2,5	20	0	0,666	0,1,1	0,0,0	GT:AD:AF:I
250	1,85	27	6,501	1,65	0,1,1	0,0,0	GT:AD:AF:I
250	2,94	17	2,077	1,426	0,1,1	0,0,0	GT:AD:AF:I
250	2,25	22	1,74	0,925	0,1,1	0,0,0	GT:AD:AF:I
250	1,22	41	6,909	0,621	0,1,1	0,0,0	GT:AD:AF:I
244,13	3,57	14	0	0,593	0,1,1	0,0,0	GT:AD:AF:I
11	7,95	2	0	2,303	1,0,2	0,0,0	GT:AD:AF:I
13,08	0,12	12	6,892	2,815	0,1,1	0,0,0	GT:AD:AF:I
250	1,35	37	9,94	2,07	0,1,1	0,0,0	GT:AD:AF:I
250	0,86	58	2,15	0,46	0,1,1	0,0,0	GT:AD:AF:I
14	9,42	2	0	2,303	1,0,2	0,0,0	GT:AD:AF:I
214,29	1,1	43	0	0,828	0,1,1	0,0,0	GT:AD:AF:I
249,81	0,77	65	0	0,749	0,1,1	0,0,0	GT:AD:AF:I
246,47	2,02	24	1,685	1,23	0,1,1	0,0,0	GT:AD:AF:I
250	1,43	35	1,399	0,392	0,1,1	0,0,0	GT:AD:AF:I
250	1,47	33	14,829	2,238	0,1,1	0,0,0	GT:AD:AF:I
158,51	0,69	19	0	0,892	0,1,1	0,0,0	GT:AD:AF:I
246,37	2,34	21	0	0,399	0,1,1	0,0,0	GT:AD:AF:I
250	1,91	25	0	0,936	0,1,1	0,0,0	GT:AD:AF:I
250	3,53	14	2,027	1,329	0,1,1	0,0,0	GT:AD:AF:I
250	4,38	11	9,622	0,016	0,1,1	0,0,0	GT:AD:AF:I
13,65	1,32	18	0	0,832	0,1,1	0,0,0	GT:AD:AF:I
250	1,41	35	4,977	0,663	0,1,1	0,0,0	GT:AD:AF:I
248,25	0,87	55	1,059	0,625	0,1,1	0,0,0	GT:AD:AF:I
250	0,3	12	0	1,179	0,1,1	0,0,0	GT:AD:AF:I
250	2,08	24	9,791	0,78	0,1,1	0,0,0	GT:AD:AF:I
250	0,74	66	0,942	0,739	0,1,1	0,0,0	GT:AD:AF:I
250	0,84	58	0	0,807	0,1,1	0,0,0	GT:AD:AF:I
250	0,76	63	0,949	0,564	0,1,1	0,0,0	GT:AD:AF:I
250	1,6	24	0	0,593	0,1,1	0,0,0	GT:AD:AF:I
250	2,63	19	11,563	2,233	0,1,1	0,0,0	GT:AD:AF:I
244,77	1,49	32	1,47	1,179	0,1,1	0,0,0	GT:AD:AF:I
40,17	2,96	14	2,363	1,288	0,1,1	0,0,0	GT:AD:AF:I
250	1,96	25	0	0,936	0,1,1	0,0,0	GT:AD:AF:I
250	1,36	36	0	0,735	0,1,1	0,0,0	GT:AD:AF:I
250	2,07	24	6,204	1,763	0,1,1	0,0,0	GT:AD:AF:I
139,22	1,68	26	3,992	1,911	0,1,1	0,0,0	GT:AD:AF:I
250	1,19	41	1,202	0,481	0,1,1	0,0,0	GT:AD:AF:I
250	1	50	13,467	0,839	0,1,1	0,0,0	GT:AD:AF:I
25,59	1,81	19	2,017	0,51	0,1,1	0,0,0	GT:AD:AF:I
248,81	0,94	51	0	0,704	0,1,1	0,0,0	GT:AD:AF:I
47,38	1,24	35	3,344	0,23	0,1,1	0,0,0	GT:AD:AF:I
250	3,33	15	13,919	2,788	0,1,1	0,0,0	GT:AD:AF:I
250	0,98	51	0	0,824	0,1,1	0,0,0	GT:AD:AF:I
250	1,84	24	1,893	0,287	0,1,1	0,0,0	GT:AD:AF:I
230,38	3,83	13	0	1,022	0,1,1	0,0,0	GT:AD:AF:I
250	3,57	14	0	0,79	0,1,1	0,0,0	GT:AD:AF:I
250	1,23	38	3,01	1,473	0,1,1	0,0,0	GT:AD:AF:I

249,97	1,34	37	0	0,839	0,1,1	0,0,0	GT:AD:AF:I
245,56	2,5	20	7,06	0,407	0,1,1	0,0,0	GT:AD:AF:I
250	1,67	30	3,352	0,486	0,1,1	0,0,0	GT:AD:AF:I
250	2,36	21	0	0,551	0,1,1	0,0,0	GT:AD:AF:I
250	0,96	52	0	0,709	0,1,1	0,0,0	GT:AD:AF:I
250	1,39	36	1,325	1,127	0,1,1	0,0,0	GT:AD:AF:I
250	1,04	48	2,509	1,179	0,1,1	0,0,0	GT:AD:AF:I
250	1,09	46	0	0,846	0,1,1	0,0,0	GT:AD:AF:I
250	1,28	39	2,875	0,296	0,1,1	0,0,0	GT:AD:AF:I
247,21	0,79	63	0	0,689	0,1,1	0,0,0	GT:AD:AF:I
250	1,25	40	1,422	0,412	0,1,1	0,0,0	GT:AD:AF:I
250	0,88	57	5,324	0,803	0,1,1	0,0,0	GT:AD:AF:I
250	0,88	57	2,238	1,042	0,1,1	0,0,0	GT:AD:AF:I
245,5	0,93	54	2,346	0,991	0,1,1	0,0,0	GT:AD:AF:I
248,64	0,85	58	1,019	0,898	0,1,1	0,0,0	GT:AD:AF:I
250	2,36	20	4,561	1,518	0,1,1	0,0,0	GT:AD:AF:I
250	2,81	16	0	0,527	0,1,1	0,0,0	GT:AD:AF:I
250	1,31	38	9,513	1,815	0,1,1	0,0,0	GT:AD:AF:I
250	2,08	24	14,893	2,959	0,1,1	0,0,0	GT:AD:AF:I
241,92	2,5	20	4,32	1,506	0,1,1	0,0,0	GT:AD:AF:I
250	1,2	40	1,368	1,007	0,1,1	0,0,0	GT:AD:AF:I
250	1,28	39	6,884	1,138	0,1,1	0,0,0	GT:AD:AF:I
250	1,11	45	6,369	1,579	0,1,1	0,0,0	GT:AD:AF:I
131,99	2,53	19	0	1,022	0,1,1	0,0,0	GT:AD:AF:I
230,7	3,13	16	0	0,346	0,1,1	0,0,0	GT:AD:AF:I
250	1,07	12	0	1,721	0,1,1	0,0,0	GT:AD:AF:I
250	1,25	40	5,217	0,166	0,1,1	0,0,0	GT:AD:AF:I
250	0,88	56	5,473	0,809	0,1,1	0,0,0	GT:AD:AF:I
250	0,82	61	9,099	0,523	0,1,1	0,0,0	GT:AD:AF:I
250	0,96	52	7,66	1,583	0,1,1	0,0,0	GT:AD:AF:I
250	1,43	35	4,982	1,022	0,1,1	0,0,0	GT:AD:AF:I
249,98	1,09	46	8,917	0,523	0,1,1	0,0,0	GT:AD:AF:I
249,32	1,85	27	1,583	0,346	0,1,1	0,0,0	GT:AD:AF:I
250	1,92	25	6,455	1,502	0,1,1	0,0,0	GT:AD:AF:I
250	2,07	24	13,262	2,447	0,1,1	0,0,0	GT:AD:AF:I
250	1,22	38	1,227	0,75	0,1,1	0,0,0	GT:AD:AF:I
249,12	1,47	34	5,287	0,895	0,1,1	0,0,0	GT:AD:AF:I
250	1,43	35	3,529	1,609	0,1,1	0,0,0	GT:AD:AF:I
250	1,21	41	0	0,646	0,1,1	0,0,0	GT:AD:AF:I
250	2,27	22	6,897	0,105	0,1,1	0,0,0	GT:AD:AF:I
250	1	50	0	0,638	0,1,1	0,0,0	GT:AD:AF:I
250	1,52	33	5,46	0,525	0,1,1	0,0,0	GT:AD:AF:I
250	1,04	48	1,101	0,518	0,1,1	0,0,0	GT:AD:AF:I
250	1,91	25	1,569	0,399	0,1,1	0,0,0	GT:AD:AF:I
52,48	11,37	3	0	1,179	1,0,2	0,0,0	GT:AD:AF:I
48,06	1,07	12	0	0,105	0,1,1	0,0,0	GT:AD:AF:I
250	2,2	22	0	1,022	0,1,1	0,0,0	GT:AD:AF:I
244,34	2,69	18	0	0,832	0,1,1	0,0,0	GT:AD:AF:I
250	1,61	31	3,846	1,802	0,1,1	0,0,0	GT:AD:AF:I
249,89	1,38	35	0	0,631	0,1,1	0,0,0	GT:AD:AF:I

250	1,16	40	1,309	1,148	0,1,1	0,0,0	GT:AD:AF:I
15,57	1,79	2	0	0,693	0,1,1	0,0,0	GT:AD:AF:I
183,09	1,14	35	0	0,549	0,1,1	0,0,0	GT:AD:AF:I
250	1,24	40	4,717	0,402	0,1,1	0,0,0	GT:AD:AF:I
233,67	1,51	31	0	0,602	0,1,1	0,0,0	GT:AD:AF:I
229,43	1,33	36	6,982	2,465	0,1,1	0,0,0	GT:AD:AF:I
250	3,57	14	0	1,27	0,1,1	0,0,0	GT:AD:AF:I
250	2,08	24	6,204	0,408	0,1,1	0,0,0	GT:AD:AF:I
250	3,32	15	0	0,941	0,1,1	0,0,0	GT:AD:AF:I
250	1,7	27	3,492	1,445	0,1,1	0,0,0	GT:AD:AF:I
250	1,21	41	7,005	1,981	0,1,1	0,0,0	GT:AD:AF:I
250	1,14	36	1,356	1,022	0,1,1	0,0,0	GT:AD:AF:I
250	1,1	44	0	0,76	0,1,1	0,0,0	GT:AD:AF:I
248,45	0,82	61	0,984	0,623	0,1,1	0,0,0	GT:AD:AF:I
250	1,41	35	5,138	1,574	0,1,1	0,0,0	GT:AD:AF:I
250	1,47	34	14,489	2,499	0,1,1	0,0,0	GT:AD:AF:I
250	1,61	31	8,08	1,502	0,1,1	0,0,0	GT:AD:AF:I
249,9	1,67	30	0	0,61	0,1,1	0,0,0	GT:AD:AF:I
250	2,17	23	1,76	1,347	0,1,1	0,0,0	GT:AD:AF:I
250	1,72	29	0	0,963	0,1,1	0,0,0	GT:AD:AF:I
247,62	1	50	2,41	1,071	0,1,1	0,0,0	GT:AD:AF:I
250	1,55	32	5,121	1,428	0,1,1	0,0,0	GT:AD:AF:I
250	5	10	2,43	0,223	0,1,1	0,0,0	GT:AD:AF:I
14	9,42	2	0	2,303	1,0,2	0,0,0	GT:AD:AF:I
7,68	3,43	12	0	5,136	1,0,2	0,0,0	GT:AD:AF:I
21,29	1,66	16	2,307	1,721	0,1,1	0,0,0	GT:AD:AF:I
250	1,21	41	0	0,853	0,1,1	0,0,0	GT:AD:AF:I
248,06	2,17	23	6,897	0,105	0,1,1	0,0,0	GT:AD:AF:I
250	1,47	34	0	0,61	0,1,1	0,0,0	GT:AD:AF:I
250	1,61	31	1,643	1,292	0,1,1	0,0,0	GT:AD:AF:I
248,81	3,71	13	0	1,022	0,1,1	0,0,0	GT:AD:AF:I
250	1,32	38	13,032	1,458	0,1,1	0,0,0	GT:AD:AF:I
250	1,85	27	1,583	0,346	0,1,1	0,0,0	GT:AD:AF:I
250	1,59	31	3,192	0,78	0,1,1	0,0,0	GT:AD:AF:I
250	1,85	27	3,473	0,741	0,1,1	0,0,0	GT:AD:AF:I
250	3,6	12	0	0,941	0,1,1	0,0,0	GT:AD:AF:I
250	0,97	50	0	0,569	0,1,1	0,0,0	GT:AD:AF:I
250	0,84	59	2,188	0,954	0,1,1	0,0,0	GT:AD:AF:I
250	0,84	58	5,342	1,445	0,1,1	0,0,0	GT:AD:AF:I
250	1,14	44	2,498	0,568	0,1,1	0,0,0	GT:AD:AF:I
68,03	7,7	9	0	4,615	1,0,2	0,0,0	GT:AD:AF:I
58,62	7,92	8	0	4,407	1,0,2	0,0,0	GT:AD:AF:I
73,88	9,63	2	0	2,303	1,0,2	0,0,0	GT:AD:AF:I
46,02	0,35	3	0	2,833	1,0,2	0,0,0	GT:AD:AF:I
46,02	0,35	3	0	2,833	1,0,2	0,0,0	GT:AD:AF:I
105,66	0	7	0	0,892	0,1,1	0,0,0	GT:AD:AF:I
13,38	0,43	23	7,402	0,09	0,1,1	0,0,0	GT:AD:AF:I
21,18	3,35	22	0	1,548	1,0,2	0,0,0	GT:AD:AF:I
12,3	1,06	22	0	0,191	0,1,1	0,0,0	GT:AD:AF:I
12,17	8,56	2	0	0,693	1,0,2	0,0,0	GT:AD:AF:I

250	1,14	44	15,166	0,636	0,1,1	0,0,0	GT:AD:AF:I
250	1,55	31	5,608	1,541	0,1,1	0,0,0	GT:AD:AF:I
250	1,56	31	3,256	1,071	0,1,1	0,0,0	GT:AD:AF:I
248,11	1,56	32	0	0,874	0,1,1	0,0,0	GT:AD:AF:I
248,75	1,39	36	0	0,683	0,1,1	0,0,0	GT:AD:AF:I
250	1,29	38	1,531	0,309	0,1,1	0,0,0	GT:AD:AF:I
250	4,11	12	0	0,76	0,1,1	0,0,0	GT:AD:AF:I
250	1,56	32	1,385	1,002	0,1,1	0,0,0	GT:AD:AF:I
16,81	1,19	10	0	0,105	0,1,1	0,0,0	GT:AD:AF:I
250	1,32	38	0	0,711	0,1,1	0,0,0	GT:AD:AF:I
240,13	1,11	45	2,727	1,255	0,1,1	0,0,0	GT:AD:AF:I
250	1,09	46	1,221	1,038	0,1,1	0,0,0	GT:AD:AF:I
250	0,79	61	5,524	0,26	0,1,1	0,0,0	GT:AD:AF:I
249,42	0,9	53	3,958	1,426	0,1,1	0,0,0	GT:AD:AF:I
250	1,08	46	11,237	1,118	0,1,1	0,0,0	GT:AD:AF:I
247,6	1,09	46	1,199	0,521	0,1,1	0,0,0	GT:AD:AF:I
250	1,11	45	2,549	0,993	0,1,1	0,0,0	GT:AD:AF:I
250	0,82	57	7,5	1,453	0,1,1	0,0,0	GT:AD:AF:I
250	1,35	37	0	0,941	0,1,1	0,0,0	GT:AD:AF:I
250	1,09	43	2,67	1,165	0,1,1	0,0,0	GT:AD:AF:I
247,17	1,19	42	0	0,678	0,1,1	0,0,0	GT:AD:AF:I
250	0,66	49	10,504	0,098	0,1,1	0,0,0	GT:AD:AF:I
250	0,77	60	1	0,515	0,1,1	0,0,0	GT:AD:AF:I
28,74	1,98	20	0	1,112	0,1,1	0,0,0	GT:AD:AF:I
250	0,77	65	0,97	0,91	0,1,1	0,0,0	GT:AD:AF:I
250	2,94	17	2,197	0,283	0,1,1	0,0,0	GT:AD:AF:I
250	1,87	26	14,152	0,341	0,1,1	0,0,0	GT:AD:AF:I
250	2,63	19	4,713	0,148	0,1,1	0,0,0	GT:AD:AF:I
249,24	1,5	30	0	0,754	0,1,1	0,0,0	GT:AD:AF:I
17,72	6,13	5	0	0,446	0,1,1	0,0,0	GT:AD:AF:I
250	2,27	22	1,792	1,329	0,1,1	0,0,0	GT:AD:AF:I
250	1,72	29	0	0,631	0,1,1	0,0,0	GT:AD:AF:I
250	1,26	37	1,405	1,123	0,1,1	0,0,0	GT:AD:AF:I
250	1,72	29	0	0,836	0,1,1	0,0,0	GT:AD:AF:I
14	2,14	6	0	0,307	0,1,1	0,0,0	GT:AD:AF:I
48,59	0,89	39	1,397	0,409	0,1,1	0,0,0	GT:AD:AF:I
250	1,63	30	1,419	1,038	0,1,1	0,0,0	GT:AD:AF:I
250	1,35	37	2,944	1,348	0,1,1	0,0,0	GT:AD:AF:I
250	1,08	46	6,642	0,174	0,1,1	0,0,0	GT:AD:AF:I
250	1,51	33	10,736	0,987	0,1,1	0,0,0	GT:AD:AF:I
249,82	0,65	76	3,093	0,586	0,1,1	0,0,0	GT:AD:AF:I
249,8	0,72	69	3,224	1,096	0,1,1	0,0,0	GT:AD:AF:I
249,45	0,72	69	9,936	2,012	0,1,1	0,0,0	GT:AD:AF:I
249,88	0,87	50	0	0,749	0,1,1	0,0,0	GT:AD:AF:I
250	0,74	67	3,384	1,136	0,1,1	0,0,0	GT:AD:AF:I
250	1,09	46	6,311	1,455	0,1,1	0,0,0	GT:AD:AF:I
250	0,91	55	0	0,703	0,1,1	0,0,0	GT:AD:AF:I
249,91	1,19	42	0	0,846	0,1,1	0,0,0	GT:AD:AF:I
250	1,83	23	7,546	2,584	0,1,1	0,0,0	GT:AD:AF:I
250	1,06	47	0	0,713	0,1,1	0,0,0	GT:AD:AF:I

246,61	1,35	37	1,289	1,018	0,1,1	0,0,0	GT:AD:AF:I
250	1,19	42	1,204	0,928	0,1,1	0,0,0	GT:AD:AF:I
250	1,55	32	0	0,551	0,1,1	0,0,0	GT:AD:AF:I
250	1,66	29	0	0,892	0,1,1	0,0,0	GT:AD:AF:I
83,73	0,42	40	20,225	4,403	0,1,1	0,0,0	GT:AD:AF:I
47,96	9,47	6	0	3,912	1,0,2	0,0,0	GT:AD:AF:I
250	1	50	1,101	0,704	0,1,1	0,0,0	GT:AD:AF:I
250	0,82	61	0	0,646	0,1,1	0,0,0	GT:AD:AF:I
205,7	1,52	33	1,533	1,276	0,1,1	0,0,0	GT:AD:AF:I
250	2,85	17	1,957	0,729	0,1,1	0,0,0	GT:AD:AF:I
250	1,06	44	0	0,693	0,1,1	0,0,0	GT:AD:AF:I
250	1,61	31	11,196	1,62	0,1,1	0,0,0	GT:AD:AF:I
250	0,96	52	0	0,588	0,1,1	0,0,0	GT:AD:AF:I
250	0,93	54	4,079	0,297	0,1,1	0,0,0	GT:AD:AF:I
250	1,13	44	0	0,56	0,1,1	0,0,0	GT:AD:AF:I
250	0,82	55	5,726	0,557	0,1,1	0,0,0	GT:AD:AF:I
250	2,41	20	1,885	0,269	0,1,1	0,0,0	GT:AD:AF:I
250	1,72	29	1,441	0,446	0,1,1	0,0,0	GT:AD:AF:I
250	1,39	36	7,719	2,093	0,1,1	0,0,0	GT:AD:AF:I
248,16	2	25	0	0,569	0,1,1	0,0,0	GT:AD:AF:I
250	1,02	49	2,692	0,363	0,1,1	0,0,0	GT:AD:AF:I
250	0,79	63	0	0,752	0,1,1	0,0,0	GT:AD:AF:I
250	1,01	48	1,101	0,905	0,1,1	0,0,0	GT:AD:AF:I
250	0,72	65	2,069	0,883	0,1,1	0,0,0	GT:AD:AF:I
250	0,75	67	6,493	0,596	0,1,1	0,0,0	GT:AD:AF:I
36,13	5,64	9	0	3,056	1,0,2	0,0,0	GT:AD:AF:I
36,13	3,89	9	0	0,859	0,1,1	0,0,0	GT:AD:AF:I
27,24	7,25	8	0	1,863	1,0,2	0,0,0	GT:AD:AF:I
27,24	7,25	8	0	1,863	1,0,2	0,0,0	GT:AD:AF:I
250	0,88	52	4,154	0,26	0,1,1	0,0,0	GT:AD:AF:I
250	0,91	44	1,263	1,013	0,1,1	0,0,0	GT:AD:AF:I
42,69	4,18	10	0	0,85	0,1,1	0,0,0	GT:AD:AF:I
250	0,98	51	2,487	0,412	0,1,1	0,0,0	GT:AD:AF:I
249,7	0,74	67	0,937	0,906	0,1,1	0,0,0	GT:AD:AF:I
250	0,93	54	3,966	0,321	0,1,1	0,0,0	GT:AD:AF:I
250	1,27	39	15,896	1,292	0,1,1	0,0,0	GT:AD:AF:I
223,57	1,06	47	1,151	0,499	0,1,1	0,0,0	GT:AD:AF:I
250	1,15	43	0	0,616	0,1,1	0,0,0	GT:AD:AF:I
248,45	0,98	51	3,975	0,56	0,1,1	0,0,0	GT:AD:AF:I
250	3,95	50	0	1,157	1,0,2	0,0,0	GT:AD:AF:I
250	1,22	41	1,216	0,446	0,1,1	0,0,0	GT:AD:AF:I
246,6	0,94	36	0	0,905	0,1,1	0,0,0	GT:AD:AF:I
127,4	2,01	14	6,56	3,258	0,1,1	0,0,0	GT:AD:AF:I
238,81	2,27	22	9,185	0,031	0,1,1	0,0,0	GT:AD:AF:I
250	1,47	34	0	0,497	0,1,1	0,0,0	GT:AD:AF:I
250	0,94	53	4,124	1,414	0,1,1	0,0,0	GT:AD:AF:I
250	0,8	60	2,228	0,436	0,1,1	0,0,0	GT:AD:AF:I
250	1,52	33	0	0,569	0,1,1	0,0,0	GT:AD:AF:I
250	1	50	20,524	2,334	0,1,1	0,0,0	GT:AD:AF:I
45,93	1,2	37	34,371	0,337	0,1,1	0,0,0	GT:AD:AF:I

40,84	1,55	27	14,328	3,106	0,1,1	0,0,0	GT:AD:AF:I
68,7	0,83	50	0	0,743	0,1,1	0,0,0	GT:AD:AF:I
250	1,04	26	1,974	0,863	0,1,1	0,0,0	GT:AD:AF:I
192,1	1,35	37	9,866	0,916	0,1,1	0,0,0	GT:AD:AF:I
250	0,91	51	1,083	0,677	0,1,1	0,0,0	GT:AD:AF:I
248,34	0,88	57	0	0,627	0,1,1	0,0,0	GT:AD:AF:I
250	2,88	17	2,062	1,609	0,1,1	0,0,0	GT:AD:AF:I
250	1,25	40	0	0,668	0,1,1	0,0,0	GT:AD:AF:I
250	1,47	34	0	0,527	0,1,1	0,0,0	GT:AD:AF:I
250	1,35	37	0	0,536	0,1,1	0,0,0	GT:AD:AF:I
250	1,47	34	3,051	0,307	0,1,1	0,0,0	GT:AD:AF:I
250	2,08	24	1,569	0,412	0,1,1	0,0,0	GT:AD:AF:I
250	1,32	38	16,948	0,852	0,1,1	0,0,0	GT:AD:AF:I
221,78	1,35	37	0	0,77	0,1,1	0,0,0	GT:AD:AF:I
250	1,16	43	4,651	1,528	0,1,1	0,0,0	GT:AD:AF:I
250	1,19	42	6,462	0,781	0,1,1	0,0,0	GT:AD:AF:I
250	2,58	19	1,984	1,609	0,1,1	0,0,0	GT:AD:AF:I
250	1,81	22	4,589	1,158	0,1,1	0,0,0	GT:AD:AF:I
250	1,66	30	1,455	0,893	0,1,1	0,0,0	GT:AD:AF:I
36,4	0,37	14	0	1,426	0,1,1	0,0,0	GT:AD:AF:I
94,68	6,69	7	0	0,368	0,1,1	0,0,0	GT:AD:AF:I
250	1,25	40	1,278	0,856	0,1,1	0,0,0	GT:AD:AF:I
18,41	3,97	3	0	1,179	0,1,1	0,0,0	GT:AD:AF:I
18,41	3,97	3	0	1,179	0,1,1	0,0,0	GT:AD:AF:I
14	1,77	2	0	0,693	0,1,1	0,0,0	GT:AD:AF:I
250	0,92	54	3,84	0,574	0,1,1	0,0,0	GT:AD:AF:I
250	1,53	32	5,576	0,169	0,1,1	0,0,0	GT:AD:AF:I
250	1,87	10	0	0,368	0,1,1	0,0,0	GT:AD:AF:I
250	2,75	18	0	0,693	0,1,1	0,0,0	GT:AD:AF:I
250	5,53	9	0	1,179	0,1,1	0,0,0	GT:AD:AF:I
250	2,63	19	4,32	0,525	0,1,1	0,0,0	GT:AD:AF:I
137,69	1,31	37	2,864	0,721	0,1,1	0,0,0	GT:AD:AF:I
250	2,38	21	1,808	1,276	0,1,1	0,0,0	GT:AD:AF:I
250	1,72	29	0	0,593	0,1,1	0,0,0	GT:AD:AF:I
211,58	1,11	45	1,174	0,483	0,1,1	0,0,0	GT:AD:AF:I
250	2,63	19	0	0,859	0,1,1	0,0,0	GT:AD:AF:I
250	1,72	29	8,873	0,093	0,1,1	0,0,0	GT:AD:AF:I
250	1,06	47	2,722	0,357	0,1,1	0,0,0	GT:AD:AF:I
250	1,04	47	2,471	0,404	0,1,1	0,0,0	GT:AD:AF:I
250	0,77	65	0	0,714	0,1,1	0,0,0	GT:AD:AF:I
250	0,92	54	1,047	0,741	0,1,1	0,0,0	GT:AD:AF:I
250	1	50	1,085	0,892	0,1,1	0,0,0	GT:AD:AF:I
250	3,39	14	0	0,631	0,1,1	0,0,0	GT:AD:AF:I
101,43	1,74	27	0	0,916	0,1,1	0,0,0	GT:AD:AF:I
250	0,85	54	0	0,582	0,1,1	0,0,0	GT:AD:AF:I
250	1,39	36	4,715	1,493	0,1,1	0,0,0	GT:AD:AF:I
250	0,77	61	7,096	1,609	0,1,1	0,0,0	GT:AD:AF:I
151,6	2,17	23	6,55	2,147	0,1,1	0,0,0	GT:AD:AF:I
250	1,22	41	7,053	1,942	0,1,1	0,0,0	GT:AD:AF:I
250	1,49	32	8,272	1,477	0,1,1	0,0,0	GT:AD:AF:I

250	1,1	44	4,43	0,442	0,1,1	0,0,0	GT:AD:AF:I
250	0,96	52	1,123	1,034	0,1,1	0,0,0	GT:AD:AF:I
250	2,08	24	1,875	0,307	0,1,1	0,0,0	GT:AD:AF:I
250	2,93	17	1,957	1,08	0,1,1	0,0,0	GT:AD:AF:I
250	1,82	27	3,558	1,394	0,1,1	0,0,0	GT:AD:AF:I
250	3,26	15	2,158	1,245	0,1,1	0,0,0	GT:AD:AF:I
250	1,19	42	7,009	1,673	0,1,1	0,0,0	GT:AD:AF:I
250	1,31	27	13,278	3,937	0,1,1	0,0,0	GT:AD:AF:I
250	1,16	43	4,669	1,646	0,1,1	0,0,0	GT:AD:AF:I
249,48	1,43	35	3,274	1,501	0,1,1	0,0,0	GT:AD:AF:I
250	1,16	41	0	0,871	0,1,1	0,0,0	GT:AD:AF:I
250	4,05	45	0	0,929	1,0,2	0,0,0	GT:AD:AF:I
249,92	3,99	47	0	0,919	1,0,2	0,0,0	GT:AD:AF:I
248,79	3,74	64	0	0,892	1,0,2	0,0,0	GT:AD:AF:I
250	0,92	50	1,142	0,955	0,1,1	0,0,0	GT:AD:AF:I
250	1,19	42	0	0,678	0,1,1	0,0,0	GT:AD:AF:I
20,88	15,39	2	0	2,303	1,0,2	0,0,0	GT:AD:AF:I
21,64	0,29	116	5,807	0,399	0,1,1	0,0,0	GT:AD:AF:I
242,14	0,93	54	5,672	1,5	0,1,1	0,0,0	GT:AD:AF:I
250	1,02	46	4,278	0,289	0,1,1	0,0,0	GT:AD:AF:I
250	0,84	57	0	0,711	0,1,1	0,0,0	GT:AD:AF:I
250	1,11	45	2,635	1,128	0,1,1	0,0,0	GT:AD:AF:I
124,84	1,65	28	0	0,784	0,1,1	0,0,0	GT:AD:AF:I
179,37	1,43	35	5,402	1,828	0,1,1	0,0,0	GT:AD:AF:I
250	1,39	36	5,023	0,607	0,1,1	0,0,0	GT:AD:AF:I
250	1,24	39	1,243	0,668	0,1,1	0,0,0	GT:AD:AF:I
250	1,32	38	1,342	1,022	0,1,1	0,0,0	GT:AD:AF:I
250	1,77	28	0	0,74	0,1,1	0,0,0	GT:AD:AF:I
250	1,04	39	0	0,527	0,1,1	0,0,0	GT:AD:AF:I
250	0,95	52	2,379	0,666	0,1,1	0,0,0	GT:AD:AF:I
250	1,26	36	10,583	1,108	0,1,1	0,0,0	GT:AD:AF:I
233,49	1,09	44	18,41	2,371	0,1,1	0,0,0	GT:AD:AF:I
184,95	1,19	42	1,243	0,62	0,1,1	0,0,0	GT:AD:AF:I
250	0,94	53	4,534	1,458	0,1,1	0,0,0	GT:AD:AF:I
249,18	1,19	41	1,202	0,481	0,1,1	0,0,0	GT:AD:AF:I
250	0,83	60	2,177	0,791	0,1,1	0,0,0	GT:AD:AF:I
250	1,56	32	3,27	0,269	0,1,1	0,0,0	GT:AD:AF:I
250	1,14	44	2,527	0,694	0,1,1	0,0,0	GT:AD:AF:I
250	1,39	36	0	0,941	0,1,1	0,0,0	GT:AD:AF:I
241,89	1,65	29	1,585	0,321	0,1,1	0,0,0	GT:AD:AF:I
250	0,98	51	2,369	0,941	0,1,1	0,0,0	GT:AD:AF:I
250	1,04	43	1,194	0,686	0,1,1	0,0,0	GT:AD:AF:I
250	0,88	57	0	0,631	0,1,1	0,0,0	GT:AD:AF:I
250	0,88	57	0	0,776	0,1,1	0,0,0	GT:AD:AF:I
250	1,56	32	11,281	1,608	0,1,1	0,0,0	GT:AD:AF:I
249,86	0,97	50	1,088	0,917	0,1,1	0,0,0	GT:AD:AF:I
250	0,96	52	0	0,576	0,1,1	0,0,0	GT:AD:AF:I
250	1,79	28	3,975	1,721	0,1,1	0,0,0	GT:AD:AF:I
250	9,44	5	0	1,022	0,1,1	0,0,0	GT:AD:AF:I
250	1,38	36	1,315	0,586	0,1,1	0,0,0	GT:AD:AF:I

250	4,17	12	0	1,445	0,1,1	0,0,0	GT:AD:AF:
250	3,33	15	0	0,859	0,1,1	0,0,0	GT:AD:AF:
240,02	3,33	15	2,158	0,223	0,1,1	0,0,0	GT:AD:AF:
250	2,94	17	2,447	0,132	0,1,1	0,0,0	GT:AD:AF:
250	1,92	26	1,545	0,402	0,1,1	0,0,0	GT:AD:AF:
250	1,56	32	0	0,91	0,1,1	0,0,0	GT:AD:AF:
249,53	1,52	33	14,663	0,045	0,1,1	0,0,0	GT:AD:AF:
250	1,92	26	4,178	1,863	0,1,1	0,0,0	GT:AD:AF:
250	2	25	1,58	1,179	0,1,1	0,0,0	GT:AD:AF:
250	1,22	41	1,263	1,136	0,1,1	0,0,0	GT:AD:AF:
250	1,72	29	3,352	1,272	0,1,1	0,0,0	GT:AD:AF:
249,14	1,11	45	11,272	1,168	0,1,1	0,0,0	GT:AD:AF:
186,8	1,12	31	3,796	0,223	0,1,1	0,0,0	GT:AD:AF:
105,83	2,38	20	11,563	2,225	0,1,1	0,0,0	GT:AD:AF:
19,33	0,03	8	0	0,941	0,1,1	0,0,0	GT:AD:AF:
44,49	3,02	15	0	1,179	1,0,2	0,0,0	GT:AD:AF:
44,49	3,02	15	0	1,329	1,0,2	0,0,0	GT:AD:AF:
56,65	3,12	15	0	0,283	0,1,1	0,0,0	GT:AD:AF:
56,65	3,17	15	0	0,527	1,0,2	0,0,0	GT:AD:AF:
116,81	1,26	27	4,222	1,518	0,1,1	0,0,0	GT:AD:AF:
250	1,04	48	0	0,64	0,1,1	0,0,0	GT:AD:AF:
250	0,82	54	0	0,671	0,1,1	0,0,0	GT:AD:AF:
250	0,82	61	5,283	0,287	0,1,1	0,0,0	GT:AD:AF:
249,67	0,79	63	7,071	1,609	0,1,1	0,0,0	GT:AD:AF:
250	1,06	47	0	0,616	0,1,1	0,0,0	GT:AD:AF:
250	5,56	9	0	0,368	0,1,1	0,0,0	GT:AD:AF:
250	3,33	15	2,216	0,273	0,1,1	0,0,0	GT:AD:AF:
250	2,88	17	0	1,112	0,1,1	0,0,0	GT:AD:AF:
250	2,63	19	2,35	1,802	0,1,1	0,0,0	GT:AD:AF:
250	3,33	15	5,021	0,916	0,1,1	0,0,0	GT:AD:AF:
250	2,95	14	0	1,329	0,1,1	0,0,0	GT:AD:AF:
250	1,52	33	3,067	0,928	0,1,1	0,0,0	GT:AD:AF:
218,89	1,63	30	1,484	0,368	0,1,1	0,0,0	GT:AD:AF:
250	1,45	25	7,166	1,121	0,1,1	0,0,0	GT:AD:AF:
250	3,13	16	2,06	0,343	0,1,1	0,0,0	GT:AD:AF:
250	3,13	16	0	0,534	0,1,1	0,0,0	GT:AD:AF:
248,35	1,5	33	0	0,861	0,1,1	0,0,0	GT:AD:AF:
250	2,13	2	0	0,693	0,1,1	0,0,0	GT:AD:AF:
250	1,85	27	0	0,818	0,1,1	0,0,0	GT:AD:AF:
240,58	1,85	27	1,507	1,112	0,1,1	0,0,0	GT:AD:AF:
49,78	2,04	8	0	1,802	0,1,1	0,0,0	GT:AD:AF:
248,42	1,28	36	0	0,883	0,1,1	0,0,0	GT:AD:AF:
250	2,54	18	2,452	0,188	0,1,1	0,0,0	GT:AD:AF:
250	2,48	20	7,574	1,182	0,1,1	0,0,0	GT:AD:AF:
250	2,5	20	0	0,467	0,1,1	0,0,0	GT:AD:AF:
249,5	1,78	27	3,867	0,183	0,1,1	0,0,0	GT:AD:AF:
250	1,72	29	6,429	1,088	0,1,1	0,0,0	GT:AD:AF:
250	1,38	31	3,544	0,892	0,1,1	0,0,0	GT:AD:AF:
243,36	1,28	39	0	0,793	0,1,1	0,0,0	GT:AD:AF:
250	5	10	2,808	1,108	0,1,1	0,0,0	GT:AD:AF:

249,33	2,17	23	3,834	1,015	0,1,1	0,0,0	GT:AD:AF:I
250	3,13	16	0	1,085	0,1,1	0,0,0	GT:AD:AF:I
250	3,84	13	0	0,495	0,1,1	0,0,0	GT:AD:AF:I
250	2,94	17	0	0,859	0,1,1	0,0,0	GT:AD:AF:I
250	3,83	13	2,276	1,229	0,1,1	0,0,0	GT:AD:AF:I
250	1,13	44	0	0,7	0,1,1	0,0,0	GT:AD:AF:I
250	0,67	49	1,178	0,726	0,1,1	0,0,0	GT:AD:AF:I
250	1,47	34	5,294	0,191	0,1,1	0,0,0	GT:AD:AF:I
142,73	0,91	53	2,558	0,351	0,1,1	0,0,0	GT:AD:AF:I
139,7	0,94	50	1,111	0,876	0,1,1	0,0,0	GT:AD:AF:I
63,74	0,86	51	2,388	1,136	0,1,1	0,0,0	GT:AD:AF:I
250	1,09	46	6,632	0,188	0,1,1	0,0,0	GT:AD:AF:I
189,14	0,96	52	2,413	0,521	0,1,1	0,0,0	GT:AD:AF:I
250	2,94	17	0	0,346	0,1,1	0,0,0	GT:AD:AF:I
250	1,67	30	0	0,665	0,1,1	0,0,0	GT:AD:AF:I
250	1,79	28	3,556	1,609	0,1,1	0,0,0	GT:AD:AF:I
250	0,96	52	3,983	1,339	0,1,1	0,0,0	GT:AD:AF:I
250	0,98	50	1,102	0,46	0,1,1	0,0,0	GT:AD:AF:I
250	1,02	49	13,703	0,778	0,1,1	0,0,0	GT:AD:AF:I
250	1,28	39	12,603	2,163	0,1,1	0,0,0	GT:AD:AF:I
250	2,38	21	4,419	0,141	0,1,1	0,0,0	GT:AD:AF:I
250	1,85	27	1,527	1,179	0,1,1	0,0,0	GT:AD:AF:I
250	4,55	11	0	0,693	0,1,1	0,0,0	GT:AD:AF:I
245,49	1,39	36	2,873	0,345	0,1,1	0,0,0	GT:AD:AF:I
218,31	1,62	21	4,706	1,981	0,1,1	0,0,0	GT:AD:AF:I
238,72	1,51	33	0	0,732	0,1,1	0,0,0	GT:AD:AF:I
184,81	1,26	39	0	0,73	0,1,1	0,0,0	GT:AD:AF:I
183,52	1,3	38	1,271	0,936	0,1,1	0,0,0	GT:AD:AF:I
180,81	1,38	35	0	0,727	0,1,1	0,0,0	GT:AD:AF:I
182,35	1,24	39	0	0,813	0,1,1	0,0,0	GT:AD:AF:I
184,05	1,27	38	1,378	1,167	0,1,1	0,0,0	GT:AD:AF:I
189,84	1,18	41	0	0,542	0,1,1	0,0,0	GT:AD:AF:I
185,7	1,25	36	1,315	0,586	0,1,1	0,0,0	GT:AD:AF:I
185,66	1,25	36	1,315	0,586	0,1,1	0,0,0	GT:AD:AF:I
183,47	1,13	38	0	0,655	0,1,1	0,0,0	GT:AD:AF:I
183,47	1,14	38	0	0,548	0,1,1	0,0,0	GT:AD:AF:I
176,62	1,2	36	0	0,781	0,1,1	0,0,0	GT:AD:AF:I
131,22	1,4	33	7,791	1,51	0,1,1	0,0,0	GT:AD:AF:I
162,21	1,19	42	9,458	1,293	0,1,1	0,0,0	GT:AD:AF:I
141,57	1,09	44	1,192	0,99	0,1,1	0,0,0	GT:AD:AF:I
137,8	1,09	41	0	0,871	0,1,1	0,0,0	GT:AD:AF:I
140,75	0,69	58	2,278	1,085	0,1,1	0,0,0	GT:AD:AF:I
150,85	1,3	36	14,508	2,822	0,1,1	0,0,0	GT:AD:AF:I
161,83	1,37	36	5,153	1,863	0,1,1	0,0,0	GT:AD:AF:I
45,38	0,09	26	46,948	4,796	0,1,1	0,0,0	GT:AD:AF:I
250	0,82	56	1,031	0,542	0,1,1	0,0,0	GT:AD:AF:I
250	0,88	57	1,007	0,518	0,1,1	0,0,0	GT:AD:AF:I
250	1,08	45	0	0,686	0,1,1	0,0,0	GT:AD:AF:I
243,3	1,56	32	0	0,656	0,1,1	0,0,0	GT:AD:AF:I
250	1,91	25	13,132	1,429	0,1,1	0,0,0	GT:AD:AF:I

250	3,54	13	0	1,329	0,1,1	0,0,0	GT:AD:AF:I
250	3,33	15	2,158	0,223	0,1,1	0,0,0	GT:AD:AF:I
250	1,87	26	6,329	1,05	0,1,1	0,0,0	GT:AD:AF:I
250	2,17	23	0	0,765	0,1,1	0,0,0	GT:AD:AF:I
250	1,85	27	1,69	0,313	0,1,1	0,0,0	GT:AD:AF:I
250	1,97	25	0	0,716	0,1,1	0,0,0	GT:AD:AF:I
250	2,46	19	0	1,085	0,1,1	0,0,0	GT:AD:AF:I
247,22	1,19	42	7,585	2,012	0,1,1	0,0,0	GT:AD:AF:I
250	1,04	48	11,419	2,303	0,1,1	0,0,0	GT:AD:AF:I
250	1,32	38	1,415	1,088	0,1,1	0,0,0	GT:AD:AF:I
250	0,85	56	2,356	1,171	0,1,1	0,0,0	GT:AD:AF:I
250	1,56	32	0	0,495	0,1,1	0,0,0	GT:AD:AF:I
250	0,72	57	3,871	1,172	0,1,1	0,0,0	GT:AD:AF:I
250	1,61	31	1,398	1,061	0,1,1	0,0,0	GT:AD:AF:I
250	3,53	14	8,616	2,245	0,1,1	0,0,0	GT:AD:AF:I
250	0,74	66	0	0,734	0,1,1	0,0,0	GT:AD:AF:I
250	2,38	21	0	0,804	0,1,1	0,0,0	GT:AD:AF:I
250	1,09	46	0	0,742	0,1,1	0,0,0	GT:AD:AF:I
250	1,67	30	4,103	1,883	0,1,1	0,0,0	GT:AD:AF:I
51,93	0,89	26	10,58	3,056	0,1,1	0,0,0	GT:AD:AF:I
250	1,32	38	6,74	0,378	0,1,1	0,0,0	GT:AD:AF:I
249,55	1,87	26	14,152	0,341	0,1,1	0,0,0	GT:AD:AF:I
250	1,16	39	2,963	0,304	0,1,1	0,0,0	GT:AD:AF:I
250	1,11	43	14,774	1,308	0,1,1	0,0,0	GT:AD:AF:I
42,35	2,25	17	1,957	0,729	0,1,1	0,0,0	GT:AD:AF:I
250	1,28	39	0	0,569	0,1,1	0,0,0	GT:AD:AF:I
250	1,9	26	3,831	1,802	0,1,1	0,0,0	GT:AD:AF:I
249,72	2	25	3,619	1,256	0,1,1	0,0,0	GT:AD:AF:I
248,71	0,96	51	10,695	1,998	0,1,1	0,0,0	GT:AD:AF:I
250	3,13	16	2,158	0,842	0,1,1	0,0,0	GT:AD:AF:I
245,64	0,73	68	3,312	0,815	0,1,1	0,0,0	GT:AD:AF:I
216,03	1,1	45	0	0,709	0,1,1	0,0,0	GT:AD:AF:I
250	0,89	54	0	0,788	0,1,1	0,0,0	GT:AD:AF:I
250	2,5	20	8,022	0,328	0,1,1	0,0,0	GT:AD:AF:I
250	1,77	28	5,59	1,567	0,1,1	0,0,0	GT:AD:AF:I
250	1,82	27	3,473	0,819	0,1,1	0,0,0	GT:AD:AF:I
250	1,25	40	41,024	2,674	0,1,1	0,0,0	GT:AD:AF:I
246,61	1,31	32	0	0,693	0,1,1	0,0,0	GT:AD:AF:I
250	1,02	49	1,155	1,044	0,1,1	0,0,0	GT:AD:AF:I
250	1,03	48	8,361	0,819	0,1,1	0,0,0	GT:AD:AF:I
250	1,61	31	0	0,818	0,1,1	0,0,0	GT:AD:AF:I
242,56	1,43	35	0	0,693	0,1,1	0,0,0	GT:AD:AF:I
250	1,32	38	4,655	0,589	0,1,1	0,0,0	GT:AD:AF:I
250	1,72	29	6,107	0,132	0,1,1	0,0,0	GT:AD:AF:I
250	1,11	45	17,51	1,786	0,1,1	0,0,0	GT:AD:AF:I
250	1,91	26	0	0,693	0,1,1	0,0,0	GT:AD:AF:I
250	1,72	29	0	0,836	0,1,1	0,0,0	GT:AD:AF:I
229,62	1,19	42	19,063	1,811	0,1,1	0,0,0	GT:AD:AF:I
250	2,63	19	17,041	3,02	0,1,1	0,0,0	GT:AD:AF:I
250	2,75	18	7,259	1,784	0,1,1	0,0,0	GT:AD:AF:I

248,59	1,79	28	3,227	0,537	0,1,1	0,0,0	GT:AD:AF:I
250	0,86	58	3,657	0,691	0,1,1	0,0,0	GT:AD:AF:I
250	0,72	68	2,032	0,546	0,1,1	0,0,0	GT:AD:AF:I
249,82	1,04	48	0	0,732	0,1,1	0,0,0	GT:AD:AF:I
250	1,39	36	4,748	0,438	0,1,1	0,0,0	GT:AD:AF:I
250	1,1	44	4,3	1,254	0,1,1	0,0,0	GT:AD:AF:I
250	0,89	56	0	0,582	0,1,1	0,0,0	GT:AD:AF:I
49,32	0,07	27	16,66	4,17	0,1,1	0,0,0	GT:AD:AF:I
246,7	1,22	38	2,944	0,978	0,1,1	0,0,0	GT:AD:AF:I
249,04	1,41	35	0	0,631	0,1,1	0,0,0	GT:AD:AF:I
250	2,38	21	0	0,722	0,1,1	0,0,0	GT:AD:AF:I
250	1,61	31	3,192	0,513	0,1,1	0,0,0	GT:AD:AF:I
250	0,81	62	0	0,59	0,1,1	0,0,0	GT:AD:AF:I
250	1,39	36	1,493	0,409	0,1,1	0,0,0	GT:AD:AF:I
250	2,81	16	0	0,301	0,1,1	0,0,0	GT:AD:AF:I
247,77	1,72	29	1,527	1,329	0,1,1	0,0,0	GT:AD:AF:I
39,71		1023			1,0,2	0,0,0	GT:SQ:AD:
236,69		1191			1,0,2	0,0,0	GT:SQ:AD:
123,57	7,09	12	0	1,445	1,0,1	0,0,0	GT:AD:AF:I
250	5,74	18	0	0,693	1,0,1	0,0,0	GT:AD:AF:I
6,56	5,71	3	0	2,833	1,0,1	0,0,0	GT:AD:AF:I
250	11,34	6	0	1,329	1,0,1	0,0,0	GT:AD:AF:I
38,43	6,37	12	0	1,981	1,0,1	0,0,0	GT:AD:AF:I
25,25	9,7	6	0	0,693	1,0,1	0,0,0	GT:AD:AF:I
7,07	5,34	6	0	0,693	1,0,1	0,0,0	GT:AD:AF:I
7,07	5,34	6	0	0,693	1,0,1	0,0,0	GT:AD:AF:I
250	6,13	16	0	0,693	1,0,1	0,0,0	GT:AD:AF:I
250	6,86	13	0	0,836	1,0,1	0,0,0	GT:AD:AF:I
250	9,26	8	0	2,833	1,0,1	0,0,0	GT:AD:AF:I
250	5,28	22	0	1,329	1,0,1	0,0,0	GT:AD:AF:I
250	4,17	12	2,533	1,518	0,1,1	0,0,0	GT:AD:AF:I
250	1,35	37	4,743	1,272	0,1,1	0,0,0	GT:AD:AF:I
250	2,12	19	0	0,527	0,1,1	0,0,0	GT:AD:AF:I
249,08	2,27	22	0	0,976	0,1,1	0,0,0	GT:AD:AF:I
250	0,98	51	10,983	1,319	0,1,1	0,0,0	GT:AD:AF:I
250	1,72	29	21,093	1,711	0,1,1	0,0,0	GT:AD:AF:I
250	1,16	43	9,057	0,463	0,1,1	0,0,0	GT:AD:AF:I
248,8	1,02	49	4,112	0,411	0,1,1	0,0,0	GT:AD:AF:I
249,57	1,54	32	1,398	0,425	0,1,1	0,0,0	GT:AD:AF:I
54,45	1,1	41	6,747	1,057	0,1,1	0,0,0	GT:AD:AF:I
248,69	1,25	39	13,045	2,122	0,1,1	0,0,0	GT:AD:AF:I
39,51	1,18	33	5,501	1,122	0,1,1	0,0,0	GT:AD:AF:I
250	2,17	23	0	0,551	0,1,1	0,0,0	GT:AD:AF:I
74,15	1,42	29	0	0,741	0,1,1	0,0,0	GT:AD:AF:I
250	1,78	28	0	0,743	0,1,1	0,0,0	GT:AD:AF:I
183,65	1,25	38	1,273	0,957	0,1,1	0,0,0	GT:AD:AF:I
250	1,21	41	1,189	0,914	0,1,1	0,0,0	GT:AD:AF:I
248,04	0,97	51	1,072	0,803	0,1,1	0,0,0	GT:AD:AF:I
98,66	0,74	62	0,962	0,862	0,1,1	0,0,0	GT:AD:AF:I
13,84	2,42	6	3,979	0,838	0,1,1	0,0,0	GT:AD:AF:I

250	1,41	34	0	0,646	0,1,1	0,0,0	GT:AD:AF:
249,84	5,01	25	0	0,941	1,0,1	0,0,0	GT:AD:AF:
12,1	10,9	2	0	2,303	1,0,2	0,0,0	GT:AD:AF:
250	1,85	27	6,219	0,565	0,1,1	0,0,0	GT:AD:AF:
250	2,08	24	19,109	2,035	0,1,1	0,0,0	GT:AD:AF:
247,19	2,38	21	0	0,569	0,1,1	0,0,0	GT:AD:AF:
250	2,48	20	0	0,446	0,1,1	0,0,0	GT:AD:AF:
90,01	1,14	39	0	0,519	0,1,1	0,0,0	GT:AD:AF:
249,54	1	50	5,987	1,131	0,1,1	0,0,0	GT:AD:AF:
250	1,85	27	1,702	0,395	0,1,1	0,0,0	GT:AD:AF:
13,78	1,45	14	2,363	0,223	0,1,1	0,0,0	GT:AD:AF:
18,22	3,6	7	0	0,527	0,1,1	0,0,0	GT:AD:AF:
250	1,04	48	1,119	0,595	0,1,1	0,0,0	GT:AD:AF:
247,11	1,11	42	6,837	1,795	0,1,1	0,0,0	GT:AD:AF:
249,65	1	49	1,092	0,507	0,1,1	0,0,0	GT:AD:AF:
243,58	2,78	18	5,021	1,198	0,1,1	0,0,0	GT:AD:AF:
250	1,56	32	1,34	0,473	0,1,1	0,0,0	GT:AD:AF:
102,91	1,5	32	0	0,948	0,1,1	0,0,0	GT:AD:AF:
246,86	0,96	52	7,905	1,447	0,1,1	0,0,0	GT:AD:AF:
129,7	1,38	36	1,366	0,387	0,1,1	0,0,0	GT:AD:AF:
250	1,35	37	5,128	1,567	0,1,1	0,0,0	GT:AD:AF:
49,34	1,38	30	5,469	1,765	0,1,1	0,0,0	GT:AD:AF:
238,08	0,63	80	6,801	0,983	0,1,1	0,0,0	GT:AD:AF:

21-S-0747	Allele	onsequenc	IMPACT	Gene	eature_typ	Feature	BIOTYPE
0|1:14,20:0.588:34:8,5:6	-	frameshift	HIGH	ENSG0000	Transcript	ENST0000	protein_cc
0|1:16,20:0.556:36:8,5:8	-	frameshift	HIGH	ENSG0000	Transcript	ENST0000	protein_cc
0/1:4,6:0.6:10:2,3:2,3:26	-	frameshift	HIGH	ENSG0000	Transcript	ENST0000	protein_cc
0/1:16,25:0.61:41:11,13:	-	frameshift	HIGH	ENSG0000	Transcript	ENST0000	protein_cc
0/1:19,21:0.525:40:10,10	-	frameshift	HIGH	ENSG0000	Transcript	ENST0000	protein_cc
0/1:1,1:0.5:2:0,1:1,0:3:6,	G	frameshift	HIGH	ENSG0000	Transcript	ENST0000	protein_cc
0/1:1,1:0.5:2:0,1:1,0:6:12	GC	frameshift	HIGH	ENSG0000	Transcript	ENST0000	protein_cc
0/1:17,15:0.469:32:8,4:9,	-	frameshift	HIGH	ENSG0000	Transcript	ENST0000	protein_cc
1:0,6:1:6:0,5:0,1:19:48,0:	-	frameshift	HIGH	ENSG0000	Transcript	ENST0000	protein_cc
1:0,6:1:6:0,5:0,1:26:55,0:	GC	frameshift	HIGH	ENSG0000	Transcript	ENST0000	protein_cc
0/1:2,2:0.5:4:2,2:0,0:23:5	-	inframe_d	MODERAT	ENSG0000	Transcript	ENST0000	protein_cc
0/1:2,3:0.6:5:2,3:0,0:11:4	-	inframe_d	MODERAT	ENSG0000	Transcript	ENST0000	protein_cc
0/1:24,28:0.538:52:12,15	-	inframe_d	MODERAT	ENSG0000	Transcript	ENST0000	protein_cc
0/1:28,11:0.282:39:13,6:	-	inframe_d	MODERAT	ENSG0000	Transcript	ENST0000	protein_cc
0/1:6,9:0.6:15:1,3:5,6:39	-	inframe_d	MODERAT	ENSG0000	Transcript	ENST0000	protein_cc
0/1:14,9:0.391:23:5,2:9,7	-	inframe_d	MODERAT	ENSG0000	Transcript	ENST0000	protein_cc
0/1:35,11:0.239:46:17,5:	-	inframe_d	MODERAT	ENSG0000	Transcript	ENST0000	protein_cc
0/1:11,26:0.703:37:6,13:	-	inframe_d	MODERAT	ENSG0000	Transcript	ENST0000	protein_cc
0/1:24,20:0.455:44:13,10	-	inframe_d	MODERAT	ENSG0000	Transcript	ENST0000	protein_cc
0/1:42,33:0.44:75:25,22:	-	inframe_d	MODERAT	ENSG0000	Transcript	ENST0000	protein_cc
0/1:25,12:0.324:37:15,9:	-	inframe_d	MODERAT	ENSG0000	Transcript	ENST0000	protein_cc
0/1:24,15:0.385:39:11,9:	-	inframe_d	MODERAT	ENSG0000	Transcript	ENST0000	protein_cc
0/1:26,24:0.48:50:11,17:	-	inframe_d	MODERAT	ENSG0000	Transcript	ENST0000	protein_cc
0|1:21,24:0.533:45:11,13	-	inframe_d	MODERAT	ENSG0000	Transcript	ENST0000	protein_cc
1/1:0,4:1:4:0,2:0,2:9:43,1	GTCTTC	inframe_ir	MODERAT	ENSG0000	Transcript	ENST0000	protein_cc
0/1:23,21:0.477:44:10,5:	GTC	inframe_ir	MODERAT	ENSG0000	Transcript	ENST0000	protein_cc
0/1:16,19:0.543:35:4,8:1	GCC	inframe_ir	MODERAT	ENSG0000	Transcript	ENST0000	protein_cc
0/1:19,19:0.5:38:11,10:8,	TGCTGC	inframe_ir	MODERAT	ENSG0000	Transcript	ENST0000	protein_cc
1/1:0,20:1:20:0,8:0,12:26	GGGGCGC	inframe_ir	MODERAT	ENSG0000	Transcript	ENST0000	protein_cc
0/1:29,22:0.431:51:18,13	CAG	inframe_ir	MODERAT	ENSG0000	Transcript	ENST0000	protein_cc
0/1:14,16:0.533:30:3,7:1	GAGCCC	inframe_ir	MODERAT	ENSG0000	Transcript	ENST0000	protein_cc
0/1:13,10:0.435:23:6,8:7,	GAG	inframe_ir	MODERAT	ENSG0000	Transcript	ENST0000	protein_cc
0/1:30,36:0.545:66:19,19	CAG	inframe_ir	MODERAT	ENSG0000	Transcript	ENST0000	protein_cc
0/1:27,32:0.542:59:9,16:	T	missense_	MODERAT	ENSG0000	Transcript	ENST0000	protein_cc
0/1:16,21:0.568:37:6,7:1	T	missense_	MODERAT	ENSG0000	Transcript	ENST0000	protein_cc
0/1:16,16:0.5:32:8,6:8,10	T	missense_	MODERAT	ENSG0000	Transcript	ENST0000	protein_cc
0/1:18,10:0.357:28:11,6:	C	missense_	MODERAT	ENSG0000	Transcript	ENST0000	protein_cc
0/1:21,20:0.488:41:11,10	T	missense_	MODERAT	ENSG0000	Transcript	ENST0000	protein_cc
0/1:22,22:0.5:44:7,12:15,	T	missense_	MODERAT	ENSG0000	Transcript	ENST0000	protein_cc
0/1:20,17:0.459:37:10,11	C	missense_	MODERAT	ENSG0000	Transcript	ENST0000	protein_cc
0/1:11,9:0.45:20:3,4:8,5:	A	missense_	MODERAT	ENSG0000	Transcript	ENST0000	protein_cc
0/1:22,22:0.5:44:10,13:1	A	missense_	MODERAT	ENSG0000	Transcript	ENST0000	protein_cc
0/1:32,29:0.475:61:15,14	A	missense_	MODERAT	ENSG0000	Transcript	ENST0000	protein_cc
0/1:18,29:0.617:47:12,16	A	missense_	MODERAT	ENSG0000	Transcript	ENST0000	protein_cc
0/1:14,13:0.481:27:6,9:8,	G	missense_	MODERAT	ENSG0000	Transcript	ENST0000	protein_cc
0/1:8,14:0.636:22:6,7:2,7	A	missense_	MODERAT	ENSG0000	Transcript	ENST0000	protein_cc
0/1:16,27:0.628:43:9,12:	C	missense_	MODERAT	ENSG0000	Transcript	ENST0000	protein_cc
0/1:8,29:0.784:37:2,9:6,2	A	missense_	MODERAT	ENSG0000	Transcript	ENST0000	protein_cc
0/1:14,10:0.417:24:8,5:6,	A	missense_	MODERAT	ENSG0000	Transcript	ENST0000	protein_cc

0/1:14,2:0.125:16:7,1:7,1	A		missense_	MODERAT	ENSG0000	Transcript	ENST0000(	protein_cc
0/1:19,14:0.424:33:11,9:8	G		missense_	MODERAT	ENSG0000	Transcript	ENST0000(	protein_cc
0/1:7,13:0.65:20:2,6:5,7:	G		missense_	MODERAT	ENSG0000	Transcript	ENST0000(	protein_cc
0/1:10,17:0.63:27:6,7:4,1	T		missense_	MODERAT	ENSG0000	Transcript	ENST0000(	protein_cc
0/1:8,9:0.529:17:7,2:1,7:	A		missense_	MODERAT	ENSG0000	Transcript	ENST0000(	protein_cc
0/1:12,10:0.455:22:7,5:5,	G		missense_	MODERAT	ENSG0000	Transcript	ENST0000(	protein_cc
0/1:15,25:0.625:40:8,12:	G		missense_	MODERAT	ENSG0000	Transcript	ENST0000(	protein_cc
0/1:6,8:0.571:14:3,5:3,3:	A		missense_	MODERAT	ENSG0000	Transcript	ENST0000(	protein_cc
1|1:0,2:1:2:0,0:0,2:9:56,1	C		missense_	MODERAT	ENSG0000	Transcript	ENST0000(	protein_cc
0/1:9,3:0.25:12:2,1:7,2:4	C		missense_	MODERAT	ENSG0000	Transcript	ENST0000(	protein_cc
0/1:17,20:0.541:37:6,8:1	T		missense_	MODERAT	ENSG0000	Transcript	ENST0000(	protein_cc
0/1:28,30:0.517:58:16,14	A		missense_	MODERAT	ENSG0000	Transcript	ENST0000(	protein_cc
1/1:0,2:1:2:0,1:0,1:17:58,	T		missense_	MODERAT	ENSG0000	Transcript	ENST0000(	protein_cc
0/1:25,18:0.419:43:13,8:	G		missense_	MODERAT	ENSG0000	Transcript	ENST0000(	protein_cc
0/1:30,35:0.538:65:9,19:	C		missense_	MODERAT	ENSG0000	Transcript	ENST0000(	protein_cc
0/1:14,10:0.417:24:7,7:7,	G		missense_	MODERAT	ENSG0000	Transcript	ENST0000(	protein_cc
0|1:17,18:0.514:35:10,11	G		missense_	MODERAT	ENSG0000	Transcript	ENST0000(	protein_cc
0/1:19,14:0.424:33:5,9:1	T		missense_	MODERAT	ENSG0000	Transcript	ENST0000(	protein_cc
0/1:16,3:0.158:19:7,3:9,0	G		missense_	MODERAT	ENSG0000	Transcript	ENST0000(	protein_cc
0/1:12,9:0.429:21:5,3:7,6	G		missense_	MODERAT	ENSG0000	Transcript	ENST0000(	protein_cc
0/1:15,10:0.4:25:5,5:10,5	C		missense_	MODERAT	ENSG0000	Transcript	ENST0000(	protein_cc
0/1:8,6:0.429:14:2,5:6,1:	G		missense_	MODERAT	ENSG0000	Transcript	ENST0000(	protein_cc
0/1:7,4:0.364:11:3,2:4,2:	A		missense_	MODERAT	ENSG0000	Transcript	ENST0000(	protein_cc
0/1:11,7:0.389:18:6,2:5,5	C		missense_	MODERAT	ENSG0000	Transcript	ENST0000(	protein_cc
0/1:19,16:0.457:35:8,9:1	A		missense_	MODERAT	ENSG0000	Transcript	ENST0000(	protein_cc
0/1:31,23:0.426:54:17,10	T		missense_	MODERAT	ENSG0000	Transcript	ENST0000(	protein_cc
0/1:10,2:0.167:12:5,2:5,0	C		missense_	MODERAT	ENSG0000	Transcript	ENST0000(	protein_cc
0/1:9,15:0.625:24:6,9:3,6	G		missense_	MODERAT	ENSG0000	Transcript	ENST0000(	protein_cc
0/1:36,30:0.455:66:18,17	G		missense_	MODERAT	ENSG0000	Transcript	ENST0000(	protein_cc
0/1:32,26:0.448:58:15,17	T		missense_	MODERAT	ENSG0000	Transcript	ENST0000(	protein_cc
0/1:35,28:0.444:63:20,17	A		missense_	MODERAT	ENSG0000	Transcript	ENST0000(	protein_cc
0/1:17,6:0.261:23:9,3:8,3	C		missense_	MODERAT	ENSG0000	Transcript	ENST0000(	protein_cc
0/1:9,10:0.526:19:4,5:5,5	C		missense_	MODERAT	ENSG0000	Transcript	ENST0000(	protein_cc
0/1:19,13:0.406:32:12,6:	G		missense_	MODERAT	ENSG0000	Transcript	ENST0000(	protein_cc
0/1:5,9:0.643:14:1,5:4,4:	A		missense_	MODERAT	ENSG0000	Transcript	ENST0000(	protein_cc
0/1:14,11:0.44:25:9,9:5,2	A		missense_	MODERAT	ENSG0000	Transcript	ENST0000(	protein_cc
0/1:20,16:0.444:36:12,10	T		missense_	MODERAT	ENSG0000	Transcript	ENST0000(	protein_cc
0/1:13,11:0.458:24:7,4:6,	A		missense_	MODERAT	ENSG0000	Transcript	ENST0000(	protein_cc
0/1:17,9:0.346:26:9,6:8,3	G		missense_	MODERAT	ENSG0000	Transcript	ENST0000(	protein_cc
0/1:23,18:0.439:41:13,8:	C		missense_	MODERAT	ENSG0000	Transcript	ENST0000(	protein_cc
0/1:24,25:0.51:49:14,11:	G		missense_	MODERAT	ENSG0000	Transcript	ENST0000(	protein_cc
0/1:6,13:0.684:19:1,9:5,4	A		missense_	MODERAT	ENSG0000	Transcript	ENST0000(	protein_cc
0/1:29,22:0.431:51:17,11	C		missense_	MODERAT	ENSG0000	Transcript	ENST0000(	protein_cc
0/1:13,22:0.629:35:5,10:8	G		missense_	MODERAT	ENSG0000	Transcript	ENST0000(	protein_cc
0/1:7,8:0.533:15:3,2:4,6:	G		missense_	MODERAT	ENSG0000	Transcript	ENST0000(	protein_cc
0/1:20,31:0.608:51:8,16:	C		missense_	MODERAT	ENSG0000	Transcript	ENST0000(	protein_cc
0/1:16,8:0.333:24:8,1:8,7	A		missense_	MODERAT	ENSG0000	Transcript	ENST0000(	protein_cc
0/1:7,6:0.462:13:4,3:3,3:	T		missense_	MODERAT	ENSG0000	Transcript	ENST0000(	protein_cc
0/1:7,7:0.5:14:4,1:3,6:44	C		missense_	MODERAT	ENSG0000	Transcript	ENST0000(	protein_cc
0/1:22,15:0.405:37:11,8:	C		missense_	MODERAT	ENSG0000	Transcript	ENST0000(	protein_cc

0/1:20,17:0.459:37:7,11:	T	missense_	MODERAT	ENSG0000	Transcript	ENST0000(	protein_cc
0/1:9,11:0.55:20:4,5:5,6:	C	missense_	MODERAT	ENSG0000	Transcript	ENST0000(	protein_cc
0/1:14,15:0.517:29:6,7:8,	C	missense_	MODERAT	ENSG0000	Transcript	ENST0000(	protein_cc
0/1:11,10:0.476:21:4,4:7,	A	missense_	MODERAT	ENSG0000	Transcript	ENST0000(	protein_cc
0/1:27,25:0.481:52:17,16	A	missense_	MODERAT	ENSG0000	Transcript	ENST0000(	protein_cc
0/1:16,20:0.556:36:10,9:	C	missense_	MODERAT	ENSG0000	Transcript	ENST0000(	protein_cc
0/1:24,23:0.489:47:13,12	A	missense_	MODERAT	ENSG0000	Transcript	ENST0000(	protein_cc
0/1:22,24:0.522:46:14,13	T	missense_	MODERAT	ENSG0000	Transcript	ENST0000(	protein_cc
0/1:18,21:0.538:39:11,8:	G	missense_	MODERAT	ENSG0000	Transcript	ENST0000(	protein_cc
0/1:33,30:0.476:63:16,14	A	missense_	MODERAT	ENSG0000	Transcript	ENST0000(	protein_cc
0/1:19,21:0.525:40:8,9:1	A	missense_	MODERAT	ENSG0000	Transcript	ENST0000(	protein_cc
0/1:29,28:0.491:57:17,16	G	missense_	MODERAT	ENSG0000	Transcript	ENST0000(	protein_cc
0/1:27,30:0.526:57:13,12	T	missense_	MODERAT	ENSG0000	Transcript	ENST0000(	protein_cc
0/1:23,31:0.574:54:5,12:	A	missense_	MODERAT	ENSG0000	Transcript	ENST0000(	protein_cc
0/1:31,27:0.466:58:16,12	A	missense_	MODERAT	ENSG0000	Transcript	ENST0000(	protein_cc
0/1:12,7:0.368:19:6,0:6,7	T	missense_	MODERAT	ENSG0000	Transcript	ENST0000(	protein_cc
0/1:11,5:0.312:16:9,3:2,2	G	missense_	MODERAT	ENSG0000	Transcript	ENST0000(	protein_cc
0/1:20,18:0.474:38:7,5:1	C	missense_	MODERAT	ENSG0000	Transcript	ENST0000(	protein_cc
0/1:10,14:0.583:24:4,6:6,	A	missense_	MODERAT	ENSG0000	Transcript	ENST0000(	protein_cc
0/1:10,10:0.5:20:3,6:7,4:	T	missense_	MODERAT	ENSG0000	Transcript	ENST0000(	protein_cc
0/1:23,17:0.425:40:10,10	G	missense_	MODERAT	ENSG0000	Transcript	ENST0000(	protein_cc
0/1:20,19:0.487:39:10,10	C	missense_	MODERAT	ENSG0000	Transcript	ENST0000(	protein_cc
0/1:23,22:0.489:45:13,8:	T	missense_	MODERAT	ENSG0000	Transcript	ENST0000(	protein_cc
0/1:10,9:0.474:19:6,7:4,2	C	missense_	MODERAT	ENSG0000	Transcript	ENST0000(	protein_cc
0/1:8,8:0.5:16:4,4:4,4:45	C	missense_	MODERAT	ENSG0000	Transcript	ENST0000(	protein_cc
0/1:10,2:0.167:12:3,0:7,2	T	missense_	MODERAT	ENSG0000	Transcript	ENST0000(	protein_cc
0/1:20,20:0.5:40:7,8:13,1	C	missense_	MODERAT	ENSG0000	Transcript	ENST0000(	protein_cc
0/1:30,26:0.464:56:16,16	T	missense_	MODERAT	ENSG0000	Transcript	ENST0000(	protein_cc
0/1:27,33:0.55:60:15,14:	T	missense_	MODERAT	ENSG0000	Transcript	ENST0000(	protein_cc
0/1:27,25:0.481:52:18,16	T	missense_	MODERAT	ENSG0000	Transcript	ENST0000(	protein_cc
0/1:17,18:0.514:35:8,12:	A	missense_	MODERAT	ENSG0000	Transcript	ENST0000(	protein_cc
0/1:17,29:0.63:46:11,12:	A	missense_	MODERAT	ENSG0000	Transcript	ENST0000(	protein_cc
0|1:13,14:0.519:27:7,6:6	T	missense_	MODERAT	ENSG0000	Transcript	ENST0000(	protein_cc
0/1:15,10:0.4:25:7,4:8,6:	T	missense_	MODERAT	ENSG0000	Transcript	ENST0000(	protein_cc
0/1:13,11:0.458:24:4,4:9,	G	missense_	MODERAT	ENSG0000	Transcript	ENST0000(	protein_cc
0/1:23,15:0.395:38:12,6:	A	missense_	MODERAT	ENSG0000	Transcript	ENST0000(	protein_cc
0/1:14,20:0.588:34:6,11:	C	missense_	MODERAT	ENSG0000	Transcript	ENST0000(	protein_cc
0/1:13,22:0.629:35:6,10:	G	missense_	MODERAT	ENSG0000	Transcript	ENST0000(	protein_cc
0/1:22,19:0.463:41:10,10	A	missense_	MODERAT	ENSG0000	Transcript	ENST0000(	protein_cc
0/1:10,12:0.545:22:6,5:4,	T	missense_	MODERAT	ENSG0000	Transcript	ENST0000(	protein_cc
0/1:20,30:0.6:50:10,13:1	G	missense_	MODERAT	ENSG0000	Transcript	ENST0000(	protein_cc
0/1:13,20:0.606:33:8,13:	G	missense_	MODERAT	ENSG0000	Transcript	ENST0000(	protein_cc
0/1:21,27:0.562:48:13,20	G	missense_	MODERAT	ENSG0000	Transcript	ENST0000(	protein_cc
0/1:15,10:0.4:25:7,4:8,6:	C	missense_	MODERAT	ENSG0000	Transcript	ENST0000(	protein_cc
1/1:0,3:1:3:0,2:0,1:4:49,5	C	missense_	MODERAT	ENSG0000	Transcript	ENST0000(	protein_cc
0/1:10,2:0.167:12:5,0:5,2	C	missense_	MODERAT	ENSG0000	Transcript	ENST0000(	protein_cc
0/1:13,9:0.409:22:9,2:4,7	C	missense_	MODERAT	ENSG0000	Transcript	ENST0000(	protein_cc
0/1:11,7:0.389:18:7,3:4,4	A	missense_	MODERAT	ENSG0000	Transcript	ENST0000(	protein_cc
0/1:14,17:0.548:31:7,10:	G	missense_	MODERAT	ENSG0000	Transcript	ENST0000(	protein_cc
0/1:20,15:0.429:35:7,8:1	C	missense_	MODERAT	ENSG0000	Transcript	ENST0000(	protein_cc

0/1:24,16:0.4:40:11,12:1	T	missense_	MODERAT	ENSG0000	Transcript	ENST0000(	protein_cc
0/1:1,1:0.5:2:0,0:1,1:4:38	G	missense_	MODERAT	ENSG0000	Transcript	ENST0000(	protein_cc
0/1:24,11:0.314:35:13,2:	T	missense_	MODERAT	ENSG0000	Transcript	ENST0000(	protein_cc
0/1:21,19:0.475:40:7,10:	T	missense_	MODERAT	ENSG0000	Transcript	ENST0000(	protein_cc
0/1:19,12:0.387:31:11,5:8	C	missense_	MODERAT	ENSG0000	Transcript	ENST0000(	protein_cc
0/1:21,15:0.417:36:12,10	T	missense_	MODERAT	ENSG0000	Transcript	ENST0000(	protein_cc
0/1:7,7:0.5:14:4,5:3,2:45	T	missense_	MODERAT	ENSG0000	Transcript	ENST0000(	protein_cc
0/1:11,13:0.542:24:7,6:4,	T	missense_	MODERAT	ENSG0000	Transcript	ENST0000(	protein_cc
0/1:8,7:0.467:15:2,6:6,1:4	G	missense_	MODERAT	ENSG0000	Transcript	ENST0000(	protein_cc
0/1:17,10:0.37:27:8,6:9,4	T	missense_	MODERAT	ENSG0000	Transcript	ENST0000(	protein_cc
0/1:22,19:0.463:41:13,12	A	missense_	MODERAT	ENSG0000	Transcript	ENST0000(	protein_cc
0/1:24,12:0.333:36:14,7:	A	missense_	MODERAT	ENSG0000	Transcript	ENST0000(	protein_cc
0/1:25,19:0.432:44:12,11	A	missense_	MODERAT	ENSG0000	Transcript	ENST0000(	protein_cc
0/1:26,35:0.574:61:14,14	G	missense_	MODERAT	ENSG0000	Transcript	ENST0000(	protein_cc
0/1:19,16:0.457:35:8,7:1	A	missense_	MODERAT	ENSG0000	Transcript	ENST0000(	protein_cc
0/1:14,20:0.588:34:9,11:5	G	missense_	MODERAT	ENSG0000	Transcript	ENST0000(	protein_cc
0/1:15,16:0.516:31:7,10:8	A	missense_	MODERAT	ENSG0000	Transcript	ENST0000(	protein_cc
0/1:12,18:0.6:30:5,11:7,7	A	missense_	MODERAT	ENSG0000	Transcript	ENST0000(	protein_cc
0/1:12,11:0.478:23:4,7:8,	A	missense_	MODERAT	ENSG0000	Transcript	ENST0000(	protein_cc
0/1:13,16:0.552:29:7,7:6,	G	missense_	MODERAT	ENSG0000	Transcript	ENST0000(	protein_cc
0/1:20,29:0.592:49:8,12:	T	missense_	MODERAT	ENSG0000	Transcript	ENST0000(	protein_cc
0/1:17,15:0.469:32:11,8:(	C	missense_	MODERAT	ENSG0000	Transcript	ENST0000(	protein_cc
0/1:4,6:0.6:10:2,4:2,2:47	C	missense_	MODERAT	ENSG0000	Transcript	ENST0000(	protein_cc
1/1:0,2:1:2:0,0:0,2:10:58,	C	missense_	MODERAT	ENSG0000	Transcript	ENST0000(	protein_cc
1/1:0,12:1:12:0,6:0,6:30:	C	missense_	MODERAT	ENSG0000	Transcript	ENST0000(	protein_cc
0/1:10,6:0.375:16:2,5:8,1	C	missense_	MODERAT	ENSG0000	Transcript	ENST0000(	protein_cc
0/1:22,19:0.463:41:9,10:	A	missense_	MODERAT	ENSG0000	Transcript	ENST0000(	protein_cc
0/1:10,12:0.545:22:7,8:3,	T	missense_	MODERAT	ENSG0000	Transcript	ENST0000(	protein_cc
0/1:17,16:0.485:33:8,8:9,	T	missense_	MODERAT	ENSG0000	Transcript	ENST0000(	protein_cc
0/1:16,15:0.484:31:3,6:1	A	missense_	MODERAT	ENSG0000	Transcript	ENST0000(	protein_cc
0/1:8,5:0.385:13:2,1:6,4:	G	missense_	MODERAT	ENSG0000	Transcript	ENST0000(	protein_cc
0/1:15,22:0.595:37:7,10:8	T	missense_	MODERAT	ENSG0000	Transcript	ENST0000(	protein_cc
0/1:13,14:0.519:27:3,6:1(	T	missense_	MODERAT	ENSG0000	Transcript	ENST0000(	protein_cc
0|1:17,14:0.452:31:6,8:1	A	missense_	MODERAT	ENSG0000	Transcript	ENST0000(	protein_cc
0/1:13,14:0.519:27:8,4:5,	G	missense_	MODERAT	ENSG0000	Transcript	ENST0000(	protein_cc
0/1:9,3:0.25:12:3,1:6,2:2	A	missense_	MODERAT	ENSG0000	Transcript	ENST0000(	protein_cc
0/1:28,22:0.44:50:13,10:	T	missense_	MODERAT	ENSG0000	Transcript	ENST0000(	protein_cc
0/1:31,28:0.475:59:16,15	A	missense_	MODERAT	ENSG0000	Transcript	ENST0000(	protein_cc
0|1:32,26:0.448:58:18,12	A	missense_	MODERAT	ENSG0000	Transcript	ENST0000(	protein_cc
0/1:21,23:0.523:44:12,13	T	missense_	MODERAT	ENSG0000	Transcript	ENST0000(	protein_cc
1/1:0,9:1:9:0,6:0,3:22:71,	C	missense_	MODERAT	ENSG0000	Transcript	ENST0000(	protein_cc
1/1:0,8:1:8:0,6:0,2:19:69,	A	missense_	MODERAT	ENSG0000	Transcript	ENST0000(	protein_cc
1/1:0,2:1:2:0,2:0,0:3:53,3	A	missense_	MODERAT	ENSG0000	Transcript	ENST0000(	protein_cc
1|1:0,3:1:3:0,2:0,1:4:28,1	G	missense_	MODERAT	ENSG0000	Transcript	ENST0000(	protein_cc
1|1:0,3:1:3:0,2:0,1:4:28,1	C	missense_	MODERAT	ENSG0000	Transcript	ENST0000(	protein_cc
0/1:6,1:0.143:7:5,1:1,0:7	A	missense_	MODERAT	ENSG0000	Transcript	ENST0000(	protein_cc
0/1:15,8:0.348:23:8,4:7,4	A	missense_	MODERAT	ENSG0000	Transcript	ENST0000(	protein_cc
1/1:2,20:0.909:22:1,12:1,	T	missense_	MODERAT	ENSG0000	Transcript	ENST0000(	protein_cc
0/1:11,11:0.5:22:5,6:6,5:(	A	missense_	MODERAT	ENSG0000	Transcript	ENST0000(	protein_cc
1|1:0,2:1:2:0,2:0,0:4:49,5	G	missense_	MODERAT	ENSG0000	Transcript	ENST0000(	protein_cc

0/1:18,26:0.591:44:7,12:	C		missense_	MODERAT	ENSG0000	Transcript	ENST0000	protein_cc
0/1:18,13:0.419:31:9,6:9,	T		missense_	MODERAT	ENSG0000	Transcript	ENST0000	protein_cc
0/1:18,13:0.419:31:11,6:	A		missense_	MODERAT	ENSG0000	Transcript	ENST0000	protein_cc
0/1:15,17:0.531:32:11,12	T		missense_	MODERAT	ENSG0000	Transcript	ENST0000	protein_cc
0/1:15,21:0.583:36:9,14:	T		missense_	MODERAT	ENSG0000	Transcript	ENST0000	protein_cc
0/1:21,17:0.447:38:5,10:	C		missense_	MODERAT	ENSG0000	Transcript	ENST0000	protein_cc
0/1:7,5:0.417:12:3,3:4,2:	C		missense_	MODERAT	ENSG0000	Transcript	ENST0000	protein_cc
0/1:13,18:0.581:31:8,10:	A		missense_	MODERAT	ENSG0000	Transcript	ENST0000	protein_cc
0/1:8,2:0.2:10:4,2:4,0:5:3	T		missense_	MODERAT	ENSG0000	Transcript	ENST0000	protein_cc
0/1:19,19:0.5:38:11,11:8,	T		missense_	MODERAT	ENSG0000	Transcript	ENST0000	protein_cc
0/1:16,29:0.644:45:6,13:	C		missense_	MODERAT	ENSG0000	Transcript	ENST0000	protein_cc
0/1:22,24:0.522:46:11,13	C		missense_	MODERAT	ENSG0000	Transcript	ENST0000	protein_cc
0/1:34,27:0.443:61:14,13	C		missense_	MODERAT	ENSG0000	Transcript	ENST0000	protein_cc
0/1:30,23:0.434:53:13,10	A		missense_	MODERAT	ENSG0000	Transcript	ENST0000	protein_cc
0/1:24,22:0.478:46:11,15	T		missense_	MODERAT	ENSG0000	Transcript	ENST0000	protein_cc
0/1:21,24:0.533:45:9,14:	T		missense_	MODERAT	ENSG0000	Transcript	ENST0000	protein_cc
0/1:21,24:0.533:45:14,9:	C		missense_	MODERAT	ENSG0000	Transcript	ENST0000	protein_cc
0/1:33,24:0.421:57:19,15	C		missense_	MODERAT	ENSG0000	Transcript	ENST0000	protein_cc
0/1:17,20:0.541:37:6,8:1	A		missense_	MODERAT	ENSG0000	Transcript	ENST0000	protein_cc
0/1:25,18:0.419:43:14,11	A		missense_	MODERAT	ENSG0000	Transcript	ENST0000	protein_cc
0/1:17,25:0.595:42:5,14:	A		missense_	MODERAT	ENSG0000	Transcript	ENST0000	protein_cc
0/1:34,15:0.306:49:15,9:	C		missense_	MODERAT	ENSG0000	Transcript	ENST0000	protein_cc
0/1:35,25:0.417:60:18,11	T		missense_	MODERAT	ENSG0000	Transcript	ENST0000	protein_cc
0/1:4,15:0.789:19:1,10:3,	A		missense_	MODERAT	ENSG0000	Transcript	ENST0000	protein_cc
0/1:27,38:0.585:65:14,21	A		missense_	MODERAT	ENSG0000	Transcript	ENST0000	protein_cc
0/1:7,10:0.588:17:5,4:2,6	A		missense_	MODERAT	ENSG0000	Transcript	ENST0000	protein_cc
0/1:15,11:0.423:26:7,4:8,	C		missense_	MODERAT	ENSG0000	Transcript	ENST0000	protein_cc
0/1:8,11:0.579:19:4,7:4,4	G		missense_	MODERAT	ENSG0000	Transcript	ENST0000	protein_cc
0/1:19,11:0.367:30:9,7:1	A		missense_	MODERAT	ENSG0000	Transcript	ENST0000	protein_cc
0/1:3,2:0.4:5:1,2:2,0:11:4	A		missense_	MODERAT	ENSG0000	Transcript	ENST0000	protein_cc
0/1:9,13:0.591:22:3,9:6,4	A		missense_	MODERAT	ENSG0000	Transcript	ENST0000	protein_cc
0/1:11,18:0.621:29:8,8:3,	C		missense_	MODERAT	ENSG0000	Transcript	ENST0000	protein_cc
0/1:22,15:0.405:37:12,6:	A		missense_	MODERAT	ENSG0000	Transcript	ENST0000	protein_cc
0/1:15,14:0.483:29:4,7:1	A		missense_	MODERAT	ENSG0000	Transcript	ENST0000	protein_cc
0/1:4,2:0.333:6:3,1:1,1:1	A		missense_	MODERAT	ENSG0000	Transcript	ENST0000	protein_cc
0/1:26,13:0.333:39:12,7:	T		missense_	MODERAT	ENSG0000	Transcript	ENST0000	protein_cc
0/1:17,13:0.433:30:12,7:	G		missense_	MODERAT	ENSG0000	Transcript	ENST0000	protein_cc
0/1:19,18:0.486:37:8,10:	A		missense_	MODERAT	ENSG0000	Transcript	ENST0000	protein_cc
0/1:24,22:0.478:46:11,12	T		missense_	MODERAT	ENSG0000	Transcript	ENST0000	protein_cc
0/1:17,16:0.485:33:5,7:1	T		missense_	MODERAT	ENSG0000	Transcript	ENST0000	protein_cc
0/1:40,36:0.474:76:19,21	G		missense_	MODERAT	ENSG0000	Transcript	ENST0000	protein_cc
0/1:30,39:0.565:69:14,26	C		missense_	MODERAT	ENSG0000	Transcript	ENST0000	protein_cc
0/1:25,44:0.638:69:11,21	A		missense_	MODERAT	ENSG0000	Transcript	ENST0000	protein_cc
0/1:31,19:0.38:50:16,12:	C		missense_	MODERAT	ENSG0000	Transcript	ENST0000	protein_cc
0/1:36,31:0.463:67:15,15	A		missense_	MODERAT	ENSG0000	Transcript	ENST0000	protein_cc
0/1:22,24:0.522:46:12,9:	T		missense_	MODERAT	ENSG0000	Transcript	ENST0000	protein_cc
0/1:27,27:0.5:54:12,10:1	T		missense_	MODERAT	ENSG0000	Transcript	ENST0000	protein_cc
0/1:16,26:0.619:42:7,17:	A		missense_	MODERAT	ENSG0000	Transcript	ENST0000	protein_cc
0/1:16,7:0.304:23:6,4:10,	T		missense_	MODERAT	ENSG0000	Transcript	ENST0000	protein_cc
0/1:22,25:0.532:47:11,16	G		missense_	MODERAT	ENSG0000	Transcript	ENST0000	protein_cc

0/1:18,19:0.514:37:10,6:	C		missense_	MODERAT	ENSG0000	Transcript	ENST0000	protein_cc
0|1:21,21:0.5:42:11,14:1	T		missense_	MODERAT	ENSG0000	Transcript	ENST0000	protein_cc
0/1:17,15:0.469:32:13,8:	A		missense_	MODERAT	ENSG0000	Transcript	ENST0000	protein_cc
0/1:17,12:0.414:29:7,5:1	T		missense_	MODERAT	ENSG0000	Transcript	ENST0000	protein_cc
0/1:18,8:0.308:26:12,2:6,	C		missense_	MODERAT	ENSG0000	Transcript	ENST0000	protein_cc
1/1:0,6:1:6:0,4:0,2:21:75,	T		missense_	MODERAT	ENSG0000	Transcript	ENST0000	protein_cc
0/1:22,28:0.56:50:11,19:	A		missense_	MODERAT	ENSG0000	Transcript	ENST0000	protein_cc
0/1:28,32:0.533:60:14,14	A		missense_	MODERAT	ENSG0000	Transcript	ENST0000	protein_cc
0/1:12,20:0.625:32:7,8:5,	A		missense_	MODERAT	ENSG0000	Transcript	ENST0000	protein_cc
0/1:10,7:0.412:17:6,6:4,1	C		missense_	MODERAT	ENSG0000	Transcript	ENST0000	protein_cc
0/1:26,18:0.409:44:13,11	T		missense_	MODERAT	ENSG0000	Transcript	ENST0000	protein_cc
0/1:14,17:0.548:31:7,6:7,	T		missense_	MODERAT	ENSG0000	Transcript	ENST0000	protein_cc
0/1:27,25:0.481:52:17,11	A		missense_	MODERAT	ENSG0000	Transcript	ENST0000	protein_cc
0/1:19,35:0.648:54:8,21:	T		missense_	MODERAT	ENSG0000	Transcript	ENST0000	protein_cc
0/1:23,21:0.477:44:14,11	A		missense_	MODERAT	ENSG0000	Transcript	ENST0000	protein_cc
0/1:33,22:0.4:55:17,13:1	T		missense_	MODERAT	ENSG0000	Transcript	ENST0000	protein_cc
0/1:12,8:0.4:20:6,3:6,5:3	T		missense_	MODERAT	ENSG0000	Transcript	ENST0000	protein_cc
0/1:12,16:0.571:28:6,9:6,	T		missense_	MODERAT	ENSG0000	Transcript	ENST0000	protein_cc
0/1:16,20:0.556:36:10,12	C		missense_	MODERAT	ENSG0000	Transcript	ENST0000	protein_cc
0/1:13,12:0.48:25:5,6:8,6	G		missense_	MODERAT	ENSG0000	Transcript	ENST0000	protein_cc
0/1:25,24:0.49:49:11,13:	T		missense_	MODERAT	ENSG0000	Transcript	ENST0000	protein_cc
0/1:30,33:0.524:63:9,17:	A		missense_	MODERAT	ENSG0000	Transcript	ENST0000	protein_cc
0/1:27,21:0.438:48:19,8:	G		missense_	MODERAT	ENSG0000	Transcript	ENST0000	protein_cc
0/1:37,27:0.422:64:13,8:	T		missense_	MODERAT	ENSG0000	Transcript	ENST0000	protein_cc
0/1:34,33:0.493:67:18,19	T		missense_	MODERAT	ENSG0000	Transcript	ENST0000	protein_cc
1/1:0,9:1:9:0,2:0,7:20:77,	A		missense_	MODERAT	ENSG0000	Transcript	ENST0000	protein_cc
0/1:2,7:0.778:9:0,2:2,5:8	T		missense_	MODERAT	ENSG0000	Transcript	ENST0000	protein_cc
1/1:0,8:1:8:0,2:0,6:11:73,	A		missense_	MODERAT	ENSG0000	Transcript	ENST0000	protein_cc
1/1:0,8:1:8:0,2:0,6:12:75,	G		missense_	MODERAT	ENSG0000	Transcript	ENST0000	protein_cc
0/1:31,21:0.404:52:20,15	G		missense_	MODERAT	ENSG0000	Transcript	ENST0000	protein_cc
0/1:29,15:0.341:44:15,6:	A		missense_	MODERAT	ENSG0000	Transcript	ENST0000	protein_cc
0/1:5,5:0.5:10:2,2:3,3:14	C		missense_	MODERAT	ENSG0000	Transcript	ENST0000	protein_cc
0/1:19,32:0.627:51:13,12	A		missense_	MODERAT	ENSG0000	Transcript	ENST0000	protein_cc
0/1:35,32:0.478:67:13,12	T		missense_	MODERAT	ENSG0000	Transcript	ENST0000	protein_cc
0/1:19,34:0.642:53:10,15	A		missense_	MODERAT	ENSG0000	Transcript	ENST0000	protein_cc
0/1:21,18:0.462:39:11,14	A		missense_	MODERAT	ENSG0000	Transcript	ENST0000	protein_cc
0/1:19,28:0.596:47:8,18:	C		missense_	MODERAT	ENSG0000	Transcript	ENST0000	protein_cc
0/1:23,20:0.465:43:11,7:	A		missense_	MODERAT	ENSG0000	Transcript	ENST0000	protein_cc
0/1:23,28:0.549:51:13,15	T		missense_	MODERAT	ENSG0000	Transcript	ENST0000	protein_cc
1/1:0,50:1:50:0,23:0,27:6	G		missense_	MODERAT	ENSG0000	Transcript	ENST0000	protein_cc
0/1:19,22:0.537:41:11,11	A		missense_	MODERAT	ENSG0000	Transcript	ENST0000	protein_cc
0/1:26,10:0.278:36:14,4:	T		missense_	MODERAT	ENSG0000	Transcript	ENST0000	protein_cc
0|1:10,4:0.286:14:6,1:4,3	C		missense_	MODERAT	ENSG0000	Transcript	ENST0000	protein_cc
0/1:7,15:0.682:22:3,9:4,6	C		missense_	MODERAT	ENSG0000	Transcript	ENST0000	protein_cc
0/1:17,17:0.5:34:11,12:6,	A		missense_	MODERAT	ENSG0000	Transcript	ENST0000	protein_cc
0/1:27,26:0.491:53:14,11	T		missense_	MODERAT	ENSG0000	Transcript	ENST0000	protein_cc
0/1:33,27:0.45:60:20,15:	A		missense_	MODERAT	ENSG0000	Transcript	ENST0000	protein_cc
0/1:13,20:0.606:33:7,8:6,	T		missense_	MODERAT	ENSG0000	Transcript	ENST0000	protein_cc
0/1:23,27:0.54:50:13,14:	C		missense_	MODERAT	ENSG0000	Transcript	ENST0000	protein_cc
0/1:15,22:0.595:37:9,15:	C		missense_	MODERAT	ENSG0000	Transcript	ENST0000	protein_cc

0/1:12,15:0.556:27:9,9:3,	G		missense_	MODERAT	ENSG0000	Transcript	ENST0000	protein_cc
0/1:30,20:0.4:50:17,10:1	G		missense_	MODERAT	ENSG0000	Transcript	ENST0000	protein_cc
0/1:21,5:0.192:26:14,3:7,	T		missense_	MODERAT	ENSG0000	Transcript	ENST0000	protein_cc
0/1:19,18:0.486:37:10,11	G		missense_	MODERAT	ENSG0000	Transcript	ENST0000	protein_cc
0/1:30,21:0.412:51:17,11	A		missense_	MODERAT	ENSG0000	Transcript	ENST0000	protein_cc
0/1:28,29:0.509:57:14,10	A		missense_	MODERAT	ENSG0000	Transcript	ENST0000	protein_cc
0/1:10,7:0.412:17:6,2:4,5	T		missense_	MODERAT	ENSG0000	Transcript	ENST0000	protein_cc
0/1:19,21:0.525:40:11,13	A		missense_	MODERAT	ENSG0000	Transcript	ENST0000	protein_cc
0/1:10,24:0.706:34:5,9:5,	A		missense_	MODERAT	ENSG0000	Transcript	ENST0000	protein_cc
0/1:17,20:0.541:37:6,11:	T		missense_	MODERAT	ENSG0000	Transcript	ENST0000	protein_cc
0/1:14,20:0.588:34:7,14:	A		missense_	MODERAT	ENSG0000	Transcript	ENST0000	protein_cc
0/1:11,13:0.542:24:9,7:2,	T		missense_	MODERAT	ENSG0000	Transcript	ENST0000	protein_cc
0/1:15,23:0.605:38:5,15:	A		missense_	MODERAT	ENSG0000	Transcript	ENST0000	protein_cc
0/1:18,19:0.514:37:11,8:	G		missense_	MODERAT	ENSG0000	Transcript	ENST0000	protein_cc
0/1:16,27:0.628:43:6,15:	G		missense_	MODERAT	ENSG0000	Transcript	ENST0000	protein_cc
0/1:16,26:0.619:42:9,11:	A		missense_	MODERAT	ENSG0000	Transcript	ENST0000	protein_cc
0/1:11,8:0.421:19:3,2:8,6	T		missense_	MODERAT	ENSG0000	Transcript	ENST0000	protein_cc
0/1:16,6:0.273:22:2,3:14,	T		missense_	MODERAT	ENSG0000	Transcript	ENST0000	protein_cc
0/1:15,14:0.483:29:7,8:8,	G		missense_	MODERAT	ENSG0000	Transcript	ENST0000	protein_cc
0/1:11,3:0.214:14:7,2:4,1	G		missense_	MODERAT	ENSG0000	Transcript	ENST0000	protein_cc
0/1:3,4:0.571:7:1,2:2,2:3	T		missense_	MODERAT	ENSG0000	Transcript	ENST0000	protein_cc
0/1:15,25:0.625:40:11,12	A		missense_	MODERAT	ENSG0000	Transcript	ENST0000	protein_cc
0|1:1,2:0.667:3:0,1:1,1:6	A		missense_	MODERAT	ENSG0000	Transcript	ENST0000	protein_cc
0|1:1,2:0.667:3:0,1:1,1:6	A		missense_	MODERAT	ENSG0000	Transcript	ENST0000	protein_cc
0/1:1,1:0.5:2:0,1:1,0:4:37	C		missense_	MODERAT	ENSG0000	Transcript	ENST0000	protein_cc
0/1:28,26:0.481:54:15,15	A		missense_	MODERAT	ENSG0000	Transcript	ENST0000	protein_cc
0/1:18,14:0.438:32:9,6:9,	G		missense_	MODERAT	ENSG0000	Transcript	ENST0000	protein_cc
0/1:8,2:0.2:10:2,1:6,1:12	A		missense_	MODERAT	ENSG0000	Transcript	ENST0000	protein_cc
0/1:10,8:0.444:18:6,3:4,5	C		missense_	MODERAT	ENSG0000	Transcript	ENST0000	protein_cc
0/1:5,4:0.444:9:2,1:3,3:3	G		missense_	MODERAT	ENSG0000	Transcript	ENST0000	protein_cc
0/1:10,9:0.474:19:8,5:2,4	T		missense_	MODERAT	ENSG0000	Transcript	ENST0000	protein_cc
0/1:19,18:0.486:37:11,11	T		missense_	MODERAT	ENSG0000	Transcript	ENST0000	protein_cc
0/1:10,11:0.524:21:5,4:5,	C		missense_	MODERAT	ENSG0000	Transcript	ENST0000	protein_cc
0/1:15,14:0.483:29:8,7:7,	T		missense_	MODERAT	ENSG0000	Transcript	ENST0000	protein_cc
0/1:23,22:0.489:45:12,10	A		missense_	MODERAT	ENSG0000	Transcript	ENST0000	protein_cc
0/1:8,11:0.579:19:4,6:4,5	A		missense_	MODERAT	ENSG0000	Transcript	ENST0000	protein_cc
0/1:13,16:0.552:29:8,9:5,	T		missense_	MODERAT	ENSG0000	Transcript	ENST0000	protein_cc
0/1:24,23:0.489:47:15,12	T		missense_	MODERAT	ENSG0000	Transcript	ENST0000	protein_cc
0/1:26,21:0.447:47:15,6:	T		missense_	MODERAT	ENSG0000	Transcript	ENST0000	protein_cc
0/1:28,37:0.569:65:13,18	A		missense_	MODERAT	ENSG0000	Transcript	ENST0000	protein_cc
0/1:28,26:0.481:54:9,18:	G		missense_	MODERAT	ENSG0000	Transcript	ENST0000	protein_cc
0/1:26,24:0.48:50:12,10:	C		missense_	MODERAT	ENSG0000	Transcript	ENST0000	protein_cc
0/1:9,5:0.357:14:5,3:4,2:	T		missense_	MODERAT	ENSG0000	Transcript	ENST0000	protein_cc
0/1:10,17:0.63:27:4,11:6,	G		missense_	MODERAT	ENSG0000	Transcript	ENST0000	protein_cc
0/1:32,22:0.407:54:17,13	A		missense_	MODERAT	ENSG0000	Transcript	ENST0000	protein_cc
0/1:18,18:0.5:36:10,9:8,9	C		missense_	MODERAT	ENSG0000	Transcript	ENST0000	protein_cc
0/1:35,26:0.426:61:19,15	T		missense_	MODERAT	ENSG0000	Transcript	ENST0000	protein_cc
0/1:10,13:0.565:23:7,7:3,	A		missense_	MODERAT	ENSG0000	Transcript	ENST0000	protein_cc
0/1:21,20:0.488:41:12,7:	G		missense_	MODERAT	ENSG0000	Transcript	ENST0000	protein_cc
0/1:19,13:0.406:32:6,4:1	C		missense_	MODERAT	ENSG0000	Transcript	ENST0000	protein_cc

0/1:25,19:0.432:44:8,5:1	A		missense_	MODERAT	ENSG0000	Transcript	ENST0000(	protein_cc
0/1:20,32:0.615:52:13,14	A		missense_	MODERAT	ENSG0000	Transcript	ENST0000(	protein_cc
0|1:11,13:0.542:24:4,6:7	A		missense_	MODERAT	ENSG0000	Transcript	ENST0000(	protein_cc
0/1:9,8:0.471:17:5,3:4,5:	A		missense_	MODERAT	ENSG0000	Transcript	ENST0000(	protein_cc
0/1:15,12:0.444:27:10,9:	G		missense_	MODERAT	ENSG0000	Transcript	ENST0000(	protein_cc
0/1:9,6:0.4:15:3,1:6,5:41	C		missense_	MODERAT	ENSG0000	Transcript	ENST0000(	protein_cc
0/1:17,23:0.575:40:9,9:8,	T		missense_	MODERAT	ENSG0000	Transcript	ENST0000(	protein_cc
0/1:15,12:0.444:27:7,7:8,	G		missense_	MODERAT	ENSG0000	Transcript	ENST0000(	protein_cc
0/1:19,24:0.558:43:9,11:	C		missense_	MODERAT	ENSG0000	Transcript	ENST0000(	protein_cc
0/1:18,17:0.486:35:8,11:	A		missense_	MODERAT	ENSG0000	Transcript	ENST0000(	protein_cc
0/1:24,17:0.415:41:15,7:	C		missense_	MODERAT	ENSG0000	Transcript	ENST0000(	protein_cc
1/1:0,45:1:45:0,23:0,22:5	T		missense_	MODERAT	ENSG0000	Transcript	ENST0000(	protein_cc
1/1:0,47:1:47:0,28:0,19:5	A		missense_	MODERAT	ENSG0000	Transcript	ENST0000(	protein_cc
1/1:0,64:1:64:0,34:0,30:6	C		missense_	MODERAT	ENSG0000	Transcript	ENST0000(	protein_cc
0/1:29,20:0.408:49:13,10	T		missense_	MODERAT	ENSG0000	Transcript	ENST0000(	protein_cc
0/1:17,25:0.595:42:7,16:	C		missense_	MODERAT	ENSG0000	Transcript	ENST0000(	protein_cc
1/1:0,2:1:2:0,1:0,1:4:56,5	T		missense_	MODERAT	ENSG0000	Transcript	ENST0000(	protein_cc
0/1:59,57:0.491:116:34,2	G		missense_	MODERAT	ENSG0000	Transcript	ENST0000(	protein_cc
0/1:27,27:0.5:54:16,16:1	T		missense_	MODERAT	ENSG0000	Transcript	ENST0000(	protein_cc
0/1:27,19:0.413:46:13,13	C		missense_	MODERAT	ENSG0000	Transcript	ENST0000(	protein_cc
0/1:32,25:0.439:57:16,12	A		missense_	MODERAT	ENSG0000	Transcript	ENST0000(	protein_cc
0/1:21,23:0.523:44:14,12	T		missense_	MODERAT	ENSG0000	Transcript	ENST0000(	protein_cc
0/1:15,13:0.464:28:9,6:6,	T		missense_	MODERAT	ENSG0000	Transcript	ENST0000(	protein_cc
0/1:14,21:0.6:35:7,14:7,7	T		missense_	MODERAT	ENSG0000	Transcript	ENST0000(	protein_cc
0/1:17,18:0.514:35:9,8:8,	G		missense_	MODERAT	ENSG0000	Transcript	ENST0000(	protein_cc
0/1:22,17:0.436:39:8,8:1	T		missense_	MODERAT	ENSG0000	Transcript	ENST0000(	protein_cc
0/1:12,26:0.684:38:8,12:	A		missense_	MODERAT	ENSG0000	Transcript	ENST0000(	protein_cc
0/1:15,13:0.464:28:4,7:1	T		missense_	MODERAT	ENSG0000	Transcript	ENST0000(	protein_cc
0/1:26,13:0.333:39:12,6:	A		missense_	MODERAT	ENSG0000	Transcript	ENST0000(	protein_cc
0/1:28,24:0.462:52:15,11	G		missense_	MODERAT	ENSG0000	Transcript	ENST0000(	protein_cc
0|1:22,14:0.389:36:13,9:	A		missense_	MODERAT	ENSG0000	Transcript	ENST0000(	protein_cc
0/1:25,19:0.432:44:12,7:	A		missense_	MODERAT	ENSG0000	Transcript	ENST0000(	protein_cc
0/1:18,21:0.538:39:14,11	A		missense_	MODERAT	ENSG0000	Transcript	ENST0000(	protein_cc
0/1:26,27:0.509:53:15,17	T		missense_	MODERAT	ENSG0000	Transcript	ENST0000(	protein_cc
0/1:23,18:0.439:41:10,9:	C		missense_	MODERAT	ENSG0000	Transcript	ENST0000(	protein_cc
0/1:28,31:0.525:59:9,15:	A		missense_	MODERAT	ENSG0000	Transcript	ENST0000(	protein_cc
0/1:12,20:0.625:32:2,9:1	T		missense_	MODERAT	ENSG0000	Transcript	ENST0000(	protein_cc
0/1:18,26:0.591:44:11,14	T		missense_	MODERAT	ENSG0000	Transcript	ENST0000(	protein_cc
0/1:16,19:0.543:35:8,9:8,	G		missense_	MODERAT	ENSG0000	Transcript	ENST0000(	protein_cc
0/1:17,12:0.414:29:8,7:9,	G		missense_	MODERAT	ENSG0000	Transcript	ENST0000(	protein_cc
0/1:23,28:0.549:51:10,10	T		missense_	MODERAT	ENSG0000	Transcript	ENST0000(	protein_cc
0/1:26,17:0.395:43:12,10	T		missense_	MODERAT	ENSG0000	Transcript	ENST0000(	protein_cc
0/1:29,28:0.491:57:13,14	C		missense_	MODERAT	ENSG0000	Transcript	ENST0000(	protein_cc
0/1:29,28:0.491:57:18,13	T		missense_	MODERAT	ENSG0000	Transcript	ENST0000(	protein_cc
0/1:15,17:0.531:32:9,7:6,	T		missense_	MODERAT	ENSG0000	Transcript	ENST0000(	protein_cc
0/1:28,22:0.44:50:14,16:	A		missense_	MODERAT	ENSG0000	Transcript	ENST0000(	protein_cc
0/1:27,25:0.481:52:17,10	G		missense_	MODERAT	ENSG0000	Transcript	ENST0000(	protein_cc
0/1:10,18:0.643:28:5,12:	C		missense_	MODERAT	ENSG0000	Transcript	ENST0000(	protein_cc
0/1:2,3:0.6:5:1,0:1,3:37:7	G		missense_	MODERAT	ENSG0000	Transcript	ENST0000(	protein_cc
0/1:19,17:0.472:36:9,9:1	T		missense_	MODERAT	ENSG0000	Transcript	ENST0000(	protein_cc

0/1:6,6:0.5:12:3,5:3,1:41	T	missense_	MODERAT	ENSG0000	Transcript	ENST0000(	protein_cc
0/1:7,8:0.533:15:2,3:5,5:	C	missense_	MODERAT	ENSG0000	Transcript	ENST0000(	protein_cc
0/1:7,8:0.533:15:4,3:3,5:	T	missense_	MODERAT	ENSG0000	Transcript	ENST0000(	protein_cc
0/1:8,8:0.5:16:4,0:4,8:47	G	missense_	MODERAT	ENSG0000	Transcript	ENST0000(	protein_cc
0/1:11,15:0.577:26:6,9:5,	A	missense_	MODERAT	ENSG0000	Transcript	ENST0000(	protein_cc
0/1:12,19:0.613:31:2,11:	A	missense_	MODERAT	ENSG0000	Transcript	ENST0000(	protein_cc
0/1:15,18:0.545:33:7,6:8,	G	missense_	MODERAT	ENSG0000	Transcript	ENST0000(	protein_cc
0/1:9,17:0.654:26:4,9:5,8	T	missense_	MODERAT	ENSG0000	Transcript	ENST0000(	protein_cc
0/1:12,13:0.52:25:8,7:4,6	A	missense_	MODERAT	ENSG0000	Transcript	ENST0000(	protein_cc
0|1:18,23:0.561:41:10,11	T	missense_	MODERAT	ENSG0000	Transcript	ENST0000(	protein_cc
0/1:15,14:0.483:29:6,6:9,	C	missense_	MODERAT	ENSG0000	Transcript	ENST0000(	protein_cc
0/1:20,25:0.556:45:10,10	A	missense_	MODERAT	ENSG0000	Transcript	ENST0000(	protein_cc
0/1:22,8:0.267:30:10,4:1	G	missense_	MODERAT	ENSG0000	Transcript	ENST0000(	protein_cc
0/1:10,10:0.5:20:6,5:4,5:	A	missense_	MODERAT	ENSG0000	Transcript	ENST0000(	protein_cc
0/1:3,4:0.571:7:3,2:0,2:4	T	missense_	MODERAT	ENSG0000	Transcript	ENST0000(	protein_cc
1/1:3,11:0.786:14:3,4:0,7	C	missense_	MODERAT	ENSG0000	Transcript	ENST0000(	protein_cc
1/1:3,12:0.8:15:3,4:0,8:4	G	missense_	MODERAT	ENSG0000	Transcript	ENST0000(	protein_cc
0/1:7,7:0.5:14:4,3:3,4:3:4	C	missense_	MODERAT	ENSG0000	Transcript	ENST0000(	protein_cc
1/1:2,12:0.857:14:2,5:0,7	T	missense_	MODERAT	ENSG0000	Transcript	ENST0000(	protein_cc
0/1:19,7:0.269:26:14,5:5,	G	missense_	MODERAT	ENSG0000	Transcript	ENST0000(	protein_cc
0/1:21,27:0.562:48:9,16:	G	missense_	MODERAT	ENSG0000	Transcript	ENST0000(	protein_cc
0/1:33,21:0.389:54:19,12	T	missense_	MODERAT	ENSG0000	Transcript	ENST0000(	protein_cc
0/1:32,29:0.475:61:11,18	A	missense_	MODERAT	ENSG0000	Transcript	ENST0000(	protein_cc
0/1:31,32:0.508:63:11,19	A	missense_	MODERAT	ENSG0000	Transcript	ENST0000(	protein_cc
0/1:23,24:0.511:47:14,10	T	missense_	MODERAT	ENSG0000	Transcript	ENST0000(	protein_cc
0/1:4,5:0.556:9:2,2:2,3:3	T	missense_	MODERAT	ENSG0000	Transcript	ENST0000(	protein_cc
0/1:5,10:0.667:15:3,4:2,6	A	missense_	MODERAT	ENSG0000	Transcript	ENST0000(	protein_cc
0/1:10,7:0.412:17:6,3:4,4	G	missense_	MODERAT	ENSG0000	Transcript	ENST0000(	protein_cc
0/1:10,9:0.474:19:5,4:5,5	G	missense_	MODERAT	ENSG0000	Transcript	ENST0000(	protein_cc
0/1:7,8:0.533:15:3,6:4,2:	G	missense_	MODERAT	ENSG0000	Transcript	ENST0000(	protein_cc
0/1:10,4:0.286:14:2,2:8,2	A	missense_	MODERAT	ENSG0000	Transcript	ENST0000(	protein_cc
0/1:16,17:0.515:33:13,7:	T	missense_	MODERAT	ENSG0000	Transcript	ENST0000(	protein_cc
0/1:17,13:0.433:30:7,5:1	T	missense_	MODERAT	ENSG0000	Transcript	ENST0000(	protein_cc
0/1:18,6:0.25:24:7,4:11,2	G	missense_	MODERAT	ENSG0000	Transcript	ENST0000(	protein_cc
0/1:5,11:0.688:16:2,6:3,5	T	missense_	MODERAT	ENSG0000	Transcript	ENST0000(	protein_cc
0/1:6,10:0.625:16:2,6:4,4	G	missense_	MODERAT	ENSG0000	Transcript	ENST0000(	protein_cc
0/1:18,15:0.455:33:11,5:	A	missense_	MODERAT	ENSG0000	Transcript	ENST0000(	protein_cc
0/1:1,1:0.5:2:1,0:0,1:4:38	T	missense_	MODERAT	ENSG0000	Transcript	ENST0000(	protein_cc
0/1:13,14:0.519:27:6,8:7,	T	missense_	MODERAT	ENSG0000	Transcript	ENST0000(	protein_cc
0/1:12,15:0.556:27:7,10:	G	missense_	MODERAT	ENSG0000	Transcript	ENST0000(	protein_cc
0/1:5,2:0.286:7:5,2:0,0:1	C	missense_	MODERAT	ENSG0000	Transcript	ENST0000(	protein_cc
0/1:22,14:0.389:36:14,7:	T	missense_	MODERAT	ENSG0000	Transcript	ENST0000(	protein_cc
0/1:12,6:0.333:18:7,3:5,3	C	missense_	MODERAT	ENSG0000	Transcript	ENST0000(	protein_cc
0/1:11,9:0.45:20:7,5:4,4:	A	missense_	MODERAT	ENSG0000	Transcript	ENST0000(	protein_cc
0/1:8,12:0.6:20:7,5:1,7:4	A	missense_	MODERAT	ENSG0000	Transcript	ENST0000(	protein_cc
0/1:16,11:0.407:27:9,6:7,	T	missense_	MODERAT	ENSG0000	Transcript	ENST0000(	protein_cc
0/1:9,19:0.679:28:5,11:4,	T	missense_	MODERAT	ENSG0000	Transcript	ENST0000(	protein_cc
0/1:20,10:0.333:30:8,6:1	G	missense_	MODERAT	ENSG0000	Transcript	ENST0000(	protein_cc
0/1:20,19:0.487:39:11,14	T	missense_	MODERAT	ENSG0000	Transcript	ENST0000(	protein_cc
0/1:4,6:0.6:10:4,5:0,1:42	T	missense_	MODERAT	ENSG0000	Transcript	ENST0000(	protein_cc

0/1:10,13:0.565:23:5,10:	C	missense_	MODERAT	ENSG0000	Transcript	ENST0000	protein_cc
0/1:7,9:0.562:16:6,4:1,5:	C	missense_	MODERAT	ENSG0000	Transcript	ENST0000	protein_cc
0/1:7,6:0.462:13:5,4:2,2:	A	missense_	MODERAT	ENSG0000	Transcript	ENST0000	protein_cc
0/1:6,11:0.647:17:3,9:3,2	T	missense_	MODERAT	ENSG0000	Transcript	ENST0000	protein_cc
0/1:7,6:0.462:13:4,3:3,3:	A	missense_	MODERAT	ENSG0000	Transcript	ENST0000	protein_cc
0/1:23,21:0.477:44:11,9:	G	missense_	MODERAT	ENSG0000	Transcript	ENST0000	protein_cc
0/1:34,15:0.306:49:16,9:	T	missense_	MODERAT	ENSG0000	Transcript	ENST0000	protein_cc
0/1:15,19:0.559:34:5,10:	A	missense_	MODERAT	ENSG0000	Transcript	ENST0000	protein_cc
0/1:23,30:0.566:53:11,19	C	missense_	MODERAT	ENSG0000	Transcript	ENST0000	protein_cc
0/1:29,21:0.42:50:20,10:	A	missense_	MODERAT	ENSG0000	Transcript	ENST0000	protein_cc
0/1:26,25:0.49:51:14,16:	A	missense_	MODERAT	ENSG0000	Transcript	ENST0000	protein_cc
0/1:22,24:0.522:46:9,10:	G	missense_	MODERAT	ENSG0000	Transcript	ENST0000	protein_cc
0/1:19,33:0.635:52:7,18:	C	missense_	MODERAT	ENSG0000	Transcript	ENST0000	protein_cc
0/1:8,8:0.5:16:3,5:5,3:45	G	missense_	MODERAT	ENSG0000	Transcript	ENST0000	protein_cc
0/1:11,19:0.633:30:8,8:3,	T	missense_	MODERAT	ENSG0000	Transcript	ENST0000	protein_cc
0/1:12,16:0.571:28:4,7:8,	C	missense_	MODERAT	ENSG0000	Transcript	ENST0000	protein_cc
0/1:23,29:0.558:52:15,15	G	missense_	MODERAT	ENSG0000	Transcript	ENST0000	protein_cc
0/1:27,23:0.46:50:15,8:1	A	missense_	MODERAT	ENSG0000	Transcript	ENST0000	protein_cc
0/1:22,27:0.551:49:11,16	C	missense_	MODERAT	ENSG0000	Transcript	ENST0000	protein_cc
0/1:18,21:0.538:39:11,10	C	missense_	MODERAT	ENSG0000	Transcript	ENST0000	protein_cc
0/1:10,11:0.524:21:5,7:5,	G	missense_	MODERAT	ENSG0000	Transcript	ENST0000	protein_cc
0/1:14,13:0.481:27:6,6:8,	C	missense_	MODERAT	ENSG0000	Transcript	ENST0000	protein_cc
0/1:3,8:0.727:11:3,4:0,4:	C	missense_	MODERAT	ENSG0000	Transcript	ENST0000	protein_cc
0/1:17,19:0.528:36:9,9:8,	A	missense_	MODERAT	ENSG0000	Transcript	ENST0000	protein_cc
0/1:16,5:0.238:21:11,3:5,	A	missense_	MODERAT	ENSG0000	Transcript	ENST0000	protein_cc
0|1:17,15:0.469:32:11,6:	A	missense_	MODERAT	ENSG0000	Transcript	ENST0000	protein_cc
0|1:15,24:0.615:39:7,14:	C	missense_	MODERAT	ENSG0000	Transcript	ENST0000	protein_cc
0|1:14,24:0.632:38:6,14:	A	missense_	MODERAT	ENSG0000	Transcript	ENST0000	protein_cc
0|1:13,22:0.629:35:6,13:	C	missense_	MODERAT	ENSG0000	Transcript	ENST0000	protein_cc
0|1:15,24:0.615:39:6,13:	T	missense_	MODERAT	ENSG0000	Transcript	ENST0000	protein_cc
0|1:15,23:0.605:38:6,12:	G	missense_	MODERAT	ENSG0000	Transcript	ENST0000	protein_cc
0|1:18,23:0.561:41:7,12:	C	missense_	MODERAT	ENSG0000	Transcript	ENST0000	protein_cc
0|1:19,17:0.472:36:5,10:	T	missense_	MODERAT	ENSG0000	Transcript	ENST0000	protein_cc
0|1:19,17:0.472:36:5,10:	G	missense_	MODERAT	ENSG0000	Transcript	ENST0000	protein_cc
0|1:21,16:0.432:37:5,9:1	A	missense_	MODERAT	ENSG0000	Transcript	ENST0000	protein_cc
0|1:21,17:0.447:38:5,10:	C	missense_	MODERAT	ENSG0000	Transcript	ENST0000	protein_cc
0|1:20,16:0.444:36:5,8:1	C	missense_	MODERAT	ENSG0000	Transcript	ENST0000	protein_cc
0/1:17,16:0.485:33:5,7:1	A	missense_	MODERAT	ENSG0000	Transcript	ENST0000	protein_cc
0|1:16,26:0.619:42:6,16:	T	missense_	MODERAT	ENSG0000	Transcript	ENST0000	protein_cc
0/1:25,19:0.432:44:14,11	C	missense_	MODERAT	ENSG0000	Transcript	ENST0000	protein_cc
0/1:23,18:0.439:41:15,11	T	missense_	MODERAT	ENSG0000	Transcript	ENST0000	protein_cc
0|1:36,22:0.379:58:18,9:	T	missense_	MODERAT	ENSG0000	Transcript	ENST0000	protein_cc
0|1:17,18:0.514:35:7,10:	A	missense_	MODERAT	ENSG0000	Transcript	ENST0000	protein_cc
0|1:18,18:0.5:36:8,12:10	G	missense_	MODERAT	ENSG0000	Transcript	ENST0000	protein_cc
0/1:15,10:0.4:25:7,3:8,7:	A	missense_	MODERAT	ENSG0000	Transcript	ENST0000	protein_cc
0/1:33,23:0.411:56:17,8:	A	missense_	MODERAT	ENSG0000	Transcript	ENST0000	protein_cc
0/1:29,28:0.491:57:15,13	G	missense_	MODERAT	ENSG0000	Transcript	ENST0000	protein_cc
0/1:25,20:0.444:45:17,14	A	missense_	MODERAT	ENSG0000	Transcript	ENST0000	protein_cc
0/1:13,19:0.594:32:5,12:	C	missense_	MODERAT	ENSG0000	Transcript	ENST0000	protein_cc
0/1:15,10:0.4:25:3,4:12,6	T	missense_	MODERAT	ENSG0000	Transcript	ENST0000	protein_cc

0/1:9,4:0.308:13:2,1:7,3:	C		missense_	MODERAT	ENSG0000	Transcript	ENST0000(	protein_cc
0/1:7,8:0.533:15:3,4:4,4:	G		missense_	MODERAT	ENSG0000	Transcript	ENST0000(	protein_cc
0/1:15,11:0.423:26:7,4:8,	A		missense_	MODERAT	ENSG0000	Transcript	ENST0000(	protein_cc
0/1:10,13:0.565:23:6,10:	G		missense_	MODERAT	ENSG0000	Transcript	ENST0000(	protein_cc
0/1:13,14:0.519:27:7,9:6,	A		missense_	MODERAT	ENSG0000	Transcript	ENST0000(	protein_cc
0/1:14,11:0.44:25:8,6:6,5	A		missense_	MODERAT	ENSG0000	Transcript	ENST0000(	protein_cc
0/1:12,7:0.368:19:3,5:9,2	C		missense_	MODERAT	ENSG0000	Transcript	ENST0000(	protein_cc
0/1:17,25:0.595:42:5,17:	T		missense_	MODERAT	ENSG0000	Transcript	ENST0000(	protein_cc
0/1:22,26:0.542:48:8,14:	C		missense_	MODERAT	ENSG0000	Transcript	ENST0000(	protein_cc
0/1:15,23:0.605:38:6,11:	T		missense_	MODERAT	ENSG0000	Transcript	ENST0000(	protein_cc
0/1:32,24:0.429:56:14,13	A		missense_	MODERAT	ENSG0000	Transcript	ENST0000(	protein_cc
0/1:16,16:0.5:32:8,8:8,8:	T		missense_	MODERAT	ENSG0000	Transcript	ENST0000(	protein_cc
0/1:36,21:0.368:57:18,14	T		missense_	MODERAT	ENSG0000	Transcript	ENST0000(	protein_cc
0/1:14,17:0.548:31:8,8:6,	G		missense_	MODERAT	ENSG0000	Transcript	ENST0000(	protein_cc
0/1:8,6:0.429:14:6,3:2,3:	A		missense_	MODERAT	ENSG0000	Transcript	ENST0000(	protein_cc
0/1:36,30:0.455:66:15,12	T		missense_	MODERAT	ENSG0000	Transcript	ENST0000(	protein_cc
0/1:10,11:0.524:21:3,6:7,	T		missense_	MODERAT	ENSG0000	Transcript	ENST0000(	protein_cc
0/1:21,24:0.533:45:17,14	T		missense_	MODERAT	ENSG0000	Transcript	ENST0000(	protein_cc
0/1:9,21:0.7:30:3,13:6,8:	T		missense_	MODERAT	ENSG0000	Transcript	ENST0000(	protein_cc
0/1:16,9:0.36:25:6,5:10,4	G		missense_	MODERAT	ENSG0000	Transcript	ENST0000(	protein_cc
0/1:19,19:0.5:38:10,7:9,1	G		missense_	MODERAT	ENSG0000	Transcript	ENST0000(	protein_cc
0/1:15,11:0.423:26:3,7:1	A		missense_	MODERAT	ENSG0000	Transcript	ENST0000(	protein_cc
0/1:24,15:0.385:39:13,8:	A		missense_	MODERAT	ENSG0000	Transcript	ENST0000(	protein_cc
0/1:25,18:0.419:43:12,9:	C		missense_	MODERAT	ENSG0000	Transcript	ENST0000(	protein_cc
0/1:10,7:0.412:17:7,2:3,5	C		missense_	MODERAT	ENSG0000	Transcript	ENST0000(	protein_cc
0/1:19,20:0.513:39:8,13:	A		missense_	MODERAT	ENSG0000	Transcript	ENST0000(	protein_cc
0/1:14,11:0.44:25:9,9:5,2	G		missense_	MODERAT	ENSG0000	Transcript	ENST0000(	protein_cc
0/1:11,14:0.56:25:6,7:5,7	T		missense_	MODERAT	ENSG0000	Transcript	ENST0000(	protein_cc
0/1:28,23:0.451:51:17,13	T		missense_	MODERAT	ENSG0000	Transcript	ENST0000(	protein_cc
0/1:6,10:0.625:16:1,6:5,4	A		missense_	MODERAT	ENSG0000	Transcript	ENST0000(	protein_cc
0/1:36,32:0.471:68:18,15	A		missense_	MODERAT	ENSG0000	Transcript	ENST0000(	protein_cc
0/1:24,21:0.467:45:13,6:	T		missense_	MODERAT	ENSG0000	Transcript	ENST0000(	protein_cc
0/1:30,24:0.444:54:10,10	A		missense_	MODERAT	ENSG0000	Transcript	ENST0000(	protein_cc
0/1:7,13:0.65:20:4,6:3,7:	C		missense_	MODERAT	ENSG0000	Transcript	ENST0000(	protein_cc
0/1:15,13:0.464:28:6,5:9,	A		missense_	MODERAT	ENSG0000	Transcript	ENST0000(	protein_cc
0/1:15,12:0.444:27:6,5:9,	T		missense_	MODERAT	ENSG0000	Transcript	ENST0000(	protein_cc
0/1:18,22:0.55:40:11,10:	A		missense_	MODERAT	ENSG0000	Transcript	ENST0000(	protein_cc
0/1:20,10:0.333:30:11,7:	T		missense_	MODERAT	ENSG0000	Transcript	ENST0000(	protein_cc
0/1:22,27:0.551:49:9,17:	A		missense_	MODERAT	ENSG0000	Transcript	ENST0000(	protein_cc
0/1:26,22:0.458:48:12,11	G		missense_	MODERAT	ENSG0000	Transcript	ENST0000(	protein_cc
0/1:16,15:0.484:31:9,7:7,	A		missense_	MODERAT	ENSG0000	Transcript	ENST0000(	protein_cc
0/1:17,17:0.5:34:6,8:11,9	C		missense_	MODERAT	ENSG0000	Transcript	ENST0000(	protein_cc
0/1:16,22:0.579:38:9,7:7,	A		missense_	MODERAT	ENSG0000	Transcript	ENST0000(	protein_cc
0/1:14,15:0.517:29:7,9:7,	A		missense_	MODERAT	ENSG0000	Transcript	ENST0000(	protein_cc
0/1:20,25:0.556:45:8,17:	C		missense_	MODERAT	ENSG0000	Transcript	ENST0000(	protein_cc
0/1:14,12:0.462:26:11,5:	T		missense_	MODERAT	ENSG0000	Transcript	ENST0000(	protein_cc
0/1:15,14:0.483:29:7,4:8,	C		missense_	MODERAT	ENSG0000	Transcript	ENST0000(	protein_cc
0/1:20,22:0.524:42:15,12	C		missense_	MODERAT	ENSG0000	Transcript	ENST0000(	protein_cc
0/1:10,9:0.474:19:4,1:6,8	G		missense_	MODERAT	ENSG0000	Transcript	ENST0000(	protein_cc
0/1:10,8:0.444:18:2,6:8,2	T		missense_	MODERAT	ENSG0000	Transcript	ENST0000(	protein_cc

0/1:13,15:0.536:28:10,6:	T	missense_	MODERAT	ENSG0000	Transcript	ENST0000(	protein_cc
0/1:29,29:0.5:58:11,18:1	C	missense_	MODERAT	ENSG0000	Transcript	ENST0000(	protein_cc
0/1:37,31:0.456:68:22,15	T	missense_	MODERAT	ENSG0000	Transcript	ENST0000(	protein_cc
0/1:19,29:0.604:48:12,14	G	missense_	MODERAT	ENSG0000	Transcript	ENST0000(	protein_cc
0/1:17,19:0.528:36:8,11:	C	missense_	MODERAT	ENSG0000	Transcript	ENST0000(	protein_cc
0/1:25,19:0.432:44:12,13	T	missense_	MODERAT	ENSG0000	Transcript	ENST0000(	protein_cc
0/1:29,27:0.482:56:14,13	T	missense_	MODERAT	ENSG0000	Transcript	ENST0000(	protein_cc
0/1:20,7:0.259:27:10,4:1	G	missense_	MODERAT	ENSG0000	Transcript	ENST0000(	protein_cc
0/1:23,15:0.395:38:15,8:	T	missense_	MODERAT	ENSG0000	Transcript	ENST0000(	protein_cc
0/1:19,16:0.457:35:11,8:	T	missense_	MODERAT	ENSG0000	Transcript	ENST0000(	protein_cc
0/1:9,12:0.571:21:2,5:7,7	T	missense_	MODERAT	ENSG0000	Transcript	ENST0000(	protein_cc
0/1:16,15:0.484:31:9,10:	T	missense_	MODERAT	ENSG0000	Transcript	ENST0000(	protein_cc
0/1:27,35:0.565:62:11,17	A	missense_	MODERAT	ENSG0000	Transcript	ENST0000(	protein_cc
0/1:12,23:0.657:35:6,8:6,	A	missense_	MODERAT	ENSG0000	Transcript	ENST0000(	protein_cc
0/1:11,5:0.312:16:6,2:5,3	A	missense_	MODERAT	ENSG0000	Transcript	ENST0000(	protein_cc
0/1:14,14:0.5:28:6,3:8,11	G	missense_	MODERAT	ENSG0000	Transcript	ENST0000(	protein_cc
1/1:91.87:1,753:0.999:0,	G	missense_	MODERAT	ENSG0000	Transcript	ENST0000(	protein_cc
1/1:98.13:0,1189:1:0,584	G	missense_	MODERAT	ENSG0000	Transcript	ENST0000(	protein_cc
1:0,12:1:12:0,7:0,5:45:80	T	missense_	MODERAT	ENSG0000	Transcript	ENST0000(	protein_cc
1:0,18:1:18:0,9:0,9:55:90	A	missense_	MODERAT	ENSG0000	Transcript	ENST0000(	protein_cc
1:0,3:1:3:0,1:0,2:7:41,0:7	G	missense_	MODERAT	ENSG0000	Transcript	ENST0000(	protein_cc
1:0,6:1:6:0,1:0,5:38:73,0:	G	missense_	MODERAT	ENSG0000	Transcript	ENST0000(	protein_cc
1:0,12:1:12:0,8:0,4:25:60	A	missense_	MODERAT	ENSG0000	Transcript	ENST0000(	protein_cc
1:0,6:1:6:0,4:0,2:42:77,0:	C	missense_	MODERAT	ENSG0000	Transcript	ENST0000(	protein_cc
1:0,6:1:6:0,5:0,1:19:53,0:	A	missense_	MODERAT	ENSG0000	Transcript	ENST0000(	protein_cc
1:0,6:1:6:0,5:0,1:20:55,0:	T	missense_	MODERAT	ENSG0000	Transcript	ENST0000(	protein_cc
1:0,16:1:16:0,8:0,8:45:80	T	missense_	MODERAT	ENSG0000	Transcript	ENST0000(	protein_cc
1:0,13:1:13:0,8:0,5:47:82	T	missense_	MODERAT	ENSG0000	Transcript	ENST0000(	protein_cc
1:0,8:1:8:0,1:0,7:39:74,0:	T	missense_	MODERAT	ENSG0000	Transcript	ENST0000(	protein_cc
1:0,22:1:22:0,10:0,12:54:	T	missense_	MODERAT	ENSG0000	Transcript	ENST0000(	protein_cc
0/1:5,7:0.583:12:0,3:5,4:	G	missense_	MODERAT	ENSG0000	Transcript	ENST0000(	protein_cc
0/1:15,22:0.595:37:8,11:	T	missense_	MODERAT	ENSG0000	Transcript	ENST0000(	protein_cc
0/1:14,5:0.263:19:5,1:9,4	A	missense_	MODERAT	ENSG0000	Transcript	ENST0000(	protein_cc
0/1:8,14:0.636:22:2,11:6,	T	missense_	MODERAT	ENSG0000	Transcript	ENST0000(	protein_cc
0/1:19,32:0.627:51:7,14:	G	missense_	MODERAT	ENSG0000	Transcript	ENST0000(	protein_cc
0/1:11,18:0.621:29:6,9:5,	T	missense_	MODERAT	ENSG0000	Transcript	ENST0000(	protein_cc
0/1:21,22:0.512:43:7,10:	A	missense_	MODERAT	ENSG0000	Transcript	ENST0000(	protein_cc
0/1:22,27:0.551:49:14,15	T	missense_	MODERAT	ENSG0000	Transcript	ENST0000(	protein_cc
0/1:17,14:0.452:31:9,12:	T	missense_	MODERAT	ENSG0000	Transcript	ENST0000(	protein_cc
0/1:15,26:0.634:41:10,13	A	missense_	MODERAT	ENSG0000	Transcript	ENST0000(	protein_cc
0/1:22,17:0.436:39:11,8:	G	missense_	MODERAT	ENSG0000	Transcript	ENST0000(	protein_cc
0/1:22,11:0.333:33:13,3:	C	missense_	MODERAT	ENSG0000	Transcript	ENST0000(	protein_cc
0/1:11,12:0.522:23:5,4:6,	A	missense_	MODERAT	ENSG0000	Transcript	ENST0000(	protein_cc
0|1:16,12:0.429:28:8,7:8	G	missense_	MODERAT	ENSG0000	Transcript	ENST0000(	protein_cc
0/1:15,13:0.464:28:7,6:8,	T	missense_	MODERAT	ENSG0000	Transcript	ENST0000(	protein_cc
0|1:19,19:0.5:38:6,12:13	T	missense_	MODERAT	ENSG0000	Transcript	ENST0000(	protein_cc
0/1:21,19:0.475:40:13,14	T	missense_	MODERAT	ENSG0000	Transcript	ENST0000(	protein_cc
0/1:27,24:0.471:51:11,7:	T	missense_	MODERAT	ENSG0000	Transcript	ENST0000(	protein_cc
0/1:34,28:0.452:62:13,13	A	splice_acc	HIGH	ENSG0000	Transcript	ENST0000(	protein_co
0/1:3,3:0.5:6:3,2:0,1:7:41	G	splice_acc	HIGH	ENSG0000	Transcript	ENST0000(	protein_co

0/1:20,14:0.412:34:9,6:1	C	splice_acc	HIGH	ENSG0000	Transcript	ENST0000	protein_co
1:0,25:1:25:0,10:0,15:51:	-	splice_acc	HIGH	ENSG0000	Transcript	ENST0000	protein_co
1/1:0,2:1:2:0,1:0,1:9:60,1	T	splice_acc	HIGH	ENSG0000	Transcript	ENST0000	unprocess
0/1:11,16:0.593:27:5,8:6,	-	splice_acc	HIGH	ENSG0000	Transcript	ENST0000	lncRNA
0/1:11,13:0.542:24:3,5:8,	T	splice_don	HIGH	ENSG0000	Transcript	ENST0000	protein_co
0/1:11,10:0.476:21:6,4:5,	A	splice_don	HIGH	ENSG0000	Transcript	ENST0000	protein_co
0/1:11,9:0.45:20:2,5:9,4:	G	splice_don	HIGH	ENSG0000	Transcript	ENST0000	protein_co
0/1:22,17:0.436:39:16,11	G	splice_don	HIGH	ENSG0000	Transcript	ENST0000	protein_co
0/1:21,29:0.58:50:11,16:	A	splice_don	HIGH	ENSG0000	Transcript	ENST0000	transcribe
0/1:8,19:0.704:27:4,9:4,1	A	splice_don	HIGH	ENSG0000	Transcript	ENST0000	lncRNA
0/1:10,4:0.286:14:7,2:3,2	A	stop_gain	HIGH	ENSG0000	Transcript	ENST0000	protein_co
0/1:2,5:0.714:7:2,0:0,5:8	C	stop_gain	HIGH	ENSG0000	Transcript	ENST0000	protein_co
0/1:25,23:0.479:48:13,10	A	stop_gain	HIGH	ENSG0000	Transcript	ENST0000	protein_co
0/1:25,17:0.405:42:13,10	A	stop_gain	HIGH	ENSG0000	Transcript	ENST0000	protein_co
0/1:27,22:0.449:49:16,11	A	stop_gain	HIGH	ENSG0000	Transcript	ENST0000	protein_co
0/1:7,9:0.562:16:5,4:2,5:	T	stop_gain	HIGH	ENSG0000	Transcript	ENST0000	protein_co
0/1:14,18:0.562:32:4,7:1	A	stop_gain	HIGH	ENSG0000	Transcript	ENST0000	protein_co
0/1:16,16:0.5:32:5,8:11,8	G	stop_gain	HIGH	ENSG0000	Transcript	ENST0000	protein_co
0/1:25,27:0.519:52:13,14	A	stop_gain	HIGH	ENSG0000	Transcript	ENST0000	protein_co
0/1:19,17:0.472:36:8,7:1	C	stop_gain	HIGH	ENSG0000	Transcript	ENST0000	protein_co
0/1:15,22:0.595:37:8,7:7,	A	stop_gain	HIGH	ENSG0000	Transcript	ENST0000	protein_co
0/1:16,14:0.467:30:2,6:1	T	stop_gain	HIGH	ENSG0000	Transcript	ENST0000	protein_co
0/1:29,35:0.547:64:18,23	TGATCTGC	stop_gain	HIGH	ENSG0000	Transcript	ENST0000	protein_co

EXON	INTRON	HGVSc	HGVSp	›NA_positi	DS_positio	tein_posit	mino_acid	Codons
5/10		ENST0000(	ENSP0000(	2055-2056	1467-1468	489-490	QQ/QX	caGCag/ca
5/10		ENST0000(	ENSP0000(	2058-2076	1470-1488	490-496	QQQQQQ(	caGCAGCA
32/58		ENST0000(	ENSP0000(	4604	4271	1424	A/X	gCa/ga
4/18		ENST0000(	ENSP0000(	406	381	127	T/X	acC/ac
7/7		ENST0000(	ENSP0000(	1001	936	312	P/X	ccC/cc
1/20		ENST0000(	ENSP0000(	344-345	41-42	14	A/AX	gcg/gcGg
1/20		ENST0000(	ENSP0000(	347-348	44-45	15	R/RX	cgg/cgGCg
4/4		ENST0000(	ENSP0000(	3715	3654	1218	F/X	ttT/tt
1/1		ENST0000(	ENSP0000(	441-442	403-404	135	C/X	TGc/c
1/1		ENST0000(	ENSP0000(	439-440	401-402	134	G/GX	ggc/ggGCc
1/5		ENST0000(	ENSP0000(	202-219	30-47	10-16	GAAHGAG	ggGGCCGC
1/1		ENST0000(	ENSP0000(	390-419	324-353	108-118	ACGSCGGS	gcCTGTGG
22/23		ENST0000(	ENSP0000(	3767-3769	3619-3621	1207	K/-	AAG/-
1/1		ENST0000(	ENSP0000(	433-462	402-431	134-144	CQSSCCKP	tgCCAGTC(
14/18		ENST0000(	ENSP0000(	2182-2184	2173-2175	725	L/-	TTG/-
17/18		ENST0000(	ENSP0000(	2406-2417	2300-2311	767-771	AEDTT/A	gCAGAAG/
1/1		ENST0000(	ENSP0000(	126-149	66-89	22-30	SGYGCGY(	tcTGGTTAT
12/12		ENST0000(	ENSP0000(	1597-1611	1252-1266	418-422	QQQQQ/-	CAGCAGC/
11/22		ENST0000(	ENSP0000(	1205-1207	1102-1104	368	E/-	GAG/-
3/3		ENST0000(	ENSP0000(	9581-9595	9094-9108	3032-3036	LPGNT/-	CTTCCCGG
4/14		ENST0000(	ENSP0000(	881-883	750-752	250-251	DE/E	gaCGAa/ga
7/18		ENST0000(	ENSP0000(	820-825	751-756	251-252	QQ/-	CAGCAG/-
3/4		ENST0000(	ENSP0000(	492-494	359-361	120-121	EG/G	gAGGga/g
4/34		ENST0000(	ENSP0000(	761-769	667-675	223-225	QQQ/-	CAGCAGC/
59/90		ENST0000(	ENSP0000(	7442-7443	7407-7408	2469-2470	-/ED	-/GAAGAC
1/13		ENST0000(	ENSP0000(	703-704	2-3	1	M/MS	atg/atGTC
1/11		ENST0000(	ENSP0000(	638-639	203-204	68	A/AA	gcc/gcGGC
5/5		ENST0000(	ENSP0000(	413-414	348-349	116-117	-/AA	-/GCAGCA
1/1		ENST0000(	ENSP0000(	167-168	146-147	49	P/PSCCAP	ccg/ccCAG
10/11		ENST0000(	ENSP0000(	1673-1674	1602-1603	534-535	-/L	-/CTG
1/5		ENST0000(	ENSP0000(	385-386	369-370	123-124	-/EP	-/GAGCCC
10/11		ENST0000(	ENSP0000(	2032-2033	1659-1660	553-554	-/E	-/GAG
2/12		ENST0000(	ENSP0000(	6231-6232	6140-6141	2047	I/IS	atc/atCAG
11/39		ENST0000(	ENSP0000(	1199	1094	365	R/Q	cGg/cAg
12/22		ENST0000(	ENSP0000(	1968	1631	544	L/H	cTc/cAc
8/15		ENST0000(	ENSP0000(	1108	812	271	T/M	aCg/aTg
4/4		ENST0000(	ENSP0000(	1131	1037	346	L/P	cTc/cCc
6/16		ENST0000(	ENSP0000(	586	560	187	T/M	aCg/aTg
9/9		ENST0000(	ENSP0000(	1815	1471	491	V/M	Gtg/Atg
8/22		ENST0000(	ENSP0000(	1337	1097	366	I/T	aTt/aCt
16/37		ENST0000(	ENSP0000(	2609	1978	660	V/F	Gtt/Ttt
9/10		ENST0000(	ENSP0000(	1234	1195	399	A/T	Gct/Act
8/13		ENST0000(	ENSP0000(	1094	1056	352	S/R	agC/agA
3/6		ENST0000(	ENSP0000(	465	287	96	A/V	gCg/gTg
4/5		ENST0000(	ENSP0000(	780	637	213	M/V	Atg/Gtg
15/23		ENST0000(	ENSP0000(	2592	2449	817	D/N	Gat/Aat
6/6		ENST0000(	ENSP0000(	1048	854	285	G/A	gGt/gCt
10/11		ENST0000(	ENSP0000(	1559	1454	485	R/H	cGc/cAc
9/27		ENST0000(	ENSP0000(	1841	1225	409	G/S	Ggt/Agt

3/9	ENST0000(	ENSP0000(	633	499	167	D/N	Gac/Aac
7/7	ENST0000(	ENSP0000(	1170	1145	382	L/P	cTg/cCg
2/23	ENST0000(	ENSP0000(	181	7	3	P/A	Cca/Gca
8/17	ENST0000(	ENSP0000(	969	853	285	V/I	Gtt/Att
7/21	ENST0000(	ENSP0000(	1794	1154	385	P/L	cCg/cTg
4/21	ENST0000(	ENSP0000(	1307	667	223	S/P	Tct/Cct
5/23	ENST0000(	ENSP0000(	605	584	195	K/R	aAg/aGg
13/34	ENST0000(	ENSP0000(	2330	2074	692	R/C	Cgc/Tgc
6/10	ENST0000(	ENSP0000(	1305	613	205	C/R	Tgc/Cgc
3/4	ENST0000(	ENSP0000(	1090	245	82	V/G	gTt/gGt
1/5	ENST0000(	ENSP0000(	1004	290	97	S/L	tCa/tTa
6/19	ENST0000(	ENSP0000(	1291	1041	347	H/Q	caT/caA
3/3	ENST0000(	ENSP0000(	6140	6064	2022	A/T	Gca/Aca
3/3	ENST0000(	ENSP0000(	2744	2668	890	S/P	Tct/Cct
14/14	ENST0000(	ENSP0000(	2157	2042	681	P/R	cCt/cGt
3/5	ENST0000(	ENSP0000(	321	287	96	V/A	gTg/gCg
39/41	ENST0000(	ENSP0000(	5797	4385	1462	V/A	gTg/gCg
2/2	ENST0000(	ENSP0000(	1062	977	326	S/F	tCt/tTt
13/25	ENST0000(	ENSP0000(	3786	3691	1231	I/L	Att/Ctt
16/35	ENST0000(	ENSP0000(	3072	2809	937	P/A	Cca/Gca
7/7	ENST0000(	ENSP0000(	1416	1348	450	N/D	Aac/Gac
7/12	ENST0000(	ENSP0000(	1062	1025	342	I/T	aTt/aCt
18/28	ENST0000(	ENSP0000(	4445	4213	1405	R/C	Cgt/Tgt
12/24	ENST0000(	ENSP0000(	5385	5254	1752	A/P	Gct/Cct
4/8	ENST0000(	ENSP0000(	947	755	252	C/F	tGc/tTc
6/16	ENST0000(	ENSP0000(	646	497	166	T/M	aCg/aTg
2/12	ENST0000(	ENSP0000(	238	15	5	K/N	aaA/aaC
27/32	ENST0000(	ENSP0000(	3875	3758	1253	E/A	gAa/gCa
2/4	ENST0000(	ENSP0000(	277	232	78	V/L	Gtg/Ctg
38/116	ENST0000(	ENSP0000(	10275	10115	3372	A/V	gCg/gTg
81/116	ENST0000(	ENSP0000(	20421	20261	6754	S/N	aGc/aAc
6/12	ENST0000(	ENSP0000(	1116	829	277	S/G	Agc/Ggc
7/7	ENST0000(	ENSP0000(	2783	1603	535	I/L	Atc/Ctc
3/6	ENST0000(	ENSP0000(	426	403	135	I/V	Ata/Gta
26/26	ENST0000(	ENSP0000(	5813	5107	1703	L/F	Ctt/Ttt
13/19	ENST0000(	ENSP0000(	1523	1349	450	P/L	cCa/cTa
5/38	ENST0000(	ENSP0000(	903	151	51	P/T	Ccc/Acc
7/14	ENST0000(	ENSP0000(	1244	1001	334	P/L	cCg/cTg
15/36	ENST0000(	ENSP0000(	2021	1831	611	T/A	Acc/Gcc
2/2	ENST0000(	ENSP0000(	383	166	56	K/E	Aag/Gag
2/2	ENST0000(	ENSP0000(	870	688	230	C/R	Tgt/Cgt
11/31	ENST0000(	ENSP0000(	1011	959	320	R/Q	cGg/cAg
1/6	ENST0000(	ENSP0000(	365	82	28	T/A	Acc/Gcc
3/10	ENST0000(	ENSP0000(	939	835	279	S/G	Agt/Ggt
2/2	ENST0000(	ENSP0000(	427	196	66	Q/E	Caa/Gaa
6/6	ENST0000(	ENSP0000(	1689	1223	408	M/T	aTg/aCg
8/33	ENST0000(	ENSP0000(	3420	2728	910	V/I	Gta/Ata
3/9	ENST0000(	ENSP0000(	461	370	124	R/W	Cgg/Tgg
6/6	ENST0000(	ENSP0000(	3048	2140	714	S/P	Tct/Cct
19/26	ENST0000(	ENSP0000(	2523	2398	800	R/G	Cgg/Ggg

6/9	ENST0000(	ENSP0000(	480	421	141	A/T	Gct/Act
6/38	ENST0000(	ENSP0000(	1002	884	295	E/G	gAa/gGa
20/20	ENST0000(	ENSP0000(	3363	3150	1050	E/D	gaG/gaC
15/16	ENST0000(	ENSP0000(	1579	1318	440	V/I	Gtc/Atc
5/5	ENST0000(	ENSP0000(	546	478	160	G/R	Gga/Aga
3/12	ENST0000(	ENSP0000(	694	608	203	S/C	tCc/tGc
3/14	ENST0000(	ENSP0000(	357	241	81	L/M	Ctg/Atg
14/58	ENST0000(	ENSP0000(	1761	1675	559	V/M	Gtg/Atg
3/3	ENST0000(	ENSP0000(	811	751	251	M/V	Atg/Gtg
8/13	ENST0000(	ENSP0000(	1771	1687	563	P/T	Ccg/Acg
11/14	ENST0000(	ENSP0000(	1347	1325	442	P/L	cCc/cTc
1/14	ENST0000(	ENSP0000(	29	7	3	W/R	Tgg/Cgg
9/18	ENST0000(	ENSP0000(	1147	1003	335	A/S	Gcc/Tcc
5/5	ENST0000(	ENSP0000(	395	364	122	A/T	Gct/Act
5/6	ENST0000(	ENSP0000(	515	484	162	R/S	Cgt/Agt
2/7	ENST0000(	ENSP0000(	289	170	57	R/H	cGc/cAc
2/2	ENST0000(	ENSP0000(	895	644	215	L/S	tTg/tCg
9/12	ENST0000(	ENSP0000(	2344	2060	687	N/S	aAt/aGt
2/2	ENST0000(	ENSP0000(	429	391	131	L/F	Ctc/Ttc
4/4	ENST0000(	ENSP0000(	2330	349	117	A/T	Gca/Aca
1/28	ENST0000(	ENSP0000(	237	23	8	Q/R	cAa/cGa
1/1	ENST0000(	ENSP0000(	959	391	131	I/V	Att/Gtt
20/23	ENST0000(	ENSP0000(	3291	3143	1048	R/Q	cGa/cAa
1/1	ENST0000(	ENSP0000(	851	851	284	V/A	gTc/gCc
1/1	ENST0000(	ENSP0000(	161	161	54	G/A	gGc/gCc
3/3	ENST0000(	ENSP0000(	928	742	248	V/I	Gtc/Atc
1/14	ENST0000(	ENSP0000(	678	377	126	D/G	gAt/gGt
2/16	ENST0000(	ENSP0000(	364	191	64	T/M	aCg/aTg
1/1	ENST0000(	ENSP0000(	1473	1456	486	A/T	Gca/Aca
7/12	ENST0000(	ENSP0000(	828	659	220	R/H	cGc/cAc
12/17	ENST0000(	ENSP0000(	1164	1139	380	R/Q	cGg/cAg
3/3	ENST0000(	ENSP0000(	493	347	116	S/L	tCg/tTg
7/14	ENST0000(	ENSP0000(	1206	931	311	R/W	Cgg/Tgg
28/34	ENST0000(	ENSP0000(	5358	4516	1506	V/I	Gtt/Att
2/4	ENST0000(	ENSP0000(	455	339	113	R/S	agG/agC
2/8	ENST0000(	ENSP0000(	471	365	122	R/H	cGc/cAc
7/9	ENST0000(	ENSP0000(	1192	994	332	F/V	Ttt/Gtt
2/2	ENST0000(	ENSP0000(	553	503	168	Y/C	tAc/tGc
3/28	ENST0000(	ENSP0000(	738	592	198	V/M	Gtg/Atg
3/3	ENST0000(	ENSP0000(	256	212	71	Y/F	tAt/tTt
8/11	ENST0000(	ENSP0000(	750	533	178	K/R	aAa/aGa
3/3	ENST0000(	ENSP0000(	604	460	154	P/A	Cca/Gca
2/6	ENST0000(	ENSP0000(	317	283	95	A/P	Gca/Cca
1/6	ENST0000(	ENSP0000(	173	23	8	Q/P	cAa/cCa
3/4	ENST0000(	ENSP0000(	406	368	123	P/R	cCa/cGa
4/5	ENST0000(	ENSP0000(	388	350	117	K/R	aAa/aGa
16/33	ENST0000(	ENSP0000(	2287	2149	717	Y/H	Tat/Cat
17/17	ENST0000(	ENSP0000(	2796	2560	854	V/M	Gtg/Atg
7/10	ENST0000(	ENSP0000(	1854	1666	556	S/R	Agt/Cgt
1/9	ENST0000(	ENSP0000(	548	494	165	V/A	gTg/gCg

8/13	ENST0000(	ENSP0000(	2988	2798	933	R/H	cGc/cAc
2/9	ENST0000(	ENSP0000(	485	416	139	Q/P	cAg/cCg
6/9	ENST0000(	ENSP0000(	1254	1186	396	D/N	Gac/Aac
2/28	ENST0000(	ENSP0000(	225	89	30	P/L	cCt/cTt
12/22	ENST0000(	ENSP0000(	1471	1216	406	L/V	Ctg/Gtg
29/33	ENST0000(	ENSP0000(	3525	3344	1115	R/H	cGc/cAc
23/27	ENST0000(	ENSP0000(	4739	4658	1553	T/I	aCt/aTt
20/21	ENST0000(	ENSP0000(	4354	4127	1376	S/N	aGt/aAt
6/21	ENST0000(	ENSP0000(	3002	2775	925	L/F	ttG/ttC
2/21	ENST0000(	ENSP0000(	759	532	178	V/I	Gtt/Att
13/16	ENST0000(	ENSP0000(	2852	2795	932	R/Q	cGg/cAg
9/13	ENST0000(	ENSP0000(	1301	959	320	R/Q	cGg/cAg
63/79	ENST0000(	ENSP0000(	10782	10746	3582	D/E	gaC/gaA
6/27	ENST0000(	ENSP0000(	1014	905	302	G/A	gGt/gCt
6/9	ENST0000(	ENSP0000(	1842	1609	537	L/I	Ctc/Atc
8/8	ENST0000(	ENSP0000(	1749	1471	491	S/P	Tct/Cct
20/23	ENST0000(	ENSP0000(	3109	2773	925	Q/K	Cag/Aag
55/61	ENST0000(	ENSP0000(	8076	8054	2685	R/H	cGc/cAc
22/27	ENST0000(	ENSP0000(	9050	8851	2951	A/T	Gcc/Acc
9/20	ENST0000(	ENSP0000(	1173	911	304	E/G	gAa/gGa
1/4	ENST0000(	ENSP0000(	23	5	2	A/V	gCg/gTg
40/83	ENST0000(	ENSP0000(	6378	6059	2020	Y/C	tAt/tGt
12/12	ENST0000(	ENSP0000(	1521	1404	468	E/D	gaG/gaC
1/1	ENST0000(	ENSP0000(	202	202	68	W/R	Tgg/Cgg
5/11	ENST0000(	ENSP0000(	975	923	308	V/A	gTt/gCt
1/1	ENST0000(	ENSP0000(	869	869	290	N/S	aAt/aGt
2/2	ENST0000(	ENSP0000(	523	307	103	V/I	Gta/Ata
2/2	ENST0000(	ENSP0000(	135	62	21	G/D	gGc/gAc
5/16	ENST0000(	ENSP0000(	1328	290	97	R/Q	cGg/cAg
3/6	ENST0000(	ENSP0000(	2429	546	182	M/I	atG/atA
9/18	ENST0000(	ENSP0000(	988	731	244	N/T	aAt/aCt
3/3	ENST0000(	ENSP0000(	552	367	123	V/M	Gtg/Atg
5/6	ENST0000(	ENSP0000(	1132	819	273	E/D	gaA/gaT
8/12	ENST0000(	ENSP0000(	2150	1808	603	T/M	aCg/aTg
10/14	ENST0000(	ENSP0000(	1969	1932	644	L/F	ttG/ttC
12/42	ENST0000(	ENSP0000(	2149	1784	595	S/F	tCc/tTc
7/7	ENST0000(	ENSP0000(	1428	1301	434	R/K	aGg/aAg
4/4	ENST0000(	ENSP0000(	3702	3340	1114	R/W	Cgg/Tgg
4/4	ENST0000(	ENSP0000(	453	278	93	G/D	gGc/gAc
7/7	ENST0000(	ENSP0000(	15249	15112	5038	V/I	Gtc/Atc
7/7	ENST0000(	ENSP0000(	6564	6427	2143	N/D	Aac/Gac
7/7	ENST0000(	ENSP0000(	6555	6418	2140	T/S	Acc/Tcc
7/7	ENST0000(	ENSP0000(	6259	6122	2041	P/L	cCt/cTt
7/7	ENST0000(	ENSP0000(	4200	4063	1355	A/P	Gca/Cca
7/7	ENST0000(	ENSP0000(	4194	4057	1353	L/V	Ctg/Gtg
7/7	ENST0000(	ENSP0000(	4144	4007	1336	P/L	cCg/cTg
2/19	ENST0000(	ENSP0000(	258	160	54	P/S	Cct/Tct
19/19	ENST0000(	ENSP0000(	1911	1813	605	H/N	Cac/Aac
18/19	ENST0000(	ENSP0000(	1675	1577	526	A/V	gCt/gTt
16/19	ENST0000(	ENSP0000(	1527	1429	477	C/R	Tgc/Cgc

4/21	ENST0000(	ENSP0000(	1602	1117	373	L/V	Ctc/Gtc
2/12	ENST0000(	ENSP0000(	414	82	28	E/K	Gag/Aag
2/13	ENST0000(	ENSP0000(	269	238	80	A/T	Gcg/Acg
13/15	ENST0000(	ENSP0000(	1454	1281	427	D/E	gaT/gaA
9/19	ENST0000(	ENSP0000(	2498	2420	807	A/V	gCg/gTg
4/10	ENST0000(	ENSP0000(	383	140	47	N/S	aAt/aGt
28/85	ENST0000(	ENSP0000(	2870	2797	933	T/A	Aca/Gca
10/12	ENST0000(	ENSP0000(	947	931	311	R/W	Cgg/Tgg
11/18	ENST0000(	ENSP0000(	1317	1052	351	A/V	gCg/gTg
2/4	ENST0000(	ENSP0000(	2185	1930	644	I/L	Ata/Tta
1/14	ENST0000(	ENSP0000(	176	79	27	G/R	Ggc/Cgc
1/14	ENST0000(	ENSP0000(	392	143	48	I/T	aTc/aCc
1/2	ENST0000(	ENSP0000(	430	161	54	S/T	aGc/aCc
11/41	ENST0000(	ENSP0000(	1229	1177	393	V/I	Gtc/Atc
4/21	ENST0000(	ENSP0000(	904	622	208	D/Y	Gat/Tat
7/7	ENST0000(	ENSP0000(	834	752	251	R/K	aGa/aAa
8/10	ENST0000(	ENSP0000(	1071	1013	338	A/G	gCt/gGt
4/4	ENST0000(	ENSP0000(	715	689	230	V/A	gTt/gCt
3/10	ENST0000(	ENSP0000(	1062	1016	339	S/F	tCt/tTt
2/10	ENST0000(	ENSP0000(	846	800	267	T/I	aCa/aTa
5/6	ENST0000(	ENSP0000(	478	302	101	T/M	aCg/aTg
1/7	ENST0000(	ENSP0000(	164	107	36	Y/C	tAt/tGt
1/2	ENST0000(	ENSP0000(	274	164	55	R/H	cGc/cAc
11/31	ENST0000(	ENSP0000(	1228	1097	366	R/H	cGc/cAc
16/41	ENST0000(	ENSP0000(	2018	1913	638	S/L	tCg/tTg
10/12	ENST0000(	ENSP0000(	1492	1243	415	P/S	Ccc/Tcc
3/12	ENST0000(	ENSP0000(	568	319	107	K/E	Aag/Gag
19/62	ENST0000(	ENSP0000(	2897	2586	862	R/S	agG/agC
6/25	ENST0000(	ENSP0000(	2872	2681	894	S/N	aGt/aAt
4/8	ENST0000(	ENSP0000(	1161	380	127	R/H	cGc/cAc
4/4	ENST0000(	ENSP0000(	950	790	264	E/K	Gaa/Aaa
2/7	ENST0000(	ENSP0000(	1380	1172	391	F/C	tTc/tGc
2/3	ENST0000(	ENSP0000(	1936	1907	636	T/M	aCg/aTg
6/16	ENST0000(	ENSP0000(	602	367	123	A/T	Gct/Act
83/86	ENST0000(	ENSP0000(	14598	14414	4805	T/M	aCg/aTg
46/86	ENST0000(	ENSP0000(	7458	7274	2425	G/D	gGc/gAc
1/30	ENST0000(	ENSP0000(	695	271	91	K/Q	Aag/Cag
3/27	ENST0000(	ENSP0000(	565	341	114	R/Q	cGg/cAg
6/7	ENST0000(	ENSP0000(	1746	1321	441	V/I	Gtc/Atc
4/6	ENST0000(	ENSP0000(	668	502	168	D/N	Gac/Aac
12/15	ENST0000(	ENSP0000(	1277	1249	417	M/V	Atg/Gtg
12/15	ENST0000(	ENSP0000(	1361	1333	445	G/R	Ggc/Cgc
6/51	ENST0000(	ENSP0000(	849	593	198	A/V	gCg/gTg
3/3	ENST0000(	ENSP0000(	1085	1006	336	I/V	Att/Gtt
10/26	ENST0000(	ENSP0000(	1599	1496	499	A/V	gCg/gTg
15/31	ENST0000(	ENSP0000(	1755	1711	571	G/R	Ggg/Agg
2/12	ENST0000(	ENSP0000(	511	271	91	V/I	Gtc/Atc
11/12	ENST0000(	ENSP0000(	1274	1208	403	R/H	cGt/cAt
6/8	ENST0000(	ENSP0000(	774	676	226	E/K	Gag/Aag
2/11	ENST0000(	ENSP0000(	272	55	19	R/G	Cga/Gga

17/23	ENST0000(	ENSP0000(	2783	2763	921	N/K	aaC/aaG
1/2	ENST0000(	ENSP0000(	952	152	51	G/D	gGc/gAc
4/24	ENST0000(	ENSP0000(	1476	1397	466	T/I	aCc/aTc
12/35	ENST0000(	ENSP0000(	1454	1349	450	R/Q	cGa/cAa
1/1	ENST0000(	ENSP0000(	285	227	76	K/R	aAg/aGg
1/1	ENST0000(	ENSP0000(	68	68	23	T/I	aCt/aTt
7/13	ENST0000(	ENSP0000(	1272	841	281	R/W	Cgg/Tgg
4/13	ENST0000(	ENSP0000(	508	283	95	A/T	Gcc/Acc
7/19	ENST0000(	ENSP0000(	922	799	267	S/C	Agt/Tgt
6/6	ENST0000(	ENSP0000(	2279	1679	560	H/R	cAc/cGc
15/18	ENST0000(	ENSP0000(	1413	1388	463	R/Q	cGg/cAg
12/19	ENST0000(	ENSP0000(	944	773	258	S/L	tCa/tTa
2/13	ENST0000(	ENSP0000(	350	193	65	L/F	Ctc/Ttc
48/64	ENST0000(	ENSP0000(	7793	7535	2512	P/L	cCg/cTg
2/3	ENST0000(	ENSP0000(	623	322	108	P/S	Ccc/Tcc
5/5	ENST0000(	ENSP0000(	1618	1463	488	A/V	gCg/gTg
4/12	ENST0000(	ENSP0000(	1876	970	324	E/K	Gaa/Aaa
24/28	ENST0000(	ENSP0000(	2583	2411	804	S/N	aGc/aAc
12/12	ENST0000(	ENSP0000(	3502	3089	1030	V/A	gTc/gCc
15/17	ENST0000(	ENSP0000(	3799	3656	1219	Y/C	tAc/tGc
8/31	ENST0000(	ENSP0000(	1088	1037	346	S/L	tCg/tTg
8/15	ENST0000(	ENSP0000(	1032	796	266	P/T	Cct/Act
15/15	ENST0000(	ENSP0000(	1672	1646	549	Q/R	cAg/cGg
11/19	ENST0000(	ENSP0000(	1693	1672	558	A/T	Gcg/Acg
13/15	ENST0000(	ENSP0000(	2656	2533	845	V/L	Gtg/Ttg
5/8	ENST0000(	ENSP0000(	1201	1082	361	T/I	aCt/aTt
5/8	ENST0000(	ENSP0000(	1146	1027	343	G/S	Ggt/Agt
5/8	ENST0000(	ENSP0000(	1003	884	295	T/I	aCt/aTt
5/8	ENST0000(	ENSP0000(	997	878	293	S/T	aGt/aCt
14/18	ENST0000(	ENSP0000(	2037	2023	675	V/L	Gtg/Ctg
5/18	ENST0000(	ENSP0000(	583	467	156	G/V	gGg/gTg
4/7	ENST0000(	ENSP0000(	652	628	210	E/Q	Gag/Cag
12/12	ENST0000(	ENSP0000(	1283	1250	417	R/Q	cGg/cAg
8/10	ENST0000(	ENSP0000(	883	724	242	G/R	Ggg/Agg
5/5	ENST0000(	ENSP0000(	867	814	272	R/C	Cgc/Tgc
1/24	ENST0000(	ENSP0000(	33	8	3	A/V	gCg/gTg
12/42	ENST0000(	ENSP0000(	1421	1253	418	H/R	cAc/cGc
5/8	ENST0000(	ENSP0000(	691	602	201	R/H	cGt/cAt
17/19	ENST0000(	ENSP0000(	2051	2015	672	R/Q	cGa/cAa
4/10	ENST0000(	ENSP0000(	352	313	105	D/H	Gac/Cac
4/14	ENST0000(	ENSP0000(	477	464	155	A/V	gCg/gTg
4/4	ENST0000(	ENSP0000(	1017	892	298	H/Y	Cac/Tac
3/3	ENST0000(	ENSP0000(	976	658	220	T/A	Act/Gct
4/4	ENST0000(	ENSP0000(	1495	1406	469	N/T	aAt/aCt
20/29	ENST0000(	ENSP0000(	3413	2746	916	A/S	Gcc/Tcc
5/5	ENST0000(	ENSP0000(	887	839	280	G/E	gGa/gAa
13/21	ENST0000(	ENSP0000(	1282	1189	397	R/S	Cgc/Agc
4/4	ENST0000(	ENSP0000(	851	733	245	A/T	Gca/Aca
4/8	ENST0000(	ENSP0000(	1291	662	221	P/R	cCc/cGc
3/3	ENST0000(	ENSP0000(	799	437	146	P/R	cCc/cGc

2/3	ENST0000(	ENSP0000(	288	82	28	R/G	Cgc/Ggc
3/3	ENST0000(	ENSP0000(	588	404	135	D/A	gAc/gCc
8/12	ENST0000(	ENSP0000(	1584	1376	459	R/H	cGc/cAc
4/4	ENST0000(	ENSP0000(	1840	1723	575	I/V	Ata/Gta
11/14	ENST0000(	ENSP0000(	2717	2563	855	A/T	Gct/Act
19/23	ENST0000(	ENSP0000(	3910	3850	1284	V/L	Gtg/Ttg
3/3	ENST0000(	ENSP0000(	3387	3362	1121	R/Q	cGa/cAa
9/44	ENST0000(	ENSP0000(	1322	1139	380	P/H	cCc/cAc
74/74	ENST0000(	ENSP0000(	14637	14498	4833	G/D	gGt/gAt
15/16	ENST0000(	ENSP0000(	1777	1492	498	R/C	Cgc/Tgc
1/1	ENST0000(	ENSP0000(	535	428	143	P/L	cCc/cTc
23/38	ENST0000(	ENSP0000(	2393	2351	784	T/K	aCa/aAa
2/5	ENST0000(	ENSP0000(	235	143	48	R/H	cGt/cAt
80/80	ENST0000(	ENSP0000(	11005	10610	3537	I/T	aTa/aCa
6/6	ENST0000(	ENSP0000(	1401	1373	458	N/S	aAt/aGt
11/12	ENST0000(	ENSP0000(	1171	1094	365	P/L	cCg/cTg
11/17	ENST0000(	ENSP0000(	1718	1658	553	I/N	aTc/aAc
2/77	ENST0000(	ENSP0000(	257	163	55	H/Y	Cac/Tac
7/9	ENST0000(	ENSP0000(	1087	964	322	M/V	Atg/Gtg
4/7	ENST0000(	ENSP0000(	876	723	241	D/E	gaT/gaG
68/88	ENST0000(	ENSP0000(	4074	3958	1320	D/N	Gat/Aat
7/8	ENST0000(	ENSP0000(	774	658	220	V/I	Gta/Ata
15/15	ENST0000(	ENSP0000(	2047	1954	652	R/W	Cgg/Tgg
15/15	ENST0000(	ENSP0000(	2042	1949	650	T/M	aCg/aTg
15/15	ENST0000(	ENSP0000(	2018	1925	642	K/R	aAa/aGa
2/2	ENST0000(	ENSP0000(	1370	1132	378	A/T	Gcg/Acg
11/24	ENST0000(	ENSP0000(	929	913	305	I/V	Att/Gtt
131/182	ENST0000(	ENSP0000(	20270	20078	6693	T/I	aCc/aTc
5/10	ENST0000(	ENSP0000(	2267	1889	630	V/A	gTg/gCg
26/29	ENST0000(	ENSP0000(	3643	3572	1191	I/T	aTt/aCt
5/10	ENST0000(	ENSP0000(	646	469	157	P/S	Ccc/Tcc
1/1	ENST0000(	ENSP0000(	751	710	237	R/H	cGc/cAc
326/363	ENST0000(	ENSP0000(	84161	83936	27979	Q/R	cAa/cGa
14/23	ENST0000(	ENSP0000(	1544	1453	485	P/S	Cct/Tct
3/6	ENST0000(	ENSP0000(	350	265	89	G/R	Gga/Aga
9/12	ENST0000(	ENSP0000(	1031	914	305	A/V	gCa/gTa
3/3	ENST0000(	ENSP0000(	401	350	117	R/H	cGc/cAc
7/8	ENST0000(	ENSP0000(	1097	987	329	M/I	atG/atT
24/25	ENST0000(	ENSP0000(	3084	2991	997	E/D	gaG/gaT
11/32	ENST0000(	ENSP0000(	1791	1613	538	R/Q	cGg/cAg
7/7	ENST0000(	ENSP0000(	1713	1010	337	Y/C	tAt/tGt
2/2	ENST0000(	ENSP0000(	2042	1712	571	D/G	gAt/gGt
17/26	ENST0000(	ENSP0000(	1921	1748	583	R/H	cGt/cAt
2/3	ENST0000(	ENSP0000(	305	236	79	G/A	gGg/gCg
9/9	ENST0000(	ENSP0000(	1674	1625	542	R/Q	cGg/cAg
6/9	ENST0000(	ENSP0000(	1264	1245	415	L/F	ttG/ttC
8/15	ENST0000(	ENSP0000(	1319	1235	412	P/L	cCc/cTc
2/3	ENST0000(	ENSP0000(	1267	1250	417	R/H	cGt/cAt
2/2	ENST0000(	ENSP0000(	877	782	261	T/S	aCc/aGc
14/14	ENST0000(	ENSP0000(	3498	3413	1138	V/G	gTa/gGa

2/7	ENST0000(	ENSP0000(	111	76	26	W/R	Tgg/Agg
10/14	ENST0000(	ENSP0000(	1406	1243	415	R/W	Cgg/Tgg
3/5	ENST0000(	ENSP0000(	357	327	109	N/K	aaC/aaA
4/4	ENST0000(	ENSP0000(	2022	1229	410	T/I	aCa/aTa
4/18	ENST0000(	ENSP0000(	446	295	99	R/G	Aga/Gga
1/1	ENST0000(	ENSP0000(	665	493	165	L/V	Ctc/Gtc
19/39	ENST0000(	ENSP0000(	2608	2506	836	A/T	Gca/Aca
2/7	ENST0000(	ENSP0000(	232	101	34	Q/R	cAg/cGg
4/4	ENST0000(	ENSP0000(	672	112	38	S/G	Agt/Ggt
29/37	ENST0000(	ENSP0000(	5959	5813	1938	R/H	cGc/cAc
6/14	ENST0000(	ENSP0000(	633	496	166	I/L	Att/Ctt
2/14	ENST0000(	ENSP0000(	179	43	15	G/S	Ggt/Agt
2/21	ENST0000(	ENSP0000(	91	73	25	D/N	Gat/Aat
1/7	ENST0000(	ENSP0000(	32	17	6	V/A	gTc/gCc
3/7	ENST0000(	ENSP0000(	508	382	128	V/I	Gtt/Att
10/12	ENST0000(	ENSP0000(	997	832	278	R/G	Agg/Ggg
4/9	ENST0000(	ENSP0000(	670	491	164	P/L	cCg/cTg
1/1	ENST0000(	ENSP0000(	3327	2842	948	V/L	Gtc/Ctc
13/14	ENST0000(	ENSP0000(	1568	1562	521	R/Q	cGg/cAg
7/7	ENST0000(	ENSP0000(	1375	1309	437	G/R	Ggg/Cgg
6/9	ENST0000(	ENSP0000(	595	419	140	R/Q	cGa/cAa
11/11	ENST0000(	ENSP0000(	2658	2587	863	A/T	Gca/Aca
7/12	ENST0000(	ENSP0000(	774	476	159	R/Q	cGa/cAa
4/17	ENST0000(	ENSP0000(	587	293	98	S/F	tCc/tTc
21/25	ENST0000(	ENSP0000(	2921	2806	936	I/V	Att/Gtt
5/13	ENST0000(	ENSP0000(	3053	2789	930	P/L	cCt/cTt
2/3	ENST0000(	ENSP0000(	203	164	55	A/V	gCa/gTa
14/15	ENST0000(	ENSP0000(	1691	1673	558	R/H	cGc/cAc
5/15	ENST0000(	ENSP0000(	414	379	127	A/T	Gcg/Acg
12/15	ENST0000(	ENSP0000(	2180	1855	619	E/Q	Gag/Cag
2/3	ENST0000(	ENSP0000(	224	191	64	G/V	gGc/gTc
11/12	ENST0000(	ENSP0000(	1073	949	317	V/I	Gtc/Atc
4/9	ENST0000(	ENSP0000(	735	476	159	R/Q	cGg/cAg
1/35	ENST0000(	ENSP0000(	2749	2299	767	G/S	Ggc/Agc
3/21	ENST0000(	ENSP0000(	279	115	39	V/L	Gtg/Ctg
4/20	ENST0000(	ENSP0000(	341	211	71	E/K	Gaa/Aaa
18/19	ENST0000(	ENSP0000(	1538	1454	485	T/I	aCa/aTa
28/28	ENST0000(	ENSP0000(	5874	5665	1889	E/K	Gag/Aag
16/16	ENST0000(	ENSP0000(	2461	2348	783	P/R	cCg/cGg
3/3	ENST0000(	ENSP0000(	1230	1090	364	P/A	Cca/Gca
26/26	ENST0000(	ENSP0000(	2887	2825	942	R/H	cGc/cAc
6/21	ENST0000(	ENSP0000(	768	700	234	V/M	Gtg/Atg
2/32	ENST0000(	ENSP0000(	292	109	37	P/A	Ccg/Gcg
7/20	ENST0000(	ENSP0000(	2351	2087	696	R/Q	cGg/cAg
5/6	ENST0000(	ENSP0000(	801	770	257	R/Q	cGg/cAg
30/69	ENST0000(	ENSP0000(	3279	3203	1068	R/Q	cGg/cAg
30/69	ENST0000(	ENSP0000(	3341	3265	1089	S/G	Agt/Ggt
13/15	ENST0000(	ENSP0000(	1413	1352	451	S/T	aGc/aCc
58/74	ENST0000(	ENSP0000(	9420	9239	3080	I/T	aTa/aCa
15/19	ENST0000(	ENSP0000(	2953	2338	780	V/I	Gtc/Atc

2/2	ENST0000(	ENSP0000(	2040	1582	528	T/S	Act/Tct
20/41	ENST0000(	ENSP0000(	2299	2233	745	H/D	Cat/Gat
4/9	ENST0000(	ENSP0000(	421	397	133	E/K	Gag/Aag
13/22	ENST0000(	ENSP0000(	3768	3656	1219	R/P	cGc/cCc
13/22	ENST0000(	ENSP0000(	2669	2557	853	L/F	Ctt/Ttt
3/16	ENST0000(	ENSP0000(	553	424	142	A/S	Gcc/Tcc
4/5	ENST0000(	ENSP0000(	2214	2048	683	N/S	aAt/aGt
3/3	ENST0000(	ENSP0000(	1690	1180	394	R/W	Cgg/Tgg
7/11	ENST0000(	ENSP0000(	597	584	195	R/H	cGt/cAt
1/6	ENST0000(	ENSP0000(	76	34	12	W/R	Tgg/Agg
4/10	ENST0000(	ENSP0000(	656	539	180	A/G	gCa/gGa
17/25	ENST0000(	ENSP0000(	15021	14909	4970	T/M	aCg/aTg
2/25	ENST0000(	ENSP0000(	12810	12698	4233	V/A	gTa/gCa
2/25	ENST0000(	ENSP0000(	11870	11758	3920	R/C	Cgt/Tgt
2/25	ENST0000(	ENSP0000(	7901	7789	2597	D/N	Gac/Aac
2/25	ENST0000(	ENSP0000(	7880	7768	2590	T/A	Acc/Gcc
2/25	ENST0000(	ENSP0000(	7877	7765	2589	D/H	Gac/Cac
2/25	ENST0000(	ENSP0000(	7862	7750	2584	S/A	Tca/Gca
2/25	ENST0000(	ENSP0000(	7854	7742	2581	S/N	aGc/aAc
2/25	ENST0000(	ENSP0000(	7493	7381	2461	D/H	Gac/Cac
24/28	ENST0000(	ENSP0000(	3347	3218	1073	S/T	aGc/aCc
2/18	ENST0000(	ENSP0000(	1538	1358	453	G/V	gGa/gTa
12/19	ENST0000(	ENSP0000(	1952	1930	644	V/M	Gtg/Atg
4/9	ENST0000(	ENSP0000(	1020	805	269	V/M	Gtg/Atg
9/11	ENST0000(	ENSP0000(	1676	1630	544	R/C	Cgc/Tgc
23/33	ENST0000(	ENSP0000(	4378	3883	1295	E/K	Gaa/Aaa
15/22	ENST0000(	ENSP0000(	2165	2047	683	S/C	Agt/Tgt
14/27	ENST0000(	ENSP0000(	3858	2179	727	T/A	Acg/Gcg
4/4	ENST0000(	ENSP0000(	494	476	159	D/A	gAc/gCc
13/24	ENST0000(	ENSP0000(	1600	1357	453	T/A	Acc/Gcc
64/74	ENST0000(	ENSP0000(	10493	9806	3269	R/Q	cGg/cAg
46/68	ENST0000(	ENSP0000(	8004	7297	2433	V/I	Gta/Ata
3/4	ENST0000(	ENSP0000(	592	331	111	D/N	Gac/Aac
4/5	ENST0000(	ENSP0000(	3415	3232	1078	K/E	Aaa/Gaa
11/13	ENST0000(	ENSP0000(	887	734	245	R/Q	cGa/cAa
6/10	ENST0000(	ENSP0000(	566	346	116	S/G	Agt/Ggt
3/9	ENST0000(	ENSP0000(	394	112	38	P/T	Cca/Aca
4/10	ENST0000(	ENSP0000(	470	293	98	T/I	aCa/aTa
5/7	ENST0000(	ENSP0000(	1416	805	269	G/R	Gga/Aga
9/9	ENST0000(	ENSP0000(	947	769	257	I/L	Att/Ctt
11/11	ENST0000(	ENSP0000(	2628	2440	814	S/R	Agc/Cgc
18/25	ENST0000(	ENSP0000(	3984	3317	1106	S/F	tCc/tTc
6/14	ENST0000(	ENSP0000(	718	661	221	T/A	Aca/Gca
10/47	ENST0000(	ENSP0000(	3088	2599	867	E/K	Gaa/Aaa
26/53	ENST0000(	ENSP0000(	5304	5125	1709	P/S	Ccc/Tcc
35/35	ENST0000(	ENSP0000(	4295	4166	1389	R/Q	cGg/cAg
10/10	ENST0000(	ENSP0000(	1133	1123	375	R/C	Cgt/Tgt
5/5	ENST0000(	ENSP0000(	1478	1013	338	V/A	gTc/gCc
1/1	ENST0000(	ENSP0000(	386	197	66	R/L	cGc/cTc
23/36	ENST0000(	ENSP0000(	3778	3161	1054	R/Q	cGa/cAa

8/23	ENST0000(	ENSP0000(	927	689	230	V/G	gTt/gGt
3/23	ENST0000(	ENSP0000(	453	215	72	D/G	gAt/gGt
15/20	ENST0000(	ENSP0000(	2539	2134	712	L/F	Ctt/Ttt
33/65	ENST0000(	ENSP0000(	4954	4312	1438	E/K	Gaa/Aaa
4/25	ENST0000(	ENSP0000(	727	379	127	V/I	Gtc/Atc
3/21	ENST0000(	ENSP0000(	1210	830	277	S/T	aGc/aCc
1/4	ENST0000(	ENSP0000(	168	95	32	R/L	cGt/cTt
2/15	ENST0000(	ENSP0000(	163	128	43	C/Y	tGt/tAt
1/1	ENST0000(	ENSP0000(	1603	1411	471	G/R	Ggc/Cgc
1/1	ENST0000(	ENSP0000(	1665	1517	506	I/N	aTc/aAc
1/4	ENST0000(	ENSP0000(	2141	1936	646	V/I	Gtc/Atc
1/4	ENST0000(	ENSP0000(	1005	818	273	V/G	gTc/gGc
1/4	ENST0000(	ENSP0000(	2025	1838	613	G/A	gGg/gCg
13/33	ENST0000(	ENSP0000(	1227	1162	388	I/V	Atc/Gtc
1/1	ENST0000(	ENSP0000(	712	491	164	T/I	aCc/aTc
4/9	ENST0000(	ENSP0000(	524	255	85	Q/H	caG/caC
1/4	ENST0000(	ENSP0000(	423	180	60	M/I	atG/atC
1/10	ENST0000(	ENSP0000(	1902	517	173	A/T	Gca/Aca
6/9	ENST0000(	ENSP0000(	1880	1775	592	N/S	aAc/aGc
5/13	ENST0000(	ENSP0000(	680	428	143	H/R	cAc/cGc
5/14	ENST0000(	ENSP0000(	2182	1618	540	V/L	Gta/Cta
5/5	ENST0000(	ENSP0000(	1092	844	282	Y/D	Tat/Gat
20/22	ENST0000(	ENSP0000(	4491	3953	1318	A/G	gCa/gGa
3/16	ENST0000(	ENSP0000(	283	116	39	R/Q	cGa/cAa
10/15	ENST0000(	ENSP0000(	4242	4202	1401	G/V	gGc/gTc
2/4	ENST0000(	ENSP0000(	4104	3871	1291	A/T	Gca/Aca
5/8	ENST0000(	ENSP0000(	1012	991	331	M/V	Atg/Gtg
5/8	ENST0000(	ENSP0000(	1010	989	330	A/V	gCt/gTt
5/8	ENST0000(	ENSP0000(	1006	985	329	T/A	Acc/Gcc
5/8	ENST0000(	ENSP0000(	1000	979	327	V/M	Gtg/Atg
5/8	ENST0000(	ENSP0000(	977	956	319	V/A	gTt/gCt
5/8	ENST0000(	ENSP0000(	946	925	309	M/V	Atg/Gtg
4/8	ENST0000(	ENSP0000(	912	891	297	S/R	agC/agA
4/8	ENST0000(	ENSP0000(	893	872	291	Q/P	cAa/cCa
4/8	ENST0000(	ENSP0000(	876	855	285	M/I	atG/atT
4/8	ENST0000(	ENSP0000(	874	853	285	M/V	Atg/Gtg
4/8	ENST0000(	ENSP0000(	850	829	277	Q/E	Caa/Gaa
4/8	ENST0000(	ENSP0000(	748	727	243	R/W	Cgg/Tgg
4/8	ENST0000(	ENSP0000(	644	623	208	P/H	cCc/cAc
3/8	ENST0000(	ENSP0000(	560	539	180	L/R	cTg/cGg
3/8	ENST0000(	ENSP0000(	548	527	176	A/E	gCg/gAg
3/8	ENST0000(	ENSP0000(	433	412	138	D/N	Gac/Aac
2/8	ENST0000(	ENSP0000(	133	112	38	R/W	Cgg/Tgg
2/8	ENST0000(	ENSP0000(	119	98	33	D/A	gAc/gCc
1/6	ENST0000(	ENSP0000(	147	41	14	A/V	gCg/gTg
19/58	ENST0000(	ENSP0000(	2507	2226	742	D/E	gaC/gaA
9/11	ENST0000(	ENSP0000(	2333	1882	628	V/L	Gtg/Ctg
11/13	ENST0000(	ENSP0000(	3476	3223	1075	E/K	Gag/Aag
15/32	ENST0000(	ENSP0000(	1763	1589	530	D/G	gAt/gGt
8/11	ENST0000(	ENSP0000(	1246	986	329	T/I	aCa/aTa

1/7	ENST0000(	ENSP0000(	1087	362	121	N/S	aAt/aGt
40/104	ENST0000(	ENSP0000(	10858	10615	3539	Y/H	Tac/Cac
2/2	ENST0000(	ENSP0000(	799	191	64	A/V	gCg/gTg
11/13	ENST0000(	ENSP0000(	1137	1111	371	I/V	Ata/Gta
2/2	ENST0000(	ENSP0000(	958	557	186	A/E	gCg/gAg
7/65	ENST0000(	ENSP0000(	1030	922	308	E/K	Gag/Aag
8/20	ENST0000(	ENSP0000(	1283	1178	393	Q/R	cAg/cGg
6/7	ENST0000(	ENSP0000(	1649	1310	437	G/D	gGt/gAt
11/23	ENST0000(	ENSP0000(	2544	2347	783	A/P	Gct/Cct
4/4	ENST0000(	ENSP0000(	1277	1133	378	R/Q	cGa/cAa
2/10	ENST0000(	ENSP0000(	1275	646	216	G/R	Ggg/Agg
2/9	ENST0000(	ENSP0000(	264	172	58	V/I	Gtc/Atc
13/17	ENST0000(	ENSP0000(	1693	1600	534	A/S	Gcg/Tcg
10/11	ENST0000(	ENSP0000(	2081	1468	490	M/V	Atg/Gtg
66/82	ENST0000(	ENSP0000(	10946	10739	3580	R/H	cGc/cAc
3/3	ENST0000(	ENSP0000(	797	781	261	P/S	Cct/Tct
10/16	ENST0000(	ENSP0000(	945	724	242	R/W	Cgg/Tgg
2/19	ENST0000(	ENSP0000(	321	250	84	G/S	Ggc/Agc
2/23	ENST0000(	ENSP0000(	218	164	55	S/L	tCg/tTg
2/8	ENST0000(	ENSP0000(	1948	1873	625	I/L	Att/Ctt
16/31	ENST0000(	ENSP0000(	1286	961	321	Y/H	Tac/Cac
5/9	ENST0000(	ENSP0000(	634	445	149	D/N	Gac/Aac
10/13	ENST0000(	ENSP0000(	1714	1538	513	S/F	tCc/tTc
2/13	ENST0000(	ENSP0000(	322	146	49	Q/R	cAg/cGg
3/4	ENST0000(	ENSP0000(	336	224	75	Q/R	cAg/cGg
7/9	ENST0000(	ENSP0000(	1127	859	287	H/N	Cat/Aat
13/20	ENST0000(	ENSP0000(	3757	3397	1133	V/L	Gtg/Ctg
11/19	ENST0000(	ENSP0000(	1507	1378	460	R/C	Cgc/Tgc
6/8	ENST0000(	ENSP0000(	1415	1282	428	A/T	Gca/Aca
12/13	ENST0000(	ENSP0000(	1422	1319	440	R/Q	cGa/cAa
6/7	ENST0000(	ENSP0000(	2393	2234	745	R/Q	cGg/cAg
2/12	ENST0000(	ENSP0000(	908	817	273	T/S	Aca/Tca
10/19	ENST0000(	ENSP0000(	1392	1085	362	P/L	cCg/cTg
16/21	ENST0000(	ENSP0000(	1821	1790	597	L/S	tTa/tCa
2/12	ENST0000(	ENSP0000(	404	175	59	D/N	Gat/Aat
1/30	ENST0000(	ENSP0000(	202	79	27	G/S	Ggc/Agc
9/9	ENST0000(	ENSP0000(	1004	944	315	R/H	cGc/cAc
8/10	ENST0000(	ENSP0000(	1354	1354	452	D/N	Gat/Aat
4/4	ENST0000(	ENSP0000(	793	715	239	H/Y	Cac/Tac
2/4	ENST0000(	ENSP0000(	696	338	113	L/P	cTa/cCa
7/9	ENST0000(	ENSP0000(	942	886	296	G/R	Gga/Aga
31/40	ENST0000(	ENSP0000(	3857	3821	1274	N/S	aAt/aGt
1/13	ENST0000(	ENSP0000(	70	59	20	A/V	gCg/gTg
2/6	ENST0000(	ENSP0000(	742	686	229	T/M	aCg/aTg
1/16	ENST0000(	ENSP0000(	35	13	5	G/R	Gga/Cga
5/7	ENST0000(	ENSP0000(	503	374	125	S/N	aGt/aAt
2/14	ENST0000(	ENSP0000(	333	86	29	N/S	aAc/aGc
1/9	ENST0000(	ENSP0000(	161	37	13	I/V	Atc/Gtc
13/14	ENST0000(	ENSP0000(	1292	1279	427	K/E	Aaa/Gaa
8/10	ENST0000(	ENSP0000(	1275	1198	400	P/T	Cca/Aca

10/16		ENST0000(	ENSP0000(	750	613	205	V/M	Gtg/Atg
3/3		ENST0000(	ENSP0000(	1466	1085	362	F/S	tTc/tCc
3/12		ENST0000(	ENSP0000(	1534	1508	503	T/K	aCg/aAg
14/24		ENST0000(	ENSP0000(	2938	2926	976	L/V	Ctg/Gtg
13/16		ENST0000(	ENSP0000(	1782	1714	572	Q/E	Cag/Gag
10/12		ENST0000(	ENSP0000(	1676	1621	541	E/K	Gag/Aag
4/12		ENST0000(	ENSP0000(	731	676	226	A/T	Gcc/Acc
11/11		ENST0000(	ENSP0000(	1466	1316	439	V/A	gTc/gCc
11/13		ENST0000(	ENSP0000(	3418	3230	1077	T/K	aCg/aAg
6/9		ENST0000(	ENSP0000(	636	610	204	D/N	Gac/Aac
34/39		ENST0000(	ENSP0000(	5421	5356	1786	V/I	Gtc/Atc
8/19		ENST0000(	ENSP0000(	4187	4036	1346	A/T	Gcc/Acc
12/30		ENST0000(	ENSP0000(	2794	2545	849	P/T	Cct/Act
1/10		ENST0000(	ENSP0000(	219	190	64	V/I	Gtc/Atc
1/1		ENST0000(	ENSP0000(	12	12	4	N/K	aaT/aaA
1/9		ENST0000(	ENSP0000(	34	22	8	P/A	Cca/Gca
1/1		ENST0000(	ENSP0000(	334	334	112	T/A	Aca/Gca
1/1		ENST0000(	ENSP0000(	580	580	194	T/A	Aca/Gca
2/2		ENST0000(	ENSP0000(	1311	1162	388	P/S	Cca/Tca
1/1		ENST0000(	ENSP0000(	581	469	157	E/K	Gaa/Aaa
2/5		ENST0000(	ENSP0000(	183	67	23	M/V	Atg/Gtg
2/2		ENST0000(	ENSP0000(	636	587	196	N/S	aAt/aGt
1/1		ENST0000(	ENSP0000(	2001	1963	655	S/T	Tcc/Acc
1/1		ENST0000(	ENSP0000(	460	422	141	H/R	cAc/cGc
1/1		ENST0000(	ENSP0000(	451	413	138	Q/L	cAg/cTg
1/1		ENST0000(	ENSP0000(	444	406	136	S/T	Tct/Act
8/13		ENST0000(	ENSP0000(	1411	1323	441	R/S	agG/agT
3/3		ENST0000(	ENSP0000(	496	307	103	Q/K	Caa/Aaa
6/26		ENST0000(	ENSP0000(	3901	3610	1204	T/S	Acc/Tcc
16/16		ENST0000(	ENSP0000(	2083	1948	650	R/C	Cgc/Tgc
7/12		ENST0000(	ENSP0000(	1390	1249	417	G/R	Gga/Cga
6/23		ENST0000(	ENSP0000(	996	832	278	A/T	Gcg/Acg
10/26		ENST0000(	ENSP0000(	1288	1196	399	S/F	tCt/tTt
16/21		ENST0000(	ENSP0000(	3098	2924	975	R/Q	cGa/cAa
11/14		ENST0000(	ENSP0000(	1923	1775	592	G/A	gGa/gCa
21/29		ENST0000(	ENSP0000(	2342	2163	721	K/N	aaG/aaT
3/12		ENST0000(	ENSP0000(	339	319	107	D/N	Gat/Aat
14/22		ENST0000(	ENSP0000(	1645	1610	537	R/Q	cGg/cAg
16/19		ENST0000(	ENSP0000(	2236	2020	674	G/R	Ggg/Agg
2/3		ENST0000(	ENSP0000(	216	10	4	G/R	Ggg/Agg
8/10		ENST0000(	ENSP0000(	1706	1669	557	W/R	Tgg/Cgg
19/76		ENST0000(	ENSP0000(	2019	1432	478	S/P	Tct/Cct
14/28		ENST0000(	ENSP0000(	1987	1715	572	P/L	cCg/cTg
12/12		ENST0000(	ENSP0000(	892	827	276	Q/R	cAg/cGg
9/11		ENST0000(	ENSP0000(	2077	1892	631	R/Q	cGg/cAg
4/8		ENST0000(	ENSP0000(	916	895	299	E/K	Gag/Aag
8/13		ENST0000(	ENSP0000(	743	707	236	T/M	aCg/aTg
11/27		ENST0000(	ENSP0000(	1351	1280	427	S/L	tCg/tTg
ding	2/2	ENST00000567955.3:c.-56-1G>A						
ding	12/17	ENST00000683005.1:c.1410-2A>G						

ıding	19/25	ENST00000448612.6:c.2004-2A>G						
ıding	1/2	ENST00000356661.7:c.-138-2del						
ed_pseudo	1/1	ENST00000622095.1:n.136-2G>A						
	4/6	ENST00000650234.1:n.680-2del						
ıding	14/14	ENST00000367080.8:c.1350+1G>T						
ıding	6/6	ENST00000278319.10:c.712+1G>A						
ıding	7/9	ENST00000354883.11:c.568+2T>G						
ıding	2/4	ENST00000246868.7:c.258+2T>C						
d_unitary_	1/9	ENST00000462729.2:n.50+1G>T						
	2/4	ENST00000661690.1:n.285+1G>A						
1/1		ENST0000(	ENSP0000(	537	487	163	Q/*	Caa/Taa
25/71		ENST0000(	ENSP0000(	3353	3161	1054	S/*	tCa/tGa
1/6		ENST0000(	ENSP0000(	348	288	96	C/*	tgC/tgA
2/3		ENST0000(	ENSP0000(	1682	1227	409	C/*	tgT/tgA
1/6		ENST0000(	ENSP0000(	221	96	32	W/*	tgG/tgA
23/23		ENST0000(	ENSP0000(	3445	3343	1115	Q/*	Cag/Tag
3/4		ENST0000(	ENSP0000(	311	157	53	R/*	Cga/Tga
4/4		ENST0000(	ENSP0000(	1378	1260	420	Y/*	taT/taG
5/14		ENST0000(	ENSP0000(	583	416	139	W/*	tGg/tAg
2/3		ENST0000(	ENSP0000(	219	168	56	Y/*	taC/taG
2/5		ENST0000(	ENSP0000(	424	135	45	Y/*	taT/taA
1/11		ENST0000(	ENSP0000(	293	58	20	R/*	Cga/Tga
10/17		ENST0000(	ENSP0000(	982-983	832-833	278	I/TGVDHP(	att/aCTGG

ne_descript	DISTANCE	STRAND	FLAGS	RIANT_CLA	MBOL_SOU	HGNC_ID	CANONICA	ANE_SELE
Atrophin 1 (Atn1), Tra		1		deletion	HGNC	HGNC:303	YES	NM_00194
Atrophin 1 (Atn1), Tra		1		deletion	HGNC	HGNC:303	YES	NM_00194
Neurofibromin 1 (Nf1		1		deletion	HGNC	HGNC:776	YES	NM_00104
Mks Transition Zone (		-1		deletion	HGNC	HGNC:712	YES	NM_01777
Junctional Sarcoplasm		-1		deletion	HGNC	HGNC:249	YES	NM_14461
At-Rich Interaction Do		1		insertion	HGNC	HGNC:180	YES	NM_00137
At-Rich Interaction Do		1		insertion	HGNC	HGNC:180	YES	NM_00137
Family With Sequence		-1		deletion	HGNC	HGNC:419	YES	NM_00114
Zinc Finger X-Linked D		-1		deletion	HGNC	HGNC:131	YES	NM_00715
Zinc Finger X-Linked D		-1		insertion	HGNC	HGNC:131	YES	NM_00715
Syndecan 3 (Sdc3), M		-1		deletion	HGNC	HGNC:106	YES	NM_01465
Keratin Associated Pro		1		deletion	HGNC	HGNC:236	YES	NM_00100
Immunoglobulin Supe		-1		deletion	HGNC	HGNC:267	YES	NM_17358
Keratin Associated Pro		1		deletion	HGNC	HGNC:236	YES	NM_02104
Cwf19 Like Cell Cycle		-1		deletion	HGNC	HGNC:265	YES	NM_15243
Heat Shock Protein 90		1		deletion	HGNC	HGNC:120	YES	NM_00329
GGCTGTGGCTATGGCT		-1		deletion			YES	NM_00137
Myocyte Enhancer Fa		1		deletion	HGNC	HGNC:699	YES	NM_00131
Periplakin (Ppl), Mrna		-1		deletion	HGNC	HGNC:927	YES	NM_00270
Zinc Finger Protein 46		1		deletion	HGNC	HGNC:232	YES	NM_00136
Nucleolin (Ncl), Mrna.		-1		deletion	HGNC	HGNC:766	YES	NM_00538
Mediator Complex Su		1		deletion	HGNC	HGNC:142	YES	NM_00100
Male-Enhanced Antig		-1		deletion	HGNC	HGNC:698	YES	NM_01462
Swi/Snf Related, Matr		1		deletion	HGNC	HGNC:110	YES	NM_00307
Nbpf Member 10 (Nb		-1		insertion	HGNC	HGNC:319	YES	NM_00130
Protein Phosphatase 1		1		insertion	HGNC	HGNC:930	YES	NM_00624
Dachshund Family Tra		-1		insertion	HGNC	HGNC:266	YES	NM_08075
Signal Recognition Pa		-1		insertion	HGNC	HGNC:112	YES	NM_00313
Keratin Associated Pro		-1		insertion	HGNC	HGNC:205	YES	NM_19868
Scavenger Receptor C		-1		insertion	HGNC	HGNC:198	YES	NM_18289
Mitochondrial Riboso		1		insertion	HGNC	HGNC:876	YES	NM_01664
Lamin B1 (Lmnb1), Tra		1		insertion	HGNC	HGNC:663	YES	NM_00557
Mucin 3A, Cell Surface		1		insertion	HGNC	HGNC:751	YES	NM_00596
Cilia And Flagella Asso		-1		SNV	HGNC	HGNC:293	YES	NM_00130
Cytoplasm, Cytoskele		-1		SNV	HGNC	HGNC:248	YES	NM_01470
Tnf Receptor Superfa		1		SNV	HGNC	HGNC:119	YES	NM_00124
Prame Family Membe		1		SNV	HGNC	HGNC:267	YES	NM_00109
Peptidyl Arginine Dei		1		SNV	HGNC	HGNC:183	YES	NM_01238
Intermediate Filamen		-1		SNV	HGNC	HGNC:270	YES	NM_00113
Von Willebrand Facto		1		SNV	HGNC	HGNC:265	YES	NM_00103
Eukaryotic Translatior		-1		SNV	HGNC	HGNC:329	YES	NM_00139
Rh Blood Group D Ant		1		SNV	HGNC	HGNC:100	YES	NM_01612
Selenoprotein N (Sele		1		SNV	HGNC	HGNC:159	YES	NM_02045
Four And A Half Lim D		-1		SNV	HGNC	HGNC:370	YES	NM_00446
Ribosomal Modificati		1		SNV	HGNC	HGNC:287	YES	NM_17364
Cilia And Flagella Asso		1		SNV	HGNC	HGNC:264	YES	NM_00137
Diencephalon/Mesen		1		SNV	HGNC	HGNC:190	YES	NM_17222
Complement C8 Alpha		1		SNV	HGNC	HGNC:135	YES	NM_00056
Cache Domain Contai		1		SNV	HGNC	HGNC:293	YES	NM_02092

Interferon Induced Pr	1	SNV	HGNC	HGNC:178	YES	NM_00682
Chitobiase (Ctbs), Mrr	-1	SNV	HGNC	HGNC:249	YES	NM_00438
Dynein Axonemal Inte	1	SNV	HGNC	HGNC:307	YES	NM_14517
Sas-6 Centriolar Asser	-1	SNV	HGNC	HGNC:254	YES	NM_19429
Denn Domain Contair	-1	SNV	HGNC	HGNC:247	YES	NM_00125
Denn Domain Contair	-1	SNV	HGNC	HGNC:247	YES	NM_00125
Transcription Termina	1	SNV	HGNC	HGNC:123	YES	NM_00359
Notch Receptor 2 (No	-1	SNV	HGNC	HGNC:788	YES	NM_02440
Srgap2 Antisense Rna	1	SNV	HGNC	HGNC:305	YES	NM_00132
Family With Sequence	-1	SNV	HGNC	HGNC:306	YES	NM_00128
Circadian Associated I	1	SNV	HGNC	HGNC:252	YES	NM_14469
Adamts Like 4 (Adamt	1	SNV	HGNC	HGNC:197	YES	NM_01903
Hornerin (Hrnr), Mrna	-1	SNV	HGNC	HGNC:208	YES	NM_00100
Hornerin (Hrnr), Mrna	-1	SNV	HGNC	HGNC:208	YES	NM_00100
Creb Regulated Trans	-1	SNV	HGNC	HGNC:273	YES	NM_18171
Phosphomevalonate I	-1	SNV	HGNC	HGNC:914	YES	NM_00655
Rho Guanine Nucleoti	-1	SNV	HGNC	HGNC:145	YES	NM_19823
Atypical Chemokine R	1	SNV	HGNC	HGNC:403	YES	NM_00203
Coagulation Factor V	-1	SNV	HGNC	HGNC:354	YES	NM_00013
Proline Rich Coiled-Cc	1	SNV	HGNC	HGNC:249	YES	NM_00138
Most Important Serin	-1	SNV	HGNC	HGNC:775	YES	NM_00048
Coagulation Factor Xii	-1	SNV	HGNC	HGNC:353	YES	NM_00199
Assembly Factor For S	-1	SNV	HGNC	HGNC:190	YES	NM_01813
Immunoglobulin Like	1	SNV	HGNC	HGNC:246	YES	NM_00116
Ethanolamine Kinase	-1	SNV	HGNC	HGNC:255	YES	NM_01820
Cyclin Dependent Kin	1	SNV	HGNC	HGNC:875	YES	NM_21250
Complement Compor	1	SNV	HGNC	HGNC:132	YES	NM_00071
Glutamyl-Prolyl-Trna	-1	SNV	HGNC	HGNC:341	YES	NM_00444
Wnt Family Member	-1	SNV	HGNC	HGNC:127	YES	NM_00339
Obscurin, Cytoskeleta	1	SNV	HGNC	HGNC:157	YES	NM_00138
Obscurin, Cytoskeleta	1	SNV	HGNC	HGNC:157	YES	NM_00138
The Mammalian Zona	-1	SNV	HGNC	HGNC:157	YES	NM_02118
Cholinergic Receptor	1	SNV	HGNC	HGNC:195	YES	NM_00137
Suppressor Of Variega	1	SNV	HGNC	HGNC:172	YES	NM_00119
Rho Gtpase Activating	-1	SNV	HGNC	HGNC:237	YES	NM_02082
Yme1 Like 1 Atpase (Y	-1	SNV	HGNC	HGNC:128	YES	NM_01426
Supervillin (Svil), Tran	-1	SNV	HGNC	HGNC:114	YES	NM_02173
Enhancer Of Polycom	-1	SNV	HGNC	HGNC:198	YES	NM_00127
Ankyrin Repeat Doma	1	SNV	HGNC	HGNC:172	YES	NM_05299
Depp1 Autophagy Reg	-1	SNV	HGNC	HGNC:233	YES	NM_00702
May Be Involved In Tr	-1	SNV	HGNC	HGNC:235	YES	NM_15303
Wash Complex Subun	1	SNV	HGNC	HGNC:234	YES	NM_00100
Inositol Polyphosphat	-1	SNV	HGNC	HGNC:207	YES	NM_15223
Shieldin Complex Sub	1	SNV	HGNC	HGNC:287	YES	NM_00133
Interferon Induced Pr	1	SNV	HGNC	HGNC:133	YES	NM_01242
Cytochrome P450 Far	1	SNV	HGNC	HGNC:205	YES	NM_18337
Phospholipase C Epsil	1	SNV	HGNC	HGNC:171	YES	NM_01634
Cytochrome P450 Far	1	SNV	HGNC	HGNC:262	YES	NM_00077
Zinc Finger Protein 51	1	SNV	HGNC	HGNC:290	YES	NM_00133
Sec31 Homolog B, Co	-1	SNV	HGNC	HGNC:231	YES	NM_01549

Cue Domain Containir	-1	SNV	HGNC	HGNC:283	YES	NM_02404
Cilia And Flagella Assc	-1	SNV	HGNC	HGNC:266	YES	NM_02514
Inositol Polyphosphat	1	SNV	HGNC	HGNC:170	YES	NM_01493
Btb Domain Containir	1	SNV	HGNC	HGNC:263	YES	NM_14458
O-6-Methylguanine-D	1	SNV	HGNC	HGNC:705	YES	NM_00241
Transcription Elongati	-1	SNV	HGNC	HGNC:235	YES	NM_17493
Dihydropyrimidinase	1	SNV	HGNC	HGNC:301	YES	NM_00642
Cilia And Flagella Assc	-1	SNV	HGNC	HGNC:252	YES	NM_00120
Vent Homeobox (Ven	1	SNV	HGNC	HGNC:136	YES	NM_01446
Plakophilin 3 (Pkp3), T	1	SNV	HGNC	HGNC:902	YES	NM_00718
Lamin Tail Domain Co	-1	SNV	HGNC	HGNC:285	YES	NM_17357
Lamin Tail Domain Co	-1	SNV	HGNC	HGNC:285	YES	NM_17357
Phd And Ring Finger D	1	SNV	HGNC	HGNC:243	YES	NM_00128
Mitochondrion. (From	1	SNV	HGNC	HGNC:103	YES	NM_02113
	1	SNV	HGNC	HGNC:10322		
Tripartite Motif Conta	-1	SNV	HGNC	HGNC:113	YES	NM_00314
Olfactory Receptor Fa	-1	SNV	HGNC	HGNC:151	YES	NM_03077
Family With Sequence	-1	SNV	HGNC	HGNC:253	YES	NM_00109
Olfactory Receptor Fa	-1	SNV	HGNC	HGNC:147	YES	NM_15344
Odorant Receptor (Pc	-1	SNV	HGNC	HGNC:151	YES	NM_00100
Ubiquitin Specific Pep	1	SNV	HGNC	HGNC:200	YES	NM_00128
Potassium Inwardly R	-1	SNV	HGNC	HGNC:625	YES	NM_00052
Igsf22-As1 (From Gen	-1	SNV	HGNC	HGNC:267	YES	NM_17358
Olfactory Receptor Fa	1	SNV	HGNC	HGNC:312	YES	
Olfactory Receptor Fa	1	SNV	HGNC	HGNC:151	YES	NM_00100
Olfactory Receptor Fa	-1	SNV	HGNC	HGNC:832	YES	NM_00100
Heterogeneous Nucle	-1	SNV	HGNC	HGNC:254	YES	NM_00107
Signal-Induced Prolife	1	SNV	HGNC	HGNC:108	YES	NM_00674
Cd248 Molecule (Cd2	-1	SNV	HGNC	HGNC:182	YES	NM_02040
Mediates Equilibrativ	-1	SNV	HGNC	HGNC:110	YES	NM_00153
Bardet-Biedl Syndrom	1	SNV	HGNC	HGNC:966	YES	NM_02464
Fibroblast Growth Fac	-1	SNV	HGNC	HGNC:367	YES	NM_00511
Solute Carrier Organic	1	SNV	HGNC	HGNC:109	YES	NM_00725
Teneurin Transmemb	-1	SNV	HGNC	HGNC:299	YES	NM_00109
V-Set And Transmem	-1	SNV	HGNC	HGNC:344	YES	NM_00114
Ankyrin Repeat And K	1	SNV	HGNC	HGNC:210	YES	NM_17851
Catalyzes The Initial S	-1	SNV	HGNC	HGNC:299	YES	NM_00138
Olfactory Receptor Fa	1	SNV	HGNC	HGNC:816	YES	NM_00135
Roundabout Guidance	1	SNV	HGNC	HGNC:134	YES	NM_02237
Prostate And Testis E	1	SNV	HGNC	HGNC:354	YES	NM_00114
St3 Beta-Galactoside	1	SNV	HGNC	HGNC:108	YES	NM_00125
Rad9-Hus1-Rad1 Inte	1	SNV	HGNC	HGNC:282	YES	NM_00125
Complement C1R Sub	-1	SNV	HGNC	HGNC:212	YES	NM_01654
C-Type Lectin Domain	1	SNV	HGNC	HGNC:145	YES	NM_00100
Proline Rich Protein B	-1	SNV	HGNC	HGNC:934	YES	NM_00272
Proline Rich Protein B	-1	SNV	HGNC	HGNC:933	YES	
Contains 1 Pi3K/Pi4K	1	SNV	HGNC	HGNC:897	YES	NM_00128
	1	SNV	HGNC	HGNC:191	YES	NM_00137
Atp Binding Cassette	-1	SNV	HGNC	HGNC:66	YES	NM_00516
Peripherin (Prph), Mr	1	SNV	HGNC	HGNC:946	YES	NM_00626

Nck Associated Protei	-1	SNV	HGNC	HGNC:293	YES	NM_00103
Keratin 81 (Krt81), Mı	-1	SNV	HGNC	HGNC:645	YES	NM_00228
Keratin 6B (Krt6B), Mı	-1	SNV	HGNC	HGNC:644	YES	NM_00555
Erb-B2 Receptor Tyro:	1	SNV	HGNC	HGNC:343	YES	NM_00198
Carries Out A Dual Fu	-1	SNV	HGNC	HGNC:113	YES	NM_00315
Ptprf Interacting Prot	-1	SNV	HGNC	HGNC:924	YES	NM_00362
Leucine Rich Repeats	1	SNV	HGNC	HGNC:257	YES	NM_00107
Fyve, Rhogef And Ph [	-1	SNV	HGNC	HGNC:217	YES	NM_01835
Fyve, Rhogef And Ph [	-1	SNV	HGNC	HGNC:217	YES	NM_01835
Fyve, Rhogef And Ph [	-1	SNV	HGNC	HGNC:217	YES	NM_01835
2'-5'-Oligoadenylate S	1	SNV	HGNC	HGNC:808	YES	NM_00618
Serine/Arginine Repet	1	SNV	HGNC	HGNC:293	YES	NM_19428
Dynein Axonemal Hea	1	SNV	HGNC	HGNC:294	YES	NM_00137
Deah-Box Helicase 37	-1	SNV	HGNC	HGNC:172	YES	NM_03265
Membrane| Single-Pa	1	SNV	HGNC	HGNC:254	YES	NM_00113
Zinc Finger Mym-Type	-1	SNV	HGNC	HGNC:130	YES	NM_00114
Atpase H+/K+ Transp	1	SNV	HGNC	HGNC:138	YES	NM_00167
Fry Microtubule Bindi	1	SNV	HGNC	HGNC:203	YES	NM_02303
Involved In Double-St	1	SNV	HGNC	HGNC:110	YES	NM_00005
N-Alpha-Acetyltransfe	1	SNV	HGNC	HGNC:261	YES	NM_02456
Cln5, Intracellular Tra	1	SNV	HGNC	HGNC:207	YES	NM_00649
Myc Binding Protein 2	-1	SNV	HGNC	HGNC:233	YES	NM_01505
Dnaj Heat Shock Prot	1	SNV	HGNC	HGNC:943	YES	NM_00626
Olfactory Receptor Fa	1	SNV	HGNC	HGNC:307	YES	NM_00101
Pote Ankyrin Domain	1	SNV	HGNC	HGNC:370	YES	NM_00114
Olfactory Receptor Fa	-1	SNV	HGNC	HGNC:147	YES	NM_00119
Egila (From Genesyml	1	SNV	HGNC	HGNC:100	YES	NM_00293
Ribonuclease A Family	-1	SNV	HGNC	HGNC:100	YES	NM_00293
Ndrg Family Member	-1	SNV	HGNC	HGNC:144	YES	NM_00132
Leucine Rich Repeat A	1	SNV	HGNC	HGNC:203	YES	NM_15244
Exocyst Complex Com	-1	SNV	HGNC	HGNC:106	YES	NM_00654
Zinc Finger And Btb D	-1	SNV	HGNC	HGNC:131	YES	NM_00697
Sushi Domain Contain	1	SNV	HGNC	HGNC:199	YES	NM_01473
Mitogen-Activated Pr	-1	SNV	HGNC	HGNC:686	YES	NM_00128
Nrde-2, Necessary For	-1	SNV	HGNC	HGNC:201	YES	NM_01797
Autophagy Related 2E	-1	SNV	HGNC	HGNC:201	YES	NM_01803
Brain Enriched Guany	-1	SNV	HGNC	HGNC:241	YES	NM_00138
Retrotransposon Gag	-1	SNV	HGNC	HGNC:146	YES	NM_00113
Lbh Domain Containir	1	SNV	HGNC	HGNC:523	YES	NM_00133
Ahnak Nucleoprotein	-1	SNV	HGNC	HGNC:201	YES	NM_13842
Ahnak Nucleoprotein	-1	SNV	HGNC	HGNC:201	YES	NM_13842
Ahnak Nucleoprotein	-1	SNV	HGNC	HGNC:201	YES	NM_13842
Ahnak Nucleoprotein	-1	SNV	HGNC	HGNC:201	YES	NM_13842
Ahnak Nucleoprotein	-1	SNV	HGNC	HGNC:201	YES	NM_13842
Ahnak Nucleoprotein	-1	SNV	HGNC	HGNC:201	YES	NM_13842
Ahnak Nucleoprotein	-1	SNV	HGNC	HGNC:201	YES	NM_13842
Golgin A8 Family Men	-1	SNV	HGNC	HGNC:444	YES	NM_00128
Golgin A8 Family Men	-1	SNV	HGNC	HGNC:386	YES	NM_00128
Golgin A8 Family Men	-1	SNV	HGNC	HGNC:386	YES	NM_00128
Golgin A8 Family Men	-1	SNV	HGNC	HGNC:386	YES	NM_00128

	-1	SNV	HGNC	HGNC:376	YES	NM_00127
Exonuclease 3'-5' Dom	-1	SNV	HGNC	HGNC:285	YES	NM_00128
Wd Repeat Domain 7	1	SNV	HGNC	HGNC:257	YES	NM_0249C
Signal Peptide Peptid	-1	SNV	HGNC	HGNC:302	YES	NM_0328C
Cingulin Like 1 (Cgnl1)	1	SNV	HGNC	HGNC:259	YES	NM_03286
Bcl2 Interacting Prote	-1	SNV	HGNC	HGNC:108	YES	NM_00433
Vacuolar Protein Sorti	-1	SNV	HGNC	HGNC:235	YES	NM_02082
Gram Domain Contair	-1	SNV	HGNC	HGNC:272	YES	NM_00101
Golgin A6 Family Men	1	SNV	HGNC	HGNC:322	YES	NM_00116
Membrane Integral N	1	SNV	HGNC	HGNC:291	YES	NM_0152C
Alpha Kinase 3 (Alpk3	1	SNV	HGNC	HGNC:175	YES	NM_02077
Semaphorin 4B (Sema	1	SNV	HGNC	HGNC:107	YES	NM_19892
Wap, Follistatin/Kazal	1	SNV	HGNC	HGNC:309	YES	NM_05328
The Sequence Shown	1	SNV	HGNC	HGNC:269	YES	NM_14529
Telomere Maintenanc	1	SNV	HGNC	HGNC:290	YES	NM_01611
Spla/Ryanodine Recep	-1	SNV	HGNC	HGNC:306	YES	NM_08086
Ribosomal Protein L3	-1	SNV	HGNC	HGNC:103	YES	NM_00506
Ring Finger Protein 15	1	SNV	HGNC	HGNC:232	YES	NM_1749C
Mefv Innate Immuity	-1	SNV	HGNC	HGNC:699	YES	NM_00024
Mefv Innate Immuity	-1	SNV	HGNC	HGNC:699	YES	NM_00024
Cell Death Inducing P5	-1	SNV	HGNC	HGNC:132	YES	NM_01339
Tektin 5 (Tekt5), Mrna	-1	SNV	HGNC	HGNC:265	YES	NM_14467
Protamine 2 (Prm2), T	-1	SNV	HGNC	HGNC:944	YES	NM_00276
Nodal Modulator 1 (N	1	SNV	HGNC	HGNC:300	YES	NM_01428
Myosin Heavy Chain 1	-1	SNV	HGNC	HGNC:756	YES	NM_00247
Protein Disulfide Isom	-1	SNV	HGNC	HGNC:273	YES	NM_17492
Protein Disulfide Isom	-1	SNV	HGNC	HGNC:273	YES	NM_17492
Dynein Axonemal Hea	-1	SNV	HGNC	HGNC:294	YES	NM_00134
Trinucleotide Repeat	1	SNV	HGNC	HGNC:119	YES	NM_01449
Nuclear Pore Complex	1	SNV	HGNC	HGNC:419	YES	
Zinc Finger Protein 26	1	SNV	HGNC	HGNC:130	YES	NM_00341
Nedd4 Binding Protei	-1	SNV	HGNC	HGNC:298	YES	NM_15302
Transcriptional Repre	-1	SNV	HGNC	HGNC:105	YES	NM_00296
Chromosome 16 Oper	1	SNV	HGNC	HGNC:295	YES	NM_02518
Hydin Axonemal Cent	-1	SNV	HGNC	HGNC:193	YES	NM_00127
Hydin Axonemal Cent	-1	SNV	HGNC	HGNC:193	YES	NM_00127
Polycystin 1 Like 3, Tr	-1	SNV	HGNC	HGNC:217	YES	NM_18153
Deah-Box Helicase 38	1	SNV	HGNC	HGNC:172	YES	NM_0140C
Chromodomain Y Like	-1	SNV	HGNC	HGNC:230	YES	NM_15234
Hydroxysteroid Dehyd	-1	SNV	HGNC	HGNC:164	YES	NM_03146
Cytosolic Thiouridylas	1	SNV	HGNC	HGNC:280	YES	NM_00101
Cytosolic Thiouridylas	1	SNV	HGNC	HGNC:280	YES	NM_00101
Piezo Type Mechanos	-1	SNV	HGNC	HGNC:289	YES	NM_00114
Spermatogenesis Asso	-1	SNV	HGNC	HGNC:283	YES	NM_15233
Clustered Mitochondr	-1	SNV	HGNC	HGNC:290	YES	NM_00136
Integrin Subunit Alpha	-1	SNV	HGNC	HGNC:614	YES	NM_0022C
Purinergic Receptor P	-1	SNV	HGNC	HGNC:853	YES	NM_00255
Enolase 3 (Eno3), Trar	1	SNV	HGNC	HGNC:335	YES	NM_05301
Tektin 1 (Tekt1), Mrna	-1	SNV	HGNC	HGNC:155	YES	NM_05328
Solute Carrier Family	1	SNV	HGNC	HGNC:110	YES	NM_00104

Cst Telomere Replicat	-1	SNV	HGNC	HGNC:261	YES	NM_0250[illegible]
Heparan Sulfate-Gluc	-1	SNV	HGNC	HGNC:519	YES	NM_00604
Sperm Associated Ant	-1	SNV	HGNC	HGNC:134	YES	NM_0064[illegible]
Dna Topoisomerase Ii	-1	SNV	HGNC	HGNC:119	YES	NM_0010[illegible]
Keratin Associated Pr	-1	SNV	HGNC	HGNC:172	YES	NM_0319[illegible]
Keratin Associated Pr	1	SNV	HGNC	HGNC:189	YES	NM_0012[illegible]
Pleckstrin Homology,	-1	SNV	HGNC	HGNC:261	YES	NM_02492
Mapt Antisense Rna 1	1	SNV	HGNC	HGNC:235	YES	NM_0043[illegible]
Cell Division Cycle 27	-1	SNV	HGNC	HGNC:172	YES	NM_0012[illegible]
Zinc Finger Protein 65	-1	SNV	HGNC	HGNC:291	YES	NM_00114
Mks Transition Zone	-1	SNV	HGNC	HGNC:712	YES	NM_0177[illegible]
Regulator Of G Protei	1	SNV	HGNC	HGNC:100	YES	NM_00383
Glutamate Ionotropic	-1	SNV	HGNC	HGNC:458	YES	NM_00083
Myosin Xvb (From Hg	1	SNV	HGNC	HGNC:140	YES	NM_0013[illegible]
Forkhead Box J1 (Foxj	-1	SNV	HGNC	HGNC:381	YES	NM_0014[illegible]
Fascin Actin-Bundling	1	SNV	HGNC	HGNC:396	YES	NM_01241
Establishment Of Siste	-1	SNV	HGNC	HGNC:246	YES	NM_05291
Oxysterol Binding Pro	-1	SNV	HGNC	HGNC:163	YES	NM_0805[illegible]
Asxl Transcriptional R	1	SNV	HGNC	HGNC:293	YES	NM_03063
Ph Domain And Leuci	1	SNV	HGNC	HGNC:206	YES	NM_19444
Zinc Finger Protein 23	1	SNV	HGNC	HGNC:130	YES	NM_0013[illegible]
Apc Regulator Of Wnt	1	SNV	HGNC	HGNC:240	YES	NM_0058[illegible]
Signal Peptide Peptid	1	SNV	HGNC	HGNC:306	YES	NM_1529[illegible]
Zinc Finger Rna Bindir	-1	SNV	HGNC	HGNC:291	YES	NM_01517
Chromatin Assembly	1	SNV	HGNC	HGNC:191	YES	NM_0054[illegible]
May Play A Role In Tri	-1	SNV	HGNC	HGNC:293	YES	NM_0013[illegible]
May Play A Role In Tri	-1	SNV	HGNC	HGNC:293	YES	NM_0013[illegible]
May Play A Role In Tri	-1	SNV	HGNC	HGNC:293	YES	NM_0013[illegible]
May Play A Role In Tri	-1	SNV	HGNC	HGNC:293	YES	NM_0013[illegible]
Lon Peptidase 1, Mito	-1	SNV	HGNC	HGNC:947	YES	NM_0047[illegible]
Regulatory Factor X2	-1	SNV	HGNC	HGNC:998	YES	NM_00063
C-Type Lectin Domain	1	SNV	HGNC	HGNC:135	YES	NM_0142[illegible]
Ppan-P2Ry11 Readthr	1	SNV	HGNC	HGNC:922	YES	NM_02023
Yip1 Domain Family M	-1	SNV	HGNC	HGNC:284	YES	NM_00132
Acid Phosphatase 5, T	-1	SNV	HGNC	HGNC:124	YES	NM_00161
Epidermal Growth Fa	-1	SNV	HGNC	HGNC:246	YES	NM_0012[illegible]
C3 And Pzp Like Alpha	-1	SNV	HGNC	HGNC:232	YES	NM_0156[illegible]
Modulates The O-Gly	1	SNV	HGNC	HGNC:132	YES	NM_0121[illegible]
Cartilage Oligomeric	-1	SNV	HGNC	HGNC:222	YES	NM_0000[illegible]
Copi Coat Complex Su	-1	SNV	HGNC	HGNC:223	YES	NM_0072[illegible]
Surp And G-Patch Dor	-1	SNV	HGNC	HGNC:186	YES	NM_17223
Zinc Finger Protein 10	1	SNV	HGNC	HGNC:128	YES	NM_0332[illegible]
Zinc Finger Protein 67	-1	SNV	HGNC	HGNC:204	YES	NM_0010[illegible]
Zinc Finger Protein 72	1	SNV	HGNC	HGNC:322	YES	NM_00134
Nphs1 Adhesion Mole	-1	SNV	HGNC	HGNC:790	YES	NM_00464
Ccnp (From Genesym	-1	SNV	HGNC	HGNC:258	YES	NM_02487
Clk4 Associating Serin	1	SNV	HGNC	HGNC:177	YES	NM_0070[illegible]
Dishevelled Binding A	-1	SNV	HGNC	HGNC:307	YES	NM_1450[illegible]
Solute Carrier Family	-1	SNV	HGNC	HGNC:109	YES	NM_00562
Chorionic Gonadotro	-1	SNV	HGNC	HGNC:188	YES	NM_00073

Chorionic Gonadotrop	1	SNV	HGNC	HGNC:167	YES	NM_03337
Chorionic Gonadotrop	-1	SNV	HGNC	HGNC:167	YES	NM_03337
Solute Carrier Family	-1	SNV	HGNC	HGNC:136	YES	NM_01403
Zinc Finger Protein 84	1	SNV	HGNC	HGNC:251	YES	NM_13837
Scavenger Receptor C	1	SNV	HGNC	HGNC:266	YES	NM_00114
Peroxidasin (Pxdn), M	-1	SNV	HGNC	HGNC:149	YES	NM_01229
Rad51 Associated Pro	-1	SNV	HGNC	HGNC:344	YES	NM_00109
Carbamoyl-Phosphate	1	SNV	HGNC	HGNC:142	YES	NM_00434
Baculoviral Iap Repea	1	SNV	HGNC	HGNC:135	YES	NM_01625
Promotes Matrix Asse	1	SNV	HGNC	HGNC:126	YES	NM_05327
C1Galt1 Specific Chap	-1	SNV	HGNC	HGNC:516	YES	NM_00110
Leucine Rich Pentatric	-1	SNV	HGNC	HGNC:157	YES	NM_13325
Chromosome 2 Open	1	SNV	HGNC	HGNC:268	YES	NM_00110
Ubiquitin Specific Pep	-1	SNV	HGNC	HGNC:200	YES	NM_01470
Prenylcysteine Oxidas	1	SNV	HGNC	HGNC:205	YES	NM_01629
Aup1 Lipid Droplet Re	-1	SNV	HGNC	HGNC:891	YES	NM_18157
Gc-Rich Sequence Dna	-1	SNV	HGNC	HGNC:131	YES	NM_00320
Dynein Axonemal Hea	1	SNV	HGNC	HGNC:295	YES	NM_00137
Threonine Synthase L	1	SNV	HGNC	HGNC:256	YES	NM_01827
Tripartite Motif Conta	1	SNV	HGNC	HGNC:190	YES	NM_13880
Event	-1	SNV	HGNC	HGNC:329	YES	NM_00139
Cyclic Nucleotide Gat	1	SNV	HGNC	HGNC:215	YES	NM_00129
Pote Ankyrin Domain	-1	SNV	HGNC	HGNC:370	YES	NM_00127
Pote Ankyrin Domain	-1	SNV	HGNC	HGNC:370	YES	NM_00127
Pote Ankyrin Domain	-1	SNV	HGNC	HGNC:370	YES	NM_00127
Ly6/Plaur Domain Co	1	SNV	HGNC	HGNC:449	YES	NM_00150
Rab3 Gtpase Activatir	1	SNV	HGNC	HGNC:170	YES	NM_01223
Nebulin (Neb), Transc	-1	SNV	HGNC	HGNC:772	YES	NM_00116
Polypeptide N-Acetylg	1	SNV	HGNC	HGNC:412	YES	NM_01456
Tetratricopeptide Rep	-1	SNV	HGNC	HGNC:256	YES	NM_02475
Ceramide Synthase 6	1	SNV	HGNC	HGNC:238	YES	NM_20346
Tetratricopeptide Rep	-1	SNV	HGNC	HGNC:258	YES	NM_15227
Titin (Ttn), Transcript	-1	SNV	HGNC	HGNC:124	YES	NM_00126
Fibrous Sheath Intera	1	SNV	HGNC	HGNC:216	YES	NM_17365
Eukaryotic Translatior	1	SNV	HGNC	HGNC:320	YES	NM_00195
Dystrotelin (Dytn), M	-1	SNV	HGNC	HGNC:232	YES	NM_00109
Crystallin Gamma D (	-1	SNV	HGNC	HGNC:241	YES	NM_00689
Bcs1 Homolog, Ubiqu	1	SNV	HGNC	HGNC:102	YES	NM_00107
Serine/Threonine Kin	1	SNV	HGNC	HGNC:191	YES	NM_05290
The Sequence Shown	1	SNV	HGNC	HGNC:516	YES	NM_00135
Syntaphilin (Snph), Tr	1	SNV	HGNC	HGNC:159	YES	NM_00131
Adrenoceptor Alpha 1	-1	SNV	HGNC	HGNC:280	YES	NM_00067
Kinesin Family Memb	-1	SNV	HGNC	HGNC:158	YES	NM_02470
Human Saliva Appear	-1	SNV	HGNC	HGNC:247	YES	NM_00189
Matrix Metallopeptid	1	SNV	HGNC	HGNC:717	YES	NM_00669
Actin Related Protein	1	SNV	HGNC	HGNC:146	YES	NM_02485
Belongs To The Polya	1	SNV	HGNC	HGNC:157	YES	NM_00137
Semenogelin 2 (Semg	1	SNV	HGNC	HGNC:107	YES	NM_00300
Zinc Finger Swim-Typ	1	SNV	HGNC	HGNC:161	YES	NM_08060
Event	-1	SNV	HGNC	HGNC:292	YES	NM_02103

Mitochondrial Riboso	1	SNV	HGNC	HGNC:162	YES	NM_01566
Rbbp8 N-Terminal Lik	-1	SNV	HGNC	HGNC:161	YES	NM_08083
Rna Binding Motif Prc	1	SNV	HGNC	HGNC:989	YES	NM_14477
Modulates Transcripti	-1	SNV	HGNC	HGNC:800	YES	NM_00348
Ubiquitin Specific Pep	1	SNV	HGNC	HGNC:126	YES	NM_00644
Claudin 8 (Cldn8), Mr	-1	SNV	HGNC	HGNC:205	YES	NM_19932
Urb1 Ribosome Bioge	-1	SNV	HGNC	HGNC:173	YES	NM_01482
Interferon Gamma Re	1	SNV	HGNC	HGNC:544	YES	NM_00553
Potassium Voltage-Ga	-1	SNV	HGNC	HGNC:624	YES	NM_00021
Dop1 Leucine Zipper l	1	SNV	HGNC	HGNC:129	YES	NM_00132
Chromatin Assembly I	1	SNV	HGNC	HGNC:191	YES	NM_00544
Salt Inducible Kinase :	-1	SNV	HGNC	HGNC:111	YES	NM_17335
Pwp2 Small Subunit P	1	SNV	HGNC	HGNC:971	YES	NM_00504
Glutamine Amidotran	1	SNV	HGNC	HGNC:127	YES	NM_00464
Inducible T Cell Costir	-1	SNV	HGNC	HGNC:170	YES	NM_01525
Dnmt3L Antisense Rn	-1	SNV	HGNC	HGNC:298	YES	NM_17586
Transmembrane Prot	1	SNV	HGNC	HGNC:336	YES	NM_00124
Rims Binding Protein :	-1	SNV	HGNC	HGNC:293	YES	NM_01567
Proline Dehydrogenas	-1	SNV	HGNC	HGNC:945	YES	NM_01633
T-Box Transcription Fa	1	SNV	HGNC	HGNC:115	YES	NM_00137
Transport And Golgi C	1	SNV	HGNC	HGNC:254	YES	NM_15290
Scavenger Receptor C	-1	SNV	HGNC	HGNC:198	YES	NM_18289
Aspartate Rich 1 (Dric	-1	SNV	HGNC	HGNC:280	YES	NM_01644
Specc1L-Adora2A Rea	1	SNV	HGNC	HGNC:290	YES	NM_01533
Small G Protein Signal	1	SNV	HGNC	HGNC:294	YES	NM_00109
Event	1	SNV	HGNC	HGNC:293	YES	NM_00114
Chromosome 22 Oper	-1	SNV	HGNC	HGNC:269	YES	NM_01537
Solute Carrier Family	-1	SNV	HGNC	HGNC:110	YES	NM_01422
Target Of Myb1 Mem	1	SNV	HGNC	HGNC:119	YES	NM_00548
Myocardin Related Tr	-1	SNV	HGNC	HGNC:143	YES	NM_02083
Tnf Receptor Superfa	-1	SNV	HGNC	HGNC:177	YES	NM_05294
Rho Gtpase Activating	1	SNV	HGNC	HGNC:677	YES	NM_18133
Family With Sequence	1	SNV	HGNC	HGNC:131	YES	NM_01791
Cadherin Egf Lag Seve	-1	SNV	HGNC	HGNC:185	YES	NM_00137
Copine Family Memb	1	SNV	HGNC	HGNC:243	YES	NM_15363
Villin Like (Vill), Trans	1	SNV	HGNC	HGNC:309	YES	NM_01587
Xylulokinase (Xylb), Tr	1	SNV	HGNC	HGNC:128	YES	NM_00510
Sodium Voltage-Gate	-1	SNV	HGNC	HGNC:105	YES	NM_00033
Trafficking Kinesin Prc	1	SNV	HGNC	HGNC:299	YES	NM_00104
May Be Involved In Tr	1	SNV	HGNC	HGNC:237	YES	NM_00113
Als2 C-Terminal Like (	-1	SNV	HGNC	HGNC:206	YES	NM_14712
Solute Carrier Family	-1	SNV	HGNC	HGNC:144	YES	NM_02291
Binding To Cells Via A	-1	SNV	HGNC	HGNC:648	YES	NM_00229
Macrophage Stimulat	-1	SNV	HGNC	HGNC:738	YES	NM_00244
Ras Association Doma	-1	SNV	HGNC	HGNC:988	YES	NM_00718
Stabilin 1 (Stab1), Mr	1	SNV	HGNC	HGNC:186	YES	NM_01513
Stabilin 1 (Stab1), Mr	1	SNV	HGNC	HGNC:186	YES	NM_01513
G Protein Nucleolar 3	1	SNV	HGNC	HGNC:299	YES	NM_01436
Dynein Axonemal Hea	-1	SNV	HGNC	HGNC:294	YES	NM_00136
Leucine Rich Repeats	-1	SNV	HGNC	HGNC:173	YES	NM_01554

Casein Kinase 2 Subur	1	SNV	HGNC	HGNC:536	YES	NM_00136
Muscle Contraction (E	-1	SNV	HGNC	HGNC:310	YES	NM_01498
Nucleolus And Neural	-1	SNV	HGNC	HGNC:244	YES	NM_01541
Golgin B1 (Golgb1), Tr	-1	SNV	HGNC	HGNC:442	YES	NM_00136
Golgin B1 (Golgb1), Tr	-1	SNV	HGNC	HGNC:442	YES	NM_00136
Cilia And Flagella Assc	-1	SNV	HGNC	HGNC:292	YES	NM_00139
Tripartite Motif Conta	1	SNV	HGNC	HGNC:190	YES	NM_15261
Divergent Protein Kin	1	SNV	HGNC	HGNC:284	YES	NM_17355
Preferential Release C	1	SNV	HGNC	HGNC:229	YES	NM_00187
Retinoic Acid Recepto	-1	SNV	HGNC	HGNC:986	YES	NM_20696
Lipase H (Liph), Mrna.	-1	SNV	HGNC	HGNC:184	YES	NM_13924
Mucin 4, Cell Surface	-1	SNV	HGNC	HGNC:751	YES	NM_01840
Mucin 4, Cell Surface	-1	SNV	HGNC	HGNC:751	YES	NM_01840
Mucin 4, Cell Surface	-1	SNV	HGNC	HGNC:751	YES	NM_01840
Mucin 4, Cell Surface	-1	SNV	HGNC	HGNC:751	YES	NM_01840
Mucin 4, Cell Surface	-1	SNV	HGNC	HGNC:751	YES	NM_01840
Mucin 4, Cell Surface	-1	SNV	HGNC	HGNC:751	YES	NM_01840
Mucin 4, Cell Surface	-1	SNV	HGNC	HGNC:751	YES	NM_01840
Mucin 4, Cell Surface	-1	SNV	HGNC	HGNC:751	YES	NM_01840
Mucin 4, Cell Surface	-1	SNV	HGNC	HGNC:751	YES	NM_01840
Cyclin G Associated Ki	-1	SNV	HGNC	HGNC:411	YES	NM_00525
Inhibits Signal Transdu	1	SNV	HGNC	HGNC:999	YES	NM_00139
Mannosidase Alpha C	1	SNV	HGNC	HGNC:296	YES	NM_01527
Htra Serine Peptidase	1	SNV	HGNC	HGNC:304	YES	NM_05304
Trna Methyltransfera:	1	SNV	HGNC	HGNC:266	YES	NM_15254
Arfgap With Rhogap D	-1	SNV	HGNC	HGNC:169	YES	NM_01523
Corin, Serine Peptidas	-1	SNV	HGNC	HGNC:190	YES	NM_00658
Cell Membrane| Mult	1	SNV	HGNC	HGNC:209	YES	NM_00138
Joining Chain Of Mult	-1	SNV	HGNC	HGNC:571	YES	NM_14464
Uso1 Vesicle Transpo	1	SNV	HGNC	HGNC:309	YES	NM_00371
Fraser Extracellular M	1	SNV	HGNC	HGNC:191	YES	NM_02507
Wd Repeat And Fyve	-1	SNV	HGNC	HGNC:207	YES	NM_01499
Phospholipase A2 Grc	-1	SNV	HGNC	HGNC:185	YES	NM_03082
Synaptopodin 2 (Synp	1	SNV	HGNC	HGNC:177	YES	NM_13347
Annexin A5 (Anxa5), M	-1	SNV	HGNC	HGNC:543	YES	NM_00115
Zinc Finger Protein 33	1	SNV	HGNC	HGNC:154	YES	NM_01448
Adp Ribosylation Fact	1	SNV	HGNC	HGNC:214	YES	NM_00102
Glycine Receptor Beta	1	SNV	HGNC	HGNC:432	YES	NM_00082
Vascular Endothelial C	-1	SNV	HGNC	HGNC:126	YES	NM_00542
Interferon Regulatory	-1	SNV	HGNC	HGNC:611	YES	NM_00219
Pr/Set Domain 9 (Prdı	1	SNV	HGNC	HGNC:139	YES	NM_02022
Pdz Domain Containir	1	SNV	HGNC	HGNC:184	YES	NM_17814
Alanine--Glyoxylate A	-1	SNV	HGNC	HGNC:144	YES	NM_03190
Nipbl Cohesin Loading	1	SNV	HGNC	HGNC:288	YES	NM_13343
Ciliogenesis And Plana	-1	SNV	HGNC	HGNC:258	YES	NM_00138
Nucleoporin 155 (Nup	-1	SNV	HGNC	HGNC:806	YES	NM_15348
Pentatricopeptide Re	1	SNV	HGNC	HGNC:257	YES	NM_02475
Ankyrin Repeat Doma	-1	SNV	HGNC	HGNC:337	YES	NM_00100
G Protein-Coupled Re	1	SNV	HGNC	HGNC:236	YES	NM_19924
Chromodomain Helica	-1	SNV	HGNC	HGNC:191	YES	NM_00127

Erythrocyte Membrar	-1	SNV	HGNC	HGNC:132	YES	NM_02214
Erythrocyte Membrar	-1	SNV	HGNC	HGNC:132	YES	NM_02214
Centrosomal Protein :	-1	SNV	HGNC	HGNC:266	YES	NM_00137
Fibrillins Are Structura	-1	SNV	HGNC	HGNC:360	YES	NM_00199
Rad50 Double Strand	1	SNV	HGNC	HGNC:981	YES	NM_00573
Af4/Fmr2 Family Men	-1	SNV	HGNC	HGNC:178	YES	NM_01442
Calcium Modulating L	1	SNV	HGNC	HGNC:147	YES	NM_00174
Polycystin 2 Like 2, Tr	1	SNV	HGNC	HGNC:901	YES	NM_00130
Protocadherin Beta 7	1	SNV	HGNC	HGNC:869	YES	NM_01894
Protocadherin Beta 1:	1	SNV	HGNC	HGNC:868	YES	NM_01893
Protocadherin Gamm	1	SNV	HGNC	HGNC:870	YES	NM_01891
Protocadherin Gamm	1	SNV	HGNC	HGNC:871	YES	NM_00373
Protocadherin Gamm	1	SNV	HGNC	HGNC:871	YES	NM_00373
Fbxo38-Dt (From Gen	1	SNV	HGNC	HGNC:154	YES	NM_00684
Adrenoceptor Beta 2	1	SNV	HGNC	HGNC:286	YES	NM_00002
Gamma-Aminobutyri	1	SNV	HGNC	HGNC:408	YES	NM_00081
Dual Specificity Phosp	-1	SNV	HGNC	HGNC:306	YES	NM_00441
Cytoplasmic Polyader	1	SNV	HGNC	HGNC:217	YES	NM_03062
Family With Sequence	-1	SNV	HGNC	HGNC:255	YES	NM_00119
5-Phosphohydroxy-L-l	-1	SNV	HGNC	HGNC:282	YES	NM_15337
Cancer Antigen 1 (Cag	-1	SNV	HGNC	HGNC:216	YES	NM_00117
Glial Cells Missing Tra	-1	SNV	HGNC	HGNC:419	YES	NM_00475
Nucleoporin 153 (Nup	-1	SNV	HGNC	HGNC:806	YES	NM_00512
Cdk5 Regulatory Subu	1	SNV	HGNC	HGNC:210	YES	NM_01777
Mdc1 Antisense Rna 1	-1	SNV	HGNC	HGNC:211	YES	NM_01464
Membrane| Single-Pa	1	SNV	HGNC	HGNC:397	YES	NM_00139
Major Histocompatibi	-1	SNV	HGNC	HGNC:493	YES	NM_00211
Major Histocompatibi	-1	SNV	HGNC	HGNC:493	YES	NM_00211
Major Histocompatibi	-1	SNV	HGNC	HGNC:493	YES	NM_00211
Major Histocompatibi	-1	SNV	HGNC	HGNC:493	YES	NM_00211
Major Histocompatibi	-1	SNV	HGNC	HGNC:493	YES	NM_00211
Major Histocompatibi	-1	SNV	HGNC	HGNC:493	YES	NM_00211
Major Histocompatibi	-1	SNV	HGNC	HGNC:493	YES	NM_00211
Major Histocompatibi	-1	SNV	HGNC	HGNC:493	YES	NM_00211
Major Histocompatibi	-1	SNV	HGNC	HGNC:493	YES	NM_00211
Major Histocompatibi	-1	SNV	HGNC	HGNC:493	YES	NM_00211
Major Histocompatibi	-1	SNV	HGNC	HGNC:493	YES	NM_00211
Major Histocompatibi	-1	SNV	HGNC	HGNC:493	YES	NM_00211
Major Histocompatibi	-1	SNV	HGNC	HGNC:493	YES	NM_00211
Major Histocompatibi	-1	SNV	HGNC	HGNC:493	YES	NM_00211
Major Histocompatibi	-1	SNV	HGNC	HGNC:493	YES	NM_00211
Major Histocompatibi	-1	SNV	HGNC	HGNC:493	YES	NM_00211
Major Histocompatibi	-1	SNV	HGNC	HGNC:493	YES	NM_00211
Major Histocompatibi	-1	SNV	HGNC	HGNC:493	YES	NM_00211
Major Histocompatibi	-1	SNV	HGNC	HGNC:494	YES	NM_00212
Receptor For Inositol	1	SNV	HGNC	HGNC:618	YES	NM_00222
Glutamate Metabotrc	-1	SNV	HGNC	HGNC:459	YES	NM_00084
Bromodomain And Pł	1	SNV	HGNC	HGNC:142	YES	NM_01569
Exportin 5 (Xpo5), Tra	-1	SNV	HGNC	HGNC:176	YES	NM_02075
Dna Polymerase Eta (l	1	SNV	HGNC	HGNC:918	YES	NM_00650

Bone Morphogenetic	-1	SNV	HGNC	HGNC:107	YES	NM_02107
Dystonin (Dst), Transc	-1	SNV	HGNC	HGNC:109	YES	NM_00137
Cannabinoid Recepto	-1	SNV	HGNC	HGNC:215	YES	NM_01608
Afg1 Like Atpase (Afg	1	SNV	HGNC	HGNC:164	YES	NM_14531
Myristoylated Alanine	1	SNV	HGNC	HGNC:675	YES	NM_00235
Laminin Subunit Alpha	1	SNV	HGNC	HGNC:648	YES	NM_00042
Interacts With Fcgr1A	-1	SNV	HGNC	HGNC:337	YES	NM_00143
Ldl Receptor Related	-1	SNV	HGNC	HGNC:169	YES	NM_03283
Fibronectin Type Iii Do	1	SNV	HGNC	HGNC:211	YES	NM_03253
Dishevelled Binding A	-1	SNV	HGNC	HGNC:212	YES	NM_21446
Fam20C Golgi Associa	1	SNV	HGNC	HGNC:221	YES	NM_02022
Transmembrane Prot	-1	SNV	HGNC	HGNC:287	YES	NM_00109
Adaptor Related Prot	1	SNV	HGNC	HGNC:221	YES	NM_01485
Aryl Hydrocarbon Rec	1	SNV	HGNC	HGNC:348	YES	NM_00162
Dynein Axonemal Hea	1	SNV	HGNC	HGNC:294	YES	NM_00127
Inmt-Mindy4 Readthr	1	SNV	HGNC	HGNC:606	YES	NM_00677
Avl9 Cell Migration As	1	SNV	HGNC	HGNC:289	YES	NM_01506
Npc1 Like Intracellula	-1	SNV	HGNC	HGNC:789	YES	NM_00110
Oxoglutarate Dehydro	1	SNV	HGNC	HGNC:812	YES	NM_00254
Nac Alpha Domain Co	-1	SNV	HGNC	HGNC:221	YES	NM_00114
Tensin 3 (Tns3), Mrna	-1	SNV	HGNC	HGNC:216	YES	NM_02274
Chromosome 7 Open	1	SNV	HGNC	HGNC:222	YES	NM_00110
Cordon-Bleu Wh2 Rep	-1	SNV	HGNC	HGNC:221	YES	NM_01519
Cordon-Bleu Wh2 Rep	-1	SNV	HGNC	HGNC:221	YES	NM_01519
Zinc Finger Protein 47	-1	SNV	HGNC	HGNC:232	YES	NM_00137
Fkbp Prolyl Isomerase	1	SNV	HGNC	HGNC:372	YES	NM_00360
Bromodomain Adjace	-1	SNV	HGNC	HGNC:961	YES	NM_03240
Coiled-Coil Domain Co	1	SNV	HGNC	HGNC:292	YES	NM_02087
Atp5Mf-Ptcd1 Readth	-1	SNV	HGNC	HGNC:221	YES	NM_01554
Cytochrome P450 Far	1	SNV	HGNC	HGNC:174	YES	NM_05709
Neuronal Tyrosine Ph	1	SNV	HGNC	HGNC:220	YES	NM_17356
Mucin 3A, Cell Surface	1	SNV	HGNC	HGNC:751	YES	NM_00596
Procollagen-Lysine,2-	-1	SNV	HGNC	HGNC:908	YES	NM_00108
Solute Carrier Family	1	SNV	HGNC	HGNC:881	YES	NM_00044
Interferon Related De	1	SNV	HGNC	HGNC:545	YES	NM_00155
Calcium Dependent Se	-1	SNV	HGNC	HGNC:160	YES	NM_01795
Aldo-Keto Reductase	1	SNV	HGNC	HGNC:388	YES	NM_00598
Nobox Oogenesis Hor	-1	SNV	HGNC	HGNC:224	YES	NM_00108
Zinc Finger Protein 78	-1	SNV	HGNC	HGNC:218	YES	NM_15241
Leucine Zipper Tumor	-1	SNV	HGNC	HGNC:138	YES	NM_02102
Rna Polymerase Iii Su	1	SNV	HGNC	HGNC:108	YES	NM_00172
Kinesin Family Memb	-1	SNV	HGNC	HGNC:144	YES	NM_01525
Glutathione-Disulfide	-1	SNV	HGNC	HGNC:462	YES	NM_00063
Rab11 Family Interact	-1	SNV	HGNC	HGNC:302	YES	NM_00100
Ash2 Like, Histone Lys	1	SNV	HGNC	HGNC:744	YES	NM_00467
Ring Finger Protein 17	-1	SNV	HGNC	HGNC:253	YES	NM_03095
Atpase H+ Transporti	-1	SNV	HGNC	HGNC:183	YES	NM_01594
Lysophospholipase 1	-1	SNV	HGNC	HGNC:673	YES	NM_00633
Alcohol Dehydrogena	1	SNV	HGNC	HGNC:163	YES	NM_14465
Nudc Domain Contain	-1	SNV	HGNC	HGNC:243	YES	NM_03286

N-Myc Downstream R	-1	SNV	HGNC	HGNC:767	YES	NM_0060
Zinc Finger Protein 69	1	SNV	HGNC	HGNC:258	YES	NM_0308
Zinc Finger Ccch-Type	-1	SNV	HGNC	HGNC:289	YES	NM_01511
Wd Repeat Domain 9	1	SNV	HGNC	HGNC:269	YES	NM_00131
Bop1 Ribosomal Bioge	-1	SNV	HGNC	HGNC:155	YES	NM_0152
Solute Carrier Family	-1	SNV	HGNC	HGNC:171	YES	NM_13084
Solute Carrier Family	-1	SNV	HGNC	HGNC:171	YES	NM_13084
Wash Complex Subun	-1	SNV	HGNC	HGNC:243	YES	NM_00137
Kinesin Family Memb	-1	SNV	HGNC	HGNC:199	YES	NM_19431
Rho Guanine Nucleoti	-1	SNV	HGNC	HGNC:259	YES	NM_03281
Transient Receptor Po	-1	SNV	HGNC	HGNC:179	YES	NM_0176
Prune Homolog 2 Wit	-1	SNV	HGNC	HGNC:252	YES	NM_01522
The Sequence Shown	1	SNV	HGNC	HGNC:145	YES	NM_00664
Polypeptide N-Acetyl	1	SNV	HGNC	HGNC:198	YES	NM_02464
Olfactory Receptor Fa	1	SNV	HGNC	HGNC:821	YES	NM_0010
Exosome Component	1	SNV	HGNC	HGNC:170	YES	NM_0142
Mitochondrion Inner	1	SNV	HGNC	HGNC:741	YES	
Component Of The Ul	1	SNV	HGNC	HGNC:742	YES	
Mage Family Member	1	SNV	HGNC	HGNC:237	YES	NM_17352
Dual Specificity Phosp	1	SNV	HGNC	HGNC:204	YES	NM_02207
G Antigen 12G (Gage1	1	SNV	HGNC	HGNC:319	YES	NM_0010
Bone Morphogenetic	1	SNV	HGNC	HGNC:106	YES	NM_00544
Zinc Finger X-Linked D	1	SNV	HGNC	HGNC:131	YES	NM_0071
Zinc Finger X-Linked D	-1	SNV	HGNC	HGNC:131	YES	NM_0071
Zinc Finger X-Linked D	-1	SNV	HGNC	HGNC:131	YES	NM_0071
Zinc Finger X-Linked D	-1	SNV	HGNC	HGNC:131	YES	NM_0071
Germ Cell Nuclear Aci	1	SNV	HGNC	HGNC:158	YES	NM_0529
Transcription Elongati	-1	SNV	HGNC	HGNC:286	YES	NM_1533
Adhesion G Protein-C	1	SNV	HGNC	HGNC:189	YES	NM_1538
Myotubularin Related	1	SNV	HGNC	HGNC:744	YES	NM_0013
Fc Receptor Like 4 (Fc	-1	SNV	HGNC	HGNC:185	YES	NM_0312
Poly(Adp-Ribose) Poly	-1	SNV	HGNC	HGNC:270	YES	NM_00161
Nucleoporin 133 (Nup	-1	SNV	HGNC	HGNC:180	YES	NM_0182
Ercc Excision Repair 6	-1	SNV	HGNC	HGNC:343	YES	NM_00012
Carboxypeptidase X,	-1	SNV	HGNC	HGNC:269	YES	NM_19814
N-Acetyltransferase 1	1	SNV	HGNC	HGNC:298	YES	NM_0246
Zona Pellucida Glycop	1	SNV	HGNC	HGNC:131	YES	NM_20734
Endoplasmic Reticulu	-1	SNV	HGNC	HGNC:169	YES	NM_0332
Dna Topoisomerase Ii	-1	SNV	HGNC	HGNC:119	YES	NM_00461
Chorionic Gonadotro	1	SNV	HGNC	HGNC:167	YES	NM_03337
Solute Carrier Family	-1	SNV	HGNC	HGNC:109	YES	NM_0122
Ankyrin Repeat Doma	1	SNV	HGNC	HGNC:240	YES	NM_0013
Erb-B2 Receptor Tyro	-1	SNV	HGNC	HGNC:343	YES	NM_0052
Ribosomal Rna Proces	1	SNV	HGNC	HGNC:294	YES	NM_0145
Ligand Of Numb-Prot	-1	SNV	HGNC	HGNC:665	YES	NM_00112
Major Histocompatibi	-1	SNV	HGNC	HGNC:493	YES	NM_00211
Drebrin Like (Dbnl), T	1	SNV	HGNC	HGNC:269	YES	NM_00101
Probably Involved In	1	SNV	HGNC	HGNC:240	YES	NM_20692
Potassium Inwardly R	1	SNV	HGNC	HGNC:390	YES	NM_0011
Pote Ankyrin Domain	1	SNV	HGNC	HGNC:338	YES	NM_0010

Wd Repeat Domain 2	-1	SNV	HGNC	HGNC:212	YES	NM_18255
Mage Family Member	1	deletion	HGNC	HGNC:679	YES	NM_00498
	-1	SNV			YES	
Long Intergenic Non-F	1	deletion	HGNC	HGNC:270	YES	
6-Phosphofructo-2-Ki	1	SNV	HGNC	HGNC:887	YES	NM_00621
Zinc Finger Protein 21	1	SNV	HGNC	HGNC:130	YES	NM_01325
Solute Carrier Family	1	SNV	HGNC	HGNC:206	YES	NM_00137
Sbds Ribosome Matur	-1	SNV	HGNC	HGNC:194	YES	NM_01603
Crystallin Zeta Like 2,	-1	SNV	HGNC	HGNC:521	YES	
Long Intergenic Non-F	1	SNV	HGNC	HGNC:526	YES	
Peptidylprolyl Isomera	-1	SNV	HGNC	HGNC:339	YES	NM_00116
Nbpf Member 14 (Nb	-1	SNV	HGNC	HGNC:252	YES	NM_00139
Tripartite Motif Conta	1	SNV	HGNC	HGNC:241	YES	NM_01543
Growth Differentiatio	1	SNV	HGNC	HGNC:421	YES	NM_00496
Sphingomyelin Phosp	1	SNV	HGNC	HGNC:111	YES	NM_00054
Atp/Gtp Binding Prote	1	SNV	HGNC	HGNC:265	YES	NM_00138
Ankrd40 C-Terminal L	-1	SNV	HGNC	HGNC:260	YES	NM_00135
Zinc Finger Protein 57	1	SNV	HGNC	HGNC:131	YES	NM_17348
Cytochrome P450 Far	1	SNV	HGNC	HGNC:268	YES	NM_17348
Crystallin Gamma D (	-1	SNV	HGNC	HGNC:241	YES	NM_00689
May Interact With Ak	1	SNV	HGNC	HGNC:240	YES	NM_03191
Xg Glycoprotein (Xg B	1	SNV	HGNC	HGNC:128	YES	NM_00114
Cystathionine Beta-Sy	-1	insertion	HGNC	HGNC:155	YES	NM_00007

_PLUS_CLI	TSL	CCDS	ENSP	SWISSPROT	TREMBL	UNIPARC	SOURCE	ENE_PHEN
0.4	1	CCDS3173	ENSP0000	P54259.192		UPI000006F554		1
0.4	1	CCDS3173	ENSP0000	P54259.192		UPI000006F554		1
2492.3	1	CCDS4229	ENSP0000	P21359.242		UPI000012FFAE		1
7.4	1	CCDS1160	ENSP0000	Q9NXB0.146		UPI00002010BB		1
6.4	2	CCDS1208	ENSP0000	Q96MG2.133		UPI000006EA5D		
4828.1	2		ENSP00000490491		A0A6Q8N	UPI00125CB504		1
4828.1	2		ENSP00000490491		A0A6Q8N	UPI00125CB504		1
1917.2	2	CCDS5530	ENSP0000	Q6ZU69.107		UPI00017EE92B		
6.5		CCDS1437	ENSP0000	P98168.173		UPI000013C494		
6.5		CCDS1437	ENSP0000	P98168.173		UPI000013C494		
4.4	1	CCDS3066	ENSP0000	O75056.177		UPI000045619B		1
1480.3		CCDS4159	ENSP0000	Q7O1N2.112		UPI0000E592E5		
8.4	5	CCDS4162	ENSP0000	Q8N9C0.146		UPI0001D3B05B		
6.3		CCDS4168	ENSP0000	O75690.135		UPI000013ECDA		
4.3	1	CCDS8336	ENSP0000	Q2TBE0.129		UPI00005A81B4		
9.3	1	CCDS9094	ENSP0000	P14625.24	V9HWP2.6	UPI0000129FBA		
0368.2			ENSP00000496978		A0A3B3IR	UPI0000DD81E2		
9206.4	5	CCDS8192	ENSP0000	Q02078.2	A0A0S2Z4	UPI000002ADD2		1
5.5	1	CCDS1052	ENSP0000	O60437.188		UPI00001AE832		
7624.2			ENSP00000456500		H3BS19.6	UPI0004620D6A		1
1.3	2	CCDS3339	ENSP0000	P19338.231		UPI0000456F25		
3891.3	1	CCDS3360	ENSP0000	Q96RN5.189		UPI00001313C4		
3.4	1	CCDS4879	ENSP0000	Q16626.148		UPI000007291B		
0.5	5	CCDS3497	ENSP0000	P51531.220		UPI00001AE8EB		1
2371.3	5	CCDS7620	ENSP00000463957		A0A075B7	UPI00051EF319		
3.4	1	CCDS1503	ENSP0000	Q15172.195		UPI0000124E90		
9.6	1	CCDS4189	ENSP0000	Q9UI36.186		UPI000007308B		
4.6	1	CCDS4201	ENSP0000	P37108.202		UPI0000001C32		
8.3		CCDS4295	ENSP0000	P60371.132		UPI000021C43D		
5.5	1	CCDS4666	ENSP0000	Q96GP6.165		UPI000004D28D		1
0.4	1	CCDS3951	ENSP0000	Q9NP92.166		UPI000013C944		
3.4	1	CCDS4140	ENSP0000	P20700.218		UPI000013D170		1
0.2	5	CCDS7826	ENSP0000	Q02505.154		UPI000455B941		
4360.2			ENSP00000508276		A0A804HL	UPI000387B44C		
4.4	5	CCDS3057	ENSP0000	O60308.156		UPI0000139AA8		1
3.5	1	CCDS144.1	ENSP0000	P28908.207		UPI0000000971		
8376.3	1	CCDS4405	ENSP0000	P0DUQ1.1&P0DUQ2.		UPI0000199BA7		
7.3	1	CCDS180.1	ENSP0000	Q9UM07.189		UPI000013DFF1		
6265.2	5	CCDS4407	ENSP0000	Q5TF58.114		UPI0000EE7032		
9500.3	5		ENSP0000	Q5TIE3.112		UPI0000EE708C		
1906.1	1		ENSP0000	O43432.193		UPI0005D02ED3		
4.6	1	CCDS262.1	ENSP0000	Q02161.1	A0A1B1R0	UPI0000246EA2		1
1.3	1	CCDS4128	ENSP0000	Q9NZV5.169		UPI00003761B2		1
8.5	1	CCDS3067	ENSP0000	Q13643.185		UPI0000070486		
2.4	1	CCDS466.2	ENSP0000	Q8IXN7.158		UPI0000160ABF		
8189.1	5		ENSP0000	Q96MR6.146		UPI0001D89705		
5.2	1	CCDS536.1	ENSP0000	Q8NFW5.155		UPI0000070B63		
2.3	1	CCDS606.1	ENSP0000	P07357.215		UPI0000127C5A		1
5.4			ENSP0000	Q5VU97.131		UPI00015B3BA1		

20.4	1	CCDS687.2	ENSP00000	Q53G44.112		UPI0000374C64	
38.3	1	CCDS698.1	ENSP00000	Q01459.167		UPI00001293CB	
72.5	1	CCDS702.1	ENSP00000	Q8IWG1.1	A0A140VJ	UPI00000744F1	
92.3	1	CCDS764.1	ENSP00000	Q6UVJ0.139		UPI00001BDF30	1
56404.2	5	CCDS5801	ENSP00000	Q68D51.127		UPI000048AF71	
56404.2	5	CCDS5801	ENSP00000	Q68D51.127		UPI000048AF71	
94.4	1	CCDS892.1	ENSP00000	Q9UNY4.181		UPI000013CCE7	
08.4	1	CCDS908.1	ENSP00000	Q04721.238		UPI000013CF1D	1
29984.2	5	CCDS8136	ENSP00000	P0DJJ0.63		UPI0002656649	
37385.2	1	CCDS7285	ENSP00000	H0Y354.59		UPI0002742DFA	
97.4	1	CCDS949.1	ENSP00000	Q8N365.124		UPI000000DBBF	
32.6	5	CCDS955.1	ENSP00000	Q6UY14.160		UPI00001E0572	1
09931.3	1	CCDS3085	ENSP00000	Q86YZ3.159		UPI00001D7CAD	
09931.3	1	CCDS3085	ENSP00000	Q86YZ3.159		UPI00001D7CAD	
15.3	1	CCDS3087	ENSP00000	Q53ET0.132		UPI00001A9468	
56.4	1	CCDS1073	ENSP00000	Q15126.18	Q6FGV9.1	UPI000013E263	1
36.3	1	CCDS1163	ENSP00000	O15085.195		UPI00001D62A7	
36.4	1	CCDS1183	ENSP00000	Q16570.18	Q5Y7A2.1	UPI000016A110	1
30.5	1	CCDS1281	ENSP00000	P12259.240		UPI000016A8CE	1
37844.1			ENSP00000	Q9Y520.13	A0A2R8YE	UPI0001AE797A	
38.4	1	CCDS1313	ENSP00000	P01008.25	A0A024R9	UPI000002C0C1	1
94.3	1	CCDS1388	ENSP00000	P05160.195		UPI000013D8E0	1
36.5	1	CCDS1389	ENSP00000	Q8IZT6.178		UPI0000458904	1
54586.2	5	CCDS5345	ENSP00000	Q86VF2.159		UPI0001B300F4	
08.4	1	CCDS1442	ENSP00000	Q9NVF9.1	A0A024R9	UPI000007286B	
02.3	1	CCDS4430	ENSP00000	Q07002.20	A0A024R9	UPI00001D7D3B	
15.4	1	CCDS1477	ENSP00000	P04003.201		UPI0000126C28	
46.3	1	CCDS3102	ENSP00000	P07814.241		UPI0000205E8C	1
95.4	1	CCDS3104	ENSP00000	O14904.17	D9ZGG3.8	UPI000005104B	
36125.1			ENSP00000505517		A0A7P0Z4	UPI0003EB0068	
36125.1			ENSP00000505517		A0A7P0Z4	UPI0003EB0068	
36.5	1	CCDS1615	ENSP00000	Q12836.157		UPI000006F0E4	
75978.1		CCDS1616	ENSP00000	P20309.20	A0A024R3	UPI0000050453	1
93424.2	5	CCDS5349	ENSP00000	Q9H5I1.184		UPI0000136177	
24.4	1	CCDS7144	ENSP00000	Q5T5U3.159		UPI0001639C78	
53.4	1	CCDS7151	ENSP00000	Q96TA2.185		UPI0000001C16	1
38.3	1	CCDS7164	ENSP00000	O95425.185		UPI0000366678	1
72004.3	1	CCDS6051	ENSP00000	Q9H2F5.170		UPI000013D399	
97.3	5		ENSP00000	Q9BXX3.13	R4GNA2.6	UPI000170CC5D	
21.4	1	CCDS7210	ENSP00000	Q9NTK1.138		UPI000006E55C	
34.4	1	CCDS7312	ENSP00000	Q96MN9.156		UPI00000736CF	
05751.3	1	CCDS4152	ENSP00000	Q641Q2.128		UPI000044FEAB	
30.5	1	CCDS7250	ENSP00000	Q8NFU5.156		UPI000006FB8E	1
30112.2	5	CCDS8148	ENSP00000	Q86V20.142		UPI00004DC638	
20.3	1	CCDS7403	ENSP00000	Q13325.180		UPI000012D3E6	
74.3		CCDS7425	ENSP00000	Q6V0L0.147		UPI00002289F0	1
41.4	1	CCDS4155	ENSP00000	Q9P212.182		UPI00001F93EE	1
72.3	1	CCDS7435	ENSP00000	P33260.187		UPI000013DE1D	
30736.2	1	CCDS7317	ENSP00000	Q6AHZ1.159		UPI00003FEC7C	
90.4	1	CCDS7495	ENSP00000	Q9NQW1.145		UPI0000070A8E	

0.3	1	CCDS4156	ENSP0000	Q9H467.130		UPI000006EFB6	
5.7	1	CCDS3128	ENSP0000	Q8NDM7.140		UPI0000D60FC7	1
7.4		CCDS7616	ENSP0000	Q9Y2H2.145		UPI000006FBCA	
7.5	2	CCDS3130	ENSP0000	Q32M84.124		UPI00003CEFB9	
.2.5			ENSP0000	P16455.205		UPI00001112F1	1
7.4	1	CCDS7662	ENSP0000	Q5VWI1.142		UPI00004589C8	
6.3	1	CCDS7665	ENSP0000	O14531.181		UPI000013DC70	
0049.3	5	CCDS5810	ENSP0000	Q8IYW2.137		UPI0001B79116	
8.4	1	CCDS7675	ENSP0000	O95231.171		UPI0000070A25	
3.4	1	CCDS7695	ENSP0000	Q9Y446.171		UPI0000034ACC	
3.3	1	CCDS7701	ENSP0000	Q8IXW0.123		UPI0000456441	
3.3	1	CCDS7701	ENSP0000	Q8IXW0.123		UPI0000456441	
6581.2	1	CCDS6598	ENSP0000	Q9P1Y6.1	A0A024RC	UPI000041A715	
4.4	1	CCDS3133	ENSP0000	Q16540.1	A0A024RC	UPI000006EBEF	
	2		ENSP00000380465		A8MYK1.9	UPI000155D4D4	
1.4	1	CCDS4452	ENSP0000	P19474.228		UPI000013450B	
4.4		CCDS7751	ENSP0000	Q9H255.1	A0A126GV	UPI000003B49B	
8794.2	1	CCDS4453	ENSP0000	Q8N612.128		UPI000006E6DD	
5.2		CCDS7783	ENSP0000	Q8WZ94.1	A0A126GV	UPI000004B1F2	
4461.2			ENSP0000	Q8NH74.1	A0A126GV	UPI0000041DF4	
2659.2	1	CCDS6072	ENSP0000	Q96K76.173		UPI00004DC7F5	
5.4		CCDS3143	ENSP0000	Q14654.2	B2RC52.97	UPI0000161990	1
8.4	5	CCDS4162	ENSP0000	Q8N9C0.146		UPI0001D3B05B	
		CCDS8620	ENSP00000491133				
5274.1		CCDS3149	ENSP0000	Q8NH70.1	A0A126GV	UPI0000061EB2	
5566.3		CCDS3155	ENSP0000	Q96R09.154		UPI000004B227	
9559.3	1	CCDS4165	ENSP0000	Q1KMD3.141		UPI0000161949	
7.4	1	CCDS8108	ENSP0000	Q96FS4.178		UPI0000135D8A	
4.3		CCDS8134	ENSP0000	Q9HCU0.161		UPI0000049803	
2.3	1	CCDS8137	ENSP0000	Q14542.183		UPI000013F0E7	
9.5	1	CCDS8142	ENSP0000	Q8NFJ9.153		UPI0000073430	1
.7.3	1	CCDS8193	ENSP0000	O95750.1	A0A7U3L4	UPI0000037216	
6.5	1	CCDS8235	ENSP00000289575		A0A024R5	UPI000000D99A	
8816.3	5	CCDS4468	ENSP0000	Q6N022.146		UPI0000DD8112	1
4871.2	5	CCDS4470	ENSP0000	A8MXK1.96		UPI00001AEA3E	
.0.2	1	CCDS4473	ENSP0000	Q8NFD2.148		UPI000000DCCF	
2.4	1	CCDS8411	ENSP0000	Q9H3H5.1	A0A024R3	UPI000012BA3F	1
5213.3		CCDS8625	ENSP0000	Q8NH80.127		UPI000004B22A	
0.4	1	CCDS4475	ENSP0000	Q96MS0.167		UPI000035AA82	1
4874.1	2	CCDS4476	ENSP0000	P0C8F1.80		UPI0000251F10	
4757.2	5	CCDS5819	ENSP0000	Q11206.183		UPI0000000CA8	
2499.3	1	CCDS8518	ENSP0000	Q9BSD3.134		UPI0000070995	
6.4	1	CCDS8573	ENSP0000	Q9NZP8.161		UPI0000EE67FA	
7033.2	1	CCDS3173	ENSP0000	Q6EIG7.133		UPI00003BE8D6	
3.6	5	CCDS8641	ENSP00000279575		E9PAL0.85	UPI000013DBDC	
	1	CCDS8642	ENSP00000420826		A0A4W8X	UPI00002371B8	
8772.2	5	CCDS7345	ENSP00000445381		F5H369.87	UPI00020655D2	
0298.3	5		ENSP00000449273		F8VWL3.8	UPI00020CE034	1
4.4	1	CCDS8734	ENSP0000	Q9UBJ2.180		UPI000004C4C6	
2.4	1	CCDS8783	ENSP0000	P41219.182		UPI000013CF92	1

37806.4	5	CCDS4178	ENSP0000	Q9HCH0.129		UPI00006C1298	
31.4	1	CCDS3180	ENSP0000	Q14533.176		UPI0000DBEEF8	1
55.4	1	CCDS8828.	ENSP0000	P04259.201		UPI000013CD50	1
32.4	1	CCDS3183	ENSP0000	P21860.235		UPI000012A113	1
53.5	1	CCDS8931.	ENSP0000	P42226.211		UPI00000473FE	1
25.5	1	CCDS5585	ENSP0000	O75334.166		UPI0000168655	
79910.2	1	CCDS4181	ENSP0000	Q96JM4.1	A0A140VJI	UPI0000ED4E82	
51.4	1	CCDS3187	ENSP0000	Q6ZV73.157		UPI00001FB2F4	
51.4	1	CCDS3187	ENSP0000	Q6ZV73.157		UPI00001FB2F4	
51.4	1	CCDS3187	ENSP0000	Q6ZV73.157		UPI00001FB2F4	
37.4	1	CCDS4498	ENSP0000	Q9Y6K5.169		UPI000034ECD3	
36.4	1	CCDS4499	ENSP0000	A7MD48.9	V5T9A0.52	UPI00001FBC3F	
72106.1			ENSP00000501095		A0A669KB	UPI000387C235	1
56.4	1	CCDS9261.	ENSP0000	Q8IY37.165		UPI00000742DC	1
36103.3	5	CCDS4500	ENSP0000	Q8N3T6.110		UPI0001C0B37C	
42684.2	5		ENSP0000	Q9UJ78.150		UPI000178DE9C	
76.7	1	CCDS3194	ENSP0000	P54707.206		UPI000016B154	
37.3	5	CCDS4187	ENSP0000	Q5TBA9.137		UPI000046FD40	1
59.4	5	CCDS9344.	ENSP0000	P51587.230		UPI00001FCBCC	1
51.5	1	CCDS9379.	ENSP0000	Q6N069.1	A4FU51.12	UPI00001B559E	
93.4	1		ENSP0000	O75503.173		UPI0001722CE5	1
57.5	1		ENSP0000	O75592.199		UPI0001CB7C13	
50.5	1	CCDS9479.	ENSP0000	Q13217.19	A8KA82.12	UPI000006F088	1
.3354.1		CCDS3201	ENSP0000	B2RN74.95		UPI00004EAFF2	
45442.1	1	CCDS7360	ENSP0000	A6NI47.104		UPI00006C1407	
97287.2			ENSP00000485150		A0A2C9F2	UPI000017DF06	
37.5	1	CCDS9555.	ENSP0000	P34096.18	Q53XB4.14	UPI000000CBC2	
33.5	1	CCDS9559.	ENSP0000	P07998.20	W0UV93.5	UPI000013448E	
20329.2	5	CCDS9565.	ENSP0000	Q9UN36.175		UPI000012FEDE	
47.5	1	CCDS9678.	ENSP0000	Q96NI6.164		UPI000000DA1E	
44.4	1	CCDS4511	ENSP0000	O00471.161		UPI0000047E53	
77.5	1	CCDS9765.	ENSP0000	P24278.196		UPI0000074252	
34.4	1	CCDS9796.	ENSP0000	Q92537.150		UPI00001394F6	
34230.2	1	CCDS6148	ENSP0000	P80192.194		UPI0000D62427	
70.4	1	CCDS9890.	ENSP0000	Q9H7Z3.157		UPI00001FD9DB	
36.7	5	CCDS9944.	ENSP0000	Q96BY7.152		UPI000155D51F	
35089.1	5		ENSP00000451125		G3V3A2.5	UPI0005D00A29	
34888.3		CCDS5391	ENSP0000	A6NKG5.102		UPI00001D7B9E	1
30236.2	2	CCDS8185	ENSP0000	A0A0U1RRK4.25		UPI000719A0D8	
20.4	5	CCDS4517	ENSP0000	Q8IVF2.136		UPI00015BB2CA	
20.4	5	CCDS4517	ENSP0000	Q8IVF2.136		UPI00015BB2CA	
20.4	5	CCDS4517	ENSP0000	Q8IVF2.136		UPI00015BB2CA	
20.4	5	CCDS4517	ENSP0000	Q8IVF2.136		UPI00015BB2CA	
20.4	5	CCDS4517	ENSP0000	Q8IVF2.136		UPI00015BB2CA	
20.4	5	CCDS4517	ENSP0000	Q8IVF2.136		UPI00015BB2CA	
20.4	5	CCDS4517	ENSP0000	Q8IVF2.136		UPI00015BB2CA	
32468.3	5	CCDS6157	ENSP0000	H3BSY2.66		UPI0001A5E7A0	
32493.2	5	CCDS6157	ENSP00000426691			UPI0001A5E7CC	
32493.2	5	CCDS6157	ENSP00000426691			UPI0001A5E7CC	
32493.2	5	CCDS6157	ENSP00000426691			UPI0001A5E7CC	

'7313.2	5	CCDS6158	ENSP0000(	Q68DA7.132		UPI0001AE68B4	
36441.2	2	CCDS6673	ENSP0000(	Q8NHP7.143		UPI000191512C	
)8.4	1	CCDS1010(	ENSP0000(	Q9H967.158		UPI000013D462	
)2.4	1	CCDS1013	ENSP0000(	Q8TCT8.156		UPI0000013591	1
56.5	1	CCDS1016	ENSP0000(	Q0VF96.105		UPI000019B4EF	
30.4	1		ENSP0000(	Q12982.174		UPI000013019E	
21.3		CCDS3225	ENSP0000(	Q709C8.138		UPI000023B7D3	1
.2642.3	1	CCDS3228	ENSP0000(	Q8IUY3.111		UPI00001FE7DE	
54404.2	1	CCDS5838	ENSP0000(	A6NDK9.95		UPI00001AECE1	
)6.3	1	CCDS3230(	ENSP0000(	Q9UPX6.128		UPI00001B2F56	
'8.5	1		ENSP0000(	Q96L96.139		UPI00001FEA90	1
25.4	1	CCDS4534	ENSP0000(	Q9NPR2.172		UPI0000197391	
34.3	1	CCDS1041	ENSP0000(	Q96NZ8.157		UPI0000043725	
)4.5	5	CCDS4209	ENSP0000(	Q96KV7.159		UPI0000D67C48	
.1.4	1	CCDS3236	ENSP0000(	Q9Y4R8.169		UPI000016961D	1
51.4	1	CCDS3236	ENSP0000(	Q6PJ21.137		UPI000006FA20	
51.3	1	CCDS1045(	ENSP0000(	Q92901.162		UPI0000161C28	1
)3.6	1	CCDS5840	ENSP0000(	Q2KHN1.123		UPI00001FFB20	
43.3	1	CCDS1049	ENSP0000(	O15553.208		UPI000004C0CA	1
43.3	1	CCDS1049	ENSP0000(	O15553.208		UPI000004C0CA	1
)9.3	1	CCDS4211	ENSP0000(	Q9H305.144		UPI00000711C0	
'4.2	1	CCDS1054	ENSP0000(	Q96M29.119		UPI000006D1A0	
52.4	1	CCDS4211	ENSP0000(	P04554.17	Q1LZN1.11	UPI000012CD8E	
37.4	1	CCDS1055(	ENSP0000(	Q15155.192		UPI000013D37E	
'4.3	1	CCDS1056	ENSP0000(	P35749.21	A0A024QZ	UPI000012FB86	1
24.2	1	CCDS1058	ENSP0000(	Q8N807.147		UPI000000DAC6	
24.2	1	CCDS1058	ENSP0000(	Q8N807.147		UPI000000DAC6	
47886.2			ENSP00000513632			UPI0003EAEB78	
)4.4	5	CCDS1062	ENSP0000(	Q8NDV7.186		UPI000059D33E	1
	1	CCDS7385	ENSP0000(	F8W1W9.55		UPI0001E6F917	
.4.6	1	CCDS3244(	ENSP0000(	Q14586.209		UPI000045696F	
29.4	1	CCDS4547	ENSP0000(	O75113.149		UPI000013D2A2	
58.3	1	CCDS1074	ENSP0000(	Q9NSC2.181		UPI000013CCD8	1
37.5	1	CCDS1082	ENSP0000(	Q9BSU1.1	A0A024R6	UPI0000137911	
'0974.2	5	CCDS5926	ENSP0000(	Q4G0P3.146		UPI0001FEF4F9	1
'0974.2	5	CCDS5926	ENSP0000(	Q4G0P3.146		UPI0001FEF4F9	1
36.2	1	CCDS7391	ENSP0000(	Q7Z443.147		UPI00001A36E5	
)3.4	1	CCDS1090	ENSP0000(	Q92620.203		UPI000002FBFE	1
42.4	1	CCDS3249	ENSP0000(	Q8N8U2.149		UPI00001B2954	
53.5	1	CCDS1094	ENSP0000(	Q3SXM5.144		UPI0000049039	
.2759.3	1	CCDS4554	ENSP0000(	Q2VPK5.126		UPI0000251EAE	1
.2759.3	1	CCDS4554	ENSP0000(	Q2VPK5.126		UPI0000251EAE	1
42864.4	1	CCDS5405	ENSP0000(	Q92508.171		UPI0001B300F3	1
39.4	1	CCDS1098	ENSP0000(	Q8IUW3.128		UPI00000746EF	
56661.1			ENSP00000498679		A0A494C0	UPI0000200606	
)8.5	1	CCDS3253	ENSP0000(	P38570.191		UPI000049DE2D	
58.4	1	CCDS1104(	ENSP0000(	P51575.186		UPI000000DAAB	
.3.4	2	CCDS1106	ENSP0000(	P13929.21	E5RGZ4.70	UPI000016A894	1
35.2	1	CCDS1108	ENSP0000(	Q969V4.148		UPI0000136BA9	
42.3	1	CCDS1109	ENSP0000(	P14672.230		UPI000004F0B3	

9.6		CCDS4225	ENSP0000	Q2NKJ3.123		UPI000041A9A9	1
2.3	1	CCDS1116	ENSP0000	Q9Y663.156		UPI000000DAA6	
1.4	1	CCDS3259	ENSP0000	Q96R06.164		UPI0000073414	
7.4	1	CCDS4567	ENSP0000	P11388.249		UPI0000137195	1
0.3		CCDS4567	ENSP0000	Q9BYQ9.141		UPI0000366BFD	
7332.1		CCDS5928	ENSP0000	A8MTY7.91		UPI0000DD83F7	
7.5	1	CCDS1143	ENSP0000	Q7Z736.149		UPI0000200DD6	
2.5	1	CCDS4235	ENSP0000	P34998.208		UPI000002A71D	
6.6	1	CCDS1150	ENSP0000	P30260.217		UPI000012722D	
5365.3	1	CCDS3267	ENSP0000	Q9Y2D9.155		UPI000006D93D	
7.4	1	CCDS1160	ENSP0000	Q9NXB0.146		UPI00002010BB	1
5.4	1	CCDS4237	ENSP0000	O75916.197		UPI000013382A	1
5.6	1	CCDS3272	ENSP0000	Q14957.192		UPI00001AEBA4	
5058.1			ENSP00000495242		A0A2R8YF	UPI001BE0E8ED	
4.4	1	CCDS3273	ENSP0000	Q92949.1	A0A024R8	UPI000000DB81	1
8.4	1	CCDS4581	ENSP0000	O14926.164		UPI000012AC5E	1
1.3	1	CCDS3280	ENSP0000	Q5FWF5.1	A0A024RC	UPI00001C1FF4	
7.4	1	CCDS1188	ENSP0000	Q9BXW6.1	B0YJ56.11	UPI0000130E95	
2.3	5	CCDS4584	ENSP0000	Q9C0F0.126		UPI000156D0F3	1
9.4	1	CCDS4588	ENSP0000	O60346.191		UPI000051AE2E	
6089.2	1	CCDS7720	ENSP00000322361		J9JID5.63	UPI000268B415	
3.3	1	CCDS1206	ENSP0000	O95996.16	A0A0C4DG	UPI0000073D85	1
8.3	1	CCDS7425	ENSP0000	Q8TCT7.168		UPI0000033768	
4.2	5	CCDS4592	ENSP0000	Q9UPR6.135		UPI0000DD84BE	
3.3	1	CCDS3287	ENSP0000	Q13111.195		UPI000034E58B	
7868.2	5		ENSP0000	Q96Q06.129		UPI00024CDDA2	
7868.2	5		ENSP0000	Q96Q06.129		UPI00024CDDA2	
7868.2	5		ENSP0000	Q96Q06.129		UPI00024CDDA2	
7868.2	5		ENSP0000	Q96Q06.129		UPI00024CDDA2	
3.4	1	CCDS1214	ENSP0000	P36776.206		UPI000012E7EF	1
5.4	1	CCDS1215	ENSP0000	P48378.170		UPI000013D4B1	
7.5	1	CCDS1218	ENSP0000	Q9H2X3.190		UPI0000073C48	
0.7	1	CCDS1222	ENSP0000	Q9NQ55.182		UPI0000135FBE	
1439.2	1	CCDS1225	ENSP0000	Q9BWQ6.	A0A024R7	UPI00000729B3	
1.5		CCDS1226	ENSP0000	P13686.20	A0A024R7	UPI0000001288	1
8374.3	2	CCDS5865	ENSP0000	Q9UBC2.184		UPI0000D4C04A	1
2.5	1		ENSP0000	Q8IZJ3.138		UPI000059D6A5	1
9.3	1	CCDS1238	ENSP0000	Q9UK28.1	A0A024R7	UPI0000034024	
5.3	1	CCDS1238	ENSP0000	P49747.207		UPI000013C7F6	1
3.4	1	CCDS1238	ENSP0000	O14579.182		UPI000013D334	
1.4	1	CCDS1239	ENSP0000	Q8IWZ8.149		UPI00001B6B07	
4.4	1	CCDS3297	ENSP0000	Q8IZC7.157		UPI0000074138	
1411.3	2	CCDS4253	ENSP0000	Q8N7Q3.148		UPI00002376EC	
9726.2		CCDS8673	ENSP0000	P0DPD5.21		UPI0009ACDB03	
6.4	1	CCDS3299	ENSP0000	O60500.189		UPI000004EF61	1
7.4	2	CCDS1255	ENSP0000	Q9H8S5.135		UPI00017A831A	
6.3	1	CCDS1265	ENSP00000221455		A0A0A0M	UPI00003D7B96	
6.3	5	CCDS1268	ENSP0000	Q96B18.131		UPI0000202707	
8.3	1	CCDS1269	ENSP0000	Q15758.205		UPI000012507D	
7.5	1	CCDS1274	ENSP0000	P0DN86.3	A0A0F7RQ	UPI0000035497	

78.2	1	CCDS1275(	ENSP0000(	Q6NT52.126		UPI000059D759	
77.2	1	CCDS1275	ENSP0000(	A6NKQ9.116		UPI0000EE7C13	
37.3	5	CCDS4259(	ENSP0000(	Q9GZN6.150		UPI00001305CE	
74.3	4	CCDS4617(	ENSP0000(	Q96IR2.158		UPI0001662BAC	
44950.2	1	CCDS4619(	ENSP0000(	A1L4H1.113		UPI000192952A	
93.3	1	CCDS4622	ENSP0000(	Q92626.190		UPI00001C1DC2	1
99218.3	1	CCDS4265(	ENSP0000(	Q09MP3.91		UPI0000418FD4	
41.5	1	CCDS1742	ENSP0000(	P27708.236		UPI000013D558	1
52.4	1	CCDS3317	ENSP0000(	Q9NR09.188		UPI000159689D	1
76.4	2	CCDS3318(	ENSP0000(	Q6UXI7.122		UPI000006E0F8	
01330.3		CCDS8244	ENSP0000(	P0DN25.33		UPI000013E1EA	
59.4	1	CCDS3318	ENSP0000(	P42704.20	E5KNY5.94	UPI000019B4D2	1
00396.2	1	CCDS4628	ENSP0000(	Q8N5S3.99		UPI00001C1DC4	
09.4	5	CCDS4268(	ENSP0000(	Q70CQ2.146		UPI0000410E09	
97.4	1	CCDS1902	ENSP0000(	Q9UHG3.185		UPI0000048F25	
75.5	1	CCDS4270	ENSP0000(	Q9Y679.177		UPI000000163A	
03.5	1	CCDS1961	ENSP0000(	P16383.190		UPI000013C96B	
70.2	5	CCDS4634	ENSP0000(	Q9C0G6.144		UPI000163AC9D	
71.5		CCDS2002	ENSP0000(	Q86YJ6.127		UPI00002088D9	
00.3	1	CCDS2015	ENSP0000(	Q96BQ3.147		UPI000006E7BB	
93982.1	5		ENSP0000(	Q5JPF3.109		UPI0003EAEB48	
98.3	1	CCDS2034	ENSP0000(	Q16281.198		UPI000004717B	1
77406.2	1	CCDS5943	ENSP0000(	P0CG38.87		UPI00006C04CB	
77406.2	1	CCDS5943	ENSP0000(	P0CG38.87		UPI00006C04CB	
77406.2	1	CCDS5943	ENSP0000(	P0CG38.87		UPI00006C04CB	
08.3	1	CCDS2170	ENSP0000(	O43194.167		UPI0000046406	
33.3	1	CCDS3329	ENSP0000(	Q15042.15	B9A6J2.91	UPI0000072FAA	1
54508.2	5	CCDS5440	ENSP0000(	P20929.200		UPI0002065BA0	1
58.3	1	CCDS2203	ENSP0000(	Q7Z7M9.144		UPI000019AD19	
53.5	1	CCDS3331	ENSP0000(	Q7Z4L5.162		UPI000020900A	1
53.3	2	CCDS2228	ENSP0000(	Q6ZMG9.162		UPI000020902D	
75.4		CCDS2276	ENSP0000(	Q86WT1.152		UPI0000209161	
57550.2	5	CCDS5943	ENSP00000467141		A0A0A0M	UPI00046209C6	1
51.4	5	CCDS5442(	ENSP0000(	Q5CZC0.101		UPI000198D023	1
59.4	1	CCDS2367	ENSP0000(	P24534.23	A0A024R3	UPI0000000C68	1
93730.1	1	CCDS4650	ENSP0000(	A2CJ06.106		UPI0000EE0AB9	
91.4	1	CCDS2378	ENSP0000(	P07320.20	A0A140CT	UPI0000161BE3	1
79866.2	1	CCDS2419	ENSP0000(	Q9Y276.17	A0A024R4	UPI0000073C9C	1
02.4	1	CCDS4652	ENSP0000(	Q8N1F8.149		UPI000013E286	
51305.2			ENSP00000508848			UPI000A20F37B	
18234.2	1	CCDS8259(	ENSP0000(	O15079.154		UPI0000135B3B	
78.4	1	CCDS1307	ENSP0000(	P25100.17	B0ZBE0.11	UPI000003B078	
04.5	1	CCDS1312	ENSP0000(	Q96L93.18	A0A140VK	UPI0000206A5A	
98.3	1	CCDS1316(	ENSP0000(	P01037.199		UPI000013E9BC	
90.4	1	CCDS4659	ENSP0000(	Q9Y5R2.190		UPI000012F259	
55.4	1	CCDS1330	ENSP0000(	Q9H9F9.148		UPI000006F9EF	
72179.1	5		ENSP00000217073		A0A6Q8JF	UPI0011CAB1D0	
08.3	1	CCDS1334(	ENSP0000(	Q02383.161		UPI0000135845	
03.5	2	CCDS1338	ENSP0000(	Q9BR11.132		UPI0000470896	
35.3	1	CCDS1341	ENSP0000(	Q9P2E3.163		UPI000012DD83	1

6.4	5	CCDS1349	ENSP0000	Q9H4K7.158		UPI000006EF41	
3.3	2	CCDS1349	ENSP0000	Q8NC74.133		UPI000013CDA2	
0.5	1	CCDS4663	ENSP0000	P57052.163		UPI0000133382	
9.4	2	CCDS1356	ENSP0000	P48552.187		UPI000004A0A8	1
7.3	1	CCDS1358	ENSP0000	Q9Y5T5.189		UPI00001379FA	
8.3		CCDS1358	ENSP0000	P56748.16	A0A0K0K1	UPI00000389FD	
5.3	1	CCDS4664	ENSP0000	O60287.166		UPI0000185F65	
4.4	1	CCDS3354	ENSP0000	P38484.19	A8K881.10	UPI00001514B0	1
.9.6	1	CCDS1363	ENSP0000	P15382.20	C7S316.95	UPI000012F141	1
20714.2		CCDS1364	ENSP00000509598			UPI000013D876	
1.3	1	CCDS1364	ENSP0000	Q13112.212		UPI0000126DD1	
4.5	1	CCDS3357	ENSP0000	P57059.199		UPI0000206F2B	1
9.3	1	CCDS3357	ENSP0000	Q15269.195		UPI0000169D5D	
9.8	1	CCDS3358	ENSP0000	P0DPI2.20	A0A140VK	UPI0000169D5E	
9.6	1	CCDS4295	ENSP0000	O75144.1	A0N0L8.12	UPI0000049DCB	
7.3	1	CCDS4665	ENSP0000	Q9UJW3.175		UPI0000167F8E	
2313.1	5	CCDS7481	ENSP0000	P0C7N4.71		UPI00020EA2BF	
2.2		CCDS4666	ENSP0000	Q9UFD9.158		UPI0000237729	
5.6	1	CCDS1375	ENSP0000	O43272.189		UPI00001AE5E1	1
9200.1			ENSP00000497003		A0A3B3IS1	UPI0005D01A40	1
6.7	1	CCDS1377	ENSP0000	Q6ICL3.125		UPI0000050339	1
5.5	1	CCDS4666	ENSP0000	Q96GP6.165		UPI000004D28D	1
9.4	1	CCDS4298	ENSP0000	Q6PGQ1.111		UPI00001CE017	
0.6	1	CCDS3361	ENSP0000	Q69YQ0.144		UPI0004620B3B	1
8497.3	1	CCDS4667	ENSP0000	Q2NKQ1.123		UPI0001533DB1	
5206.2	1	CCDS4667	ENSP0000	Q9BY89.130		UPI00002073DC	
0.2	1	CCDS1384	ENSP0000	O95567.126		UPI0000073FE0	
7.3	1	CCDS1390	ENSP0000	Q9NY91.159		UPI00001359F4	
8.3	1	CCDS1391	ENSP0000	O60784.193		UPI0000137118	1
1.6	1		ENSP00000347847		A0A499FIJ	UPI000EA833A5	1
5.4	1	CCDS1402	ENSP0000	Q96RJ3.16	Q5H8V1.1	UPI0000049F80	1
5.3	1	CCDS1406	ENSP0000	P85298.112		UPI00000372DE	
.1.4	1	CCDS1406	ENSP0000	Q9NWS6.1	A0A024R4	UPI000022B897	
8328.1			ENSP00000501367		A0A6I8PR	UPI00002079D0	1
5.3	5	CCDS2574	ENSP0000	Q8IYJ1.127		UPI0000412048	
3.4	5	CCDS2670	ENSP0000	O15195.170		UPI000022BFB0	
8.4	1	CCDS2678	ENSP0000	O75191.142		UPI0000160544	
5.5	1	CCDS4679	ENSP0000	Q14524.225		UPI0000074136	1
2646.3	1	CCDS4307	ENSP0000	Q9UPV9.167		UPI0000139F52	1
4442.3	2	CCDS2719	ENSP0000	Q8TBZ5.158		UPI0000072CFB	
9.5	1	CCDS2743	ENSP0000	Q60I27.13	A0A024R2	UPI00001B5641	
.1.3	1	CCDS4308	ENSP0000	Q9BXS9.167		UPI0000135460	
2.4	1	CCDS2789	ENSP0000	P55268.200		UPI000013EA62	1
7.4	1	CCDS2807	ENSP0000	Q04912.216		UPI000159688A	1
2.5	1	CCDS4309	ENSP0000	Q9NS23.180		UPI0000073E95	
6.3	1	CCDS3376	ENSP0000	Q9NY15.183		UPI0000140C12	
6.3	1	CCDS3376	ENSP0000	Q9NY15.183		UPI0000140C12	
6.5	1	CCDS2861	ENSP0000	Q9BVP2.183		UPI000003C48A	
6028.2	5		ENSP00000418137		E9PG32.73	UPI0004620994	
1.3	1	CCDS3378	ENSP0000	Q96JA1.162		UPI000004C5BE	

8165.1	4		ENSP0000	A0A1B0GTH6.20		UPI0005D01EE5	
1.3			ENSP00000508967			UPI00001AE9AC	
2.4	1	CCDS3382	ENSP0000	Q6NW34.138		UPI0000367197	1
6282.2	1		ENSP0000	Q14789.182		UPI000020A040	
6282.2	1		ENSP0000	Q14789.182		UPI000020A040	
4090.1			ENSP00000496592		A0A2R8YF	UPI0011494E36	
6.5	1	CCDS3113	ENSP0000	Q8IWZ5.156		UPI00001AEAE0	
2.5	1	CCDS3130	ENSP0000	Q8NDZ4.121		UPI000006EE5C	
1.3	1	CCDS3387	ENSP0000	P15086.205		UPI00001271CD	
3.2	1	CCDS3184	ENSP0000	P49788.151		UPI000006E088	
8.3	1	CCDS3272	ENSP0000	Q8WWY8.148		UPI000003AEB8	1
6.7	5	CCDS5470	ENSP00000417498		E9PDY6.71	UPI0001B3CB30	1
6.7	5	CCDS5470	ENSP00000417498		E9PDY6.71	UPI0001B3CB30	1
6.7	5	CCDS5470	ENSP00000417498		E9PDY6.71	UPI0001B3CB30	1
6.7	5	CCDS5470	ENSP00000417498		E9PDY6.71	UPI0001B3CB30	1
6.7	5	CCDS5470	ENSP00000417498		E9PDY6.71	UPI0001B3CB30	1
6.7	5	CCDS5470	ENSP00000417498		E9PDY6.71	UPI0001B3CB30	1
6.7	5	CCDS5470	ENSP00000417498		E9PDY6.71	UPI0001B3CB30	1
6.7	5	CCDS5470	ENSP00000417498		E9PDY6.71	UPI0001B3CB30	1
6.7	5	CCDS5470	ENSP00000417498		E9PDY6.71	UPI0001B3CB30	1
5.4	1	CCDS3340	ENSP0000	O14976.200		UPI000012B04A	
4154.1	1	CCDS3366	ENSP0000	O14924.191		UPI0000133830	
4.3	1	CCDS3395	ENSP0000	Q9Y2E5.176		UPI000004BF05	
4.5	1	CCDS3400	ENSP0000	P83110.172		UPI0000001647	
4.3	5	CCDS3402	ENSP0000	Q8IYL2.133		UPI0000DE1FA2	
0.4	1	CCDS3441	ENSP0000	Q8WZ64.176		UPI000013E917	
7.4	1	CCDS3477	ENSP0000	Q9Y5Q5.191		UPI0000168657	1
7552.1			ENSP00000507980		A0A804HK	UPI0005D036A5	
6.4	1	CCDS3545	ENSP0000	P01591.177		UPI00000700B2	
5.4	1	CCDS7514	ENSP0000	O60763.183		UPI0000EE2C4E	
4.7	5	CCDS5477	ENSP0000	Q86XX4.170		UPI000021D4C2	1
1.6	1	CCDS3609	ENSP0000	Q8IZQ1.17	A0A024RD	UPI000013E2C7	1
1.5	1	CCDS3686	ENSP0000	Q9BZM1.1	Q542Y6.1	UPI0000001BF6	
7.3	1	CCDS3405	ENSP0000	Q9UMS6.165		UPI00001D75EB	
4.4	1	CCDS3720	ENSP0000	P08758.23	V9HWE0.5	UPI000002BA00	1
7.6	1	CCDS3754	ENSP0000	Q9Y3S2.150		UPI000006E4AB	
5595.3	5	CCDS3408	ENSP0000	P53367.17	B4E273.11	UPI0000125668	
4.5	1	CCDS3796	ENSP0000	P48167.201		UPI000000161E	1
9.5	1	CCDS4328	ENSP0000	P49767.182		UPI0000001C2A	1
9.4	1	CCDS3835	ENSP0000	P14316.193		UPI000012D888	
7.4	1	CCDS4330	ENSP0000	Q9NQV7.174		UPI00006C9E90	
0.4	1	CCDS3413	ENSP0000	O15018.1	A0A024RE	UPI000069648B	
0.4	1	CCDS3908	ENSP0000	Q9BYV1.163		UPI0000125709	1
3.4	1	CCDS3920	ENSP0000	Q6KC79.180		UPI00003761B5	1
4732.1			ENSP00000498265		A0A494BZ	UPI000387B4DC	1
5.3	1	CCDS3921	ENSP0000	O75694.1	A0A024R0	UPI0000001C7F	1
4.5	5	CCDS4014	ENSP0000	Q8WV60.1	A0A024RA	UPI000066D9F8	
4441.3	1	CCDS3419	ENSP0000	A5PLL1.104		UPI00003672FA	
3.3		CCDS4074	ENSP0000	Q8NGU9.131		UPI000003BCD0	
0.4	5	CCDS3420	ENSP0000	O14646.200		UPI000013DD75	1

40.5	1	CCDS4335	ENSP00000	Q9HCS5.157		UPI000020C3F8	
40.5	1	CCDS4335	ENSP00000	Q9HCS5.157		UPI000020C3F8	
75405.1	5	CCDS4134	ENSP00000	Q8N960.145		UPI0000D79C73	1
99.4	1	CCDS3422	ENSP00000	P35556.216		UPI0000519468	1
32.4	1	CCDS3423	ENSP00000	Q92878.196		UPI000006E9ED	1
23.4	1	CCDS4164	ENSP00000	Q9UHB7.171		UPI000006F558	1
45.4	1	CCDS4178	ENSP00000	P49069.165		UPI0000126E88	
00921.2	1	CCDS7806	ENSP00000	Q9NZM6.156		UPI000013C93B	
40.4		CCDS4249	ENSP00000	Q9Y5E2.164		UPI00001273E3	
31.3		CCDS4253	ENSP00000	Q9Y5F2.173		UPI00001273E6	
.6.4	1	CCDS4729	ENSP00000	Q9Y5H0.166		UPI0000161C1A	
36.4	1	CCDS5492	ENSP00000	Q9UN71.157		UPI000006F773	
36.4	1	CCDS5492	ENSP00000	Q9UN71.157		UPI000006F773	
46.4	1	CCDS4338	ENSP00000	Q9NQ38.193		UPI000020CF22	1
24.6		CCDS4292	ENSP00000	P07550.23	X5DQM5.4	UPI000013EAD0	1
.1.3	1	CCDS4356	ENSP00000	Q16445.187		UPI000013DA14	
.7.4	1	CCDS4380	ENSP00000	P28562.200		UPI000004EED8	
27.4	1	CCDS4390	ENSP00000	Q17RY0.127		UPI000020C179	
00946.3	5	CCDS5495	ENSP00000	Q96PV7.127		UPI0001D3BB6A	
73.4	1	CCDS4434	ENSP00000	Q8IUZ5.162		UPI00000746D3	1
70692.2	5	CCDS5496	ENSP00000	Q8TC20.137		UPI0001C0B380	
52.4	1	CCDS4517	ENSP00000	O75603.159		UPI0000073DE7	1
24.4	1	CCDS4541	ENSP00000	P49790.199		UPI000013D251	
74.3	1	CCDS4546	ENSP00000	Q5VV42.155		UPI000013DA21	
41.3	5	CCDS3438	ENSP00000	Q14676.20	A0A1U9XB	UPI00001AF453	
05414.1	2	CCDS5900	ENSP00000	E2RYF6.66		UPI0001E92A31	
.7.6		CCDS3439	ENSP00000	P10321.20	Q6R739.17	UPI000008AEBB	1
.7.6		CCDS3439	ENSP00000	P10321.20	Q6R739.17	UPI000008AEBB	1
.7.6		CCDS3439	ENSP00000	P10321.20	Q6R739.17	UPI000008AEBB	1
.7.6		CCDS3439	ENSP00000	P10321.20	Q6R739.17	UPI000008AEBB	1
.7.6		CCDS3439	ENSP00000	P10321.20	Q6R739.17	UPI000008AEBB	1
.7.6		CCDS3439	ENSP00000	P10321.20	Q6R739.17	UPI000008AEBB	1
.7.6		CCDS3439	ENSP00000	P10321.20	Q6R739.17	UPI000008AEBB	1
.7.6		CCDS3439	ENSP00000	P10321.20	Q6R739.17	UPI000008AEBB	1
.7.6		CCDS3439	ENSP00000	P10321.20	Q6R739.17	UPI000008AEBB	1
.7.6		CCDS3439	ENSP00000	P10321.20	Q6R739.17	UPI000008AEBB	1
.7.6		CCDS3439	ENSP00000	P10321.20	Q6R739.17	UPI000008AEBB	1
.7.6		CCDS3439	ENSP00000	P10321.20	Q6R739.17	UPI000008AEBB	1
.7.6		CCDS3439	ENSP00000	P10321.20	Q6R739.17	UPI000008AEBB	1
.7.6		CCDS3439	ENSP00000	P10321.20	Q6R739.17	UPI000008AEBB	1
.7.6		CCDS3439	ENSP00000	P10321.20	Q6R739.17	UPI000008AEBB	1
.7.6		CCDS3439	ENSP00000	P10321.20	Q6R739.17	UPI000008AEBB	1
.7.6		CCDS3439	ENSP00000	P10321.20	Q6R739.17	UPI000008AEBB	1
.7.6		CCDS3439	ENSP00000	P10321.20	Q6R739.17	UPI000008AEBB	1
24.4		CCDS4740	ENSP00000	P01911.17	D7RIH8.94	UPI000008A1F7	1
24.4	1	CCDS4783	ENSP00000	Q14573.205		UPI000013CB74	1
41.4	2	CCDS4787	ENSP00000	Q14833.17	A1L4F9.13	UPI000004A7DE	
95.3	1	CCDS3443	ENSP00000	Q9ULD4.163		UPI00001C1E4C	
50.3	1	CCDS4743	ENSP00000	Q9HAV4.170		UPI000006CC97	
02.3	1	CCDS4902	ENSP00000	Q9Y253.18	A0A024RD	UPI000006F8FD	1

'3.4	1	CCDS4958.	ENSP0000(	P22003.19	M9VUD0.6	UPI0000126A2C	1
'4736.1			ENSP00000505098		A0A7P0T8	UPI000387B240	1
33.6	1	CCDS5015.	ENSP0000(	P21554.21	S5TLS4.58	UPI00000008AA	
.5.5	1	CCDS5067.	ENSP0000(	Q8WV93.149		UPI0000072226	
56.7	1	CCDS5101.	ENSP0000(	P29966.185		UPI000013DDF0	
!6.4	5	CCDS5138.	ENSP0000(	P24043.220		UPI00003673E0	1
31.4	1	CCDS5141.	ENSP0000(	O43491.196		UPI0000129AF9	
32.6	1	CCDS5220.	ENSP0000(	Q86VZ4.151		UPI0000042403	
32.3	1	CCDS4751	ENSP0000(	Q4ZHG4.135		UPI0000579B80	
52.5	2	CCDS4751	ENSP0000(	Q5SW24.120		UPI00001D8145	
!3.4	1	CCDS4752	ENSP0000(	Q8IXL6.151		UPI00001B5C04	1
)7620.2	1	CCDS4353	ENSP0000(	Q6ZMB5.119		UPI000013E413	
55.3		CCDS4752	ENSP0000(	O43299.151		UPI00003E5903	1
!1.5	1	CCDS5366.	ENSP0000(	P35869.21	A0A024R9	UPI0000125727	1
'7115.2	5	CCDS6460	ENSP0000(	Q96DT5.175		UPI0002B8CE70	1
'4.5	1	CCDS5430.	ENSP0000(	O95050.168		UPI000013C526	
50.3	2	CCDS3461	ENSP0000(	Q8NBF6.1	A0A024RA	UPI0000049D97	
)1648.2	1	CCDS4357	ENSP00000370552		A0A0C4DF	UPI000015C225	1
11.4	1	CCDS3462	ENSP0000(	Q02218.213		UPI000006D5FE	1
16334.2	5	CCDS4758	ENSP0000(	O15069.141		UPI00001D747D	
18.12	1	CCDS5506.	ENSP0000(	Q68CZ2.148		UPI00001AE9DA	
)0159.3	1	CCDS4758	ENSP0000(	Q8NEG2.107		UPI00001C1E6B	
)8.5	1	CCDS3463	ENSP0000(	O75128.146		UPI00001A9480	
)8.5	1	CCDS3463	ENSP0000(	O75128.146		UPI00001A9480	
'0129.2	1	CCDS4359	ENSP0000(	Q96JC4.162		UPI000006E615	1
)2.5	1	CCDS4359	ENSP0000(	O75344.189		UPI000000165F	1
)8.4	1	CCDS5549.	ENSP0000(	Q9UIG0.204		UPI0000126731	1
'9.3	1	CCDS3467	ENSP0000(	Q8IYE0.129		UPI000020F44F	
15.4	1	CCDS3469	ENSP0000(	O75127.14	A4D273.11	UPI0000223FFA	
)5.3	1	CCDS5676.	ENSP0000(	Q9HB55.176		UPI0000126C0D	
54.4	2	CCDS5696.	ENSP0000(	Q6ZVC0.117		UPI00001C0A2E	
50.2	5	CCDS7826	ENSP0000(	Q02505.154		UPI000455B941	
34.5	1	CCDS5715.	ENSP0000(	O60568.192		UPI0000046664	1
11.2		CCDS5746.	ENSP0000(	O43511.202		UPI00001315A4	1
50.4	1	CCDS3473	ENSP0000(	O00458.15	A4D0U1.1	UPI00000304DC	1
54.11	5	CCDS5515	ENSP0000(	Q86UW7.159		UPI0000668808	
39.4	1	CCDS5846.	ENSP0000(	P51857.173		UPI0000125764	1
30413.3	5	CCDS4773	ENSP0000(	O60393.153		UPI00019B220B	1
.1.4	1	CCDS4773	ENSP0000(	Q8N393.153		UPI000013FD40	
!0.5	5	CCDS6015.	ENSP0000(	Q9Y250.164		UPI000006DEE8	1
!2.3	1	CCDS3485	ENSP0000(	P05423.189		UPI000006CE69	
54.4	1	CCDS5521	ENSP0000(	Q9NQT8.185		UPI000035B257	
37.5	1	CCDS3487	ENSP0000(	P00390.25	V9HW90.6	UPI000012BBFF	1
)2814.3	1	CCDS3488	ENSP0000(	Q6WKZ4.159		UPI0000D624B1	
'4.5	1	CCDS6101.	ENSP0000(	Q9UBL3.190		UPI0000038D65	
54.4	1	CCDS6138.	ENSP0000(	Q96K19.147		UPI000013CAA2	1
11.4	1	CCDS6153.	ENSP0000(	Q9UI12.19	A0A024R7	UPI0000000966	
30.4	1	CCDS6157.	ENSP0000(	O75608.18	Q6IAQ1.13	UPI0000072858	
50.3	1	CCDS6190.	ENSP0000(	Q8IWW8.131		UPI000004966C	
59.4	1	CCDS6312.	ENSP0000(	Q96RS6.129		UPI000021048A	

)6.4	1	CCDS3494!	ENSP0000(	Q92597.199		UPI000012FEDD	1
)5.3	1	CCDS6399.	ENSP0000(	Q9H7X3.157		UPI000013E0BC	
.7.3	1	CCDS6402.	ENSP0000(	Q8IXZ2.149		UPI0000160D96	
.6309.2	5	CCDS8333.	ENSP0000(	A6NE52.107		UPI000173AA02	
)1.5	1	CCDS6418.	ENSP0000(	Q14137.212		UPI0000126A63	
I9.4	1	CCDS6424.	ENSP0000(	Q6P5W5.150		UPI0002064DC4	1
I9.4	1	CCDS6424.	ENSP0000(	Q6P5W5.150		UPI0002064DC4	1
'8090.1		CCDS7837!	ENSP00000512441			UPI0000251DC1	
.3.4	5	CCDS6551.	ENSP0000(	Q5T7B8.156		UPI00004F9D23	
.8.3	1	CCDS6584.	ENSP0000(	Q8N4T4.146		UPI000006FAA2	
;2.5	1	CCDS6647.	ENSP0000(	Q9BX84.164		UPI000006E041	1
!5.3	5	CCDS4798.	ENSP0000(	Q8WUY3.143		UPI0001612CC0	
I8.4	5		ENSP00000411181		E9PCD1.73	UPI00001B09BB	1
I2.5	1	CCDS6737.	ENSP0000(	Q8IXK2.173		UPI000004D295	1
)5236.3		CCDS3512	ENSP00000310773		A0A0B4J1!	UPI0000041E03	
35.7	1	CCDS6935.	ENSP0000(	Q13868.202		UPI0000134AD4	1
			ENSP0000(	P00846.20	Q0ZFE3.14	UPI0000001709	1
			ENSP0000(	P00156.21	Q0ZFD6.14	UPI0000DA7DCA	1
!3.2	1	CCDS1421	ENSP0000(	Q8N7X4.132		UPI00001413F4	
'6.4		CCDS1426.	ENSP0000(	Q9H596.152		UPI00000342DF	
)8409.4	1	CCDS4394	ENSP0000(	P0CL81.66&O76087.1		UPI00000015F9	
I8.2	1	CCDS1433.	ENSP0000(	O95972.171		UPI000013CD7C	1
;7.4		CCDS3531.	ENSP0000(	P98169.167		UPI000013C495	
;6.5		CCDS1437(	ENSP0000(	P98168.173		UPI000013C494	
;6.5		CCDS1437(	ENSP0000(	P98168.173		UPI000013C494	
;6.5		CCDS1437(	ENSP0000(	P98168.173		UPI000013C494	
;7.5	1	CCDS3532(	ENSP0000(	Q96QF7.124		UPI0000072023	
33.3	1	CCDS1450.	ENSP0000(	Q8IYN2.130		UPI00000437D3	
34.4	1	CCDS3540!	ENSP0000(	Q8IZF6.152		UPI00004CEC5B	
)6144.3	1	CCDS7851.	ENSP00000414178		F8WA39.8.	UPI000004FD4F	
32.3	1	CCDS1166.	ENSP0000(	Q96PJ5.164		UPI000006E26B	1
.8.4	1	CCDS1554.	ENSP0000(	P09874.25	A0A024R3	UPI000013D92D	1
30.3	1	CCDS1579.	ENSP0000(	Q8WUM0.188		UPI000013D17A	1
!4.4	1	CCDS7229.	ENSP0000(	Q03468.219		UPI000000D8DA	1
I8.3	1	CCDS7637.	ENSP0000(	Q8N436.160		UPI00001AE6BE	
;2.3	1	CCDS7889.	ENSP0000(	Q9H0A0.189		UPI000013CF8E	
I1.4	1	CCDS3157.	ENSP0000(	P60852.13	V9HWI9.5.	UPI0000351AA0	1
;6.4	1	CCDS3240	ENSP0000(	Q76MJ5.157		UPI000004A5C0	
.8.5	1	CCDS1119.	ENSP0000(	Q13472.187		UPI00001371A0	1
'8.2	1	CCDS1275(	ENSP0000(	Q6NT52.126		UPI000059D759	
;4.3	1	CCDS1298.	ENSP0000(	Q9Y2P5.148		UPI0000072ECE	1
;4587.1	5		ENSP0000(	A6QL64.104		UPI000387C901	
35.3	1	CCDS2394.	ENSP0000(	Q15303.231		UPI00000499DF	1
)9.5	1	CCDS1403	ENSP0000(	Q9H4I8.16	A0A140VK	UPI0000135810	
!6328.3	1	CCDS4705	ENSP0000(	Q8TBB1.180		UPI000012E7A6	
.7.6		CCDS3439.	ENSP0000(	P10321.20	Q6R739.17	UPI000008AEBB	1
.4436.3	1	CCDS3462.	ENSP0000(	Q9UJU6.190		UPI000006EBFF	
!0.3	1	CCDS7010.	ENSP0000(	Q6UXC1.128		UPI0000237828	
)4958.2	1	CCDS7401!	ENSP0000(	B7U540.95		UPI0002064ECF	1
33538.3		CCDS4641.	ENSP0000(	Q6S8J3.153		UPI0000F58EC8	

;2.5	1	CCDS4752(	ENSP0000(	A2RRH5.114		UPI00015E06AF	
38.5	1	CCDS7605:	ENSP0000(	P43355.177		UPI0000035FCB	
.2.2	1	CCDS3100	ENSP0000(	O60825.201		UPI000012A3F0	
;0.4	1	CCDS7775.	ENSP0000(	Q9UL58.174		UPI000013DB6D	
'9210.1	2	CCDS2905.	ENSP0000(	Q70HW3.137		UPI000003615A	1
38.4	1	CCDS5537.	ENSP0000(	Q9Y3A5.19	A0A0S2Z5I	UPI000013559C	1
;4261.2		CCDS5919!	ENSP0000(	F5H284.83		UPI000016003C	
)5631.1	5		ENSP00000479693		A0A087W\	UPI000387D10E	
31.4	1	CCDS1636.	ENSP0000(	Q8NG06.131		UPI000020590E	
;2.5	1	CCDS7311	ENSP0000(	P55107.168		UPI0000126A13	
13.5	1	CCDS4453:	ENSP0000(	P17405.222		UPI000013E592	1
36094.1	5		ENSP00000490608		A0A1B0GV	UPI0005CFFF47	1
;8683.3	3	CCDS8661(	ENSP0000(	Q53H64.69		UPI0000201024	
30.3	1	CCDS1209:	ENSP0000(	Q68EA5.14	A5HJR3.12	UPI000006FE5C	
33.4	2	CCDS1233	ENSP0000(	Q6NT55.152		UPI000013D84B	1
)1.4	1	CCDS2378.	ENSP0000(	P07320.20	A0A140CT.	UPI0000161BE3	1
.6.5	1	CCDS3879.	ENSP0000(	Q96C74.128		UPI000013D9F3	
11919.2		CCDS4807:	ENSP0000(	P55808.149		UPI0000D61D1F	
'1.3	1	CCDS1369:	ENSP0000(	P35520.234		UPI0000036BC5	1

DOMAINS	SOMATIC	PHENO	PUBMED	print_sou	print_stat	OMIM_ID	M_phenot	OTIF_NAM
AFDB-ENSI	0&1	0&1				607462	Dentatorubral-pallido	
AFDB-ENSP_mappings:AF-P54259-F1.A&Coiled-coils_(Ncoils):Coil						607462	Dentatorubral-pallido	
Gene3D:1.10.506.10&PDB-ENSP_mappings:1nf1.A&PDB-ENSP_m						613113	Watson syndrome, 19	
AFDB-ENSP_mapping		1&1	26092869			609883	Bardet-Biedl syndrom	
AFDB-ENSP_mappings:AF-Q96MG2-F1.A&PANTHER:PTHR22397&						608743		
MobiDB_lite:mobidb-lite&Low_complexity_(Seg):seg						614556	Coffin-Siris syndrome	
MobiDB_lite:mobidb-lite&Low_complexity_(Seg):seg						614556	Coffin-Siris syndrome	
AFDB-ENSP_mappings:AF-Q6ZU69-F1.A								
AFDB-ENSI	0&1	1&1				300235		
AFDB-ENSI	0&1	0&1				300235		
AFDB-ENSP_mappings:AF-O75056-F1.A&Low_complexity_(Seg):se						186357	{Obesity, association	
AFDB-ENSP_mappings:AF-Q701N2-F1.A&PANTHER:PTHR23262&PANTHER:PTHR23262:SF173&Low_								
PANTHER:PTHR13817&PANTHER:PTHR13817:SF10								
AFDB-ENSP_mappings:AF-O75690-F1.A&PANTHER:PTHR23262&PANTHER:PTHR23262:SF212&Low_								
Gene3D:3.30.428.10&PDB-ENSP_mappings:6id0.U&PDB-ENSP_mappings:6id1.U&AFDB-ENSP_map								
AFDB-ENSP_mappings:AF-P14625-F1.A&Low_complexity_(Seg):se						191175		
AFDB-ENSP_mappings:AF-A0A3B3IRS2-F1.A&Low_complexity_(Seg):seg&PANTHER:PTHR31294								
Low_complexity_(Seg		1	15958500&24106602&1711266			600660	{Coronary artery disea	
AFDB-ENSP_mapping		1				602871		
PANTHER:PTHR21465		1				612078	Brittle cornea syndror	
Gene3D:3.30.70.330&AFDB-ENSP_mappings:AF-P19338-F1.A&PA						164035		
PDB-ENSP_mappings:7emf.O&PDB-ENSP_mappings:7ena.o&PDB-						607372		
AFDB-ENSP_mappings:AF-Q16626-F1.A&Pfam:PF06910&PANTHEI						143170		
AFDB-ENSP_mapping		1	18414213&15368101			600014	Nicolaides-Baraitser s	
Pfam:PF06758&PROSITE_profiles:PS51316&PANTHER:PTHR1419						614000		
PDB-ENSP_mappings:6nts.B&AFDB-ENSP_mappings:AF-Q15172-F						601643		
PANTHER:PTHR12577&PANTHER:PTHR12577:SF14&MobiDB_lite:						603803		
Gene3D:3.30.720.10&PDB-ENSP_mappings:7nfx.t&AFDB-ENSP_m						600708		
AFDB-ENSP_mappings:AF-P60371-F1.A&PANTHER:PTHR23262&P						612920	?Deafness, autosomal	
PANTHER:PTHR24043&PANTHER:PTHR24043:SF5						613619	Van den Ende-Gupta s	
PDB-ENSP_mappings:3j7y.s&PDB-ENSP_mappings:3j9m.s&PDB-El						611991		
PDB-ENSP_mappings:3jt0.A&PDB-ENSP_mappings:3jt0.B&AFDB-E						150340	Leukodystrophy, adul	
AFDB-ENSP_mappings:AF-Q02505-F10.A&AFDB-ENSP_mappings:/						158371		
PANTHER:PTHR22538&Coiled-coils_(Ncoils):Coil								
PDB-ENSP_mappings:5lph.A&PDB-ENSP_mappings:5lph.B&AFDB-						616690	Joubert syndrome 25,	
AFDB-ENSP_mappings:AF-P28908-F1.A&CDD:cd13409&SMART:SN						153243		
Gene3D:3.	0&1	0&1						
PDB-ENSP_	0&1	0&1				605347		
AFDB-ENSP_mappings:AF-Q5TF58-F1.A&Low_complexity_(Seg):seg&PANTHER:PTHR14516:SF1&PA								
PROSITE_p	0&1	0&1						
PANTHER:PTHR23253&PANTHER:PTHR23253:SF23&MobiDB_lite:						603929		
AFDB-ENSP_mapping		0&1				111680	{Hemolytic disease of	
PANTHER:PTHR16213						606210	Myopathy, congenital	
PDB-ENSP_	0&1	0&1				602790		
Gene3D:3.30.470.20&AFDB-ENSP_mappings:AF-Q8IXN7-F1.A&Pfa						618949		
AFDB-ENSP_mappings:AF-Q96MR6-F1.A&Coiled-coils_(Ncoils):Coi						614259		
PANTHER:I	0&1	0&1				607410		
PDB-ENSP_	0&1&1	1&1&1	24033266			120950	C8 deficiency, type I, 6	
AFDB-ENSP_mappings:AF-Q5VU97-F1.A&PROSITE_profiles:PS50234&PANTHER:PTHR10166&PANTI								

Gene3D:3.	0&1	0&1				613975	
AFDB-ENSP_mappings:AF-Q01459-F1.A&PROSITE_profiles:PS5191						600873	
AFDB-ENSI	0&1	1&1				617968	
PDB-ENSP_mappings:6ys4.A&PDB-ENSP_mappings:6ys4.B&PDB-E						609321	Microcephaly 14, prim
AFDB-ENSP_mappings:AF-Q68D51-F1.A&PANTHER:PTHR15288&PANTHER:PTHR15288:SF6							
AFDB-ENSP_mappings:AF-Q68D51-F1.A&PANTHER:PTHR15288&PANTHER:PTHR15288:SF6							
AFDB-ENSP_mappings:AF-Q9UNY4-F1.A&MobiDB_lite:mobidb-lite						604718	
Gene3D:2.10.25.10&AFDB-ENSP_mappings:AF-Q04721-F1.A&Pfar						600275	Alagille syndrome 2, 6
AFDB-ENSP_mappings:AF-P0DJJ0-F1.A&PROSITE_profiles:PS5174						614704	
AFDB-ENSP_mappings:AF-H0Y354-F1.A&PANTHER:PTHR31841&P						616853	
AFDB-ENSP_mappings:AF-Q8N365-F1.A&Pfam:PF15673&PANTHE						615782	
AFDB-ENSP_mappings:AF-Q6UY14-F1.A&PROSITE_profiles:PS500						610113	Ectopia lentis et pupill
AFDB-ENSI	0&1	0&1				616293	
AFDB-ENSI	0&1	0&1				616293	
AFDB-ENSI	1	1				608972	
Gene3D:3.	0&1	1&1	25741868&29290336			607622	Porokeratosis 1, multi
PANTHER:I	0&1	0&1				605708	
AFDB-ENSI	0&1	0&1	32688055			613665	[Blood group, Duffy sy
PDB-ENSP_	0&1	1&1				612309	Thrombophilia 2 due t
PANTHER:I	0&1	0&1				617373	
PDB-ENSP_mappings:1ant.I&PDB-ENSP_mappings:1ant.L&PDB-EN						107300	Thrombophilia 7 due t
AFDB-ENSI	0&1	1&1	33114181&34204301			134580	Factor XIIIB deficiency
AFDB-ENSI	0&1	1&1	25741868&18414213&2952251			605481	Microcephaly 5, prima
PANTHER:PTHR13817:SF66&PANTHER:PTHR13817						617309	
AFDB-ENSP_mappings:AF-Q9NVF9-F1.A&CDD:cd05157&PANTHER						609859	
AFDB-ENSP_mappings:AF-Q07002-F1.A&PROSITE_profiles:PS5001						169190	
AFDB-ENSI	1	1				120830	
PDB-ENSP_mappings:4hvc.A&PDB-ENSP_mappings:4hvc.B&PDB-E						138295	Leukodystrophy, hypo
AFDB-ENSI	1	1				602863	
Gene3D:2.60.40.10&I		1		geneimpri	predicted (	608616	
Gene3D:1.20.900.10&Pfam:PF00621&PROS				geneimpri	predicted (	608616	
Gene3D:2.	0&1	0&1	30679663			613514	
AFDB-ENSI	0&1	0&1				118494	Prune belly syndrome
Gene3D:2.170.270.10&PDB-ENSP_mappings:2r3a.A&PDB-ENSP_n						606503	
AFDB-ENSP_mappings:AF-Q5T5U3-F1.A&PANTHER:PTHR23175&P						609870	
HAMAP:MF_01458&PANTHER:PTHR23076&PANTHER:PTHR2307						607472	?Optic atrophy 11, 61
AFDB-ENSP_mappings:AF-O95425-F1.A&MobiDB_lite:mobidb-lite						604126	Myofibrillar myopathy
PANTHER:PTHR14898&PANTHER:PTHR14898:SF6						610999	
AFDB-ENSP_mappings:AF-Q9BX			27863482			610856	
AFDB-ENSP_mappings:AF-Q9NTK1-F1.A&PANTHER:PTHR15426&P						611309	
AFDB-ENSP_mappings:AF-Q96MN9-F1.A&PANTHER:PTHR16516&PANTHER:PTHR16516:SF5							
AFDB-ENSP_mappings:AF-Q641Q2-F1.A&PANTHER:PTHR21669&PANTHER:PTHR21669:SF1&MobiD							
AFDB-ENSP_mappings:AF-Q8NFU5-F1.A&PANTHER:PTHR12400&F						609851	
PANTHER:PTHR14495						618029	
Gene3D:1.25.40.10&PDB-ENSP_mappings:3zgq.A&PDB-ENSP_ma						616135	
Gene3D:1.10.630.10&AFDB-ENSP_mappings:AF-Q6V0L0-F1.A&Pf						608428	Focal facial dermal dy
AFDB-ENSP_mapping		1				608414	Nephrotic syndrome,
AFDB-ENSI	0&1	0&1	30038720&24340040			601131	
AFDB-ENSP_mappings:AF-Q6AHZ1-F1.A&PANTHER:PTHR24403&P						617733	
AFDB-ENSI	0&1	0&1				610258	

AFDB-ENSP_mappings:AF-Q9H467-F1.A&Low_complexity_(Seg):s						614142	
AFDB-ENSP_mappings:AF-Q8NDM7-F1.A&PANTHER:PTHR14885:S						617558	Hydrocephalus, norma
AFDB-ENSI	0&1	1&1	32778093	PanelApp-I	maternally	609389	
AFDB-ENSI	0&1	0&1					
Gene3D:1.	0&1	0&1	19902366&27471558&1748289			156569	
AFDB-ENSP_mapping		1					
Gene3D:2.	0&1	0&1				608407	
AFDB-ENSP_mappings:AF-Q8IYW2-F1.A&AFDB-ENSP_mappings:A						618543	
AFDB-ENSI	0&1&1	0&1&1		geneimpri	predicted (	607158	
Gene3D:1.25.10.10&AFDB-ENSP_mappings:				geneimpri	predicted (	605561	
AFDB-ENSI	0&1	0&1					
AFDB-ENSP_mappings:AF-Q8IXW0-F1.A&MobiDB_lite:mobidb-lite&MobiDB_lite:mobidb-lite							
AFDB-ENSI	0&1	0&1				611780	
PDB-ENSP_	0&1	0&1	35076017			600789	
PANTHER:I	0&1	0&1					
PDB-ENSP_	0&1	0&1				109092	
AFDB-ENSP_mappings:AF-Q9H255-F1.A&Transmembrane_helices						611268	
AFDB-ENSP_mappings:AF-Q8N612-F1.A&PANTHER:PTHR21705&PANTHER:PTHR21705:SF4							
Gene3D:1.20.1070.10&AFDB-ENSP_mappings:AF-Q8WZ94-F1.A&Pfam:PF13853&Prints:PR00245&I							
Gene3D:1.	0&1	0&1					
						614460	
Gene3D:1.10.287.70&Gene3D:2.60.40.1400&PDB-ENSP_mapping						600937	Diabetes, permanent
Gene3D:2.60.40.10&Pfam:PF07679&PROSITE_profiles:PS50835&PANTHER:PTHR13817&PANTHER:							
Gene3D:1.20.1070.10&AFDB-ENSP_mappings:AF-Q8NH70-F1.A&Pfam:PF13853&PROSITE_profiles:							
AFDB-ENSI	0&1	0&1					
AFDB-ENSP_mappings:AF-Q1KMD3-F1.A&Low_complexity_(Seg):seg&MobiDB_lite:mobidb-lite&PA							
AFDB-ENSI	0&1	0&1				602180	
AFDB-ENSP_mapping		1				606064	
AFDB-ENSI	0&1	0&1				602110	
PDB-ENSP_	0&0&1	1&1&1	27434533			209901	Bardet-Biedl syndrom
PDB-ENSP_	0&1	0&1				603891	
CDD:cd174	0&1	0&1				604988	
Gene3D:2.	0&1	0&1				610084	Essential tremor, here
Gene3D:2.	0&1	0&1		PanelApp-I	maternally imprinted (paternal allele expres		
Gene3D:1.	0&0&1	0&1&1	25450229			608774	
PDB-ENSP_	0&1	1&1	31824661			191350	Myasthenic syndrome
Gene3D:1.20.1070.10&AFDB-ENSP_mappings:AF-Q8NH80-F1.A&Pfam:PF13853&PROSITE_profiles:							
Gene3D:2.60.40.10&/		1				608630	Gaze palsy, familial hc
AFDB-ENSP_mappings:AF-P0C8F1-F1.A&Gene3D:2.10.60.10							
Gene3D:3.90.1480.20&AFDB-ENSP_mappings:AF-Q11206-F1.A&P						104240	
AFDB-ENSP_mappings:AF-Q9BSD3-F1.A&Pfam:PF15319&PANTHE						614085	
Gene3D:2.	0&1	0&1				608974	
AFDB-ENSP_mappings:AF-Q6EIG7-F1.A&PANTHER:PTHR22802&P/						613579	
Pfam:PF15	0&1	0&1				180990	
Pfam:PF15	0&1	0&1				180989	
Gene3D:1.25.40.70&Pfam:PF00613&PROSITE_profiles:PS51545&I						609001	
Gene3D:2.	0&1	1&1					
Gene3D:3.40.50.300&AFDB-ENSP_mappings:AF-Q9UBJ2-F1.A&Pfa						601081	
Gene3D:1.20.5.1160&AFDB-ENSP_mappings:AF-P41219-F1.A&Coi						170710	{Amyotrophic lateral s

AFDB-ENSI	0&1&1	0&1&1					615104	
AFDB-ENSI	0&1	1&1					602153	Monilethrix, 158000 (
AFDB-ENSI	0&1	0&1					148042	Pachyonychia congeni
PDB-ENSP_mappings:			1	23741632			190151	?Lethal congenital cor
Gene3D:2.60.40.630&PDB-ENSP_mappings:4y5u.A&PDB-ENSP_m							601512	
Gene3D:1.	0&1	0&1					603143	
AFDB-ENSP_mappings:AF-Q96JM4-F1.A&PANTHER:PTHR10877:SF184&PANTHER:PTHR10877								
Gene3D:2.30.29.30&AFDB-ENSP_mappings:AF-Q6ZV73-F1.A&Pfar							613520	
Gene3D:1.20.900.10&AFDB-ENSP_mappings:AF-Q6ZV73-F1.A&Pfa							613520	
AFDB-ENSP_mappings:AF-Q6ZV73-F1.A							613520	
Gene3D:1.	0&1	0&1					603351	
AFDB-ENSI	0&1	0&1					613103	
Gene3D:3.40.50.300&Pfam:PF12781&PANTHER:PTHR45703&PAN							605884	Spermatogenic failure
PDB-ENSP_mappings:7mqa.NS&AFDB-ENSP_mappings:AF-Q8IY37							617362	Neurodevelopmental
AFDB-ENSP_mappings:AF-Q8N3T6-F1.A&Pfam:PF16070&PANTHER:PTHR13388&PANTHER:PTHR13								
AFDB-ENSP_mappings:AF-Q9UJ78-F1.A&PANTHER:PTHR45736:SF							616443	
Gene3D:1.	0&1	0&1					182360	
AFDB-ENSP_mappings:AF-Q5TBA9-F10.A&AFDB-ENSP_mappings:/							614818	
Gene3D:6.	0&0&1	1&1&1		25741868&24033266&2472832'			600185	Fanconi anemia, comp
AFDB-ENSP_mappings:AF-Q6N069-F1.A&PANTHER:PTHR22767:SF							619497	
AFDB-ENSI	0&1	1&1					608102	Ceroid lipofuscinosis,
AFDB-ENSP_mappings:AF-O75592-F10.A&AFDB-ENSP_mappings:/							610392	
Gene3D:1.10.287.110&AFDB-ENSP_mappings:AF-Q13217-F1.A&P							601184	Ataxia, combined cere
AFDB-ENSP_mappings:AF-B2RN74-F1.A&PROSITE_profiles:PS50262&PANTHER:PTHR24242&PANTH								
PANTHER:PTHR24118&PANTHER:PTHR24118:SF56&Gene3D:1.25.40.20&Pfam:PF12796&Superfam								
Gene3D:1.	0&1	0&1						
PDB-ENSP_mappings:1rnf.A&PDB-ENSP_mappings:1rnf.B&PDB-EN							601030	
AFDB-ENSP_mappings:AF-P07998-F1.A&PANTHER:PTHR11437:SF.							180440	
PDB-ENSP_	0&1	0&1					605272	
PDB-ENSP_mappings:5xnp.A&PDB-ENSP_mappings:5xnp.B&PDB-I							612811	
AFDB-ENSP_mappings:AF-O00471-F1.A&Pfam:PF07393&PANTHEI							604469	
AFDB-ENSP_mappings:AF-P24278-F1.A&CDD:cd18213&PANTHER:							194541	
AFDB-ENSP_mappings:AF-Q92537-F1.A&PANTHER:PTHR46839&P							616761	
AFDB-ENSI	0&1	0&1					600136	
AFDB-ENSP_mappings:AF-Q9H7Z3-F1.A&Pfam:PF08424&PANTHE							618631	
AFDB-ENSP_mappings:AF-Q96BY7-F1.A&PANTHER:PTHR13190:SF							616226	
PANTHER:I	0&1	0&1					618597	
AFDB-ENSP_mapping:			1		PanelApp-I	maternally	611896	
AFDB-ENSP_mappings:AF-A0A0U1RRK4-F1.A&PANTHER:PTHR14987&PANTHER:PTHR14987:SF3&M								
AFDB-ENSI	0&1	0&1					608570	
AFDB-ENSP_mappings:AF-Q8IVF2-F10.A&AFDB-ENSP_mappings:A							608570	
AFDB-ENSP_mappings:AF-Q8IVF2-F10.A&AFDB-ENSP_mappings:A							608570	
AFDB-ENSI	0&1	0&1					608570	
AFDB-ENSP_mappings:AF-Q8IVF2-F1.A&AFDB-ENSP_mappings:AF							608570	
AFDB-ENSP_mappings:AF-Q8IVF2-F1.A&AFDB-ENSP_mappings:AF							608570	
AFDB-ENSP_mappings:AF-Q8IVF2-F1.A&AFDB-ENSP_mappings:AF							608570	
AFDB-ENSP_mappings:AF-H3BSY2-F1.A&PANTHER:PTHR10881&PANTHER:PTHR10881:SF62&MobiD								
AFDB-ENSI	0&1	0&1						
AFDB-ENSP_mappings:AF-D6RF30-F1.A&PANTHER:PTHR10881&PANTHER:PTHR10881:SF62&Low_c								
AFDB-ENSP_mappings:AF-D6RF30-F1.A&PANTHER:PTHR10881&PANTHER:PTHR10881:SF62								

AFDB-ENSI	0&1	1&1				
PANTHER:PTHR46628						
AFDB-ENSP_mappings:AF-Q9H967-F1.A						
AFDB-ENSI	0&1	1&1			608238	Immunodeficiency 86,
AFDB-ENSI	0&1	0&1			607856	
AFDB-ENSP_mappings:AF-Q12982-F1.A&PANTHER:PTHR12112&P					603292	
AFDB-ENSI	0&1	1&1			608879	Parkinson disease 23,
AFDB-ENSI	0&1	0&1				
AFDB-ENSP_mappings:AF-A6NDK9-F1.A&Coiled-coils_(Ncoils):Coil&Pfam:PF15070&PANTHER:PTHR						
AFDB-ENSI	0&1	0&1			618054	
PANTHER:PTHR47091&PANTHER:PTHR47091:SF1&MobiDB_lite:m					617608	Cardiomyopathy, fam
Gene3D:2.130.10.10&AFDB-ENSP_mappings:AF-Q9NPR2-F1.A&PF					617029	
AFDB-ENSP_mappings:AF-Q96NZ8-F1.A&Pfam:PF00095&Gene3D:					608021	
Gene3D:2.130.10.10&AFDB-ENSP_mappings:AF-Q96KV7-F1.A&PA					618290	
PDB-ENSP_mappings:7ole.K&AFDB-ENSP_mappings:AF-Q9Y4R8-F					611140	You-Hoover-Fong sync
Gene3D:2.60.120.920&AFDB-ENSP_mappings:AF-Q6PJ21-F1.A&Pf					611659	
Gene3D:2.40.30.10&AFDB-ENSP_mappings:AF-Q92901-F1.A&Pfar					617416	Cardiomyopathy, dilat
AFDB-ENSP_mappings:AF-Q2KHN1-F1.A&MobiDB_lite:mobidb-lite						
AFDB-ENSI	0&0&1	1&1&1	21413889&22903357&3208207		608107	Neutrophilic dermato
AFDB-ENSP_mapping	1&1		23505238&9668175&15024744		608107	Neutrophilic dermato
AFDB-ENSP_mappings:AF-Q9H305-F1.A&Low_complexity_(Seg):s					610503	
AFDB-ENSI	0&1	0&1			618686	
AFDB-ENSI	0&1	0&1			182890	
Gene3D:2.60.40.1120&AFDB-ENSP_mappings:AF-Q15155-F1.A&P					609157	
Gene3D:1.	0&1	1&1			160745	Megacystis-microcolo
PDB-ENSP_mappings:5xf7.A&AFI			30651579		618588	
PDB-ENSP_mappings:5xf7.A&AFDB-ENSP_mappings:AF-Q8N807-F					618588	
PANTHER:I	0&1	0&1	26135620		603334	
AFDB-ENSP_mappings:AF-Q8NDV7-F1.A&PANTHER:PTHR13020&I					610739	?Epilepsy, familial adu
AFDB-ENSP_mappings:AF-F8W1W9-F1.A&Pfam:PF06409&PANTHER:PTHR15438&PANTHER:PTHR1						
Gene3D:3.30.160.60&AFDB-ENSP_mappings:AF-Q14586-F1.A&PA					604752	
AFDB-ENSP_mappings:AF-O75113-F1.A&PANTHER:PTHR12876&P					619138	
AFDB-ENSP_mappings:AF-Q9NSC2-F1.A&Lo			geneimpri	predicted (	602218	Townes-Brocks syndro
AFDB-ENSP_mappings:AF-Q9BSU1-F1.A&PANTHER:PTHR13465:SF2&PANTHER:PTHR13465&Pfam:F						
AFDB-ENSP_mappings:AF-Q4G0P3-F19.A&AFDB-ENSP_mappings:/					610812	Ciliary dyskinesia, prin
AFDB-ENSI	0&1	0&1			610812	Ciliary dyskinesia, prin
Gene3D:3.10.100.10&AFDB-ENSP_mappings:AF-Q7Z443-F1.A&Pfa					607895	
PDB-ENSP_	0&1	1&1			605584	Retinitis pigmentosa 8
Gene3D:3.90.226.10&AFDB-ENSP_mappings:AF-Q8N8U2-F1.A&Pf					618816	
AFDB-ENSP_mappings:AF-Q3SXM5-F1.A&CDD:cd05356&PANTHEI					619067	
AFDB-ENSP_mappings:AF-Q2VPK5-F1.A&HAMAP:MF_03054&Pfar					617057	Microcephaly, facial d
AFDB-ENSI	0&1	0&1			617057	Microcephaly, facial d
AFDB-ENSP_mappings:AF-Q92508-F1.A&PANTHER:PTHR13167&P					611184	Lymphatic malformati
AFDB-ENSP_mappings:AF-Q8IUW3-F1.A&PANTHER:PTHR15326&PANTHER:PTHR15326:SF7						
HAMAP:MF_03013&Pfam:PF13236&PROSITE_profiles:PS51823&I					616184	
AFDB-ENSI	0&1	0&1			604682	
AFDB-ENSP_mappings:AF-P51575-F1.A&PANTHER:PTHR10125:SF					600845	
PDB-ENSP_	0&1	1&1			131370	Glycogen storage dise
AFDB-ENSP_mappings:AF-Q969V4-F1.A&PANTHER:PTHR19960&P					609002	
Gene3D:1.20.1250.20&AFDB-ENSP_mappings:AF-P14672-F1.A&P/					138190	

PDB-ENSP_mappings:6w6w.A&PDB-ENSP_mappings:6w6w.B&AFI				613129	Cerebroretinal microa
AFDB-ENSI	0&1	0&1		604057	
AFDB-ENSI	0&1	0&1		615562	
PDB-ENSP_mappings:4fm9.A&PDB-ENSP_mappings:5gwk.A&PDB-				126430	DNA topoisomerase II
AFDB-ENSI	0&1	0&1			
AFDB-ENSI	0&1	0&1			
AFDB-ENSI	0&1&1	1&1&1	31816047		
Gene3D:4.	0&1	0&1		122561	
PDB-ENSP_	1	1		116946	
AFDB-ENSP_mappings:AF-Q9Y2D9-F1.A&Low_complexity_(Seg):se				613907	
AFDB-ENSI	0&1	1&1	25741868	609883	Bardet-Biedl syndrom
Gene3D:4.	0&1&1	1&1&1	21886588	604067	Bradyopsia, 608415 (3
AFDB-ENSP_mapping:		1		138254	
PROSITE_profiles:PS50002&CDD:cd12068&PANTHER:PTHR22692&PANTHER:PTHR22692:SF16&Pfa					
AFDB-ENSI	0&1	0&1		602291	Ciliary dyskinesia, prin
AFDB-ENSP_mapping:		1		607643	Retinitis pigmentosa 3
AFDB-ENSP_mappings:AF-Q5FWF5-F1.A&Coiled-coils_(Ncoils):Coi				609674	
Gene3D:2.	0&1	0&1		606730	
AFDB-ENSP_mappings:AF-Q9C0F0-F1.A&PANTHER:PTHR13578&P				615115	Bainbridge-Ropers syr
Gene3D:3.60.40.10&AFDB-ENSP_mappings:AF-O60346-F1.A&Pfar				609396	
Low_complexity_(Seg		1		604760	
AFDB-ENSP_mapping:		1		612034	Cortical dysplasia, con
AFDB-ENSI	0&1	0&1		608239	
AFDB-ENSP_mappings:AF-Q9UPR6-F1.A&PANTHER:PTHR45762&F				619284	
AFDB-ENSP_mappings:AF-Q13111-F1.A&Pfam:PF15539&PANTHEI				601246	
AFDB-ENSI	0&1	0&1		613247	
AFDB-ENSI	0&1&1	0&1&1	29267878	613247	
AFDB-ENSP_mappings:AF-Q96Q06-F1.A&PANTHER:PTHR47538				613247	
AFDB-ENSI	0&1	0&1		613247	
PDB-ENSP_mappings:		1		605490	CODAS syndrome, 60(
AFDB-ENSI	0&1	0&1		142765	
PDB-ENSP_	0&1	0&1		605872	
AFDB-ENSP_mappings:AF-Q9NQ55-F1.A&PANTHER:PTHR12661:SI				607793	
AFDB-ENSI	0&1	0&1		617522	
PDB-ENSP_mappings:		1		171640	Spondyloenchondrody
				616826	
AFDB-ENSI	0&1	0&1		608841	Anterior segment dys
AFDB-ENSI	0&1	0&1		617096	
Gene3D:2.	0&1	0&1		600310	Pseudoachondroplasia
PDB-ENSP_mappings:6tzt.A&PDB-ENSP_mappings:6tzt.C&PDB-EN				606942	
AFDB-ENSP_mappings:AF-Q8IWZ8-F1.A&PANTHER:PTHR23340&P				607992	
Gene3D:3.	0&1	0&1		603983	
Gene3D:3.	0&1	0&1			
Gene3D:3.30.160.60&AFDB-ENSP_mappings:AF-P0DPD5-F1.A&Pfam:PF00096&PROSITE_patterns:F					
AFDB-ENSI	0&0&1	1&1&1	25741868&19406966&2334933	602716	Nephrotic syndrome,
AFDB-ENSP_mappings:AF-Q9H8S5-F1.A&PANTHER:PTHR10177&PANTHER:PTHR10177:SF257&Gen					
Low_complexity_(Seg):seg&PANTHER:PTHR13161:SF4&PANTHER:				618532	
AFDB-ENSP_mappings:AF-Q96B18-F1.A&PANTHER:PTHR15919&P				611112	
Gene3D:1.10.3860.10&PDB-ENSP_mappings:5llm.A&PDB-ENSP_n				109190	
PDB-ENSP_mappings:1hcn.B&PDB-ENSP_mappings:1hrp.B&PDB-E				118860	

AFDB-ENSP_mappings:AF-Q6NT52-F1.A&CDD:cd00069&PANTHER					608824	
MobiDB_lite:mobidb-lite&PANTHER:PTHR11515&PANTHER:PTHR					608823	
AFDB-ENSI	0&1	0&1			607972	
AFDB-ENSP_mappings:AF-Q96IR2-F1.A&PANTHER:PTHR24377&PANTHER:PTHR24377&PANTHER:P						
Gene3D:3.	0&1	0&1			618194	
AFDB-ENSI	0&1	1&1	25741868		605158	Anterior segment dys
AFDB-ENSP_mappings:AF-Q09MP3-F1.A&Pfam:PF15696&PANTHER:PTHR15361&PANTHER:PTHR15						
AFDB-ENSI	0&1	1&1			114010	Developmental and e
AFDB-ENSI	0&1	0&1			605638	
Gene3D:3.40.50.410&PANTHER:PTHR24020&PANTHER:PTHR2402					617693	
Gene3D:3.90.550.50&AFDB-ENSP_mappings:AF-P0DN25-F1.A&PA					612723	
AFDB-ENSP_mappings:AF-P42704-F1.A&PANTHER:PTHR46669:SF					607544	Mitochondrial comple
AFDB-ENSI	0&1	0&1				
AFDB-ENSP_mappings:AF-Q70CQ2-F12.A					615295	
AFDB-ENSI	0&1	0&1			610995	
AFDB-ENSI	0&1	0&1			602434	
AFDB-ENSP_mappings:AF-P16383-F1.A&Pfam:PF07842&PANTHEF					189901	
AFDB-ENSP_mapping:		1			603336	
AFDB-ENSP_mappings:AF-Q86YJ6-F1.A&CDD:cd01560&PANTHER:					611261	
AFDB-ENSP_mappings:AF-Q96BQ3-F1.A&PANTHER:PTHR24103&PANTHER:PTHR24103:SF621						
PANTHER:PTHR24147&PANTHER:PTHR24147:SF50&MobiDB_lite:mobidb-lite&MobiDB_lite:mobid						
Gene3D:1.10.287.70&AFDB-ENSP_mappings:AF-Q16281-F1.A&Pf					600053	Achromatopsia 2, 216
AFDB-ENSI	0&1	0&1				
AFDB-ENSP_mappings:AF-P0CG38-F1.A&Coiled-coils_(Ncoils):Coil&Pfam:PF14915&PANTHER:PTHR						
AFDB-ENSP_mappings:AF-P0CG38-F1.A&PANTHER:PTHR24118&PANTHER:PTHR24118:SF56						
AFDB-ENSP_mapping:		1			610450	
AFDB-ENSP_mapping:		1	25741868		602536	Martsolf syndrome 2,
PROSITE_p	0&1	1&1	25741868		161650	Nemaline myopathy 2
AFDB-ENSI	0&1	1&1	33462484&34226706		615129	
Gene3D:1.25.40.10&AFDB-ENSP_mappings:AF-Q7Z4L5-F1.A&PAN					612014	Short-rib thoracic dys
AFDB-ENSI	0&1	0&1	26090850		615336	
AFDB-ENSI	0&1	0&1				
Gene3D:2.60.40.10&Pfam:PF07679&PROSITE_profiles:PS50835&I					188840	Muscular dystrophy, l
AFDB-ENSP_mapping:		1			615796	Spermatogenic failure
AFDB-ENSI	0&1	0&1			600655	
AFDB-ENSI	0&1	0&1			618510	
PDB-ENSP_mappings:1h4a.X&PDB-ENSP_mappings:1hk0.X&PDB-I					123690	Cataract 4, multiple ty
AFDB-ENSP_mappings:AF-Q9Y276-F1.A&CDD:cd00009&PANTHER					603647	GRACILE syndrome, 60
AFDB-ENSP_mappings:AF-Q8N1F8-F1.A&PANTHER:PTHR15454&P					607172	
Low_comp	0&1	0&1				
Pfam:PF15290&PANTHER:PTHR16208&PANTHER:PTHR16208:SF1					604942	
AFDB-ENSP_mappings:AF-P25100-F1.A					104219	
AFDB-ENSI	0&1	0&1			618171	
Gene3D:3.	0&1&1&1	0&1&1&1		PanelApp-I imprinted	123855	
Gene3D:2.	0&1	0&1			604871	
PDB-ENSP_mappings:6hts.H&AFDB-ENSP_mappings:AF-Q9H9F9-F					619730	
Low_complexity_(Seg):seg&PANTHER:PTHR24012&PANTHER:PTHR24012:SF466&TIGRFAM:TIGR01						
AFDB-ENSP_mappings:AF-Q02383-F1.A&Pfam:PF05474&PANTHE					182141	
AFDB-ENSP_mappings:AF-Q9BR11-F1.A&PANTHER:PTHR31569&PANTHER:PTHR31569:SF0						
AFDB-ENSP_mappings:AF-Q9P2E3-F1.A&CDD:cd18808&PANTHER					618931	Immunodeficiency 91

PDB-ENSP_mappings:7o9k.G&PDB-ENSP_mappings:7odt.t&PDB-E				610919	
AFDB-ENSI	0&1	0&1			
AFDB-ENSI	0&1	0&1		617937	
AFDB-ENSP_mappings:AF-P48552-F1.A&PANTHER:PTHR15088&M				602490	?Congenital anomalie
PDB-ENSP_mappings:2i50.A&AFDB-ENSP_mappings:AF-Q9Y5T5-F				604735	
Gene3D:1.20.140.150&AFDB-ENSP_mappings:AF-P56748-F1.A&P				611231	
AFDB-ENSP_mappings:AF-O60287-F1.A&PANTHER:PTHR13500				608865	
PDB-ENSP_mappings:5eh1.A&PDB-ENSP_mappings:6e3k.E&PDB-I				147569	Immunodeficiency 28,
PDB-ENSP_	0&0&1	1&1&1	25741868&21056700&2403326(	176261	Jervell and Lange-Niel
PANTHER:I	0&1	1&1	29197136	604803	
Gene3D:2.130.10.10{		1&1	25558065	601245	
AFDB-ENSI	0&1	1&1	31142279&27837419&3116592!	605705	Developmental and e
Gene3D:2.	0&1	0&1	25358694	601475	
AFDB-ENSI	0&1	0&1		601659	
PDB-ENSP_	0&1	1&1	29875488&30072576	605717	
PDB-ENSP_	0&0&1	0&1&1	30554804&22942708&2889428:	606588	
AFDB-ENSP_mappings:AF-P0C7N4-F1.A&PANTHER:PTHR38498					
AFDB-ENSP_mappings:AF-Q9UFD9-F1.A&PANTHER:PTHR14234&P				612699	
Gene3D:3.	0&0&1	1&1&1	29334895&17708757&1969300!	606810	{Schizophrenia, susce
PANTHER:PTHR11267		1		602054	Tetralogy of Fallot, 18
AFDB-ENSP_mapping:		1	25741868	616830	Metabolic encephalor
PANTHER:PTHR24043&PANTHER:PTHR24043:SF5&MobiDB_lite:m				613619	Van den Ende-Gupta s
AFDB-ENSI	0&1	0&1			
AFDB-ENSI	0&1	1&1	33462484	614140	Teebi hypertelorism s
Gene3D:1.10.8.270&Pfam:PF00566&PROSITE_profiles:PS50086&I				611417	
AFDB-ENSP_mappings:AF-Q9BY89-F1.A&PANTHER:PTHR22042&PANTHER:PTHR22042:SF3					
AFDB-ENSI	0&1	0&1			
Gene3D:1.20.1730.10&AFDB-ENSP_mappings:AF-Q9NY91-F1.A&P				618633	
Gene3D:1.25.40.90&PDB-ENSP_mappings:1elk.A&PDB-ENSP_map				604700	?Immunodeficiency 8!
PANTHER:PTHR22793&PANTHER:PTHR22793:SF6				606078	?Immunodeficiency 6(
AFDB-ENSP_mapping:		1	19666484	606269	Immunodeficiency, co
Gene3D:1.10.555.10&Pfam:PF00620&PROSITE_profiles:PS50238{				609405	
AFDB-ENSI	0&1	0&1			
Gene3D:2.60.40.60&Pfam:PF00028&PROSITE_profiles:PS50268&I				604523	Lymphatic malformati
AFDB-ENSI	0&1	0&1			
Gene3D:3.40.20.10&AFDB-ENSP_mappings:AF-O15195-F1.A&Pfar				619666	
Gene3D:3.	0&1	0&1		604049	
Gene3D:1.10.238.10{		1&1	24631775&22705208	600163	Ventricular fibrillation
AFDB-ENSI	0&1	1&1		608112	Developmental and e
AFDB-ENSP_mappings:AF-Q8TBZ5-F1.A&PROSITE_profiles:PS50157&PANTHER:PTHR24393:SF37&P					
AFDB-ENSI	0&1&1	0&1&1		612402	
AFDB-ENSP_mappings:AF-Q9BXS9-F1.A&Pfam:PF00916&PANTHEI				610068	
Gene3D:2.60.120.26C		1	20556798	150325	Nephrotic syndrome,
AFDB-ENSP_mappings:AF-Q04912-F1.A&CDD:cd01179&PIRSF:PIR				600168	{Nasopharyngeal carci
Gene3D:3.10.20.90&Pfam:PF00788&PROSITE_profiles:PS50200&I				605082	
Gene3D:2.	0&1	0&1		608560	
Gene3D:2.30.180.10&AFDB-ENSI			33536631	608560	
AFDB-ENSP_mapping:		1	33536631	608011	
Gene3D:6.10.140.1060&Pfam:PF12781&PANTHER:PTHR10676&P				603340	
Gene3D:2.60.40.10&PDB-ENSP_mappings:4u7m.A&AFDB-ENSP_n				608868	

AFDB-ENSP_mappings:AF-A0A1B0GTH6-F1.A&PANTHER:PTHR35825					
Gene3D:1.20.5.48208		1		609929	
AFDB-ENSI	0&1	1&1		617089	Anauxetic dysplasia 3,
Coiled-coil	0&1	1&1	33462484	602500	
Coiled-coil	0&1	1&1		602500	
Pfam:PF15	0&1	0&1			
Gene3D:2.60.40.10&AFDB-ENSP_mappings:AF-Q8IWZ5-F1.A&Pfam:PF00041&PROSITE_profiles:PS!					
AFDB-ENSI	0&1	0&1		612200	
PDB-ENSP_	0&1	0&1		114852	
AFDB-ENSP_mappings:AF-P49788-F1.A&PIRSF:PIRSF011132&PIRS				605090	
AFDB-ENSP_mappings:AF-Q8WWY8-F1.A&CDD:cd00707&PANTHE				607365	Hypotrichosis 7, 6043
PANTHER:I	0&1	0&1		158372	
PANTHER:I	0&1	0&1		158372	
MobiDB_lite:mobidb-lite				158372	
MobiDB_li	0&1&1	0&1&1		158372	
Low_comp	0&1	0&1		158372	
Low_comp	0&1	0&1		158372	
Low_comp	0&1	0&1		158372	
Low_comp	0&1	0&1		158372	
Low_comp	0&1	0&1		158372	
AFDB-ENSP_mappings:AF-O14976-F1.A&PANTHER:PTHR23172&P				602052	
AFDB-ENSP_mappings:AF-O14924-F1.A&PANTHER:PTHR45945&P				602512	
Gene3D:2.	0&1	0&1		618899	
PDB-ENSP_	0&1	0&1	33230300	608785	
AFDB-ENSP_mappings:AF-Q8IYL2-F1.A&PANTHER:PTHR21210				614309	
Gene3D:1.	0&1	0&1		606645	
Gene3D:4.10.400.108		1		605236	Preeclampsia/eclamps
Gene3D:2.	0&1	0&1	27652281&30696812	616417	
PDB-ENSP_mappings:6kxs.J&PDB-ENSP_mappings:6lx3.J&PDB-EN				147790	
PDB-ENSP_mappings:2w3c.A&AFDB-ENSP_mappings:AF-O60763-				603344	
PANTHER:I	0&0&1	1&1&1	25741868	607830	Fraser syndrome 1, 21
AFDB-ENSI	0&1	0&1		617485	?Microcephaly 18, pri
AFDB-ENSI	0&1	1&1	32042192&32888494&3138789	611652	
PANTHER:PTHR24217&PANTHER:PTHR24217:SF9					
Gene3D:1.	0&1	0&1		131230	{Pregnancy loss, recur
AFDB-ENSP_mappings:AF-Q9Y3S2-F1.A&Pfam:PF06524&PANTHE				609550	
AFDB-ENSP_mappings:AF-P53367-F1.A&PANTHER:PTHR12141&P/				605928	
Gene3D:2.70.170.10&PDB-ENSP_mappings:5bkf.E&PDB-ENSP_ma				138492	Hyperekplexia 2, 6146
AFDB-ENSP_mappings:AF-P49767-F1.A&PANTHER:PTHR12025&P/				601528	Lymphatic malformati
AFDB-ENSI	0&1	0&1		147576	
Gene3D:3.	0&1	0&1	20041164	609760	
AFDB-ENSI	0&0&1	0&1&1	28007035	610697	
Gene3D:3.40.640.10&Gene3D:3.90.1150.10&AFDB-ENSP_mappin				612471	[Beta-aminoisobutyric
AFDB-ENSP_mappings:AF-Q6KC79-F1.A&AFDB-ENSP_mappings:AI				608667	Cornelia de Lange syn
PANTHER:PTHR14492				614571	Orofaciodigital syndro
PDB-ENSP_mappings:5a9q.A&PD			32118046	606694	?Atrial fibrillation 15,
AFDB-ENSP_mappings:AF-Q8WV60-F1.A&PANTHER:PTHR14700				615484	
AFDB-ENSP_mappings:AF-A5PLL1-F1.A&PANTHER:PTHR24156&P/				618581	
Gene3D:1.20.1070.10&AFDB-ENSP_mappings:AF-Q8NGU9-F1.A&Prints:PR00237&PROSITE_profiles					
AFDB-ENSI	0&1	0&1		602118	Pilarowski-Bjornsson s

AFDB-ENSI	0&1	0&1		612141	
AFDB-ENSP_mappings:AF-Q9HCS5-F1.A&CDD:cd17107&Pfam:PF0				612141	
AFDB-ENSP_mapping:		1&1	27208211&33230300	613446	Short-rib thoracic dys
Gene3D:2.	0&1&1	1&1&1	25741868&24033266&3101689	612570	Macular degeneratior
Gene3D:3.	0&0&1	1&1&1	26314886&27153395&2355531	604040	Nijmegen breakage sy
AFDB-ENSP_mapping:		1		604417	CHOPS syndrome, 61
AFDB-ENSP_mappings:AF-P49069-F1.A&PANTHER:PTHR15026&P				601118	
AFDB-ENSP_mappings:AF-Q9NZM6-F1.A&Transmembrane_helice				604669	
AFDB-ENSI	0&1	0&1		606333	
AFDB-ENSP_mappings:AF-Q9Y5F2-F1.A&CDD:cd11304&Gene3D:2				606337	
AFDB-ENSI	0&1	0&1		606290	
AFDB-ENSI	0&1	0&1		603058	
AFDB-ENSP_mappings:AF-Q9UN71-F1.A&CDD:cd11304&Gene3D:				603058	
PDB-ENSP_mappings:1h0z.A&Gene3D:3.30.60.30&AFDB-ENSP_m				605010	Netherton syndrome,
PDB-ENSP_mappings:		1&1	19131662&19330901&19559392&30409984&27247849&267914		
Gene3D:2.70.170.10		1		137143	
AFDB-ENSP_mappings:AF-P2856			23986905	600714	
AFDB-ENSP_mappings:AF-Q17RY0-F1.A&PANTHER:PTHR12566&P				610607	
PANTHER:I	0&1	0&1		615813	
Gene3D:3.40.640.10&Gene3D:3.90.1150.10&AFDB-ENSP_mappin				614683	[?Phosphohydroxylysi
PANTHER:I	0&1	0&1		608304	
AFDB-ENSI	0&0&1	1&1&1		603716	Hypoparathyroidism,
AFDB-ENSP_mappings:AF-P49790-F1.A&PANTHER:PTHR23193&P				603948	
AFDB-ENSI	0&1	1&1	26563541	611259	
AFDB-ENSP_mappings:AF-Q14676-F1.A&PANTHER:PTHR23196&P				607593	
AFDB-ENSI	0&1	0&1		613917	
AFDB-ENSI	0&1	0&1		142840	{Psoriasis susceptibilit
AFDB-ENSI	0&1	0&1		142840	{Psoriasis susceptibilit
AFDB-ENSI	0&1	0&1		142840	{Psoriasis susceptibilit
AFDB-ENSI	0&1	0&1	28062682	142840	{Psoriasis susceptibilit
AFDB-ENSI	0&1	0&1		142840	{Psoriasis susceptibilit
AFDB-ENSI	0&1&1	0&1&1		142840	{Psoriasis susceptibilit
PDB-ENSP_	0&1	0&1	30886520	142840	{Psoriasis susceptibilit
PDB-ENSP_	0&1	0&1	30886520	142840	{Psoriasis susceptibilit
PDB-ENSP_	0&1	0&1		142840	{Psoriasis susceptibilit
PDB-ENSP_	0&1	0&1	30886520	142840	{Psoriasis susceptibilit
PDB-ENSP_	0&1	0&1	30123242	142840	{Psoriasis susceptibilit
PDB-ENSP_	0&1	0&1	34506591	142840	{Psoriasis susceptibilit
PDB-ENSP_	0&1&1	0&1&1	29760565	142840	{Psoriasis susceptibilit
PDB-ENSP_	0&1&1	0&1&1	20604894&27081498&3509599	142840	{Psoriasis susceptibilit
PDB-ENSP_	0&1&1	0&1&1		142840	{Psoriasis susceptibilit
PDB-ENSP_	0&1	1&1	31217584&34445011&3459403	142840	{Psoriasis susceptibilit
PDB-ENSP_	0&1	0&1		142840	{Psoriasis susceptibilit
PDB-ENSP_	0&1&1	0&1&1	30344693	142840	{Psoriasis susceptibilit
Gene3D:3.	0&1	0&1		142857	{Multiple sclerosis, su
PDB-ENSP_mappings:		1	16960798	147267	{Diabetes, type 1, sus
PDB-ENSP_	1	1		604100	
PDB-ENSP_mappings:3pfs.A&PDB-ENSP_mappings:3pfs.B&AFDB-I				616856	
PDB-ENSP_mappings:		1	21552306	607845	
Gene3D:3.30.1490.1C		1	29216386&22973298	603968	Xeroderma pigmento

Gene3D:2.60.120.970		1		112265	
				113810	Neuropathy, heredita
PDB-ENSP_mappings:6n4b.R&AFDB-ENSP_mappings:AF-P21554-F				114610	
AFDB-ENSP_mappings:AF-Q8WV93-F1.A&Pfam:PF03969&PANTHI				617469	
AFDB-ENSP_mappings:AF-P29966-F1.A&Low_complexity_(Seg):se				177061	
Gene3D:2.60.120.260		1	25741868	156225	Muscular dystrophy, l
AFDB-ENSI	0&1	0&1		603237	
AFDB-ENSP_mappings:AF-Q86VZ4-F1.A&PANTHER:PTHR46876&MobiDB_lite:mobidb-lite					
AFDB-ENSP_mappings:AF-Q4ZHG4-F1.A&PANTHER:PTHR23197&F				609991	
AFDB-ENSP_mappings:AF-Q5SW24-F1.A&MobiDB_lite:mobidb-lit				608966	
PDB-ENSP_	0&1	1&1		611061	Raine syndrome, 2597
AFDB-ENSP_mappings:AF-Q6ZMB5-F1.A&PANTHER:PTHR23423&PANTHER:PTHR23423:SF59&Pfam					
AFDB-ENSI	0&1	1&1	25741868	613653	Spastic paraplegia 48,
AFDB-ENSI	0&1	1&1		600253	?Retinitis pigmentosa
AFDB-ENSI	0&0&1	1&1&1	25741868&24033266&2763730	603339	Ciliary dyskinesia, prin
PDB-ENSP_	0&1	0&1		604854	
AFDB-ENSP_mappings:AF-Q8NBF6-F1.A&Pfam:PF09794&PROSITE				612927	
PANTHER:PTHR45727:SF3&PANTHER:PTHR45727&Pfam:PF16414				608010	[Ezetimibe, nonrespor
AFDB-ENSI	0&1	1&1		613022	?Oxoglutarate dehydr
AFDB-ENSI	0&1	0&1		619419	
AFDB-ENSP_mappings:AF-Q68CZ2-F1.A&PANTHER:PTHR45734:SF				606825	
AFDB-ENSI	0&1	0&1			
AFDB-ENSI	0&1	1&1		610317	
AFDB-ENSP_mappings:AF-O75128-F1.A&PANTHER:PTHR47008				610317	
Gene3D:6.10.140.140&AFDB-ENSP_mappings:AF-Q96JC4-F1.A&PI				617444	
Gene3D:1.25.40.10&AFDB-ENSP_mappings:AF-O75344-F1.A&PAN				604839	
AFDB-ENSP_mappings:AF-Q9UIG0-F1.A&PANTHER:PTHR46802&L				605681	
AFDB-ENSI	0&1	0&1		619829	
AFDB-ENSI	0&1	0&1		614774	
AFDB-ENSI	0&1	0&1		606534	
AFDB-ENSP_mappings:AF-Q6ZVC0-F1.A&Pfam:PF15452&PANTHE				615477	
AFDB-ENSP_mappings:AF-Q02505-F1.A&AFDB-ENSP_mappings:AI				158371	
PDB-ENSP_mappings:6fxk.A&PDB-ENSP_mappings:6fxm.A&PDB-E				603066	Lysyl hydroxylase 3 de
Gene3D:3.30.750.24	1&1		25741868&24033266&1974433	605646	Deafness, autosomal r
AFDB-ENSI	0&1	0&1		603502	
AFDB-ENSP_mappings:AF-Q86UW7-F1.A&PANTHER:PTHR12166:S				609978	
PDB-ENSP_	0&1	0&1		604741	Bile acid synthesis def
AFDB-ENSI	0&0&1	1&1&1	30595370	610934	Premature ovarian fai
Gene3D:3.30.160.60&AFDB-ENSP_mappings:AF-Q8N393-F1.A&PANTHER:PTHR24376&PANTHER:P					
AFDB-ENSI	0&1	1&1		606551	Esophageal squamous
PDB-ENSP_mappings:7a6h.N&PDB-ENSP_mappings:7ae1.N&PDB-				187280	
AFDB-ENSP_mapping		1		607350	
AFDB-ENSP_mappings:AF-P00390-F1.A&Low_complexity_(Seg):se				138300	Hemolytic anemia due
AFDB-ENSP_mappings:AF-Q6WKZ4-F1.A&PANTHER:PTHR15746&I				608737	
AFDB-ENSI	0&1	0&1		604782	
Gene3D:3.30.40.10&		1		614649	Ataxia, sensory, 1, aut
PDB-ENSP_mappings:6wm2.P&PDB-ENSP_mappings:6wm3.P&PD				608861	
PDB-ENSP_mappings:1fj2.A&PDB-ENSP_mappings:1fj2.B&Gene3D				605599	
Gene3D:1.20.1090.10&AFDB-ENSP_mappings:AF-Q8IWW8-F1.A&				611083	
AFDB-ENSP_mappings:AF-Q96RS6-F1.A&PANTHER:PTHR21664				606109	

Gene3D:3.40.50.182C		1				605262	Charcot-Marie-Tooth
Gene3D:3.	0&1	0&1					
AFDB-ENSI	0&1	0&1				618640	
AFDB-ENSP_mappings:AF-A6NE52-F1.A&PANTHER:PTHR45532&PANTHER:PTHR45532:SF1							
AFDB-ENSP_mappings:AF-Q14137-F1.A&Low_complexity_(Seg):se						610596	
AFDB-ENSP_mappings:AF-Q6P5W5-F1.A&Pfam:PF02535&PANTHE						607059	Acrodermatitis enterc
AFDB-ENSP_mappings:AF-Q6P5W5-F1.A&PANTHER:PTHR12191&I						607059	Acrodermatitis enterc
PANTHER:I	0&1	0&1				613632	
AFDB-ENSI	0&1&1	0&1&1				613747	
Gene3D:1.20.900.10&AFDB-ENSP_mappings:AF-Q8N4T4-F1.A&PANTHER:PTHR47056&Superfamily							
Gene3D:3.	0&1&1	0&1&1				607009	Hypomagnesemia 1, ir
AFDB-ENSP_mappings:AF-Q8WUY3-F1.A&AFDB-ENSP_mappings:/						610691	
Low_complexity_(Seg):seg&PANTHER:PTHR13902:SF10&PANTHEI						606249	
PDB-ENSP_mappings:		1				610290	{Colorectal cancer, su:
PANTHER:PTHR48001&PANTHER:PTHR48001:SF51&Superfamily:SSF81321							
PDB-ENSP_mappings:		1				602238	Short stature, hearing
AFDB-ENSP_mapping:		1	33728047			516060	
PDB-ENSP_mappings:		1	31267007&34069212&3511857:			516020	
Gene3D:1.10.10.1210&AFDB-ENSP_mappings:AF-Q8N7X4-F1.A&F						300467	
AFDB-ENSI	0&1	0&1				300678	
AFDB-ENSI	0&1	0&1				300731	
AFDB-ENSP_mappings:AF-O95972-F1.A&PANTHER:PTHR11848:SF						300247	Premature ovarian fai
AFDB-ENSI	0&1	0&1				300236	
AFDB-ENSI	0&1	0&1				300235	
AFDB-ENSI	0&1	0&1				300235	
AFDB-ENSP_mappings:AF-P98168-F1.A&PANTHER:PTHR46179&P/						300235	
AFDB-ENSP_mappings:AF-Q96QF7-F1.A&PANTHER:PTHR23099&N						300369	Spermatogenic failure
AFDB-ENSP_mappings:AF-Q8IYN2-F1.A&PANTHER:PTHR14754&PANTHER:PTHR14754:SF11&Pfam:I							
AFDB-ENSI	0&1	0&1				301085	
PANTHER:I	0&1	0&1				300171	
AFDB-ENSP_mappings:AF-Q96PJ5-F1.A&PANTHER:PTHR11481&P/						605876	
PDB-ENSP_mappings:2jvn.A&PDB-ENSP_mappings:2riq.A&PDB-EN						173870	
PDB-ENSP_	0&1	0&1				607613	?Galloway-Mowat syr
PDB-ENSP_mappings:		1				609413	UV-sensitive syndrom
Gene3D:3.	0&1&1	0&1&1		PanelApp-I	maternally	617348	
PDB-ENSP_mappings:7mq8.NJ&PDB-ENSP_mappings:7mq8.NK&P						609221	
AFDB-ENSP_mappings:AF-P60852-F1.A&PANTHER:PTHR23343&P/						195000	Oocyte maturation de
AFDB-ENSI	0&1	0&1				604034	
Gene3D:3.30.65.10&PDB-ENSP_mappings:4cgy.A&PDB-ENSP_ma						601243	?Progressive external
AFDB-ENSP_mappings:AF-Q6NT52-F1.A&PANTHER:PTHR11515:SF						608824	
AFDB-ENSP_mappings:AF-Q9Y2P5-F1.A&CDD:cd05938&PANTHER						603314	
AFDB-ENSP_mappings:AF-A6QL64-F1.A&PANTHER:PTHR24147:SF50&PANTHER:PTHR24147&MobiI							
Gene3D:2.	0&1	0&1				600543	Amyotrophic lateral s(
AFDB-ENSI	0&1	0&1				619045	
AFDB-ENSI	0&1	0&1				609732	
PDB-ENSP_	0&1	0&1				142840	{Psoriasis susceptibilit
AFDB-ENSP_mappings:AF-Q9UJU6-F1.A&PANTHER:PTHR10829&P						610106	
PANTHER:I	0&1	0&1				617208	
						613236	{Thyrotoxic periodic p
						608914	

	0&1	0&1					
						300016	
						171835	
		1		PanelApp-I	paternally	605016	
						611037	Combined oxidative p
	0&0&1	1&1&1	25741868&25525159&2030172:			607444	{Aplastic anemia, susc
Gene3D:2.40.100.10&AFDB-ENSP_mappings:AF-F5H284-F1.A&PIRSF:PIRSF001467&PROSITE_profil							
PROSITE_profiles:PS51316&PANTHER:PTHR14199:SF35&PANTHEI						614003	
AFDB-ENSP_mappings:AF-Q8NG06-F1.A&Low_complexity_(Seg):seg&CDD:cd19780&Gene3D:3.30.:							
AFDB-ENSP_mappings:AF-P55107-F1.A&PROSITE_profiles:PS5136						601361	
AFDB-ENSP_mapping:	1&1		15221801&12369017&1787672:			607608	Niemann-Pick disease
Low_complexity_(Seg):seg&MobiDB_lite:mobidb-lite						615496	Corneal dystrophy, Fu
AFDB-ENSP_mappings:AF-Q53H64-F1.A&PANTHER:PTHR24192&PANTHER:PTHR24192:SF2							
AFDB-ENSP_mappings:AF-Q68EA5-F1.A&PROSITE_profiles:PS50157&PANTHER:PTHR24379:SF9&PA							
AFDB-ENSP_mappings:AF-Q6NT55-F1.A&PANTHER:PTHR24291&P						611495	Ichthyosis, congenital,
PDB-ENSP_mappings:	1&1		22995991&19390652			123690	Cataract 4, multiple ty
AFDB-ENSP_mapping:	0&1					611756	
PANTHER:I	0&1	0&1				300879	
PDB-ENSP_mappings:1jbq.A&PDB-ENSP_mappings:1jbq.B&PDB-E						613381	Thrombosis, hyperhor

MOTIF_PO	GH_INF_P	_SCORE_C	000Gp3_A	Aloft_pred	Del_addAF	inPred_pr	ANN_scor	OGEN2_pr
luysian atrophy, 125370 (3), Autosomal dominant| Congenital hypotonia, epilepsy, developmental c								
luysian atrophy, 125370 (3), Autosomal dominant| Congenital hypotonia, epilepsy, developmental c								
3520 (3), Autosomal dominant| Leukemia, juvenile myelomonocytic, 607785 (3), Autosomal domin								
e 13, 615990 (3), Autosomal recessive| Meckel syndrome 1, 249000 (3), Autosomal recessive| Joub								
1, 135900 (3), Autosomal dominant								
1, 135900 (3), Autosomal dominant								
with}, 601665 (3), Autosomal recessive, Autosomal dominant, Multifactorial								
complexity(Seg):seg								
complexity(Seg):seg								
pings:AF-Q2TBE0-F1.A&Pfam:PF04677&PANTHER:PTHR12072&PANTHER:PTHR12072:SF5&Superfa								
ase, autosomal dominant, 1}, 608320 (3), Autosomal dominant								
ne 1, 229200 (3), Autosomal recessive								
yndrome, 601358 (3), Autosomal dominant| Blepharophimosis-impaired intellectual development s								
recessive 98, 614861 (3), Autosomal recessive| Ectodermal dysplasia 14, hair/tooth type with or w								
syndrome, 600920 (3), Autosomal recessive								
t-onset, autosomal dominant, 169500 (3), Autosomal dominant| Microcephaly 26, primary, autosor								
			0,001797		T	T	0,998161	T
616781 (3), Autosomal recessive					T	T	0,901098	T
				.&.	T	T	0,848948	.&T
					T	D	0,992439	
					T	D	0,998695	T
NTHER:PTHR14516&MobiDB_lite			0,019169	.&.	T	T	0,997698	T&.
			0,002995	.&.	T	T	0,998063	T&.
			0,0002	.&.&.&.&.	T	T	0,982108	.&T&.&T&
fetus and newborn, RH-induced},			0,001398		T	T	0,555831	T
, with fiber-type disproportion, 255310 (3),				.&.&.	T	D	0,996673	.&T&.
			0,001997		T	T	0,998834	T
				.&.	D	D	0,996482	.&T
				.&.	T	T	0,997599	T&T
			0,000998	.&.	T	T	0,815127	T&.
513790 (3), Autosomal recessive			0,000599		T	T	0,963048	T
HER:PTHR10166:SF40&Gene3D:3			0,0002	.&.	T	T	0,998223	.&.

		.&.	T	T	0,110974	T&.
	0,002796		T	T	0,941829	T
	0,001398	.&.&.&.	T	T	0,955348	.&.&T&.
ıary, autosomal recessive, 616402 (3), Autosomal reces			T	T	0,896312	T
		.&.	T	D	0,997877	T&T
	0,01278	.&.&.	T	T	0,981898	.&T&T
			T	T	0,928092	T
;10205 (3), Autosomal dominant| Hajdu-Che		.&.	D	D	0,999439	D&D
	0,165335	.&.	T		0,840816	T&.
						T
		.&.&.&.&.	T	T	0,998996	.&T&.&.&T
lae, 225200 (3), Autosomal recessive| Ectop		.&.&.&.	T	T	0,65521	T&.&.&T
			T	T	0,409562	T
	0,003794		T	T	0,561278	T
		.&.	D	D	0,998389	.&D
iple types, 175800 (3), Autosomal	0,001797		T	T	0,97968	T
		.&.	T	T	0,990019	.&T
ystem], 110700 (3), Autosomal re	0,000399	.&.&.	T	T	0,996884	T&T&.
to activated protein C resistance, 188055 (3)		.&.	T	T	0,209902	T&T
	0,001997	.&.&.	T	T	0,28872	T&.&.
to antithrombin III deficiency, 613118 (3), Au		.&.	T	D	0,9978	.&D
, 613235 (3), Autosomal recessiv	0,003395		T	T	0,992806	T
ary, autosomal recessive, 608716	0,004193		T	T	0,999211	T
	0,0002		T	T	0,952852	
		.&.&.&.&.	D	D	0,990242	.&T&T&T&
	0,002796	.&.&.&.&.	T	T	0,999234	.&.&.&T&T
		.&.	T	T	0,972599	T&T
ımyelinating, 15, 617951 (3), Autosomal recessive			D	D	0,996642	D
			T	T	0,448461	T
		.&.&.&.&.&	T	T	0,992959	.&T&.&.&T
		.&.&.&.&.	T	T	0,984614	.&T&.&.&T
	0,002796	.&.	T	T	0,998	D&D
, 100100 (3), Autosomal recessive		.&.	T	D	0,986642	T&T
		.&.&.&.	T	T	0,956486	T&.&.&T
	0,002196	.&.&.	T	T	0,997739	.&.&.
7302 (3), Autosomal recessive		.&.&.&.	D	D	0,998623	.&D&.&D
/ 10, 619040 (3), Autosomal recessive		.&.&.	D	D	0,997905	.&.&T
		.&.&.	T	D	0,985524	.&.&T
	0,026158	.&.&.&.	T	T	0,484575	T&T&.&.
		.&.	T	T	0,638016	T&.
		.&.				T&.
B_lite:mobidb-lite&MobiDB_lite:	0,001797	.&.&.&.	T	T	0,729784	.&T&T&.
	0,000599		T	T	0,781583	T
		.&.	T	T	0,841536	T&.
	0,000399		T	T	0,987373	T
splasia 4, 614974 (3), Autosomal recessive			D	D	0,963971	T
type 3, 610725 (3), Autosomal re	0,003395	.&.&.	T	T	0,961355	T&.&.
	0,006589	.&.	T	T	0,998495	T&.
	0,002995	.&.&.	T		0,699814	T&T&T
	0,001597		T	T	0,965022	T

			T	T	0,508256	T
al pressure, 1, 236690 (3), Autosomal domin		.&.&.&.	T	T	0,733772	T&T&.&T
	0,002396	.&.&.&.&.&	T	T	0,994215	T&T&.&.&
	0,0002		T	T	0,758443	T
	0,001597		T	T	0,998349	
	0,000399		T	T	0,976459	T
		.&.	T	T	0,147125	T&T
	0,000599		T	T	0,998195	T
	0,005591		T	T	0,440434	T
			T	T	0,787489	T
	0,000399		T	T	0,804085	T
	0,002396		T	T	0,514049	T
	0,003195	.&.&.&.	T	T	0,970203	T&.&T&.
	0,142772	.&.	T	T	0,765852	T&T
	0,78095	.&.	T	T	0,247333	T&T
	0,0002		T	T	0,904196	T
	0,0002	.&.	T	D	0,998483	T&T
		.&.&.	T	D	0,990341	T&.&T
PROSITE_profiles:PS50262&PANT	0,005591	.&.	T	T	0,992215	T&T
	0,000799	.&.&.	T	T	0,711779	T&T&T
		.&.&.	T	T	0,921906	.&.&T
neonatal 2, with or without neurologic featu		.&.&.	D	D	0,998636	.&.&.
PTHR13817:SF10&SMART:SM00	0,002796		T	T	0,998945	
PS50262&PANTHER:PTHR48002&PANTHER:PTHR4800			T	T	0,796915	T
		.&.	T	T	0,069439	T&T
ANTHER:PTHR12381:SF66&PANTHER:PTHR12381			T	T	0,544805	T
	0,000799	.&.&.&.	T	T	0,994718	T&.&T&T
	0,004393		T	T	0,902354	T
	0,0002	.&.&.&.&.	T	D	0,998461	T&T&T&T&
e 1, 209900 (3), Autosomal recessive, Digeni		.&.&.&.	D	D	0,999506	.&D&T&.
			T	D	0,998206	D
		.&.&.&.&.	T	T	0,98754	.&T&.&.&.
editary, 5, 616736 (3), Autosomal	0,000399		T	T	0,919611	T
sed)	0,005192		T	T	0,966699	T
	0,004193		T	T	0,973323	T
, congenital, 13, with tubular agg	0,000599	.&.&.	T	T	0,9634	T&T&.
PS50262&PANTHER:PTHR26453&PANTHER:		.&.&.	T	D	0,994063	T&T&T
orizontal, with progressive scolios	0,000399	.&.	T	T	0,99881	T&.
		.&.	T	T	0,267356	.&T
	0,001198	.&.&.&.&.&	T	T	0,99563	.&T&.&T&
	0,000799	.&.&.	T	T	0,934974	.&T&T
		.&.&.&.	T	T	0,981386	T&T&T&T
	0,011981		T	T	0,927005	T
		.&.	T	T	0,077428	T&T
	0,007788	.&.	T	T	0,082044	.&T
	0,0002	.&.&.	T	T	0,99799	.&T&T
	0,001398	.&.&.&.&.	T	T	0,998358	T&T&T&T&
			D	D	0,99797	D
sclerosis, susceptibility to}, 105400 (3), Auto		.&.	T	T	0,953328	D&T

	0,000399		T	T	0,999518	
3), Autosomal dominant		.&.	T	T	0,99145	D&.
ita 4, 615728 (3), Autosomal dom	0,003794		T	T	0,997278	D
ntractural syndrome 2, 607598 (3	0,000998	.&.&.	T	T	0,808928	T&D&.
	0,0002	.&.&.&.&.8	T	T	0,998266	D&.&D&D8
	0,003994	.&.&.&.&.8	T	T	0,993942	.&.&T&.&.
			T	D	0,999142	T
			T	D	0,996601	T
		.&.&.	D	D	0,998908	T&.&T
	0,000599	.&.&.	T	T	0,157834	T&.&T
	0,004193		T	T	0,986033	T
			T	T	0,872406	T
56, 619515 (3), Autosomal reces	0,010982	.&.	T	T	0,996729	.&T
disorder with brain anomalies and with or w		.&.	D	D	0,996857	T&T
388:SF4	0,000998		T	T	0,995485	T
		.&.	T	T	0,376511	T&T
	0,0002	.&.	D	D	0,992701	.&D
		.&.&.&.&.	T	D	0,99742	.&.&.&T&T
plementation group D1, 605724 (	0,009984	.&.	T	T	0,99907	.&.
	0,000599	.&.	T	T	0,979036	T&.
neuronal, 5, 256731 (3), Autosomal recessiv		.&.&.&.&.8	T	T	0,997732	T&T&T&T8
		.&.	T	T	0,99805	.&.
ebellar and peripheral, with hearing loss and		.&.	T	D	0,998675	D&T
HER:PTHR24242:SF201&Gene3D::	0,010383		T	T	0,246474	T
ily:SSF48403&SMART:SM00248&PROSITE_profiles:PS50088&PROSITE_profiles:PS50297						T
	0,005791	.&.	T		0,464291	T&.
	0,004393	.&.&.	T	T	0,978076	T&T&T
	0,002995	.&.&.&.	T	T	0,983965	T&T&T&T
	0,000998	.&.&.&.&.8	T	T	0,999455	T&.&T&.&T
		.&.&.	D	D	0,988191	T&T&.
	0,002596	.&.&.	T	T	0,978335	T&T&T
		.&.	T	T	0,998482	T&T
			T	T	0,613493	T
		.&.&.&.&.8	T	T	0,999345	T&T&.&T8
	0,005391		T	T	0,998906	T
	0,0002		T	T	0,592368	T
	0,000998	.&.&.&.	T	T	0,836041	.&T&.&T
	0,002995	.&.	T	T	0,991932	T&T
MobiDB_lite:mobidb-lite	0,004193				0,791815	
		.&.	T	T	0,913711	.&T
	0,062101		T	T	0,298914	T
	0,077476		T	T	0,216503	T
	0,205671		T	T	0,99364	T
			T	T	0,990358	T
	0,056709		T	T	0,089582	T
			T	D	0,998886	T
DB_lite:mobidb-lite&MobiDB_lite:mobidb-lite			T		0,900299	T
	0,001597		T	T	0,887596	
complexity_(Seg):seg			T	T	0,991853	
			T	T	0,355735	

	0,003195	.&.&.&.	T	T	0,988355	T&T&T&.
		.&.	T	T	0,997114	.&.
	0,001797	.&.&.	T	T	0,746952	T&T&.
, mycobacteriosis, 619549 (3), Au	0,001797		T	T	0,996298	T
			T	T	0,995878	T
	0,000799	.&.&.&.	T	T	0,996284	T&T&T&.
autosomal recessive, early onset	0,000599	.&.&.&.&.&	T	T	0,986257	T&.&T&.&
	0,0002		T	D	0,988738	T
:10881&PANTHER:PTHR10881:SF44&Low_complexity_(			T	T	0,762228	T
	0,003395		T	T	0,817793	
ilial hypertrophic 27, 618052 (3), Autosomal recessive			T	D	0,999031	T
	0,002796	.&.&.&.&.	T	T	0,970615	T&T&T&.&
			T	T	0,637741	T
		.&.	T	T	0,948464	T&T
drome, 616954 (3), Autosomal recessive			T	D	0,986532	T
		.&.	T	T	0,906429	T&T
ted, 2D, 619371 (3), Autosomal re	0,002796		T	T	0,972145	T
	0,001398	.&.	T	T	0,333434	T&.
sis, acute febrile, 608068 (3), Aut	0,0002	.&.&.&.	T	T	0,996323	T&.&.&.
sis, acute febrile, 608068 (3), Aut	0,0002		T	T	0,939214	T
		.&.&.&.&.&	T	T	0,986167	T&T&T&.&
	0,003794		T	T	0,98451	T
		.&.	T	T	0,992803	D&.
	0,000599	.&.&.	T	T	0,999071	T&T&T
n-intestinal hypoperistalsis syndrome 2, 619		.&.&.&.	T	T	0,998903	.&.&.&D
	0,000998		T	T	0,999063	T
	0,000799	.&.	T	T	0,99813	T&T
	0,003594		T	T	0,990525	T
ılt myoclonic, 6, 618074 (3), Autosomal dom		.&.	T	T	0,434802	.&T
5438:SF4		.&.	T	T	0,947919	.&T
	0,0002		T	T	0,977395	T
			T	T	0,908653	T
ome 1, 107480 (3), Autosomal dominant| To		.&.&.	T	T	0,928068	.&T&T
'F03676	0,002796	.&.&.&.&.	T	T	0,923065	T&.&T&T&
nary, 5, 608647 (3), Autosomal recessive			T	T	0,607147	T
nary, 5, 608647 (3), Autosomal re	0,000799		T	T	0,232872	T
			T		0,560275	T
34, 618220 (3), Autosomal recessive		.&.&.	T	T	0,996321	T&.&.
		.&.&.&.	D	D	0,998887	T&.&.&.
		.&.	T	T	0,998614	T&.
ysmorphism, renal agenesis, and ambiguous		.&.&.	T	T	0,632544	T&.&.
ysmorphism, renal agenesis, and ambiguous		.&.&.&.	T	T	0,59343	T&.&.&T
ion 6, 616843 (3), Autosomal recessive| Dehydrated he			T	T	0,994624	T
			T	T	0,417914	T
		.&.&.	T	T	0,994983	T&T&T
	0,000599		T	T	0,93039	T
			T	T	0,992609	T
ase XIII, 612932 (3), Autosomal r	0,001597	.&.&.	T	T	0,998906	D&.&D
		.&.	T	T	0,978576	T&T
	0,008187	.&.&.	T	T	0,955918	T&T&.

ıngiopathy with calcifications and cysts, 612199 (3), Aut			T	T	0,871135	D
	0,001597		T	T	0,996721	T
			T	T	0,9873	T
, resistance to inhibition of, by amsacrine (3)			T	D	0,999147	T
			T	T	0,814192	T
	0,439896		T	T	0,598644	T
	0,002196	.&.	T	T	0,999177	T&T
	0,0002	.&.&.&.&.	T	T	0,948625	.&.&.&.&T
		.&.&.&.	D	D	0,953948	T&.&.&.
		.&.	T	T	0,986509	T&T
e 13, 615990 (3), Autosomal rece	0,000599	.&.	T	T	0,997514	T&.
3)	0,016174	.&.&.&.	T	T	0,801159	T&.&.&T
	0,002596	.&.	T	T	0,973187	T&.
m:PF07653&Gene3D:2.30.30.40	0,000399	.&.&.			0,899722	T&T&.
nary, 43, 618699 (3), Autosomal dominant			T	T	0,990926	T
30, 607921 (3)		.&.	T	T	0,998798	T&.
	0,001198		T	T	0,998772	T
	0,001797	.&.&.&.	T	T	0,944234	T&.&.&.
ndrome, 615485 (3), Autosomal dominant		.&.	T	T	0,979646	T&.
			T	D	0,99711	T
	0,001597	.&.&.	T	T	0,992839	T&T&.
nplex, with other brain malforma	0,0002	.&.&.&.	T	T	0,978861	T&T&T&T
	0,004193		T		0,711545	T
			T	T	0,783248	T
			T	T	0,968253	T
	0,122404	.&.	T	T	0,994651	T&.
	0,167732	.&.	T	T	0,994671	T&.
	0,000998	.&.	T	T	0,992572	T&.
	0,003195	.&.	T	T	0,1126	T&.
0373 (3), Autosomal recessive	0,003994	.&.&.&.&.	T	T	0,977478	T&T&.&.&
	0,000799	.&.&.&.	T	T	0,995733	T&T&.&T
	0,021366	.&.&.&.&.&	T	T	0,046632	.&.&T&.&T
	0,001997	.&.&.&.	T	T	0,758952	.&.&T&.
	0,0002	.&.&.&.&.&	T	T	0,994938	T&T&.&.&
ysplasia with immune dysregulati	0,001997	.&.&.&.&.	T	T	0,996547	D&D&D&D
		.&.&.&.&.	T	D	0,99612	.&T&.&T&
genesis 8, 617319 (3), Autosomal	0,004792	.&.	T	T	0,930242	.&T
	0,0002	.&.	T	T	0,867819	T&T
a, 177170 (3), Autosomal dominant| Carpal		.&.&.	D	D	0,999483	.&D&.
	0,000599	.&.&.	T	T	0,996146	T&.&.
	0,0002	.&.	T	T	0,999093	T&T
	0,005791	.&.	T	T	0,876215	T&.
		.&.	T	T	0,300638	T&.
PS00028&PROSITE_profiles:PS50	0,002796				0,279296	
type 1, 256300 (3), Autosomal re	0,000599	.&.	T	T	0,99817	T&.
e3D:1.10.472.10		.&.	T	T	0,988349	T&.
	0,001797	.&.&.	T	T	0,972468	T&.&.
	0,001198	.&.	T	T	0,996593	T&T
	0,000998	.&.&.	T	T	0,951374	T&.&T
			T	T	0,241685	T

	0,00599	.&.	T	T	0,987908	.&.
		.&.	T	T	0,480415	.&.
	0,004792	.&.&.	T	T	0,924294	T&.&.
THR24377:SF625&PANTHER:PTH	0,001597	.&.	T	T	0,7984	T&T
	0,0002	.&.	T	T	0,881204	.&T
genesis 7, with sclerocornea, 269	0,013778		T	T	0,990718	T
5361:SF6	0,000998		T	T	0,903265	T
pileptic encephalopathy 50, 6164	0,000998	.&.	T	T	0,83613	T&T
	0,000399		T	T	0,820158	
	0,0002	.&.&.&.&.8	T	D	0,99919	.&T&.&T&
	0,005192				0,728372	T
ex IV deficiency, nuclear type 5, (French-Canadian), 220:			T	T	0,924333	T
		.&.	T	T	0,994442	.&T
		.&.	T	T	0,992848	T&T
	0,000599		T	T	0,957974	T
	0,0002		T	T	0,86985	
	0,004393	.&.	T	T	0,991316	T&.
	0,004193	.&.	T	T	0,989552	T&T
		.&.&.	T	D	0,969715	.&.&T
			T	T	0,994755	T
ɔ-lite		.&.	T	T	0,996449	T&.
900 (3), Autosomal recessive		.&.&.&.	D	D	0,995656	T&.&T&.
	0,707069		T	T	0,946702	T
24118&PANTHER:PTHR24118:SF!	0,708067		T	T	0,641127	T
	0,695088		T	T	0,949831	T
	0,002995		T	T	0,903318	T
619420 (3), Autosomal recessive	0,008986	.&.&.	T	T	0,676891	T&.&.
!, autosomal recessive, 256030 (3	0,003395	.&.&.&.&.8	T	T	0,733073	.&.&T&.&T
	0,001198		T	T	0,996656	T
plasia 4 with or without polydactyly, 613819 (3), Autosc			T	T	0,897524	T
	0,000799	.&.	T	T	0,984885	D&.
	0,001997		T	T	0,993157	T
imb-girdle, autosomal recessive 10, 608807		.&.&.&.&.8	T	T	0,913112	.&.&.&.&.8
: 34, 618153 (3), Autosomal recessive			T	T	0,559807	T
	0,017772	.&.&.&.	T	T	0,993701	T&T&T&T
	0,005391		T	T	0,997237	T
ypes, 115700 (3), Autosomal dominant			T	T	0,656625	T
03358 (3), Autosomal recessive| Mitochondı		.&.&.&.&.8	D	D	0,996212	D&D&D&D
			T	T	0,996223	
	0,003395		T	T	0,41854	T
	0,001398	.&.&.&.	T	T	0,984673	.&T&.&.
			T	D	0,997798	T
	0,009585	.&.&.	T	T	0,998616	T&T&.
	0,007388	.&.	T	T	0,244746	T&T
	0,0002		T	T	0,997863	T
	0,0002		T	T	0,998525	T
628	0,000799	.&.	T	T	0,995429	T&T
	0,002196		T	T	0,97998	T
	0,002196	.&.	T	T	0,976112	T&T
and hyperinflammation, 619644	0,000799	.&.	D	T	0,99665	D&D

		.&.	T	T	0,227721	T&.
	0,001997		T	T	0,952435	T
	0,007188		T	T	0,941745	T
s of kidney and urinary tract 3, 61	0,002796	.&.&.	T	T	0,998908	T&T&T
		.&.&.&.	T	D	0,973611	.&T&T&T
			D	D	0,998066	T
			T	T	0,946268	T
, mycobacteriosis, 614889 (3), Autosomal re		.&.	T	T	0,359927	T&.
sen syndrome 2, 612347 (3), Aut	0,673922	.&.&.&.&.&	T	T	0,891096	T&T&T&T&
	0,008786		T	T	0,999569	T
			D	D	0,980422	T
pileptic encephalopathy 30, 6163	0,166534	.&.	T	T	0,980069	T&.
	0,572085	.&.	T	T	0,998739	T&T
	0,591853	.&.&.&.	T	T	0,526013	.&.&T&.
	0,269169	.&.&.	T	T	0,840752	.&T&.
	0,165136	.&.&.	T	T	0,637184	.&D&.
	0,546126		T		0,94929	T
	0,000599		T	T	0,618206	T
otibility to, 4}, 600850 (3), Autosc	0,905551	.&.&.&.	T	T	0,856331	T&.&.&T
7500 (3), Autosomal dominant| DiGeorge sy		.&.	D	D	0,997873	.&.
nyopathic crises, recurrent, with	0,001997	.&.&.&.&.&	T	T	0,998617	.&.&.&.&T
syndrome, 600920 (3), Autosomal recessive		.&.	T	T	0,998185	.&.
		.&.	T	T	0,619676	T&T
yndrome 1, 145420 (3), Autosom	0,000799	.&.&.&.&.	T	T	0,99662	.&.&.&.&.
	0,004193	.&.&.	T	T	0,997639	.&.&T
		.&.	T	T	0,626417	T&T
	0,002796		T	T	0,78357	T
	0,009385		T	T	0,993257	T
5 and autoimmunity, 619510 (3), Autosomal		.&.&.&.&.&	T	D	0,999037	T&.&T&T&
6, 618847 (3), Autosomal recessive		.&.&.&.&.&	T	D	0,997472	T&.&T&T&
mmon variable, 4, 613494 (3), Au	0,001997		T	T	0,984576	T
	0,002396	.&.&.&.	T	T	0,974215	.&.&T&.
		.&.&.	T	T	0,641528	T&T&.
ion 9, 619319 (3), Autosomal dominant			T	D	0,997598	T
	0,001198	.&.&.	T	T	0,99718	T&T&.
		.&.&.&.	T	D	0,994288	T&.&T&T
	0,000399	.&.	T	T	0,902039	T&.
, familial, 1, 603829 (3)| Heart block, progre		.&.&.&.&.&	D	D	0,999133	.&.&.&.&.&
pileptic encephalopathy 68, 6182	0,001597	.&.&.	T	T	0,998424	.&D&T
ANTHER:PTHR24393:SF37&PANTHER:PTHR2		.&.&.	T	T	0,979878	T&T&T
		.&.	T	T	0,90755	T&T
		.&.&.&.&.	T	T	0,828531	.&T&T&.&
type 5, with or without ocular ab	0,001597	.&.	T	T	0,589275	T&T
inoma, susceptibility to, 3}, 617075 (3), Autc		.&.&.	T	T	0,995819	T&.&.
	0,001597	.&.&.&.&.	T	T	0,999295	.&.&.&T&.
	0,000998		T	T	0,600984	T
	0,000998		T	T	0,962382	T
	0,000998	.&.	T	T	0,986286	T&.
						T
	0,0002	.&.	T	T	0,898318	T&.

					0,713636	
	0,000399		T	T	0,993516	T
618853 (3), Autosomal recessive	0,004393	.&.	T	T	0,999175	T&T
	0,001997	.&.&.&.	T	T	0,606897	T&.&.&.
	0,004593	.&.&.&.	T	T	0,963113	T&.&.&.
	0,000799	.&.&.&.	T	T	0,99537	.&.&T&.
50853&PANTHER:PTHR25465&PANTHER:PTHR25465:SI			T	T	0,998936	T
	0,0002	.&.&.&.	T	T	0,998141	.&.&.&.
	0,003994	.&.&.	T	T	0,990014	T&T&T
		.&.	T	T	0,497451	T&.
79 (3), Autosomal recessive| Woolly hair, autosomal re			D	D	0,998155	D
	0,001198	.&.&.&.	T	T	0,844977	.&.&.&.
	0,008786	.&.	T	T	0,180526	.&.
	0,002995	.&.	T	T	0,885373	.&.
		.&.	T	T	0,953692	.&.
		.&.	T	T	0,611939	.&.
	0,003395	.&.	T	T	0,717398	.&.
		.&.	T	T	0,3657	.&.
		.&.	T	T	0,732541	.&.
		.&.	T	T	0,759424	.&.
		.&.&.	T	T	0,261164	T&.&T
	0,000399	.&.&.	T	T	0,616653	T&.&T
	0,000599	.&.	T	T	0,996556	T&.
	0,004393	.&.	T	T	0,998964	D&.
	0,002396	.&.	T	T	0,957019	.&T
		.&.	T	T	0,987597	T&T
sia 5, 614595 (3)	0,002196	.&.&.&.&.&	T	T	0,972812	D&.&.&D&
	0,004992	.&.&.&.&.&	T	T	0,606363	.&.&.&T&T
		.&.&.	T	D	0,995115	D&D&D
		.&.	D	T	0,364554	T&.
19000 (3), Autosomal recessive	0,001398		T	T	0,999538	
mary, autosomal dominant, 6175	0,0002	.&.	T	T	0,602104	T&T
	0,002396	.&.&.	T	T	0,99911	T&T&.
		.&.&.&.	T	D	0,998426	.&.&T&.
rent, susceptibility to, 3}, 614391	0,0002	.&.&.	T	T	0,998488	T&T&T
	0,0002	.&.&.&.	T	T	0,998503	T&.&.&T
		.&.&.&.	T	T	0,712395	T&T&.&.
519 (3), Autosomal recessive		.&.&.	D	D	0,99884	D&.&D
ion 4, 615907 (3), Autosomal don	0,0002		T	T	0,995722	T
			T	T	0,972083	T
			T	T	0,836721	T
	0,006789		T	T	0,996876	T
acid, urinary excretion of], 210100 (3), Aut		.&.&.	T	T	0,614156	T&.&.
drome 1, 122470 (3), Autosomal dominant		.&.	D	D	0,997216	T&.
me VI, 277170 (3), Autosomal recessive| Jo		.&.&.	T	D	0,995793	T&T&T
615770 (3), Autosomal recessive	0,0002	.&.&.	T	D	0,999473	D&.&T
		.&.&.&.	T	D	0,992372	.&T&T&.
	0,004393		T	T	0,887819	T
s:PS50262&PANTHER:PTHR24241&PANTHER:PTHR2424			T	D	0,994807	T
syndrome, 617682 (3), Autosomal dominant		.&.	T	D	0,999484	T&T

	0,000399	.&.	D	D	0,99633	D&D
		.&.	D	T	0,99865	D&D
plasia 13 with or without polydac	0,001797	.&.&.&.	T	T	0,999088	T&T&.&T
ı, early-onset, 616118 (3), Autosc	0,003594	.&.&.&.&.	T	T	0,998339	D&.&D&D&
ndrome-like disorder, 613078 (3)	0,000399	.&.&.&.&.&	T	T	0,997527	.&.&.&T&T
5368 (3), Autosomal dominant		.&.	T	T	0,968002	T&.
		.&.	D	D	0,997937	T&.
	0,0002	.&.&.&.	T	T	0,997142	.&.&D&.
	0,002396		T	T	0,998356	T
		.&.	T	D	0,991018	T&.
		.&.	T	T	0,99516	.&T
	0,0002	.&.	T	T	0,966753	.&T
	0,003994	.&.	T	T	0,995962	.&T
256500 (3), Autosomal recessive		.&.&.&.	T	T	0,508024	.&.&.&T
-77&24944790&15726497&2526	0,003994		T	T	0,925585	T
	0,000599	.&.	T	T	0,942681	D&.
	0,001797		T	T	0,990759	T
		.&.&.&.	T	D	0,987728	T&.&.&T
	0,001797		T	T	0,991016	
nuria], 615011 (3)		.&.	D	D	0,957755	T&.
	0,002796	.&.&.&.&.&	T	T	0,662299	.&.&.&T&.
familial isolated 2, 618883 (3), Au	0,003395		T	T	0,912868	T
		.&.&.	T	D	0,996878	.&.&T
	0,002596	.&.&.	T	T	0,999242	T&.&T
			T	T	0,696354	T
	0,009585		T		0,443701	T
y 1}, 177900 (3), Multifactorial| {	0,777356	.&.&.	T	T	0,279491	T&.&.
y 1}, 177900 (3), Multifactorial| {	0,757588	.&.&.	T	T	0,288215	T&.&.
y 1}, 177900 (3), Multifactorial| {	0,777356	.&.&.	T	T	0,205089	T&.&.
y 1}, 177900 (3), Multifactorial| {	0,141573	.&.&.	T	T	0,359102	T&.&.
y 1}, 177900 (3), Multifactorial| {HIV-1 viren		.&.&.	T	T	0,190008	T&.&.
y 1}, 177900 (3), Multifactorial| {	0,763978	.&.&.	T	T	0,310319	T&.&.
y 1}, 177900 (3), Multifactorial| {	0,749601	.&.&.	T	T	0,693825	T&.&.
y 1}, 177900 (3), Multifactorial| {	0,721246	.&.&.	T	T	0,198033	T&.&.
y 1}, 177900 (3), Multifactorial| {	0,147564	.&.&.	T	T	0,41265	T&.&.
y 1}, 177900 (3), Multifactorial| {	0,770767	.&.&.	T	T	0,314457	T&.&.
y 1}, 177900 (3), Multifactorial| {HIV-1 viren		.&.&.	T	T	0,29004	T&.&.
y 1}, 177900 (3), Multifactorial| {	0,386182	.&.&.	T	T	0,735313	T&.&.
y 1}, 177900 (3), Multifactorial| {	0,760184	.&.&.	T	T	0,277333	T&.&.
y 1}, 177900 (3), Multifactorial| {	0,371206	.&.&.	T	T	0,509896	T&.&.
y 1}, 177900 (3), Multifactorial| {	0,429113	.&.&.	T	T	0,572888	T&.&.
y 1}, 177900 (3), Multifactorial| {	0,256789	.&.&.	T	T	0,70924	T&.&.
y 1}, 177900 (3), Multifactorial| {	0,136581	.&.	T	T	0,902534	T&.
y 1}, 177900 (3), Multifactorial| {	0,165735	.&.	T	T	0,331367	T&.
sceptibility to, 1}, 126200 (3), Mu	0,17512		T	T	0,959239	T
ceptibility to}, 222100 (2), Autosc	0,00619	.&.	T	T	0,996762	D&D
		.&.&.&.&.&	T	D	0,997261	.&.&T&.&.
	0,005591	.&.&.	T	T	0,995433	T&.&.
	0,005591		T	T	0,985744	T
sum, variant type, 278750 (3), Au	0,004593	.&.	T	T	0,994502	T&.

	0,011182		T	T	0,924952	T
ry sensory and autonomic, type V	0,0002	.&.	T	T	0,085673	T&.
		.&.&.&.&.&	T	T	0,979742	T&T&T&.&
	0,010982		T	T	0,526202	T
			T	D	0,949456	T
imb-girdle, autosomal recessive 2	0,0002	.&.&.	T	T	0,99893	.&T&T
	0,000599	.&.&.&.&.&	T	T	0,998123	.&.&.&T&.
			T	T	0,238093	T
	0,000599		T	T	0,918246	T
		.&.	T	T	0,427265	T&.
775 (3), Autosomal recessive	0,003395		T	T	0,998886	T
:PF03619&Transmembrane_heli	0,000399	.&.&.&.&.	T	T	0,766072	T&.&.&.&.
autosomal recessive, 613647 (3), Autosoma		.&.	T	T	0,660502	T&T
85, 618345 (3), Autosomal reces	0,001398	.&.&.	T	T	0,381638	.&T&.
nary, 7, with or without situs inve	0,004792	.&.&.	T	T	0,997755	.&.&T
	0,015775	.&.	T	T	0,592046	T&.
		.&.&.&.	D	D	0,999089	T&T&.&T
nse to], 617966 (3)| [Low density lipoprotein		.&.&.&.	T	D	0,997913	D&T&.&.
ogenase deficiency, 203740 (3), A	0,002396	.&.&.&.&.&	T	T	0,998809	T&.&.&.&T
			T	T	0,203902	T
		.&.&.	T	D	0,99508	D&.&T
	0,005192	.&.&.&.&.	T	T	0,998444	T&.&.&.&T
	0,004193	.&.&.&.	T	T	0,996097	T&T&T&.
	0,0002	.&.&.&.&.&	T	T	0,897588	T&.&.&.&.
	0,003395	.&.&.	T	T	0,700009	T&T&T
		.&.&.&.	T	T	0,962538	.&.&.&T
		.&.	T	T	0,982114	T&T
	0,004193		T	T	0,922794	T
	0,002196	.&.	T	T	0,658047	T&.
	0,0002	.&.&.&.	T	T	0,921083	T&.&.&.
	0,0002	.&.	T	T	0,999354	T&T
		.&.	T	T	0,612391	.&.
eficiency, 612394 (3), Autosomal recessive			D	D	0,998671	D
recessive 4, with enlarged vestibu	0,008586	.&.	D	T	0,998755	D&D
		.&.&.&.&.&	T	T	0,999077	T&.&.&.&T
		.&.&.&.	T	T	0,99688	.&T&.&T
ect, congenital, 2, 235555 (3), Autosomal re		.&.	T	T	0,981537	.&T
lure 5, 611548 (3), Autosomal do	0,004393	.&.&.&.	T	T	0,130129	.&.&T&.
THR24376:SF185&Superfamily:SSF57667		.&.	T	T	0,925456	.&T
cell carcinoma, somatic, 133239	0,002995	.&.&.	T	T	0,99907	D&D&.
		.&.	D	D	0,982467	T&T
	0,007188		T	T	0,703974	T
to glutathione reductase deficie	0,000399	.&.&.&.	T	T	0,996568	T&.&.&.
	0,003195	.&.&.&.	T	T	0,998493	.&T&T&.
	0,002596	.&.	T	T	0,994747	T&T
osomal dominant, 608984 (3), Au	0,002596	.&.&.&.	T	T	0,931672	.&T&T&.
		.&.&.&.&.	T	T	0,995119	.&T&T&T&
		.&.&.&.&.&	T	T	0,666914	T&T&.&.&
		.&.	T	T	0,723254	T&.
		.&.	T	D	0,987975	T&.

disease, type 4D, 601455 (3), Autosomal rec		.&.&.&.	T	T	0,910877	T&.&T&.
			T	T	0,646854	T
	0,005192		T	T	0,650686	T
	0,000799		T	T	0,987601	T
			T	D	0,992275	T
pathica, 201100 (3), Autosomal recessive		.&.	D	D	0,999039	.&D
pathica, 201100 (3), Autosomal recessive		.&.&.	T	T	0,997011	.&T&.
	0,483826		T		0,894345	T
	0,005591	.&.	T	T	0,547968	T&.
:SSF48065&Superfamily:SSF5072	0,000399		T	T	0,989845	T
ntestinal, 602014 (3), Autosomal recessive		.&.&.	T	T	0,747772	.&.&T
		.&.&.	T	T	0,058065	T&T&T
	0,000998	.&.	T	T	0,294306	T&.
sceptibility to, 1}, 608812 (3)			T	T	0,948914	T
		.&.	T	T	0,306191	T&.
loss, retinitis pigmentosa, and di	0,004393	.&.&.&.&.	T	T	0,980052	T&.&T&.&
						T
	0,002119		T	T	0,797605	T
			D	D	0,999076	D
			T	T	0,375816	T
lure 4, 300510 (3), X-linked| Ovarian dysgenesis 2, 300			T	T	0,881268	T
	0,004768		T	T	0,963162	T
			T	T	0,187491	T
	0,184371		T	T	0,344905	T
	0,184371		T	T	0,598335	T
, X-linked, 4, 301077 (3), X-linked	0,00053	.&.	T	T	0,819627	T&T
PF04538	0,000265	.&.&.	T	T	0,991665	T&T&T
	0,002119	.&.&.	T	T	0,624695	T&T&.
		.&.	T	D	0,998274	D&T
			T	T	0,999195	T
			T	D	0,988275	T
ndrome 8, 618349 (3), Autosomal	0,003594		T	T	0,998245	D
e 1, 600630 (3), Autosomal reces	0,001398		T	T	0,999347	D
	0,000799	.&.	T	T	0,998299	T&T
		.&.&.	T	T	0,99459	T&.&.
fect 1, 615774 (3), Autosomal re	0,001398		T	T	0,996491	T
	0,0002	.&.	T	T	0,999371	.&T
ophthalmoplegia with mitochondrial DNA d		.&.&.	T	D	0,942014	T&T&.
	0,003594		T	T	0,998163	
		.&.	D	D	0,995855	T&.
DB_lite:mobidb-lite		.&.	T	D	0,979993	.&T
clerosis 19, 615515 (3), Autosomal dominan		.&.&.	T	D	0,98886	T&D&.
	0,002396	.&.&.	T	T	0,690608	T&.&.
		.&.	T	T	0,990758	.&T
y 1}, 177900 (3), Multifactorial| {	0,177716	.&.&.	T	T	0,672694	T&.&.
	0,001997	.&.&.&.&.	T	T	0,996116	T&.&.&.&T
	0,003594	.&.	T	T	0,812602	.&T
aralysis, susceptibility to, 2}, 613239 (3), Autosomal dominant						

hosphorylation deficiency 28, 616794 (3), Autosomal recessive					
:eptibility to}, 609135 (3)| Shwachman-Diamond syndrome 1, 260400 (3), Autosomal recessive					
es:PS50072&PANTHER:PTHR11071&PANTHER:PTHR11(			D	0,943739	
		.&.&.			.&.&.
160.60&Pfam:PF00643&SMART:SM00336&!		Recessive	D	0,973653	
, type B, 607616 (3), Autosomal recessive| N		.&.	D	0,986658	.&.
chs endothelial, 8, 615523 (3), Au	0,003794			0,753083	
	0,005192	.&.&.		0,881686	.&.&.
ANTHER:PTHR24379&Gene3D:3.3	0,002796	.&.&.	T	0,964937	.&.&.
, autosomal recessive 5, 604777 (3), Autosor		Recessive&	D	0,996539	.&.
ypes, 115700 (3), Autosomal dom	0,003594	Recessive	D	0,992156	
	0,000998	.&.	D	0,993701	.&.
		.&.&.&.	D	0,989777	.&.&.&.
nocysteinemic, 236200 (3), Autosomal recessive| Homocystinuria, B6-responsive and nonresponsiv					

n-phred_co	THMM_pr	GERP++_RS	TEx_V8_ge	Ex_V8_tiss	oCanyon_s	_eQTL_tar	erpro_dom	LRT_pred
delay, and digital anomalies, 618494 (3), Autosomal dominant								
delay, and digital anomalies, 618494 (3), Autosomal dominant								
ant, Somatic mutation| Neurofibromatosis, familial spinal, 162210 (3), Autosomal dominant| Neurc								
ert syndrome 28, 617121 (3), Autosomal recessive								
mily:SSF54197								
yndrome, 619293 (3), Autosomal dominant								
ithout hypohidrosis, 618180 (3), Autosomal recessive								
nal dominant, 619179 (3), Autosomal dominant								
1,260635		1,74			0,500303			N
1,916105	T	5,03			0,475262		TOG_dom	D
0,231081	D&D	-3,39			0,944919		.&TNFR/N	N
1,470419	T	1,22			2,09E-06			D
4,114949	T	3,96			0,864485		Protein-ar	D
1,026314	D&T	-0,586			0,999999		.&.	N
5,892872	T&T	5,44			1		von_Wille	N
0,403352	T&T&T&.&	-1,14			0,999462		.&.&.&.&.	N
0,198871	T	-2,49			0,000555			
2,546033	D&D&D	3,27			0,996024		.&.&.	N
4,153065	D	5,94			1		Zinc_finge	D
3,901141	.&.	5,74			0,99995		ATP-grasp_	D
3,341542	T&.	5,34			1		.&.	
1,003495	D&D	2,29			0,149715		.&.	N
0,401282	D	0,696			0,989464		Membrane	N
4,264661	T&.	5,42			1		von_Wille	D

0,042251	T&T	-1,16		0,999649	.&.	N
1,400123	T	3,16		0,000203		N
0,649302	T&T&T&.	1,26		1,44E-05	.&.&.&.	N
0,680188	T	1,43		0,087104		N
4,353606	T&T	4,58		0,919856	.&.	N
2,5551	T&T&T	1,08		0,005205	.&.&.	N
0,341798	D	-1,61		0,134208		N
6,66215	D&.	5,71		1	EGF-like_d	U
	.&.	2,35		0,000494	F-BAR_domain&F-BAI	
	T					
4,382471	.&.&.&.&.	4,97		1	.&.&.&.&.	N
0,012091	T&T&T&T	-6,2		1	.&.&.&.	
0,005091	T	-6,74		0,035335		
1,91488	T	1,71		2,43E-05		
7,372608	D&T	4,65		1	Transduce	D
2,819632	T	5,88		0,999993		D
1,664934	T&T	3,02		0,999914	.&.	N
2,666896	T&T&T	2,98		0,983135	.&.&.	
0,014485	T&T	-4,25		0,003819	.&.	N
0,069548	T&T&T	-4		0,009923	.&.&.	N
7,677833	.&D	5,74		1	Serpin_do	D
3,269162	T	5,99		0,500877	Sushi/SCR/	N
7,793392	T	4,33		0,855008	IQ_motif&	D
0,46448	D	0,671		1,4E-05		
7,007331	T&T&T&T8	5,86		1	.&.&.&.&.	D
11,21586	T&T&T&T8	4,78		1	Protein_ki	D
0,466113	T&T	1,19		0,990216	.&.	N
11,02302	T	5,93		0,999999	Aminoacyl	D
1,406529	T	4,87		0,999349		N
0,760916	T&T&T&T8	0,438		0,004342	.&Immuno	N
2,414016	T&T&T&.&	4,07		0,066124	.&Dbl_hon	D
5,178898	.&D	4,96		0,999984	Zona_pellu	D
4,317633	T&.	5,73		0,999813	GPCR&_rh	D
1,314418	D&D&D&.	2,13		0,798892	.&.&.&.	D
4,567511	T&.&.	4,77		0,974396	.&.&.	D
16,07842	.&T&T&.	5,72		1	.&AAA+_A	D
8,702797	T&T&T	5,82		1	.&.&.	D
2,692589	T&T&T	5,7		0,999875	.&.&.	N
0,24317	.&T&T&T	-3,17	AL117339. Brain_Spin	1,13E-05	.&.&.&.	
0,100729	T&T	-3,48		1	.&.	N
	T&T				.&.	
0,00767	.&.&.&.	-4,56		2,45E-05	.&.&.&.	N
0,247105	T	-3,81		1		N
0,783748	T&T	1,69		0,000119	.&.	N
1,943971	D	5,12		0,999797		N
3,642084	T	5,17		1		D
0,637581	T&T&T	1,79		0,000614	.&.&.	N
2,04154	T&T	0,268		0,000226	.&.	U
	.&.&.	-1,95		0,004497	.&.&.	
1,445948	T	4,04		0,662764		N

0,050241	D	-5,22		0,999994		N
0,035617	T&T&T&T	-7,96		0,785827	.&.&.&.	N
3,256845	T&.&.&.&.	5,01		0,999674	.&.&.&.&.‹	N
0,040473	T	-4,95		0,999564		N
5,768074	T	5,06		0,99977		D
1,448807	T	3,94		0,375198		N
0,173514	D&D	0,537		0,762383	Amidohydı	N
2,110352	T	3,33		0,097611		N
0,166758	D	0,867		0,315819		U
0,170718	T	-1,23		0,016162		U
0,045139	D	-6,43		1	Lamin_tail	N
0,039077	T	-5,68	LMNTD2& Artery_Tib	1		N
0,626452	T&T&T&T	2,75		0,083777	.&.&.&.	N
0,002551	T&T	-6,48		0,999998	.&.	
0,354555	T&T	-0,865		0,35295	.&.	U
0,003176	D	-8,91		1	U_box_doı	N
5,373145	.&T	3,57		0,018675	GPCR&_rh	N
2,918273	T&T&T	5,11		0,99802	.&.&.	D
1,75955	.&T	4,37		0,672184	GPCR&_rh	D
1,02284	.&.&.	2,54		9,09E-05	GPCR&_rh	N
2,768253	T&T&T	4,26		1	.&.&.	
6,011242	D&D&D	4,92		1	.&.&.	D
0,865606	T	1,83		0,84553		
0,649689	T	1,22		1,69E-06	GPCR&_rhodopsin-lik	
0,058215	.&T	2,55		3,43E-06	GPCR&_rh	N
0,594385	T	2,49		1		N
2,928272	D&T&D&D	5,38		1	.&.&.&.	U
0,095832	D	-4,9		0,999995		
1,817264	T&T&.&T&	-0,184		0,999746	.&.&.&.&.	N
6,684632	T&T&T&D	4,47		0,999877	.&.&.&.	
3,492756	D	3,02		1		N
0,561519	T&T&T&T‹	-1,52		0,963418	.&.&.&.&.	N
2,449559	D	5,52		0,999703		D
0,73672	T	-4,14		0,964614	Immunoglı	U
0,991803	D	0,761		0,385633	Serine-thrı	N
1,6608	D&D&.	4,59		0,999972	.&.&.	D
2,060151	.&.&T	1,24		0,003115	GPCR&_rh	N
5,853138	T&T	3,73		0,452905	Immunoglı	N
0,525131	.&D	-1,53		2,45E-06	.&.	
2,607148	T&T&T&T‹	4,78		0,994387	.&.&.&.&.‹	N
1,329279	.&.&.	3,32		0,409966	.&.&.	N
1,086066	T&T&T&T	2,1		1	CUB_domı	N
1,29459	T	0,64		0,999423		N
0,205124	T&T	-0,165		1,7E-06	.&.	
0,225271	T&.	-1,39		9,92E-05	.&.	
4,113292	T&T&T	3,82		0,0001	Phosphoin	N
4,650083	T&T&T&T‹	4,39		0,191829	Pleckstrin_	N
3,530369	D	4,59		0,232632	ABC_trans	D
1,838559	D&.	3,98		0,999795	Intermediı	N

6,749927	T	5,23	1		D
3,001184	T&.	2,52	0,998646	Intermedia	U
2,009386	D	-0,249	0,944437	Intermedia	D
2,493237	T&D&T	5,44	1	.&.&.	N
3,142016	T&T&T&T8	4,55	1	STAT_tran:	D
6,881378	D&.&D&D.	5,91	1	.&.&Sterile	D
6,621136	T	5,36	1		
8,029325	T	4,98	1	Pleckstrin_	D
6,280196	T&T&T	5,79	0,836646	Dbl_homo	N
0,00925	T&T&T	-2,77	0,999759	.&.&.	N
3,280609	T	4,16	0,999864	2'-5'-oligo:	U
0,496546	T	-2,43	0,999906		N
6,047201	.&.	3,72	0,01799	Dynein_he	D
4,45413	T&T	5,3	0,99999	DEAD/DEA	D
8,789569	T	5	0,999966	Transmem	D
0,088446	T&T	-2,09	0,004569	.&.	N
6,581741	D&D	5,19	1	.&Cation-t	D
5,170664	.&.&.&.&.	3,79	1	.&.&.&.&.	N
9,334969	T&T	5,6	1	.&.	D
1,470158	T&T	4,43	0,999892	.&.	D
0,500743	.&.&.&.&.8	1,92	1	.&.&.&.&.8	N
7,795173	T&T	5,58	1	.&.	D
4,476258	T&.	3,14	0,99618	.&.	N
0,039059	T	-1,17	1,29E-06	GPCR&_rh	N
				Ankyrin_repeat-conta	
	.&.	1,26	0,000306	GPCR&_rhodopsin-lik	
1,618967	T&T&T	2,92	0,999923	Ribonuclea	N
1,344823	T&T&T&T	-1,08	1	.&.&.&.	N
5,002983	T&T&T&T8	5,01	1	.&.&.&.&.8	N
3,555122	D&D&D	5,56	0,999999	.&.&.	D
1,12127	.&T&T	1,83	0,004399	.&.&.	D
3,57243	.&T	6,04	0,988167	.&.	D
0,158793	T	-1,01	1		N
6,326589	.&T&T&T8	5,97	1	.&.&.&.&.8	N
5,558823	T	5,9	1		D
1,198241	T	2,83	0,506349		U
1,409568	.&.&.&.	3,49	0,999932	.&.&.&.	N
0,498252	.&T	-0,166	0,030419	.&.	
		-2,92	0,999888		
0,445674	T&T	-2,89	0,97231	.&.	U
0,040563	T	3,41	0,001212		
0,002573	T	-7,87	0,905829		
2,633305	T	3,87	0,088158		
1,043559	T	-0,627	0,000104		
0,013209	T	0,17	0,000145		
8,184124	T	4,31	0,992254		
	T	-0,27	1,12E-06		
1,537199		1,81	2,48E-05		
1,743996		0,702	8,18E-06		
0,184558		-0,514	1,51E-06		

0,666527	.&T&T&.	1,51			0,999684	.&.&.&.	N
3,372943	T&.	3,76			0,999941	.&.	
0,243184	D&D&T	-3,81			0,999989	.&.&.	N
7,308039	T	5,73			0,781394		D
3,024032	T	4,68			0,997747	Myosin_ta	N
1,724402	T&.&.&T	4,27			0,999998	.&.&.&.	D
3,291531	.&T&T&.&	5,05			0,993679	.&.&.&.&.&	D
1,575697	T	2,51			0,996722		N
0,001987	T				0,189361		
0,828347	T	-1,75			0,021615		N
4,57952	T	3,94			1		D
1,403582	T&T&.&T&	3,69			1	.&Sema_d	N
0,213449	T	-2,49			1	WAP-type_	N
0,387999	T&T	1,74			0,995904	.&.	U
3,602163	D	3			0,231559		D
0,231369	T&T	-1,5			0,999999	SPRY_dom	N
1,236484	T	3,3			0,091081		D
0,007104	D&D	-7,97			0,995958	.&.	N
2,283897	T&T&T&T	2,6			0,999348	.&.&.&.	N
0,131693	T	-4,25			0,999957		N
2,20186	D&D&D&..	5,95			1	.&.&.&.&.&	D
4,732678	T	5,29			0,998493		D
0,095047	.&.	-4,61			0,983294	.&.	N
5,55943	T&.&.	3,31			0,98439	.&.&.	D
3,421701	D&D&D&D	5,47			1	.&.&Myosi	D
8,262718	T	5			0,385394	Thioredoxi	D
2,927373	T&T	4,33			0,272334	Thioredoxi	N
3,865617	T	2,45	UQCRC2&	Heart_Atri	0,018995	Dynein_he	D
1,160085	T&T	3,76			0,881156	.&.	D
0,491103	T&T				1,1E-06	.&.	
0,487286	T	-0,702			0,002622		
1,707007	T	0,65			0,983348		N
0,555841	T&T&T	1,52			0,994709	.&.&.	N
2,690772	.&.&.&.&.	4,08			0,999081	.&.&.&.&.	N
0,764509	T	3,87			0,0001		U
0,178569	T	-4,4			0,999961		U
		2,12			0,143248	C-type_lectin-like	
2,669477	T&.&.	5,26			1	.&.&.	N
7,603781	.&T&T&T	4,75			1	.&.&.&.	D
3,289835	T&D	5,35			0,820621	.&.	N
0,897119	T&T&T	4,32			0,99946	.&.&.	N
0,132082	T&T&T&.	-0,915			0,997552	.&.&.&.	N
1,634335	T	4,4			0,999996		
0,128234		-1,06			0,999085		N
1,569773	T&T&T	4,44			1	CLU_doma	D
0,576636	T	-0,147			0,253982		
2,464289	T	4,87			0,99782		N
4,264272	T&T&T	5,32			1	Enolase&_	D
1,286714	T&T	1,47			0,045401	.&.	N
0,724348	T&T&D	3,12			1	.&.&.	N

0,837309	D	-0,412			0,085576		N
1,266688	T	3,2			1		
0,683066		2,56			0,454663		N
3,651578	T	4,31			0,999853		D
0,079138	T	-0,342			7,94E-05		N
0,419139	T	0,77			1,77E-06		
4,754117	D&D	4,43	NT5C3B&I	Esophagus	1	MyTH4_dc	D
0,83374	.&T&T&T8	4,84			0,011826	GPCR&_fa	N
2,374585	T&T&T&.	5,64			1	.&.&.&.	D
1,942244	D&D	4,51			0,046745	.&.	D
2,850753	T&T	5,24			0,982115	.&.	D
0,188323	.&T&T&T	-2,42			0,999989	G-protein_	N
3,862214	T&T	3,36			0,100369	.&.	N
	.&.&.	4,57			1	SH3_domain&SH3_dc	
2,777474	D	5			1		D
2,977195	T&T	4,2			1	Fascin_doı	D
7,371358	T	5,74			0,999987		D
2,268942	T&T&T&T	5,89			0,613633	.&.&.&.	D
1,67025	T&.	4,62			0,73894	.&.	N
4,23611	T	4,97			0,999997	PPM-type_phosphata	
2,200538	T&T&T	4,99			0,999972	.&.&.	N
1,702524	D&D&D&.	1,94			0,966385	.&.&.&.	N
		3,14			0,005358		
0,034548	T	-4,97			0,930751		
2,218963	T	3,46			0,999999	Chromatin_assembly_	
0,766409	T&.	-2,82			0,000227	.&.	U
1,373962	T&.	1,21			0,000121	.&.	N
1,373855	T&.	1,07			0,000106	.&.	N
0,048479	T&.	-4,72			0,001275	.&.	N
1,589645	T&T&T&T8	2,61	RPL36&LO	Adipose_S	1	.&.&.&.&.	D
3,523864	T&T&T&T	3,07			1	.&.&.&.	D
0,0207	T&.&T&T8	0,592			1,77E-06	.&.&.&.&.&.&.&.	
0,046077	T&T&T&T	-0,093			1	.&.&.&.	
1,889597	T&T&.&.&	-0,191			0,73941	Yip1_dom	N
2,124283	.&T&T&T8	4,82			0,005837	.&.&.&.&.	N
1,896238	T&T&T&T8	2,89			1	.&.&.&.&.	U
2,272618	T&T	1,82			0,048001	.&.	N
0,110117	T&T	-3,11			0,001999	.&.	N
5,361286	D&D&D	4,71			1	Thrombos	U
7,906044	T&T&T	4,76			1	.&.&.	D
1,470554	T&.	4,44			0,015261	.&.	N
0,295653	T&T	0,235			0,003013	Zinc_finger_C2H2-typ	
0,154369	T&.	-1,7			1,51E-06	Zinc_finger_C2H2-typ	
		-0,39			8,36E-06	Zinc_finger_C2H2-typ	
5,201376	T&T	4,78			0,27312	Immunogl	D
0,714702	T&T	2,21			0,999988	.&.	
0,610125	T&T&T	2,19			0,045044	.&.&.	N
1,313742	T&T	0,0201			0,002526	.&.	
1,450529	T&T&T	2,6			1	.&.&.	
0,594755	T	-1,32			7,89E-05		

2,584822	D&D	0,624			5,27E-06	Glycoprote	N
0,160066	T&T	0,728			1,16E-06	.&.	N
0,184848	T&T&T	0,257			0,002842	.&.&.	N
0,226928	T&T	-0,759			0,000269	.&.	
0,033545	T&T	-9,39			0,999969	.&SRCR_domain&SRC	
2,76128	T	5,27			1	Peroxidasi	N
0,899789	T	3,25			7,5E-05	RAD51_interacting_m	
1,490478	D&D	3,21			0,994789	.&.	N
0,700724	T	1,76			0,16037		N
3,970151	T&T&T&T&	3,46			0,999999	.&.&.&.&.&	N
		2,75	OXER1	Skin_Not_	0,999999		
1,982505	T	3,95			0,999649		N
1,797121	T&T	2,56			0,814963	.&.	N
3,156555	T&.	5,52			1	.&.	D
0,725815	T	-7,46			0,944377	Prenylcyst	N
0,746407		1,49			0,999679		N
2,947072	T&T	5,7			0,986934	GC-rich_se	N
1,528405	T&T	4,61			0,011177	.&.	
2,23875	.&T&T	5,77			0,953539	.&.&Pyridc	D
2,06941	T	1,33			1,01E-05		
1,396127	T&.	0,852			1,38E-05	.&.	
2,444083	D&D&D&D	4,38			0,999997	Ion_transp	D
0,953719	T	0,993			5,76E-06	CCDC144C-like&_coile	
0,061217	T	-1,99			2,57E-06	CCDC144C-like&_coile	
0,939813	T	0,993			1,22E-06		
0,094913	T	-6,36			0,999738		N
0,248139	T&T&T	-5,78			0,991108	.&.&.	N
1,029415	T&T&.&T&	4,92			0,806386	.&.&.&.&.&	N
2,968709	T	6,02			1	Glycosyltra	N
2,349024	T	5,48			0,99999	Tetratricop	D
2,428902	D&D	5,08			0,999737	TRAM/LAG	D
2,639941	T	4,9			1		N
1,840911	T&T&T&T&	2,43			0,064337	.&.&Immunoglobulin_	
0,782912	T	1,95			8,87E-05		N
2,446732	T&T&T&T	5,17			1	.&.&.&.	D
2,821518	T	5,09			0,999687		
0,208233	T	0,472			1,19E-05	Beta/gamr	N
5,467171	D&D&D&D	4,94			1	ATPase&_	D
2,497593	T	3,73			0,978243		N
0,028938		-7,58			0,988566		
3,4153	.&.&.&.	4,75			0,319251	.&.&.&.	N
4,525181	T	3,51			1		
3,677632	T&.&T	5,76			1	.&.&.	D
0,003204	T&T	-1,6			0,001058	Cystatin_d	N
2,023618	T	4,16			0,999531	Hemopexi	N
4,380308	D	4,18			0,999999		N
2,665662	T&T	4,56			0,999719	.&.	D
0,260805	T	-1,67			0,000112		
1,18004	T&T	3,11			0,999997	.&.	U
10,94457	D&D	5,41			1	.&.	D

0,006059	T&T	-8,49		1		.&.	N
0,279405	T	-4,57		0,999999			N
0,582151	T	2,56		0,000206			N
6,702658	T&T&T	6,02		1		.&.&.	D
1,840809	T&T&T&T	-2,75		0,998918		.&Zinc_fing	D
8,628573	D	5,01		1			D
1,263295	D	0,15		0,999943			N
0,061057	T&T	-7,26		1		Fibronectin	N
0,111114	D&.&.&D&	0,18		0,00782		.&.&.&.&.&	N
6,364786	T	5,29		0,999999			D
3,399812	T	4,94		0,999957		WD40-rep	D
0,784487	T&.	2,39		1		.&.	N
2,570003	T&T	3,87		0,999999	ENSG0000	.&.	D
0,028097	T&.&T&T	-1,32		1	ENSG0000	.&.&.&.	N
0,571315	T&T&T	1,05		0,999999		.&Immuno	N
0,292617	T&.&T	0,124		0,997287		.&.&.	N
		0,409		0,236338			
0,167254		-2,08		1,89E-05			N
1,043981	.&T&T&T	4,67		0,316881		Proline_de	N
3,168089	D&.	3,39		1		.&.	U
3,412214	T&.&.&T&T	4,52		1		.&.&.&.&.&	D
0,779706	.&.	2,02		0,999967		.&.	N
0,057334	T&.	-1,3		0,011665		.&.	
2,158229	T&T&T&T&	5,09		1		.&.&.&.&.	N
2,802978	.&T&T	5,62		0,971033		Rab-GTPas	D
0,135344	.&.	0,565		0,017129		.&.	N
0,076307	T	-5,23		0,987366			N
2,341997	D	3,48		0,999553			D
6,989716	.&T&.&T&T	4,74		1		VHS_doma	D
4,846233	T&.&.&T&T	4,89		0,999987		.&.&.&.&.&	D
0,627937	T	0,969	AL021878. Adipose_S	1			N
1,462506	T&T&T&T	3,05		0,99845		Rho_GTPa	U
0,658402	T&T&T	2,76		0,113529		SIR2-like_c	N
4,504909	T	4,61		1		Cadherin-li	U
4,453627	T&.&T	4,46		1		C2_domain	D
1,818289	T&T&T&T	3,89		1		Gelsolin-lik	D
0,065343	T&.	-3,43		0,060842		.&.	N
8,406484	D&D&D&D	4,86		1		.&.&.&.&.&	D
4,948293	.&T&T	4,45		1		.&.&.	D
1,094075	T&T&T	2,31		0,003132		Zinc_finger_C2H2-typ	
0,315951	T&T	-0,407		0,922137		VPS9_dom	N
2,126847	D&D&D&D	4,78		0,999996		.&SLC26A/SulP_trans	
0,520063	T&T	3,22		1		.&.	N
1,230362	T&.&T	1,82		0,998082		IPT_domai	N
5,142977	T&.&T&T&	5,55		1		.&.&.&Ras	D
0,071815	D	-3,94		0,999104		FAS1_dom	N
1,493659	D	4,44		0,984284		FAS1_dom	D
2,918996	T&T	5,08		1		.&.	D
						Dynein_heavy_chain&	
0,41653	T&T	-2,12		0,999995		Immunogl	N

		0,713			1,16E-06		
4,462189	T	6,06			0,999948	Myosin_head&_moto	
8,593468	T&T	5,9			1	Domain_o	D
0,150129	T&T&.&T	-7,43	CCDC58&A	Brain_Cere	0,999928	.&.&.&.	N
2,723528	T&T&.&T	4,3	AC083798.	Esophagus	0,625015	.&.&.&.	N
2,991725	.&.&.&.	3,81			0,561599	Domain_o	N
5,691432	T	5,65			0,070616	Fibronecti	D
6,545416	T&.&.&.	5,91			1	FAM69&_	D
2,442081	T&T&T	3,98			0,000116	Peptidase_	N
0,008201	T&T	-2,92			1	.&.	N
17,95384	D	5,8			1	Lipase/vite	D
0,015172	T&T&T&T	-9,39			1	.&.&.&.	N
0,132137	T&T	-4,54			0,000259	.&.	
0,550583	T&T				1,21E-05	.&.	
0,466969	T&T				1,19E-05	.&.	
0,138493	T&T				1,45E-05	.&.	
0,131598	T&T				1,19E-05	.&.	
0,455267	T&T	1,19			1,94E-05	.&.	
0,287987	T&T	1,19			1,35E-05	.&.	
0,210334	T&T				2,04E-05	.&.	
0,48359	T&T&.	2,9			0,008406	.&.&.	N
0,608076	T&T&T	2,37			0,997859	.&.&.	N
2,082353	T&T	1,51			0,005523	Glycosyl_h	N
6,227842	D&D	4,19			1	.&.	U
0,328213	T&T	1,68			0,995902	.&.	N
0,972493	T&.	5,02			0,986146	Rho_GTPa	N
3,557323	D&.&D&D	2,6			0,999995	Low-densi	N
1,576077	T&T&T&T	4,22			0,184242	.&.&GAIN_	N
4,714434	.&.&.	6,07			1	.&.&.	N
0,69384	.&.	4,02			0,932252	Vesicle_te	D
10,35117		5,7			1		D
0,051868	T&T	-6,69			0,923103	.&.	N
6,796156	.&.&.	5,47			1	.&.&.	D
6,053459	.&T&T&T	5,19			1	.&.&.&.	D
5,030303	T&T&T	5,52			1	.&.&.	D
9,338087	T&T&T&T	5,93			1	.&.&.&.	D
2,541599	T&T&T&T	5,93			0,999986	.&.&.&.	D
11,55267	T&T&T	5,37			1	Neurotran	D
2,534489		5,33			0,999985		N
2,268166	D	2,83			0,999876	Interferon_	N
1,214875	T	3,02			0,024692	Zinc_finger_C2H2-typ	
1,719747	T	4,81			0,999969		N
0,015875	D&.&D	-12			1	.&.&.	N
2,975259	D&D	5,99			1	.&.	D
1,660968	T&T&T	3,42			0,999722	.&.&.	N
4,484702	T&T&T	5,05			0,997542	.&.&.	D
4,414461	T&.&T&T	5,64			1	.&.&.&.	N
0,665343	T	2,32			0,00175		N
3,200723	T	3,16			1	GPCR&_rh	N
3,112847	.&D	5,39			1	.&.	U

6,644794	.&D	5,58			0,999999		FERM&_C-	D
11,79667	.&T	5,95			0,999937		FERM&_N-	D
9,6322	T&T&T&T	5,8			0,999992		.&.&.&.	D
2,442566	D&.&D&D&	4,17	SLC12A2	Cells_Cultu	0,964691		EGF-like_c	D
6,556754	.&.&.&T&.	5,69			0,622703		.&.&.&.&R	D
3,276287	T&T	5,01			1		.&.	D
8,57946	T&T	5,93			1		.&.	D
9,096755	T&T&T&T	5,8			1		.&.&.&.	D
4,023498	T	3,56			0,076215		Cadherin-like	
3,942772	T&.	2,51			0,987707		Cadherin-like&Cadhe	
6,569944	.&T	5,12			0,999468		.&Cadherin	U
1,619474	.&T	2,42			0,985322		.&Cadherin-like&Cadh	
4,057204	.&T	5,05			0,967081		.&Cadherin-like&Cadh	
0,372979	T&T&T&T	0,851			0,013234		.&.&Kazal_	N
1,397243	T	5,65			1		GPCR&_rh	D
3,613778	T&T	2,74			4,78E-06		Neurotran	D
2,075267	T	4,91			1		Rhodanese	D
2,193736	T&T&T&T	5,82			1		.&.&.&.	D
1,45463	T	3,4			0,997976			N
5,509636	D&D	5,84			1		.&.	D
0,382422	T&T&T&T&	0,564			4,12E-05		.&.&.&.&.&	N
0,918837	T	-0,376	MAK&SYCI	Adipose_S	1,59E-05			N
3,857441	.&T&T	4,6			0,994452		.&.&.	N
7,918152	T&.&T	5,5			1		.&.&.	D
0,007349	T	-6,24			0,997173			
	T	-3,92	AL662844.	Adipose_S	0,003185			
0,141185	T&T&.	1,6			0,999999		.&.&.	N
0,129172	T&T&.	-2,1			0,999999		.&.&.	
0,010515	T&T&.	-0,555			0,999999		.&.&.	N
0,475653	T&T&.	-1,63			0,999999	ENSG0000	.&.&.	
0,008114	T&T&.	-0,218			1	ENSG0000	.&.&.	N
0,014493	T&T&.	-3,63			1		.&.&.	
0,237856	T&T&.	2,67			0,999997		Immunogl	N
0,023012	T&T&.	-1,54			0,999996		Immunogl	N
1,414194	T&T&.	2,67			1		Immunogl	U
0,365254	T&T&.	2,67			0,999997		Immunogl	N
0,023626	T&T&.	-1,85			0,999997	ENSG0000	Immunogl	N
0,909372	T&T&.	0,7			0,999992	ENSG0000	Immunogl	U
0,268185	T&T&.	1,77			0,999995		.&.&.	N
0,000538	T&T&.	-5,62			1		MHC_class	N
0,00022	T&T&.	-5,62			1		MHC_class	U
1,39E-05	T&T&.	-5,62			1		MHC_class	N
1,386714	T&T	2,49			1	ENSG0000	MHC_class	N
0,284402	T&T	-4,05			1		MHC_class	U
0,270333	T	-2,03			1			N
3,227275	D&.	2,94			0,999966		.&.	D
3,028271	D&.&D&D&	4,89			1		.&GPCR_fa	D
7,625519	T&T&T	6,04			1		PWWP_do	D
2,977055	D	5,4			1			D
2,229089	T&T	4,89			0,999973		DNA_polyr	D

1,387411	T	3,6			0,999992	TGF-beta&	N
0,15229	T&T	-1,07			0,936818	.&.	N
2,091306	T&T&T&T&	4,9			1	.&.&.&.&.&	N
0,690169		2,46			0,207699		N
0,459868		-2,4			0,999995		U
6,20951	.&.&T	5,92			0,999985	Laminin_E	D
7,605465	T&T&T&T&	5,31			1	.&.&.&FER	D
0,327023	T	2,54			0,896001		N
0,372018	T	-2,45			0,008914		N
0,011511	T&.	-6,7			0,999884	.&.	N
3,984861	T	3,89			1		N
0,045827	T&T&T&T&	-8,26			1	.&.&.&.&.	N
0,250937	T&.	-1,25			0,814973	.&.	N
0,044178	.&T&.	-9,3			1	.&.&.	N
5,521427	.&T&.	5,36			0,994321	Dynein_he	D
0,143942	T&T	0,439	INMT	Artery_Tib	0,999949	.&.	N
6,522061	T&T&.&T	5,31			0,998861	AVL9/DEN	D
3,025651	D&D&D&D	4,9			1	Niemann-F	N
3,79694	T&T&T&T&	3,85			1	2-oxogluta	D
0,000363	T	-2,95			0,995822		N
2,407139	T&T&T	4,62			1	.&.&.	N
4,824248	T&.&T&T&	4,56			0,999778	.&.&.&.&.	D
1,243616	T&T&T&.	3,79			0,999952	.&.&.&.	N
0,45175	T&T&.&.&	-3,39			0,997563	.&.&.&.&.&	N
0,077751	T&.&.	-0,203			1,12E-06	Krueppel-associated_	
3,364042	T&T&T&T	4,63			0,999175	.&.&.&.	N
1,686745	T&T	3,73			0,357881	.&.	N
2,613909	T	5,71			0,999979		D
0,268933	T&T	2,75			0,000145	.&.	N
1,810592	D&D&D&D	0,447			0,256978	Cytochrom	N
4,792475	T&T	5,08			1	Neuronal_	D
0,516805	.&.	0,934			0,966333	.&.	
4,152308	D	4,44			1		D
6,663954	D&.	5,72			0,999997	STAS_dom	D
7,99002	T&.&.&.&T	5,06			1	Interferon-	D
1,108786	T&.&T&T	3,62			1	.&.&.&.	N
0,6272	T&T	0,955			0,792215	.&.	N
0,17041	.&D&D&.	2,07	ARHGEF35	Breast_Ma	0,000491	.&.&.&.	N
1,254055	T&T	1,98			1	.&.	N
7,601635	T&T&T	6,02			1	.&.&.	N
3,717347	.&.	5,43			1	.&.	D
0,32546	T	0,146			0,999999	Kinesin-like	N
1,219567	T&T&T&T	2,53			1	.&.&.&.	U
4,387869	T&T&T&T	5,63	FGFR1	Whole_Blc	0,999942	.&.&.&.	D
4,183908	T&.	5,19			1	.&.	D
2,20285	D&D&D&D	5,4			0,001488	.&Zinc_fing	D
3,535117	.&.&.&.&.	5,85			1	.&.&.&.&.	D
0,217006	T&.&T&T&	-0,722			1	Phospholip	N
0,941084	T&T	4,56			0,999999	Alcohol_de	N
5,363434	T&T	5,7			1	.&.	D

1,13609	T&T&T&T	2,5		0,999995	.&.&.&.	N
0,138573	T	-3,3		0,945542	Zinc_finger_C2H2-typ	
0,493708	T	0,53		0,999404		N
0,998241	T	1,83		0,991494		
2,683136	T	3,04		0,9881		D
6,803224	T&T	4,48		1	.&.	D
0,662704	T&T&T	1,38		1	.&.&.	N
		1,18		0,000147		
0,265521	T&T	-5,83	NUDT2&N Esophagus	1	.&.	N
1,914254	T	4,94		0,99999		N
0,716985	T&T&T	-0,852		0,077799	.&.&MHCK	N
0,993352	T&.&T	1,78		0,999776	.&.&.	N
0,002268	T&T	-2,31		0,999463	.&.	N
1,826011	T	3,48		1		
0,097433	T&T	-4,82		0,002103	.&.	
2,489714	.&.&.&.&.	6,06		1	.&.&.&.&.	D
	T					
	T	-0,755		5,52E-06	MAGE_hoı	U
	D	3,95		1	Dual_speci	D
	T	-1,4		6,75E-06	GAGE&GAGE	
	T	2,95		0,0031		N
	T	3,77		0,999977		D
	T	2,46		1		N
	T	-3,88		1		N
	T	-1,18		1		N
	T&T	0,158		0,982583	.&.	
	T&T&T	4,52		1	.&.&.	D
	T&T&T	-3,32		0,99943	.&.&.	
	T&T	4,21		0,999995	.&.	D
5,795811	T	4,63		0,999813		U
2,369423	T	5,15		0,999998		D
5,367897	T	4,09		0,083498		N
9,108773	T	5,56		1	Helicase&_C-terminal	
10,25163	.&T	5,23		1	Peptidase_	D
1,872367	T&T&.	3,21		0,161833	GNAT_don	D
2,407167	T	4,28		0,99963		N
6,0421	T&T	5,84		1	Protein_ki	D
4,264613	.&T&T	5,84		0,999849	DNA_topo	D
3,803721	T	2,45		1,88E-05		N
7,991892	T&T	5,1		1	AMP-depe	D
1,57419	T&T	0,945		1,34E-05	.&.	
7,920037	.&T&T	4,21		0,066949	Growth_fa	D
0,03753	T&T&T	0,677		0,000415	Alpha/beta	N
1,595453	T&T	2,49		1	.&.	N
0,700303	T&T&.	1,78		0,999995	.&.&.	U
1,903626	T&T&T&T8	2,13		1	.&.&.&.&.8	U
0,030011	T&T	-2,68		0,999775	.&.	N

4,182487		0,419	1,77E-06	Cyclophilir	U
	.&.&.			Neuroblastoma_breal	
1,234767		-4,97	1	B-box-type	N
				Transforming_growth	
4,074371	.&.	4,04	1	.&.	N
		-3,72	0,220261		
	.&.&.	2,77	1	.&.&.	
1,471662	.&.&.	-2,74	0,000678	Zinc_finger_C2H2-typ	
9,295277	.&.	4,56	0,977904	.&.	D
5,335459		3,44	0,976804	Beta/gamr	D
3,936446	.&.	0,619	0,999651	.&.	D
	.&.&.&.	1,3	0,999953	.&.&.&.	U

e types, 236200 (3), Autosomal recessive

I-CAP_pre	letaLR_pre	etaSVM_pr	onAssesso	tionTaster_	OVEAN_pr	en2_HDIV	en2_HVAR	IFT4G_pre

ofibromatosis, type 1, 162200 (3), Autosomal dominant| Neurofibromatosis-Noonan syndrome, 601

	T	T		N		D	B	T
D	T	T	M	D	D	B	B	T
D	D	T	.&L	N&N	N&N	.&D	.&P	T&D
T	T	T		N&N	D			D
T	T	T	M	N	D	D	P	T
	T	T	N&.	N	N&N	B&.	B&.	T&T
	T	T	M&M	D&D&D&D&D	D&D	D&P	D&B	D&D
D	T	T	.&N&.&.&.	N&N&N&N	N&N&.&.&	.&B&.&.&.	.&B&.&.&.	T&T&T&T&
T	T	T	L	N&N&N&N	N	B	B	T
D	D	D	.&L&.	D&D&D	N&N&N	.&B&B	.&B&B	D&D&D
D	T	T	N	D	N	D	P	T
T	T	T	.&M	D	D&D	.&B	.&B	D&T
T	T	T	.&.	D	N&.	.&.	.&.	T&T
D	T	T	N&.	N&N	N&N	B&B	B&B	T&T
D	D	D	M	P	D	B	B	D
T	T	T	.&.	D&D	N&.	.&.	.&.	T&.

T	T	T	N&.	N&N	N&N	B&.	B&.	T&T
T	T	T	L	N	N	B	B	T
T	T	T	M&M&M&	N&N&N	N&N&N&N	B&B&B&.	B&B&B&.	D&D&D&D
T	T	T	N	N&N	N	B	B	T
D	T	T	M&M	D&D&D	D&D	D&D	P&P	D&D
	T	T	L&L&L	N&N&N	N&N&N	P&P&P	B&P&P	T&T&T
T	T	T	L	N	N	B	B	T
D	D	D	M&.	D	N&.	D&D	P&D	T&T
R_domain			.&.		.&.	.&.	.&.	T&T
								T
T	T	T	.&M&.&.&	D&D&D	D&D&D&D	.&P&.&.&P	.&P&.&.&P	D&D&D&D
T	T	T	L&L&L&L	N&N&N&N	N&N&N&N	B&.&B&B	B&.&B&B	T&T&T&T
T	T	T	N	N	N	B	B	T
	T	T	L	N	N	D	D	D
D	D	D	.&L	D&D	D&D	D&D	P&D	D&D
T	T	T	M	D	N	P	B	T
T	T	T	.&L	N&N&N	N&N	P&B	B&B	T&T
T	T	T	L&L&.	N&N&N&N	N&N&N	D&D&.	D&D&.	D&D&D
T	T	T	N&.	N&N	N&N	B&.	B&.	T&T
	T	T	.&N&N	N&N&N&N	N&N&N	.&B&B	.&B&B	T&T&T
D	D	D	.&M	D	.&D	.&D	.&D	T&T
	T	T	M	N	D	P	P	D
	D	D	M	D&D&D	D	D	D	D
D	T	T		N&N&N	N			D
D	T	T	M&M&.&.	D&D&D&D	D&D&D&D	D&D&.&.&	D&D&.&.&	D&D&.&.&
	T	D	.&.&.&.&.	D&D&D	D&D&D&D	.&D&.&.&.	.&D&.&.&.	D&D&D&D
T	T	T	N&.	N	N&N	B&.	B&.	D&D
D	T	T	M	D	D	D	D	T
D	T	T	N	D	N	B	B	T
D	T	T	L&L&.&.&L	N&N&N&N	N&N&.&N	P&B&.&.&	B&B&.&.&	D&D&D&D
D	T	T	L&L&.&.&L	D&D&D&D	N&N&.&.&	B&B&.&.&	B&B&.&.&	D&D&D&D
	D	D	M&M	D	.&D	P&P	P&P	T&T
T	T	T	L&L	D	N&.	P&P	B&B	D&D
D	T	T	L&.&.&.	D&D&N	N&N&N&N	B&.&.&.	B&.&.&.	T&T&T&T
	T	T	.&.&.	D	D&.&.	.&.&.	.&.&.	D&D&D
D	D	D	.&H&.&.	D&D&D	.&D&D&.	.&D&D&.	.&D&D&.	D&D&D&D
D	T	T	M&M&M	D&D&D	D&D&D	D&.&D	P&.&D	D&T&T
T	T	T	L&.&L	D&D&D	N&N&N	B&B&B	B&B&B	T&T&T
	T	T	N&.&.&.	N&N	.&N&N&.	P&.&.&.	P&.&.&.	T&D&D&D
T	T	T	.&.	N&N&N&N	N&N	B&.	B&.	T&T
			.&.		.&.	.&.	.&.	T&T
T	T	T	.&.&.&.	N&N&N&N	N&N&N&N	B&B&B&B	B&B&B&B	T&T&T&T
T	T	T	N	N	N	B	B	T
T	T	T	.&.	N&N&N	N&N	P&.	B&.	D&D
D	D	T	L	N&N	N	B	B	T
T	T	T	L	D	D	P	B	T
	T	T	N&.&.	N&N&N&N	N&N&N	B&B&B	B&B&B	T&T&T
	T	T	H&H	N&N	D&D	D&.	D&.	D&D
			N&N&N		.&.&.	B&B&B	B&B&B	T&T&T
D	T	T	M	N	N	B	B	T

D	T	T	L	N	N	B	B	T
T	T	T	.&.&.&.	N&N&N&N	N&N&N&N	B&.&.&.	B&.&.&.	T&T&T&T
T	T	T	L&L&.&.&.	D&D	N&.&.&.&.	B&B&.&.&	B&B&.&.&	T&.&.&.&.
T	T	T	N	N&N	N	B	B	T
D	T	T		D	D			D
T	T	T	L	N	D	B	B	T
D	T	T	N&.	N&N&N	N&N	B&.	B&.	T&T
D	T	T		N&N	N			D
	T	T	N	N	N	B	B	T
D	T	T	N	N	N	B	B	T
D	D	D		N	N	B	B	T
	T	T		N&N	N	B	B	D
	T	T	L&.&.&L	N&N&N&N	N&N&N&N	B&B&B&B	B&B&B&B	T&T&T&T
	T	T	N&N	P&P&P&P&	N&N	B&B	B&B	T&T
	T	T	.&.	P&P	N&N	.&.	.&.	D&.
T	T	T	N	N&N	N	B	B	T
T	T	T	M&M	N	.&D	D&D	P&P	.&D
T	T	T	L&.&.	N&N&N	N&N&N	P&P&D	B&B&D	T&T&T
	T	T	M&M	D	.&D	B&B	B&B	.&D
T	T	T	L&L&L	N	.&.&.	B&B&B	B&B&B	.&.&.
D	T	T	N&N&N	D&D&D&N	N&N&N	P&.&.	P&.&.	T&T&T
D	D	D	.&.&.	D&D	N&N&N	.&.&.	.&.&.	D&T&.
T	T	T		N	D			D
T	T	T	L	N	D	B	B	T
T	T	T	N&N	N	.&N	B&B	B&B	.&T
D	T	T	N	N	N	B	B	D
D	T	T	L&.&.&L	N&N&N&N	N&D&N&N	P&.&P&P	B&.&B&B	D&T&D&D
	T	T	N	N	N	B	B	T
D	T	T	.&M&.&M	D&D&D&D	D&D&.&D&	D&B&D&B	P&B&P&B&	T&T&T&T&
D	D	D	.&M&.&.	D&D&D&D	N&N&N&N	.&D&D&.	.&P&D&.	D&D&D&T
D	D	D	M	D	D	D	P	D
D	T	T	.&.&.&.&.	N&N&N&N	N&N&N&N	.&.&.&.&.	.&.&.&.&.	D&D&D&D
D	T	T	N	D	N	P	B	T
	T	T	M	D	N	B	B	D
	T	T	L	D	N	B	B	T
D	T	T	N&N&.	D&D&D	N&N&.	B&B&.	B&B&.	T&T&.
T	T	T	.&.&.	N	.&.&D	D&D&D	D&D&D	.&.&D
D	D	D	M&.	D&D	N&N	D&.	D&.	D&D
D	T	T	.&.	N&N	D&N	.&P	.&B	D&T
T	T	T	.&N&.&N&	N&N&N&N	N&N&N&N	.&P&.&P&	.&B&.&B&	T&T&T&T&
T	T	T	.&L&L	N&N&D	D&.&D	.&B&B	.&B&B	D&D&D
T	T	T	N&.&.&.	D&N&N&N	N&N&N&N	B&P&.&P	B&P&.&B	T&T&.&T
	T	T	M	N	N	P	B	D
T	T	T	.&.	N&N&N	N&N	B&B	B&B	T&T
	T	T	.&.	N&N&N	N&.	.&.	.&.	T&T
D	T	T	.&L&L	N&N&N	N&N&N	D&D&D	D&D&D	T&T&T
T	T	T	.&.&L&.&.	D&D&D&D	N&N&N&N	.&P&D&.&	.&P&D&.&	T&D&T&T&
D	D	D	M	D	D	P	P	D
D	T	T	N&.	D	N&N	B&.	B&.	T&T

D	T	T		D	N			D
D	D	T	M&.	P&P	D&.	D&.	D&.	D&T
D	D	D	M	D	D	D	P	D
D	T	T	.&L&L	D&D&D&D	D&D&D	.&B&P	.&B&P	T&T&T
D	T	T	M&.&M&N	D&D&D&D	N&N&N&N	P&.&P&P&	P&.&P&P&	D&D&D&D
	D	D	.&.&L&.&.	D&D&D&D	N&.&N&N	.&.&D&.&.	.&.&D&.&.	T&T&T&T&
D	D	D	L	D	D	D	D	D
D	D	D	M	D	N	D	D	D
D	T	T	M&M&.	D&D	D&D&D	D&.&.	D&.&.	D&D&D
T	T	T	N&N&.	N&N	N&N&N	B&.&.	B&.&.	T&T&T
	T	T	M	N	D	D	D	T
T	T	T	L	N	N	B	B	T
	T	T	.&M	D	.&.	.&D	.&D	.&.
D	T	T	N&.	D&D	D&D	P&.	P&.	T&T
D	T	D	M	D&D&D	N	D	D	D
T	T	T	N&.	N	N&N	P&.	B&.	T&T
D	D	D	.&M	D&D	N&N	D&D	D&P	T&T
T	T	T	.&.&.&.&L	D&D	.&.&.&.&.	.&.&.&.&D	.&.&.&.&P	.&.&.&D&
	T	T	.&.	D&D	N&N	.&.	.&.	D&D
T	T	T	L&L	D&D&D	D&D	B&B	B&B	D&T
T	T	T	.&.&N&.&.	N	.&.&.&.&.&	.&.&B&.&.	.&.&B&.&.	.&.&.&.&.&
T	T	T	.&.	D&D&D	D&D	.&.	.&.	D&D
T	T	T	M&.	D	.&.	D&.	P&.	D&D
	T	T	N		N	B	B	T
ining_domain&Ankyrin_repeat-containing_domain&Ankyrin_repeat-containing_domain								T
e&_7TM&GPCR&_rhodopsin-like			M&.		.&.	.&.	.&.	.&.
	T	T	L&L&L	N&N&N	N&N&N	B&B&B	B&B&B	T&T&T
	D	T	M&M&M&	N&N&N&N	N&N&N&N	D&D&D&D	D&D&D&D	T&T&T&T
D	T	T	L&.&L&.&.	D&D&D&D	N&N&N&N	D&.&D&.&	P&.&P&.&	D&D&D&T
D	D	D	N&.&.	D&D&D	N&N&N	B&.&B	B&.&B	D&D&D
	T	T	L&.&.	D&D	.&N&N	B&.&B	B&.&B	T&T&T
T	T	T	M&M	D&D&D&D	.&N	P&P	P&P	T&T
T	T	T		N	N	B	B	T
T	T	T	.&N&N&.&	D&D&N&N	.&N&N&N	.&D&D&D&	.&P&D&D&	D&D&D&D
	T	T	M	D&D	N	D	D	D
T	T	T	L	N	N	B	B	T
	T	T	.&L&.&L	N&N&N	.&N&.&N	.&B&.&B	.&B&.&B	.&T&.&T
T	T	T	N&N	N	.&N	.&.	.&.	.&D
								T
T	T	T	.&L	N&N	N&N	.&B	.&B	T&T
	T	T	N	N&N	N	B	B	T
	T	T	N	N&N	N	B	B	T
	T	T	M	D&N	D	D	D	D
T	T	T	M	N&N	N	P	P	T
	T	T	N	N&N	N	B	B	T
T	T	T	M	D&D	D	D	D	D
T				N&N	D			T
	T	T		N				T
T	T	T		N				T
T	T	T		D	N			T

T	T	T	L&.&L&L		.&N&N&N	.&.&.&.	.&.&.&.	T&T&T&T
D	T	T	.&.	D&N	N&D	.&.	.&.	T&D
D	T	T	L&.&.	N&N	N&N&N	B&.&.	B&.&.	T&T&T
D	T	T	M	D	D	D	D	D
D	T	T	M	D	N	P	B	T
T	T	T	.&.&L&.	D&D	N&.&.&N	.&.&B&B	.&.&B&B	T&T&T&T
T	T	T	M&.&M&N	D&D&D&D	.&N&N&N&.&	B&P&B&P&	B&B&B&B&B	.&.&.&.&.&
T	T	T		N	D	P	B	D
	T	T	N	N	N	B	B	T
	T	T		N	N			T
D	T	T	L	D	D	D	D	D
	T	T	.&.&.&.&.	D&D&D	N&N&N&N	.&.&.&.&.	.&.&.&.&.	T&T&T&T&
T	T	T	N	N	N	B	B	T
T	T	T	.&L	N&N&D&D	N&N	.&P	.&B	T&T
D	D	D	M	D	D	D	D	D
T	T	T	N&N	N&N	N&N	B&B	B&B	T&T
T	T	T	N	N	N	B	B	T
	T	T	N&.	N&N	N&N	B&.	B&.	T&T
D	T	T	L&.&.&.	N&N&N&N	D&N&N&N	D&.&.&.	P&.&.&.	T&T&D&T
T	T	T	N	A&A&A&A	N	P	B	T
D	T	T	N&N&N&.	D&D&D&D	N&N&N&.	B&B&B&.&	B&B&B&.&	T&T&T&.&
	T	T	M	D	D	D	D	D
T	T	T	L&L	N&N&N	D&D	B&.	B&.	T&T
T	T	T	M&.&.	D	N&.&.	D&.&.	P&.&.	T&T&D
D	D	T	.&.&L&L	D&D&D&D	N&N&.&N	.&.&.&B	.&.&.&B	T&T&T&T
T	T	T	M	D	D	D	D	D
T	T	T	L&.	N	N&.	D&.	P&.	T&.
	T	T	M	D&D	D	D	D	
T	T	T	N&N	N&N	N&N	B&B	B&B	T&T
D	T	T	.&.	N&N&N	N&N	.&P	.&B	T&T
T	T	T	L	N	N	P	B	T
T	T	T	L	N	N	P	B	T
T	T	T	.&L&.	N&N&N&N	N&N&N	.&B&.	.&B&.	D&D&.
	T	T	.&.&L&L&.	D&D	N&N&N&N	.&.&D&D&	.&.&P&P&	T&T&T&T&
T	T	T	M	N&N	N			
T	T	T	L	N&N	N			
						D	P	D
T	T	T	L&.&.	D&D	N&N&N	B&.&.	B&.&.	T&T&T
D	D	D	L&.&.&.	D&D&D&D	.&N&N&N	P&.&.&.	P&.&.&.	D&D&D&D
D	D	D	M&.	D&N	N&N	P&.	P&.	T&T
T	T	T	M&M&.	N&N&N&N	N&N&N	B&B&.	B&B&.	T&T&T
T	T	T	N&N&.&.	N&N&N&N	N&N&N&N	B&P&.&.	B&B&.&.	T&T&T&T
D	T	T	M	N	N	P	B	D
T	T	T	N	N&N	N	B	B	T
D	T	T	L&L&.	D&D&D&D	N&.&.	B&B&.	B&B&.	T&T&.
T	T	T	M	N	D	B	B	T
T	T	T	L	D	N	B	B	T
D	T	T	H&.&H	D&D&D	D&D&D	B&B&B	B&B&B	T&T&T
T	T	T	.&L	D&N	.&N	.&B	.&B	T&T
	T	T	.&.&.	N&N&N	N&.&N	B&.&B	B&.&B	T&T&T

D	T	T	L	N	D	B	B	D
	T	T	L	N	N	B	B	T
T	T	T	N	N	N	B	B	T
T	T	T	M	D	N	B	B	D
T	T	T	N	N	N	B	B	T
	T	T	L	P	N			D
	D	D	L&.	D&D&D	.&D	D&.	D&.	D&D
T	T	T	.&N&.&N&	D&D&D&D	.&N&N&.&	.&B&B&B&	.&B&B&B&	T&T&T&T&
T	T	T	L&L&.&.	D&D&D&D	N&N&N&.	B&.&B&.	B&.&B&.	T&T&T&.
D	T	T	L&L	D&D	N&N	B&B	B&B	T&T
D	T	T	M&.	D&D&D&D	N&N	B&.	B&.	T&T
	T	T	.&.&.&N	N&N&N	.&.&N&N	.&.&B&B	.&.&B&B	.&T&T&T
T	T	T	L&.	N&N	N&.	P&.	P&.	D&D
omain&SH3_domain&MYO15B&_			.&.&.	D	.&.&.	.&.&.	.&.&.	D&D&.
D	D	D	L	D	N	P	B	T
	T	T	L&.	D&D	N&N	P&.	B&.	D&D
D	T	T	M	D	N	D	P	T
T	T	T	N&.&.&.	D&D&D	N&N&N&.	B&.&.&.	B&.&.&.	T&T&T&T
T	T	T	L&.	N	N&.	B&.	B&.	T&.
D	T	T	L	D&D	N	D	D	D
D	T	T	.&M&.	N&N	N&N&.	.&P&.	.&B&.	T&T&T
D	T	T	.&.&.&.	D&N&N	.&D&D&.	.&P&P&.	.&B&B&.	T&T&T&T
						B	B	T
T	T	T	L	N	N	P	B	T
T	T	T	M	N	N	P	B	T
	T	T	M&.	N	N&.	P&.	B&.	D&D
	T	T	M&.	P	N&.	D&.	P&.	T&T
D	T	T	M&.	N	N&.	P&.	P&.	D&D
	T	T	N&.	N	N&.	B&.	B&.	T&T
	T	T	N&.&.&.&.	D&D&D&D	N&.&.&N&	B&.&.&.&.	B&.&.&.&.	T&T&T&T&
D	T	T	M&M&M&	D&D&D	D&D&.&.	P&P&P&.	P&P&P&.	D&D&D&D
	T	T	.&.&.&.&N	N&N&N&N	N&.&N&N	.&B&B&.&	.&B&B&.&	T&T&T&T&
	T	T	.&.&N&.	N&N&N&N	N&N&N&N	.&.&B&.	.&.&B&.	T&T&T&T
T	T	T	N&N&.&.&	D&D&D	N&.&.&.&.	D&D&.&.&	P&P&.&.&	T&T&T&T&
D	T	T	M&M&M&	N&N&N&N	.&D&D&.&	D&D&D&D	B&B&B&B	.&T&T&T&
D	T	T	L&L&L&.&	D&N&N&N	N&N&N&.	.&D&.&.&.	.&D&.&.&.	D&D&D&D
	T	T	.&.	N&N	.&N	.&.	.&.	T&D
T	T	T	N&N	N	.&N	B&B	B&B	T&T
D	D	D	.&M&.	D&D&D	D&D&D	.&D&.	.&D&.	T&T&T
D	T	T	M&M&.	D&D&D	D&D&.	D&.&.	D&.&.	D&D&D
T	T	T	M&.	D&N&N	N&.	B&.	B&.	T&T
e&Zinc_fin	T	T	N&.	D&N&N	.&N	B&.	B&.	D&T
e&.	T	T	L&.	N	D&.	B&.	B&.	D&.
e&Zinc_finger_C2H2-type&Zinc_finger_C2H2-type&Zinc_finger_C2H2-type								
D	T	T	L&L	D&D	N&N	D&.	D&.	D&D
T	T	T	N&.	N&N	N&N	.&.	.&.	T&T
	T	T	.&.&.	D&D&D	N&N&N	.&.&B	.&.&B	D&D&D
	T	T	.&L	N&N	N&N	.&D	.&P	T&T
D	T	T	M&.&.	D&D&D	D&D&.	B&.&.	B&.&.	T&T&T
T	T	T		N	N			T

	D	D	.&.	N	.&D	.&.	.&.	T&T
	T	T	.&.	N&N	N&.	D&.	P&.	T&.
	T	T	N&N&.	N&N	N&N&.	B&.&.	B&.&.	T&T&.
T	T	T	N&N	N	N&.	B&B	B&B	D&D
T	T	T	N&N	N&N	.&N	.&B	.&B	T&T
	T	T	L	D	N	B	B	T
T	T	T	N	N	N	B	B	T
D	D	D	N&.	N&N	N&N	B&P	B&D	T&T
T	T	T		N	N			T
D	D	T	.&M&.&.&	D&D&D&D	D&D&D&D	D&D&.&.&	D&D&.&.&	D&D&D&D
				D				D
T	T	T	L	N	N	B	B	T
T	T	T	.&L	D&N	N&N	.&P	.&B	T&T
T	T	T	N&.	D	N&N	B&.	B&.	T&D
D	T	T	L	D&D&D&D	D	D	D	T
T	T	T		D	N			T
	T	T	L&.	D&D&D	D&D	D&.	P&.	D&D
	T	T	L&L	N&N&N	N&N	P&P	B&B	.&.
T	T	T	.&N&N	D&D&D&D	.&N&N	B&B&B	B&B&B	T&T&T
T	T	T	M	N	D	D	D	D
T	T	T	.&.	N	N&.	D&.	D&.	D&T
D	D	D	L&.&L&.	D&D&D&D	N&N&N&N	B&.&B&.	B&.&B&.	T&T&T&T
ed-coil_don	T	T	N	P	N			D
ed-coil_don	T	T	N	P	N			T
	T	T	M	P	N			T
	T	T	L	N&N	N	B	B	T
	T	T	L&L&.	N&N&N	N&N&N	B&.&.	B&.&.	T&T&T
	T	T	N&.&.&.&	N&N&N&N	N&N&.&N	.&.&.&.&B	.&.&.&.&B	T&T&T&T&
T	T	T	N	D	N	P	P	T
D	T	T	L	D&D	N	B	B	T
D	T	T	L&L	D&D	D&D	B&.	B&.	T&T
T	T	T	L	D	D	B	B	T
T	T	T	.&.&.&N&.	N&N&N&N	N&D&.&.&	.&.&.&B&.	.&.&.&B&.	.&.&.&.&.&
T	T	T	N	N&N	D			D
	T	T	L&L&L&.	D&D&D	N&N&N&N	B&B&B&.	B&B&B&.	T&T&T&T
	T	T	M	N	N	P	P	D
T	T	T	N	N	N	B	B	T
D	T	T	M&M&M&	D&D&D&D	D&D&D&D	D&D&D&D	D&D&D&D	D&D&D&D
T	T	T		N	N			T
	T	T						T
	T	T	.&N&.&.	D&D	.&N&N&.	.&D&D&D	.&P&P&P	.&T&T&T
D	T	T	L	D	N	D	D	D
	T	T	N&.&N	D&D&D&D	N&.&N	P&.&P	B&.&B	T&.&T
	T	T	N&N	N&N	N&N	B&B	B&B	T&T
T	T	T	N	D&D	N	B	B	T
D	D	D	M	D	N	D	P	T
T	T	T	M&M	D&D&D&D	D&D	B&B	B&B	D&D
	T	T	M	N	N	D	P	D
T	T	T	M&M	N&N	N&N	B&B	B&B	T&T
D	D	D	M&M	D&D&D	D&D	D&D	P&P	D&D

T	T	T	N&.	N&N&N	N&N	B&B	B&B	T&T
	T	T	N	N	N	B	B	D
	T	T	N	N	N	B	B	T
	T	T	M&M&M	D&D&D	D&D&D	P&P&P	P&P&P	D&D&D
T	T	T	M&M&M&	D&D&D&D	D&D&D&D	D&D&D&.	D&D&D&.	T&T&T&T
D	D	D	M	D&D	D	D	D	D
D	T	T	N	N	N	B	B	T
T	T	T	N&.	N&N&N	N&N	B&B	B&B	T&T
	T	T	N&N&N&N	P&P&P&P&	N&.&.&N&	B&B&B&B	B&B&B&B	T&T&T&T&
	T	T	M	D	D	D	D	D
D	T	T	L	D	N	P	B	T
	T	T	N&.	P	N&.	B&.	B&.	T&.
	T	T	L&.	P	D&D	P&.	B&.	T&T
	T	T	.&.&.&.	P&P&P	N&.&N&N	.&.&.&.	.&.&.&.	T&.&T&T
	T	T	M&M&.	P&P&P&P	N&N&N	.&P&.	.&B&.	T&T&T
	T	T	M&M&.	P&P	D&.&D	P&P&.	B&B&.	D&D&D
								T
T	T	T	L	N&N				T
	T	T	.&.&.&.	P&P&P	.&N&N&N	.&B&B&.	.&B&B&.	T&T&T&T
D	D	D	.&.	D&D&D	N&.	.&.	.&.	T&.
	T	T	.&.&.&.&M	D&D&D&D	N&.&.&N&	.&.&.&.&B	.&.&.&.&B	D&T&D&D
D	T	T	.&.	N&N	.&.	.&.	.&.	D&D
T	T	T	N&N	N	N&.	P&P	B&B	T&T
T	T	T	.&.&.&.&.	D&D&D	N&N&N&N	.&.&.&.&.	.&.&.&.&.	T&D&T&T&
	T	T	.&.&N	D&D	.&N&N	.&B&D	.&B&D	T&T&T
T	T	T	N&N	N&N	D&D	B&B	B&B	D&D
	T	T	L	N	N	B	B	T
	T	T	H	D	D	B	B	T
D	T	T	.&.&.&.&L&	D&D&D&D	.&D&.&D&	.&.&.&.&D	.&.&.&.&D	T&T&T&T&
D	T	T	.&.&.&.&.&	D&N&N&N	N&.&.&N&	D&.&.&D&	P&.&.&P&	T&T&T&T&
	T	T	L	N	D	B	B	D
T	T	T	.&.&L&.	D&D&D&N	N&N&N&N	B&P&B&.	B&B&B&.	T&T&T&T
T	T	T	N&N&.	N&N&N	N&N&N	B&B&.	B&B&.	T&T&T
D	T	T	L	D&D	D	D	D	T
T	T	T	M&.&M	D&D	N&.&N	P&.&.	P&.&.	T&T&T
D	T	T	M&.&.&M	D&D&D	D&D&D&D	B&.&.&B	B&.&.&B	D&T&T&D
T	T	T	L&.	N&N	N&.	B&.	B&.	T&.
D	D	D	.&.&.&.&.&	D&D&D&D	D&D&D&D	D&D&.&D&	D&D&.&D&	D&D&D&D
D	T	T	.&M&.	D&D	.&D&D	.&D&D	.&D&D	D&D&D
T	T	T	N&N&N	N&N&N	N&N&N	B&B&B	B&B&B	T&T&T
T	T	T	L&L	N&N&N	N&N	B&B	B&B	T&T
D	D	D	L&.&L&L&	D&N&N&N	N&N&N&N	.&.&B&B&	.&.&B&B&	T&T&T&T&
T	T	T	N&N	N&N	N&N	B&B	B&B	T&T
D	T	T	N&.&N	N&N	N&.&N	P&.&.	B&.&.	T&T&T
D	T	T	.&.&.&M&	D&D&D&D	N&.&N&N	.&.&.&D&I	.&.&.&D&I	T&T&T&T&
T	T	T	N	N	N	B	B	T
D	T	T	L	D	N	B	B	T
T	T	T	M&.	D&D	N&N	P&.	B&.	T&T
&_ATP-binding_dynein_motor_region_D5								
T	T	T	L&.	D&N&D	N&N	B&B	B&B	T&T

D	T	T	L	D	D	B	P	T
	T	T	.&.	D&D&D	D&D	D&.	D&.	D&.
T	T	T	N&.&.&.	N&N	N&N&.&N	B&.&.&B	B&.&.&B	T&T&T&.
	T	T	M&.&.&.	N&N	N&N&.&N	B&.&.&B	B&.&.&B	T&T&T&.
T	T	T	.&.&M&.	N&N&N	D&.&D&D	P&.&P&.	P&.&P&.	D&.&D&D
T	T	T	L	D	N	D	D	D
D	T	T	.&.&.&.	D&D&D	N&N&N&N	.&.&.&.	.&.&.&.	D&D&D&D
	T	T	L&L&.	N&N	N&N&N	P&P&.	P&P&.	T&T&T
T	T	T	N&N	N&N	N&N	B&B	B&B	T&T
D	D	D	H	D&D	D	D	D	D
T	T	T	.&.&.&.	N&N&N&N	N&N&N&N	D&D&B&B	P&P&B&B	T&T&T&T
	T	T	.&.	N&N&N&N	N&N	.&.	.&.	T&T
	T	T	.&.	D&D&N&N	N&N	.&.	.&.	T&T
T	T	T	.&.	N&N&N&N	N&N	.&.	.&.	T&T
T	T	T	.&.	N&N&N&N	N&N	.&.	.&.	T&T
	T	T	.&.	N&N&N&N	N&N	.&.	.&.	T&T
T	T	T	.&.	N&N&N&N	N&N	.&.	.&.	T&T
	T	T	.&.	D&D&N&N	N&N	.&.	.&.	T&T
T	T	T	.&.	N&N&N&N	N&N	.&.	.&.	D&D
T	T	T	L&.&.	D&D	N&N&.	B&.&.	B&.&.	T&T&T
D	T	T	L&L&L	N&N&N&N	N&N&N	B&B&B	B&B&B	D&D&D
T	T	T	M&.	N&N	N&N	D&D	D&D	T&T
	D	D	M&M	D&D	D&D	D&D	D&D	D&D
	T	T	.&N	N&N	N&N	B&B	B&B	T&T
T	T	T	N&.	D	N&.	B&.	B&.	T&T
	D	D	M&.&.&.&	D&D&D&D	D&.&D&D	D&.&.&.&.	P&.&.&.&.	D&D&D&D
	T	T	.&.&.&.&.&	N&N&N&N	N&N&N&N	B&.&.&.&.	B&.&.&.&.	T&T&T&T&
T	T	T	M&M&M	D&D&D	D&.&D	D&D&D	P&P&P	D&D&D
T	T	T	L&.	D&D&D	.&.	B&.	B&.	T&T
D	T	T		D				D
T	T	T	N&.	N&N	N&N	B&.	B&.	T&.
T	T	T	M&.&.	D&D	D&D&.	D&.&.	P&.&.	D&D&.
D	T	T	.&M&M&M	D&D&D&D	.&N&N&N	.&D&P&.	.&D&P&.	D&D&D&D
T	T	T	M&.&.	D&D&D&D	D&D&D	D&P&P	P&B&B	T&T&T
T	T	T	M&.&.&.	D&D	D&D&D&D	D&.&.&.	P&.&.&.	D&D&D&D
D	T	T	L&L&L&L	D&D&D&D	N&N&N&N	B&B&.&.	B&B&.&.	T&T&T&T
D	D	D	M&M&M	D&D&D&D	D&D&D	D&.&D	D&.&D	D&D&D
T	T	T	M	N		B	B	T
D	T	T	L	D	N	B	B	T
e&Zinc_fin	T	T	L	N	N	P	B	T
	T	T	M	N&N	D	P	B	D
T	T	T	N&N&N	N	N&.&N	B&.&.	B&.&.	T&T&T
D	D	T	L&L	D&D	N&N	B&B	B&B	T&T
T	T	T	.&.&.	N&N&N	D&D&D	.&.&.	.&.&.	T&T&T
D	T	T	L&.&.	D&D&D	D&D&D	P&.&D	B&.&P	D&T&T
T	T	T	.&.&M&.	D&D&D	D&.&D&D	.&.&D&.	.&.&P&.	D&D&D&D
	T	T	N	N	N	B	B	T
D	T	T	M	N	N	D	P	T
D	D	T	N&N	D	.&N	P&P	B&B	T&T

D	D	D	M&M	D	.&D	P&P	P&P	D&D
D	D	D	H&H	D	.&D	D&D	D&D	D&D
	T	T	M&M&.&.	D&D&D	D&D&D&D	D&D&.&.	D&D&.&.	D&D&D&D
	T	T	L&.&L&.&.	D&D&D&D	N&.&N&D	B&.&B&.&	B&.&B&.&	.&.&.&T&T
T	T	T	.&.&.&.&L	D&D	.&.&.&N&.	.&.&.&.&D	.&.&.&.&D	.&.&.&T&T
T	T	T	M&M	D&D	N&N	P&P	B&B	T&T
D	T	T	M&.	D&D	D&D	D&D	D&D	D&D
D	D	D	M&M&M&	D&D&D&D	D&D&D&D	.&.&D&D	.&.&D&D	D&D&D&D
T	T	T	M	N	D	D	D	T
D	D	D	H&.	D&D	D&.	D&.	D&.	D&D
D	T	T	M&M	D&D&N	.&N	D&P	P&P	D&T
T	T	T	L&L	N&N&N&N	.&D	P&P	P&P	T&T
ierin-like	T	T	M&M	D&D&D&D	.&D	D&D	D&D	T&T
T	T	T	L&L&.&L	N&N&N	N&N&N&N	B&B&.&B	B&B&.&B	T&T&T&T
	T	T	N	D	N	B	B	T
D	T	T	M&.	D&D	D&D	D&.	D&.	D&D
D	T	T	N	D	N	B	B	T
T	T	T	N&.&N&.	D&D&D&D	N&N&N&N	B&B&B&B	B&B&B&B	T&T&T&T
T	T	T		D&D&N	N			T
D	D	T	N&.	D	D&D	D&.	D&.	D&.
	T	T	.&L&.&L&I	N&N&N&N	N&N&N&N	.&.&.&B&E	.&.&.&B&E	T&T&T&T&
	T	T	M	N&N	D	B	B	D
T	T	T	.&.&L	D&D	.&N&N	.&.&P	.&.&B	T&T&T
	T	T	.&.&.	D&D&N	N&.&N	P&D&P	B&D&B	T&T&T
T	T	T	N	N&N	N	B	B	T
			N	N	N			T
	T	T	.&.&.	N&N&N&N	N&N&.	B&B&.	B&B&.	T&T&.
	T	T	.&.&.	P&P&P&P	N&N&.	B&B&.	B&B&.	T&T&.
	T	T	.&.&.	P&P&P&P	N&N&.	B&B&.	B&B&.	T&T&.
	T	T	.&.&.	P&P&P&P	N&N&.	B&B&.	B&B&.	T&T&.
	T	T	.&.&.	P&P&P&P	N&N&.	B&B&.	B&B&.	T&T&.
	T	T	.&.&.	P&P&P&P	N&N&.	B&B&.	B&B&.	T&T&.
	T	T	.&.&.	P&P&P&P	N&N&.	B&B&.	B&B&.	T&T&.
	T	T	.&.&.	P&P&P&P	N&N&.	B&B&.	B&B&.	T&T&.
	T	T	.&.&.	P&P&P&P	N&N&.	B&B&.	B&B&.	D&D&.
	T	T	.&.&.	P&P&P&P	N&N&.	B&B&.	B&B&.	T&T&.
	T	T	.&.&.	P&P&P&P	N&N&.	B&B&.	B&B&.	T&T&.
	T	T	.&.&.	P&P&P&P	D&D&.	B&B&.	B&B&.	D&D&.
	T	T	.&.&.	P&P&P&P	D&D&.	B&B&.	B&B&.	D&D&.
	T	T	.&.&.	P&P&P&P	N&N&.	B&B&.	B&B&.	T&T&.
	T	T	.&.&.	P&P&P&P	N&N&.	B&B&.	B&B&.	T&T&.
	T	T	.&.&.	P&P&P&P	N&N&.	B&B&.	B&B&.	T&T&.
	T	T	.&.	P&P&P&P	D&D	B&B	B&B	D&D
	T	T	.&.	N&N&N&N	N&N	B&B	P&B	T&T
	T	T		P	N	B	B	T
	D	D	M&M	D	D&.	D&D	D&D	T&T
D	D	T	.&.&N&.&.	D&D&D&D	N&.&N&N	.&B&D&.&	.&B&D&.&	T&T&T&T&
	T	T	L&.&.	D&D&N&N	D&D&D	P&.&.	P&.&.	T&T&T
	T	T	M	D	N	B	B	D
	T	T	L&L	D&D	D&D	B&.	B&.	D&T

	T	T	N	D&D	N	B	B	T
T	T	T	.&.		N&N	.&B	.&B	.&T
D	T	T	L&L&L&.&	D&D&D&D	N&N&N&N	B&B&B&B	B&B&B&B	T&T&T&T&
	T	T		N	N	B	B	T
T	T	T	N	N		B	B	T
T	T	T	.&.&M	D	.&.&N	.&.&P	.&.&P	.&.&.
D	D	D	L&L&L&L&	D&D&D&D	N&N&N&N	.&.&.&D&I	.&.&.&D&I	T&T&T&D&
T	T	T	N	N&N	N	B	B	T
T	T	T	L	N&N	N	P	B	T
T	T	T	N&.	N&N	N&.	B&.	B&.	T&T
D	T	T		D	D	D	P	T
T	T	T	N&.&.&.&.	N	N&N&N&N	B&.&.&.&.	B&.&.&.&.	T&.&.&.&.
T	T	T	N&N	N&N	N&.	B&B	B&B	T&.
T	T	T	.&N&.	N	.&N&.	.&B&.	.&B&.	.&T&.
	T	T	.&.&.	D	.&D&.	.&.&.	.&.&.	.&.&.
	T	T	N&.	N&N	N&N	B&.	B&.	T&T
D	T	T	M&.&.&.	D&D&D	N&N&.&N	D&.&.&.	D&.&.&.	D&D&.&D
D	D	D	M&.&.&M	D&D&D&D	D&N&D&D	.&.&.&D	.&.&.&D	D&D&D&D
	T	T	.&M&.&M	D&D&D&D	N&D&D&D	B&.&P&.&	B&.&P&.&	T&T&D&D
T	T	T	N	P	N	B	B	T
T	T	T	M&.&.	D&D&D	D&D&D	B&.&.	B&.&.	T&.&D
	T	T	.&.&.&.&L	D&D&D&D	N&.&D&N	.&.&.&.&P	.&.&.&.&B	T&D&D&T
	T	T	.&.&M&.	N&N	D&D&D&.	.&.&D&B	.&.&D&B	D&D&D&D
T	T	T	M&M&M&	N&N&N&N	D&D&D&D	B&B&.&B&	B&B&.&B&	T&T&T&T&
box&Kruep	T	T	N&N&.	N	N&.&.	B&B&.	B&B&.	T&T&T
D	T	T	.&.&.&L	D&D&D	N&N&N&N	D&.&.&P	P&.&.&B	T&T&T&T
T	T	T	L&L	D&D	N&N	B&B	B&B	T&T
	T	T	L	N&N	D	B	B	D
	T	T	N&.	N&N&N	N&N	B&B	B&B	T&T
D	D	D	H&.&.&.	D&D&D&D	D&D&D&D	D&.&.&.	D&.&.&.	D&D&D&D
D	T	T	L&.	D&D&D	N&N	D&D	D&D	T&T
	T	T	N&N		N&.	.&.	.&.	T&T
D	T	T	M	D	D	D	D	D
	D	D	M&M	D&D&D&D	D&.	D&D	D&D	T&.
T	T	T	L&.&.&.&L	D&D&D&D	N&.&D&D	D&.&.&.&I	P&.&.&.&P	D&D&T&D
D	T	T	.&.&N&N	D&D&D&D	N&.&N&N	.&.&P&B	.&.&B&B	T&T&T&T
T	T	T	.&.	D&N&N	N&N	.&B	.&B	T&T
	T	T	.&.&N&.	N&N&N	.&N&N&.	.&.&B&.	.&.&B&.	.&T&T&.
T	T	T	.&L	N&N&N	N&N	.&P	.&B	T&T
D	T	T	M&M&M	D&D&D	D&D&D	D&D&D	D&D&D	D&D&D
D	T	T	M&M	D&D	D&D	P&P	B&B	T&T
	T	T	L	N	D			T
D	T	T	N&N&N&N	N&N&N&N	N&N&N&N	B&.&.&.	B&.&.&.	T&D&D&T
	T	T	M&M&.&.	D&D&D&D	D&D&D&D	D&D&D&D	D&D&D&D	D&D&D&D
	T	T	N&.	D&D&D	N&N	D&.	D&.	D&D
T	T	T	L&L&L&.	D&D&D&D	N&N&N&N	B&B&B&.	B&B&B&.	T&T&T&T
T	T	T	L&L&L&.&	D&D&D	N&N&N&N	B&B&B&.&	B&B&B&.&	T&T&T&T&
D	T	T	N&.&N&.&	D&D&D	N&.&N&N	B&B&B&.&	B&B&B&.&	T&T&T&.&
T	T	T	N&.	N&N	N&N	B&.	B&.	T&T
T	T	T	M&.	D&D	D&D	B&P	B&B	T&D

T	T	T	N&.&N&.	D&D&N&N	N&N&N&N	B&.&B&.	B&.&B&.	T&T&T&T
T	T	T	N	N	N	B	B	T
	T	T	N	N	N	B	B	T
D	T	T		N	N			D
D	T	T			D			D
D	T	D	.&H	D&D	D&D	.&D	.&D	D&D
D	T	T	.&L&.	N&N	N&N&N	.&B&.	.&B&.	T&T&.
			N			B	B	T
	T	T	N&.	N&N&N&N	N&N	B&.	B&.	T&T
T	T	T	L	D&N&N	N	B	B	T
T	T	T	.&.&N	N&N&N&N	N&N&N	B&B&B	B&B&B	T&T&T
T	T	T	L&.&.	N&N	N&.&N	B&.&.	B&.&.	T&T&T
T	T	T	M&.	N&N&N&N	N&N	B&B	B&B	T&T
D	T	T	N	D	N	B	B	T
T	T	T	N&.	N&N	N&N	B&.	B&.	T&T
	T	T	M&M&.&N	D&D&D&D	N&N&N&N	B&.&.&.&.	B&.&.&.&.	T&T&T&T&
			M	N	D			T
	T	T	L	N	D	D	D	T
D	D	D	M	D	D	D	D	D
D	T	T		N	N			T
D	T	T	M	N	N	P	B	T
	T	T	M	D	N	D	D	T
	T	T	N	N	N	B	B	T
	T	T	N	N	N	B	B	T
	T	T	N	N	N	B	B	T
D	T	T	L&L	N&N	N&N	B&B	B&B	T&T
D	T	T	M&M&.	N&N	N&N&N	D&D&.	D&D&.	T&T&.
T	T	T	.&.&.	N&N&N&N	N&N&N	B&B&B	B&B&B	T&T&T
D	T	T	M&.	D&D&D&D	D&D	D&D	D&D	D&D
T	T	T	M	N	D	D	D	D
T	T	T	L	D	N	B	B	T
	T	T	M	D&D&D	D	P	P	D
D	D	D	M	D&D	D	P	B	D
	T	T	.&H	D&D	.&D	.&D	.&D	D&D
D	T	T	L&.&.	D&D&D	D&D&.	B&.&.	B&.&.	T&T&T
T	T	T	L	D	N	P	B	T
D	T	T	.&L	D&D	D&N	D&.	P&.	D&D
T	T	T	M&M&.	D&D&D&D	.&D&.	D&D&.	D&D&.	D&D&.
	T	T		D	N			D
D	T	T	H&.	D	D&.	D&.	D&.	D&D
T	T	T	.&N	N&N	N&N	.&D	.&P	D&D
D	T	T	.&M&M	D&D&D	.&D&D	.&P&P	.&B&B	.&T&T
T	T	T	N&.&.	N&N&N&N	N&N&N	B&.&B	B&.&B	T&T&T
T	T	T	.&L	D&D&D	N&N	B&B	B&B	T&T
	T	T	.&.&.	P&P&P&P	N&N&.	B&B&.	B&B&.	D&D&.
D	T	T	M&.&.&.&	N&N&N&N	N&N&N&N	D&.&.&.&	B&.&.&.&	T&T&T&T&
	T	T	N&N	N&N	N&N	B&.	B&.	T&T

		N				
point_family_(NBPF)_domain&N	.&.&.		.&.&.	.&.&.	.&.&.	.&.&.
		A				
_factor-beta&_C-terminal&Transforming_growth_factor_beta&_conserved_site&Transforming_grc						
	.&.	A&A&A&A	.&.	.&.	.&.	.&.
		N&N&N				
	.&.&.		.&.&.	.&.&.	.&.&.	.&.&.
e&Zinc_finger_C2H2-type&Zinc_f	.&.&.	D&D	.&.&.	.&.&.	.&.&.	.&.&.
	.&.	A	.&.	.&.	.&.	.&.
		A				
	.&.	A&A	.&.	.&.	.&.	.&.
	.&.&.&.	A&A&A	.&.&.&.	.&.&.&.	.&.&.&.	.&.&.&.

SIFT_pred	-MKL_codi	n-XF_codi	nomADe_A	mADe_AFR	nADe_AMI	mADe_ASJ	mADe_EAS	mADe_FIN
			0,000284	9,72E-05	0	0	0	5,61E-05
			0,000245	9,49E-05	0	0	0	0
321 (3), Autosomal dominant								
			4,01E-06	0	0	0	0	0
			0,000496	0	0,000376	0	0	0,000179
			0,000228	0,000171	0,000467	0	0,000304	0
			0,000356	0	0,000933	0,000299	0,00066	0
			0,002677	0,007794	0,003942	0,001088	0,003056	0,000145
			0,000458	0,000129	0,000487	0,000236	0,000278	0
			0,004594	0,000309	0,002065	0,01736	0	0,000139
			0,00015	0,000248	8,82E-05	0	5,51E-05	0
			0,01434	0,001709	0,02906	0,002103	0,00196	0,001907
			0,001571	0,000803	0,001452	0,000699	0	9,29E-05
			0,003073	0,000778	0,00223	0,005195	0	0
			0,002635	0,002093	0,00197	0,000496	0,000766	0,001575
			0,002151	0,007074	0,005088	0,008631	0	0
			0,008683	0,002046	0,006107	0,00625	0	0,005685
			0,000909	0,01276	0,002809	0	0	0
			0,002207	0,000362	0,001932	0,001146	0	0,001265
			1,19E-05	0	0	0	0	0
			0,004762	0,001068	0,002086	0,0163	0,000621	0,000902
			0,000232	0	0,000116	0	0,000109	4,62E-05
	N		0,007165	0,000969	0,004261	0,007648	5,56E-05	0,01735
D	D	D	7,56E-05	0	0,000174	0	0	0
D&D	N	N	5,17E-05	0,000123	0	9,92E-05	5,44E-05	0
D	N	N						
T	D	N	8,35E-05	0	0,000231	0	0	0
D&D	D	N	0,01268	0,000631	0,001863	0,004123	0,04174	0,01045
D&D	D	D	0,002806	0	0,000532	0,001977	0	0
T&T&.&.&	N	N	0,000235	0	0	0	0	0
T	N	N	0,004362	0,000766	0,004421	0,000764	0	0,000263
D&D&D	D	D						
D	D	D	0,00162	0,001539	0,002429	0,01063	0,000163	0
D&D	D	D	3,98E-06	0	0	0	5,44E-05	0
T&.	D	D	0,000255	0	4,39E-05	0,003483	0	0
T&T	D	N	0,001587	0,000434	0,000405	0,000697	0	0,000463
D	N	D	0,001954	0,000123	0,002429	0,01022	0,000109	0
T&.	D	D	0,000342	6,15E-05	0,00026	0	5,44E-05	0

T&T	N	N	8,01E-06	0	2,93E-05	0	0	0
T	N	N	0,005809	0,001907	0,006162	0,004467	5,44E-05	0,001996
D&D&D&D&D	N	N	0,006024	0,001051	0,00342	0,00439	0	0,007081
T	N	N	0	0	0	0	0	0
T&T	D	D	7,31E-05	0,000287	4,07E-05	0,000357	0	0
D&T&T	N	N	0,007545	0,01692	0,003587	0,005757	0,000598	0,000924
T	N	N	3,59E-05	0	2,9E-05	0	0	0
T&.	D	D	7,96E-06	0	0	0	0	0
.&.	N		0,2114	0,5058	0,3163	0,08216	0,655	0,04629
		N						
D&D&D&D&D	D	N	0,000143	0	0,000174	0	0	0
T&T&T&T	N	N						
T	N	N	0,03648	0,009979	0,07409	0,02057	0,165	0,003599
D	N	N	0,005314	0	5,78E-05	0,003869	0,00984	0,04093
D&D	D	D						
T	D	D	0,004307	0,000923	0,001475	0,000298	5,44E-05	0,002495
D&D	D	N	0,000637	0	0	0,008756	0	0,000185
D&D&D	N	N	0,001816	0,000254	0,000145	0,006	0,000272	0,001434
T&T	N	N						
T&T&T	N	N	0,00414	0,001053	0,006168	0,006992	0	0,000693
.&T	D	D	7,96E-06	0	0	0	0	0
D	D	D	0,007277	0,001046	0,008291	0,009145	0	0,001063
D	D	D	0,005215	0,000743	0,001371	0,008993	0	0,000839
T	N	N	0,000438	0,000337	0,000136	0	0,001735	0
D&D&D&D&D	D	D	7,96E-06	0	0	0	0	0
D&D&D&D&D	D	D	0,004174	0,000433	0,002665	0,02345	0	0,00088
D&D	N	D						
D	D	D	7,96E-06	0	0	0	0	0
T	D	D						
D&D&.&T&	D	N	0,000805	6,47E-05	0,000522	0	0,000167	0
D&D&.&.&	D	N	0,000129	6,48E-05	0	0	0	0
.&D	D	D	0,007017	0,006646	0,001447	0,001986	0,000272	0,01162
D&.	D	D	1,19E-05	0	2,89E-05	0	0	0
T&T&T&T	D	N	3,98E-06	0	0	0	0	0
D&.&.	D	N	0,002605	0,000204	0,003639	0,0095	5,49E-05	0,000221
.&D&D&.	D	D						
D&D&D	D	D						
T&T&T	D	D	1,59E-05	0	0	0	0	0
.&T&T&.	N	N	0,01502	0,005362	0,004147	0,000993	0,1022	0,005009
T&T	N	N	4,03E-06	0	0	0	0	0
.&.		N	0,001336	0,0008	0,000347	0	0	0,000323
T&T&T&T	N	N	0,002818	0,001309	0,000572	0,000943	0	0,01221
T	N	N	0,000655	0	0,001755	0,002421	0	0
T&T	N	N	4,32E-05	0	0	0	0	0
T	D	N	0,000374	0,000185	0	0	0	0
T	D	D						
D&D&D	N	N	0,000826	0,00717	0,001477	0	0	0
D&D	N	D	0,009112	0,0032	0,005301	0,007048	0,0192	0,001109
.&.&.	N		0,004793	0,000908	0,005173	0,007712	5,58E-05	0,001706
T	D	N	0,003761	0,000493	0,00217	0	0	0,003374

T	N	N	4,02E-06	0	0	0	5,56E-05	0
T&T&T&T	N	N						
T&.&.&.&.	D	D	0,00545	0,0016	0,0035	0	0	0,006561
T	N	N	2,81E-05	0,000185	2,97E-05	0	0	0
D	D	D	0,000615	0,004404	0,000608	0	0	0
T	D	N	0,003068	0,000431	0,00217	0,00139	0	0,005502
T&T	N	N	9,57E-05	0	0,000203	0	0	0
D	N	N	0,001105	0,000295	0,001727	0,002287	0	6,53E-05
D	N	N	0,006297	0,00085	0,00587	0,0133	0	0,000144
T	N	N						
T	N	N	0,004236	0,001371	0,002916	0,004272	9,04E-05	0,00382
D	N	N	0,009145	0,002809	0,004403	0,03116	0	0,004999
T&T&T&D	N	N	0,003653	0,000413	0,002138	0,02693	0	0,001654
T&T	N	N	0,1769	0,04065	0,2385	0,2012	0,1498	0,166
D&D	N	N	0,7971	0,7909	0,8854	0,7529	0,7712	0,8351
T	N	N	0,000104	6,46E-05	0,000319	0	5,56E-05	0
.&D	D	D	9,15E-05	6,15E-05	0	0	0	0
T&T&D	D	N						
.&D	D	N	0,01002	0,002602	0,00801	0,01092	5,44E-05	0,004021
.&.&.	N	N	0,002934	0,000494	0,000608	0,00288	5,45E-05	0,002634
T&T&T	N	N						
D&D&D	D	D						
D	N	N	0,004035	0,000969	0,002346	0,000298	0	0,001994
T	N	N	3,59E-05	0	0	0	0	0
.&T	N	N	0,000187	6,15E-05	0,000232	0	0,00049	0
T	N	N						
D&D&D&D	N	N	0,000745	0,000362	0,000381	0,00021	0	0,000418
D	N	N	0,006873	0,001354	0,006651	0,00873	0	0,001711
T&D&.&D&	N	N	1,99E-05	0	2,89E-05	9,92E-05	0,000109	0
T&D&D&T	D	D	1,99E-05	6,15E-05	0	0	0,000218	0
D	D	N	3,28E-05	0	2,94E-05	0	0	0
T&T&T&T&	N	N	3,98E-06	0	0	9,93E-05	0	0
T	D	N	3,21E-05	0	5,8E-05	0	0	0
T	D	N	0,007289	0,001378	0,004334	0,007765	0	0,009548
T	D	N	0,00908	0,001939	0,0103	0,007654	0,000167	0,005717
T&T&.	D	D	0,001201	6,15E-05	0,001908	0,000694	0	4,62E-05
.&.&D	N	N	7,88E-05	0	4,07E-05	0	0	0
D&D	D	N	7,2E-05	0,000101	3,69E-05	0,000453	0	0
D&T	N	N						
T&T&T&T&	D	N	0,00039	0,004737	0,000145	0	0	0
T&.&T	N	N	0,000915	0,000308	0,000318	0,000397	0	0,000277
T&T&T&T	N	N	3,21E-05	0	2,91E-05	0	0	0
D	N	N	0,009495	0,001975	0,00999	0,03324	5,44E-05	0,001019
T&T	N	N	7,46E-05	0,000523	0,000118	0	0	0
T&.	N	N	0,000193	0,000155	3,07E-05	0	5,66E-05	9,78E-05
T&T&T	D	N	0,000432	0	0,000243	0,000103	0	0,000791
D&T&T&D	D	N	0,001242	0,000185	0,000607	0,000199	0,000544	0,000554
D	D	D						
T&T	D	N						

D	D	D	0,000212	0,00014	2,95E-05	0,000617	0	0,000668
D&.	N	N	0,02426	0,0118	0,01037	0,0449	0,005143	0,04425
D	D	N	0,004903	0,000738	0,003382	0,005258	0	0,003788
T&T&T	D	D	0,002274	0,000123	0,000809	9,92E-05	0,000163	0,008594
D&D&D&D&[	D	D	4,38E-05	0	0	0	0	0
T&.&T&T&	D	D	0,006786	0,001372	0,0031	0,009981	5,92E-05	0,001459
D	D	D	2,24E-05	0	0	0	0	0
D	D	D	4,4E-06	0	0	0	0	0
D&D&D	D	N						
T&T&T	N	N	0,000426	6,15E-05	0,00084	0,002382	0	0
T	D	N	0,009365	0,001426	0,002496	0,008458	5,57E-05	0,02528
T	D	N	0,00023	6,59E-05	0,000333	0,001299	0	4,75E-05
.&.	D	D	0,01099	0,001681	0,003328	0,005901	0,000279	0,001579
T&T	D	D	4,01E-06	0	0	0	0	0
D	D	D	0,000836	0,000378	0,000283	0,000118	0	0,000237
T&T	N	N	0,00011	0	0,000145	0	0	0
D&D	D	D	1,21E-05	0	0	0	0	0
.&.&.&.&.	D	D						
D&D	D	D	0,00955	0,001233	0,03784	0,001591	0,000163	0,000786
D&T	D	N	0,000764	0,000257	7,04E-05	0	0	0,000327
.&.&.&.&.&	N	N	0,000694	7,05E-05	5,86E-05	0,0134	0	0
D&D	D	D	0,000664	0,000123	5,78E-05	0,01329	0	0
.&.	D	N	8,19E-06	0	0	0	0	0
T	N	N	0,000587	0,001872	0,000708	0,000338	0,00147	0,000197
.&.	D		0,00676	0,00273	0,008705	0,004142	0	0,001822
T&T&T	N	D	0,001357	0,01668	0,001474	9,93E-05	0	0
D&D&D&D&[	N		0,000585	0,007871	0,000463	0	0	5,28E-05
D&D&D&D&[	D	D	0,000541	0,002092	0,001185	9,92E-05	0	0,000508
D&T&T	D	D	7,97E-06	0	0	0	0	0
.&T&T	D	N	0,00431	0,001422	0,002267	0	0	0,005667
.&D	D		0,000155	0	0	0	0	0
T	N	N						
.&D&D&D&	D	D	2,4E-05	0	0,000148	0	0	0
D	D	N	0,007451	0,001232	0,004135	0,000398	0	0,01497
T	D	N	0,000326	0	0,000465	9,94E-05	0	0,000139
.&T&.&T	D	N	0,002664	0	0,000293	0,002845	0	0,01614
.&D	N		0,004874	0,001597	0,003489	0,002607	0	0,007208
	N							
T&T	N	N	0,000321	0,000194	5,79E-05	0	0	0
T	N		0,0186	0,004395	0,03489	0,03462	0,002079	0,01223
T	N		0,01157	0,003272	0,0157	0,02471	0,001128	0,004027
D	N		0,01601	0,002673	0,007607	0,0384	0,001333	0,002044
T	N		0,009321	0,001684	0,01911	0,01503	0	0,001796
T	N		0,02325	0,01405	0,03532	0,03689	0,002505	0,004679
D	D		9,15E-05	0,000134	0,000375	0	0,000112	0
T	N	N						
	N	N	0,001174	0,007454	0,001376	0	0	0
	D	N	0,001	0,005108	0,00119	0	0	0
T	N	N	0	0	0	0	0	0

.&T&T&T	N	N	0,005695	0,000769	0,005107	0,003851	9,52E-05	0,000714
D&D	D	N	0,00026	0,000155	0,000368	0	0	0,000371
T&T&T	N	N	0,002208	0,000739	0,003848	0,001587	0	9,24E-05
D	D	D	0,002617	0,000753	0,003456	0,007906	0	0,000165
T	D	N	3,18E-05	0	8,68E-05	0	0	0
T&.&.&T	D	D	0,001908	0,000234	0,000238	0	0	0,004943
.&T&T&.&'	D	N	0,003304	0,000933	0,000283	0,001644	0	0,005538
T	N	N	1,19E-05	0	0	0	0	0
T	N	N	0,005879	0,001511	0,002491	0,008655	0,00089	0,023
T	D	N	0,00645	0,000677	0,003643	0,01102	0	0,002495
D	D	N						
T&T&D&T&	D	N	0,003462	0,00057	0,00104	0,000586	0	0,002041
T	N	N						
T&T	N	N	1,24E-05	0	0	0	0	0
D	D	N	4E-05	0	0	9,98E-05	0	0
T&T	D	N	5,4E-06	0	0	0	0	0
D	D	D	0,002874	0,000862	0,002286	0,000596	0	0,001017
T&T	N	N	0,00251	0,00032	0,002839	0,0138	0	0,00177
T&D&D&D	D	D	0,000186	0,000124	2,9E-05	0,000501	0	0
T	N	N	0,000159	0	0,000202	9,92E-05	0	0
T&T&T&.&	D	D	1,47E-05	0,000168	3,45E-05	0	0	0
D	D	N	0,005613	0,001108	0,002401	0,002786	0	0,004484
D&D	N	N	3,61E-05	0	0	0	0	0
T&.&.	D	D	0,001815	0,000373	0,002251	0,002198	0	0,000602
T&T&.&T	D	D	0,000104	0	8,71E-05	0	0	0
D	D	D	0,003538	0,000677	0,00214	0,001987	0	0,000601
T&.	D	N	0,001229	6,15E-05	0,001474	0,001389	0	4,62E-05
D	D	N	0,006241	0,000923	0,003614	0,01271	0	0,01626
T&T	D	N	1,22E-05	0	0	0	0	0
T&T	N	N	0,07351	0,00497	0,1334	0,05785	0,02842	0,1058
T	N	N	0,000432	6,43E-05	0,000205	0	0	0,000371
D	N	N	2,03E-05	0	0	0	0	0
T&D&D	N	N	0,000104	6,2E-05	0	0	0,000109	0,000649
T&T&T&T&	D	D	0,005555	0,001661	0,003411	0,007837	0	0,005913
T	N	N						
T	N	N	0,00012	0,000853	0	0	0	0
	N		1,28E-05	0,000125	0	0	0	0
T&T&T	D	D	8,78E-05	0,000124	2,9E-05	0	0	0
.&D&D&D	D	D						
T&T	D	N	6,76E-05	0	0	0	0,000544	9,24E-05
T&T&T	D	N	4,01E-06	0	0	0	0	0
T&T&T&T	N	N	8,42E-06	0	0	0	0	0
D	N	N	2,18E-05	0	4,08E-05	0	0	0
T	N	N	3,33E-05	0	0	0	0	0
T&.&.	D	D	8,08E-06	0	0	0	0	0
T	N	N	0,001961	0,000369	0,00217	0,000298	5,44E-05	0,000185
T	D	N	0,00006	0	2,89E-05	0	0	0
D&D&D	D	D	0,001272	0	0	0	0	0
.&T	D	N	3,98E-06	0	0	0	0	0
T&.&T	N	N	0,001658	0,01993	0,001821	0	0	0

T	N	N	8,03E-06	0	0	0	0	0
D	N	N	0,001979	0,000585	0,001038	0,000236	0	0,00159
T	N	N	1,99E-05	0	0	0	0	0
D	D	N	8,69E-06	0	0,000035	0	0	4,75E-05
T	N	N	0	0	0	0	0	0
T	N	N	0,3022	0,0867	0,685	0,4287	0,2578	0,1389
.&D	D	D	0,008678	0,001468	0,008109	0,00698	9,35E-05	0,01327
.&T&T&.&'	N	N	4,43E-05	6,48E-05	5,79E-05	0	0	0
D&D&D&.	D	D						
D&D	D	N	1,65E-05	0	0	0	0	0
T&T	D	D	0,002196	0,000194	0,00113	0,01202	0,000111	0,000696
.&.&T&T	N	N	0,00928	0,000323	0,004373	0,007645	0,02704	0,009554
D&.	D	N	0,004991	0,000929	0,00229	0,0039	0	0,004204
.&.&.	D		0,000507	0	0,000858	0,00012	0	0
T	D	N	2,92E-05	0	0	0	5,51E-05	0
D&D	D	D	0,000922	0	0,001348	0,001244	0	0,00146
D	D	D	0,001388	0,000312	0,001794	0,002085	0	4,62E-05
T&T&T&.	D	N	0,002733	6,15E-05	0,002402	0,007245	0	0,001109
D&.	D	N						
T	D	N	4,01E-06	0	0	0	0	0
T&T&.	D	N	0,002235	0,000717	0,000956	0,003084	5,56E-05	0,001391
.&T&T&.	N	N	0,000201	6,89E-05	8,92E-05	0,000105	0	0
	N		0,01016	0,001542	0,004352	0,004195	5,59E-05	0,02667
T	N	N	3,38E-05	0,000133	8,35E-05	0	0	0
T	D	N						
D&.	N	N	6,24E-05	0,001118	9,81E-05	0	0	0
T&.	N	N	0,000429	0,005829	0,000651	0,000601	0,000119	0
D&.	N	N						
T&.	N	N						
T&.&.&T&	D	D	0,007291	0,001125	0,004759	0,003309	5,48E-05	0,01243
D&D&.&.	D	D	0,002379	0,000746	0,000179	0	0,000112	0,002005
T&.&T&T&	N	N	9,43E-05	0	0,000231	0,000119	9,25E-05	0
T&T&T&T	N	N	0,007881	0,001424	0,003124	0,02794	0	0,01321
D&.&.&.&.	D	N	0,000391	0	0,000261	0,000501	0	4,8E-05
.&T&T&.&'	N	N	0,003154	0,00037	0,003759	0,00129	0,000163	0,002957
D&D&D&..	N	N	0	0	0	0	0	0
.&T	D	D	0,009874	0,002481	0,006206	0,01417	0,000112	0,003677
.&T	N	N	0,001795	0,000123	0,000521	0,02034	0	0,000234
D&D&D	D	D	1,99E-05	0	5,78E-05	0	0	0
D&D&.	D	D	0,00112	0,000314	0,000758	0	0	9,58E-05
T&.	D	N	3,2E-05	0	2,9E-05	0	5,45E-05	0
.&D	N	N	0,006589	0,001047	0,002632	0,005161	5,44E-05	0,002496
T&.	N	N	0,000683	0,000797	4,36E-05	0	0,00039	0,000238
	N							
D&D	D	D	0,001909	0,000246	0,000694	0,00248	0	0,00097
D&D	N	N	1,95E-05	0	0	0	0	0
T&T&T	N	N	0,009749	0,002166	0,008485	0,02734	0	0,001214
D&T	N	N	0,01303	0	0	0,03333	0	0
D&T&.	D	D	0,001881	0,000246	0,000492	0	0	0,000464
T	N	N						

.&D	D	N	0,008636	0,00155	0,01134	0,02801	0	0,002996
T&.	N	N	0,002803	0,01094	0,002404	0,003406	0,001251	0,000185
T&T&.	N	N	0,003335	0,02119	0,002521	0,001291	0	0,000325
D&.	N	N	0,000536	0,006336	0,000231	0	0	0
.&T	N	N	0,000205	0,000127	0,000203	0	0	0
T	D	D	0,01143	0,001484	0,007443	0,02494	0	0,000511
T	N	N	0,001699	0,000199	0,000408	0,002826	0	0,000932
T&T	D	N	0,00101	0,000123	0,000665	0	0,000109	9,24E-05
T	D	N	0,001076	0,000431	0,000405	0,005859	0	0
D&D&D&D&[	D	D	7,96E-06	0	0	0	5,44E-05	0
	D		0,01077	0,001864	0,002518	0,03863	0	0,007646
T	D	N	3,98E-06	0	0	0	0	0
D&D	D	N	1,34E-05	0	3,15E-05	0	0	0
D&D	D	D	8,07E-06	0	0	0	0	0
T	D	N	0,000498	0,000185	0,000607	0	0,000163	9,24E-05
T	N	N	0,000449	0,000194	0,000377	0	0,000111	9,3E-05
D&D	D	N	0,009707	0,000931	0,002697	0,04625	5,56E-05	0,02048
D&D	N	N	0,006486	0,001334	0,00599	0,002932	0	0,009595
.&D&T	D	D						
D	N	N	0,002692	0,001973	0,001981	0,005037	0,001604	0,000869
D&.	N	N						
T&T&T&T	D	N	3,98E-06	0	0	0	0	0
D	N		0,01716	0,005093	0,03588	0,00717	0,05357	0,001279
T	N		0,00149	0,001319	0,006267	0	0	0,000615
T	N		0,00545	0,005823	0,01375	0,001807	0	0,00188
T	N	N	0,006789	0,001423	0,006631	0,03087	0	0,002098
T&T&T	N	N	0,008742	0,001599	0,005117	0,02183	0,000218	0,002634
T&T&.&T&	N	N	0,007142	0,001679	0,007012	0,000298	0	0,000835
T	D	D	0,002278	0,000246	0,001621	0,00149	0,000109	0,00074
T	D	N	1,59E-05	0	0	0	0	0
D&T	D	N	0,000844	0,000308	0,000203	9,93E-05	0	0,000231
D	D		0,002935	0,000554	0,000752	0,01161	0,000163	0,002541
T&T&.&.&	D	D	8,09E-06	0	2,92E-05	0	0	0
T	N	N	7,27E-05	0	4,09E-05	0	0	0
T&T&T&T	D	D	0,01228	0,001476	0,004654	0,01131	0,0318	0,001709
D	D	N	0,009147	0,002648	0,007361	0,01404	5,58E-05	0,002392
T	N	N	1,19E-05	0	0	0	0	0
D&D&D&[	D	D						
T	N	N	5,86E-05	0	9,4E-05	0	0	0
	N	N	0,006204	0,001931	0,003223	0,001323	0	0,01318
.&T&D&.	D	N	0,004305	6,23E-05	0,001649	0,000199	0	0,03429
D	D	D						
T&.&T	D	D	0,005647	0,003943	0,02151	0	0,02268	0
T&T	N	N	0,00517	0,02042	0,004395	0,03384	5,44E-05	4,62E-05
D	D	N	0,000627	0,00097	0,002232	1E-04	0	0
T	D	N	5,97E-05	0,000431	2,89E-05	0	0	0
D&D	D	D	8,61E-05	0,001248	2,91E-05	0	0	0
D	N	N	0,001407	0,009805	0,000492	9,94E-05	0,000163	0,001804
D&D	D	N	0,000597	0,005229	0,00052	0	0	0
T&T	D	D	0,000163	0,002276	0,000116	0	0	0

T&T	N	N						
T	N	N	0,004007	0,001369	0,003471	0,008082	0,000191	0,000674
T	N	N	0,007234	0,008946	0,01017	0,02974	0	0,001941
D&D&D	D	D	0,001629	0	0,000116	0	5,44E-05	0
T&T&T&T	D	N						
D	D	D						
T	D	N	7,67E-05	0	4,05E-05	0	0	0
T&T	N	N						
T&.&.&T&'	N	N	0,6428	0,7123	0,5929	0,6993	0,7109	0,5729
D	D	N	0,01022	0,000677	0,07222	0	0	0,000277
T	D	D	1,27E-05	0	0	0	0	0
T&.	N	N	0,2307	0,04069	0,3133	0,2141	0,2104	0,2594
T&D	D	D	0,7239	0,3008	0,6771	0,7431	0,7062	0,8439
T&.&T&T	N	N	0,7259	0,3517	0,6822	0,7479	0,7068	0,8366
T&T&T	N	N	0,2671	0,3428	0,254	0,3179	0,1677	0,2813
D&.&D	N	N	0,2105	0,2315	0,1119	0,2265	0,04595	0,2813
	N		0,01599	0,02315	0,01483	0,011	0,02259	0,005391
	N	N	0,001347	6,46E-05	0,001072	0,01054	0	0
.&T&T&T	N	N	0,9283	0,8159	0,9646	0,9528	0,9577	0,9163
D&.	D	D						
D&.&.&T&	D	N	0,006001	0,001538	0,003181	0,001786	0,000109	0,01331
.&.	N	N	2,03E-05	0	6,85E-05	0	0	0
T&.	N	N	0,000206	0,000129	0,000145	0	0,000111	0
T&T&T&T8	D	D	0,003845	0,000861	0,001937	0,003175	0	0,006698
.&T&T	D	D	0,009832	0,002001	0,006372	0,009342	0	0,01156
T&T	N	N						
T	N	N	0,00797	0,001845	0,004857	0,003572	0	0,02037
T	D	D	0,004989	0,001785	0,001331	9,92E-05	0,02218	0,001386
.&T&.&T&'	D	D	3,98E-06	6,16E-05	0	0	0	0
T&.&.&T&'	D	D						
T	N	N	0,008679	0,003306	0,003172	0,009331	0	0,01142
T&T&T&T	D	N	0,005415	0,001108	0,002778	0,02734	0	0,007495
T&T&T	N	N	0,000592	0,000329	0	0	0	0,001741
D	D	D	4E-06	0	0	0	0	0
T&.&T	D	D	0,003804	0,001086	0,004169	0,00081	0	0,002786
D&D&D&D&E	D	D	1,25E-05	0	0	0	0	0
T&.	N	N	0,000792	6,15E-05	0,000782	0	0,000109	0,000185
D&D&D&D&E	D	D	1,2E-05	0	0	0	0	0
.&T&D	D	D	0,004031	0,000904	0,002433	0,009237	0	0,004368
T&T&T	N	N						
T&T	N	N	1,2E-05	0	0	0	0	0
T&T&T&T8	D	N	4,03E-06	0	2,9E-05	0	0	0
T&T	N	N	0,002676	0,000806	0,001968	0,000498	0	0,001867
T&.&T	N	N	1,99E-05	0	0	0	0	0
T&.&T&T&	D	D	0,002191	0,000624	0,001922	0,000431	0	0,001268
T	N	N	9,94E-05	0,000884	0	0	0	0
T	D	D	0,00182	0,000273	0,001358	0,002384	0	0,000593
T&T	D	D	0,002323	0,000431	0,001157	0,002877	0	0,000693
T&T	D	N	7,56E-05	0	5,78E-05	0	0,000816	0

	N							
D	D	D	0,000642	0,000194	0,000116	0	0	0
D&D	D	D	0,008771	0,002338	0,003934	0,009728	5,44E-05	0,0213
T&T&.&T	N	N	0,005727	0,002154	0,004973	0,000199	0	0,000739
T&D&.&T	D	N	0,008951	0,003263	0,006567	0,001192	0,000109	0,004114
D&.&D&D	D	N	0,004239	0,001354	0,002462	0,008151	0	0,003014
D	D	N	0,000127	0,000123	0,000607	0	0,000272	0
D&D&D&D&	D	D	3,18E-05	0	0	0	0,000218	0
T&T&T	N	N	0,008218	0,002033	0,004464	0,01093	0	0,005277
T&T	N	N	0,001859	0,000631	0,000855	0,00258	0	0,001379
D	D	D	0,000171	0	0,000145	0	0	0
T&T&D&D	N	N	0,002509	0,000923	0,002342	0,005952	0,000272	0,001248
T&T	N	N	0,000163	0,000295	0,00019	0,000119	0,000154	0
T&D	N	N	0,008892	0,001436	0,002045	0,002077	0	0,008222
T&T	N	N	7,59E-05	0	0	0	0,000219	6,51E-05
T&T	N	N	0,000234	0,004278	0,000208	0	0	0
T&T	N	N						
D&T	N	N	0,005444	0,001012	0,001364	0,005848	0,000678	0,007506
T&T	N	N	0,03001	0,01357	0,02535	0,02172	0,02296	0,01924
D&D	N	N	0,001385	0,002196	0,000943	0,000294	0,000407	0,000148
T&T&.	N	N	9,56E-05	6,16E-05	2,9E-05	0	0	0
D&D&D	D	N	0,001172	0,00031	0,000318	0	0	0,000254
T&T	D	N	0,000935	0	0,00081	0,003671	0	0
D&D	D	D	0,009126	0,001547	0,004745	0,04865	0	0,008917
T&T	N	N	0,001611	0,000123	0,000405	0	0,000109	0
T&.	D	N	2,93E-05	0	0	0	0	0
D&.&D&D&	D	D	0,003499	0,000492	0,001823	0,001392	5,45E-05	0,000554
T&T&T&T&	N	N	0,007364	0,002003	0,005724	0,008549	0	0,001718
D&.&D	D	D						
.&.	D	D	4,02E-06	0	0	0	0	0
	D	D	0,005176	0,001681	0,002513	0,000409	0	0,003609
T&T	N	N	0,000036	0	0,000175	0	0,000109	0
D&D&.	D	D	0,003031	0,000738	0,001214	0	0	0,000508
.&D&D&D	D	D						
T&T&T	D	D	0,001036	0	0,000655	0,01464	0	4,65E-05
D&D&D&D&	D	D	0,00035	0	0,000116	0,001886	0	0
T&T&T&T	D	D	8E-06	0	0	0	0	0
D&D&D	D	D	1,69E-05	0	0	0	5,75E-05	0
	D	N	0,000372	0,000195	0,000412	0,000301	0	4,65E-05
T	D	N	2,81E-05	0	0	0	0	0
T	N	N	0,000624	0,003887	0,000167	0	0,000357	0,000328
D	N	N	0,009264	0,008	0,00561	0,006747	0,000218	0,02292
T&.&T	N	N	0,000119	0	0,000694	0	0	0,000185
D&D	D	D						
T&T&T	D	N						
D&D&D	D	N	1,99E-05	0	5,78E-05	0	0	0
T&.&T&T	D	N	2,4E-05	0	0	0	0	0
T	D	N	0,003964	0,000924	0,002054	0,000298	0	0,000416
T	N	N						
.&T	D	D	1,21E-05	0	0	0	5,52E-05	0

.&D	D	D	0,000132	6,46E-05	0,000261	0	0,000167	4,64E-05
.&D	D	D	0,000437	0,000777	0,000116	0	0	0,000464
D&D&D&D&[	D	D	0,003768	0,001093	0,00142	0	0	0,01617
T&.&T&T&	D	D	0,00801	0,001907	0,00437	0,002383	0,002176	0,01215
.&.&.&T&.	D	N	0,001647	6,15E-05	0,001417	0,01002	5,44E-05	4,62E-05
T&T	D	D	0,000553	6,15E-05	2,89E-05	0,000694	0	4,62E-05
D&D	D	D	0,00012	0	0	0	0	0
D&D&D&D&[	D	D	0,000544	0,000357	0,000124	0,00132	0	4,74E-05
D	N	N	0,003862	0,000615	0,00107	0,01042	5,44E-05	0,001663
D&.	D	N	6,36E-05	0	0,000231	0	0	0
.&T	D	D	0,000193	0,001002	0,000175	0,000203	0,000278	0
.&T	N	N	0,00191	0,000323	0,000753	0	0	0,000835
.&T	N	N	0,008356	0,001847	0,007572	0,008591	5,59E-05	0,01046
T&T&T&T	N	N						
T	D	D	0,00907	0,001661	0,007198	0,008533	5,44E-05	0,004943
D&D	D	D	0,000911	0,000185	0,000434	0	0	0,000416
D	D	D	0,003353	0,00052	0,001772	0,001296	0	0,002827
T&T&T&T	D	D						
T	D	N	0,003532	0,000803	0,003423	0	0,00157	0,001773
D&D	D	D						
T&T&T&T&	N	N	0,005132	0,000715	0,008285	0,008377	0	0,00028
D	N	N	0,009133	0,001476	0,005897	0,01776	0,000109	0,01136
.&T&T	D	D	3,98E-06	0	0	0	0	0
T&.&T	D	D	0,005386	0,001046	0,002573	0,006251	0,000272	0,007116
T	N	N	0,007698	0,01313	0,01076	0,003883	0,01932	0,000954
T	N	N	0,01073	0,00115	0,00715	0,004572	0,001247	0,003902
T&T&.	N	N	0,7425	0,8373	0,781	0,89	0,8438	0,7342
T&T&.	N	N	0,7246	0,7572	0,7641	0,8589	0,842	0,7246
T&T&.	N	N	0,7392	0,8337	0,7775	0,886	0,8411	0,7324
D&D&.	N	N	0,09838	0,1789	0,1547	0,1532	0,04163	0,1234
T&T&.	N	N	0,7427	0,8194	0,7828	0,8874	0,8328	0,7271
T&T&.	N	N	0,7317	0,7811	0,7683	0,8722	0,8243	0,715
T&T&.	N	N	0,5182	0,6527	0,562	0,7908	0,6751	0,5719
T&T&.	N	N	0,5416	0,5897	0,5756	0,7945	0,6936	0,5763
D&D&.	N	N	0,08277	0,1355	0,1336	0,1347	0,04862	0,1198
T&T&.	N	N	0,6732	0,7621	0,7142	0,8999	0,7765	0,6565
T&T&.	N	N	0,7227	0,7406	0,7565	0,8533	0,8334	0,7082
T&T&.	N	N	0,3279	0,3422	0,3892	0,2465	0,5777	0,4514
T&T&.	N	N	0,7234	0,7452	0,7542	0,8455	0,8112	0,7089
T&T&.	N	N	0,3363	0,3243	0,3284	0,4564	0,3343	0,3355
T&T&.	N	N	0,4233	0,4353	0,4084	0,4966	0,4835	0,3851
T&T&.	N	N	0,2514	0,3573	0,3294	0,3787	0,1798	0,2109
D&D	N	N	0,05348	0,104	0,09519	0,07039	0,02676	0,07837
T&T	N	N	0,05457	0,1079	0,09154	0,06163	0,05622	0,06577
T	N	N	0,2304	0,3355	0,1889	0,24	0,2617	0,2232
T&.	D	D	0,002994	0,01286	0,002429	0,005463	0,001849	0,00088
T&.&T&T&	D	D						
D&T&T	D	D	0,008153	0,001108	0,00561	0,005262	0,000163	0,007747
T	D	D	0,001804	0,02225	0,002226	0	0	0
D&D	D	N	0,001388	0,0163	0,001735	0	0	0

T	D	D	0,002311	0,0285	0,001449	0	0	0
T&T	N	N	0,00012	0	9,33E-05	0	0	0
D&D&D&D&D	D	D	3,18E-05	0,000123	2,89E-05	0	0	0
T	N	N	0,008136	0,001355	0,01309	0,000397	0,001525	0,009623
	N	N						
.&.&T	D	D	0,001062	0,000246	0,000463	0,001786	0	0
T&T&T&D&	D	D	0,000299	0,000123	0,000202	0	5,44E-05	0
T	N	N	7,95E-06	0	0	0	0	0
D	N	N	0,000918	0,000324	0,000812	0	0	0,000517
T&.	N	N	0,000187	0	0,000122	0	0	0
T	D	D	0,004418	0,00143	0,00483	0,00041	0	0,001845
T&T&T&T&	N	N	0,001795	0,000205	0,00051	0,000415	5,65E-05	0,001612
T&.	N	N	0,00024	0	0	0,004884	0	0
.&T&.	N	N	0,002172	0,000431	0,004511	0,009228	5,44E-05	0
.&D&.	D	D	0,008733	0,003682	0,004432	0,01074	5,56E-05	0,00339
T&T	N	N	0,01358	0,002283	0,003506	0,008165	5,44E-05	0,02072
D&D&.&D	D	D	2,42E-05	6,16E-05	5,82E-05	0	0	0
D&D&D&D&D	D	N	7,96E-06	0	2,89E-05	0	0	0
D&D&D&D&D	D	D	0,006826	0,00123	0,005204	0,005952	5,44E-05	0,005867
T	N	N	0,000642	0,004398	0,000842	0,000134	0,000829	9,19E-05
T&T&D	D	D						
T&.&T&T&	D	N	0,009388	0,001748	0,007143	0,02151	0	0,007945
T&T&T&.	N	N	0,008243	0,001729	0,009959	0,009106	0	0,004747
T&T&D&T&	N	N	1,59E-05	0	5,78E-05	0	0	0
T&.&.	N	N	0,006206	0,001668	0,007145	0,0137	0,000111	0,002229
T&T&T&T	D	N	0,000389	0	0,000232	0,004369	0	0
T&T	D	N						
D	D	N	0,003359	0,000371	0,001602	0,01246	0,000218	0,000231
T&T	N	N	0,002316	0,000492	0,001217	0,001716	0	4,65E-05
D&D&D&D&D	D	D	0,000183	0	0,000232	9,93E-05	0	0
T&T	D	D	1,21E-05	0,000105	0	0	0	0
T&.	N	N	1,31E-05	0	0	0	0	0
D	D	D	0,00012	6,16E-05	0	0	0	0
D&.	D	D	0,008331	0,0016	0,002315	0,02056	0,000109	0,000601
D&.&D&D&	D	D	3,98E-05	0,000185	0	0	0	0
T&.&T&T	N	N	2,09E-05	0	0,000115	0	0	0
T&D	N	N	3,98E-05	0,000246	0,000116	0	0	0
.&T&T&.	N	N	0,01103	0,00129	0,007849	0,03245	0	0,01052
T&D	N	N	6,88E-05	0	8,74E-05	0	0	9,32E-05
D&D&D	D	D	0,004232	0,001255	0,004225	0,01038	0	0,00108
D&D	D	D	1,2E-05	0	5,78E-05	0	0	0
T	N	N	0,002229	0,02533	0,00223	0	0,000337	4,66E-05
D&D&D&D&D	D		0,001664	0,001748	0	0	0	0,01546
D&D&D&D&D	D	N	0,008741	0,002215	0,005666	0,01806	0,000218	0,01104
D&D	D	D	0,007322	0,001694	0,005821	0,01693	7,3E-05	0,004661
T&T&T&T	D	N	0,004762	0,00037	0,003588	0,01629	0	0,001849
T&T&T&T&	D	D	5,17E-05	0	2,89E-05	9,92E-05	0	0
T&.&T&T&	D	N	4,94E-05	0	0	0	0	0
T&T	D	N	6,4E-05	0	0	0	0	0
D&D	D	D						

T&T&T&T	D	N	9,54E-05	0,000492	0	0	0	4,62E-05
T	N		1,41E-05	0	0	0	0	0
T	N	N	0,01014	0,002288	0,006511	0,0138	5,47E-05	0,007319
D	N	N	0,001015	0	0,000655	0,01002	0	0
D	D	N	0,00012	0	0	0	0	0
D&D	D	D	5,2E-06	0	0	0	0	0
T&T&T	N	N	4,03E-06	0	0	0	0	0
	N		0,1983	0,5975	0,3317	0,1028	0,5628	0,05551
T&T	N	N	0,01174	0,003223	0,009731	0,06177	5,45E-05	0,005782
T	D	N	0,001293	0,000185	0,00107	0,002183	5,44E-05	0,000185
T&T&T	D	N	0,000175	0	0,000174	9,93E-05	0	0
T&.&T	N	N	9,25E-05	0,00013	5,79E-05	0	0,000223	0
T&T	N	N	0,002915	0,000821	0,001564	0,0003	0,000164	0,000278
T	D	N						
T&T	N	N						
T&T&T&T8	D	N	0,007066	0,001495	0,008429	0,01095	0	0,006145

D	N		0,007698	0,00152	0,00299	0,009086	0	0,01744
D	D		2,18E-05	0	0	0	0	0
T	N							
T	N		5,45E-06	0	0	0	0	0
T	N		0,005843	0,00055	0,001958	0,002234	0	0,002017
T	N		0,002775	0,003138	0,003891	0,001412	0,005521	0,00094
T	N		0,000296	0,000314	0,00047	0,000546	0,0003	0
T	N		0,000241	0,000168	0,00048	0,000279	0,000469	0
D&D	N		0,001273	0,000266	4E-05	0,000141	0	0,000276
T&T&T	D		0,000133	0	0	0	0	0
T&T&T	N		0,00358	0,00076	0,004277	0,000671	0	0,00233
D&D	D		1,67E-05	0	3,69E-05	0	0	0
D	D	D	0,000232	0	0	0	5,45E-05	0
T	D	N						
D	D	N	0,003586	0,000636	0,004314	0,006041	0	0,003571
D	D	D	0,001275	0,000369	0,001822	0	0	0
.&D	D	D	0,003177	0,000313	0,000378	0	0	0,01868
T&T&.	D	N	0,000151	0	0,000405	0	0	0
D	D	N	0,002534	0,000185	0,002821	0,02023	0,001252	0
D&T	D	D	0,001113	0,000186	0,001013	0,002884	0	0,00051
.&D&.	D	D	7,97E-06	0	0	0	5,44E-05	0
T	D	N	0,004592	0,000758	0,005512	0,002464	5,87E-05	0,003034
D&.	D	D						
D&D	N	N						
.&T&T	D	D	1,19E-05	6,15E-05	2,89E-05	0	0	0
T&T&T	N	N	0,002392	0,001603	0,000814	0,003222	0,001852	0,002784
T&T	D	D	4,39E-05	0,000185	0	0	0,000109	0
T&T&.	N	N	0,09205	0,2449	0,1421	0,1743	0,04958	0,116
T&T&T&T8	D	N	0,002424	0,000555	0,003761	0,002188	0	0,000278
T&T	N	N	0,005578	0,001886	0,003174	0,01024	0	0,001521

			0	0	0	0	0	0
			1,59E-05	0	0	0	0	0
			0,001207	0,000432	0,000116	0,001094	0	0,001943
			4,36E-06	0	0	0	0	0
			0,003881	0,00123	0,002719	0,002381	0,004797	0,009576
	N	N						
.&.&.		N						
	N	N						
owth_factor-beta&_C-		D						
.&.	N	N						
	N							
.&.&.	D							
.&.&.	N		0,00103	0,0138	0,000723	0	0	0
.&.	D	N						
	D	N	0,006266	0,000985	0,031	0,004411	0,002732	0,000326
.&.	D	N	0,001206	0,000492	0,000753	0,002581	0	0,000139
.&.&.&.	N		4,38E-05	6,15E-05	0	0	0	0

mADe_NFE	mADe_OTH	mADe_SAS	AD_exome	AD_exome	AD_exome	AD_exome	xomes_PC	xomes_PC
0,000224	0,000226	0,001383						
0,000214	0,000213	0,001126						
8,83E-06	0	0						
0,000868	0,000746	0,000175						
0,000146	0,000423	0,00041						
0,000204	0,000453	0,00043						
0,002228	0,005004	0,002328						
0,000481	0,000234	0,000925						
0,002629	0,005774	0,01849						
8,17E-05	0	0,000662						
0,006692	0,01473	0,04236						
0,002734	0,001968	0						
0,003552	0,004866	0,005841						
0,00267	0,003265	0,005995						
0,001231	0,003257	9,8E-05						
0,01477	0,009983	0,002327						
0,000172	0	0						
0,00359	0,002273	0,000582						
2,64E-05	0	0						
0,005923	0,006727	0,006433						
0,00045	0	0						
0,009949	0,007423	6,54E-05		1788	0,007165	249538	1127	0,009949
0,000106	0,000163	0		19	7,56E-05	251482	6	0,000173
5,28E-05	0	9,8E-05		13	5,17E-05	251444	2	0,000123
0,000106	0	3,27E-05		21	8,35E-05	251438	8	0,000231
0,004288	0,00866	0,04176		1956	0,012675	154320	951	0,041758
0,000757	0,003939	0,01564		392	0,002806	139678	308	0,015644
0,000502	0,000327	0		59	0,000235	251288	57	0,000502
0,007934	0,00588	0,00077	segdup	979	0,004362	224432	755	0,007934
0,001136	0,001958	0,001535		407	0,00162	251230	84	0,002429
0	0	0		1	3,98E-06	251336	1	5,44E-05
0,000115	0,000248	0		36	0,000255	140958	6	0,000115
0,003141	0,000654	0		398	0,001587	250798	356	0,003141
0,001444	0,004239	0,003593		491	0,001954	251300	110	0,003593
0,000572	0,000163	0,000294		86	0,000342	251382	65	0,000572

8,85E-06	0	0		2	8,01E-06	249788	1	2,93E-05
0,008404	0,007999	0,003988		1459	0,005809	251180	955	0,008404
0,009627	0,006917	0,001308		1499	0,006024	248844	1088	0,009627
0	0	0						
7,16E-05	0,000228	0		11	7,31E-05	150390	2	0,000287
0,003274	0,005879	0,03267		1896	0,007545	251282	1000	0,032669
7,05E-05	0	0		9	3,59E-05	251032	8	7,05E-05
1,76E-05	0	0		2	7,96E-06	251268	2	1,76E-05
0,1624	0,2489	0,1068	segdup	24620	0,211381	116472	4958	0,654954
0,000255	0,000163	0		36	0,000143	251378	29	0,000255
0,01662	0,05114	0,05089	segdup	8097	0,036483	221936	2375	0,165022
0,001416	0,006358	0,000916	segdup	1336	0,005314	251412	181	0,00984
0,007841	0,003909	0,001404		1083	0,004307	251470	892	0,007841
0,00059	0,000163	0		160	0,000637	251252	67	0,00059
0,003018	0,001317	0		454	0,001816	249942	341	0,003018
0,00573	0,006564	0,001144		1034	0,00414	249748	213	0,006168
1,76E-05	0	0		2	7,96E-06	251328	2	1,76E-05
0,01058	0,007379	0,005357		1824	0,007277	250658	1197	0,010581
0,006334	0,003296	0,01314		1301	0,005215	249482	400	0,013141
0,000556	0	0,000298	segdup	53	0,000438	121114	15	0,001735
1,76E-05	0	0		2	7,96E-06	251144	2	1,76E-05
0,004029	0,004742	0,006773		1047	0,004174	250818	207	0,006773
1,76E-05	0	0		2	7,96E-06	251156	2	1,76E-05
0,000418	0,000663	0,00415		200	0,000804	248614	127	0,00415
0,000204	0,000166	0,000229		32	0,000129	248442	7	0,000229
0,01073	0,004241	0,002712		1763	0,007017	251256	1220	0,010734
1,76E-05	0	0		3	1,19E-05	251372	1	2,89E-05
8,8E-06	0	0	segdup	1	3,98E-06	251208	1	8,8E-06
0,003079	0,006344	0,00093	segdup	622	0,002605	238814	123	0,003639
2,64E-05	0,000163	0		4	1,59E-05	251354	3	2,64E-05
0,009638	0,01091	0,01325	segdup	3741	0,01502	249076	1838	0,102236
8,95E-06	0	0		1	4,03E-06	247898	1	8,95E-06
0,002619	0,000977	0						
0,002614	0,000692	0,000216	segdup	143	0,002818	50752	48	0,002614
0,000541	0,001176	0,000328	segdup	160	0,000655	244140	60	0,001755
9,65E-05	0	0	segdup	10	4,32E-05	231436	10	9,65E-05
0,000775	0,000489	0		94	0,000374	251300	88	0,000774
0,000354	0,00066	0		206	0,000826	249360	111	0,00717
0,01289	0,007993	0,00307	segdup	2288	0,009112	251090	352	0,019197
0,007343	0,005184	0,000855		1183	0,004793	246830	821	0,007343
0,006697	0,003588	0,000196		945	0,003761	251246	761	0,006697

0	0	0		1	4,02E-06	248584	1	5,56E-05
0,008989	0,005537	0,000817		1370	0,00545	251394	1022	0,008989
1,76E-05	0	3,29E-05		7	2,81E-05	249402	3	0,000185
0,000186	0,000819	0,001177		154	0,000615	250312	71	0,004404
0,003819	0,004734	0,003039		770	0,003068	251016	433	0,003819
0,00015	0	0		24	9,57E-05	250892	7	0,000203
0,0016	0,001652	0,000178		161	0,001105	145714	42	0,001727
0,004662	0,01046	0,02048	segdup	1468	0,006297	233142	597	0,020483
0,006293	0,005463	0,001818		755	0,004236	178216	514	0,006293
0,0113	0,009698	0,004943		663	0,009145	72496	292	0,011302
0,002152	0,004932	0,007917		880	0,003653	240902	240	0,007917
0,1533	0,1808	0,282		44292	0,176881	250406	8621	0,281953
0,8281	0,7969	0,6316		116783	0,79711	146508	21742	0,885405
7,09E-05	0	0,000163		26	0,000104	249136	11	0,000319
8,8E-05	0	0,000392		23	9,15E-05	251390	12	0,000392
0,01539	0,01665	0,004867	segdup	2516	0,010018	251140	1748	0,015388
0,004993	0,002617	0,001241	segdup	736	0,002934	250880	566	0,004993
0,005729	0,002803	0,006503		1007	0,004035	249578	199	0,006503
7,94E-05	0	0	segdup	9	3,59E-05	250810	9	7,94E-05
0,000221	0	0,000131	segdup	47	0,000187	250838	9	0,00049
0,000992	0,002099	0,000991		170	0,000745	228190	102	0,000992
0,01103	0,007497	0,001666		1728	0,006873	251402	1254	0,011027
8,79E-06	0	0		5	1,99E-05	251476	2	0,000109
0	0	0		5	1,99E-05	251472	4	0,000217
2,76E-05	0,000335	6,7E-05		8	3,28E-05	243898	2	6,7E-05
0	0	0		1	3,98E-06	251424	0	0
3,54E-05	0	6,54E-05		8	3,21E-05	248986	2	6,54E-05
0,009141	0,00748	0,009221		1144	0,007289	156948	210	0,009221
0,01324	0,01355	0,003173		2260	0,00908	248886	1493	0,013237
0,00189	0,001303	0,000131		302	0,001201	251490	66	0,001908
0,000181	0	0		11	7,88E-05	139638	10	0,000181
7,87E-05	0,000207	0		13	7,2E-05	180578	1	0,000101
0,000123	0,000326	0		98	0,00039	251476	77	0,004737
0,001503	0,000652	0,000947		230	0,000915	251486	171	0,001503
4,44E-05	0,000165	3,29E-05		8	3,21E-05	249576	5	4,44E-05
0,009192	0,01533	0,01671		2383	0,009495	250980	511	0,016715
3,58E-05	0,000332	5,96E-05						
0,000226	0,000176	0,000494	segdup	45	0,000193	233540	14	0,000494
0,000495	0,000685	0,00069		105	0,000432	242852	20	0,00069
0,001964	0,003424	0,000653		312	0,001242	251240	223	0,001964

0,000239	0,000171	3,34E-05		51	0,000212	240920	26	0,000239
0,0449	0,03896	0,01832	segdup	452	0,024257	18634	287	0,0449
0,006786	0,005537	0,005324	segdup	1233	0,004903	251490	772	0,006786
0,00225	0,002769	0,00258		572	0,002274	251490	79	0,00258
8,8E-05	0,000163	0		11	4,38E-05	250898	10	8,8E-05
0,008594	0,007221	0,01395		1557	0,006786	229458	389	0,013946
3,87E-05	0	3,8E-05		5	2,24E-05	222912	4	3,87E-05
0	0	3,99E-05		1	4,4E-06	227434	1	3,99E-05
0,000255	0,000654	0,000655		107	0,000426	251116	29	0,00084
0,0132	0,01226	0,00095		2328	0,009365	248572	1487	0,013203
0,000211	0,000349	0,000175		55	0,00023	239572	11	0,000333
0,008644	0,0143	0,04707		2727	0,010985	248246	1429	0,047072
8,89E-06	0	0		1	4,01E-06	249282	1	8,89E-06
0,001869	0,000683	0		131	0,000836	156674	113	0,001869
0,000154	0,000332	9,82E-05		27	0,00011	246216	17	0,000154
2,7E-05	0	0		3	1,21E-05	248922	3	2,7E-05
0,004933	0,006704	0,01417		2395	0,00955	250796	1306	0,037842
0,001461	0,000905	0		176	0,000764	230318	158	0,001461
0,000245	0,00069	3,29E-05		163	0,000694	234904	25	0,000245
0,000211	0,000652	3,27E-05		167	0,000664	251480	24	0,000211
1,79E-05	0	0		2	8,19E-06	244254	2	1,79E-05
0,000428	0,001298	0,000143	segdup	132	0,000587	224770	25	0,001872
0,01071	0,01433	0,003345	segdup	454	0,00676	67160	272	0,010708
7,92E-05	0,000654	3,27E-05		337	0,001357	248252	271	0,016677
0	0	3,27E-05		145	0,000585	247802	127	0,007871
0,000378	0,000977	0		136	0,000541	251490	34	0,002092
1,76E-05	0	0		2	7,97E-06	251038	2	1,76E-05
0,007147	0,003653	0,000725	segdup	1070	0,00431	248268	804	0,007147
1,76E-05	0	0,001209		39	0,000155	251490	37	0,001209
0	0	3,31E-05		6	2,4E-05	249776	5	0,000148
0,01035	0,01076	0,004574		1872	0,007451	251232	1175	0,010345
0,000487	0,000828	3,28E-05		81	0,000325	248862	55	0,000487
0,0017	0,003436	0,001267		398	0,002664	149426	115	0,0017
0,007656	0,006884	0,001275		753	0,004874	154508	454	0,007656
0,000646	0,000331	0		80	0,000321	249228	73	0,000646
0,01977	0,0298	0,01639	segdup	3161	0,018605	169902	900	0,034892
0,01339	0,0179	0,01581	segdup	1959	0,011568	169348	325	0,015809
0,0282	0,03046	0,01942	segdup	2258	0,016008	141058	1435	0,028204
0,01088	0,01804	0,006456	segdup	1697	0,009321	182060	537	0,01911
0,02849	0,03986	0,02061	segdup	3988	0,023252	171510	932	0,035319
2,3E-05	0	3,98E-05						
0,000675	0,004084	0,000828	segdup	136	0,001174	115842	49	0,007454
0,000409	0,000753	0,000851	segdup	36	0,001	35994	17	0,005108
0	0	0						

0,009589	0,00331	0,00369		784	0,005695	137658	519	0,009589
0,000397	0,000483	0		35	0,00026	134578	21	0,000397
0,003104	0,006358	0		555	0,002208	251408	133	0,003848
0,003284	0,004806	0,001049	segdup	643	0,002617	245748	119	0,003456
0,000044	0	0		8	3,18E-05	251280	3	8,68E-05
0,002568	0,002167	0,000706		362	0,001908	189738	233	0,002568
0,0053	0,003466	0,000792		744	0,003304	225174	553	0,0053
2,64E-05	0	0		3	1,19E-05	251366	3	2,64E-05
0,008871	0,004215	0,007375						
0,01059	0,009289	0,001927		1621	0,00645	251336	1203	0,010585
0,005802	0,002437	0,004439		280	0,003462	80872	174	0,005802
9,18E-06	0	6,62E-05		3	1,24E-05	241720	2	6,62E-05
7,97E-05	0	0		10	4E-05	250234	9	7,97E-05
0	0,000208	0		1	5,4E-06	185254	0	0
0,005123	0,0031	0		722	0,002874	251226	582	0,005123
0,002291	0,004837	0,001687		563	0,00251	224286	92	0,002839
0,000288	0,000164	0,000164		46	0,000185	248014	32	0,000288
0,000185	0,000163	0,000327		40	0,000159	251456	10	0,000327
0	0	0		3	1,47E-05	203502	2	0,000168
0,008585	0,005387	0,00575		1410	0,005613	251202	975	0,008585
1,77E-05	0	0,000229		9	3,61E-05	249544	7	0,000229
0,002075	0,00214	0,002897	segdup	452	0,001815	248968	88	0,002897
0,000178	0	9,83E-05	segdup	26	0,000104	249428	20	0,000178
0,005562	0,004407	0,003661		889	0,003538	251274	632	0,005562
0,001011	0,001792	0,003789		309	0,001229	251490	116	0,003789
0,007884	0,007665	0,000196		1568	0,006241	251226	895	0,007884
2,69E-05	0	0		3	1,22E-05	245310	3	2,69E-05
0,07413	0,09189	0,06751	segdup	772	0,07351	10502	275	0,133366
0,0008	0,000166	0	segdup	107	0,000432	247846	90	0,0008
4,47E-05	0	0		5	2,03E-05	246252	5	4,47E-05
7,96E-05	0	0	segdup	26	0,000104	250590	2	0,000109
0,008537	0,005865	0,001241		1397	0,005555	251468	971	0,008537
0,000118	0,00017	6,8E-05		29	0,00012	241178	13	0,000853
1,65E-05	0	0		2	1,28E-05	155914	1	0,000125
0,000141	0,000164	6,54E-05		22	8,78E-05	250606	16	0,000141
3,52E-05	0	3,27E-05		17	6,76E-05	251494	10	0,000544
8,9E-06	0	0		1	4,01E-06	249654	1	8,9E-06
9,47E-06	0	3,33E-05		2	8,42E-06	237566	1	3,33E-05
1,82E-05	0	4,41E-05		3	2,18E-05	137908	1	4,41E-05
6,37E-05	0,000167	0		8	3,33E-05	240084	7	6,37E-05
1,79E-05	0	0		2	8,08E-06	247506	2	1,79E-05
0,00313	0,003589	0,000849		493	0,001961	251392	356	0,00313
0,000124	0	0		15	6E-05	249986	14	0,000124
0,000114	0,000163	0,009995	segdup	320	0,001272	251488	306	0,009995
8,8E-06	0	0		1	3,98E-06	251268	1	8,8E-06
0,000167	0,001303	9,8E-05		417	0,001658	251496	324	0,019931

1,77E-05	0	0		2	8,03E-06	249208	2	1,77E-05
0,003148	0,001538	0,002158		330	0,001979	166772	212	0,003148
4,4E-05	0	0		5	1,99E-05	251360	5	4,4E-05
0	0	0		2	8,69E-06	230076	1	3,5E-05
0	0	0						
0,1973	0,4094	0,5542	segdup	29012	0,302215	95998	8671	0,685021
0,01338	0,01203	0,00128		1283	0,008678	147842	746	0,013382
5,31E-05	0	6,54E-05		11	4,43E-05	248498	2	6,54E-05
2,77E-05	0,000167	0	segdup	4	1,65E-05	242742	3	2,77E-05
0,002878	0,001981	0,00098		548	0,002196	249540	326	0,002878
0,00241	0,01122	0,03431		2316	0,00928	249562	1050	0,034311
0,007208	0,007223	0,0055		1245	0,004991	249440	810	0,007208
0,000426	0,001418	0,000845		71	0,000507	139964	21	0,000858
5,49E-05	0	0		7	2,92E-05	239574	1	5,51E-05
0,000852	0,001088	0,000569		57	0,000922	61836	14	0,001348
0,002055	0,001642	0,000523		348	0,001388	250742	233	0,002055
0,003677	0,005545	0,001764		687	0,002733	251340	418	0,003677
8,85E-06	0	0		1	4,01E-06	249266	1	8,85E-06
0,00329	0,002645	0,002091		555	0,002235	248272	369	0,00329
0,000317	0,001042	0,000103		47	0,000201	233910	33	0,000317
0,01361	0,009993	0,005026		2472	0,010164	243218	1482	0,013606
1,77E-05	0	4,42E-05		5	3,38E-05	147914	1	0,000133
1,07E-05	0	7,15E-05		13	6,24E-05	208252	7	0,001118
0,000322	0,000673	0,000125		79	0,000429	184026	23	0,005829
0,01057	0,01006	0,002816		1814	0,007291	248796	1183	0,010565
0,004254	0,002051	0,001564		563	0,002379	236694	444	0,004254
0,000118	0	0						
0,01089	0,007379	0,000327		1952	0,007881	247678	1208	0,010894
0,000401	0,001155	0,000981		97	0,000391	248232	30	0,000981
0,004841	0,003915	9,8E-05		792	0,003154	251074	549	0,004841
0	0	0						
0,0138	0,01365	0,01122	segdup	2446	0,009874	247720	1548	0,013802
0,001646	0,003115	0,000523		449	0,001795	250160	186	0,001646
1,76E-05	0	3,27E-05		5	1,99E-05	251426	2	5,78E-05
0,002105	0,000831	0,000132		277	0,00112	247234	235	0,002105
4,44E-05	0	3,27E-05		8	3,2E-05	249870	1	5,45E-05
0,008041	0,007339	0,01575	segdup	1656	0,006589	251326	482	0,015746
0,001254	0,000257	0	segdup	119	0,000683	174244	102	0,001254
0,003412	0,002609	6,53E-05		480	0,001909	251432	388	0,003412
5,03E-05	0	0		3	1,95E-05	153922	3	5,03E-05
0,01499	0,0138	0,001284		1333	0,009749	136726	790	0,014985
0,01389	0	0,01042		8	0,013029	614	6	0,013889
0,002932	0,001145	0,003332		471	0,001881	250428	102	0,003332

0,008892	0,01326	0,00702	segdup	1448	0,008636	167666	303	0,011344
0,003085	0,002635	0,00085	segdup	697	0,002803	248650	164	0,010942
0,002473	0,002145	0,003399		832	0,003335	249490	328	0,021186
0,000204	0,000329	3,27E-05	segdup	134	0,000536	249984	100	0,006336
0,000331	0	0,000264		32	0,000205	156238	20	0,000331
0,007007	0,0175	0,04605		2849	0,011428	249298	1409	0,046046
0,002641	0,00156	0,001533		409	0,001699	240776	292	0,002641
0,001011	0,000815	0,00343		254	0,00101	251470	105	0,00343
0,0009	0,002121	0,002452		270	0,001076	251004	75	0,002452
8,8E-06	0	0		2	7,96E-06	251298	1	5,44E-05
0,01012	0,01104	0,02636		2690	0,010766	249870	807	0,026364
8,8E-06	0	0		1	3,98E-06	251214	1	8,8E-06
2,01E-05	0	0		3	1,34E-05	223580	1	3,15E-05
1,78E-05	0	0	segdup	2	8,07E-06	247930	2	1,78E-05
0,00037	0,000327	0,0017		125	0,000498	251032	52	0,0017
0,000477	0,000165	0,001209		112	0,000449	249452	37	0,001209
0,00873	0,01025	0,01163		2378	0,009707	244970	329	0,01163
0,01021	0,009839	0,000264		1026	0,006486	158184	629	0,010215
0,00358	0,005487	0,001298	segdup	645	0,002692	239586	381	0,00358
8,79E-06	0	0		1	3,98E-06	251282	1	8,79E-06
0,01939	0,02921	0,02559	segdup	564	0,017163	32862	3	0,053571
0,0006	0,005671	0,002418	segdup	56	0,00149	37594	23	0,006267
0,00325	0,01559	0,01186	segdup	196	0,00545	35962	49	0,013749
0,008847	0,01032	0,001079		1685	0,006789	248184	982	0,008847
0,009278	0,009612	0,0196		2198	0,008742	251426	600	0,019599
0,01155	0,008612	0,004412		1778	0,007142	248948	1302	0,011545
0,004036	0,003428	0		572	0,002278	251066	458	0,004036
3,52E-05	0	0		4	1,59E-05	251180	4	3,52E-05
0,0017	0,000163	0		212	0,000844	251154	193	0,0017
0,004336	0,003426	0,000457	segdup	738	0,002935	251416	493	0,004336
8,94E-06	0	0		2	8,09E-06	247142	1	2,92E-05
0,00013	0,000475	0		10	7,27E-05	137468	7	0,00013
0,008017	0,01059	0,03887	segdup	3088	0,012279	251482	1190	0,038869
0,01324	0,009278	0,00781		2273	0,009147	248506	1490	0,013235
1,76E-05	0	3,27E-05	segdup	3	1,19E-05	251458	1	3,27E-05
8,93E-05	0,000179	0		13	5,86E-05	221770	3	9,4E-05
0,01003	0,005698	0,001999		898	0,006204	144746	529	0,010026
0,002154	0,003759	0,000294		1077	0,004305	250192	243	0,002154
0,000115	0,002946	0,005331		1416	0,005647	250736	416	0,022683
0,003552	0,008472	0,000555	segdup	1300	0,00517	251448	332	0,020423
0,000498	0,000995	0		155	0,000627	247210	77	0,002232
5,28E-05	0,000163	0		15	5,97E-05	251386	7	0,000431
0	0,000168	0		21	8,61E-05	243916	19	0,001248
0,000379	0,001471	0,00268	segdup	353	0,001407	250928	159	0,009805
0,000334	0,000815	0,000131		150	0,000596	251484	85	0,005229
0	0	0		41	0,000163	251370	37	0,002276

0,006448	0,005962	0,000371		814	0,004007	203160	562	0,006448
0,007122	0,01372	0,001881		1211	0,007234	167406	239	0,010168
1,76E-05	0,001307	0,01287		409	0,001629	251014	394	0,012872
0,000182	0	0		12	7,67E-05	156508	11	0,000182
0,6427	0,6387	0,6536		161537	0,642836	251288	11568	0,712315
0,00015	0,006189	0		2570	0,010221	251438	2498	0,072222
2,73E-05	0	0		3	1,27E-05	236306	3	2,73E-05
0,2541	0,2426	0,1392		51876	0,230736	224828	10217	0,31327
0,8139	0,7568	0,5802		181913	0,723922	251288	92505	0,813875
0,814	0,7617	0,582		161913	0,725931	223042	81724	0,813984
0,2739	0,2856	0,2482		54207	0,267143	202914	5145	0,342817
0,2686	0,2214	0,1387		52546	0,210464	249668	30170	0,268555
0,01868	0,01119	0,01103	segdup	409	0,015992	25576	32	0,023155
0,00135	0,003479	0,00059	segdup	335	0,001347	248732	152	0,00135
0,9319	0,9307	0,909	segdup	185101	0,928281	199402	28721	0,964634
0,008818	0,007655	0,000523		1509	0,006001	251438	1003	0,008818
2,2E-05	0	0						
0,000325	0,000496	9,8E-05	segdup	51	0,000206	247060	36	0,000325
0,005512	0,005863	0,001502	segdup	967	0,003845	251476	627	0,005512
0,01495	0,0104	0,003431		2451	0,009832	249280	1689	0,014945
0,01103	0,007494	0,000947		2004	0,00797	251438	1254	0,011027
0,002129	0,002445	0,01578		1254	0,004989	251336	408	0,022184
0	0	0		1	3,98E-06	251194	1	6,16E-05
0,01482	0,01059	0,00283		787	0,008679	90680	531	0,014823
0,005581	0,006052	0,004541	segdup	1347	0,005415	248738	621	0,005581
0,000935	0,000701	0,000203	segdup	133	0,000592	224842	93	0,000935
8,88E-06	0	0		1	4E-06	249800	1	8,87E-06
0,006077	0,005236	9,85E-05		930	0,003804	244496	670	0,006077
2,79E-05	0	0		3	1,25E-05	239346	3	2,78E-05
0,001126	0,001305	0,000948		199	0,000792	251338	128	0,001126
2,65E-05	0	0	segdup	3	1,2E-05	249280	3	2,65E-05
0,005172	0,005946	0,003236		1005	0,004031	249324	585	0,005172
2,68E-05	0	0		3	1,2E-05	249646	3	2,68E-05
0	0	0		1	4,02E-06	248468	1	2,9E-05
0,004438	0,003114	0,000752		670	0,002676	250362	502	0,004438
3,53E-05	0	3,27E-05		5	1,99E-05	250996	4	3,53E-05
0,003506	0,002913	0,000826		527	0,002191	240480	384	0,003506
9,15E-05	0	3,93E-05		20	9,94E-05	201206	11	0,000884
0,002879	0,002058	0,001261		341	0,00182	187366	229	0,002879
0,003895	0,002282	0,001176		584	0,002323	251410	443	0,003895
1,76E-05	0	0		19	7,56E-05	251370	15	0,000816

0,001309	0,000826	0		160	0,000642	249280	148	0,001309
0,01096	0,009123	0,005521		2205	0,008771	251402	1246	0,010957
0,009731	0,006686	0,002221		1439	0,005727	251256	1105	0,009731
0,01496	0,01051	0,003398		2240	0,008951	250248	1690	0,014962
0,006686	0,00462	0,000654		1057	0,004239	249328	756	0,006686
2,64E-05	0,000163	0		32	0,000127	251474	21	0,000607
2,64E-05	0	3,27E-05		8	3,18E-05	251368	4	0,000217
0,01331	0,008352	0,002975		2061	0,008218	250778	1508	0,013308
0,00352	0,002932	0,000355		184	0,001859	98962	124	0,00352
6,15E-05	0	0,001013		43	0,000171	251482	31	0,001013
0,003692	0,003094	0,000131		631	0,002509	251468	420	0,003692
0,000134	0,00045	0,000284	segdup&d	32	0,000163	196800	3	0,000295
0,01818	0,007869	0,001579	segdup&d	949	0,008892	106720	757	0,018176
9,78E-05	0	0,000105	segdup&d	10	7,59E-05	131808	2	0,000219
0,000133	0,000298	5,22E-05	segdup&d	28	0,000234	119658	16	0,004278
0,007567	0,006018	0,006643	segdup&d	732	0,005444	134460	388	0,007567
0,03906	0,04776	0,03177	segdup&d	877	0,030008	29226	440	0,039063
0,002383	0,001252	0,001065	segdup&d	168	0,001385	121298	106	0,002383
0,000194	0	0		24	9,56E-05	251072	22	0,000194
0,002258	0,001479	0,000196		291	0,001172	248358	255	0,002258
0,000994	0,001305	0,0016		235	0,000935	251342	49	0,0016
0,0114	0,01341	0,001503		2284	0,009126	250276	1286	0,011404
0,001373	0,001793	0,007186		405	0,001611	251328	220	0,007186
5,48E-05	0	3,56E-05		7	2,93E-05	238950	6	5,48E-05
0,003327	0,003597	0,01245		878	0,003499	250928	381	0,012447
0,01219	0,009109	0,0017		1833	0,007364	248910	1375	0,012187
8,87E-06	0	0		1	4,02E-06	248988	1	8,86E-06
0,009326	0,006705	6,63E-05		1276	0,005176	246530	1041	0,009326
0	0,000164	0		9	3,6E-05	249986	6	0,000175
0,005876	0,003588	0,000229	segdup	762	0,003031	251388	668	0,005876
0,000622	0,001187	0,000351		251	0,001036	242366	21	0,000655
0,000422	0,000326	0,00049		88	0,00035	251324	15	0,00049
1,77E-05	0	0		2	7,99E-06	250162	2	1,77E-05
2,81E-05	0	0		4	1,69E-05	236418	1	5,75E-05
0,000524	0,000668	0,000265		92	0,000372	247444	59	0,000524
6,28E-05	0	0		7	2,81E-05	249172	7	6,28E-05
0,000697	0,000895	0,000139		145	0,000624	232206	42	0,003887
0,01047	0,01583	0,004867		2328	0,009264	251302	1190	0,010474
1,76E-05	0	0		30	0,000119	251336	24	0,000694
2,64E-05	0	0		5	1,99E-05	251402	2	5,78E-05
5,31E-05	0	0	segdup	6	2,4E-05	250284	6	5,31E-05
0,003275	0,003592	0,01647		996	0,003964	251256	504	0,016465
1,77E-05	0	0		3	1,21E-05	247994	1	5,52E-05

0,000124	0,000165	0,000131		33	0,000132	249414	9	0,000261
0,000725	0,000165	0		109	0,000437	249358	12	0,000777
0,004406	0,002747	0		914	0,003768	242590	488	0,004406
0,01118	0,009132	0,005782		2013	0,00801	251322	1271	0,011182
0,001953	0,002774	0,000719		414	0,001647	251350	222	0,001953
0,001099	0,000652	0	segdup	139	0,000553	251474	125	0,001099
0,000258	0,000164	0		30	0,00012	249776	29	0,000258
0,000917	0,000195	0		118	0,000544	216836	96	0,000917
0,004221	0,005867	0,008688	segdup	971	0,003862	251434	266	0,008688
5,28E-05	0,000326	0	segdup	16	6,36E-05	251410	8	0,000231
0,000137	0,000338	9,83E-05	segdup	47	0,000193	243564	14	0,001002
0,003451	0,002147	0,000784		476	0,00191	249264	390	0,003451
0,009927	0,00996	0,009715	segdup	2067	0,008356	247372	1112	0,009927
0,01427	0,01026	0,00405		2281	0,00907	251490	1624	0,014274
0,001513	0,001141	0,000751		229	0,000911	251310	172	0,001513
0,006846	0,002396	0,000593		325	0,003353	96930	247	0,006846
0,005648	0,005404	0,000303		797	0,003532	225634	579	0,005648
0,007074	0,008661	0,001346		1268	0,005132	247078	284	0,008285
0,01311	0,01189	0,002515		2297	0,009133	251492	1492	0,013114
8,82E-06	0	0		1	3,98E-06	250974	1	8,82E-06
0,007607	0,00783	0,003691		1354	0,005386	251394	865	0,007607
0,005582	0,0123	0,00981						
0,01125	0,01052	0,0245	segdup	1377	0,010735	128276	547	0,024503
0,6755	0,7541	0,7779	segdup	128135	0,742545	172562	11494	0,843782
0,6624	0,7321	0,7656	segdup	124844	0,724599	172294	11426	0,842004
0,6722	0,7486	0,7732	segdup	127799	0,73915	172900	11495	0,841139
0,07506	0,09285	0,08592	segdup	21673	0,098375	220310	2473	0,178944
0,685	0,7578	0,7759	segdup	149105	0,742666	200770	13070	0,832802
0,6806	0,7456	0,7644	segdup	156052	0,731738	213262	13453	0,824326
0,4121	0,5796	0,5743	segdup	71634	0,518186	138240	7792	0,6751
0,4499	0,5945	0,6122	segdup	78481	0,541592	144908	8272	0,693611
0,05449	0,09113	0,08337	segdup	17576	0,082767	212354	1727	0,135536
0,5867	0,7047	0,7523	segdup	107105	0,673218	159094	10130	0,776483
0,6674	0,7261	0,765	segdup	126292	0,722701	174750	11827	0,833357
0,2715	0,2944	0,2599	segdup	76513	0,327884	233354	9728	0,577672
0,6793	0,7321	0,7558	segdup	173256	0,723359	239516	14406	0,81124
0,3294	0,3406	0,3379						
0,4018	0,462	0,466	segdup	80352	0,423293	189826	6751	0,483458
0,2383	0,2581	0,1851	segdup	57268	0,251387	227808	5094	0,357273
0,03648	0,06198	0,04205	segdup	11367	0,053484	212532	1300	0,103983
0,03414	0,0644	0,05278	segdup	11149	0,054567	204318	1289	0,107902
0,2096	0,2147	0,2951						
0,001077	0,001958	0,007056		751	0,002994	250868	209	0,012857
0,01098	0,008516	0,01022		2025	0,008153	248362	1246	0,010977
0,00016	0,000999	9,94E-05		447	0,001804	247826	344	0,022245
0,00015	0,000814	6,53E-05		349	0,001388	251468	265	0,016302

0,0003	0,00098	0,000882		580	0,002311	251026	463	0,028499
0,000221	0,000357	0		27	0,000119	226026	22	0,000221
4,4E-05	0	0		8	3,18E-05	251398	2	0,000123
0,004802	0,007853	0,02414		2038	0,008136	250502	735	0,024141
0,00147	0,001141	0,001797		267	0,001062	251330	55	0,001797
0,000555	0,000326	0		75	0,000299	251096	63	0,000555
8,79E-06	0	3,27E-05		2	7,95E-06	251492	1	3,27E-05
0,001394	0,001492	0,000589		228	0,000918	248360	157	0,001394
0,000284	0,000691	0,00022		28	0,000187	149830	17	0,000284
0,006536	0,006007	0,002992		1051	0,004418	237880	711	0,006536
0,002658	0,001715	0,002511		431	0,001795	240070	286	0,002658
8,69E-05	0,000175	0		56	0,00024	233302	9	8,69E-05
0,00197	0,004402	0,001241		546	0,002172	251410	156	0,004511
0,0141	0,009927	0,004315		2175	0,008733	249044	1591	0,014099
0,01098	0,00971	0,04647		3377	0,013577	248738	1416	0,046472
8,91E-06	0	6,72E-05		6	2,42E-05	248210	2	6,71E-05
8,81E-06	0	0		2	7,96E-06	251148	1	2,89E-05
0,01073	0,009778	0,001568		1716	0,006826	251406	1220	0,010732
0,000452	0,000957	0,000433	segdup	73	0,000642	113636	20	0,004398
0,01334	0,0124	0,003145		2335	0,009388	248722	1504	0,013341
0,01275	0,009418	0,000464		2024	0,008243	245536	1392	0,012748
1,76E-05	0	0		4	1,59E-05	251288	2	5,78E-05
0,006483	0,01177	0,008222	segdup	1475	0,006206	237680	249	0,008222
0,000283	0,00099	0,000229		97	0,000389	249572	32	0,000282
0,00223	0,003614	0,01217		840	0,003359	250048	371	0,012171
0,00197	0,002791	0,00892		580	0,002316	250386	272	0,00892
0,000299	0,000326	3,27E-05	segdup	46	0,000183	251304	34	0,000299
1,52E-05	0	0		2	1,21E-05	165434	1	0,000105
2,83E-05	0	0	decoy	3	1,31E-05	229574	3	2,83E-05
0,000255	0	0		30	0,000119	251084	29	0,000255
0,007561	0,007516	0,0281		2092	0,008331	251102	860	0,028099
6,16E-05	0	0		10	3,98E-05	251400	3	0,000185
0	0	0		2	2,09E-05	95532	2	0,000115
1,76E-05	0	0		10	3,98E-05	251482	4	0,000246
0,01416	0,01556	0,006962		2618	0,011027	237412	1504	0,014161
0,000107	0	0		17	6,88E-05	247190	12	0,000107
0,005932	0,007038	0,0019		1035	0,004232	244572	650	0,005932
8,84E-06	0	0	segdup	3	1,2E-05	250790	2	5,78E-05
0,00049	0,000836	0,000562		551	0,002229	247240	391	0,025334
0,001693	0,000725	0		72	0,001664	43274	1	0,001748
0,01261	0,009938	0,001502		2198	0,008741	251458	1434	0,012608
0,01126	0,008582	0,001989		1353	0,007322	184778	846	0,011263
0,00457	0,005225	0,01017		1196	0,004762	251134	311	0,010168
8,79E-05	0,000163	0		13	5,17E-05	251458	10	8,79E-05
9,65E-05	0	0		5	4,94E-05	101158	5	9,65E-05
9,72E-05	0,000165	0,000132		16	6,4E-05	250188	4	0,000131

0,000132	0	0		24	9,54E-05	251480	8	0,000492
3,03E-05	0	0		1	1,41E-05	70932	1	3,03E-05
0,0158	0,01251	0,003902		2492	0,010142	245712	1753	0,015802
0,000561	0,000961	0,000222		139	0,001015	136992	16	0,000655
0,000168	0,001186	0,000158						
1,2E-05	0	0						
8,92E-06	0	0		1	4,03E-06	248322	1	8,92E-06
0,1037	0,2012	0,1897	segdup	25449	0,198349	128304	3161	0,597543
0,01406	0,0194	0,003465		2916	0,011738	248416	1562	0,014056
0,00197	0,000815	0,000948		325	0,001293	251402	224	0,00197
0,00029	0	0,000131		44	0,000175	251372	33	0,00029
0,000115	0,000165	3,27E-05		23	9,25E-05	248700	4	0,000223
0,002918	0,003294	0,009804		726	0,002915	249060	300	0,009804
0,009823	0,01199	0,000853		1745	0,007066	246954	1095	0,009823
0,01056	0,009055	0,002989	segdup	1412	0,007698	183421	865	0,010562
4,89E-05	0	0		4	2,18E-05	183348	4	4,89E-05
1,22E-05	0	0		1	5,45E-06	183371	1	1,22E-05
0,008616	0,005125	0,01621	segdup	627	0,005843	107315	171	0,016205
0,002291	0,003791	0,003019	segdup	261	0,002775	94061	36	0,005521
0,000261	0	0,000398	segdup	28	0,000296	94455	7	0,00047
0,000122	0,000431	0,000413	segdup	22	0,000241	91192	7	0,00048
0,002346	0,000486	0,001898	segdup	203	0,001273	159435	161	0,002346
0	0	0,001271		24	0,000132	181158	24	0,001271
0,005651	0,004655	0,00021		654	0,00358	182659	460	0,005651
2,51E-05	0	0		3	1,67E-05	179558	1	3,69E-05
0,00023	0,000327	0,000948		58	0,000232	250250	29	0,000947
0,004355	0,00348	0,003003		809	0,003586	225606	467	0,004355
0,002159	0,000816	3,27E-05		320	0,001275	251002	245	0,002159
0,003187	0,002812	0,000132		785	0,003177	247126	354	0,003187
3,52E-05	0,000815	0,00049		38	0,000151	251432	15	0,00049
0,002436	0,003216	0,000828		616	0,002534	243100	96	0,002821
0,001519	0,000982	0,000753		279	0,001113	250568	172	0,001519
8,83E-06	0	0		2	7,97E-06	250856	1	5,44E-05
0,005539	0,007244	0,005322	segdup	985	0,004592	214500	546	0,005539
8,8E-06	0	0		3	1,19E-05	251374	1	6,15E-05
0,003034	0,002142	0,00204		597	0,002392	249622	342	0,003034
4,42E-05	0,000163	0		11	4,39E-05	250800	3	0,000185
0,05592	0,1018	0,08603	segdup	15906	0,092049	172800	2645	0,244907
0,003144	0,006538	0,00147		608	0,002424	250778	130	0,003761
0,008996	0,005804	0,002651		1342	0,005578	240602	966	0,008996

0	0	0						
3,52E-05	0	0						
0,002026	0,001474	0						
9,75E-06	0	0						
0,003652	0,004731	0,003204						
7,03E-05	0,000326	0		259	0,00103	251430	224	0,013798
0,00268	0,004454	0,001439	segdup	1554	0,006266	248012	1067	0,030998
0,001926	0,001143	0,000458		303	0,001206	251302	219	0,001926
8,82E-05	0	0	segdup	11	4,38E-05	251082	10	8,82E-05

exomes_PO	mes_POPM	exomes_co	exomes_co	exomes_co	mes_cont	_exomes_A	AD_genome	AD_genom
113282	7	767	0,007061	108618	5	8		891
34592	0	7	6,4E-05	109408	0	0		4
16256	0	5	4,57E-05	109406	0	0		5
								71
34592	0	4	3,66E-05	109406	0	0		8
22774	31	910	0,014152	64300	21	44		692
19688	3	171	0,00302	56630	3	3		105
113624	0	29	0,000265	109394	0	0		41
95158	218	400	0,004068	98320	116	264		509
34580	0	174	0,001591	109398	1	1		234
18392	0	0	0	109406	0	0		2
52128	0	7	0,000115	60724	0	0		23
113346	3	153	0,0014	109298	2	3		222
30616	1	183	0,001673	109406	0	4		170
113700	0	40	0,000366	109384	0	1		25

34150	0	2	1,84E-05	108944	0	0	2
113634	5	600	0,00549	109296	4	11	855
113014	9	612	0,005649	108330	7	14	838
6972	0	4	6,19E-05	64606	0	0	20
30610	30	898	0,008209	109388	13	34	1046
113478	0	4	3,66E-05	109372	0	0	5
113570	0	0	0	109402	0	0	3
7570	2136	10555	0,213803	49368	4267	10191	
113672	0	16	0,000146	109404	0	0	25
14392	1171	3557	0,036881	96446	1701	3862	
18394	3	768	0,007021	109388	13	23	579
113754	6	436	0,003985	109404	5	6	642
113634	0	48	0,000439	109380	0	1	61
112982	1	179	0,001638	109282	0	1	287
							49
34534	3	398	0,003645	109194	5	10	610
113630	0	1	9,14E-06	109402	0	0	
113124	10	705	0,006448	109336	9	18	901
30438	8	536	0,004919	108976	3	11	601
8644	0	12	0,000256	46876	0	0	35
113478	0	1	9,15E-06	109292	0	0	
30564	3	371	0,003398	109194	2	9	468
113496	0	0	0	109390	0	0	1
30600	2	86	0,000793	108386	0	2	45
30582	0	12	0,000111	108240	0	0	7
113660	4	755	0,006906	109320	6	8	1196
34588	0	0	0	109404	0	0	
113642	0	1	9,15E-06	109276	0	0	
33796	1	232	0,002238	103656	0	2	364
113738	0	2	1,83E-05	109308	0	0	
17978	96	1735	0,015979	108580	53	107	1329
111782	0	1	9,19E-06	108788	0	0	1
							288
18360	0	97	0,004207	23056	0	0	752
34180	0	63	0,000588	107108	0	0	74
103610	0	2	1,96E-05	102028	0	0	6
113628	0	37	0,000338	109386	0	0	64
15482	0	97	0,000893	108616	0	0	398
18336	2	902	0,008249	109344	5	16	1165
111800	4	427	0,003963	107756	2	4	658
113632	4	419	0,003832	109336	2	5	431

17972	0	1	9,22E-06	108468	0	0	3
113692	9	607	0,005548	109408	3	10	774
16222	0	2	1,84E-05	108652	0	0	14
16122	0	70	0,00064	109294	0	0	184
113392	3	345	0,003158	109240	2	5	362
34546	0	8	7,32E-05	109342	0	0	6
24318	0	68	0,001073	63400	0	0	180
29146	8	653	0,006234	104750	8	12	653
81680	1	314	0,003977	78952	0	1	691
25836	3	258	0,008085	31912	2	8	1255
30314	0	307	0,002962	103652	2	4	320
30576	1268	19457	0,178662	108904	2061	4499	
24556	9658	50858	0,800057	63568	20640	47117	
34526	0	8	7,37E-05	108530	0	0	5
30616	0	15	0,000137	109402	0	0	1
113596	18	1018	0,009309	109356	10	27	1267
113354	2	300	0,002743	109372	1	4	431
30602	3	408	0,003756	108636	2	5	551
113294	0	2	1,83E-05	109348	0	0	2
18380	0	24	0,00022	109326	0	0	27
							1
102800	0	78	0,000791	98624	0	2	79
113716	6	680	0,006217	109376	4	9	1016
18394	0	1	9,14E-06	109396	0	0	
18394	0	4	3,66E-05	109408	0	0	6
29870	0	1	9,33E-06	107238	0	0	2
0	0	0	0	109396	0	0	1
30580	0	2	1,84E-05	108406	0	0	1
22774	2	477	0,007375	64674	1	5	907
112794	6	891	0,008225	108326	7	13	1145
34592	0	110	0,001005	109408	0	0	242
55274	0	4	7,31E-05	54698	0	0	14
9896	0	3	3,93E-05	76286	0	0	16
16256	0	41	0,000375	109408	0	0	244
113764	0	97	0,000887	109408	0	1	155
112636	0	2	1,84E-05	108768	0	0	1
30572	10	976	0,008931	109288	11	25	1104
							35
28330	0	20	0,000195	102636	1	1	172
28994	0	47	0,000443	106112	0	0	42
113536	0	125	0,001143	109408	0	0	162
							1

108696	0	22	0,000211	104398	0	0	42
6392	1	181	0,022631	7998	1	2	
113766	3	474	0,004332	109406	4	8	677
30616	0	275	0,002514	109402	1	1	342
113614	0	3	2,75E-05	109166	0	0	3
27894	7	623	0,006333	98380	5	11	745
103334	0	1	1,02E-05	98276	0	0	5
25038	0	0	0	100576	0	0	
							1
34510	0	41	0,000375	109308	0	0	43
112622	15	951	0,008785	108256	9	25	1427
33040	0	18	0,000173	104334	0	0	71
30358	44	1201	0,011101	108190	22	54	829
112516	0	0	0	108726	0	0	1
60466	0	35	0,000542	64522	0	0	157
110192	0	10	9,22E-05	108450	0	0	16
111264	0	2	1,83E-05	109398	0	0	5
34512	31	1082	0,0099	109290	18	41	1173
108138	0	79	0,000782	101000	0	0	132
102200	0	45	0,000424	106124	0	0	70
113756	0	46	0,00042	109408	0	0	68
111946	0	1	9,36E-06	106872	0	0	
13352	10	56	0,00057	98254	19	41	874
							228
25402	4	163	0,005495	29666	2	6	1058
16250	0	145	0,001364	106290	0	0	744
16136	0	74	0,000695	106498	0	0	345
16256	0	61	0,000558	109408	0	0	125
113530	1	2	1,83E-05	109326	1	1	
112490	3	456	0,00421	108324	2	3	664
30616	0	22	0,000201	109406	0	0	3
33874	0	5	4,6E-05	108670	0	0	1
113580	3	794	0,007259	109376	5	6	1147
112874	1	29	0,000267	108470	0	1	30
67640	0	250	0,003522	70980	2	3	284
59302	1	287	0,004485	63986	1	1	738
							1223
112982	0	26	0,00024	108526	0	0	66
25794	378	1217	0,016046	75846	468	1289	
20558	126	810	0,010706	75662	314	796	
50880	515	891	0,01425	62528	323	811	47
28100	179	708	0,008795	80502	204	466	
26388	439	1539	0,020195	76206	699	1819	
							19
6574	17	61	0,001295	47120	22	43	224
3328	2	16	0,001095	14618	1	4	134

54126	2	317	0,005938	53382	1	3	834
52858	0	14	0,000264	53010	1	1	20
34564	0	206	0,001883	109380	0	0	323
34428	2	246	0,002332	105486	0	3	423
34572	0	4	3,66E-05	109356	0	0	8
90732	1	194	0,002275	85266	0	1	165
104340	2	290	0,002947	98402	0	4	503
113696	0	2	1,83E-05	109388	0	0	2
							755
113650	6	629	0,00575	109398	1	6	908
29988	0	127	0,003894	32618	0	0	442
30192	0	0	0	106968	0	0	9
112906	0	4	3,66E-05	109322	0	0	16
0	0	0	0	77616	0	0	
113600	0	276	0,002524	109364	1	3	426
32410	0	190	0,001938	98042	0	0	261
110984	0	18	0,000165	109060	0	0	22
30616	0	17	0,000155	109408	0	0	16
11930	0	2	2,24E-05	89452	0	0	4
113568	4	563	0,005149	109350	2	6	743
30602	0	3	2,76E-05	108628	0	0	2
30372	1	193	0,001773	108862	1	3	165
112648	0	13	0,000119	108864	0	0	9
113624	2	334	0,003054	109372	1	3	509
30616	0	133	0,001216	109408	0	1	70
113524	4	665	0,006078	109402	5	11	874
111364	0	1	9,37E-06	106756	0	0	5
2062	0	327	0,07118	4594	1	4	61
112448	0	50	0,000461	108530	0	0	68
111764	0	0	0	107646	0	0	
18382	0	13	0,000119	109300	0	0	18
113746	3	571	0,005219	109408	1	6	697
15246	0	14	0,000132	105842	0	0	41
7994	0	1	1,56E-05	64250	0	0	1
113144	0	11	0,000101	109310	0	0	9
18394	0	6	5,48E-05	109408	0	0	2
112356	0	0	0	109338	0	0	3
30060	0	1	9,55E-06	104664	0	0	3
22688	0	2	3,74E-05	53474	0	0	2
109962	0	2	1,96E-05	102298	0	0	2
111706	0	1	9,25E-06	108154	0	0	
113720	1	181	0,001655	109386	0	2	257
113020	0	5	4,59E-05	108824	0	0	6
30616	5	173	0,001581	109408	1	5	24
113596	0	0	0	109378	0	0	
16256	6	196	0,001791	109408	2	6	915

113104	0	0	0	108552	0	0	1
67352	0	128	0,001781	71884	1	2	260
113704	0	1	9,14E-06	109370	0	0	9
28572	0	2	1,97E-05	101638	0	0	1
12658	4112	11169	0,277188	40294	5117	13132	
55746	5	540	0,00864	62500	5	10	1231
30600	0	4	3,7E-05	107966	0	0	7
108164	0	1	9,38E-06	106606	0	0	5
113260	1	191	0,001758	108634	0	3	331
30602	28	1090	0,010034	108636	21	37	599
112372	4	513	0,004701	109128	3	8	644
24464	0	23	0,000433	53076	0	0	40
18148	0	2	1,95E-05	102448	0	0	3
10382	0	15	0,000572	26216	0	0	77
113398	2	129	0,00118	109340	0	2	205
113680	3	248	0,002267	109374	3	3	299
113022	0	1	9,21E-06	108526	0	0	2
112170	1	221	0,002035	108608	0	1	314
104128	0	14	0,000136	102756	0	0	18
108924	14	1065	0,009906	107506	17	27	1416
7526	0	4	6,42E-05	62264	0	0	3
6262	0	4	4,38E-05	91352	0	0	19
3946	8	21	0,000258	81478	3	10	216
111972	6	784	0,00719	109036	6	11	1065
104378	1	237	0,002249	105394	1	2	334
110882	11	782	0,007201	108590	8	18	1059
30568	0	37	0,000342	108234	0	0	32
113416	3	345	0,003153	109404	2	3	475
							2
112154	9	961	0,008895	108038	5	14	1344
112974	0	139	0,001274	109100	2	6	219
34592	0	1	9,14E-06	109408	0	0	2
111620	1	105	0,000972	108010	0	1	162
18364	0	4	3,66E-05	109148	0	0	1
30610	2	667	0,0061	109348	2	5	702
81342	0	8	0,000103	77468	0	0	
							1048
113732	0	185	0,001691	109398	0	0	315
59616	0	1	1,56E-05	64208	0	0	4
52718	5	468	0,008558	54686	3	9	1295
432	0	2	0,013889	144	0	0	611
30612	1	189	0,001729	109318	0	1	267

26710	3	546	0,007933	68826	4	13	943
14988	6	300	0,002751	109064	4	9	
15482	2	364	0,003351	108620	3	6	1034
15784	0	61	0,000558	109382	0	0	269
60344	0	15	0,000234	64232	1	1	20
30600	43	1232	0,011353	108520	23	52	773
110558	0	167	0,001595	104708	0	0	182
30616	5	128	0,00117	109402	4	5	100
30592	1	102	0,000934	109230	0	2	97
18380	0	2	1,83E-05	109398	0	0	
30610	16	1075	0,009868	108938	6	28	1131
113588	0	0	0	109350	0	0	
31718	0	1	1,04E-05	95742	0	0	1
112212	0	2	1,84E-05	108436	0	0	
30596	0	48	0,000439	109358	1	1	33
30600	0	44	0,000405	108588	0	0	55
28290	2	979	0,009162	106854	18	32	1148
61578	2	435	0,006642	65490	3	4	1033
106412	46	249	0,002384	104442	32	79	
							1
113732	0	0	0	109238	0	0	
56	0	243	0,018451	13170	1	1	
3670	2	22	0,001463	15042	3	5	
3564	2	84	0,005885	14274	8	19	
110994	2	602	0,005527	108922	3	9	900
30614	9	912	0,008336	109408	12	20	910
112774	12	703	0,006479	108508	5	13	1038
113486	1	258	0,00236	109338	0	1	331
113518	0	1	9,14E-06	109382	0	0	3
113550	2	76	0,000695	109364	1	2	143
113708	0	292	0,002669	109404	0	0	435
34298	0	1	9,26E-06	107982	0	0	2
54018	0	3	5,62E-05	53358	0	0	3
30616	23	1385	0,012659	109408	26	42	1058
112578	14	931	0,0086	108256	14	21	1353
30616	0	2	1,83E-05	109408	0	0	
31900	0	6	6,4E-05	93808	0	0	10
52764	3	428	0,006766	63260	0	3	950
112816	0	620	0,005668	109382	6	8	596
18340	4	702	0,006424	109274	5	14	390
16256	1	460	0,004205	109400	3	12	1297
34494	0	57	0,000532	107090	0	0	124
16256	0	6	5,48E-05	109406	0	0	16
15222	0	12	0,000112	107598	0	0	48
16216	1	165	0,001509	109366	1	2	506
16256	0	59	0,000539	109404	0	0	267
16256	0	20	0,000183	109408	0	0	89

87158	1	310	0,003421	90626	1	2	603
23506	2	411	0,005899	69670	6	11	1202
30608	4	194	0,001774	109378	2	4	52
							1
60542	0	6	9,34E-05	64236	0	0	24
16240	4146	69613	0,636514	109366	22318	52150	14
34588	124	1246	0,011389	109406	62	124	400
109834	0	0	0	102902	0	0	2
32614	1589	22611	0,230593	98056	2873	6444	
113660	37618	78632	0,719151	109340	29421	68303	
100400	33201	69047	0,720907	95778	25657	60512	
15008	872	23515	0,266634	88192	3274	7492	
112342	4124	22217	0,203531	109158	2739	6477	
1382	0	159	0,015796	10066	4	7	31
112584	0	92	0,000848	108448	0	1	134
29774	13869	79012	0,927393	85198	36685	86047	63
113748	9	633	0,005787	109376	5	14	695
110824	0	20	0,000184	108624	0	0	27
113754	1	393	0,003592	109408	0	3	619
113014	10	982	0,009048	108528	7	13	1400
							731
113718	14	890	0,008135	109408	10	18	1179
18392	5	572	0,00523	109362	6	14	449
16246	0	1	9,14E-06	109374	0	0	6
35822	7	322	0,00863	37310	5	14	1015
111268	3	506	0,004633	109206	3	9	762
99438	1	48	0,000492	97582	1	2	230
112680	0	0	0	109030	0	0	
110246	2	396	0,003704	106898	0	2	611
107736	0	1	9,53E-06	104952	0	0	2
113694	0	84	0,000768	109364	0	0	103
113032	0	0	0	108528	0	0	4
113108	1	368	0,003388	108606	0	1	508
111968	0	1	9,14E-06	109378	0	0	4
34506	0	0	0	108622	0	0	
113122	0	293	0,002685	109110	0	0	344
113464	0	2	1,83E-05	109292	0	0	2
109530	0	231	0,002191	105454	0	0	244
12450	0	8	9,21E-05	86890	0	0	49
79534	1	128	0,00158	81028	0	2	263
113726	1	230	0,002103	109388	1	2	251
							156
18390	0	8	7,31E-05	109404	0	0	2

							3
113076	0	67	0,000617	108614	0	0	92
113722	11	934	0,008539	109376	12	20	1243
113558	7	547	0,005	109396	2	10	881
112952	16	875	0,007998	109398	8	21	1372
113070	4	438	0,004034	108576	2	4	604
34590	0	11	0,000101	109406	0	0	8
18392	0	4	3,66E-05	109402	0	0	3
113316	12	868	0,007941	109304	7	15	1068
35228	0	74	0,001805	41000	0	0	383
30616	0	21	0,000192	109406	0	0	21
113758	0	257	0,002349	109398	0	0	383
10162	0	12	0,000138	86802	0	0	120
41648	301	357	0,008	44626	144	378	564
9132	0	4	7,19E-05	55630	0	0	
3740	2	11	0,000221	49774	1	2	
							190
51274	82	282	0,005008	56306	68	172	
11264	93	341	0,023379	14586	75	195	74
44476	0	72	0,001399	51450	0	0	125
113570	0	12	0,00011	109310	0	0	15
112946	0	109	0,001018	107118	1	2	166
30616	1	88	0,000804	109398	0	1	84
112770	9	872	0,007972	109384	10	24	1188
30614	4	178	0,001627	109396	1	4	120
109414	0	2	1,91E-05	104910	0	0	3
30610	1	397	0,00363	109376	1	2	339
112824	13	716	0,006601	108462	5	16	1150
112804	0	0	0	108506	0	0	1
111620	8	504	0,004675	107796	6	9	830
34244	0	4	3,67E-05	108914	0	0	
113688	3	304	0,002779	109404	2	3	502
							1
32056	0	77	0,000725	106246	0	0	97
30616	0	36	0,000329	109392	0	0	30
113210	0	0	0	108936	0	0	
17396	0	0	0	103076	0	0	
112594	0	42	0,00039	107768	0	0	44
111470	0	3	2,74E-05	109404	0	0	4
10804	0	37	0,000364	101780	0	0	360
113610	8	1038	0,009492	109350	9	19	1289
34588	0	14	0,000128	109398	0	0	13
34584	0	0	0	109392	0	0	4
113000	0	2	1,83E-05	109084	0	0	1
30610	12	441	0,004031	109400	8	14	365
							1
18112	0	1	9,27E-06	107878	0	0	1

34522	0	16	0,000147	108616	0	0	18
15440	0	32	0,000295	108620	0	0	74
110764	0	481	0,004537	106016	1	1	568
113668	8	849	0,007762	109384	9	17	1180
113648	1	154	0,001408	109398	1	4	205
113760	0	55	0,000503	109400	0	0	101
112590	0	7	6,42E-05	109032	0	0	12
104688	0	44	0,000459	95848	0	0	67
30616	4	398	0,003638	109408	4	8	395
34592	0	8	7,31E-05	109406	0	0	8
13974	0	20	0,000187	106836	0	0	113
113014	1	168	0,001548	108528	0	1	314
112018	6	895	0,008299	107838	7	13	1129
113770	17	895	0,008181	109406	8	21	1299
113666	1	79	0,000723	109306	0	1	127
36080	1	135	0,003381	39924	0	1	613
102520	5	294	0,00298	98642	0	5	500
34278	1	495	0,0046	107598	3	10	674
113768	17	878	0,008025	109408	10	24	1228
113440	0	0	0	109342	0	0	1
113706	1	549	0,005019	109390	1	2	828
							177
22324	6	572	0,010853	52702	6	11	1102
13622	5235	55249	0,741299	74530	23693	54811	6
13570	5209	53841	0,723669	74400	22793	52701	5
13666	5209	55114	0,737429	74738	23590	54601	5
13820	311	9555	0,099858	95686	902	1988	
15694	5693	64683	0,739201	87504	26150	60512	6
16320	5779	67648	0,728871	92812	26264	60902	
11542	3469	32161	0,517124	62192	13420	30014	
11926	3700	34685	0,538102	64458	14365	32537	
12742	232	7949	0,085777	92670	995	2295	
13046	4497	46630	0,665534	70064	19372	44844	158
14192	5166	55310	0,722232	76582	21948	50246	
16840	3041	34670	0,341099	101642	7374	15712	113
17758	5919	75780	0,722347	104908	28001	64149	62
							191
13964	1352	35293	0,418263	84380	5490	12623	343
14258	356	24866	0,246291	100962	1292	3241	117
12502	145	5135	0,055149	93112	548	1171	452
11946	151	4981	0,055728	89380	546	1154	594
16256	0	298	0,002725	109344	0	1	657
113514	8	799	0,007498	106558	8	14	1060
15464	1	209	0,001936	107970	0	1	983
16256	0	160	0,001462	109408	0	0	729

16246	2	256	0,002341	109356	1	2	1217
99416	1	14	0,000143	97750	0	1	10
16252	0	5	4,57E-05	109368	0	0	16
30446	14	990	0,009091	108898	12	28	684
30612	0	118	0,001079	109398	0	0	122
113424	0	36	0,000329	109390	0	0	67
30616	0	2	1,83E-05	109408	0	0	
112596	0	87	0,000805	108034	0	0	151
59828	0	11	0,000189	58342	0	0	27
108790	5	434	0,004179	103852	4	7	712
107592	3	169	0,001583	106738	0	4	187
103548	0	18	0,000175	102940	0	0	25
34580	0	224	0,002048	109396	0	1	221
112846	15	825	0,007607	108452	12	21	1337
30470	36	1578	0,014631	107856	27	49	1348
29786	0	2	1,85E-05	107988	0	0	1
34570	0	1	9,14E-06	109360	0	0	2
113684	6	690	0,006307	109408	1	9	939
4548	0	26	0,000593	43834	0	0	
112738	8	945	0,008727	108280	11	18	1205
109196	8	821	0,007551	108732	4	12	1117
34584	0	3	2,74E-05	109404	0	0	11
30284	3	588	0,005898	99690	7	16	665
113282	0	26	0,000239	108636	0	0	43
30482	5	335	0,003072	109046	2	6	268
30494	6	267	0,00245	108984	3	6	186
113668	0	21	0,000192	109368	0	0	20
9484	0	1	1,43E-05	69916	0	0	4
106008	0	3	3,08E-05	97270	0	0	4
113540	0	10	9,15E-05	109278	0	0	16
30606	23	888	0,008119	109374	9	28	854
16256	0	4	3,66E-05	109380	0	0	7
17332	0	2	4,95E-05	40438	0	0	4
16256	0	4	3,66E-05	109404	0	0	19
106208	10	1076	0,010282	104644	10	23	1207
111764	0	6	5,57E-05	107750	0	0	24
109578	3	398	0,003676	108256	5	8	558
34586	0	3	2,74E-05	109398	0	0	
15434	1	254	0,002357	107772	1	3	1175
572	0	15	0,000912	16442	0	1	246
113734	8	885	0,008089	109408	4	11	1266
75114	5	541	0,006779	79800	5	8	1016
30586	4	470	0,004299	109326	5	7	521
113762	0	2	1,83E-05	109402	0	0	6
51794	0	2	3,9E-05	51332	0	0	3
30428	0	6	5,51E-05	108886	0	0	4

16256	0	7	6,4E-05	109408	0	0	29
33004	0	0	0	29750	0	0	5
110932	14	1033	0,009584	107782	4	16	1377
24446	0	34	0,000641	53008	0	2	105
							19
112084	0	0	0	108424	0	0	
5290	1387	10602	0,18842	56268	3433	8128	44282
111130	19	1083	0,009916	109212	14	45	1346
113714	2	122	0,001116	109362	0	2	148
113684	0	18	0,000165	109398	0	0	15
17964	0	14	0,000129	108462	0	0	12
30600	4	305	0,002796	109082	1	5	300
111472	7	703	0,00651	107986	7	13	1049
81895	6	590	0,007345	80328	2	7	748
81819	0	0	0	80327	0	0	5
81830	0	0	0	80343	0	0	
10552	2	269	0,005595	48081	1	4	490
6520	2	118	0,002852	41376	11	27	
14888	0	15	0,000361	41595	0	0	
14574	0	9	0,000225	39987	0	0	76
68637	0	87	0,001253	69447	1	1	145
18881	0	10	0,000125	80035	0	0	
81404	1	266	0,003317	80186	1	2	382
27111	0	2	2,53E-05	79179	0	0	5
30608	0	30	0,000275	109206	1	1	27
107244	4	344	0,003439	100040	1	5	447
113470	1	121	0,001108	109230	0	1	229
111062	2	406	0,003748	108334	0	4	478
30614	0	14	0,000128	109396	0	0	7
34030	0	206	0,001958	105210	2	4	269
113212	1	92	0,000842	109220	0	1	158
18392	0	1	9,15E-06	109286	0	0	1
98580	1	413	0,004557	90630	3	7	620
16254	0	3	2,74E-05	109392	0	0	5
112708	5	286	0,002627	108878	7	10	732
16256	0	5	4,57E-05	109396	0	0	8
10800	548	7301	0,09507	76796	938	2068	
34568	1	226	0,002066	109366	0	4	270
107384	3	507	0,004804	105534	1	3	827

								1295
								3
								1329
								1405
16234	2	118	0,001079	109402	1	2		665
34422	18	672	0,006166	108988	8	19		566
113694	0	119	0,001088	109352	0	0		181
113402	0	4	3,66E-05	109394	0	0		8

AD_genom	AD_genom	_genomes_	ada_score	rf_score	pliceRegio	earestExon	learestGen	AI_pred_D
						ENSE0000(	-	-39
						ENSE0000(	-	
						ENSE0000:	-	2
						ENSE0000:	-	-4
						ENSE0000:	-	6
						ENSE0000:	-	
						ENSE0000:	-	
						ENSE0000:	-	18
						ENSE0000:	-	-10
						ENSE0000:	-	
						ENSE0000:	-	
						ENSE0000:	-	
						ENSE0000:	-	-1
						ENSE0000:	-	
						ENSE0000:	-	23
						ENSE0000:	-	
						ENSE0000:	-	
						ENSE0000:	-	
						ENSE0000(	-	-1
						ENSE0000:	-	
						ENSE0000:	-	-27
						ENSE0000:	-	
						ENSE0000(	-	-10
						ENSE0000:	-	
						ENSE0000:	-	
						ENSE0000:	-	
						ENSE0000:	-	
						ENSE0000:	-	
						ENSE0000:	-	
						ENSE0000:	-	
						ENSE0000(	-	
						ENSE0000:	-	
						ENSE0000:	-	
0,00622	143250	1				ENSE0000:	-	-2
2,79E-05	143330	1				ENSE0000:	-	23
3,49E-05	143294	0				ENSE0000:	-	-1
0,000503	141202	1				ENSE0000:	-	6
5,59E-05	143208	0				ENSE0000:	-	5
0,00483	143276	10				ENSE0000:	-	-2
0,000733	143278	0				ENSE0000:	-	-49
0,000286	143266	1				ENSE0000:	-	-22
0,004106	123974	126				ENSE0000:	-	22
						ENSE0000:	-	-45
0,001633	143338	0				ENSE0000:	-	-14
1,4E-05	143220	0				ENSE0000:	-	33
0,000161	143268	0				ENSE0000:	-	6
0,00155	143250	0				ENSE0000:	-	-6
0,001187	143250	0				ENSE0000:	-	2
0,000175	143252	0				ENSE0000:	-	36

1,4E-05	143140	0	ENSE00003	-	28
0,005968	143270	3	ENSE00001	-	45
0,005859	143040	3	ENSE00002	-	-20
			ENSE00003	-	3
0,00014	143224	0	ENSE00003	-	40
0,0073	143284	7	ENSE00001	-	22
3,49E-05	143318	0	ENSE00003	-	-38
2,09E-05	143210	0	ENSE00003	-	-6
			ENSE00003	-	
			ENSE00003	-	0
0,000174	143318	0	ENSE00001	-	39
			ENSE00001	-	36
			ENSE00001	-	-7
0,004045	143130	11	ENSE00001	-	-18
			ENSE00003	-	-25
0,004481	143278	2	ENSE00001	-	8
0,000426	143200	0	ENSE00003	-	17
0,002002	143362	0	ENSE00001	-	25
0,000391	125430	0	ENSE00000	-	12
0,00426	143198	2	ENSE00001	-	1
			ENSE00001	-	-38
0,006289	143276	7	ENSE00000	-	31
0,004207	142856	4	ENSE00001	-	-14
0,000727	48170	0	ENSE00003	-	16
			ENSE00003	-	40
0,003266	143316	2	ENSE00003	-	-15
			ENSE00002	-	24
6,98E-06	143270	0	ENSE00002	-	-15
			ENSE00003	-	-7
0,000314	143330	0	ENSE00002	-	-36
4,89E-05	143216	0	ENSE00001	-	14
0,008345	143316	7	ENSE00000	-	-10
			ENSE00003	-	8
			ENSE00003	-	12
0,002541	143234	2	ENSE00003	-	-8
			ENSE00001	-	-3
			ENSE00002	-	19
			ENSE00003	-	-20
0,009275	143290	23	ENSE00002	-	-3
6,98E-06	143360	0	ENSE00001	-	
0,00201	143314	0	ENSE00002	-	
0,005266	142816	3	ENSE00003	-	
0,000517	143042	0	ENSE00001	-	45
4,21E-05	142600	0	ENSE00001	-	1
0,000447	143124	0	ENSE00001	-	-2
			ENSE00003	-	-22
0,002777	143314	2	ENSE00001	-	-3
0,008138	143148	10	ENSE00002	-	-25
0,004596	143170	3	ENSE00001	-	-5
0,003009	143238	1	ENSE00003	-	44

2,09E-05	143260	0	ENSE0000(	-	-22
			ENSE0000(	-	-11
0,005402	143280	1	ENSE00003	-	32
9,77E-05	143272	0	ENSE00002	-	5
0,001283	143374	1	ENSE00003	-	2
0,002527	143242	1	ENSE00001	-	-4
4,19E-05	143294	0	ENSE00003	-	-21
0,001256	143360	0	ENSE00001	-	1
0,004564	143068	9	ENSE00001	-	9
			ENSE00001	-	-42
0,004826	143178	1	ENSE00003	-	44
0,008783	142882	10	ENSE00001	-	-20
0,002233	143322	1	ENSE00003	-	12
			ENSE00003	-	20
			ENSE00001	-	
3,49E-05	143260	0	ENSE0000(	-	-35
6,98E-06	143314	0	ENSE00001	-	-19
			ENSE0000(	-	-50
0,0089	142364	14	ENSE00003	-	-28
0,003009	143218	3	ENSE00003	-	48
			ENSE00003	-	-49
			ENSE00001	-	12
0,003845	143296	5	ENSE00003	-	-49
			ENSE00002	-	
1,4E-05	143162	0	ENSE00001	-	9
0,000189	143096	0	ENSE00003	-	-36
6,98E-06	143208	0	ENSE00001	-	1
0,000551	143280	0	ENSE00002	-	31
0,007111	142868	5	ENSE00001	-	-4
			ENSE00003	-	7
4,19E-05	143286	0	ENSE00003	-	2
1,4E-05	143246	0	ENSE00001	-	31
6,98E-06	143266	0	ENSE00003	-	-15
6,98E-06	143298	0	ENSE00001	-	27
0,00633	143294	4	ENSE00001	-	20
0,007993	143248	4	ENSE00001	-	43
0,001689	143282	0	ENSE00003	-	43
9,77E-05	143252	0	ENSE00003	-	18
0,000112	142754	0	ENSE0000(	-	11
			ENSE00001	-	-1
0,001703	143276	1	ENSE00003	-	1
0,001082	143254	0	ENSE00002	-	-35
6,97E-06	143370	0	ENSE0000(	-	30
0,007704	143310	13	ENSE00001	-	-34
0,001556	22488	0	ENSE0000(	-	15
0,001934	88950	0	ENSE00001	-	36
0,000293	143320	0	ENSE00003	-	-22
0,001131	143270	0	ENSE00001	-	19
			ENSE00001	-	19
6,98E-06	143186	0	ENSE00001	-	4

0,000293	143344	0	ENSE0000:	-	50
			ENSE0000:	-	-11
0,004728	143180	1	ENSE0000:	-	-2
0,002388	143200	0	ENSE0000:	-	-6
2,13E-05	140750	0	ENSE0000:	-	28
0,005228	142512	2	ENSE0000:	-	-2
3,52E-05	142054	0	ENSE0000:	-	8
			ENSE0000:	-	19
6,98E-06	143260	0	ENSE0000:	-	-7
0,0003	143280	0	ENSE0000:	-	-7
0,009961	143264	18	ENSE0000(	-	29
0,000496	143262	1	ENSE0000(	-	2
0,005787	143246	12	ENSE0000(	-	-46
6,98E-06	143310	0	ENSE0000:	-	8
0,001096	143286	0	ENSE0000.	-	-21
0,000112	143280	0	ENSE0000:	-	50
3,49E-05	143224	0	ENSE0000:	-	-44
			ENSE0000:	-	23
0,008187	143272	30	ENSE0000(	-	-33
0,000921	143296	0	ENSE0000:	-	-3
0,000488	143340	2	ENSE0000:	-	5
0,000475	143212	2	ENSE0000:	-	5
			ENSE0000:	-	-46
0,008553	102182	3	ENSE0000.	-	24
0,007282	31312	0	ENSE0000:	-	
0,00824	128398	4	ENSE0000.	-	-1
0,005191	143322	7	ENSE0000:	-	-3
0,00241	143144	3	ENSE0000:	-	-11
0,000873	143240	0	ENSE0000:	-	-2
			ENSE0000:	-	19
0,004635	143248	2	ENSE0000:	-	16
2,09E-05	143250	0	ENSE0000:	-	-30
			ENSE0000:	-	-1
6,98E-06	143220	0	ENSE0000:	-	3
0,008006	143266	5	ENSE0000:	-	32
0,00021	143192	0	ENSE0000:	-	-13
0,001983	143250	4	ENSE0000:	-	-2
0,00515	143312	2	ENSE0000:	-	40
0,008532	143342	7	ENSE0000:	-	23
0,000461	143274	0	ENSE0000.	-	-41
			ENSE0000.	-	18
			ENSE0000.	-	-15
0,000718	65492	7	ENSE0000.	-	-17
			ENSE0000.	-	-9
			ENSE0000.	-	26
0,000176	107718	0	ENSE0000.	-	-24
			ENSE0000:	-	27
0,002036	110022	12	ENSE0000.	-	15
0,001064	125986	0	ENSE0000.	-	-6
			ENSE0000.	-	-2

0,005818	143354	3	ENSE0000:	-	
0,00014	143214	0	ENSE00003	-	-3
0,002254	143270	0	ENSE00003	-	24
0,002951	143356	2	ENSE0000(	-	31
5,58E-05	143282	0	ENSE0000:	-	-16
0,001152	143278	1	ENSE00003	-	12
0,003512	143240	1	ENSE0000(	-	39
1,4E-05	143250	0	ENSE00003	-	-25
0,007763	97260	0	ENSE00002	-	46
0,006336	143306	2	ENSE0000:	-	44
			ENSE0000:	-	20
0,003087	143170	1	ENSE0000:	-	-8
			ENSE0000:	-	-22
6,28E-05	143292	0	ENSE00003	-	30
0,000112	143246	0	ENSE0000:	-	27
			ENSE00002	-	-40
0,002972	143346	2	ENSE0000:	-	1
0,001822	143228	0	ENSE00003	-	50
0,000154	143312	0	ENSE0000:	-	-14
0,000112	143346	0	ENSE0000:	-	-27
2,79E-05	143218	0	ENSE0000(	-	-36
0,005187	143250	2	ENSE0000:	-	22
1,4E-05	143334	0	ENSE00003	-	-19
0,00118	139840	2	ENSE00003	-	21
6,28E-05	143298	0	ENSE0000(	-	-10
0,003555	143198	3	ENSE0000:	-	5
0,000489	143260	0	ENSE00003	-	17
0,006098	143316	9	ENSE0000:	-	-48
3,49E-05	143284	0	ENSE00003	-	45
0,002225	27416	0	ENSE00003	-	2
0,000475	143242	0	ENSE0000:	-	15
			ENSE00003	-	27
0,000126	143316	0	ENSE00003	-	-36
0,004867	143210	0	ENSE00003	-	31
			ENSE00003	-	
0,000287	142854	0	ENSE00003	-	
6,98E-06	143166	0	ENSE00003	-	-3
6,28E-05	143268	0	ENSE0000(	-	2
			ENSE0000:	-	30
1,4E-05	143288	0	ENSE0000(	-	-44
2,1E-05	143164	0	ENSE00003	-	0
2,09E-05	143296	0	ENSE00003	-	-23
1,4E-05	143308	0	ENSE00002	-	-42
1,4E-05	143264	0	ENSE0000:	-	17
			ENSE00002	-	8
0,001794	143262	0	ENSE0000(	-	-2
4,18E-05	143378	0	ENSE00003	-	-29
0,000168	143252	0	ENSE00003	-	-25
			ENSE00003	-	46
0,006387	143266	9	ENSE0000:	-	-3

6,98E-06	143258	0	ENSE00003	-	-1
0,001814	143322	0	ENSE00001	-	10
6,28E-05	143306	0	ENSE00003	-	43
6,98E-06	143224	0	ENSE00000	-	-45
			ENSE00001	-	23
			ENSE00001	-	16
0,008589	143328	8	ENSE00003	-	-24
4,88E-05	143344	0	ENSE00003	-	-7
			ENSE00003	-	-10
3,67E-05	136146	0	ENSE00001	-	-45
0,002311	143238	8	ENSE00003	-	-2
0,00418	143286	9	ENSE00003	-	24
0,004493	143320	0	ENSE00002	-	33
0,000279	143284	0	ENSE00003	-	-7
2,09E-05	143270	0	ENSE00000	-	6
0,000537	143330	0	ENSE00001	-	-35
0,001431	143232	0	ENSE00001	-	11
0,002087	143246	0	ENSE00001	-	-8
			ENSE00000	-	-49
1,4E-05	143292	0	ENSE00001	-	-45
0,002193	143198	2	ENSE00000	-	50
0,000126	143216	0	ENSE00003	-	-17
0,009886	143232	20	ENSE00003	-	
2,09E-05	143298	0	ENSE00001	-	40
			ENSE00001	-	19
0,000207	91588	2	ENSE00003	-	-20
0,002695	80138	3	ENSE00003	-	-22
			ENSE00003	-	-12
			ENSE00003	-	-18
0,007432	143302	6	ENSE00003	-	-9
0,002331	143302	2	ENSE00003	-	2
			ENSE00001	-	3
0,007398	143150	8	ENSE00001	-	2
0,000223	143276	0	ENSE00003	-	-36
0,003315	143288	0	ENSE00003	-	-46
1,4E-05	143124	0	ENSE00003	-	-23
0,009378	143310	6	ENSE00001	-	-46
0,001529	143242	3	ENSE00003	-	-34
1,4E-05	143288	0	ENSE00003	-	26
0,00113	143314	0	ENSE00001	-	16
6,98E-06	143300	0	ENSE00003	-	10
0,0049	143254	2	ENSE00002	-	5
			ENSE00002	-	9
0,007331	142964	9	ENSE00001	-	
0,002199	143242	2	ENSE00001	-	-19
2,79E-05	143288	0	ENSE00002	-	-3
0,009051	143086	4	ENSE00000	-	-16
0,004386	139302	2	ENSE00003	-	49
0,001864	143250	2	ENSE00003	-	-45
			ENSE00002	-	28

0,006982	135070	1	ENSE00003	-	-21
			ENSE00002	-	-31
0,007222	143178	6	ENSE00003	-	-40
0,001879	143124	0	ENSE00003	-	44
0,00014	143276	0	ENSE00001	-	-36
0,005393	143322	2	ENSE00003	-	-6
0,001271	143190	0	ENSE00001	-	41
0,000698	143302	0	ENSE00001	-	2
0,000677	143306	0	ENSE00001	-	-21
			ENSE00001	-	14
0,007893	143286	6	ENSE00001	-	-32
			ENSE00000	-	-39
6,98E-06	143206	0	ENSE00001	-	-21
			ENSE00001	-	35
0,00023	143276	0	ENSE00001	-	1
0,000384	143294	0	ENSE00003	-	-14
0,008011	143308	9	ENSE00003	-	-33
0,007212	143236	4	ENSE00003	-	-1
			ENSE00003	-	-12
			ENSE00002	-	1
7,01E-06	142624	0	ENSE00003	-	-47
			ENSE00000	-	7
			ENSE00001	-	9
			ENSE00001	-	38
			ENSE00001	-	14
0,00628	143320	7	ENSE00001	-	20
0,006351	143294	2	ENSE00003	-	-13
0,007244	143284	11	ENSE00001	-	28
0,00231	143278	0	ENSE00000	-	-11
2,09E-05	143308	0	ENSE00000	-	41
0,000999	143122	0	ENSE00001	-	-3
0,003036	143292	4	ENSE00001	-	-21
1,4E-05	143262	0	ENSE00003	-	10
2,09E-05	143242	0	ENSE00001	-	22
0,007384	143284	7	ENSE00003	-	-50
0,009444	143258	11	ENSE00001	-	46
			ENSE00000	-	-2
			ENSE00003	-	-9
6,98E-05	143354	0	ENSE00002	-	42
0,00663	143298	3	ENSE00001	-	2
0,004157	143358	7	ENSE00003	-	-8
			ENSE00001	-	1
0,002724	143152	3	ENSE00003	-	-2
0,009052	143286	11	ENSE00001	-	7
0,000865	143300	0	ENSE00000	-	2
0,000112	143234	0	ENSE00000	-	8
0,000335	143338	0	ENSE00003	-	-31
0,003641	138972	1	ENSE00000	-	-16
0,001863	143320	2	ENSE00001	-	-13
0,000621	143334	0	ENSE00001	-	-47

			ENSE00003	-	2
0,004207	143316	2	ENSE00000	-	-12
0,008394	143194	6	ENSE00003	-	-43
0,000363	143262	2	ENSE00003	-	-40
6,98E-06	143342	0	ENSE00003	-	29
			ENSE00001	-	35
0,000167	143320	0	ENSE00001	-	33
			ENSE00003	-	-27
0,000201	69556	0	ENSE00001	-	13
0,002793	143210	8	ENSE00001	-	10
1,4E-05	143260	0	ENSE00003	-	-6
			ENSE00000	-	-2
			ENSE00001	-	13
			ENSE00001	-	
			ENSE00003	-	-25
			ENSE00003	-	27
0,000831	37292	0	ENSE00003	-	-19
0,000935	143348	0	ENSE00003	-	35
0,002813	22394	0	ENSE00003	-	-2
			ENSE00001	-	9
0,00485	143306	4	ENSE00003	-	32
			ENSE00000	-	-15
0,000188	143262	0	ENSE00001	-	-44
0,004322	143236	5	ENSE00003	-	-45
0,00977	143294	12	ENSE00003	-	-33
0,005099	143362	5	ENSE00001	-	-1
0,008229	143280	7	ENSE00000	-	32
0,003135	143218	3	ENSE00000	-	-25
4,19E-05	143260	0	ENSE00003	-	11
			ENSE00000	-	-3
0,00709	143164	5	ENSE00001	-	10
0,005316	143342	1	ENSE00003	-	2
0,00211	109010	0	ENSE00000	-	2
			ENSE00001	-	-2
0,004271	143042	3	ENSE00001	-	-5
1,39E-05	143376	0	ENSE00001	-	-12
0,000719	143298	0	ENSE00003	-	3
2,79E-05	143286	0	ENSE00001	-	-3
0,003545	143304	2	ENSE00001	-	47
			ENSE00001	-	21
2,79E-05	143292	0	ENSE00001	-	-25
			ENSE00003	-	50
0,0024	143344	1	ENSE00001	-	9
1,4E-05	143324	0	ENSE00003	-	-2
0,001703	143298	0	ENSE00003	-	-2
0,000342	143306	0	ENSE00000	-	-13
0,001836	143248	0	ENSE00000	-	0
0,001752	143288	0	ENSE00003	-	14
0,00109	143146	1	ENSE00003	-	
1,4E-05	143358	0	ENSE00003	-	-16

2,09E-05	143308	0	ENSE00003	-	
0,000642	143242	0	ENSE00003	-	2
0,008682	143178	9	ENSE00003	-	13
0,00615	143252	2	ENSE00001	-	-9
0,009578	143246	7	ENSE00001	-	-6
0,004217	143232	2	ENSE00003	-	-7
5,58E-05	143296	0	ENSE00001	-	-27
2,09E-05	143330	0	ENSE00001	-	-5
0,007467	143036	4	ENSE00003	-	-7
0,002676	143100	2	ENSE00001	-	-2
0,000148	141740	0	ENSE00001	-	12
0,002673	143272	1	ENSE00003	-	-11
0,001428	84048	0	ENSE00001	-	48
0,007655	73676	177	ENSE00001	-	-37
			ENSE00001	-	-13
			ENSE00001	-	25
0,004245	44756	0	ENSE00001	-	-45
			ENSE00001	-	44
0,001665	44438	0	ENSE00001	-	-12
0,001419	88098	0	ENSE00001	-	-46
0,000105	143320	0	ENSE00003	-	4
0,001158	143330	1	ENSE00001	-	-2
0,000586	143360	0	ENSE00003	-	-6
0,008288	143342	10	ENSE00001	-	-22
0,000837	143306	1	ENSE00003	-	-37
2,1E-05	142766	0	ENSE00001	-	43
0,002366	143294	2	ENSE00000	-	14
0,008025	143310	7	ENSE00003	-	-33
			ENSE00001	-	-45
6,98E-06	143312	0	ENSE00003	-	-8
0,005795	143222	3	ENSE00001	-	2
			ENSE00001	-	7
0,003505	143228	3	ENSE00003	-	-5
6,98E-06	143342	0	ENSE00001	-	20
0,000678	143126	1	ENSE00003	-	-24
0,000209	143222	0	ENSE00003	-	-10
			ENSE00003	-	22
			ENSE00003	-	-25
0,000307	143294	0	ENSE00003	-	-2
2,79E-05	143300	0	ENSE00003	-	-7
0,003774	95390	0	ENSE00001	-	-19
0,008999	143242	14	ENSE00003	-	-2
9,07E-05	143294	0	ENSE00000	-	-14
			ENSE00003	-	14
			ENSE00001	-	-37
2,79E-05	143158	0	ENSE00001	-	-2
6,98E-06	143190	0	ENSE00001	-	15
0,002547	143294	1	ENSE00001	-	24
6,98E-06	143330	0	ENSE00001	-	-21
6,98E-06	143220	0	ENSE00001	-	-9

0,000126	143308	0	ENSE00003	-	46
0,000516	143322	0	ENSE00003	-	-41
0,004079	139250	2	ENSE00003	-	-30
0,008237	143254	5	ENSE00003	-	-34
0,001431	143212	0	ENSE00003	-	-13
0,000705	143266	1	ENSE00003	-	-19
8,37E-05	143286	0	ENSE00001	-	-16
0,000468	143192	0	ENSE00003	-	18
0,002756	143302	1	ENSE00000	-	5
5,58E-05	143344	0	ENSE00001	-	2
0,000789	143260	0	ENSE00000	-	6
0,002192	143254	1	ENSE00002	-	-29
0,007876	143338	5	ENSE00002	-	-13
			ENSE00003	-	-45
0,009065	143302	8	ENSE00001	-	20
0,000887	143178	0	ENSE00000	-	-26
0,004278	143306	5	ENSE00000	-	33
			ENSE00002	-	-10
0,003493	143132	1	ENSE00003	-	-1
			ENSE00003	-	14
0,004705	143250	7	ENSE00003	-	-30
0,008571	143266	8	ENSE00001	-	34
6,98E-06	143302	0	ENSE00000	-	0
0,005779	143274	3	ENSE00003	-	-39
0,002179	81244	0	ENSE00001	-	-30
0,007783	141596	9	ENSE00002	-	4
0,00011	54444	0	ENSE00003	-	26
8,96E-05	55808	0	ENSE00003	-	-13
9,18E-05	54440	0	ENSE00003	-	20
			ENSE00003	-	-23
0,000108	55326	0	ENSE00003	-	9
			ENSE00003	-	29
			ENSE00003	-	-41
			ENSE00003	-	-3
			ENSE00003	-	-10
0,002972	53168	6	ENSE00003	-	1
			ENSE00003	-	30
0,001967	57444	1	ENSE00003	-	0
0,001168	53094	0	ENSE00003	-	-44
0,003687	51798	3	ENSE00003	-	43
0,006797	50464	9	ENSE00003	-	-2
0,002027	57722	0	ENSE00003	-	-6
0,005825	77596	2	ENSE00003	-	-22
0,008318	71410	3	ENSE00003	-	-48
			ENSE00003	-	-28
0,004584	143312	2	ENSE00003	-	-29
			ENSE00003	-	-13
0,007398	143288	6	ENSE00003	-	-37
0,006861	143278	9	ENSE00003	-	1
0,005089	143254	4	ENSE00001	-	-20

0,008507	143064	20	ENSE00001	-	42
6,99E-05	143104	0	ENSE00003	-	5
0,000112	143266	0	ENSE00001	-	-48
0,004772	143326	9	ENSE00003	-	-48
			ENSE00003	-	-37
0,000852	143204	0	ENSE00001	-	3
0,000468	143288	0	ENSE00002	-	29
			ENSE00000	-	50
0,001053	143348	0	ENSE00001	-	-25
0,000188	143286	0	ENSE00001	-	-21
0,004969	143278	2	ENSE00003	-	2
0,001307	143040	0	ENSE00001	-	49
0,000174	143280	0	ENSE00003	-	15
0,001542	143290	0	ENSE00001	-	14
0,009336	143210	11	ENSE00003	-	14
0,009407	143292	13	ENSE00001	-	32
6,99E-06	143128	0	ENSE00000	-	33
1,4E-05	143110	0	ENSE00000	-	-2
0,006572	142882	3	ENSE00001	-	14
			ENSE00001	-	-42
			ENSE00003	-	-6
0,00841	143286	10	ENSE00003	-	19
0,007796	143274	10	ENSE00003	-	33
0,000768 7,68E-05	143312	0	ENSE00003	-	41
0,004643	143224	3	ENSE00001	-	36
0,0003	143288	0	ENSE00003	-	34
			ENSE00000	-	-20
0,001873	143066	0	ENSE00003	-	30
0,001298	143316	1	ENSE00003	-	-4
0,00014	143276	1	ENSE00003	-	2
2,79E-05	143318	0	ENSE00003	-	-19
2,79E-05	143398	0	ENSE00003	-	18
0,000112	143296	0	ENSE00003	-	-42
0,005961	143274	6	ENSE00003	-	-47
4,89E-05	143272	0	ENSE00003	-	-10
2,79E-05	143148	0	ENSE00001	-	-12
0,000133	143226	0	ENSE00003	-	2
0,008549	141184	11	ENSE00001	-	-30
0,000167	143338	0	ENSE00001	-	30
0,003905	142892	3	ENSE00001	-	1
			ENSE00003	-	24
0,008302	141540	9	ENSE00001	-	-1
0,001728	142384	2	ENSE00001	-	2
0,008839	143236	9	ENSE00003	-	-14
0,007087	143352	7	ENSE00002	-	7
0,003639	143166	2	ENSE00003	-	-16
4,19E-05	143312	0	ENSE00003	-	19
2,1E-05	142618	0	ENSE00003	-	-21
2,79E-05	143260	0	ENSE00003	-	-17
			ENSE00003	-	31

0,000202	143210	0			ENSE00003	-	-27
3,49E-05	143256	0			ENSE00001	-	-27
0,009607	143334	16			ENSE00000	-	7
0,000733	143322	2			ENSE00003	-	-30
0,000133	143208	0			ENSE00002	-	1
					ENSE00003	-	23
					ENSE00001	-	-12
0,499549	88644	9237			ENSE00003	-	-41
0,009393	143296	9			ENSE00001	-	-18
0,001038	142602	0			ENSE00001	-	-24
0,000105	143216	0			ENSE00000	-	-33
8,37E-05	143294	0			ENSE00003	-	11
0,002097	143092	0			ENSE00003	-	-35
					ENSE00001	-	9
					ENSE00001	-	32
0,007327	143164	8			ENSE00003	-	-5
					ENSE00001	-	
					ENSE00001	-	
0,007144	104708	6			ENSE00001	-	-10
4,79E-05	104438	0			ENSE00001	-	9
					ENSE00002	-	-9
					ENSE00000	-	-1
0,005336	91830	4			ENSE00001	-	-26
					ENSE00001	-	7
					ENSE00001	-	40
0,00092	82585	2			ENSE00001	-	33
0,001372	105695	0			ENSE00001	-	23
					ENSE00001	-	-29
0,003631	105216	0			ENSE00001	-	7
4,72E-05	105891	0			ENSE00001	-	-2
0,000188	143292	0	0,999999	0,998	ENSE00003	-	-26
			0,0059	0,098	ENSE00000	-	45
0,003121	143220	2	0,976704	0,78	ENSE00001	-	1
0,001598	143270	0	0,999997	1	ENSE00002	-	0
0,003336	143272	2	0,947664	0,636	ENSE00003	-	2
4,89E-05	143290	0	0,169881	0,466	ENSE00000	-	43
0,001878	143232	0	0,925075	0,684	ENSE00000	-	20
0,001103	143264	1	0,999948	0,99	ENSE00003	-	-2
6,98E-06	143308	0	0,003884	0,07	ENSE00003	-	-2
0,004395	141070	2	0,282637	0,454	ENSE00003	-	2
			0,136902	0,502	ENSE00000	-	-2
					ENSE00002	-	-2
3,5E-05	143026	0	0,787945	0,538	ENSE00003	-	40
0,005716	128054	16			ENSE00003	-	8
5,59E-05	143190	0	0,703136	0,504	ENSE00000	-	-10
			0,99985	0,942	ENSE00003	-	0
0,001884	143326	2			ENSE00003	-	-5
0,005774	143230	5	0,001054	0,108	ENSE00003	-	-13
						-	
			0,999933	0,756		-	31

			0,999979	0,928		-	-12
						-	
						-	
						-	
			0,99999	0,936		-	30
			0,999915	0,756		-	10
			0,999939	0,952		-	28
			0,999936	0,866		-	10
						-	
						-	
0,009088	142498	16			ENSE00002	-	
					ENSE00003	-	-41
2,1E-05	143006	0			ENSE00001	-	-35
					ENSE00002	-	-17
					ENSE00002	-	9
0,009277	143264	4			ENSE00003	-	-2
0,009811	143202	3			ENSE00001	-	
0,004646	143134	4			ENSE00002	-	-45
					ENSE00000	-	2
0,003952	143222	4			ENSE00002	-	-1
0,001263	143290	0			ENSE00003	-	-23
5,59E-05	143178	0			ENSE00003	-	26
					ENSE00003	-	

AI_pred_D	AI_pred_D	AI_pred_D	AI_pred_D	AI_pred_D	AI_pred_D	AI_pred_D	AI_pred_SY	ADD_PHRE
-13	-3	9	0	0,01	0	0	ATN1	23,2
								23,8
4	-8	-1	0	0	0	0	NF1	
-18	-39	-36	0	0	0	0	MKS1	
-7	-48	-43	0	0	0	0	JSRP1	8,527
-7	-28	23	0	0,03	0	0	FAM205A	
50	-23	-30	0	0	0	0	ZXDA	14,66
								22,5
								14,69
								8,608
35	-1	-48	0,01	0,03	0	0	IGSF22	14
								10,41
50	-27	-42	0	0	0	0	CWF19L2	17,78
								19,02
								2,993
								18
2	-1	-14	0,23	0,18	0	0	PPL	21,3
								8,716
-12	5	-1	0	0	0	0	NCL	14,43
								7,247
35	-48	-45	0	0,06	0	0	MEA1	16,21
								8,986
								5,748
								21
								17,97
								3,764
								8,955
								12,7
								20,5
								6,437
37	47	-16	0	0,04	0	0	CFAP74	10,13
-12	-28	-20	0	0	0,02	0	CEP104	21,5
-18	-18	-23	0	0	0	0	TNFRSF8	2,051
-2	6	27	0,04	0	0	0	PRAMEF9	22,5
-25	13	-4	0	0	0	0	PADI4	23,6
22	22	-21	0	0,02	0	0	IFFO2	21
7	46	6	0	0	0	0	VWA5B1	25,9
1	-21	1	0	0	0	0	EIF4G3	0,211
-41	-41	32	0	0,02	0	0	RHD	0,189
3	36	12	0	0	0	0	SEPN1	18,23
37	34	4	0,01	0,01	0,04	0,27	FHL3	24,1
-42	48	-13	0	0	0	0	RIMKLA	22,3
-12	-26	47	0	0	0	0	CFAP57	24,9
-1	-8	-1	0	0	0	0	DMBX1	16
14	41	12	0	0	0	0	C8A	10,73
-47	-9	-5	0	0	0	0	CACHD1	23,6

-20	-20	28	0	0,19	0	0,19	IFI44L	1,107
21	-37	24	0	0	0	0	CTBS	19,06
12	47	-20	0,03	0	0	0	WDR63	11,26
1	3	-8	0	0	0,27	0	SASS6	14,2
22	30	-35	0	0	0	0	DENND2C	22,9
-22	10	27	0	0	0	0	DENND2C	22,3
18	-38	26	0	0	0	0	TTF2	4,434
42	-6	-24	0,01	0,01	0	0	NOTCH2	30
								21,7
-25	5	-7	0	0	0	0	FAM72C	16,49
-18	-40	38	0	0,09	0	0	CIART	26,5
6	36	6	0,02	0,07	0	0	ADAMTSL4	0,011
37	1	-45	0	0	0	0,01	HRNR	0,096
0	-28	6	0	0	0	0	HRNR	22,9
-11	-42	-49	0	0	0	0	CRTC2	27,4
-5	-25	9	0,06	0	0	0	PMVK	20,4
-41	-2	2	0	0	0	0	ARHGEF11	19,96
-6	33	-3	0	0	0	0	ACKR1	24,1
44	12	33	0	0	0	0	F5	0,006
4	-1	41	0	0	0	0	PRRC2C	0,007
-23	-38	25	0	0	0	0	SERPINC1	27,1
36	36	-8	0	0,01	0	0	F13B	22,6
20	2	-34	0	0	0	0	ASPM	27,8
34	15	-38	0	0	0	0	IGFN1	9,081
4	-5	-29	0	0	0	0	ETNK2	26,3
-40	-40	46	0	0,03	0	0	CDK18	27,3
-39	24	-39	0	0,04	0	0	C4BPA	13,88
46	6	-1	0	0	0	0	EPRS	28,7
42	-23	-33	0	0	0	0	WNT9A	15,75
-49	-50	7	0	0	0	0	OBSCN	6,584
1	27	-35	0	0	0	0	OBSCN	24,1
-2	-49	-9	0	0	0	0	ZP4	24,6
-46	11	20	0	0	0	0	CHRM3	24,1
-15	-1	16	0	0	0	0	SUV39H2	15,42
50	9	-17	0	0	0	0	ARHGAP21	24,1
15	21	-33	0	0	0	0	YME1L1	32
-42	-9	-11	0	0	0	0	SVIL	26,1
21	-5	15	0	0	0	0	EPC1	22,9
2	14	-3	0	0	0	0	ANKRD30A	2,311
								2,139
								16,21
								0,05
-21	5	-35	0	0	0	0	IPMK	10,5
42	0	-4	0	0	0	0	FAM35A	3,212
37	-46	-2	0	0	0	0	IFIT5	14,39
46	0	-5	0	0	0	0	CYP26C1	24,8
42	-3	-49	0	0	0,01	0,03	PLCE1	7,193
-38	-38	-21	0	0	0	0	CYP2C18	23,6
18	16	28	0	0	0	0	ZNF518A	12,83
-16	44	45	0,01	0	0	0	SEC31B	17,79

-6	-22	-41	0,07	0,02	0	0	CUEDC2	1,172
6	29	-11	0	0	0	0	CFAP43	0,014
1	4	48	0	0	0	0	INPP5F	18,27
-45	28	-1	0	0	0	0	BTBD16	0,102
-37	46	6	0	0	0	0	MGMT	26
-45	-4	-34	0	0	0	0	TCERG1L	18,93
-16	-49	3	0	0	0	0	DPYSL4	7,272
21	7	1	0	0	0	0	CFAP46	24,2
28	30	-8	0	0	0	0	VENTX	6,873
9	50	-40	0	0	0	0	PKP3	3,324
34	-47	-4	0	0	0	0	LMNTD2	0,72
-31	-24	-31	0	0	0	0	LMNTD2	8,013
-24	21	22	0	0	0	0	PHRF1	3,144
-38	-38	-6	0	0,02	0	0	MRPL23	0,024
								8,682
1	44	-46	0	0	0,01	0	TRIM21	0,026
-47	-45	17	0	0	0	0	OR51E2	24,7
-18	36	14	0	0	0	0	FAM160A2	20,5
-14	2	-41	0	0	0	0	OR5P3	17,71
-1	27	-50	0,01	0	0	0	OR10A6	16,95
-7	16	-20	0	0	0	0	USP47	23,2
-33	1	7	0	0	0	0	KCNJ11	25,7
25	25	5	0	0	0	0	IGSF22	14,5
								21,9
-11	9	-50	0	0	0,01	0,01	OR4A16	6,909
5	-10	-14	0	0,01	0	0	OR5B2	6,851
-14	1	-33	0	0	0,07	0,03	HNRNPUL2	15,78
-32	-19	40	0	0	0	0	SIPA1	21,7
12	-39	34	0	0	0	0	CD248	2,388
10	7	0	0,07	0	0	0	SLC29A2	19,43
14	-4	0	0,01	0	0	0	BBS1	25,2
4	-49	-9	0	0,01	0	0	FGF19	25,4
-33	-15	41	0	0,05	0,02	0	SLCO2B1	9,936
31	32	28	0	0	0	0	TENM4	16,99
-48	6	12	0,01	0	0	0	VSTM5	17,84
-23	-41	-6	0	0	0	0	ANKK1	4,972
-12	-11	-12	0	0	0,13	0	DPAGT1	22
45	-48	-3	0,01	0,01	0	0	OR10D3	22,4
15	-43	18	0	0	0	0	ROBO3	24,6
-36	32	-7	0	0,01	0	0	PATE4	0,031
3	1	3	0,24	0,07	0	0	ST3GAL4	21,2
15	47	-41	0	0,03	0	0	RHNO1	12,67
-17	-17	49	0	0	0,02	0	C1RL	20,7
25	8	2	0	0	0	0	CLEC6A	15,59
-48	-14	34	0	0	0	0,01	PRB4	0,054
-24	0	-8	0	0	0	0	PRB1	6,162
7	7	-2	0,11	0	0	0	PIK3C2G	24,1
8	-27	-5	0	0	0	0	FGD4	24,5
-2	46	-2	0,13	0,04	0	0	ABCD2	26,1
5	4	-2	0	0	0,03	0,01	PRPH	22,4

9	39	9	0	0	0	0	NCKAP5L	23
25	-11	47	0,04	0	0	0	KRT81	24,5
48	-5	-22	0	0	0	0	KRT6B	23,3
-38	17	-6	0	0	0	0	ERBB3	23
-40	3	-25	0	0,01	0	0	STAT6	24,4
33	8	-38	0	0,01	0	0	PPFIA2	24,4
-20	8	-6	0,02	0	0	0	LRRIQ1	25,8
-1	-31	1	0	0	0	0	FGD6	26,2
-1	-7	-8	0,04	0	0	0	FGD6	25,2
6	7	45	0	0	0	0	FGD6	0,012
-47	49	10	0	0,01	0	0	OAS3	25,4
33	1	32	0	0	0	0	SRRM4	8,408
10	4	29	0	0	0	0	DNAH10	24,8
12	-42	1	0	0	0	0	DHX37	22,1
46	46	-47	0	0	0	0	TMEM132	25
-19	45	16	0	0	0	0	ZMYM5	0,178
-4	-5	-4	0	0	0	0	ATP12A	23,9
-34	-8	48	0	0,02	0	0	FRY	25,7
-21	30	-47	0	0	0	0	BRCA2	26,2
4	44	-4	0,01	0	0	0	NAA16	22,7
-40	-2	7	0	0	0	0,04	CLN5	13,44
-12	-14	19	0	0	0	0	MYCBP2	28,6
-32	5	50	0	0	0	0	DNAJC3	24
-35	19	-8	0	0,02	0	0	OR11H12	0,315
								7,826
-16	-1	17	0,08	0,31	0	0	OR11H2	24,1
41	-3	8	0	0	0	0	RNASE4	15,08
46	8	41	0	0	0	0	RNASE1	19,91
37	-1	5	0,41	0,12	0,01	0	NDRG2	29,7
-15	-36	50	0	0	0	0	LRFN5	23,9
-15	-3	2	0	0	0	0	EXOC5	17,41
5	3	1	0	0	0	0	ZBTB25	22,6
-25	-2	19	0	0	0	0	SUSD6	11,15
-11	-5	-36	0	0	0	0,03	MAP3K9	27
24	-1	24	0	0	0,06	0	NRDE2	24,7
45	46	45	0,03	0	0	0	ATG2B	14,32
-29	-2	12	0	0	0	0	BEGAIN	18,68
-18	2	-2	0	0	0	0	RTL1	15,95
-21	2	-38	0	0	0	0	RP11-736N	14,98
-19	-20	1	0	0	0	0	AHNAK2	3,046
-22	-24	33	0	0	0	0	AHNAK2	0,625
9	24	-33	0	0	0	0	AHNAK2	0,006
37	-17	-31	0	0	0	0	AHNAK2	16,64
32	33	5	0	0	0	0	AHNAK2	0,493
-48	40	27	0	0	0	0	AHNAK2	0,001
4	31	-23	0	0	0	0	AHNAK2	25,8
37	15	-8	0	0	0	0	GOLGA8M	15,12
3	-33	10	0	0	0	0	GOLGA8K	16,59
50	-21	-11	0,01	0	0	0	GOLGA8K	21,3
-26	-34	24	0	0	0,01	0,01	GOLGA8K	14,84

								7,667
45	-2	16	0	0	0	0	EXD1	21,9
-23	3	-2	0	0,02	0	0	WDR76	3,161
-44	-42	31	0,07	0	0,12	0	SPPL2A	26,1
7	-2	-7	0	0	0	0	CGNL1	22,7
-6	1	-4	0	0	0	0	BNIP2	18,61
-27	-25	3	0	0	0	0	VPS13C	19
-11	-1	-11	0	0	0	0,06	GRAMD2	14,9
-4	-2	7	0	0	0	0	GOLGA6C	3,877
0	-13	27	0	0	0,03	0	KIAA1024	6,995
47	20	47	0,01	0	0	0	ALPK3	26,2
-34	12	14	0	0	0	0	SEMA4B	20,6
1	10	-16	0	0	0,01	0	WFIKKN1	0,375
-38	-7	44	0	0	0	0	WDR90	2,568
-8	-8	26	0,02	0,06	0	0	TELO2	24,9
21	7	-46	0	0	0	0	SPSB3	7,76
-2	1	-2	0	0	0	0,04	RPL3L	19,99
-7	49	-7	0	0	0	0	RNF151	0,001
17	43	35	0,01	0,05	0	0	MEFV	21,7
9	44	-34	0	0	0	0	MEFV	0,018
9	43	42	0,01	0,01	0	0	CDIP1	21
-5	1	8	0	0	0	0	TEKT5	22,5
-40	38	7	0	0	0	0	PRM2	7,638
-27	-15	10	0	0	0	0	NOMO1	25,2
22	-29	22	0	0,01	0	0	MYH11	24,7
-42	-13	5	0,02	0	0	0	PDILT	24,7
-3	19	45	0	0	0	0	PDILT	22,5
13	-5	-47	0	0	0	0	DNAH3	23,6
1	-10	-36	0,01	0	0	0	TNRC6A	17,61
-19	2	14	0	0	0	0	NPIPB9	7,218
-24	-2	15	0	0	0	0	ZNF267	5,988
-26	-26	-31	0	0	0	0	N4BP1	14,99
6	-1	-8	0	0	0	0	SALL1	7,94
-11	-11	49	0	0,05	0	0	C16orf70	23,5
								11,39
								0,262
-10	-24	16	0	0	0	0	PKD1L3	19,86
-17	1	28	0,09	0	0	0	DHX38	20,4
3	-38	1	0	0	0	0	CDYL2	25,2
21	49	-23	0	0,03	0,01	0	HSDL1	23,4
-43	-1	0	0,07	0,16	0,09	0	CTU2	13,77
-22	19	-22	0	0	0,01	0	CTU2	6,247
-10	-42	-41	0,01	0	0	0	PIEZO1	22,1
3	5	-34	0	0	0	0	SPATA2L	0,371
-14	2	9	0	0	0	0	CLUH	21,6
42	-1	-8	0	0	0	0	ITGAE	2,598
21	22	-28	0	0	0	0,02	P2RX1	20,6
-31	24	27	0	0	0	0	ENO3	25,4
10	4	43	0	0	0	0	TEKT1	20,1
-21	-3	-2	0	0	0	0	SLC2A4	17,41

-3	-17	4	0,17	0	0,06	0	CTC1	10,27
-1	6	50	0	0	0	0	HS3ST3A1	15,09
26	-40	-48	0	0	0	0	SPAG5	12,96
6	-6	-14	0	0	0	0	TOP2A	23,9
-2	0	19	0	0	0	0	KRTAP4-8	0,269
35	-37	1	0,01	0	0	0	KRTAP9-7	2,967
15	-24	-26	0	0,01	0	0	PLEKHH3	26,1
-41	29	44	0	0	0	0	CRHR1	18,77
27	28	-43	0,01	0,12	0	0,06	CDC27	23,3
-21	-21	28	0	0	0	0	ZNF652	21,7
-9	-1	5	0	0	0	0,15	MKS1	23,1
-26	-13	18	0	0,01	0	0	RGS9	8,906
-32	2	-35	0,02	0,01	0	0	GRIN2C	24,9
-41	20	1	0	0	0	0	MYO15B	25,3
-42	-31	-26	0	0	0	0	FOXJ1	22,5
29	9	-4	0	0	0	0	FSCN2	24,4
0	10	36	0	0	0	0	ESCO1	25,8
-23	-7	-18	0	0	0	0,07	OSBPL1A	20,3
-43	37	-49	0,01	0	0	0	ASXL3	19,34
11	-37	-8	0	0	0	0	PHLPP1	24,1
-19	-37	28	0	0,01	0	0	ZNF236	17,42
6	20	-28	0	0	0	0	APC2	18,8
								9,058
-38	36	13	0	0	0	0	ZFR2	0,027
-42	19	-1	0	0	0	0	CHAF1A	15,36
33	2	-32	0	0	0	0	PLIN4	10,64
32	33	32	0	0	0	0	PLIN4	12,72
33	7	-11	0	0	0	0	PLIN4	16,57
-1	-17	1	0	0	0	0	PLIN4	0,11
9	-9	1	0,04	0	0	0	LONP1	22,2
41	2	1	0	0	0	0	RFX2	24,3
-9	8	18	0	0	0	0,01	CLEC4M	0,011
-34	2	12	0	0	0	0	PPAN	1,66
39	-5	-26	0,01	0,01	0	0	YIPF2	20,3
0	42	-5	0	0	0	0	ACP5	22,9
7	2	-26	0	0	0	0	EPS15L1	24,2
6	-13	36	0	0	0	0	CPAMD8	16,13
-40	-40	16	0	0	0	0	TMEM59L	3,466
42	26	-8	0	0	0,02	0	COMP	26,8
3	16	-26	0,01	0	0	0	COPE	26,7
13	2	-11	0	0	0	0	SUGP1	18,24
50	-42	-37	0	0	0	0	ZNF101	9,048
-7	10	39	0	0	0	0	ZNF676	3,989
								0,02
6	-19	-12	0,01	0,02	0	0,01	NPHS1	24,1
14	14	15	0	0	0	0,01	CNTD2	10,3
-25	-6	24	0	0	0	0	CLASRP	7,522
-44	-4	-43	0	0	0	0	DACT3	15,69
-11	5	50	0	0	0	0	SLC1A5	19,11
49	28	49	0,01	0	0	0	CGB	0,19

33	-5	5	0	0	0,03	0	CGB2	22,8
1	2	-16	0	0	0	0	CGB1	7,152
35	-4	-40	0	0	0,03	0,03	SLC6A16	2,587
37	-1	-3	0	0	0	0	ZNF845	2,152
12	35	-42	0	0	0	0,01	SSC5D	0,01
34	2	1	0	0	0	0	PXDN	21,6
29	29	5	0	0,01	0	0	RAD51AP2	16,93
-26	46	-26	0	0,02	0	0	CAD	6,728
29	28	-1	0	0	0	0	BIRC6	17,02
-39	-16	47	0	0	0	0	VIT	27,9
-49	-30	-3	0	0	0	0	C1GALT1C	26,8
34	4	-47	0,01	0	0	0	LRPPRC	14,17
-5	8	-1	0	0	0	0,02	C2orf73	21,3
32	-37	40	0	0	0	0	USP34	23,5
9	1	9	0	0	0	0	PCYOX1	16,79
8	8	-1	0	0,01	0	0	AUP1	17,04
-12	35	-32	0	0	0	0	GCFC2	24,4
-35	-2	-6	0,17	0	0	0	DNAH6	15,45
38	41	38	0	0	0	0	THNSL2	21,7
-1	0	15	0	0	0	0	TRIM43	14,55
8	21	-21	0	0	0	0,01	ANKRD36C	18,21
-26	15	-1	0	0	0	0	CNGA3	19,4
43	9	43	0	0	0	0	POTEI	16,27
49	2	4	0	0	0	0	POTEI	0,876
25	-20	7	0	0	0	0	POTEI	14,96
-33	-7	20	0	0	0	0	GPR39	1,591
26	16	-13	0,12	0	0	0	RAB3GAP1	4,637
15	-12	28	0	0	0	0	NEB	17,69
-42	2	-11	0,01	0	0	0	GALNT5	24,3
-12	-12	28	0	0	0	0	TTC21B	21,3
20	47	24	0,01	0	0	0	CERS6	20,9
46	34	5	0	0	0	0	TTC30A	18,69
-27	17	43	0	0	0	0	TTN	22,4
-41	-15	16	0	0,11	0	0,05	FSIP2	9,821
1	3	18	0	0	0	0	EEF1B2	23
47	2	-28	0	0,07	0	0,13	DYTN	22,6
10	27	5	0	0,02	0	0	CRYGD	9,179
-17	-35	20	0	0	0	0	BCS1L	27,9
-19	3	30	0	0	0,02	0	STK11IP	17,39
-28	2	33	0	0	0	0	CROCC2	0,002
38	-1	29	0	0	0	0	SNPH	22,9
-47	1	-47	0	0	0	0	ADRA1D	28,8
29	-40	-36	0	0,14	0	0,11	KIF16B	23,3
-27	-28	16	0	0,01	0	0	CST1	0,001
-24	-4	0	0,08	0	0	0	MMP24	23,2
-50	8	48	0,15	0	0	0	ACTR5	23,9
35	4	2	0	0	0	0	PABPC1L	22,3
-9	-16	32	0	0	0	0	SEMG2	2,155
16	-5	26	0	0	0	0	ZSWIM1	15,17
-3	-12	2	0	0	0	0	ZNFX1	26,9

50	2	50	0	0	0	0	MTG2	0,055
14	2	-44	0	0	0	0	RBBP8NL	11,89
33	-21	33	0	0	0	0,03	RBM11	12,41
-34	7	10	0	0	0	0	NRIP1	24,1
-43	-43	49	0	0	0	0	USP16	22,9
-10	-26	18	0	0	0	0	CLDN8	25
0	-6	1	0	0,01	0	0	URB1	15,37
2	34	2	0,02	0	0	0	IFNGR2	4,443
-2	-35	-2	0	0	0	0	KCNE1	2,36
1	-31	9	0	0	0	0,02	DOPEY2	25,8
-40	-6	-42	0	0	0	0	CHAF1B	22,9
0	-26	0	0	0,01	0	0	SIK1	14,33
-25	-39	11	0	0	0	0	PWP2	22,5
								0,698
9	-5	-24	0	0	0	0	ICOSLG	3,199
45	-16	-32	0	0	0	0	DNMT3L	19,07
7	5	43	0,04	0	0	0	TMEM191	6,212
-18	-5	1	0	0	0	0	RIMBP3	1,724
35	-1	-23	0,01	0,02	0,01	0,01	PRODH	19,84
1	-6	-32	0	0	0	0	TBX1	26,9
-33	-38	32	0	0,05	0	0,04	TANGO2	22,8
7	12	25	0	0	0	0	SCARF2	22,5
39	40	-43	0	0,01	0	0	DRICH1	0,017
10	17	20	0	0	0	0	SPECC1L	18,84
2	12	16	0	0	0	0	SGSM1	23,9
-42	-11	-42	0	0	0	0	KIAA1671	6,372
0	2	-28	0	0	0	0,01	C22orf31	2,572
18	-25	-10	0	0	0	0	SLC5A4	23,3
-12	28	27	0,09	0	0	0	TOM1	32
-1	-3	38	0	0	0	0	MKL1	22,8
5	2	5	0	0	0	0	TNFRSF13(	18,94
-33	-8	32	0	0	0	0	ARHGAP8	12,87
46	46	0	0	0	0	0	FAM118A	18,8
39	29	16	0	0	0	0	CELSR1	22,9
17	41	-1	0,01	0	0,01	0	CPNE9	25,9
-9	-6	35	0	0	0	0	VILL	23,8
-6	-15	19	0	0,01	0	0	XYLB	13,05
9	10	-38	0	0	0	0	SCN5A	24,2
17	22	17	0	0	0	0	TRAK1	24,4
2	36	-2	0	0	0	0	ZNF502	15,39
43	-33	-8	0	0,01	0	0	ALS2CL	6,163
35	-50	5	0,01	0	0,14	0,68	SLC26A6	21,9
-23	-5	9	0,02	0,14	0	0	LAMB2	14,16
37	-1	-43	0,99	0,08	0	0	MST1R	17,52
45	-2	5	0,38	0,01	0	0	RASSF1	24,1
-28	5	-28	0	0	0	0	STAB1	1,877
-48	0	5	0	0	0,14	0	STAB1	22,5
1	0	1	0,89	0,15	0	0	GNL3	23,3
								22,5
-13	3	-38	0	0	0	0	LRIG1	9,071

								19,82
-29	-29	-26	0	0	0	0	MYH15	23,9
9	4	31	0,04	0	0	0	C3orf17	23,8
-44	-14	35	0	0	0	0	GOLGB1	0,002
-40	13	-17	0	0	0	0	GOLGB1	21,2
38	4	-29	0	0	0	0,08	KIAA1257	22,6
-2	-27	44	0	0	0	0	TRIM42	25,6
-19	24	0	0	0	0	0	C3orf58	29,9
-13	16	42	0	0	0	0	CPB1	23,5
27	-1	20	0	0	0,06	0	RARRES1	0,298
-3	49	-3	0,1	0,01	0	0	LIPH	29,5
18	37	-9	0	0	0	0	MUC4	0,005
0	-8	40	0	0,03	0	0	MUC4	0,268
20	-37	-17	0	0	0	0	MUC4	16,62
-50	27	-21	0	0	0	0	MUC4	14,63
-8	-42	7	0	0	0	0	MUC4	14,69
22	-45	3	0	0	0	0	MUC4	15,82
-4	-12	36	0	0	0	0	MUC4	5,196
36	28	-20	0	0	0	0	MUC4	7,3
50	-45	3	0	0	0	0	MUC4	13,4
-1	31	30	0	0	0	0	GAK	10,15
17	-2	-28	0	0	0,01	0	RGS12	14,36
45	7	-1	0	0	0	0	MAN2B2	16,01
-6	-2	-1	0	0,01	0	0	HTRA3	25,8
36	-37	36	0	0	0,06	0,01	TRMT44	13,14
-1	4	-11	0	0	0,01	0,05	ARAP2	22,9
-2	-21	0	0	0	0,02	0	CORIN	25,9
5	-1	15	0	0	0	0	ADGRL3	9,022
-29	9	38	0	0	0	0	JCHAIN	24,9
15	39	-44	0	0	0	0	USO1	20,7
-25	-25	40	0	0,02	0	0,12	FRAS1	32
24	24	1	0	0	0	0	WDFY3	0,006
15	-4	15	0,24	0,05	0	0	PLA2G12A	26,7
21	-1	20	0	0	0	0	SYNPO2	28
12	-50	-46	0,11	0,01	0	0	ANXA5	27,4
2	0	-15	0	0	0	0	ZNF330	27,4
-18	22	-18	0,32	0,11	0	0	ARFIP1	19,02
-23	-2	4	0	0	0	0	GLRB	27,6
40	-3	-6	0	0	0	0,01	VEGFC	22
27	27	3	0	0	0	0	IRF2	19,81
23	43	-15	0	0	0	0	PRDM9	10,51
34	-4	-47	0	0	0	0	PDZD2	13,75
29	34	-14	0	0	0,01	0,07	AGXT2	0,194
1	14	13	0	0	0	0	NIPBL	22,2
-6	-24	-4	0	0	0	0	C5orf42	17,89
-15	50	-15	0	0	0	0,02	NUP155	24,1
-46	-7	40	0	0	0	0	PTCD2	23,1
-14	-48	2	0	0	0	0	ANKRD34E	9,277
19	-11	1	0	0	0	0	GPR150	27,9
15	-1	-25	0	0	0	0	CHD1	23

-14	-42	8	0,03	0	0,04	0	EPB41L4A	26
10	10	-45	0	0,12	0	0,21	EPB41L4A	33
8	31	8	0	0	0	0	CEP120	27,5
-1	-3	-33	0	0	0	0,01	FBN2	23
47	16	-1	0	0	0	0,01	RAD50	23,5
40	-11	-1	0	0	0	0	AFF4	22,1
-11	-34	-5	0	0	0	0	CAMLG	32
-5	5	-1	0	0	0,05	0	PKD2L2	27,2
-16	-30	44	0	0	0	0	PCDHB7	24
34	-5	33	0	0	0	0	PCDHB11	25,6
-15	-2	-1	0	0	0	0	PCDHGA3	26,5
28	-29	-2	0	0	0	0	PCDHGB4	23,6
42	31	-10	0	0	0	0	PCDHGB4	23,5
6	50	-43	0	0	0	0	SPINK5	9,459
-49	-23	-49	0,04	0	0	0	ADRB2	17,38
1	3	17	0,02	0,29	0	0	GABRA6	25,7
45	-15	45	0	0	0	0	DUSP1	22,3
1	-12	-5	0	0	0	0	CPEB4	22,6
-26	49	-10	0	0	0	0	FAM193B	12,48
-25	-50	16	0	0	0	0	PHYKPL	27,1
12	-6	1	0	0	0	0,01	CAGE1	0,632
-14	3	2	0	0	0	0	GCM2	14,99
18	-41	-5	0	0	0,01	0	NUP153	23,6
-4	-29	-5	0	0,01	0	0,02	CDKAL1	25,2
1	-30	1	0	0	0	0	MDC1	0,001
34	-27	-1	0	0	0	0	MUC22	0,001
-14	-24	-4	0	0	0	0	HLA-C	2,775
24	-6	-26	0	0	0	0	HLA-C	2,074
-30	-12	-10	0,01	0	0	0	HLA-C	0,008
14	-16	-18	0	0	0	0	HLA-C	15,83
-9	-39	-9	0	0,01	0	0	HLA-C	4,871
-40	1	-40	0	0	0	0	HLA-C	0,009
-10	1	-1	0	0	0	0	HLA-C	10,17
13	0	-23	0	0	0	0	HLA-C	2,796
-4	-40	-37	0	0	0	0	HLA-C	18,96
0	-42	8	0	0	0	0	HLA-C	10,78
-9	-10	29	0	0	0	0	HLA-C	0,201
18	50	-46	0,01	0	0	0	HLA-C	21,9
-8	-13	9	0	0	0	0	HLA-C	17,1
34	-21	30	0	0	0	0	HLA-C	0,037
-11	4	-33	0	0	0	0	HLA-C	0,001
-1	-18	5	0,1	0	0	0	HLA-C	0,001
38	2	23	0	0,03	0	0	HLA-C	24,1
24	-48	9	0	0,03	0	0	HLA-C	2,076
41	38	-19	0	0	0	0	HLA-DRB1	15,39
7	-37	-10	0	0,01	0	0	ITPR3	22,4
0	-8	5	0	0	0	0	GRM4	23,8
-14	-37	14	0	0	0	0	BRPF3	27,6
16	1	8	0	0	0	0	XPO5	24,8
3	-22	22	0	0	0	0	POLH	22,2

-23	1	-3	0	0	0,01	0	BMP5	16,94
-19	36	27	0	0	0	0	DST	0,461
29	2	-34	0	0,02	0	0	CNR1	19,36
41	48	-48	0,01	0	0	0	LACE1	7,107
15	-16	-13	0	0	0	0	MARCKS	17,33
-12	-3	-4	0	0,01	0	0	LAMA2	25,7
-2	0	29	0,01	0	0	0	EPB41L2	27,1
-39	-38	1	0	0	0,04	0	LRP11	3,154
0	9	-19	0	0	0	0	FNDC1	4,094
34	10	33	0	0	0	0	DACT2	0,021
-40	-21	48	0,25	0,01	0	0	FAM20C	24,6
2	-47	-29	0	0	0	0	TMEM184	0,001
-4	-47	-5	0,05	0	0	0	AP5Z1	1,18
-6	-1	3	0	0	0	0	AHR	0,001
-47	-24	-22	0	0,03	0	0	DNAH11	22,4
-3	36	31	0	0	0	0	INMT	1,987
-4	-9	6	0	0,03	0	0	AVL9	27,8
24	-2	48	0	0,01	0	0	NPC1L1	23,2
-11	-2	49	0	0	0	0	OGDH	25
44	-15	-47	0	0	0	0	NACAD	0,248
41	41	-34	0	0	0	0	TNS3	23,4
-35	-26	-30	0	0	0	0	C7orf57	23,6
-44	-20	21	0	0	0	0	COBL	14,41
-2	-7	-2	0	0	0	0	COBL	10,95
-38	-38	-22	0	0	0,01	0	ZNF479	2,976
-29	37	34	0	0	0	0,04	FKBP6	19,35
-1	-20	1	0	0	0,01	0	BAZ1B	19,5
-39	-16	-26	0	0	0	0	CCDC146	22,8
26	43	25	0	0	0	0	PTCD1	3,233
-26	2	18	0	0,02	0	0	CYP3A43	22,7
10	-20	34	0	0	0	0	NYAP1	29,8
-11	-13	-28	0	0	0	0	MUC3A	12,97
37	17	-20	0	0	0	0,05	PLOD3	27,2
-49	13	9	0	0	0	0	SLC26A4	29,1
32	20	26	0	0	0	0	IFRD1	25,4
-25	-7	16	0	0	0	0	CADPS2	14,68
-5	-3	-6	0	0,04	0	0	AKR1D1	9,084
22	46	13	0	0,01	0	0	NOBOX	6,807
-21	2	-21	0	0	0	0	ZNF786	11,78
-7	-7	2	0	0	0,07	0	LZTS1	28,3
-39	5	35	0	0	0,14	0,03	POLR3D	22,9
-17	5	-16	0	0	0	0	KIF13B	2,535
-15	2	-14	0	0	0	0	GSR	19,77
4	2	4	0	0	0	0	RAB11FIP1	23,8
1	-22	-45	0	0	0	0	ASH2L	27,1
-1	-22	1	0	0	0,02	0	RNF170	17,88
-6	-27	-31	0	0	0	0	ATP6V1H	20,6
-2	-29	-41	0	0	0	0	LYPLA1	7,65
22	41	-9	0	0	0	0	ADHFE1	17,97
24	24	-19	0	0,03	0	0	NUDCD1	23,5

18	18	6	0	0	0	0	NDRG1	22
14	-27	22	0	0	0	0	ZNF696	11,93
34	7	8	0	0	0	0	ZC3H3	13,23
11	-1	30	0,01	0,15	0	0	WDR97	16,74
-3	-29	34	0,01	0	0,01	0	BOP1	24,4
48	-1	23	0	0	0	0	SLC39A4	29,5
-1	-20	5	0,1	0,01	0	0	SLC39A4	12,43
-12	25	2	0,02	0	0	0	WASH1	8,357
48	39	-37	0	0	0	0	KIF24	0,08
12	3	-44	0	0	0	0	ARHGEF39	21,4
36	-10	1	0	0	0	0	TRPM6	6,36
9	7	4	0	0	0	0	PRUNE2	4,578
13	26	-50	0	0	0	0	WNK2	0,009
-3	17	-1	0	0	0	0	GALNT12	18,72
12	-3	-2	0	0	0	0	OR1L1	0,006
-21	-5	9	0	0	0	0	EXOSC2	21,4
-25	32	-5	0	0	0	0	MAGEB6	15,75
-12	47	-5	0	0	0,01	0	DUSP21	25,6
-28	17	21	0	0	0	0	GAGE12G	0,01
44	-1	-9	0	0	0	0	BMP15	12,53
7	-31	-46	0	0	0	0	ZXDB	13,93
-50	-44	-6	0	0	0	0	ZXDA	2,518
-2	2	-2	0	0	0	0	ZXDA	0,235
-9	-2	-9	0	0	0	0	ZXDA	4,813
-19	-2	-1	0	0	0	0	ACRC	1,921
34	-29	34	0	0	0	0	TCEAL8	23,1
22	39	-34	0	0	0	0	ADGRG4	0,769
-10	-2	-10	0	0	0	0	MTMR1	25,5
0	-4	0	0	0	0	0,85	FCRL4	34
23	-2	23	0	0	0,02	0,02	PARP1	21,5
-23	1	25	0,09	0	0	0	NUP133	32
-29	-29	0	0	0	0,62	0,49	ERCC6	35
-2	2	1	0	0	0,01	0	CPXM2	26,5
0	0	48	0	0,01	0	0,05	NAT10	23
0	-6	48	0	0,24	0	0	ZP1	24,1
40	-49	0	0	0	0	0,77	ERN2	35
-41	-1	-5	0	0	0	0	TOP3A	23,9
0	2	18	0,53	0,01	0	0	CGB2	25,8
1	-29	-50	0	0,01	0	0,01	SLC27A5	32
9	26	-2	0,03	0	0,04	0	ANKRD36	23,6
-1	-1	-22	0	0	0	0	ERBB4	26,2
2	23	2	0,02	0,52	0	0	SERHL2	1,484
41	-4	0	0	0	0	0,02	LNX1	23,9
36	3	0	0	0	0,01	0,02	HLA-C	24,5
-2	-2	46	0	0,01	0	0,01	DBNL	22,6
-8	4	2	0	0,07	0	0,02	MAMDC4	1,99
								20,7
2	-47	2	0,09	0,99	0	0	POTEE	22,8

-2	-15	-19	0,7	1	0	0	WDR27	33
								10,51
								17,63
-30	3	-1	0	0	0,04	1	PFKFB2	33
-1	10	-1	0	0	0,25	0,86	ZNF215	31
-2	46	-2	0	0	0	0,21	SLC25A26	32
27	10	2	0	0	0,64	0,93	SBDS	33
								25,2
								0,37
								25,6
-12	7	-11	0	0,01	0	0	NBPF20	25
40	10	-45	0	0	0	0	TRIM58	32
4	-16	4	0	0	0,04	0,89	GDF10	36
-31	9	50	0	0	0	0	SMPD1	37
-33	-2	30	0	0	0,21	0,01	AGBL1	1,585
								35
-24	1	2	0	0	0	0	ZNF57	32
-48	0	5	0	0,01	0	0,01	CYP4F22	46
-34	1	4	0	0	0	0	CRYGD	39
8	2	8	0,01	0,45	0	0	ROPN1L	36
-47	3	-47	0	0	0	0	XG	33

GeNET_PN	GeNET_SC	GeNET_dis	g_InFrame_	OutOfFram	isting_uOR	JTR_varian	TR_variant	nomADlof_
-	-	-						5,4225
-	-	-						5,4225
-	-	-						8,7339
-	-	-						1,3453
-	-	-						-1,1787
-	-	-						8,4054
-	-	-						8,4054
-	-	-						4,2122
-	-	-						1,3332
-	-	-						1,3332
-	-	-						2,2512
-	-	-						1,0782
-	-	-						-0,37546
-	-	-						0,067822
-	-	-						2,3866
-	-	-						5,286
-	-	-						
-	-	-						3,9469
-	-	-						1,3275
-	-	-						4,6296
-	-	-						5,221
-	-	-						5,9649
-	-	-						0,27693
-	-	-						7,6947
-	-	-						-6,553
-	-	-						3,9835
-	-	-						4,357
-	-	-						2,4135
-	-	-						-1,4538
-	-	-						5,0102
-	-	-						1,2021
-	-	-						3,9155
-	-	-						0,96755
-	-	-						
-	-	-						3,0528
-	-	-						4,3508
-	-	-						0,61678
-	-	-						0,9329
-	-	-						3,5323
-	-	-						-0,01722
-	-	-						7,9488
-	-	-						1,7197
-	-	-						
-	-	-						2,0094
-	-	-						1,5321
-	-	-						
-	-	-						3,0648
-	-	-						0,74884
-	-	-						4,2786

-	-	-	-0,09812
-	-	-	1,0039
-	-	-	
-	-	-	3,1486
-	-	-	3,163
-	-	-	3,163
-	-	-	2,3215
-	-	-	8,8255
-	-	-	
-	-	-	0,77421
-	-	-	
-	-	-	0,97875
-	-	-	-1,1691
-	-	-	-1,1691
-	-	-	4,8396
-	-	-	0,66649
-	-	-	7,6452
-	-	-	
-	-	-	5,1161
-	-	-	10,187
-	-	-	4,1237
-	-	-	2,2354
-	-	-	4,3925
-	-	-	2,0984
-	-	-	1,3306
-	-	-	1,1299
-	-	-	3,8046
-	-	-	
-	-	-	2,9749
-	-	-	3,714
-	-	-	3,714
-	-	-	0,2068
-	-	-	3,8305
-	-	-	3,7157
-	-	-	7,4513
-	-	-	4,3107
-	-	-	7,1878
-	-	-	6,3029
-	-	-	-0,23066
-	-	-	
-	-	-	0,83083
-	-	-	
-	-	-	3,6202
-	-	-	
-	-	-	1,8264
-	-	-	1,9722
-	-	-	6,299
-	-	-	0,53381
-	-	-	
-	-	-	1,2242

-	-	-	2,5917
-	-	-	
-	-	-	4,6032
-	-	-	0,48781
-	-	-	-0,34194
-	-	-	1,3273
-	-	-	2,0214
-	-	-	
-	-	-	-0,7237
-	-	-	2,8759
-	-	-	
-	-	-	
-	-	-	5,3957
-	-	-	-1,2074
-	-	-	-1,2074
-	-	-	1,1214
-	-	-	0,24139
-	-	-	
-	-	-	1,1909
-	-	-	-0,84985
-	-	-	7,3415
-	-	-	1,1957
-	-	-	-0,37546
-	-	-	
-	-	-	-0,3487
-	-	-	0,95701
-	-	-	5,6226
-	-	-	3,8273
-	-	-	2,9815
-	-	-	0,084563
-	-	-	1,6446
-	-	-	1,4898
-	-	-	3,041
-	-	-	8,5116
-	-	-	-0,5945
-	-	-	0,92859
-	-	-	1,3284
-	-	-	0,85796
-	-	-	3,4815
-	-	-	-0,08862
-	-	-	2,8328
-	-	-	1,0834
-	-	-	1,0511
-	-	-	0,47195
-	-	-	-2,9267
-	-	-	-1,313
-	-	-	0,60465
-	-	-	4,3014
-	-	-	2,7671
-	-	-	0,17059

-	-	-	4,7773
-	-	-	-1,7586
-	-	-	0,65726
-	-	-	3,7116
-	-	-	5,4068
-	-	-	7,513
-	-	-	2,0351
-	-	-	4,5875
-	-	-	4,5875
-	-	-	4,5875
-	-	-	-0,0212
-	-	-	4,0989
-	-	-	5,5428
-	-	-	5,8911
-	-	-	4,2851
-	-	-	1,4606
-	-	-	2,5955
-	-	-	9,9278
-	-	-	4,9203
-	-	-	1,804
-	-	-	0,84553
-	-	-	13,047
-	-	-	3,6036
-	-	-	
-	-	-	0,60486
-	-	-	
-	-	-	-0,5272
-	-	-	0,023628
-	-	-	2,5183
-	-	-	3,2927
-	-	-	5,2021
-	-	-	1,1714
-	-	-	
-	-	-	4,6679
-	-	-	2,9904
-	-	-	6,4309
-	-	-	3,6914
-	-	-	3,5825
-	-	-	
-	-	-	1,545
-	-	-	1,545
-	-	-	1,545
-	-	-	1,545
-	-	-	1,545
-	-	-	1,545
-	-	-	1,545
-	-	-	-0,11816
-	-	-	-1,404
-	-	-	-1,404
-	-	-	-1,404

-	-	-	1,9307
-	-	-	0,53378
-	-	-	2,2202
-	-	-	3,4386
-	-	-	-0,05092
-	-	-	2,058
-	-	-	5,3963
-	-	-	
-	-	-	1,1808
-	-	-	
-	-	-	2,1538
-	-	-	3,8343
-	-	-	1,1785
-	-	-	-0,78467
-	-	-	1,9264
-	-	-	2,3661
-	-	-	-0,5311
-	-	-	0,31188
-	-	-	0,9861
-	-	-	0,9861
-	-	-	2,3926
-	-	-	-0,96262
-	-	-	0,98754
-	-	-	1,4317
-	-	-	7,6334
-	-	-	2,3035
-	-	-	2,3035
-	-	-	3,9623
-	-	-	8,3756
-	-	-	0,22776
-	-	-	0,74154
-	-	-	3,9911
-	-	-	4,9235
-	-	-	
-	-	-	7,1384
-	-	-	7,1384
-	-	-	
-	-	-	4,2706
-	-	-	2,9523
-	-	-	1,7876
-	-	-	-0,53286
-	-	-	-0,53286
-	-	-	5,4017
-	-	-	1,9185
-	-	-	5,9579
-	-	-	2,2831
-	-	-	0,813
-	-	-	-0,95523
-	-	-	1,2967
-	-	-	2,3802

-	-	-	3,9658
-	-	-	1,3218
-	-	-	3,3966
-	-	-	6,7477
-	-	-	0,97165
-	-	-	0,40983
-	-	-	2,7694
-	-	-	2,7064
-	-	-	5,2538
-	-	-	3,3877
-	-	-	1,3453
-	-	-	2,4109
-	-	-	2,8943
-	-	-	1,413
-	-	-	3,0395
-	-	-	-0,03636
-	-	-	5,3415
-	-	-	4,6219
-	-	-	7,0239
-	-	-	5,9535
-	-	-	7,751
-	-	-	6,2374
-	-	-	
-	-	-	0,15826
-	-	-	5,7226
-	-	-	-0,50167
-	-	-	-0,50167
-	-	-	-0,50167
-	-	-	-0,50167
-	-	-	5,3713
-	-	-	5,1343
-	-	-	1,1845
-	-	-	0,91056
-	-	-	0,72714
-	-	-	2,2515
-	-	-	5,8977
-	-	-	4,271
-	-	-	2,0309
-	-	-	2,7321
-	-	-	3,1399
-	-	-	4,7441
-	-	-	2,0488
-	-	-	0,89588
-	-	-	
-	-	-	3,534
-	-	-	
-	-	-	4,8263
-	-	-	2,422
-	-	-	2,6216
-	-	-	

-	-	-	0,206
-	-	-	0,37861
-	-	-	1,1778
-	-	-	-0,39073
-	-	-	0,94458
-	-	-	4,6165
-	-	-	0,68313
-	-	-	8,0868
-	-	-	12,544
-	-	-	-1,2715
-	-	-	
-	-	-	4,9973
-	-	-	0,078459
-	-	-	12,39
-	-	-	-0,17368
-	-	-	2,4133
-	-	-	0,65465
-	-	-	6,6872
-	-	-	1,4868
-	-	-	1,7623
-	-	-	
-	-	-	-0,39699
-	-	-	-1,0641
-	-	-	-1,0641
-	-	-	-1,0641
-	-	-	-0,57474
-	-	-	4,4977
-	-	-	11,578
-	-	-	1,6447
-	-	-	2,6384
-	-	-	3,528
-	-	-	1,565
-	-	-	22,76
-	-	-	7,3477
-	-	-	2,1671
-	-	-	0,27437
-	-	-	0,58368
-	-	-	0,64671
-	-	-	2,4649
-	-	-	
-	-	-	3,3917
-	-	-	0,83106
-	-	-	2,6841
-	-	-	-1,7876
-	-	-	3,7709
-	-	-	1,5365
-	-	-	3,1378
-	-	-	0,78709
-	-	-	-0,34533
-	-	-	7,0613

-	-	-	1,7704
-	-	-	3,0889
-	-	-	1,1111
-	-	-	4,662
-	-	-	2,8267
-	-	-	0,70343
-	-	-	5,0212
-	-	-	3,2192
-	-	-	-0,63407
-	-	-	
-	-	-	3,7698
-	-	-	4,3101
-	-	-	4,1778
-	-	-	
-	-	-	1,8015
-	-	-	0,47081
-	-	-	
-	-	-	0,2759
-	-	-	1,1481
-	-	-	3,0748
-	-	-	1,9225
-	-	-	5,0102
-	-	-	
-	-	-	5,489
-	-	-	4,8015
-	-	-	3,0413
-	-	-	0,30456
-	-	-	0,93602
-	-	-	1,376
-	-	-	
-	-	-	1,2814
-	-	-	-1,6239
-	-	-	1,5554
-	-	-	7,9688
-	-	-	3,0137
-	-	-	1,0411
-	-	-	0,11806
-	-	-	6,7004
-	-	-	4,0204
-	-	-	1,1069
-	-	-	1,0426
-	-	-	1,978
-	-	-	4,2871
-	-	-	-0,05919
-	-	-	1,0047
-	-	-	3,5969
-	-	-	3,5969
-	-	-	0,34894
-	-	-	1,1188
-	-	-	4,6105

-	-	-	
-	-	-	1,002
-	-	-	
-	-	-	7,479
-	-	-	7,479
-	-	-	
-	-	-	0,16163
-	-	-	
-	-	-	-0,95856
-	-	-	-0,08179
-	-	-	0,80502
-	-	-	1,6098
-	-	-	1,6098
-	-	-	1,6098
-	-	-	1,6098
-	-	-	1,6098
-	-	-	1,6098
-	-	-	1,6098
-	-	-	1,6098
-	-	-	1,6098
-	-	-	5,3297
-	-	-	3,9389
-	-	-	1,485
-	-	-	0,85255
-	-	-	-0,0754
-	-	-	4,9897
-	-	-	0,13409
-	-	-	
-	-	-	
-	-	-	5,189
-	-	-	5,5758
-	-	-	12,101
-	-	-	0,69449
-	-	-	3,5744
-	-	-	-0,05623
-	-	-	2,9123
-	-	-	1,7516
-	-	-	2,7983
-	-	-	3,2071
-	-	-	3,3333
-	-	-	-0,08317
-	-	-	7,5501
-	-	-	-1,445
-	-	-	11,286
-	-	-	
-	-	-	6,5876
-	-	-	0,50269
-	-	-	-1,3432
-	-	-	0,39026
-	-	-	8,2763

-	-	-	1,4643
-	-	-	1,4643
-	-	-	2,7822
-	-	-	9,8309
-	-	-	2,4845
-	-	-	6,5828
-	-	-	1,5229
-	-	-	1,6051
-	-	-	-1,1909
-	-	-	-0,08957
-	-	-	1,9697
-	-	-	1,7915
-	-	-	1,7915
-	-	-	2,0419
-	-	-	2,4578
-	-	-	0,25348
-	-	-	1,7695
-	-	-	4,6889
-	-	-	4,3922
-	-	-	0,16989
-	-	-	1,6662
-	-	-	2,2066
-	-	-	6,6164
-	-	-	3,5775
-	-	-	4,5753
-	-	-	2,4194
-	-	-	1,5087
-	-	-	1,5087
-	-	-	1,5087
-	-	-	1,5087
-	-	-	1,5087
-	-	-	1,5087
-	-	-	1,5087
-	-	-	1,5087
-	-	-	1,5087
-	-	-	1,5087
-	-	-	1,5087
-	-	-	1,5087
-	-	-	1,5087
-	-	-	1,5087
-	-	-	1,5087
-	-	-	1,5087
-	-	-	1,5087
-	-	-	1,5087
-	-	-	1,2281
-	-	-	5,697
-	-	-	4,8639
-	-	-	5,7192
-	-	-	6,651
-	-	-	2,3018

-	-	-	2,6366
-	-	-	13,318
-	-	-	2,4238
-	-	-	
-	-	-	1,466
-	-	-	4,5036
-	-	-	5,5149
-	-	-	2,3519
-	-	-	4,4432
-	-	-	1,1101
-	-	-	3,2995
-	-	-	0,34462
-	-	-	-0,79274
-	-	-	5,6381
-	-	-	5,7865
-	-	-	0,48072
-	-	-	3,6739
-	-	-	1,939
-	-	-	5,6991
-	-	-	4,5114
-	-	-	6,5739
-	-	-	-0,20653
-	-	-	4,3361
-	-	-	4,3361
-	-	-	-0,03303
-	-	-	1,4635
-	-	-	7,4774
-	-	-	1,1139
-	-	-	0,92415
-	-	-	1,112
-	-	-	4,441
-	-	-	0,96755
-	-	-	2,5356
-	-	-	0,66161
-	-	-	2,7441
-	-	-	6,0251
-	-	-	1,1548
-	-	-	2,4946
-	-	-	0,97454
-	-	-	3,5587
-	-	-	0,68393
-	-	-	5,5679
-	-	-	1,8934
-	-	-	2,1935
-	-	-	5,1236
-	-	-	2,8567
-	-	-	3,1431
-	-	-	1,3173
-	-	-	1,3949
-	-	-	1,5301

-	-	-	3,1355
-	-	-	1,7295
-	-	-	4,1047
-	-	-	
-	-	-	1,1489
-	-	-	2,6594
-	-	-	2,6594
-	-	-	
-	-	-	2,2303
-	-	-	0,61163
-	-	-	6,3741
-	-	-	4,0189
-	-	-	7,3054
-	-	-	1,7709
-	-	-	0,63675
-	-	-	1,2751
-	-	-	
-	-	-	
-	-	-	
-	-	-	-1,519
-	-	-	
-	-	-	0,94804
-	-	-	2,8688
-	-	-	1,3332
-	-	-	1,3332
-	-	-	1,3332
-	-	-	
-	-	-	1,4151
-	-	-	
-	-	-	4,4091
-	-	-	1,8059
-	-	-	4,5156
-	-	-	5,1651
-	-	-	2,9588
-	-	-	1,8149
-	-	-	2,1221
-	-	-	0,051549
-	-	-	1,3784
-	-	-	3,4202
-	-	-	0,206
-	-	-	0,69365
-	-	-	-2,0515
-	-	-	6,6323
-	-	-	-2,0447
-	-	-	2,8578
-	-	-	1,5087
-	-	-	3,2859
-	-	-	-0,70978
-	-	-	
-	-	-	1,8299

-	-	-	2,179
-	-	-	
-	-	-	
-	-	-	
-	-	-	2,5402
-	-	-	0,19251
-	-	-	0,86995
-	-	-	1,0189
-	-	-	
-	-	-	
-	-	-	
-	-	-	-3,0807
-	-	-	-0,07824
-	-	-	1,9981
-	-	-	1,2349
-	-	-	0,68119
-	-	-	
-	-	-	0,68684
-	-	-	2,4174
-	-	-	0,58368
-	-	-	0,29462
-	-	-	-0,15014
-	-	-	2,6355

nomADmis	omADoe_	ADoe_lof_	omADoe_r	ADoe_mis_	omADoe_s	ADoe_syn_	gnomADpL	nomADsyn_
1,7621	0,074967	0,194	0,81231	0,871	0,90859	1,004	1	1,2455
1,7621	0,074967	0,194	0,81231	0,871	0,90859	1,004	1	1,2455
6,5427	0,21503	0,29	0,52127	0,553	0,93806	1,01	0,9	1,1207
0,49195	0,76431	1,042	0,92271	1,016	0,99287	1,153	0	0,062417
0,15241	1,4407	1,916	0,96937	1,093	1,0164	1,21	0	-0,12223
2,5854	0,044396	0,102	0,79124	0,835	1,0068	1,084	1	-0,11981
2,5854	0,044396	0,102	0,79124	0,835	1,0068	1,084	1	-0,11981
1,6679	0,22972	0,414	0,81585	0,877	0,73286	0,826	0,32	3,4078
0,19299	0,53111	1,116	0,96856	1,068	1,0699	1,223	0	-0,65929
0,19299	0,53111	1,116	0,96856	1,068	1,0699	1,223	0	-0,65929
0,17903	0,21097	0,664	0,96839	1,076	1,1559	1,335	0,41	-1,3147
0,65657	0,44881	1,376	0,83522	0,982	1,1693	1,44	0,08	-0,98474
1,1974	1,0537	1,306	0,87952	0,937	0,76271	0,848	0	3,3294
-0,3227	0,92944	1,886	1,0945	1,291	0,94765	1,251	0,1	0,25363
-0,37565	0,60076	0,839	1,0517	1,138	0,95303	1,1	0	0,43782
2,1239	0,13725	0,271	0,71214	0,783	0,98006	1,121	1	0,19521
1,5438	0,091078	0,287	0,73913	0,83	0,92275	1,092	0,99	0,62264
-3,6562	0,84482	1,028	1,3144	1,373	1,2852	1,375	0	-4,8561
0,77372	0,20339	0,367	0,95452	0,989	0,94649	0,997	0,72	1,3787
0,84885	0,056129	0,177	0,87739	0,961	1,2001	1,364	1	-1,8552
2,5346	0,12835	0,241	0,67251	0,738	1,0607	1,188	1	-0,67772
-0,10458	0,89978	1,591	1,0291	1,21	0,98284	1,288	0	0,083895
5,054	0,12277	0,203	0,52684	0,568	1,2923	1,4	1	-4,1914
-12,407	2,0068	1,988	2,6629	1,999	2,0632	1,995	0	-10,978
2,409	0,18134	0,381	0,55401	0,642	0,88153	1,073	0,82	0,84162
2,1853	0	0,135	0,67299	0,749	0,92207	1,07	1	0,7104
0,84329	0	0,441	0,72064	0,908	1,3399	1,749	0,87	-1,4039
-1,6461	1,598	1,943	1,3286	1,471	1,4285	1,654	0	-3,1886
3,0165	0,0604	0,19	0,61792	0,679	0,78654	0,889	1	2,5525
-0,93437	0,71648	1,103	1,1659	1,284	1,3444	1,548	0	-2,7429
1,6265	0,20982	0,414	0,73984	0,825	0,95059	1,115	0,55	0,41404
-0,16031	0	1,696	1,0361	1,18	0,99373	1,218	0,4	0,040477
0,44421	0,54175	0,743	0,9458	1,018	0,85152	0,967	0	1,654
0,94113	0,18208	0,359	0,8625	0,946	0,9706	1,106	0,86	0,29858
0,070367	0	1,844	0,89202	1,814	1,7724	1,935	0,33	-0,64499
-0,0775	0,82412	1,136	1,011	1,099	0,94557	1,076	0	0,56524
2,4867	0,18352	0,42	0,56369	0,647	0,81161	0,971	0,73	1,5244
0,97414	1,0027	1,272	0,89434	0,957	0,85123	0,945	0	2,0001
2,9693	0,04915	0,112	0,71313	0,763	0,99639	1,096	1	0,049352
-0,32611	0,5606	0,951	1,0629	1,187	1,0652	1,261	0	-0,48879
0,7935	0,45012	0,845	0,83504	0,955	0,9486	1,157	0	0,34707
1,2193	0,54054	1,015	0,76305	0,87	0,77434	0,952	0	1,6252
0,83056	0,077753	0,369	0,85087	0,954	0,97837	1,151	0,93	0,17533
-1,0735	0,85358	1,184	1,1694	1,274	1,1285	1,302	0	-1,1046
1,712	0,40925	0,572	0,81883	0,877	0,94391	1,048	0	0,71762

-1,2077	1,0253	1,517	1,2168	1,339	0,97829	1,177	0	0,15525
0,65932	0,7413	1,179	0,87094	0,986	0,67078	0,851	0	2,2119
1,0075	0,458	0,679	0,83924	0,93	1,0278	1,194	0	-0,23867
0,38197	0,52433	0,723	0,95218	1,027	0,76802	0,886	0	2,4086
0,38197	0,52433	0,723	0,95218	1,027	0,76802	0,886	0	2,4086
0,80651	0,68982	0,884	0,90866	0,974	1,023	1,139	0	-0,27545
3,5024	0,058619	0,116	0,73313	0,772	1,0248	1,102	1	-0,43843
0,49015	0	1,797	0,68368	1,087	0,78463	1,591	0,36	0,42736
0,079961	0,85735	1,093	0,99132	1,057	0,97371	1,079	0	0,33708
-11,532	1,8604	1,948	2,0273	1,998	1,7082	1,817	0	-11,429
-11,532	1,8604	1,948	2,0273	1,998	1,7082	1,817	0	-11,429
0,76926	0,0909	0,235	0,89166	0,973	1,0359	1,176	1	-0,36229
0,75366	0,776	1,395	0,8066	0,955	0,79765	1,042	0	1,1122
1,6298	0,13263	0,215	0,8488	0,9	0,85216	0,937	1	2,1878
0,46164	0,43277	0,562	0,96133	1,011	1,0112	1,097	0	-0,17761
2,4949	0,071353	0,121	0,81366	0,854	1,057	1,136	1	-1,0019
1,3587	0	0,151	0,76321	0,858	0,99395	1,173	1	0,047971
0,56823	0,58679	0,853	0,91348	1,003	0,95581	1,117	0	0,37934
-0,73627	0,63219	0,743	1,0495	1,091	1,1738	1,25	0	-3,2926
0,22489	0,79014	0,94	0,98544	1,024	1,0544	1,118	0	-1,1675
1,0965	0,66259	1,074	0,7897	0,897	0,98393	1,175	0	0,11949
0,47883	0,75667	1,11	0,92305	1,019	0,82236	0,971	0	1,5398
1,6041	0,21779	0,43	0,74977	0,834	1,0029	1,169	0,46	-0,02509
1,1981	0,081804	0,388	0,78095	0,882	0,99936	1,181	0,91	0,00502
-1,1449	0,79291	0,873	1,0443	1,068	1,0519	1,087	0	-1,985
-1,1449	0,79291	0,873	1,0443	1,068	1,0519	1,087	0	-1,985
-1,2412	0,95871	1,314	1,2027	1,313	1,19	1,371	0	-1,6024
1,9591	0,052535	0,249	0,69979	0,779	1,074	1,226	0,99	-0,70549
3,2832	0,13848	0,358	0,36218	0,438	0,94684	1,155	0,93	0,35934
0,95534	0,11982	0,203	0,91729	0,967	1,1301	1,222	1	-2,0334
2,5125	0,27051	0,448	0,65495	0,724	0,95807	1,106	0,03	0,38984
0,88511	0,2623	0,358	0,93069	0,976	1,034	1,109	0	-0,61923
2,6729	0,039853	0,125	0,64945	0,715	0,92903	1,058	1	0,73673
-2,826	1,0309	1,263	1,3286	1,41	1,2011	1,335	0	-2,2668
-0,14331	0,75383	1,278	1,0279	1,151	0,97606	1,161	0	0,18274
0,77253	0,10497	0,33	0,85169	0,963	1,1023	1,312	0,96	-0,73069
0,35347	0,52428	0,915	0,93757	1,044	0,90482	1,084	0	0,72518
0,058494	0,4565	0,857	0,99011	1,094	0,82881	0,972	0	1,5432
1,381	0,35315	0,461	0,8876	0,934	0,96362	1,042	0	0,61316
-0,76021	0,86598	1,315	1,1314	1,245	1,2289	1,433	0	-1,7557
0,061856	0,82981	1,05	0,99302	1,061	1,0063	1,118	0	-0,0785

1,377	0,30699	0,645	0,70023	0,816	0,92565	1,142	0,06	0,48215
1,3523	0,36997	0,524	0,84411	0,909	1,0133	1,134	0	-0,15383
0,35581	0,90369	1,246	0,94036	1,041	1,1555	1,337	0	-1,2962
-0,62323	1,1237	1,783	1,1462	1,304	1,1535	1,393	0	-0,99198
0,46558	0,70032	1,064	0,91909	1,023	0,91329	1,08	0	0,70602
1,1463	0,59269	0,889	0,83749	0,917	1,0678	1,206	0	-0,70572
-0,6693	1,3737	1,916	1,1506	1,302	1,1009	1,334	0	-0,65454
-0,734	0,41963	0,68	1,0945	1,177	1,1693	1,296	0	-1,9772
-1,5642	0,24932	0,384	1,135	1,19	1,2532	1,341	0,06	-4,344
-0,37864	1,5056	1,931	1,1035	1,287	1,0962	1,375	0	-0,53053
-0,37864	1,5056	1,931	1,1035	1,287	1,0962	1,375	0	-0,53053
-0,29233	0,73978	1,124	1,0517	1,164	1,3487	1,561	0	-2,681
-1,5042	0,89916	1,681	1,3116	1,459	1,3069	1,554	0	-2,0242
-1,0651	0,41476	1,286	1,2349	1,388	1,2911	1,551	0,1	-1,8239
-1,9863	1,3174	1,883	1,4349	1,598	1,2609	1,52	0	-1,6234
2,4375	0,056706	0,13	0,73421	0,791	0,95111	1,063	1	0,58579
1,647	0,53021	1,21	0,71303	0,805	0,90134	1,066	0,01	0,80872
1,1974	1,0537	1,306	0,87952	0,937	0,76271	0,848	0	3,3294
-3,6846	1,183	1,881	1,8041	1,954	1,731	1,949	0	-4,4967
-0,62791	0,48922	1,471	1,139	1,287	1,1096	1,352	0,07	-0,68928
0,95608	0,10805	0,227	0,86704	0,947	1,3142	1,476	1	-3,0694
2,4584	0,30842	0,511	0,72145	0,78	0,85191	0,949	0	1,9417
1,2144	0,2912	0,575	0,8409	0,915	0,95887	1,079	0,06	0,46458
0,48089	0,98028	1,411	0,9157	1,02	0,83185	0,981	0	1,4637
0,15597	0,69212	0,981	0,97631	1,069	1,0031	1,151	0	-0,02899
0,92692	0	1,119	0,72096	0,889	0,88284	1,175	0,57	0,57155
0,94765	0,42692	0,667	0,8708	0,949	1,0037	1,133	0	-0,03935
2,7323	0,10454	0,173	0,81092	0,848	0,88371	0,945	1	2,384
0,97932	1,2245	1,849	0,73981	0,888	0,96465	1,23	0	0,19394
0,011781	0,79963	1,162	0,9984	1,082	0,97682	1,104	0	0,25012
1,4267	0,63793	1,082	0,72609	0,829	0,84559	1,025	0	1,1432
0,96015	0,58956	1,476	0,79427	0,915	0,66854	0,862	0,02	2,0896
0,60829	0,53534	0,71	0,93914	0,998	0,90316	0,996	0	1,3546
0,17587	1,049	1,853	0,92732	1,196	0,92139	1,417	0	0,24931
1,5827	0,30771	0,607	0,6905	0,793	0,87734	1,07	0,04	0,8613
0,018649	0,59626	1,251	0,99535	1,153	0,94024	1,202	0	0,32859
0,90226	0,73934	1,156	0,84985	0,945	0,96787	1,126	0	0,28122
-0,02652	0,85139	1,439	1,0073	1,186	0,97139	1,299	0	0,1311
-3,215	2,6151	1,977	1,7916	1,955	1,3192	1,635	0	-1,692
-4,0257	1,4777	1,923	2,1231	1,995	1,9805	1,985	0	-4,6471
-0,97262	0,9211	1,136	1,1045	1,173	1,0144	1,127	0	-0,17695
2,1432	0,28484	0,462	0,69648	0,77	0,93643	1,086	0,01	0,57966
2,3537	0,45919	0,718	0,66857	0,74	0,9519	1,099	0	0,44696
0,55562	0,96063	1,383	0,90497	1,006	0,9813	1,141	0	0,1632

0,74066	0,21087	0,368	0,92187	0,983	0,98798	1,086	0,63	0,16657
-0,29522	1,4817	1,906	1,0509	1,16	1,1656	1,342	0	-1,4216
-2,2339	0,83771	1,272	1,3559	1,469	1,1893	1,359	0	-1,6987
1,3904	0,51906	0,685	0,8561	0,914	1,0464	1,153	0	-0,60789
2,4144	0,16417	0,296	0,69172	0,757	0,98132	1,107	0,99	0,20432
3,2597	0,066311	0,139	0,63673	0,691	1,0432	1,164	1	-0,50479
-1,086	0,76679	0,938	1,1083	1,172	1,0727	1,181	0	-0,95258
0,96617	0,38536	0,538	0,90012	0,96	0,90529	1,007	0	1,2122
0,96617	0,38536	0,538	0,90012	0,96	0,90529	1,007	0	1,2122
0,96617	0,38536	0,538	0,90012	0,96	0,90529	1,007	0	1,2122
0,25513	1,0032	1,27	0,97104	1,039	1,0005	1,108	0	-0,00639
1,393	0,2185	0,41	0,80106	0,88	0,9779	1,125	0,46	0,20861
1,5457	0,5929	0,687	0,91272	0,945	1,0001	1,054	0	-0,00258
1,8276	0,17032	0,289	0,81007	0,867	0,96217	1,056	0,99	0,53718
1,8312	0,20624	0,387	0,79742	0,858	0,93232	1,03	0,64	0,91367
-0,05534	0,57677	1,041	1,0112	1,139	1,0995	1,34	0	-0,62827
0,83382	0,59785	0,815	0,90458	0,971	0,97122	1,082	0	0,35194
4,1944	0,15079	0,212	0,70728	0,743	1,0544	1,124	1	-1,0715
-1,2894	0,51305	0,635	1,0889	1,132	0,98882	1,059	0	0,21405
0,071521	0,72065	0,955	0,99031	1,073	1,197	1,356	0	-1,8776
-0,12335	0,78407	1,226	1,0234	1,143	0,90676	1,097	0	0,67103
6,0451	0,077305	0,115	0,65271	0,68	0,99937	1,058	1	0,014441
1,7141	0,29543	0,516	0,70337	0,794	0,89007	1,066	0,02	0,84449
-4,3356			2,2893	1,996	1,9805	1,984		-4,3816
-0,12554	0,76667	1,492	1,0354	1,22	1,1393	1,488	0	-0,64071
-0,03183	1,2889	1,905	1,0095	1,204	1,271	1,642	0	-1,2247
0,12361	0,98213	1,874	0,9636	1,15	0,83109	1,127	0,03	0,79774
0,62071	0,46503	0,753	0,88164	0,994	1,0408	1,241	0	-0,29647
1,776	0,20183	0,462	0,74569	0,822	1,0465	1,198	0,56	-0,43882
2,3445	0,056495	0,178	0,64133	0,718	0,94952	1,115	1	0,42123
0,617	0,68464	1,133	0,88638	0,995	0,8567	1,042	0	1,0327
1,2655	0,24627	0,408	0,86211	0,924	0,87123	0,972	0,12	1,6482
0,16787	0,57429	0,768	0,98137	1,048	0,99972	1,109	0	0,003545
1,1249	0,32809	0,435	0,90405	0,953	1,0233	1,111	0	-0,366
2,2364	0,1017	0,32	0,67547	0,749	0,91364	1,041	0,97	0,904
2,69	0,31444	0,533	0,7403	0,791	0,85417	0,936	0,01	2,2326
-12,234	0,53784	1,01	1,6192	1,657	1,769	1,83	0	-22,182
-12,234	0,53784	1,01	1,6192	1,657	1,769	1,83	0	-22,182
-12,234	0,53784	1,01	1,6192	1,657	1,769	1,83	0	-22,182
-12,234	0,53784	1,01	1,6192	1,657	1,769	1,83	0	-22,182
-12,234	0,53784	1,01	1,6192	1,657	1,769	1,83	0	-22,182
-12,234	0,53784	1,01	1,6192	1,657	1,769	1,83	0	-22,182
-12,234	0,53784	1,01	1,6192	1,657	1,769	1,83	0	-22,182
-2,2696	1,0297	1,508	1,5072	1,678	1,3951	1,669	0	-2,4384
-0,86995	1,4751	1,919	1,2596	1,474	1,4892	1,845	0	-2,2725
-0,86995	1,4751	1,919	1,2596	1,474	1,4892	1,845	0	-2,2725
-0,86995	1,4751	1,919	1,2596	1,474	1,4892	1,845	0	-2,2725

-1,5302	0,70442	0,934	1,1737	1,248	1,2822	1,41	0	-3,4272
-0,11208	0,8845	1,263	1,0195	1,128	1,0101	1,196	0	-0,0775
0,46165	0,58102	0,853	0,92997	1,02	1,0233	1,183	0	-0,20758
0,92885	0,29157	0,526	0,83828	0,938	0,902	1,088	0,03	0,72097
-2,015	1,0069	1,242	1,2102	1,279	1,0638	1,167	0	-0,87003
-0,24831	0,50227	0,852	1,0468	1,167	1,3394	1,567	0	-2,4396
-1,2857	0,57791	0,677	1,0844	1,125	1,1273	1,199	0	-2,5328
-1,1057	0,59779	1,179	1,2852	1,47	1,0822	1,355	0	-0,4608
0,1314	0,7161	0,911	0,98871	1,04	1,0236	1,105	0	-0,3946
0,95608	0,33539	0,533	0,8784	0,951	1,1114	1,238	0	-1,2757
-0,45384	0,67065	1,138	1,068	1,163	1,1802	1,327	0	-1,8394
-0,92583	1,0917	1,297	1,0778	1,13	1,2727	1,358	0	-4,8415
-0,36679	0,67202	0,927	1,0443	1,119	1,1553	1,272	0	-1,9472
0,78468	0,33706	0,709	0,86151	0,964	1,378	1,574	0,04	-3,1614
-0,6865	1,1299	1,607	1,1177	1,23	1,3066	1,496	0	-2,5821
0,30983	0,87171	1,648	0,93217	1,066	1,1991	1,444	0	-1,2785
-1,7163	0,80862	1,129	1,228	1,318	1,2265	1,364	0	-2,501
-1,7163	0,80862	1,129	1,228	1,318	1,2265	1,364	0	-2,501
0,43798	0	0,449	0,88774	1,042	0,85029	1,096	0,87	0,83686
-1,9492	1,2209	1,674	1,3105	1,422	1,2492	1,418	0	-2,2916
-0,98208	0,54555	1,385	1,3246	1,57	1,2557	1,66	0,02	-1,061
0,51721	0,77073	1,022	0,93399	1,009	1,1061	1,236	0	-1,1867
1,4389	0,2169	0,305	0,87903	0,927	1,1679	1,253	0,77	-2,8879
-0,3959	0,49508	0,802	1,0621	1,162	1,1342	1,299	0	-1,2092
-0,3959	0,49508	0,802	1,0621	1,162	1,1342	1,299	0	-1,2092
0,029906	0,69069	0,798	0,99823	1,033	1,0908	1,15	0	-2,1274
1,1316	0,091024	0,159	0,90193	0,952	1,0464	1,137	1	-0,71086
-0,12928	0,78257	1,864	1,1992	1,893	2,0575	1,941	0,11	-0,81956
0,57456	0,63255	1,555	0,91734	1,002	0,88153	1,028	0,02	1,0729
1,1577	0,20462	0,404	0,84437	0,92	0,85563	0,984	0,62	1,4517
0,72174	0,062296	0,196	0,92546	0,986	1,0359	1,135	1	-0,50397
0,81019	0,41509	0,505	0,95018	0,986	1,043	1,104	0	-0,96557
0,81019	0,41509	0,505	0,95018	0,986	1,043	1,104	0	-0,96557
2,6725	0,45217	0,607	0,73231	0,784	0,98629	1,083	0	0,19162
-0,10744	0,31943	0,6	1,0175	1,119	1,311	1,488	0,02	-2,8005
-1,076	0,46372	0,915	1,219	1,359	1,6021	1,856	0	-4,0617
-3,9471	1,1088	1,496	1,6244	1,747	1,789	1,952	0	-7,2286
-3,9471	1,1088	1,496	1,6244	1,747	1,789	1,952	0	-7,2286
-3,4333	0,46437	0,581	1,2472	1,295	1,5858	1,667	0	-12,05
-0,25621	0,37018	0,847	1,0453	1,157	1,3847	1,578	0,04	-3,2607
1,8383	0,16761	0,284	0,82243	0,876	1,2468	1,343	0,99	-3,8618
0,23573	0,679	0,884	0,97481	1,039	0,93989	1,04	0	0,79936
1,0221	0,81349	1,206	0,815	0,917	0,98262	1,164	0	0,13507
-0,92501	1,2236	1,686	1,1644	1,283	1,0656	1,248	0	-0,52648
0,13224	0,66947	1,085	0,97602	1,087	0,9681	1,149	0	0,24713
0,63821	0,4694	0,777	0,89446	0,991	1,1457	1,314	0	-1,2931

0,50343	0,46063	0,628	0,94575	1,009	1,0028	1,106	0	-0,03759
0,42005	0,49713	1,136	0,92078	1,034	0,99114	1,168	0,01	0,070621
1,1825	0,54331	0,72	0,86587	0,93	0,96129	1,074	0	0,46556
4,0418	0,18699	0,288	0,58795	0,636	0,86485	0,967	0,99	1,6983
-0,24408	0,55074	1,397	1,0658	1,244	0,91757	1,217	0,02	0,40018
-0,61112	0	1,879	1,1844	1,395	1,8123	1,971	0,3	-3,6124
1,6896	0,48153	0,731	0,7744	0,847	0,80454	0,913	0	2,24
1,7292	0,44046	0,714	0,70761	0,797	0,90792	1,071	0	0,76347
3,1977	0,13853	0,273	0,54336	0,609	0,92167	1,067	1	0,7285
2,3469	0,15883	0,411	0,64854	0,724	0,94697	1,104	0,82	0,4634
0,49195	0,76431	1,042	0,92271	1,016	0,99287	1,153	0	0,062417
0,038095	0,62459	0,847	0,99449	1,083	1,1469	1,304	0	-1,3978
1,6104	0,47687	0,715	0,81988	0,882	0,94809	1,049	0	0,68609
0,5203	0,56629	1,064	0,87913	1,018	0,78018	1	0	1,3123
0,69948	0	0,278	0,87076	0,978	0,87007	1,031	0,97	1,0729
-0,11388	1,009	1,479	1,0174	1,112	1,191	1,35	0	-1,8199
0,43144	0,053881	0,17	0,94097	1,022	0,98029	1,126	1	0,18776
1,5575	0,3686	0,522	0,80464	0,874	0,99949	1,13	0	0,005427
0,60715	0,11024	0,199	0,94973	0,998	1,0075	1,089	1	-0,12454
1,7489	0,14251	0,257	0,82948	0,883	1,0171	1,109	1	-0,25584
3,8358	0,093883	0,169	0,67472	0,717	1,0098	1,091	1	-0,16484
0,75411	0,11972	0,225	0,93557	0,985	1,1366	1,217	1	-2,4311
-0,00888	0,97302	1,271	1,0011	1,074	1,0285	1,139	0	-0,3592
1,8498	0,02493	0,118	0,78456	0,848	1,073	1,187	1	-0,90912
-1,6767	1,1223	1,597	1,17	1,236	1,5095	1,623	0	-7,4477
-1,6767	1,1223	1,597	1,17	1,236	1,5095	1,623	0	-7,4477
-1,6767	1,1223	1,597	1,17	1,236	1,5095	1,623	0	-7,4477
-1,6767	1,1223	1,597	1,17	1,236	1,5095	1,623	0	-7,4477
0,42807	0,11623	0,244	0,95221	1,018	1,3536	1,47	1	-4,8019
2,6556	0,0823	0,213	0,6553	0,72	0,90849	1,022	1	1,062
0,9158	0,69285	1,123	0,81898	0,932	1,0088	1,202	0	-0,06523
-1,7655	0,80733	1,163	1,2153	1,297	1,3558	1,487	0	-4,3031
-0,14193	0,80476	1,279	1,0289	1,158	0,94393	1,13	0	0,41828
0,40019	0,31653	0,724	0,91972	1,04	0,92351	1,112	0,08	0,55966
1,9019	0,13062	0,245	0,77001	0,835	0,93806	1,049	1	0,7457
0,16704	0,53635	0,674	0,98589	1,036	0,9876	1,066	0	0,21281
0,90052	0,46922	0,847	0,80995	0,93	1,011	1,215	0	-0,078
1,78	0,53112	0,765	0,76513	0,836	0,9481	1,074	0	0,559
0,285	0,17698	0,457	0,94182	1,066	1,1364	1,341	0,69	-1,0108
1,2098	0,11857	0,271	0,82979	0,909	0,96708	1,104	1	0,32867
0,98109	0	0,612	0,82251	0,925	0,93098	1,12	0,77	0,5061
-1,2492	0,39352	1,606	1,2074	1,32	1,2495	1,451	0,18	-1,9457
0,40824	0,52087	0,696	0,95745	1,019	1,0734	1,173	0	-1,0356
2,6339	0,22272	0,378	0,66762	0,731	1,0383	1,171	0,43	-0,40633
2,0137	0,19141	0,603	0,59437	0,693	0,74127	0,907	0,51	1,9479
1,8576	0,26788	0,613	0,71572	0,796	0,93502	1,072	0,17	0,64046

-0,62541	0,85468	1,84	1,1965	1,418	1,3297	1,684	0,03	-1,5735
-0,85903	0,74983	1,793	1,2377	1,433	1,2066	1,51	0,04	-1,0965
0,99304	0,76778	1,088	0,86317	0,942	0,93071	1,065	0	0,69376
-0,12103	1,2342	1,9	1,015	1,091	1,0704	1,21	0	-0,72582
1,5434	0,84775	1,111	0,85482	0,907	0,9613	1,049	0	0,59041
2,2079	0,36899	0,523	0,79765	0,847	1,1047	1,193	0	-1,6889
-0,05541	0,8779	1,179	1,0066	1,08	1,0504	1,179	0	-0,55315
4,3365	0,17132	0,251	0,66954	0,707	1,0508	1,126	1	-0,93019
3,2081	0,066571	0,104	0,81599	0,847	1,0801	1,14	1	-1,8672
-1,7733	1,2323	1,588	1,2465	1,341	1,1142	1,259	0	-1,1569
-1,9281	0,39289	0,53	1,2032	1,273	1,1657	1,28	0	-2,1277
-0,57371	0,97358	1,621	1,1408	1,306	1,2796	1,586	0	-1,505
2,3403	0,054952	0,091	0,84442	0,881	1,2699	1,347	1	-5,2715
-0,0672	1,0408	1,485	1,0112	1,114	1,0111	1,184	0	-0,09151
0,5856	0,44848	0,761	0,89304	1,001	0,98469	1,17	0	0,11699
0,003915	0,89309	1,167	0,99943	1,088	1,1647	1,331	0	-1,489
3,5535	0,48281	0,573	0,77488	0,808	0,8114	0,87	0	3,8913
0,088127	0,63184	1,024	0,9851	1,089	1,0501	1,22	0	-0,42625
-0,12926	0,43746	0,92	1,0317	1,189	1,1496	1,439	0,01	-0,81337
-0,0368	1,0843	1,486	1,0052	1,091	1,0388	1,174	0	-0,4047
-2,6615	1,4113	1,912	1,4996	1,641	1,4907	1,725	0	-3,5884
-2,6615	1,4113	1,912	1,4996	1,641	1,4907	1,725	0	-3,5884
-2,6615	1,4113	1,912	1,4996	1,641	1,4907	1,725	0	-3,5884
0,35882	1,1958	1,81	0,94195	1,039	1,014	1,165	0	-0,1308
1,1824	0,37787	0,535	0,85345	0,923	0,97493	1,104	0	0,26554
-0,04085	0,36939	0,424	1,0019	1,029	1,0126	1,059	0	-0,36419
0,47793	0,70726	0,981	0,9389	1,015	1,0365	1,172	0	-0,38023
-0,40884	0,67797	0,852	1,0437	1,11	1,0759	1,193	0	-0,92259
0,65719	0,28341	0,511	0,87484	0,986	1,0612	1,265	0,04	-0,44331
-0,61275	0,62862	0,999	1,0931	1,191	1,1413	1,3	0	-1,3234
-1,1021	0,3268	0,354	1,0233	1,036	1,0026	1,023	0	-0,16611
3,3079	0,42538	0,512	0,83001	0,858	0,85444	0,902	0	3,7749
0,32688	0,32915	0,753	0,91748	1,072	1,0638	1,343	0,07	-0,3472
-0,39378	0,94651	1,291	1,0632	1,165	1,0737	1,249	0	-0,61492
0,080754	0,7738	1,504	0,97817	1,149	1,1001	1,399	0	-0,51969
1,6158	0,85886	1,237	0,70681	0,803	1,0631	1,255	0	-0,48082
0,50895	0,65866	0,857	0,9434	1,009	0,94477	1,049	0	0,70766
1,5058	0,065226	0,309	0,75782	0,845	0,97383	1,127	0,97	0,2368
0,60095	0,72923	1,314	0,90627	0,998	0,86632	0,998	0	1,3215
1,1016	0,63504	0,827	0,88704	0,946	0,89797	0,996	0	1,3569
-2,2035	1,8822	1,96	1,6761	1,905	1,7454	1,96	0	-3,5204
2,2818	0,19545	0,411	0,65862	0,734	0,916	1,058	0,68	0,79564
0,40541	0,67817	1,006	0,93727	1,03	1,1394	1,305	0	-1,2527
1,3687	0,37489	0,621	0,80499	0,884	1,0128	1,155	0	-0,12629
-1,2606	0,55326	1,593	1,2059	1,316	1,1752	1,363	0,06	-1,4202
1,2168	1,0976	1,649	0,79173	0,887	1,0237	1,195	0	-0,19709
2,5616	0,15852	0,252	0,77832	0,824	1,035	1,123	1	-0,54509

0,31772	0,51517	0,93	0,94597	1,049	1,0236	1,178	0	-0,21599
0,426	0,32655	0,589	0,93724	1,025	1,014	1,152	0,01	-0,14127
0,37964	0,67228	1,173	0,90925	1,054	0,86293	1,11	0	0,7694
-0,45989	0,12186	0,279	1,0538	1,127	1,0922	1,215	0,99	-1,0743
0,35005	0,51999	0,749	0,95189	1,034	0,86456	1,002	0	1,2852
-0,17133	0,64737	1,579	1,0419	1,2	0,95016	1,2	0,02	0,28706
2,0908	0,45883	0,587	0,83014	0,875	0,8475	0,916	0	2,7624
0,82836	0,071445	0,339	0,82028	0,945	0,96796	1,186	0,95	0,20952
-0,61623	1,4807	1,927	1,1923	1,411	1,29	1,639	0	-1,3905
1,8177	0,22036	0,435	0,71986	0,801	0,95206	1,102	0,43	0,43862
1,489	0,16222	0,341	0,80341	0,876	0,99304	1,111	0,94	0,080874
0,325	0,31921	0,499	0,96172	1,032	1,1074	1,225	0	-1,3213
1,129	0,50941	0,919	0,77402	0,885	1,1087	1,308	0	-0,81554
0,10302	0,89266	1,297	0,98135	1,093	0,92182	1,09	0	0,63363
1,0216	0,92076	1,46	0,82583	0,922	0,77611	0,921	0	1,9369
0,064942	0,76372	1,1	0,98962	1,088	1,0896	1,255	0	-0,79223
0,73536	0,1357	0,427	0,86038	0,971	1,4636	1,685	0,84	-3,5515
0,44822	0,5074	0,885	0,90279	1,033	0,82327	1,023	0	1,1761
3,0165	0,0604	0,19	0,61792	0,679	0,78654	0,889	1	2,5525
1,5985	0,20043	0,332	0,81756	0,881	1,0089	1,125	0,86	-0,10566
1,9718	0,3322	0,483	0,79074	0,848	0,99686	1,098	0	0,042297
1,457	0,13797	0,434	0,69638	0,807	0,81815	1,013	0,82	1,2343
-0,18615	0,90569	1,492	1,0419	1,186	1,0617	1,303	0	-0,38265
0,83941	0,82646	1,133	0,88017	0,963	0,91744	1,057	0	0,79305
1,0779	0,72423	1,043	0,82723	0,918	0,88825	1,034	0	1,0211
0,64491	0,27494	1,27	0,79881	0,982	1,4215	1,759	0,27	-2,1344
-4,1868	1,3378	1,751	1,6356	1,754	1,6274	1,805	0	-6,1736
0,94846	0,57554	1,004	0,81896	0,927	1,054	1,25	0	-0,40264
1,2028	0,16835	0,25	0,92189	0,959	1,1525	1,214	1	-3,5366
1,7133	0,48201	0,707	0,73347	0,816	1,0062	1,166	0	-0,05484
0,34546	0,83555	1,091	0,95682	1,031	0,94071	1,06	0	0,66584
0,21039	0,97839	1,303	0,96634	1,063	0,98858	1,153	0	0,097238
2,7504	0,20533	0,308	0,78016	0,823	0,98079	1,056	0,91	0,34201
1,0942	0,33194	0,519	0,87333	0,939	1,0901	1,204	0	-1,1351
0,61824	0,72768	1,138	0,89625	0,995	0,82005	0,992	0	1,3618
0,16876	0,8488	1,082	0,98001	1,051	0,94818	1,055	0	0,6494
0,61761	0,6546	0,914	0,91777	0,996	1,0426	1,172	0	-0,46387
0,77479	0,51194	0,655	0,93319	0,983	0,96012	1,045	0	0,62771
0,68247	1,0086	1,263	0,93421	0,991	0,90544	0,992	0	1,4104
0,33781	0,71092	1,206	0,93286	1,053	0,96387	1,167	0	0,25216
1,0904	0,68249	0,805	0,92331	0,964	1,0897	1,159	0	-1,8214
1,0904	0,68249	0,805	0,92331	0,964	1,0897	1,159	0	-1,8214
0,28302	0,93357	1,266	0,95286	1,054	1,1912	1,388	0	-1,4958
0,61784	0,74692	1,12	0,88438	0,994	0,9088	1,104	0	0,64249
-1,1762	0,26318	0,426	1,1313	1,203	1,4211	1,545	0,04	-5,5116

-0,09878	0,89556	1,061	1,0088	1,063	0,91222	0,999	0	1,3124
1,2901	0,33039	0,42	0,90747	0,948	0,97068	1,041	0	0,55055
1,2901	0,33039	0,42	0,90747	0,948	0,97068	1,041	0	0,55055
0,39185	0,96813	1,32	0,9463	1,028	1,0669	1,208	0	-0,67931
0,33633	1,2075	1,633	0,93938	1,048	0,99498	1,186	0	0,037546
0,82317	1,0248	1,611	0,79178	0,937	0,82921	1,095	0	0,88459
0,74012	0,81513	1,209	0,86623	0,971	1,0074	1,197	0	-0,05594
-18,875	0,83536	0,993	2,0267	1,999	1,7611	1,826	0	-20,511
-18,875	0,83536	0,993	2,0267	1,999	1,7611	1,826	0	-20,511
-18,875	0,83536	0,993	2,0267	1,999	1,7611	1,826	0	-20,511
-18,875	0,83536	0,993	2,0267	1,999	1,7611	1,826	0	-20,511
-18,875	0,83536	0,993	2,0267	1,999	1,7611	1,826	0	-20,511
-18,875	0,83536	0,993	2,0267	1,999	1,7611	1,826	0	-20,511
-18,875	0,83536	0,993	2,0267	1,999	1,7611	1,826	0	-20,511
-18,875	0,83536	0,993	2,0267	1,999	1,7611	1,826	0	-20,511
-18,875	0,83536	0,993	2,0267	1,999	1,7611	1,826	0	-20,511
0,020961	0,27221	0,408	0,99794	1,057	1,1915	1,289	0,01	-2,9031
0,61402	0,43063	0,606	0,9432	0,997	1,0881	1,177	0	-1,391
-0,25655	0,7791	1,011	1,0279	1,095	0,96753	1,066	0	0,44193
0,49645	0,77532	1,233	0,91431	1,017	1,0205	1,185	0	-0,1779
-0,85461	1,0143	1,356	1,1194	1,21	1,0138	1,153	0	-0,1393
-0,81968	0,42833	0,561	1,0789	1,139	1,0347	1,136	0	-0,47735
-0,16655	0,98032	1,232	1,0192	1,09	0,98614	1,102	0	0,16418
1,2796	0,22805	0,369	0,82888	0,904	0,91022	1,045	0,33	0,8908
0,078042	0,5589	0,658	0,99526	1,031	1,0149	1,074	0	-0,3377
5,8174	0,03799	0,071	0,62316	0,654	1,0172	1,082	1	-0,35773
-0,19908	0,73728	1,441	1,0544	1,234	0,84731	1,119	0	0,78234
0,03899	0,43266	0,629	0,99583	1,061	0,97611	1,079	0	0,31343
0,55123	1,013	1,446	0,88074	1,009	0,95679	1,198	0	0,25755
0,54939	0,29874	0,59	0,88294	1,009	0,98604	1,232	0,05	0,082714
1,0836	0,55477	0,941	0,77945	0,893	1,002	1,224	0	-0,01286
0,83034	0,37972	0,663	0,85439	0,955	0,87389	1,049	0	0,96614
0,84864	0,20896	0,478	0,84261	0,949	1,0313	1,233	0,5	-0,22574
2,3082	0,16259	0,42	0,55995	0,651	0,90605	1,097	0,8	0,67636
-0,7464	1,0143	1,32	1,0977	1,181	1,0714	1,211	0	-0,73373
1,6926	0,18849	0,277	0,88208	0,921	1,0113	1,077	1	-0,23179
-0,6613	1,2913	1,692	1,1105	1,219	1,1352	1,326	0	-1,0652
5,5737	0,006653	0,032	0,58688	0,621	1,002	1,079	1	-0,03512
0,80795	0,21788	0,323	0,91594	0,976	1,0759	1,189	0,65	-0,94999
0,12763	0,8768	1,315	0,97467	1,097	1,107	1,322	0	-0,7499
0,57017	1,405	1,888	0,90166	1,003	0,93443	1,105	0	0,52789
0,60604	0,86177	1,535	0,87469	0,996	0,85433	1,034	0	1,08
4,2089	0,092848	0,162	0,59444	0,64	0,96025	1,061	1	0,53073

-1,2623	0,77441	1,015	1,1818	1,278	1,1685	1,333	0	-1,54
-1,2623	0,77441	1,015	1,1818	1,278	1,1685	1,333	0	-1,54
-0,23317	0,59172	0,795	1,029	1,106	1,0219	1,156	0	-0,22835
1,5491	0,14342	0,205	0,89209	0,932	1,0561	1,128	1	-1,0734
1,25	0,69568	0,873	0,86497	0,926	1,0732	1,195	0	-0,8592
2,4744	0,053282	0,138	0,72124	0,78	0,89527	1,006	1	1,2396
0,64507	0,5425	1,019	0,85883	0,987	0,97211	1,191	0	0,18201
1,1828	0,71313	0,99	0,80911	0,899	0,85333	1,019	0	1,1708
-2,6561	1,3063	1,798	1,3435	1,435	1,3057	1,436	0	-3,6483
-2,1497	1,0262	1,591	1,2817	1,372	1,4316	1,572	0	-5,0162
0,06157	0,59135	0,898	0,99278	1,064	1,0893	1,199	0	-1,1591
0,32837	0,61956	0,941	0,96029	1,033	0,86251	0,967	0	1,6944
0,32837	0,61956	0,941	0,96029	1,033	0,86251	0,967	0	1,6944
0,27844	0,73969	0,93	0,96749	1,037	1,031	1,165	0	-0,32625
1,347	0,18762	0,591	0,75458	0,854	1,0289	1,214	0,53	-0,22518
-0,40856	0,93918	1,378	1,0743	1,191	1,1982	1,407	0	-1,4724
1,7176	0,43607	0,917	0,6455	0,751	1,1219	1,333	0,01	-0,87228
3,309	0,14332	0,301	0,54482	0,608	0,93015	1,068	0,99	0,68558
0,83143	0,10659	0,275	0,88172	0,964	0,99685	1,138	0,99	0,031066
-0,47593	0,96078	1,383	1,0811	1,19	1,0779	1,251	0	-0,65713
0,88624	0,7041	0,977	0,87341	0,956	1,0845	1,242	0	-0,78153
0,67613	0,44095	0,796	0,88582	0,985	0,95769	1,128	0	0,34492
-0,76099	0,082553	0,174	1,0779	1,142	1,1356	1,246	1	-1,7776
1,6595	0,29777	0,52	0,73536	0,82	1,0637	1,236	0,02	-0,53639
1,7901	0,38627	0,539	0,84879	0,896	0,84307	0,92	0	2,5527
2,6549	0,36024	0,711	0,74543	0,796	0,74388	0,825	0,01	3,7202
0,13485	0,62766	1,017	0,97305	1,096	0,97826	1,174	0	0,1574
0,13485	0,62766	1,017	0,97305	1,096	0,97826	1,174	0	0,1574
0,13485	0,62766	1,017	0,97305	1,096	0,97826	1,174	0	0,1574
0,13485	0,62766	1,017	0,97305	1,096	0,97826	1,174	0	0,1574
0,13485	0,62766	1,017	0,97305	1,096	0,97826	1,174	0	0,1574
0,13485	0,62766	1,017	0,97305	1,096	0,97826	1,174	0	0,1574
0,13485	0,62766	1,017	0,97305	1,096	0,97826	1,174	0	0,1574
0,13485	0,62766	1,017	0,97305	1,096	0,97826	1,174	0	0,1574
0,13485	0,62766	1,017	0,97305	1,096	0,97826	1,174	0	0,1574
0,13485	0,62766	1,017	0,97305	1,096	0,97826	1,174	0	0,1574
0,13485	0,62766	1,017	0,97305	1,096	0,97826	1,174	0	0,1574
0,13485	0,62766	1,017	0,97305	1,096	0,97826	1,174	0	0,1574
0,13485	0,62766	1,017	0,97305	1,096	0,97826	1,174	0	0,1574
0,13485	0,62766	1,017	0,97305	1,096	0,97826	1,174	0	0,1574
0,13485	0,62766	1,017	0,97305	1,096	0,97826	1,174	0	0,1574
0,13485	0,62766	1,017	0,97305	1,096	0,97826	1,174	0	0,1574
0,13485	0,62766	1,017	0,97305	1,096	0,97826	1,174	0	0,1574
0,13485	0,62766	1,017	0,97305	1,096	0,97826	1,174	0	0,1574
1,1784	0,58586	1,156	0,69776	0,837	0,80532	1,052	0	1,065
4,5522	0,45821	0,569	0,68625	0,721	0,91865	0,981	0	1,6891
2,6835	0,1356	0,285	0,70096	0,758	0,9207	1,022	0,99	1,0292
2,9063	0,068357	0,177	0,69898	0,752	0,87672	0,973	1	1,6494
3,1112	0,15314	0,253	0,65506	0,71	1,0521	1,17	1	-0,62509
0,81695	0,56983	0,836	0,88285	0,966	0,9821	1,132	0	0,16558

0,62544	0,40009	0,698	0,89004	0,993	0,99766	1,192	0	0,017157
2,2208	0,13701	0,18	0,87595	0,907	0,98206	1,037	1	0,42973
2,6191	0,19122	0,602	0,56768	0,646	1,0481	1,21	0,51	-0,42769
0,28987	0,23428	1,101	0,92315	1,087	0,87013	1,094	0,33	0,79677
-0,02996	0,62084	0,732	1,002	1,043	1,0735	1,144	0	-1,4389
0,68759	0,15954	0,288	0,91737	0,988	1,0054	1,129	0,99	-0,06005
0,78471	0,39619	0,744	0,85283	0,961	0,95266	1,13	0	0,37163
0,23419	0,43863	0,589	0,98013	1,031	0,97842	1,057	0	0,3678
1,4012	0,55413	1,263	0,8078	0,884	0,79562	0,909	0,01	2,2497
1,0229	0,23238	0,489	0,83756	0,928	1,0409	1,192	0,31	-0,38627
-0,50279	0,91378	1,37	1,0857	1,195	1,2485	1,422	0	-2,225
-2,967	1,1392	1,469	1,3693	1,458	1,6094	1,747	0	-7,6178
0,73492	0,048874	0,154	0,90215	0,98	1,1011	1,246	1	-1,0137
-5,6082	0,58331	0,674	1,3309	1,371	1,2774	1,343	0	-6,3404
-0,35823	0,77209	1,651	1,0809	1,228	1,1419	1,379	0	-0,92167
1,2741	0,32184	0,533	0,80464	0,89	1,02	1,182	0	-0,17561
0,40858	0,7164	0,936	0,95831	1,019	1,0998	1,199	0	-1,4395
4,7595	0,15207	0,274	0,47216	0,519	0,99679	1,104	1	0,041152
2,3735	0,24225	0,411	0,75308	0,808	0,82271	0,909	0,16	2,5572
0,94464	0,097174	0,192	0,90952	0,965	1,0797	1,172	1	-1,2191
0,54689	1,0555	1,576	0,87771	1,01	0,93349	1,159	0	0,41566
-0,52133	0,29505	0,469	1,0539	1,118	1,0113	1,11	0	-0,15788
-0,52133	0,29505	0,469	1,0539	1,118	1,0113	1,11	0	-0,15788
-1,1654	1,0147	1,788	1,2041	1,323	1,1037	1,302	0	-0,77973
0,6665	0,63632	1,031	0,85985	0,983	0,88612	1,088	0	0,77259
3,723	0,042263	0,109	0,63081	0,679	1,0239	1,128	1	-0,31584
0,87845	0,83614	1,072	0,88706	0,961	1,0719	1,212	0	-0,73639
-0,5599	0,8161	1,148	1,0757	1,161	1,1898	1,326	0	-2,0927
0,14814	0,76526	1,112	0,97403	1,081	0,96281	1,148	0	0,28103
0,76762	0,040116	0,19	0,90322	0,976	1,0454	1,166	1	-0,53166
-0,16031	0	1,696	1,0361	1,18	0,99373	1,218	0,4	0,040477
0,15755	0,59411	0,819	0,97941	1,058	1,0293	1,157	0	-0,32274
-2,0111	0,88638	1,175	1,2785	1,374	1,0237	1,165	0	-0,23565
1,2607	0,38641	0,674	0,77415	0,873	0,92579	1,116	0	0,53888
1,0911	0,22646	0,344	0,8785	0,942	0,95254	1,065	0,37	0,56954
0,17215	0,70898	1,127	0,96404	1,093	1,1066	1,352	0	-0,66188
0,48147	0,46823	0,759	0,93091	1,016	0,91206	1,047	0	0,86865
-1,4503	0,79534	1,145	1,1832	1,267	1,159	1,29	0	-1,7987
0,21712	0,10792	0,34	0,96827	1,057	0,99036	1,123	0,95	0,10043
0,35802	0,84051	1,246	0,93041	1,048	1,1139	1,327	0	-0,80489
2,0492	0,39072	0,512	0,81798	0,867	1,073	1,162	0	-1,1447
0,51248	0,57157	0,909	0,91235	1,014	0,92654	1,099	0	0,58473
0,66458	0,60686	0,867	0,92913	0,991	1,0489	1,151	0	-0,66759
2,7751	0,082591	0,213	0,57883	0,651	0,8768	1,026	1	1,0946
1,418	0,20135	0,52	0,66306	0,788	0,84973	1,106	0,51	0,81047
1,2839	0,38917	0,631	0,78075	0,875	0,88693	1,065	0	0,85983
1,0333	0,6253	1,091	0,73438	0,877	1,0821	1,387	0	-0,41613
-0,06119	0,68788	1,045	1,0106	1,119	0,94537	1,116	0	0,4417
0,50219	0,6988	1,006	0,9189	1,015	0,95149	1,126	0	0,38504

0,65083	0,32159	0,58	0,88537	0,989	1,0838	1,27	0,01	-0,66376
0,1799	0,28969	0,911	0,96752	1,078	0,96447	1,129	0,21	0,29966
-0,00625	0,2181	0,41	1,0007	1,071	1,229	1,349	0,47	-2,8778
-0,26465	0,42708	1,32	1,0885	1,316	1,242	1,61	0,09	-1,0929
-0,29522	0,41521	0,704	1,0418	1,13	1,3339	1,474	0	-3,7545
-0,29522	0,41521	0,704	1,0418	1,13	1,3339	1,474	0	-3,7545
0,71155	0,66759	0,885	0,92584	0,987	0,99909	1,104	0	0,011922
0,5694	0,85222	1,278	0,88537	1,004	0,89651	1,1	0	0,69798
1,6105	0,34479	0,452	0,85882	0,908	1,0916	1,184	0	-1,4046
0,53968	0,58039	0,717	0,96197	1,003	1,0084	1,078	0	-0,16322
2,5818	0,12359	0,21	0,79727	0,839	1,0637	1,135	1	-1,2439
0,13132	0,59214	0,941	0,97784	1,081	0,98291	1,15	0	0,1434
-0,09001	0	1,839	1,02	1,162	0,822	1,038	0,33	1,1021
0,38951	0,66234	1,096	0,91522	1,047	0,90351	1,127	0	0,60246
-0,50716			1,1093	1,252	1,2759	1,514		-1,8614
0,52005	2,2216	1,957	0,85256	1,022	0,66293	0,904	0	1,7525
0,06994	0,68092	1,277	0,98442	1,124	0,9897	1,236	0	0,060911
0,9635	0	0,312	0,83584	0,933	1,2538	1,434	0,95	-2,1969
0,19299	0,53111	1,116	0,96856	1,068	1,0699	1,223	0	-0,65929
0,19299	0,53111	1,116	0,96856	1,068	1,0699	1,223	0	-0,65929
0,19299	0,53111	1,116	0,96856	1,068	1,0699	1,223	0	-0,65929
0,35945	0	1,213	0,85234	1,11	0,87511	1,39	0,54	0,37838
2,0202	0,075506	0,238	0,63122	0,723	0,99923	1,186	1	0,005864
-0,57668	0,64181	0,942	1,0978	1,208	0,9519	1,119	0	0,39901
0,81805	0,31561	0,479	0,90165	0,971	1,18	1,307	0	-2,112
1,024	0,30814	0,448	0,882	0,948	1,0416	1,162	0	-0,48569
0,098148	0,62717	0,802	0,99011	1,051	1,0626	1,166	0	-0,85125
0,17042	0,67757	0,946	0,97782	1,056	0,90391	1,028	0	1,0267
1,1717	0,70646	0,911	0,86364	0,93	0,82256	0,929	0	2,0974
0,18457	0,99021	1,33	0,97311	1,061	1,0561	1,201	0	-0,55242
-0,29475	0,7953	1,029	1,0348	1,108	0,99343	1,105	0	0,080652
1,3105	0,49218	0,683	0,84713	0,913	1,0915	1,213	0	-1,0846
-0,62541	0,85468	1,84	1,1965	1,418	1,3297	1,684	0,03	-1,5735
0,39878	0,85843	1,207	0,94551	1,027	0,8659	0,987	0	1,433
-1,378	1,2267	1,43	1,1474	1,217	1,0163	1,131	0	-0,19662
2,0107	0,1638	0,265	0,7904	0,847	1,1781	1,296	1	-2,2339
-2,0244	1,6726	1,952	1,5314	1,735	1,2644	1,565	0	-1,4438
-0,05527	0,45996	0,708	1,0075	1,091	0,93912	1,071	0	0,62271
0,13485	0,62766	1,017	0,97305	1,096	0,97826	1,174	0	0,1574
1,1122	0,30624	0,553	0,80891	0,905	0,87688	1,043	0,02	0,99136
-0,84091	1,0931	1,326	1,0898	1,157	1,2188	1,328	0	-3,0172
-2,7301	0,48188	0,905	1,4053	1,513	1,5209	1,705	0	-4,8227

-0,58967	0,66993	0,892	1,0749	1,155	1,004	1,13	0	-0,04376
0,98203			0,74438	0,89	0,94033	1,202		0,32809
2,4487	0,5104	0,775	0,60409	0,683	0,99873	1,17	0	0,010472
-1,1816	0,95848	1,348	1,2029	1,319	1,0336	1,225	0	-0,25437
-0,3906	0,76324	1,236	1,0959	1,258	0,90774	1,164	0	0,51065
0,59266	0,67954	1,225	0,85434	1	1,2214	1,52	0	-1,1992
-6,4321	1,6423	1,939	2,1675	1,997	1,8568	1,977	0	-6,3491
0,41765	1,022	1,565	0,92367	1,033	1,0963	1,285	0	-0,75509
0,58685	0,42568	0,84	0,90669	1	1,0201	1,168	0,01	-0,19144
-0,09742	0,73137	1,085	1,0141	1,104	1,1004	1,247	0	-0,99549
-2,2334	0,90035	1,142	1,2626	1,343	1,1585	1,285	0	-1,8444
-0,81106	0,69176	1,533	1,1323	1,239	1,0723	1,245	0	-0,6085
0,40167	0,49232	0,783	0,93744	1,03	0,92785	1,08	0	0,64228
0,080754	0,7738	1,504	0,97817	1,149	1,1001	1,399	0	-0,51969
0,16373	0,89949	1,551	0,96069	1,11	0,92188	1,152	0	0,47859
-0,03232	1,0479	1,664	1,0096	1,203	1,0615	1,391	0	-0,28923
0,8318	0,44953	0,728	0,87388	0,961	1,1121	1,26	0	-1,1083

homADg_A	2P_comple	G2P_flag	2P_gene_r	Informativ	ting_variation
0,000424	monoalleli	monoallelic=HET		1	rs782199287&COSV63110537
0,000354	monoalleli	monoallelic=HET		1	rs782555592
	monoalleli	monoallelic=HET		1	
	biallelic=H	biallelic=HET		1	rs863225206&CD160096
0,000265				1	rs774058645
	monoalleli	monoallelic=HET		1	
	monoalleli	monoallelic=HET		1	rs1394628236
				0,97	
0,000925				1	rs753063356&COSV105266871
0,00126				1	rs758668472&COSV105266872
0,000255				1	rs1010528390
0,000279				0,25	rs1379680071
0,000223				0,981	rs747048024
0,000204				0,929	rs532438179
0,002228				0,938	rs368279169
0,00014				0,852	rs549915078
0,008141				0,868	rs201399108
0,005146				0,881	rs3138597
0,00178				0,978	rs567914276
0,001849				1	rs281865162
0,00264				0,925	rs66781921
0,001433				0,848	
0,003449				0,98	rs368238057
0,005331	monoalleli	monoallelic=HET		0,882	rs113070757
0				1	rs1476860001
0,009107				0,917	rs572979647
0,007726				0,897	rs538753327
0,002352				0,905	rs371085676
0,000786				0,833	rs587611810
	biallelic=H	biallelic=HET		0,962	rs768242944
0,004012				0,833	rs760461308
0,000467				0,958	rs529151539
0,005466				0,904	rs1424367661
0,00622				1	rs72636304
2,79E-05		biallelic=HET		1	rs772547053
3,49E-05				1	rs778203361
0,000503				1	rs1460806637&COSV65999714
5,59E-05				1	rs745676601&COSV64923713
0,00483				1	rs41273179
0,000733				1	rs201370343&COSV99232751
0,000286				1	rs149200521
0,004106				1	rs150059028&CM076475
				1	
0,001633				1	rs142528167&COSV65952319
1,4E-05				1	rs1012321279
0,000161				1	rs746181988
0,00155				1	rs114486361&COSV63602203
0,001187				1	rs1620075&COSV63484437&COSV6348561
0,000175				1	rs145432811

1,4E-05			1	rs1316013974&COSV60727534
0,005968			1	rs34160527
0,005859			1	rs148724953&COSV99038115
			1	rs1013226851
0,00014			1	rs773027446
0,0073			1	rs61481107
3,49E-05			0,976	rs745804849
2,09E-05	monoalleli	monoallelic=HET	1	rs782169028
0,002073			1	rs2289361
0,000805			1	rs1375027500
0,000174			1	rs143005306
			1	
0,002396			1	rs200444708&COSV100913353
0,004045			1	rs41266132&COSV104425370
			1	COSV57844335
0,004481			1	rs139248801&COSV100870502
0,000426			1	rs148727257&COSV59352711
0,002002			1	rs17851570&COSV100929506
0,000391			1	rs1338927825&COSV63124943
0,00426			1	rs111928000&COSV100146440
			1	rs747412993
0,006289			1	rs17514281&COSV99054562
0,004207			1	rs139367209&COSV54126719
0,000727			1	rs61818162
			1	rs1014119141
0,003266			0,982	rs17850752
			1	COSV65536285
6,98E-06		biallelic=HET	1	rs1159903856
			1	COSV55295114
0,000314			1	rs199933567
4,89E-05			1	rs56021741
0,008345			0,958	rs36017138&COSV99053310
			1	rs772511456&COSV55103178
			1	rs763784378
0,002541			1	rs142036127
		biallelic=HET	1	
			1	
			1	rs765845646
0,009275			1	rs16937417
6,98E-06			1	rs1228601763
0,00201			0,98	rs75026444
0,005266			1	rs200437064
0,000517			1	rs145519212
4,21E-05			1	rs763971624
0,000447			1	rs143399526
			1	
0,002777			1	rs61751497
0,008138			1	rs117111102&COSV99609144
0,004596			1	rs71482397
0,003009			0,974	rs117861052&COSV99059282

2,09E-05			1	rs752346792
			1	
0,005402			0,967	rs61757087&COSV100740083
9,77E-05			1	rs200306295&COSV53266481
0,001283			1	rs2308318&COSV60033492
0,002527			1	rs41282900
4,19E-05			0,979	rs750015284&COSV58307733
0,001256			1	rs549781167
0,004564			1	rs148086757&COSV58084459&COSV58084
			1	
0,004826			1	rs199751579&COSV54247021
0,008783			1	rs200922487
0,002233			1	rs182144037&COSV52759509
			1	rs12812&COSV67413625
			1	rs190063&COSV68307176
3,49E-05			0,95	rs201475245&COSV54345060
6,98E-06			1	rs545574170
			1	
0,0089			1	rs149500920
0,003009			1	rs148069776&COSV100564276
			1	
	monoalleli	monoallelic=HET	1	
0,003845			1	rs146342750
0,000161			1	rs1219681191
1,4E-05			1	rs139771656
0,000189			1	rs140238243&COSV56907509
6,98E-06			1	rs1404419362
0,000551			1	rs200828887&COSV63138893
0,007111			0,984	rs74818906
			1	rs199873313&COSV60803252
4,19E-05		biallelic=HET	1	rs758139447&CM1615052&COSV5914713
1,4E-05			1	rs765440889&COSV53735663
6,98E-06			1	rs764985441&COSV56935085
6,98E-06			1	rs550019697&COSV99581049
0,00633			1	rs138835109&COSV68006828
0,007993			1	rs35877321&CM1414170&COSV100392667
0,001689		biallelic=HET	1	rs138544311&COSV99598298
9,77E-05			1	rs749096070
0,000112		biallelic=HET	1	rs192622083
			1	
0,001703			1	rs143878003
0,001082			1	rs147840657
6,97E-06			1	rs776639191&COSV56925819
0,007704			1	rs118084065
0,001556			1	rs11054243&COSV54394955
0,001934			1	rs140825288&COSV53702556
0,000293			1	rs200263951
0,001131		biallelic=HET	1	rs61753359&COSV56783267
			1	
6,98E-06			1	rs748642294

0,000293		1	rs74484201&COSV105905426&COSV60130[illegible]
0		1	rs201846944&COSV104649365
0,004728		1	rs138988810&COSV52882258
0,002388	biallelic=HET	1	rs56017157
2,13E-05		1	rs145406233
0,005228		1	rs61756413&COSV61025282
3,52E-05		1	rs759316816
		1	rs1419383429
6,98E-06		1	rs1320983317
0,0003		1	rs141186779
0,009961		1	rs61732395&COSV57446900
0,000496		1	rs201559885&COSV57409422
0,005787		1	rs61745785
6,98E-06	biallelic=HET	1	rs759097408
0,001096		1	rs191481498
0,000112		1	rs201812068
3,49E-05		1	rs151327434&COSV104585178
	biallelic=HET	1	
0,008187		1	rs11571769&CM970186&COSV66463542
0,000921		1	rs141436576
0,000488	biallelic=HET	0,98	rs146993892&COSV66284048
0,000475		1	rs141717634
		1	rs756344666
0,008553		1	rs141152619
0,007282		1	rs113380635
0,00824		1	rs199518800&COSV73703668
0,005191		1	rs59543367
0,00241		0,957	rs141923373
0,000873		0,971	rs140326965&COSV104404946
		1	rs753516978
0,004635		1	rs35064097
2,09E-05		0,974	rs764138536
		1	rs1206268063
6,98E-06		1	rs745589833&COSV105930974
0,008006		1	rs45462994
0,00021		1	rs201727567
0,001983		1	rs199996727&COSV62068564
0,00515		1	rs143429892
0,008532		1	rs118054948
0,000461		1	rs201434885&COSV99048410
0,005225		1	rs199550173
0,002233		1	rs200601510
0,000718		1	rs150446570&COSV60906986
0,001032		1	rs201395333
0,005415		1	rs199870471
0,000176		1	rs367600428
		1	
0,002036		1	rs527825185&COSV68625106
0,001064		1	rs1349996435
1,24E-05		1	rs1196515885

0,005818			1	rs148331737&COSV100309010
0,00014			1	rs781573282
0,002254			1	rs34623235
0,002951			1	rs116939379&COSV99961946
5,58E-05			1	rs780359042&COSV55564717
0,001152			1	rs138595084
0,003512			1	rs146460562&COSV51211324
1,4E-05			0,969	rs141361666&COSV100551043
0,007763			1	rs754738318
0,006336			1	rs117500630&COSV59585187
			1	
0,003087			1	rs199809872
			1	
6,28E-05			1	rs761389709
0,000112		biallelic=HET	1	rs142753188
			0,978	rs748724962
0,002972			1	rs147948209
0,001822			1	rs181399062
0,000154			1	rs104895157&CM060336&COSV104376124
0,000112			1	rs104895081&CM981242
2,79E-05			1	rs767541693
0,005187			1	rs145995713&COSV105871226
1,4E-05			1	rs767282296&COSV54113501
0,00118			0,95	rs137963033
6,28E-05		biallelic=HET	1	rs771128441&COSV55543719
0,003555			1	rs139748181
0,000489			1	rs139688634
0,006098			1	rs117470111&COSV54532872
3,49E-05			1	rs377571984
0,002225			1	rs1227499913
0,000475			1	rs144118842
			1	rs777178441
0,000126	monoalleli	monoallelic=HET	1	rs757169483
0,004867			1	rs34017457
		biallelic=HET	1	rs1423470729
0,000287	biallelic=H	biallelic=HET	1	rs369490320&COSV59252204
6,98E-06			1	rs757444755
6,28E-05			1	rs375181581&COSV105865985
			1	
1,4E-05			1	rs776186248
2,1E-05	biallelic=H	biallelic=HET	1	rs1286773151
2,09E-05	biallelic=H	biallelic=HET	1	rs753781703&COSV56336072
1,4E-05		biallelic=HET	1	rs1056843521
1,4E-05			1	rs757774192
			1	rs765150202
0,001794			1	rs149540181&COSV99036584
4,18E-05			0,982	rs762227785
0,000168			1	rs561474067&COSV99236843
			1	rs943371729
0,006387			1	rs139011011

6,98E-06		biallelic=HET	1	rs370836663
0,001814			1	rs200614314&COSV52375603
6,28E-05			1	rs370823173&COSV58804563
6,98E-06			1	rs746765101
0,000971			0,65	rs1407887023&COSV61565257
0,002472			1	rs4890107&COSV66940098
0,008589			1	rs200210041&COSV53190080&COSV99512
4,88E-05			0,984	rs372181383&COSV105114551
			0,97	COSV50364130
3,67E-05			1	rs779904537
0,002311		biallelic=HET	1	rs201619500&COSV99836882
0,00418			1	rs12452285&COSV52224464&COSV522255
0,004493			1	rs78349823
0,000279			1	rs139045226
2,09E-05			1	rs137872239&COSV53943968
0,000537			1	rs752007056
0,001431			1	rs148536942
0,002087			0,966	rs145164093&COSV105188607
	monoalleli	monoallelic=HET	1	
1,4E-05			1	rs754726574
0,002193			1	rs139451924
0,000126		biallelic=HET	1	rs146579468
0,009886			1	rs113009055&COSV71765613
2,09E-05			0,985	rs991571797
			1	rs1184924905
0,000207			1	rs199944112&COSV56693029
0,002695			1	rs75031432&COSV104618166&COSV56688
			1	rs542384152
0			1	rs12327801&COSV99040916
0,007432			1	rs151025018
0,002331			1	rs143838272&COSV105823874
4,44E-05			1	rs59002457&COSV50220330
0,007398			1	rs74406884
0,000223			1	rs372686960&COSV99449730
0,003315			0,981	rs147025508
1,4E-05			1	rs1437691578
0,009378			1	rs151335087&COSV52230973
0,001529			1	rs61731609&COSV53242266
1,4E-05	monoalleli	monoallelic=HET	1	rs745399869&COSV55874206
0,00113			1	rs145639224
6,98E-06			1	rs200740823
0,0049			1	rs35358946&COSV99040893
0,006283			1	rs7253392&COSV68093598
0,007331			1	rs142017118
0,002199		biallelic=HET	1	rs138173172&CM044682&COSV62287001
2,79E-05			1	rs756859794
0,009051			1	rs71352251
0,004386			1	rs550654047
0,001864			1	rs140908383
7,4E-06			1	rs112003183

0,006982			1	rs369311910
0,008795			1	rs201231416
0,007222			1	rs138253216&COSV99070084
0,001879			1	rs182252011
0,00014			1	rs141307342&COSV67477104
0,005393			1	rs147066927&COSV53244501
0,001271			1	rs183882477
0,000698		biallelic=HET	1	rs146282777&COSV53026188
0,000677			1	rs151018053&COSV101427719
			1	rs141665993
0,007893			1	rs80263019
		biallelic=HET	1	rs141384427
6,98E-06			1	rs774467569&COSV58310813
			1	rs779243938
0,00023			1	rs199723193&COSV52478350
0,000384			1	rs201648655&COSV57816707
0,008011			1	rs41286003
0,007212			1	rs112166113
			0,967	
0,006343			1	rs202239738
7,01E-06			1	rs774762527
			1	rs772982333
0,000643			1	rs533236279&COSV71575972
0,002988			1	rs4588203
0,005562			1	rs4380247
0,00628			1	rs61735719
0,006351			1	rs116775947
0,007244			1	rs35707762&COSV51464913
0,00231			1	rs79766504&COSV52038861
2,09E-05		biallelic=HET	1	rs776721597
0,000999			1	rs34110122&COSV59833823
0,003036			1	rs148495956&COSV100826260
1,4E-05		biallelic=HET	1	rs755935124
2,09E-05			1	rs750004862
0,007384			1	rs11546386&COSV51909661
0,009445			1	rs114709725&COSV99069355
	monoalleli	monoallelic=HET	1	rs781349703
		biallelic=HET	1	
6,98E-05			1	rs770663524
0,00663			1	rs115927138&COSV99068049
0,004157			1	rs144338745
			1	
0,002724			1	rs144884167&COSV104418793
0,009052			1	rs112232806&COSV59054960&COSV99063
0,000865			1	rs200102286&COSV55769028
0,000112			1	rs199604949
0,000335			1	rs200529234
0,003641			1	rs144708311
0,001863			1	rs115926000
0,000621			1	rs139644193

			1	
0,004207			1	rs45565839&COSV53348489
0,008394			1	rs141953885&COSV99071510
0,000363			1	rs369261479
6,98E-06			1	rs1174849872
			1	
0,000167			0,952	rs770645834
			1	
0,000201			1	rs1805127&CM062783&COSV61606368
0,002793			1	rs143714922&COSV104707110
1,4E-05			1	rs140630794&CM151126
			1	rs3746951&COSV54259059
			1	rs2020945&COSV52378282
0			1	rs968714&COSV52383962
			0,98	rs11558819&COSV60263467
			1	rs7354779&CM103985&COSV54263126
0,000831			1	rs2930761
0,000935			1	rs199768894
0,002813			1	rs450046&CM057943&COSV58230786
	monoalleli	monoallelic=HET	1	rs1458962515
0,00485			1	rs142442293
		biallelic=HET	0,978	rs1205842915
0,000188			1	rs374168516&COSV58504382
0,004322			1	rs35783914&COSV99041655
0,00977			0,972	rs61742274
0,005099			1	rs112587316
0,008229			1	rs35968360&COSV99326283
0,003135			1	rs74530943
4,19E-05			1	rs926916292
			1	
0,00709			1	rs547352394
0,005316			1	rs149696716
0,00211			0,929	rs62001863&COSV53416845
			1	rs769030029
0,004271			1	rs201517941&COSV56004845
1,39E-05			0,983	rs763641214
0,000719			1	rs146998254&COSV105070209
2,79E-05			1	rs766875593&CM143812
0,003545			0,972	rs143591096&COSV100565088
			1	
2,79E-05			1	rs144181784&COSV100015192&COSV1046
			1	rs1235575951
0,0024		biallelic=HET	1	rs144765752
1,4E-05			1	rs148694532
0,001703			1	rs150999288
0,000342			1	rs377541202&COSV100401952
0,001836			1	rs41292856
0,001752			1	rs34216132
0,00109			1	rs1008564972
1,4E-05			1	rs529341271

2,09E-05			1	rs1047629384
0,000642			1	rs144221103
0,008682			1	rs147762157&COSV58724425
0,00615			0,941	rs144385283&COSV61471920
0,009578			1	rs114420009&COSV61471930
0,004217			0,969	rs116187304&COSV104594149
5,58E-05			1	rs764067765
2,09E-05			1	rs199668779&COSV59856809
0,007467			1	rs145346983&COSV51572899
0,002676			1	rs754539084
0,000148			1	rs138900382
0,002673			1	rs114608303&COSV57795700
0,001428			0,968	rs11917433&COSV57781428
0,007655			1	rs146525749
0			0,875	rs779246178&COSV57762866&COSV57804
0,000268			0,933	rs76889457&COSV62131362
0,004245			1	rs74420943&COSV62137934
0,000255			0,933	rs76839144&COSV62133477
0,001665			0,933	rs71291868&COSV62133492
0,001419			0,963	rs75075507&COSV62133546
0,000105			1	rs147175932
0,001158			1	rs151194139
0,000586			1	rs138418678&COSV53453054
0,008288			1	rs111620813&COSV104600458
0,000837			1	rs149894820
2,1E-05			1	rs753347164&COSV100394049
0,002366			1	rs144722161
0,008025			1	rs61747658&COSV72230579
			1	
6,98E-06			1	rs763313965
0,005795			1	rs61729366&CM1415421&COSV53626039
			1	rs201478367&COSV55698988
0,003505			1	rs114816312&COSV99697401
6,98E-06			0,96	
0,000678			1	rs145513784&COSV56638641
0,000209			1	rs138246731
0			1	rs757017032
			1	rs778568230
0,000307			1	rs200182587
2,79E-05			1	rs200596924&COSV66930696
0,003774			0,875	rs61051796&COSV57001900
0,008999			1	rs34748216&CM1616116&COSV104616713
9,07E-05			1	rs747942546
	monoalleli	monoallelic=HET	1	rs1031384711
		biallelic=HET	1	
2,79E-05			1	rs202194194
6,98E-06			0,966	rs749096342
0,002547			0,968	rs148564433
6,98E-06			1	rs1009249542
6,98E-06	monoalleli	monoallelic=HET	1	rs768256433&COSV99399009

0,000126			1	rs200204316&COSV54882311
0,000516			1	rs199799127
0,004079			1	rs114280473&CM169746
0,008237			1	rs56168072&COSV105107849&COSV52532[illegible]
0,001431		biallelic=HET	1	rs28903086&CM172646&COSV99528782
0,000705	monoalleli	monoallelic=HET	1	rs141068875
8,37E-05			1	rs368323992
0,000468			1	rs199606354
0,002756			1	rs71583630&COSV99177626
5,58E-05			1	rs781950562
0,000789			1	rs764241006&COSV99548723
0,002192			1	rs201901866&COSV99038489
0,007876			1	rs141134077
			0,941	rs940443107
0,009065			1	rs1800888&CM984123
0,000887			1	rs115069685
0,004278			1	rs201026723
			1	
0,003493			1	rs192898913&COSV100286924
		biallelic=HET	1	
0,004705			1	rs34744890&COSV99031628
0,008571			1	rs61734277&CM1413390&COSV99058751
6,98E-06			1	rs1561852844
0,005779			1	rs111739077&COSV99214587
0,002179			1	rs748601560
0,007783			0,97	rs140778074&COSV105942141
0,00011			1	rs1130935&COSV66110395
8,96E-05			1	rs1050105&COSV66110576
9,18E-05			1	rs1130947&COSV66110404
3,34E-05			1	rs146911342&COSV66110360
0,000108			1	rs1050147&COSV66111096
0,000181			1	rs1050180&COSV66110429&COSV661142[illegible]
0,003786			1	rs2308628&COSV66110582
8,96E-05			1	rs1131015&COSV66110591
1,66E-05			0,974	rs41540117&COSV66110904
0,002972			1	rs2308622&COSV66110598
0,000206			1	rs707908&COSV66110239
0,001967			1	rs1050328&COSV66117576
0,001168			1	rs1131096&COSV66109964&COSV6611175
0,003687			1	rs2308592&COSV66109994&COSV661108[illegible]
0,006797			1	rs2308590&COSV66110458&COSV661106[illegible]
0,002027			1	rs2308575&COSV66110483
0,005825			0,972	rs41542423&COSV66111027
0,008318			1	rs1071650&COSV66111042&COSV661122[illegible]
0,000869			0,962	rs9270302&COSV63511399
0,004584			1	rs2229633
			1	COSV65219689
0,007398			1	rs142171063
0,006861			1	rs61739889
0,005089			1	rs35675573

0,008507			1	rs35124644
6,99E-05			1	rs187315105
0,000112			1	rs767810773
0,004772			1	rs138360237
			1	
0,000852		biallelic=HET	1	rs146462599
0,000468			1	rs142296383&COSV61359288
			1	rs747665356
0,001053			1	rs146014542
0,000188			1	rs200186315
0,004969			1	rs61734970&COSV58236633
0,001307			1	rs138917702
0,000174		biallelic=HET	1	rs372517211&COSV105267234
0,001542			1	rs61755968&COSV54125293
0,009336			1	rs34879202&CM140787&COSV60986400
0,009407			1	rs76297889&COSV99069799
6,99E-06			1	rs779720657
1,4E-05			0,978	rs370811034
0,006572			1	rs2230445&COSV56049973
0,006575			0,962	rs61740887&COSV71989394
			1	
0,00841			1	rs111480809&COSV62363436
0,007796			1	rs61739178&COSV99474925
7,68E-05			1	rs201325705
0,004643			1	rs199932939
0,0003	monoalleli	monoallelic=HET	1	rs201862819
			0,962	
0,001873			1	rs148164233&COSV99593305
0,001298			1	rs147045279&COSV105116000
0,00014			1	rs142155405&COSV105030253
2,79E-05			1	rs201240931
2,79E-05			1	rs375188595
0,000112		biallelic=HET	1	rs201666755
0,005961			1	rs55638457&CM011491
4,89E-05			1	rs139029166&COSV50044771
2,79E-05			1	rs1473882802
0,000133		biallelic=HET	1	rs146935906&COSV54338160
0,008549			0,938	rs112190116&CM1615601&COSV10455124
0,000167			1	rs371873525
0,003905			1	rs149140637&COSV56117052
			1	rs766476082
0,008302			0,971	rs36068949
0,001728			1	rs574980735
0,008839			1	rs61746628
0,007087			1	rs193080501&COSV51699634
0,003639			1	rs144435181
4,19E-05			1	rs374332818
2,1E-05			1	rs147994044
2,79E-05			1	rs373575116
			1	

0,000203	monoalleli	monoallelic=HET	1	rs138839833
3,49E-05			1	rs750041896&COSV57524416
0,009607			1	rs11548254&COSV105004554
0,000733			1	rs141421747
0,000133			1	rs993784537
		biallelic=HET	1	rs781958924
	biallelic=H	biallelic=HET	1	rs781897281
			1	rs71509924&COSV57924207
0,009393			1	rs34101674&COSV52658231&COSV526589
0,001038			1	rs75513621
0,000105		biallelic=HET	1	rs142946646&COSV62520097&COSV62521
8,37E-05			1	rs201299987
0,002097			1	rs146411184
	monoalleli	monoallelic=HET	0,972	rs917032910
			1	
0,007327			0,966	rs148203698
			0,737	rs2001031
			0,998	rs2853508
0,007144			1	rs147228278
4,79E-05			1	rs766627568&COSV59133782
			1	rs1215241101&COSV71275891
	x-linked_d	x-linked_dominant=H	1	rs782227114
0,005336			1	rs200727091&COSV66493151
0,004588			1	rs56863346&COSV62387781
0,001624			1	rs2235730&COSV62387660
0,00092			1	rs765984518
0,001372			1	rs144602884
9,4E-06			1	rs780817605
0,003631			1	rs143441217&COSV54956217
4,72E-05	x-linked_d	x-linked_dominant=H	1	rs782644695&COSV60939362
0,000188			1	rs199914402
		biallelic=HET	1	rs769689858
0,003121			1	rs148628311&COSV54573061
0,001598		biallelic=HET	1	rs145720191
0,003336			1	rs142483883&COSV53872252&COSV53873
4,89E-05			1	rs759259465
0,001878			1	rs145707301
0,001103			1	rs56176960&COSV105070971
6,98E-06		biallelic=HET	0,969	rs367717633
0,004395			1	rs144929524
		biallelic=HET	1	
			1	
3,5E-05			1	rs759551723&COSV53504601
0,005716			0,966	rs138265477&COSV59669355
5,59E-05			1	rs143573829&COSV55783689
0,003524			1	rs41556321&COSV66110876
0,001884			0,976	rs147233974
0,005774			1	rs145077934&COSV56032874
0,003119			1	rs1365858952
0			1	rs1368717184

		1	rs1380653968&COSV61206713
0,00382		1	rs782577564
0,008777		1	rs9729175
		1	
2,09E-05		1	rs755573907
0,001159		1	rs138660620
	biallelic=HET	1	rs757662765
0,003249	biallelic=HET	1	rs113993993&CS030125&COSV55887021
0,002218		1	rs191340041
0,00037		1	rs543527800
0,009088		1	rs1158745696
0,000191		1	rs1271530682
2,1E-05		1	rs1022493743
		1	
	biallelic=HET	1	rs786204506&CM042114
0,009277		0,889	rs78205732
0,009811		1	rs143031243
0,004646		1	rs74837809
		1	
0,003952		1	rs202233735&CM092258
0,001263		1	rs41280363&CM1610107
5,59E-05		1	rs201441238&COSV66985543
	biallelic=HET	0,8	

075&COSV99063079
